# Exploring the molecular mechanism of Ling-Gui-Zhu-Gan decoction for the treatment of type 2 diabetes mellitus based on network pharmacology and molecular docking: A review

**DOI:** 10.1097/MD.0000000000033210

**Published:** 2023-03-24

**Authors:** Feng Long, Zhe Zhang, Chunxiu Luo, Xiao Lei, Jinlian Guo, Lin An

**Affiliations:** a Department of Traditional Chinese Medicine, Affiliated Hospital of North Sichuan Medical College, Nanchong, Sichuan, China.

**Keywords:** Ling-Gui-Zhu-Gan decoction, mechanism research, network pharmacology, traditional Chinese medicine, type 2 diabetes mellitus

## Abstract

To investigate the mechanism of action of the classical formula Ling-Gui-Zhu-Gan (LGZG) decoction in treating type 2 diabetes mellitus based on network pharmacology and molecular docking. The active ingredients and targets of LGZG decoction were collected by the Traditional Chinese Medicine Systems Pharmacology Database and Analysis Platform database and mapped using Cytoscape software to show their interrelationships. GeneCards, Pharmacogenomics Knowledge Base, OMIM, Therapeutic Target Database, and Drugbank databases were used to obtain targets related to type 2 diabetes; protein-protein interaction networks were established with the help of the STRING platform. Gene Ontology and Kyoto Encyclopedia of Genes and Genomes analyses were performed on selected core targets with the help of the Metascape platform. Finally, the AutoDock platform was used to perform molecular docking and display the results by Pymol software. One hundred twenty-one active ingredients, 216 effective target genes, 11,277 type 2 diabetes mellitus-related genes, 210 crossover genes, and 18 core genes were obtained for LGZG decoction. The results obtained by Kyoto Encyclopedia of Genes and Genomes indicated that the advanced glycosylation end products-receptor of advanced glycosylation end products signaling pathway, the phosphatidylinositol 3 kinase-Akt signaling pathway, and HIF-1 signaling pathway might be the key signaling pathways. Molecular docking showed that the binding energy of quercetin, kaempferol, naringenin, and licorice chalcone A to the core target genes were all <5.0 kJ-mol^−1^, with good affinity. In this study, the potential active ingredients and mechanisms of action of LGZG decoction in the treatment of type 2 diabetes were initially investigated, which provided a basis for the in-depth study of its drug basis and mechanisms of action.

## 1. Introduction

Diabetes mellitus is a group of chronic hyperglycemic metabolic diseases caused by defective insulin secretion or utilization, with polydipsia, polyphagia, polyuria, and weight loss as typical clinical manifestations.^[1]^ According to the International Diabetes Federation, the number of people with diabetes is expected to increase to about 700 million by 2045.^[2]^ It has been documented that type 2 diabetes mellitus (T2DM) accounts for about 95% of diabetic patients,^[3]^ making it a global health problem. T2DM is a non-insulin-dependent type of diabetes, which can induce various complications, including diabetic nephropathy and cardiovascular. The clinical treatment of T2DM mainly includes lifestyle and pharmacological interventions. However, the long-term application of simple hypoglycemic drugs is often accompanied by different toxic side effects, such as heart failure and increased risk of fracture in women.^[4,5]^

In contrast, the treatment of T2DM in traditional Chinese medicine (TCM) can make up for the shortcomings of Western medicine through its holistic concept and evidence-based treatment. It can regulate blood glucose by improving pancreatic β-cell function, promoting insulin secretion, and improving insulin sensitivity,^[6,7]^ which has the advantages of less adverse reactions and stable efficacy and is receiving more and more attention from medical practitioners. The etiology of diabetes mellitus belongs to the category of “emaciation-thirst disease” in Chinese medicine. It is generally believed that the etiology of thirst is based on yin deficiency, dryness, and heat. The disease is usually located in the lung, stomach, and kidneys.^[8]^ However, the clinical etiology is complex and varies, and it is not uncommon to have atypical symptoms and spleen deficiency with dampness.^[9–12]^

**Figure 1.**
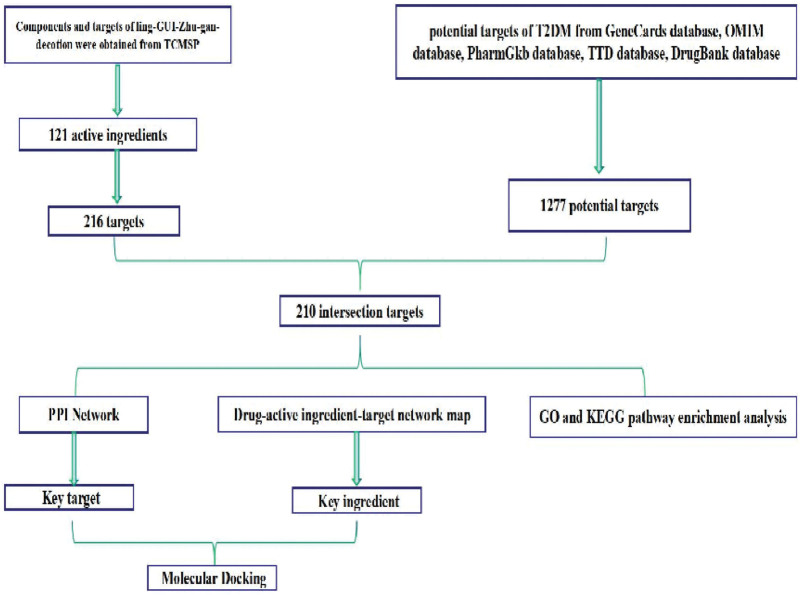
Flow chart of the study.

In this paper, we use network pharmacology and molecular docking methods to predict the core target genes of Ling-Gui-Zhu-Gan (LGZG) decoction for the treatment of T2DM and investigate the potential pathway mechanisms to pave the way for further research. The detailed workflow of this study is presented in Figure 1.

## 2. Materials and Methods

### 2.1. Acquisition of active ingredients and targets of TCM

The chemical composition of “Fu Ling,” “Gui Zhi,” “Bai Zhu,” and “Gan Cao” was retrieved from the database of Traditional Chinese Medicine Systems Pharmacology Database and Analysis Platform (TCMSP) (http://tcmspw.com/tcmsp.php). The chemical compositions of “Fu Ling,” “Gui Zhi,” “Bai Zhu,” and “Gan Cao” were searched. Based on the absorption distribution and metabolic excretion screening conditions, with oral bioavailability ≥ 30% and drug similarity ≥ 0.18, the active ingredients and potential targets of Fu Ling, Gui Zhi, Bai Zhu, and Gan Cao in LGZG decoction were obtained. The obtained compound target genes were entered into the Uniprot (https://www.uniprot.org/) database, normalized, and transformed to find the corresponding gene symbols (standard gene names).

### 2.2. Collection of potential pathogenic targets of diseases

The GeneCards (http://www.genecards.org/), OMIM (http://www.omim.org/), Pharmacogenomics Knowledge Base (https://www.pharmgkb.org), Therapeutic Target Database (http://db.idrblab.net/ttd), and DrugBank (https://www.drugbank.ca) databases for the keyword “type 2 diabetes mellitus.” The search results of the 5 databases were combined, all disease-related genes were merged, and duplicate target genes were removed to obtain the potential pathogenic targets of T2DM.

### 2.3. Venny analysis of intersecting genes

The data of the potentially active compounds and the pathogenic targets of T2DM were mapped into Venny 2.1.0. The intersection was taken, and the “intersection gene Venn map” was created.

### 2.4. “Active ingredient-target” network construction

Based on the obtained active ingredients and intersecting genes, we construct the active ingredient-target network relationship, import the constructed network relationship into Cytoscape 3.8.0 software, and build a network map, with nodes representing active ingredients and action targets respectively; edges are used to connect the nodes and show the connection between active ingredients and action targets. Furthermore, according to the software set graphics, color, character size, and other parameters to build a visual production map.

### 2.5. Construction of protein interaction network and key target screening

Protein-protein interaction (PPI) studies the correlation between compounds and disease-related protein molecules from the perspective of biochemical, signal transduction, and genetic networks. The common target genes of LGZG decoction and T2DM were imported into the STRING database (https://string-db.org/), the study species was limited to “human,” the confidence level was set to 0.9, the free nodes were hidden, and the rest of the parameters were set to default to obtain the PPIs, and exported to Save as TSV format file and save the original image. The resulting files were imported into Cytoscape 3.8.0 software for visualization. The CytoNCA plug-in was used to calculate the Betweenness, Closeness, Degree, Eigenvector, Network, and local average connectivity-based scores for each node. Each node is scored, and the node with a higher score is retained by filtering the value > median value to obtain the core gene of the network.

### 2.6. Gene Ontology (GO) functional analysis and Kyoto Encyclopedia of Genes and Genomes (KEGG) pathway enrichment analysis

To further determine the functions of the intersection target genes and their signaling pathways, set *P* < .05, with the help of the “ggplot2” “stringi” “colorspace” package in R language and the “cluster Profiler” “enrichplot” “DOSE” library in Bioconductor “ggplot2” “stringi” “colorspace” in R language and “clusterProfiler” “enrichplot” and “DOSE” in Bioconductor library; “language package to perform GO enrichment analysis on the intersection genes of LGZG decoction and T2DM, and clarify the mode of action; meanwhile, the KEGG pathway analysis was performed in the above way to clarify the pathway of action, and visualization analysis was performed to obtain cluster plots and bubble plots.

### 2.7. Pharmacophore-target molecular docking validations

The above analysis selected top-ranked active ingredients and core genes, and molecular docking was verified according to whether there was an interaction between the core compounds and the core target genes. 2D structure files of small molecule ligands were downloaded from the PubChem database, and the 2D structure was converted to a 3D structure in ChemOffice software and saved in mol2 format. The pdb format of protein 3D structure was downloaded from the Protein Data Bank database (http://www.rcsb.org), and its water molecules and small molecule ligands were removed by PyMOL software. The resulting small molecule ligand and protein receptor files were converted into PDBQT format, and the active pockets for molecular docking were determined simultaneously. Finally, molecular docking was performed using Vina software.

## 3. Results

### 3.1. Active ingredients and potential targets

As shown in Table 1, after screening in the TCMSP database according to the conditions, a total of 121 active compounds were collected, including 15 active compounds for Fuling, 7 active compounds for Gui Zhi, 7 active compounds for Bai Zhu, and 92 active compounds for Gan Cao. Sitosterol was common to both Gui Zhi and Gan Cao among these active compounds. After searching the corresponding targets in the TCMSP database based on the screened active ingredients, 216 annotated genes were obtained by normalizing the target genes to the UniProt database (as shown in Supplemental File S1, Supplemental Digital Content, http://links.lww.com/MD/I644, which illustrates the related targets and the basic information of all active compounds).

**Table 1:** Active compounds of Chinese medicine.

Mol ID	OB (%)	OB (%)	DL	Source
MOL000273	(2R)-2-[(3S,5R,10S,13R,14R,16R,17R)-3,16-dihydroxy-4,4,10,13,14-pentamethyl-2,3,5,6,12,15,16,17-octahydro-1H-cyclopenta[a]phenanthren-17-yl]-6-methylhept-5-enoic acid	30.93	0.81	Fu Ling
MOL000275	Trametenolic acid	38.71	0.8	Fu Ling
MOL000276	7,9 (11)-Dehydropachymic acid	35.11	0.81	Fu Ling
MOL000279	Cerevisterol	37.96	0.77	Fu Ling
MOL000280	(2R)-2-[(3S,5R,10S,13R,14R,16R,17R)-3,16-dihydroxy-4,4,10,13,14-pentamethyl-2,3,5,6,12,15,16,17-octahydro-1H-cyclopenta[a]phenanthren-17-yl]-5-isopropyl-hex-5-enoic acid	31.07	0.82	Fu Ling
MOL000282	Ergosta-7,22E-dien-3beta-ol	43.51	0.72	Fu Ling
MOL000283	Ergosterol peroxide	40.36	0.81	Fu Ling
MOL000285	(2R)-2-[(5R,10S,13R,14R,16R,17R)-16-hydroxy-3-keto-4,4,10,13,14-pentamethyl-1,2,5,6,12,15,16,17-octahydrocyclopenta[a]phenanthren-17-yl]-5-isopropyl-hex-5-enoic acid	38.26	0.82	Fu Ling
MOL000287	3Beta-Hydroxy-24-methylene-8-lanostene-21-oic acid	38.7	0.81	Fu Ling
MOL000289	Pachymic acid	33.63	0.81	Fu Ling
MOL000290	Poricoic acid A	30.61	0.76	Fu Ling
MOL000291	Poricoic acid B	30.52	0.75	Fu Ling
MOL000292	Poricoic acid C	38.15	0.75	Fu Ling
MOL000296	Hederagenin	36.91	0.75	Fu Ling
MOL000300	Dehydroeburicoic acid	44.17	0.83	Fu Ling
MOL001736	(−)-Taxifolin	60.51	0.27	Gui Zhi
MOL000358	Beta-sitosterol	36.91	0.75	Gui Zhi
MOL000359	Sitosterol	36.91	0.75	Gui Zhi
MOL000492	(+)-Catechin	54.83	0.24	Gui Zhi
MOL000073	Ent-epicatechin	48.96	0.24	Gui Zhi
MOL004576	Taxifolin	57.84	0.27	Gui Zhi
MOL011169	Peroxyergosterol	44.39	0.82	Gui Zhi
MOL000020	12-Senecioyl-2E,8E,10E-atractylentriol	62.4	0.22	Bai Zhu
MOL000021	14-Acetyl-12-senecioyl-2E,8E,10E-atractylentriol	60.31	0.31	Bai Zhu
MOL000022	14-Acetyl-12-senecioyl-2E,8Z,10E-atractylentriol	63.37	0.3	Bai Zhu
MOL000028	α-Amyrin	39.51	0.76	Bai Zhu
MOL000033	(3S,8S,9S,10R,13R,14S,17R)-10,13-dimethyl-17-[(2R,5S)-5-propan-2-yloctan-2-yl]-2,3,4,7,8,9,11,12,14,15,16,17-dodecahydro-1H-cyclopenta[a]phenanthren-3-ol	36.23	0.78	Bai Zhu
MOL000049	3β-Acetoxyatractylone	54.07	0.22	Bai Zhu
MOL000072	8β-Ethoxy atractylenolide III	35.95	0.21	Bai Zhu
MOL004941	(2R)-7-hydroxy-2-(4-hydroxyphenyl)chroman-4-one	71.12	0.18	Gan Cao
MOL001792	DFV	32.76	0.18	Gan Cao
MOL004835	Glypallichalcone	61.6	0.19	Gan Cao
MOL004841	Licochalcone B	76.76	0.19	Gan Cao
MOL004985	Icos-5-enoic acid	30.7	0.2	Gan Cao
MOL004996	Gadelaidic acid	30.7	0.2	Gan Cao
MOL003896	7-Methoxy-2-methyl isoflavone	42.56	0.2	Gan Cao
MOL000500	Vestitol	74.66	0.21	Gan Cao
MOL004957	HMO	38.37	0.21	Gan Cao
MOL004328	Naringenin	59.29	0.21	Gan Cao
MOL000392	Formononetin	69.67	0.21	Gan Cao
MOL000422	Kaempferol	41.88	0.24	Gan Cao
MOL000417	Calycosin	47.75	0.24	Gan Cao
MOL004991	7-Acetoxy-2-methylisoflavone	38.92	0.26	Gan Cao
MOL004990	7,2’,4’-Trihydroxy–5-methoxy-3–arylcoumarin	83.71	0.27	Gan Cao
MOL004860	Licorice glycoside E	32.89	0.27	Gan Cao
MOL000098	Quercetin	46.43	0.28	Gan Cao
MOL000497	Licochalcone a	40.79	0.29	Gan Cao
MOL000239	Jaranol	50.83	0.29	Gan Cao
MOL005016	Odoratin	49.95	0.3	Gan Cao
MOL000354	Isorhamnetin	49.6	0.31	Gan Cao
MOL004898	(E)-3-[3,4-dihydroxy-5-(3-methylbut-2-enyl)phenyl]-1-(2,4-dihydroxyphenyl)prop-2-en-1-one	46.27	0.31	Gan Cao
MOL004910	Glabranin	52.9	0.31	Gan Cao
MOL004945	(2S)-7-hydroxy-2-(4-hydroxyphenyl)-8-(3-methylbut-2-enyl)chroman-4-one	36.57	0.32	Gan Cao
MOL004848	Licochalcone G	49.25	0.32	Gan Cao
MOL004980	Inflacoumarin A	39.71	0.33	Gan Cao
MOL004961	Quercetin der.	46.45	0.33	Gan Cao
MOL002565	Medicarpin	49.22	0.34	Gan Cao
MOL004829	Glepidotin B	64.46	0.34	Gan Cao
MOL004828	Glepidotin A	44.72	0.35	Gan Cao
MOL004815	(E)-1-(2,4-dihydroxyphenyl)-3-(2,2-dimethylchromen-6-yl)prop-2-en-1-one	39.62	0.35	Gan Cao
MOL004907	Glyzaglabrin	61.07	0.35	Gan Cao
MOL004882	Licocoumarone	33.21	0.36	Gan Cao
MOL003656	Lupiwighteone	51.64	0.37	Gan Cao
MOL005020	Dehydroglyasperins C	53.82	0.37	Gan Cao
MOL004915	Eurycarpin A	43.28	0.37	Gan Cao
MOL004838	8-(6-Hydroxy-2-benzofuranyl)-2,2-dimethyl-5-chromenol	58.44	0.38	Gan Cao
MOL005000	Gancaonin G	60.44	0.39	Gan Cao
MOL004811	Glyasperin C	45.56	0.4	Gan Cao
MOL004856	Gancaonin A	51.08	0.4	Gan Cao
MOL004993	8-Prenylated eriodictyol	53.79	0.4	Gan Cao
MOL004864	5,7-Dihydroxy-3-(4-methoxyphenyl)-8-(3-methylbut-2-enyl)chromone	30.49	0.41	Gan Cao
MOL004989	6-Prenylated eriodictyol	39.22	0.41	Gan Cao
MOL004863	3-(3,4-Dihydroxyphenyl)-5,7-dihydroxy-8-(3-methylbut-2-enyl)chromone	66.37	0.41	Gan Cao
MOL004935	Sigmoidin-B	34.88	0.41	Gan Cao
MOL004866	2-(3,4-Dihydroxyphenyl)-5,7-dihydroxy-6-(3-methylbut-2-enyl)chromone	44.15	0.41	Gan Cao
MOL004883	Licoisoflavone	41.61	0.42	Gan Cao
MOL004949	Isolicoflavonol	45.17	0.42	Gan Cao
MOL004814	Isotrifoliol	31.94	0.42	Gan Cao
MOL004913	1,3-Dihydroxy-9-methoxy-6-benzofurano[3,2-c]chromenone	48.14	0.43	Gan Cao
MOL004849	3-(2,4-Dihydroxyphenyl)-8-(1,1-dimethylprop-2-enyl)-7-hydroxy-5-methoxy-coumarin	59.62	0.43	Gan Cao
MOL004808	Glyasperin B	65.22	0.44	Gan Cao
MOL004911	Glabrene	46.27	0.44	Gan Cao
MOL004833	Phaseolinisoflavan	32.01	0.45	Gan Cao
MOL004857	Gancaonin B	48.79	0.45	Gan Cao
MOL004908	Glabridin	53.25	0.47	Gan Cao
MOL004855	Licoricone	63.58	0.47	Gan Cao
MOL004879	Glycyrin	52.61	0.47	Gan Cao
MOL005012	Licoagroisoflavone	57.28	0.49	Gan Cao
MOL004912	Glabrone	52.51	0.5	Gan Cao
MOL004820	kanzonols W	50.48	0.52	Gan Cao
MOL004978	2-[(3r)-8,8-dimethyl-3,4-dihydro-2h-pyrano[6,5-f]chromen-3-yl]-5-methoxyphenol	36.21	0.52	Gan Cao
MOL004914	1,3-Dihydroxy-8,9-dimethoxy-6-benzofurano[3,2-c]chromenone	62.9	0.53	Gan Cao
MOL004810	Glyasperin F	75.84	0.54	Gan Cao
MOL001484	Inermine	75.18	0.54	Gan Cao
MOL004885	Licoisoflavanone	52.47	0.54	Gan Cao
MOL004884	Licoisoflavone B	38.93	0.55	Gan Cao
MOL004905	3,22-Dihydroxy-11-oxo-delta(12)-oleanene-27-alpha-methoxycarbonyl-29-oic acid	34.32	0.55	Gan Cao
MOL004827	Semilicoisoflavone B	48.78	0.55	Gan Cao
MOL004806	Euchrenone	30.29	0.57	Gan Cao
MOL004974	3’-Methoxyglabridin	46.16	0.57	Gan Cao
MOL004966	3’-Hydroxy-4’-o-Methylglabridin	43.71	0.57	Gan Cao
MOL005017	Phaseol	78.77	0.58	Gan Cao
MOL005003	Licoagrocarpin	58.81	0.58	Gan Cao
MOL005007	Glyasperins M	72.67	0.59	Gan Cao
MOL005008	Glycyrrhiza flavonol A	41.28	0.6	Gan Cao
MOL004824	(2S)-6-(2,4-dihydroxyphenyl)-2-(2-hydroxypropan-2-yl)-4-methoxy-2,3-dihydrofuro[3,2-g]chromen-7-one	60.25	0.63	Gan Cao
MOL004959	1-Methoxyphaseollidin	69.98	0.64	Gan Cao
MOL004904	Licopyranocoumarin	80.36	0.65	Gan Cao
MOL002311	Glycyrol	90.78	0.67	Gan Cao
MOL005013	18α-Hydroxyglycyrrhetic acid	41.16	0.71	Gan Cao
MOL004805	(2S)-2-[4-hydroxy-3-(3-methylbut-2-enyl)phenyl]-8,8-dimethyl-2,3-dihydropyrano[2,3-f]chromen-4-one	31.79	0.72	Gan Cao
MOL004891	Shinpterocarpin	80.3	0.73	Gan Cao
MOL004903	Liquiritin	65.69	0.74	Gan Cao
MOL000359	Sitosterol	36.91	0.75	Gan Cao
MOL000211	Mairin	55.38	0.78	Gan Cao
MOL005001	Gancaonin H	50.1	0.78	Gan Cao
MOL004917	Glycyroside	37.25	0.79	Gan Cao
MOL004948	Isoglycyrol	44.7	0.84	Gan Cao
MOL005018	Xambioona	54.85	0.87	Gan Cao
MOL004988	Kanzonol F	32.47	0.89	Gan Cao
MOL004924	(−)-Medicocarpin	40.99	0.95	Gan Cao

DFV = , DL = drug-likeness, HMO = , OB = oral bioavailability.

### 3.2. Acquisition of intersecting genes

**Figure 2.**
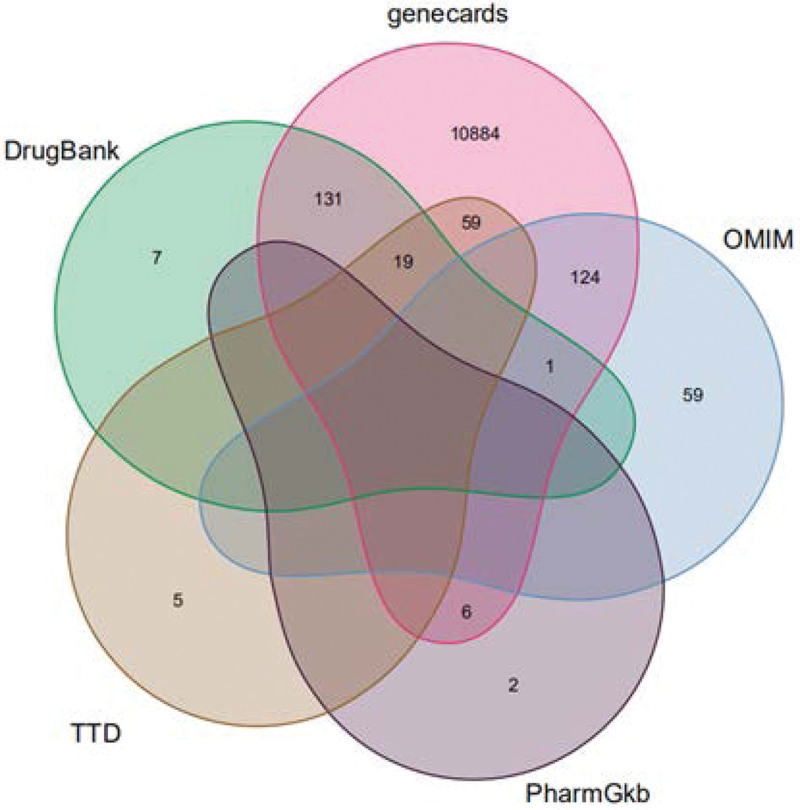
Disease-associated genes map.

**Figure 3.**
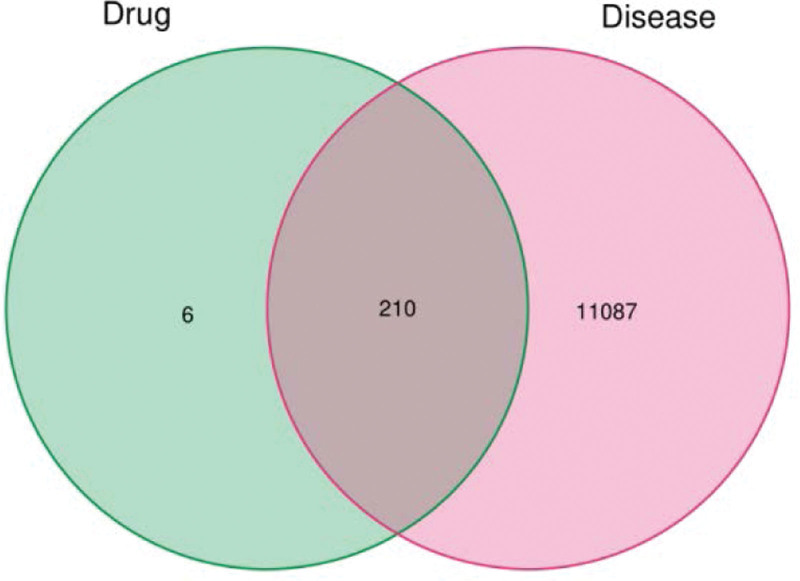
Intersectional gene maps.

As shown in Figure 2, a total of 11,277 potential targets for T2DM were obtained from the GeneCards database, OMIM database, Pharmacogenomics Knowledge Base database, Therapeutic Target Database database DrugBank database, and the retrieved disease-related genes were combined to draw a Wayne map. In Figure 3, the green and pink circles represent the predicted targets of the compound and T2DM. The intersecting part in the middle indicates the targets shared by both, suggesting that the 210 intersecting targets may be the potential targets of LGZG decoction for the treatment of T2DM.

### 3.3. Drug-active ingredient-target gene network construction

**Figure 4.**
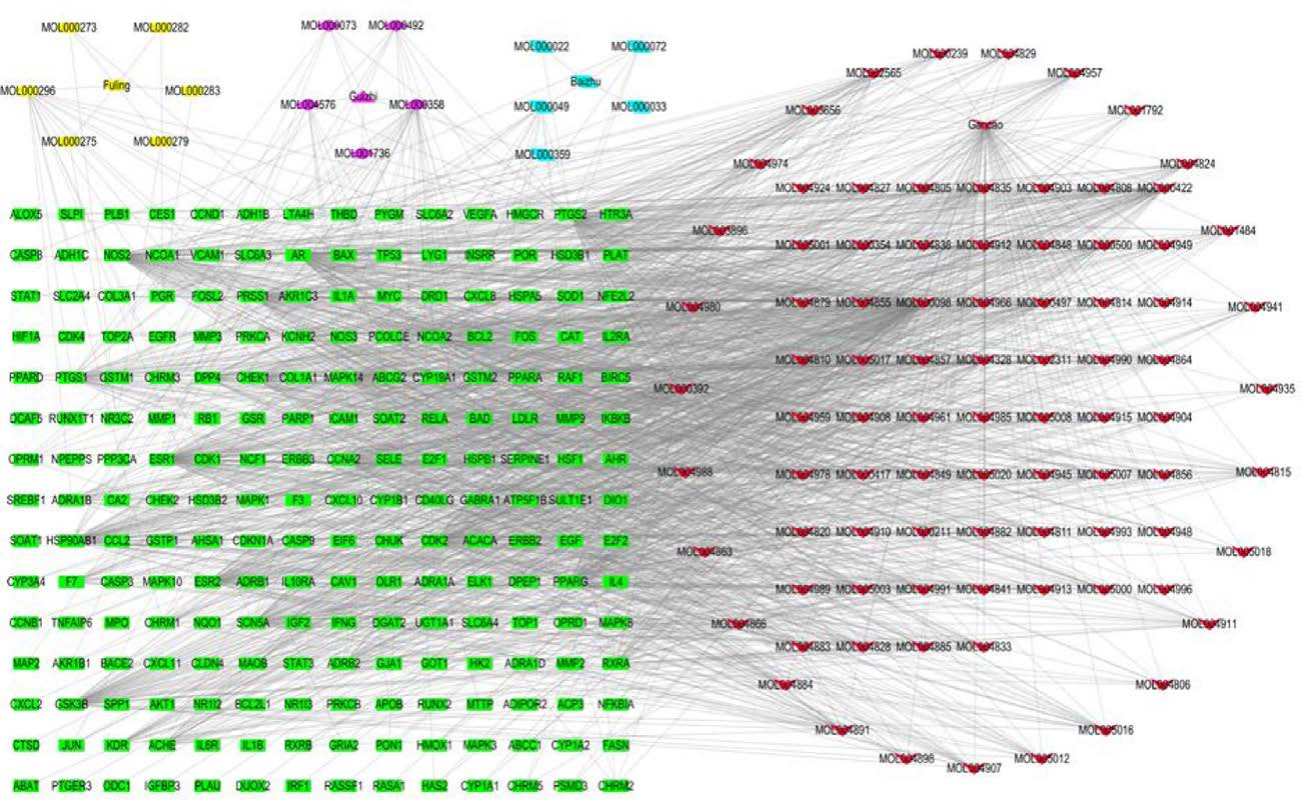
Drug-active ingredient-target network maps. Different shapes and colors represent different information. The red inverted triangle represents Gancao, the golden hexagon represents Fuling, the pink quadrilateral represents Guizhi, the blue diamond represents atractylodes, and the green rectangle represents a gene.

Figure 4 shows a “drug-active ingredient-target gene” network map with 317 nodes (4 drugs,103 active compounds, and 210 targets) and 1608 edges constructed in an Excel sheet and imported into Cytoscape 3.8.0 software. The red inverted triangle represents Gancao, the golden hexagon represents Fuling, the pink quadrilateral represents Guizhi, the blue diamond represents atractylodes, green rectangle represents a gene. The top active ingredients can be filtered out from the figure, with quercetin (quercetin) targeting up to 137 associations, kaempferol (kaempferol) with 53 associations, naringenin (naringenin) with 34 associations, and 7-methoxy-2-methyl isoflavone (isoflavone) with 34 associations (as shown in Supplemental File S2, Supplemental Digital Content, http://links.lww.com/MD/I645, which illustrates the basic information of network construction and the types of nodes).

### 3.4. Construction and analysis of PPI

**Figure 5.**
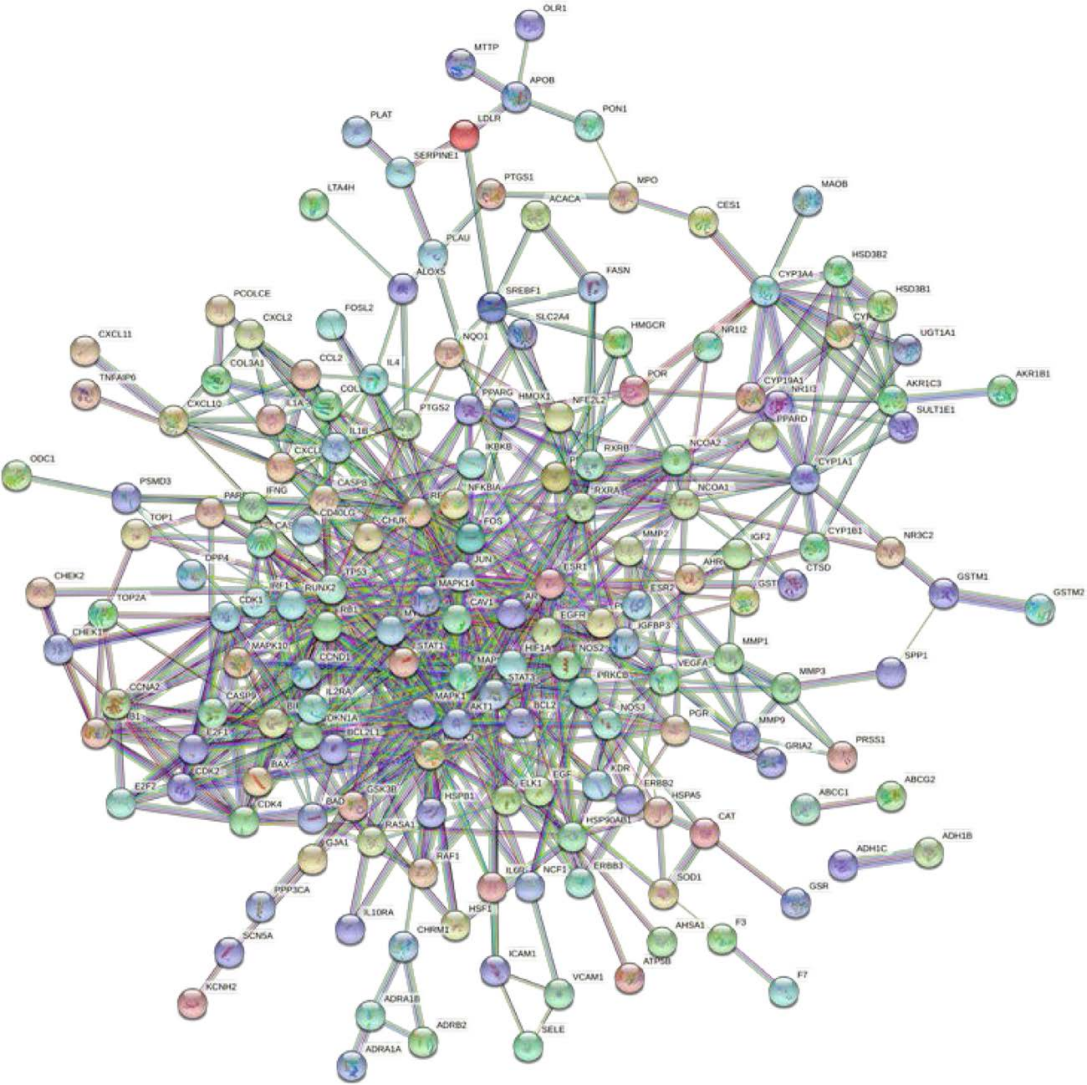
Protein interaction network (PPI) maps.

**Figure 6.**
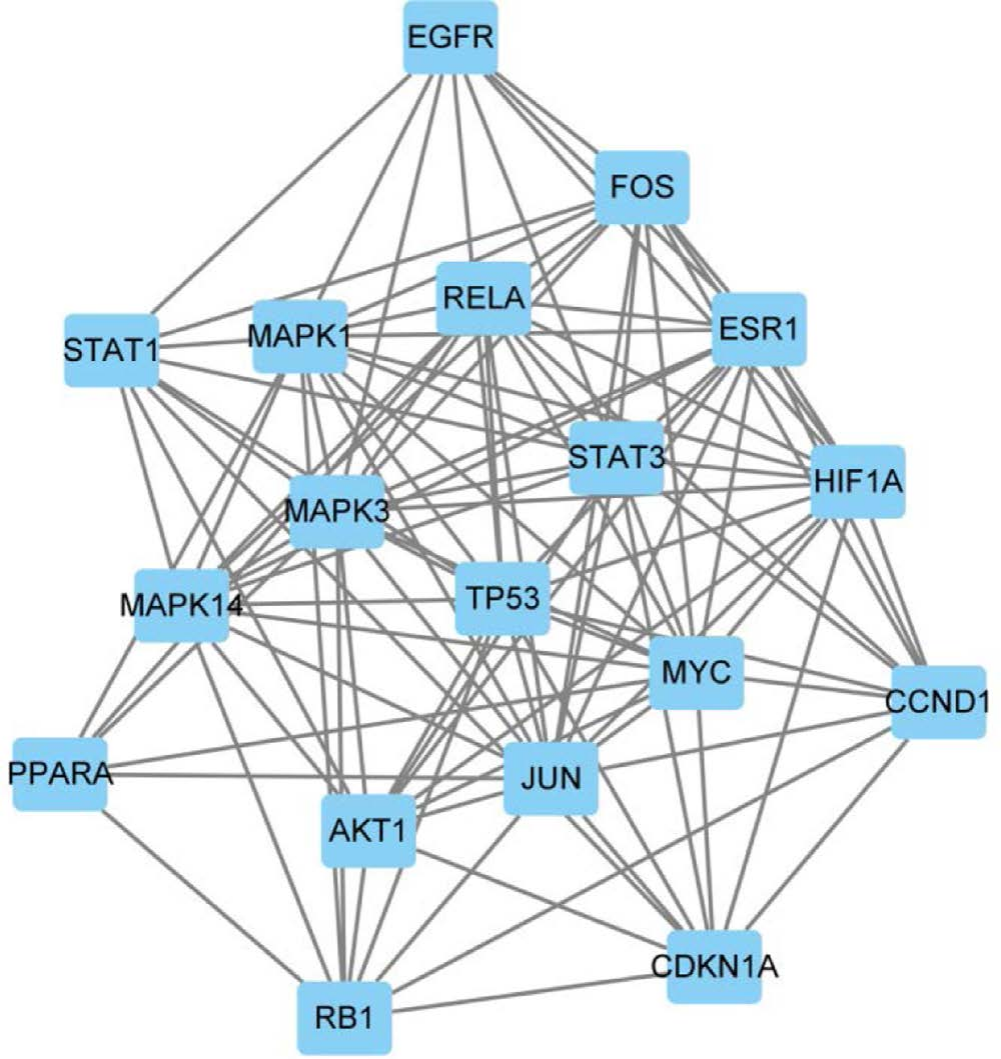
Network core genetic map.

The above 210 intersecting genes were uploaded to the STRING database to obtain the PPI map, as shown in Figure 5. The PPI network map was imported into Cytosccape 3.8.0 software, and each node was scored using the CytoNCA plug-in. As shown in Figure 6, the nodes with scores less than the median were filtered according to the scores, and the nodes with higher scores were retained to obtain the network core genes.

### 3.5. GO and KEGG enrichment analysis

**Figure 7.**
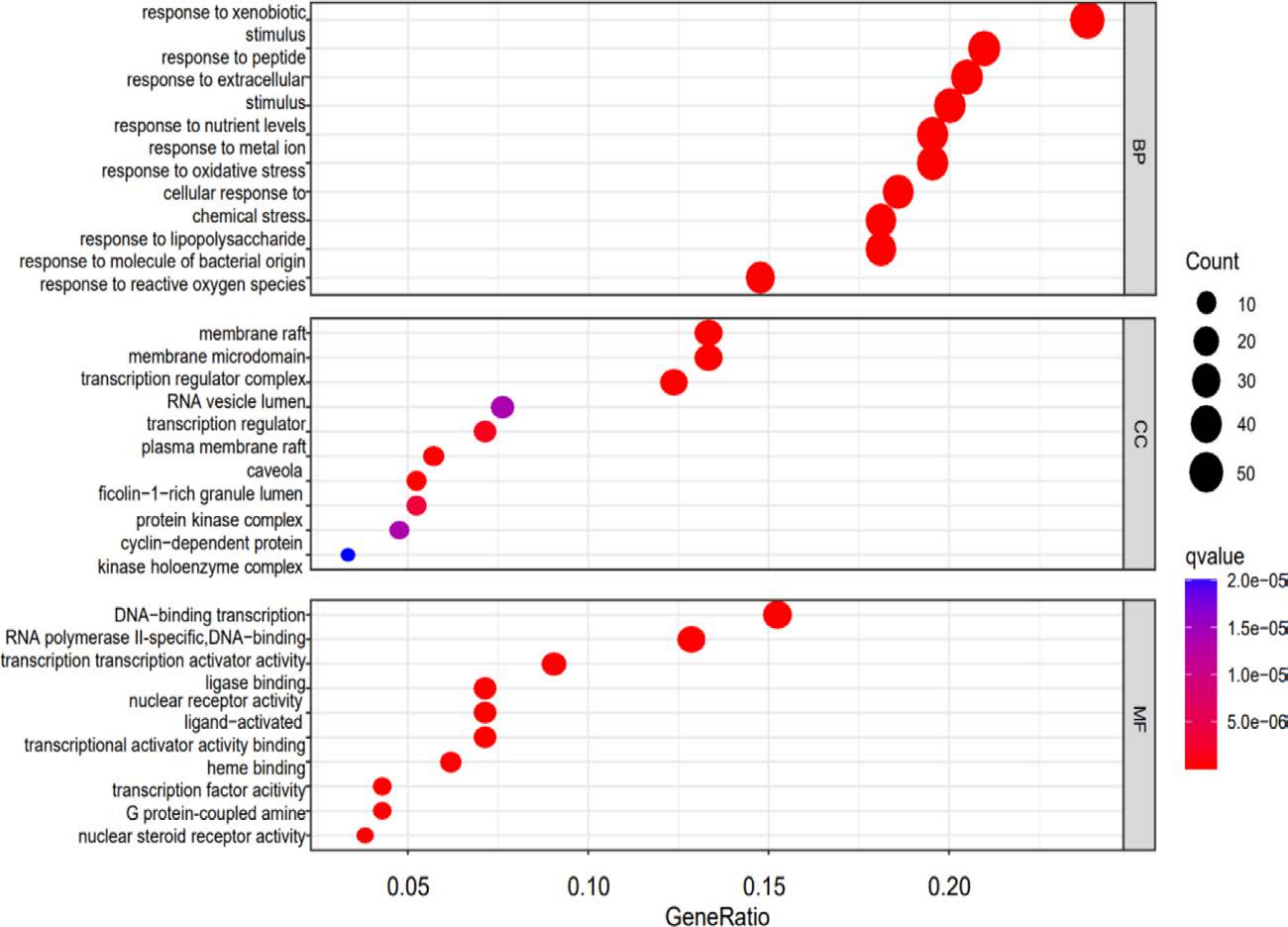
GO function enrichment analysis. The top 10 entries are retained separately according to *P* < .05, where larger and redder bubbles indicate a higher number of enriched targets. GO = Gene Ontology, BP = biological process, CC = cellular component, MF = molecular function.

The GO analysis was performed in R language for the intersection of LGZG decoction and T2DM (Fig. 7), and the *P* value was set at 0.05. The GO annotation analysis was performed in 3 aspects: biological process (BP), cellular component, and molecular function (MF). The results of GO annotation analysis showed that the BP mainly involved the response to xenobiotic stimuli, the response to metal ions, the cellular response to chemical stress, the response to lipopolysaccharide, peptide, and extracellular matrix, and the oxidative stress response. Composition mainly involves membrane rafts, membrane microstructure domains, transcriptional regulatory factors, transcriptional regulatory complexes, protein kinase complexes, vesicle lumen, etc. MFs mainly involve DNA-binding transcription factors, cytosolic receptor activity, activated ligands, ligase conjugates, subtilisin conjugates, G protein-coupled amine receptor activity, cytosolic steroid receptor activity, etc.

**Figure 8.**
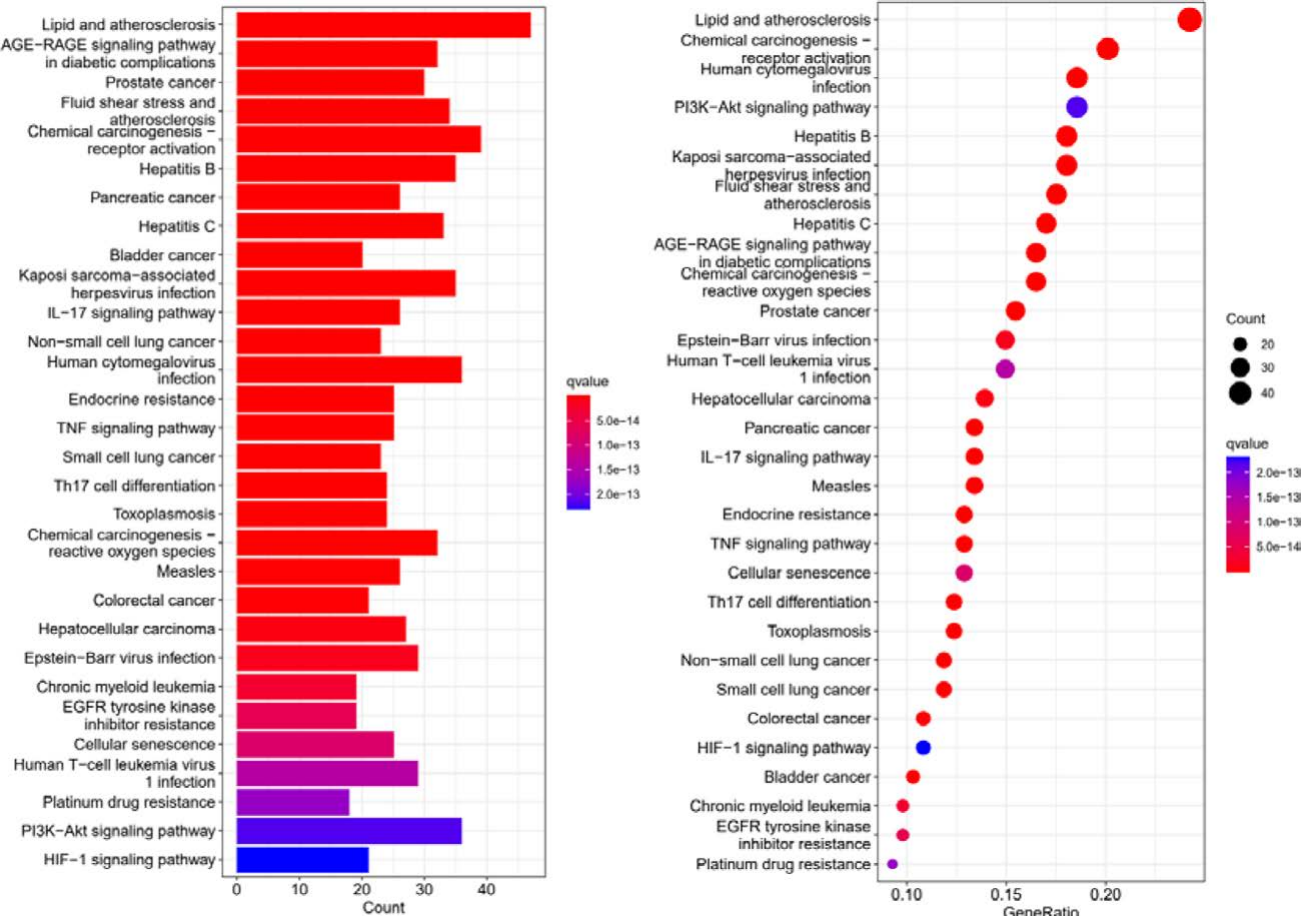
KEGG pathway enrichment analysis. The y-axis demonstrates the top 18 significantly enriched KEGG pathways, while the x-axis shows the number of enriched genes for these terms (*P* < .05). The colors and the sizes indicate different *P* value ranges; the redder and bigger it is, the more significantly enriched it is. KEGG = Kyoto Encyclopedia of Genes and Genomes.

The R software was used to analyze the pathways and visualize them by setting *P* to .05 (KEGG pathway, Fig. 8), and the top 30 signaling pathways were screened. The results indicated that the target genes were mainly involved in these signaling pathways, including lipid and atherosclerosis, advanced glycosylation end products (AGE)-in diabetic complications receptor of AGE (RAGE) signaling pathway, phosphatidylinositol 3 kinase (PI3K)-Akt signaling pathway, interleukin (IL)-17 signaling pathway, HIF-1 signaling pathway, tumor necrosis factor (TNF) signaling pathway, T helper cell 17 cell differentiation, and other pathways (as shown in Supplemental File S3, Supplemental Digital Content, http://links.lww.com/MD/I646, which illustrates GO and KEGG pathway analysis results).

### 3.6. Active ingredient-target molecular docking

**Figure 9.**
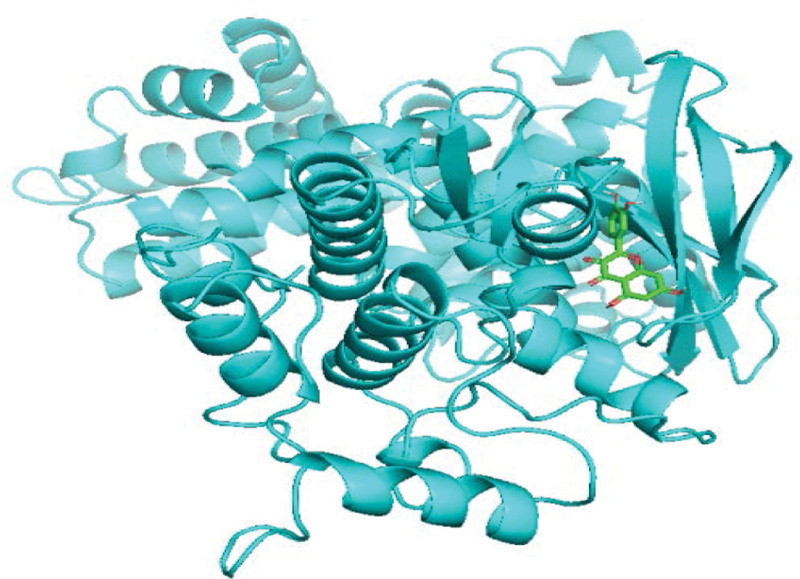
Schematic map of quercetin-CCND1 molecular docking.

**Figure 10.**
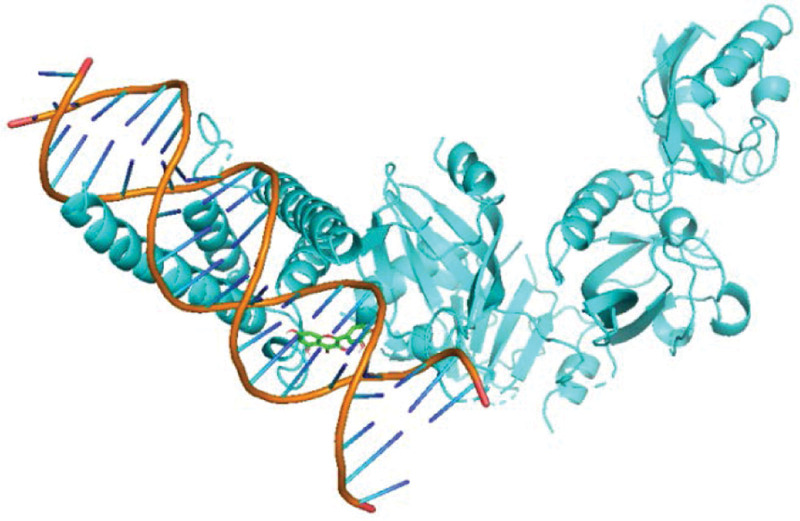
Schematic map of quercetin-HIF1A molecular docking. HIF1A = hypoxia-inducible factor-1.

**Figure 11.**
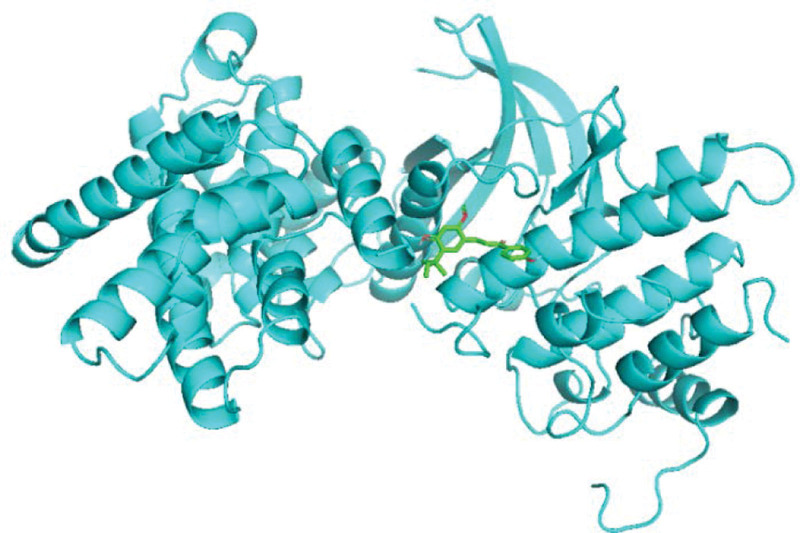
Schematic map of molecular docking of licorice chalcone A-CCND1.

As shown in Table 2, after a comprehensive analysis of their network values, statistical indicators, and count values, the top-ranked quercetin (quercetin), kaempferol (kaempferol), naringenin (naringenin) and licochalcone a (licorice chalcone A) among the obtained active ingredients were molecularly docked with the top-ranked key targets signal transducers and activators of transcription (STAT)-3, hypoxia-inducible factor-1 (HIF1A), STAT1 and AKT serine/threonine kinase 1 (AKT1), respectively. The smaller the binding energy, the greater the affinity and the higher the binding activity. The molecular docking results of licochalcone a with recombinant cyclin D1 (CCND1), quercetin with CCND1, and HIF1A were selected for demonstration according to their binding energy sizes, Figures 9–11.

**Table 2:** Molecular docking results (kJ·mol^−1^).

Core targets	Core ingredients	Combined energy (kJ·mol^−1^)
STAT3	Licochalcone a	−6.9
HIF1A	Quercetin	−8.4
STAT1	Quercetin	−7.3
	Kaempferol	−7
AKT1	Naringenin	−6.1
	Quercetin	−6.1
	Kaempferol	−5.9
CCND1	Licochalcone a	−7.3
	Quercetin	−8.8

AKT1 = AKT serine/threonine kinase 1, CCND1 = recombinant cyclin D1, HIF1A = hypoxia-inducible factor-1, STAT = signal transducers and activators of transcription.

## 4. Discussion

Our findings indicate that the top-ranked active ingredients in LGZG decoction were quercetin (quercetin), kaempferol (kaempferol), naringenin (naringenin), and licochalcone a (licorice chalcone A). It has been shown that quercetin inhibits NOX expression and ROS production in rat Achilles tendons under hyperglycemic conditions, has antioxidant and anti-inflammatory effects, and can prevent the development of diabetic tendinopathy.^[13]^ Its derivatives have a protective effect on diabetic neuropathy by inhibiting the Wnt/β-catenin signaling pathway.^[14]^ Quercetin intake was negatively correlated with the prevalence of T2DM,^[15]^ and its normalization of pancreatic β-cells was achieved by reducing iron death in T2DM by eliminating oxidative stress.^[16]^ Kaempferol modulates M1/M2 phenotype under hyperglycemia and indirectly protects against podocyte apoptosis by regulating macrophage M1/M2 differentiation^[17]^; in terms of its hypoglycemic mechanism, it may achieve hypoglycemic efficacy by inhibiting the digestion of carbohydrates.^[18]^ Another scholar found in rat experiments that naringin could prevent hyperglycemia-mediated-induced inflammation and damage to liver and pancreatic tissues by blocking the activation-mediated anti-inflammatory effects of NF-κB and its down-regulated genes, including pro-inflammatory cytokines.^[19]^ In contrast, licochalcone A is capable of modulating insulin sensitivity in modern pharmacology, thus exerting a corresponding hypoglycemic effect.^[20]^

The GO functional enrichment analysis results showed that the treatment of T2DM by LGZG decoction is extremely complex, involving multiple aspects of BPs, cell composition, and MFs. To sum up, the action targets located at the center of the network constructed by Cytoscape include STAT3, HIF1A, STAT1, AKT1, and CCND1.

It has been shown that in studying the effects of T2D risk genes and SNPs on transcriptional binding affinity, the top 4 transcription factors associated with enrichment were found to be Rfx1, Nkx2-5, NR2C2, and MZF15-13, while TF-Rfx1 is in the signal transducer and activator of transcription 3 (STAT3) pathway The expression of Rfx1 is regulated by the IL-6-STAT3 signaling pathway, which also indicates that STAT3 plays an important role in the pathogenesis of diabetes.^[21–23]^ STAT3, under high glucose conditions, after phosphorylation, causes hepatic gluconeogenesis while reducing glycogen synthesis and elevating blood glucose.^[24]^ In experiments in which systemic injection of streptozotocin-induced hyperglycemia in rats, it was found that during streptozotocin-induced diabetic retinopathy, the levels of HIF-1α, as well as the pro-inflammatory cytokines IL-1β, IL-6, and TNF-α, were increased and that HIF-1α led to upregulation of IL-6 and TNF-α and their receptors as well as Caspase-3, and inhibition of HIF-1α decreases the expression of the pro-inflammatory mediators IL-6 and TNF-αin diabetic retinopathy, thereby reducing the incidence of diabetic retinopathy.^[25]^ AKT1 is an important link in the PI3K/AKT/mTOR signaling pathway. Large amounts of AKT1 activate mTOR and enhance SREBP1 efficacy, thereby increasing intracellular triacylglycerol in tissues^[26]^ to achieve energy homeostasis. Akt is essential for insulin and nutrient-mediated regulation of hepatic metabolism in the body.^[27]^ It has also been shown that hepatic CCND1 deficiency leads to increased gluconeogenesis and, consequently, to hyperglycemia.^[28]^

The AGE-RAGE signaling pathway, PI3K-Akt signaling pathway, and HIF-1 signaling pathway in lipids and atherosclerosis, diabetic complications may play an important role in the treatment of T2DM with LGZG decoction as seen in the results of enrichment pathways in KEGG. Studies have shown that a persistent hyperglycemic state will cause plasma protein glycosylation, and insulin glycosylation can distort insulin signaling^[29]^ and reduce insulin sensitivity to adipocyte cell membrane surface receptors,^[30]^ which will cause hyperlipidemia and atherosclerosis manifestations in the long-term^[31]^ and aggravate the risk of T2DM complications. One study showed that the risk of gestational diabetes mellitus increased 18.48-fold for each unit increase in atherosclerotic plasma index, indicating that reasonable lipid control in mid-pregnancy may reduce the incidence of gestational diabetes mellitus, statistically demonstrating an association between lipids and atherosclerosis and diabetes.^[32]^ The PI3K/Akt signaling pathway of key downstream factors is closely related to the regulation of glucose and lipid metabolism,^[33]^ and data suggesting a reduction in the occurrence of diabetic osteoporosis with further upregulation of PI3K/Akt-related protein levels.^[34]^ In turn, activation of the HIF-1 signaling pathway is associated with inflammatory and fibrotic processes in the renal unit and vascular calcification in patients with T2DM.^[35,36]^

In conclusion, the pharmacological process of LGZG decoction for the treatment of T2DM may be a synergistic combination of multiple active ingredients, multiple action targets, and multiple signaling pathways. Various core ingredients in the formula, such as quercetin, kaempferol, naringenin, and licorice chalcone A, may act on STAT3, HIF1A, STAT1, AKT1, CCND1, and other potential key targets and then exert synergistic effects on multiple signaling pathways such as AGE-RAGE signaling pathway, PI3K-Akt signaling pathway and HIF-1 signaling pathway in lipid and atherosclerosis, diabetic complications. In turn, it exerts a holistic and complex regulatory effect on multiple signaling pathways, including the AGE-RAGE signaling pathway, PI3K-Akt signaling pathway, and HIF-1 signaling pathway in lipid and atherosclerosis diabetes complications. According to TCM, T2DM belongs to the category of “thirst” in TCM and is often due to spleen deficiency and water retention. The combination of the 4 herbs in LGZG decoction brings out the effect of “warming and harmonizing” to achieve the function of warming yang, transforming qi, promoting water, and dispelling dampness. This paper predicts and analyzes the possible pharmacological mechanism of LGZG decoction in the treatment of T2DM by using software technology related to network pharmacology. At the same time, the results of the current experimental research are used to prove that the results are still scientific and reasonable. However, from the perspective of scientific rigor, further experimental research data are needed to support the results, so it provides a theoretical basis and direction to explore the pharmacological process of LGZG decoction in the treatment of T2DM. However, from the point of view of scientific rigor, in the study, although we have selected as much data as possible from the database, some important targets may still be missed because the database needs to be updated in time or the research needs to be more comprehensive. Moreover, the study that some of the selected signaling pathways are indeed part of the pathogenesis of diabetes and its complications is not enough. There are differences between in vitro theoretical study and in vivo metabolism. In addition, the study aims to construct a possible active ingredient-target network through relevant software technology. Due to the limitations of the research technology, we need further experimental research data to prop up.

## Author contributions

**Conceptualization:** Feng Long, Zhe Zhang.

**Data curation:** Feng Long, Zhe Zhang.

**Methodology:** Feng Long, Zhe Zhang.

**Resources:** Feng Long, Chunxiu Luo, Jinlian Guo, Lin An.

**Software:** Feng Long, Zhe Zhang.

**Supervision:** Xiao Lei.

**Writing – original draft:** Feng Long.

**Writing – review & editing:** Zhe Zhang, Xiao Lei.

## Supplementary Material






