# Supplementary material for: Exploring the molecular mechanism of Ling-Gui-Zhu-Gan decoction for the treatment of type 2 diabetes mellitus based on network pharmacology and molecular docking: A review
Source: Medicine (Baltimore). 2023 Mar 24;102(12):e33210. doi: 10.1097/MD.0000000000033210 (PMC10036033; doi:10.1097/MD.0000000000033210)
Supplement: Supplementary file 1 [file medi-102-e33210-s001.pdf]

Supplementary file 1-Basic information on all active compounds and targets

Exploring the mechanism of Ling-Gui-Zhu-Gan-decoction for the treatment of type 2 diabetes based on network pharmacology and molecular docking

Feng Long, MS[1],Zhe Zhang, MS[1],ChunXiu Luo, MS[1],Xiao Lei, PHD[1]*，Jinlian Guo, MS[1],Lin An, MS[1]

1.Department of traditional Chinese Medicine, affiliated Hospital of North Sichuan Medical College, Nanchong, Sichuan 637000.

* **Correspondence**: Xiao Lei，2424932797@qq.com

Table S1: The basic information of all active compounds				
herbal name	Molecular name	Mol ID	OB (%)	DL
Baizhu	12-senecioyl-2E,8E,10E-atractylentriol	MOL000020	62.4	0.22
Baizhu	14-acetyl-12-senecioyl-2E,8E,10E-atractylentriol	MOL000021	60.31	0.31
Baizhu	14-acetyl-12-senecioyl-2E,8Z,10E-atractylentriol	MOL000022	63.37	0.3
Baizhu	α-Amyrin	MOL000028	39.51	0.76
Baizhu	(3S,8S,9S,10R,13R,14S,17R)-10,13-dimethyl-17-[(2R,5S)-5-propan-2-yloctan-2-yl]-2,3,4,7,8,9,11,12,14,15,16,17-dodec	MOL000033	36.23	0.78

	ahydro-1H-cyclopenta[a]phen anthren-3-ol			
Baizhu	3β-acetoxyatractylone	MOL000049	54.07	0.22
Baizhu	8β-ethoxy atractylenolide Ⅲ	MOL000072	35.95	0.21
Fuling	(2R)-2-[(3S,5R,10S,13R,14R, 16R,17R)-3,16-dihydroxy-4,4, 10,13,14-pentamethyl-2,3,5,6, 12,15,16,17-octahydro-1H-cyc lopenta[a]phenanthren-17-yl]- 6-methylhept-5-enoic acid	MOL000273	30.93	0.81
Fuling	trametenolic acid	MOL000275	38.71	0.8
Fuling	7,9(11)-dehydropachymic acid	MOL000276	35.11	0.81
Fuling	Cerevisterol	MOL000279	37.96	0.77
Fuling	(2R)-2-[(3S,5R,10S,13R,14R, 16R,17R)-3,16-dihydroxy-4,4, 10,13,14-pentamethyl-2,3,5,6, 12,15,16,17-octahydro-1H-cyc lopenta[a]phenanthren-17-yl]- 5-isopropyl-hex-5-enoic acid	MOL000280	31.07	0.82
Fuling	ergosta-7,22E-dien-3beta-ol	MOL000282	43.51	0.72
Fuling	Ergosterol peroxide	MOL000283	40.36	0.81
Fuling	(2R)-2-[(5R,10S,13R,14R,16R ,17R)-16-hydroxy-3-keto-4,4, 10,13,14-pentamethyl-1,2,5,6, 12,15,16,17-octahydrocyclope nta[a]phenanthren-17-yl]-5-iso propyl-hex-5-enoic acid	MOL000285	38.26	0.82
Fuling	3beta-Hydroxy-24-methylene- 8-lanostene-21-oic acid	MOL000287	38.7	0.81
Fuling	pachymic acid	MOL000289	33.63	0.81
Fuling	Poricoic acid A	MOL000290	30.61	0.76
Fuling	Poricoic acid B	MOL000291	30.52	0.75
Fuling	poricoic acid C	MOL000292	38.15	0.75
Fuling	hederagenin	MOL000296	36.91	0.75
Fuling	dehydroeburicoic acid	MOL000300	44.17	0.83
Gancao	(2R)-7-hydroxy-2-(4-hydroxy phenyl)chroman-4-one	MOL004941	71.12	0.18
Gancao	DFV	MOL001792	32.76	0.18
Gancao	Glypallichalcone	MOL004835	61.6	0.19
Gancao	Licochalcone B	MOL004841	76.76	0.19
Gancao	icos-5-enoic acid	MOL004985	30.7	0.2
Gancao	gadelaidic acid	MOL004996	30.7	0.2
Gancao	7-Methoxy-2-methyl	MOL003896	42.56	0.2

	isoflavone			
Gancao	Vestitol	MOL000500	74.66	0.21
Gancao	HMO	MOL004957	38.37	0.21
Gancao	naringenin	MOL004328	59.29	0.21
Gancao	formononetin	MOL000392	69.67	0.21
Gancao	kaempferol	MOL000422	41.88	0.24
Gancao	Calycosin	MOL000417	47.75	0.24
Gancao	7-Acetoxy-2-methylisoflavone	MOL004991	38.92	0.26
Gancao	7,2',4'-trihydroxy－5-methoxy-3－arylcoumarin	MOL004990	83.71	0.27
Gancao	licorice glycoside E	MOL004860	32.89	0.27
Gancao	quercetin	MOL000098	46.43	0.28
Gancao	licochalcone a	MOL000497	40.79	0.29
Gancao	Jaranol	MOL000239	50.83	0.29
Gancao	Odoratin	MOL005016	49.95	0.3
Gancao	isorhamnetin	MOL000354	49.6	0.31
Gancao	(E)-3-[3,4-dihydroxy-5-(3-methylbut-2-enyl)phenyl]-1-(2,4-dihydroxyphenyl)prop-2-en-1-one	MOL004898	46.27	0.31
Gancao	Glabranin	MOL004910	52.9	0.31
Gancao	(2S)-7-hydroxy-2-(4-hydroxyphenyl)-8-(3-methylbut-2-enyl)chroman-4-one	MOL004945	36.57	0.32
Gancao	licochalcone G	MOL004848	49.25	0.32
Gancao	Inflacoumarin A	MOL004980	39.71	0.33
Gancao	Quercetin der.	MOL004961	46.45	0.33
Gancao	Medicarpin	MOL002565	49.22	0.34
Gancao	Glepidotin B	MOL004829	64.46	0.34
Gancao	Glepidotin A	MOL004828	44.72	0.35
Gancao	(E)-1-(2,4-dihydroxyphenyl)-3-(2,2-dimethylchromen-6-yl)prop-2-en-1-one	MOL004815	39.62	0.35
Gancao	Glyzaglabrin	MOL004907	61.07	0.35
Gancao	Licocoumarone	MOL004882	33.21	0.36
Gancao	Lupiwighteone	MOL003656	51.64	0.37
Gancao	dehydroglyasperins C	MOL005020	53.82	0.37
Gancao	Eurycarpin A	MOL004915	43.28	0.37
Gancao	8-(6-hydroxy-2-benzofuranyl)-2,2-dimethyl-5-chromenol	MOL004838	58.44	0.38
Gancao	Gancaonin G	MOL005000	60.44	0.39
Gancao	Glyasperin C	MOL004811	45.56	0.4
Gancao	Gancaonin A	MOL004856	51.08	0.4

Gancao	8-prenylated eriodictyol	MOL004993	53.79	0.4
Gancao	5,7-dihydroxy-3-(4-methoxyphenyl)-8-(3-methylbut-2-enyl)chromone	MOL004864	30.49	0.41
Gancao	6-prenylated eriodictyol	MOL004989	39.22	0.41
Gancao	3-(3,4-dihydroxyphenyl)-5,7-dihydroxy-8-(3-methylbut-2-enyl)chromone	MOL004863	66.37	0.41
Gancao	Sigmoidin-B	MOL004935	34.88	0.41
Gancao	2-(3,4-dihydroxyphenyl)-5,7-dihydroxy-6-(3-methylbut-2-enyl)chromone	MOL004866	44.15	0.41
Gancao	Licoisoflavone	MOL004883	41.61	0.42
Gancao	Isolicoflavonol	MOL004949	45.17	0.42
Gancao	Isotrifoliol	MOL004814	31.94	0.42
Gancao	1,3-dihydroxy-9-methoxy-6-benzofurano[3,2-c]chromenone	MOL004913	48.14	0.43
Gancao	3-(2,4-dihydroxyphenyl)-8-(1,1-dimethylprop-2-enyl)-7-hydroxy-5-methoxy-coumarin	MOL004849	59.62	0.43
Gancao	glyasperin B	MOL004808	65.22	0.44
Gancao	Glabrene	MOL004911	46.27	0.44
Gancao	Phaseolinisoflavan	MOL004833	32.01	0.45
Gancao	Gancaonin B	MOL004857	48.79	0.45
Gancao	Glabridin	MOL004908	53.25	0.47
Gancao	Licoricone	MOL004855	63.58	0.47
Gancao	Glycyrin	MOL004879	52.61	0.47
Gancao	Licoagroisoflavone	MOL005012	57.28	0.49
Gancao	Glabrone	MOL004912	52.51	0.5
Gancao	kanzonols W	MOL004820	50.48	0.52
Gancao	2-[(3R)-8,8-dimethyl-3,4-dihydro-2H-pyrano[6,5-f]chromen-3-yl]-5-methoxyphenol	MOL004978	36.21	0.52
Gancao	1,3-dihydroxy-8,9-dimethoxy-6-benzofurano[3,2-c]chromenone	MOL004914	62.9	0.53
Gancao	glyasperin F	MOL004810	75.84	0.54
Gancao	Inermine	MOL001484	75.18	0.54
Gancao	licoisoflavanone	MOL004885	52.47	0.54
Gancao	Licoisoflavone B	MOL004884	38.93	0.55
Gancao	3,22-Dihydroxy-11-oxo-delta(12)-oleanene-27-alpha-methoxycarbonyl-29-oic acid	MOL004905	34.32	0.55

Gancao	Semilicoisoflavone B	MOL004827	48.78	0.55
Gancao	euchrenone	MOL004806	30.29	0.57
Gancao	3'-Methoxyglabridin	MOL004974	46.16	0.57
Gancao	3'-Hydroxy-4'-O-Methylglabridin	MOL004966	43.71	0.57
Gancao	Phaseol	MOL005017	78.77	0.58
Gancao	Licoagrocarpin	MOL005003	58.81	0.58
Gancao	Glyasperins M	MOL005007	72.67	0.59
Gancao	Glycyrrhiza flavonol A	MOL005008	41.28	0.6
Gancao	(2S)-6-(2,4-dihydroxyphenyl)-2-(2-hydroxypropan-2-yl)-4-methoxy-2,3-dihydrofuro[3,2-g]chromen-7-one	MOL004824	60.25	0.63
Gancao	1-Methoxyphaseollidin	MOL004959	69.98	0.64
Gancao	licopyranocoumarin	MOL004904	80.36	0.65
Gancao	Glycyrol	MOL002311	90.78	0.67
Gancao	18α-hydroxyglycyrrhetic acid	MOL005013	41.16	0.71
Gancao	(2S)-2-[4-hydroxy-3-(3-methylbut-2-enyl)phenyl]-8,8-dimethyl-2,3-dihydropyrano[2,3-f]chromen-4-one	MOL004805	31.79	0.72
Gancao	shinpterocarpin	MOL004891	80.3	0.73
Gancao	liquiritin	MOL004903	65.69	0.74
Gancao	sitosterol	MOL000359	36.91	0.75
Gancao	Mairin	MOL000211	55.38	0.78
Gancao	Gancaonin H	MOL005001	50.1	0.78
Gancao	glycyroside	MOL004917	37.25	0.79
Gancao	Isoglycyrol	MOL004948	44.7	0.84
Gancao	Xambioona	MOL005018	54.85	0.87
Gancao	Kanzonol F	MOL004988	32.47	0.89
Gancao	(-)-Medicocarpin	MOL004924	40.99	0.95

Table S2. all active compounds and related targets

Drug	MolId	Molecular name	Symbol
Baizhu	MOL000022	14-acetyl-12-senecioyl-2E,8Z,10E-atractylentriol	PTGS2
Baizhu	MOL0000	(3S,8S,9S,10R,13R,14S,17R)-10,13-dimethyl-1	PGR

	33	7-[(2R,5S)-5-propan-2-yloctan-2-yl]-2,3,4,7,8,9,11,12,14,15,16,17-dodecahydro-1H-cyclopenta[a]phenanthren-3-ol	
Baizhu	MOL000049	3β-acetoxyatractylone	CHRM3
Baizhu	MOL000049	3β-acetoxyatractylone	CHRM1
Baizhu	MOL000049	3β-acetoxyatractylone	AR
Baizhu	MOL000049	3β-acetoxyatractylone	SCN5A
Baizhu	MOL000049	3β-acetoxyatractylone	PTGS2
Baizhu	MOL000049	3β-acetoxyatractylone	RXRA
Baizhu	MOL000049	3β-acetoxyatractylone	ACHE
Baizhu	MOL000049	3β-acetoxyatractylone	ADRA1A
Baizhu	MOL000049	3β-acetoxyatractylone	CHRM2
Baizhu	MOL000049	3β-acetoxyatractylone	ADRB2
Baizhu	MOL000049	3β-acetoxyatractylone	OPRM1
Baizhu	MOL000049	3β-acetoxyatractylone	GABRA1
Baizhu	MOL000049	3β-acetoxyatractylone	DPP4
Baizhu	MOL000072	8β-ethoxy atractylenolide Ⅲ	PTGS2
Baizhu	MOL000072	8β-ethoxy atractylenolide Ⅲ	GABRA1
Baizhu	MOL000072	8β-ethoxy atractylenolide Ⅲ	NCOA2
Baizhu	MOL000072	8β-ethoxy atractylenolide Ⅲ	NCOA1
Fuling	MOL000273	(2R)-2-[(3S,5R,10S,13R,14R,16R,17R)-3,16-dihydroxy-4,4,10,13,14-pentamethyl-2,3,5,6,12,15,16,17-octahydro-1H-cyclopenta[a]phenanthren-17-yl]-6-methylhept-5-enoic acid	NR3C2
Fuling	MOL000273	(2R)-2-[(3S,5R,10S,13R,14R,16R,17R)-3,16-dihydroxy-4,4,10,13,14-pentamethyl-2,3,5,6,12,15,16,17-octahydro-1H-cyclopenta[a]phenanthren-	NCOA2

		17-yl]-6-methylhept-5-enoic acid	
Fuling	MOL000275	trametenolic acid	NR3C2
Fuling	MOL000279	Cerevisterol	NR3C2
Fuling	MOL000282	ergosta-7,22E-dien-3beta-ol	PGR
Fuling	MOL000283	Ergosterol peroxide	PGR
Fuling	MOL000296	hederagenin	PGR
Fuling	MOL000296	hederagenin	NCOA2
Fuling	MOL000296	hederagenin	CHRM3
Fuling	MOL000296	hederagenin	CHRM1
Fuling	MOL000296	hederagenin	CHRM2
Fuling	MOL000296	hederagenin	ADRA1B
Fuling	MOL000296	hederagenin	GABRA1
Fuling	MOL000296	hederagenin	GRIA2
Fuling	MOL000296	hederagenin	IGHG1
Fuling	MOL000296	hederagenin	ADH1B
Fuling	MOL000296	hederagenin	ADH1C
Fuling	MOL000296	hederagenin	LYG1
Fuling	MOL000296	hederagenin	PTGS1
Fuling	MOL000296	hederagenin	SCN5A
Fuling	MOL000296	hederagenin	PTGS2
Fuling	MOL000296	hederagenin	RXRA
Fuling	MOL000296	hederagenin	SLC6A2

Gancao	MOL001484	Inermine	PTGS1
Gancao	MOL001484	Inermine	CHRM3
Gancao	MOL001484	Inermine	SCN5A
Gancao	MOL001484	Inermine	PTGS2
Gancao	MOL001484	Inermine	HTR3A
Gancao	MOL001484	Inermine	RXRA
Gancao	MOL001484	Inermine	ADRA1B
Gancao	MOL001484	Inermine	ADRA1D
Gancao	MOL001484	Inermine	IGHG1
Gancao	MOL001484	Inermine	PRSS1
Gancao	MOL001484	Inermine	CAMKMT
Gancao	MOL001484	Inermine	CHRM1
Gancao	MOL001484	Inermine	ADRB2
Gancao	MOL001484	Inermine	OPRM1
Gancao	MOL001484	Inermine	HSP90AB1
Gancao	MOL001792	DFV	PTGS1
Gancao	MOL001792	DFV	ESR1
Gancao	MOL001792	DFV	PTGS2
Gancao	MOL001792	DFV	RXRA
Gancao	MOL001792	DFV	ADRB2
Gancao	MOL001792	DFV	HSP90AB1
Gancao	MOL001792	DFV	DPEP1

Gancao	MOL001792	DFV	MAOB
Gancao	MOL001792	DFV	SLC6A4
Gancao	MOL001792	DFV	PKIA
Gancao	MOL000211	Mairin	PGR
Gancao	MOL002311	Glycyrol	NOS2
Gancao	MOL002311	Glycyrol	ESR1
Gancao	MOL002311	Glycyrol	PPARG
Gancao	MOL002311	Glycyrol	PTGS2
Gancao	MOL002311	Glycyrol	KDR
Gancao	MOL002311	Glycyrol	MAPK14
Gancao	MOL002311	Glycyrol	GSK3B
Gancao	MOL002311	Glycyrol	CHEK1
Gancao	MOL002311	Glycyrol	CCNA2
Gancao	MOL000239	Jaranol	NOS2
Gancao	MOL000239	Jaranol	PTGS1
Gancao	MOL000239	Jaranol	AR
Gancao	MOL000239	Jaranol	SCN5A
Gancao	MOL000239	Jaranol	PTGS2
Gancao	MOL000239	Jaranol	ESR2
Gancao	MOL000239	Jaranol	DPP4
Gancao	MOL000239	Jaranol	HSP90AB1
Gancao	MOL000239	Jaranol	CDK2

Gancao	MOL000239	Jaranol	CHEK1
Gancao	MOL000239	Jaranol	PRSS1
Gancao	MOL000239	Jaranol	NCOA2
Gancao	MOL000239	Jaranol	CAMKMT
Gancao	MOL002565	Medicarpin	NOS2
Gancao	MOL002565	Medicarpin	PTGS1
Gancao	MOL002565	Medicarpin	DRD1
Gancao	MOL002565	Medicarpin	CHRM3
Gancao	MOL002565	Medicarpin	CHRM1
Gancao	MOL002565	Medicarpin	ESR1
Gancao	MOL002565	Medicarpin	SCN5A
Gancao	MOL002565	Medicarpin	CHRM5
Gancao	MOL002565	Medicarpin	PTGS2
Gancao	MOL002565	Medicarpin	CHRM4
Gancao	MOL002565	Medicarpin	RXRA
Gancao	MOL002565	Medicarpin	ADRA1A
Gancao	MOL002565	Medicarpin	CHRM2
Gancao	MOL002565	Medicarpin	ADRA1B
Gancao	MOL002565	Medicarpin	SLC6A3
Gancao	MOL002565	Medicarpin	ADRB2
Gancao	MOL002565	Medicarpin	SLC6A4
Gancao	MOL002565	Medicarpin	OPRM1

Gancao	MOL002565	Medicarpin	ESR2
Gancao	MOL002565	Medicarpin	DPP4
Gancao	MOL002565	Medicarpin	MAPK10
Gancao	MOL002565	Medicarpin	HSP90AB1
Gancao	MOL002565	Medicarpin	CDK2
Gancao	MOL002565	Medicarpin	PRSS1
Gancao	MOL002565	Medicarpin	CCNA2
Gancao	MOL002565	Medicarpin	CAMKMT
Gancao	MOL002565	Medicarpin	OPRD1
Gancao	MOL002565	Medicarpin	ADRA1D
Gancao	MOL000354	isorhamnetin	NOS2
Gancao	MOL000354	isorhamnetin	PTGS1
Gancao	MOL000354	isorhamnetin	ESR1
Gancao	MOL000354	isorhamnetin	AR
Gancao	MOL000354	isorhamnetin	PPARG
Gancao	MOL000354	isorhamnetin	PTGS2
Gancao	MOL000354	isorhamnetin	ESR2
Gancao	MOL000354	isorhamnetin	DPP4
Gancao	MOL000354	isorhamnetin	MAPK14
Gancao	MOL000354	isorhamnetin	GSK3B
Gancao	MOL000354	isorhamnetin	HSP90AB1
Gancao	MOL000354	isorhamnetin	CDK2

Gancao	MOL000354	isorhamnetin	PRSS1
Gancao	MOL000354	isorhamnetin	CCNA2
Gancao	MOL000354	isorhamnetin	NCOA2
Gancao	MOL000354	isorhamnetin	CAMKMT
Gancao	MOL000354	isorhamnetin	PYGM
Gancao	MOL000354	isorhamnetin	PPARD
Gancao	MOL000354	isorhamnetin	CHEK1
Gancao	MOL000354	isorhamnetin	AKR1B1
Gancao	MOL000354	isorhamnetin	NCOA1
Gancao	MOL000354	isorhamnetin	F7
Gancao	MOL000354	isorhamnetin	ACHE
Gancao	MOL000354	isorhamnetin	GABRA1
Gancao	MOL000354	isorhamnetin	MAOB
Gancao	MOL000354	isorhamnetin	GRIA2
Gancao	MOL000354	isorhamnetin	RELA
Gancao	MOL000354	isorhamnetin	NCF1
Gancao	MOL000354	isorhamnetin	OLR1
Gancao	MOL000359	sitosterol	PGR
Gancao	MOL000359	sitosterol	NCOA2
Gancao	MOL000359	sitosterol	NR3C2
Gancao	MOL003656	Lupiwighteone	NOS2
Gancao	MOL003656	Lupiwighteone	ESR1

Gancao	MOL003656	Lupiwighteone	AR
Gancao	MOL003656	Lupiwighteone	SCN5A
Gancao	MOL003656	Lupiwighteone	PPARG
Gancao	MOL003656	Lupiwighteone	PTGS2
Gancao	MOL003656	Lupiwighteone	ESR2
Gancao	MOL003656	Lupiwighteone	DPP4
Gancao	MOL003656	Lupiwighteone	MAPK14
Gancao	MOL003656	Lupiwighteone	GSK3B
Gancao	MOL003656	Lupiwighteone	HSP90AB1
Gancao	MOL003656	Lupiwighteone	CDK2
Gancao	MOL003656	Lupiwighteone	CHEK1
Gancao	MOL003656	Lupiwighteone	PRSS1
Gancao	MOL003656	Lupiwighteone	CCNA2
Gancao	MOL003656	Lupiwighteone	NCOA2
Gancao	MOL003656	Lupiwighteone	CAMKMT
Gancao	MOL003896	7-Methoxy-2-methyl isoflavone	NOS2
Gancao	MOL003896	7-Methoxy-2-methyl isoflavone	PTGS1
Gancao	MOL003896	7-Methoxy-2-methyl isoflavone	DRD1
Gancao	MOL003896	7-Methoxy-2-methyl isoflavone	CHRM3
Gancao	MOL003896	7-Methoxy-2-methyl isoflavone	CHRM1
Gancao	MOL003896	7-Methoxy-2-methyl isoflavone	ESR1
Gancao	MOL003896	7-Methoxy-2-methyl isoflavone	AR

Gancao	MOL003896	7-Methoxy-2-methyl isoflavone	ADRB1
Gancao	MOL003896	7-Methoxy-2-methyl isoflavone	SCN5A
Gancao	MOL003896	7-Methoxy-2-methyl isoflavone	PPARG
Gancao	MOL003896	7-Methoxy-2-methyl isoflavone	PTGS2
Gancao	MOL003896	7-Methoxy-2-methyl isoflavone	RXRA
Gancao	MOL003896	7-Methoxy-2-methyl isoflavone	ACHE
Gancao	MOL003896	7-Methoxy-2-methyl isoflavone	ADRA1B
Gancao	MOL003896	7-Methoxy-2-methyl isoflavone	SLC6A3
Gancao	MOL003896	7-Methoxy-2-methyl isoflavone	ADRB2
Gancao	MOL003896	7-Methoxy-2-methyl isoflavone	ADRA1D
Gancao	MOL003896	7-Methoxy-2-methyl isoflavone	SLC6A4
Gancao	MOL003896	7-Methoxy-2-methyl isoflavone	ESR2
Gancao	MOL003896	7-Methoxy-2-methyl isoflavone	GABRA1
Gancao	MOL003896	7-Methoxy-2-methyl isoflavone	DPP4
Gancao	MOL003896	7-Methoxy-2-methyl isoflavone	MAPK14
Gancao	MOL003896	7-Methoxy-2-methyl isoflavone	GSK3B
Gancao	MOL003896	7-Methoxy-2-methyl isoflavone	HSP90AB1
Gancao	MOL003896	7-Methoxy-2-methyl isoflavone	CDK2
Gancao	MOL003896	7-Methoxy-2-methyl isoflavone	LTA4H
Gancao	MOL003896	7-Methoxy-2-methyl isoflavone	MAOB
Gancao	MOL003896	7-Methoxy-2-methyl isoflavone	CHEK1
Gancao	MOL003896	7-Methoxy-2-methyl isoflavone	IGHG1

Gancao	MOL003896	7-Methoxy-2-methyl isoflavone	PRSS1
Gancao	MOL003896	7-Methoxy-2-methyl isoflavone	CCNA2
Gancao	MOL003896	7-Methoxy-2-methyl isoflavone	NCOA1
Gancao	MOL003896	7-Methoxy-2-methyl isoflavone	PKIA
Gancao	MOL003896	7-Methoxy-2-methyl isoflavone	CAMKMT
Gancao	MOL003896	7-Methoxy-2-methyl isoflavone	CHRM5
Gancao	MOL003896	7-Methoxy-2-methyl isoflavone	OPRM1
Gancao	MOL003896	7-Methoxy-2-methyl isoflavone	NCOA2
Gancao	MOL000392	formononetin	NOS2
Gancao	MOL000392	formononetin	PTGS1
Gancao	MOL000392	formononetin	CHRM1
Gancao	MOL000392	formononetin	ESR1
Gancao	MOL000392	formononetin	AR
Gancao	MOL000392	formononetin	PPARG
Gancao	MOL000392	formononetin	PTGS2
Gancao	MOL000392	formononetin	RXRA
Gancao	MOL000392	formononetin	ADRA1A
Gancao	MOL000392	formononetin	SLC6A3
Gancao	MOL000392	formononetin	ADRB2
Gancao	MOL000392	formononetin	SLC6A4
Gancao	MOL000392	formononetin	ESR2
Gancao	MOL000392	formononetin	DPP4

Gancao	MOL000392	formononetin	MAPK14
Gancao	MOL000392	formononetin	GSK3B
Gancao	MOL000392	formononetin	HSP90AB1
Gancao	MOL000392	formononetin	CDK2
Gancao	MOL000392	formononetin	MAOB
Gancao	MOL000392	formononetin	CHEK1
Gancao	MOL000392	formononetin	PRSS1
Gancao	MOL000392	formononetin	CCNA2
Gancao	MOL000392	formononetin	CAMKMT
Gancao	MOL000392	formononetin	PKIA
Gancao	MOL000392	formononetin	ACHE
Gancao	MOL000392	formononetin	DPEP1
Gancao	MOL000392	formononetin	JUN
Gancao	MOL000392	formononetin	PPARG
Gancao	MOL000392	formononetin	IL4
Gancao	MOL000392	formononetin	ATP5F1B
Gancao	MOL000392	formononetin	HSD3B2
Gancao	MOL000392	formononetin	HSD3B1
Gancao	MOL000417	Calycosin	NOS2
Gancao	MOL000417	Calycosin	PTGS1
Gancao	MOL000417	Calycosin	ESR1
Gancao	MOL000417	Calycosin	AR

Gancao	MOL000417	Calycosin	PPARG
Gancao	MOL000417	Calycosin	PTGS2
Gancao	MOL000417	Calycosin	RXRA
Gancao	MOL000417	Calycosin	ESR2
Gancao	MOL000417	Calycosin	DPP4
Gancao	MOL000417	Calycosin	MAPK14
Gancao	MOL000417	Calycosin	GSK3B
Gancao	MOL000417	Calycosin	HSP90AB1
Gancao	MOL000417	Calycosin	CDK2
Gancao	MOL000417	Calycosin	CHEK1
Gancao	MOL000417	Calycosin	PRSS1
Gancao	MOL000417	Calycosin	CCNA2
Gancao	MOL000417	Calycosin	NCOA2
Gancao	MOL000417	Calycosin	CAMKMT
Gancao	MOL000417	Calycosin	ADRB2
Gancao	MOL000422	kaempferol	NOS2
Gancao	MOL000422	kaempferol	PTGS1
Gancao	MOL000422	kaempferol	AR
Gancao	MOL000422	kaempferol	PPARG
Gancao	MOL000422	kaempferol	PTGS2
Gancao	MOL000422	kaempferol	HSP90AB1
Gancao	MOL000422	kaempferol	NCOA2

Gancao	MOL000422	kaempferol	DPP4
Gancao	MOL000422	kaempferol	PRSS1
Gancao	MOL000422	kaempferol	PGR
Gancao	MOL000422	kaempferol	CHRM1
Gancao	MOL000422	kaempferol	ACHE
Gancao	MOL000422	kaempferol	SLC6A2
Gancao	MOL000422	kaempferol	CHRM2
Gancao	MOL000422	kaempferol	ADRA1B
Gancao	MOL000422	kaempferol	GABRA1
Gancao	MOL000422	kaempferol	F7
Gancao	MOL000422	kaempferol	CAMKMT
Gancao	MOL000422	kaempferol	RELA
Gancao	MOL000422	kaempferol	IKBKB
Gancao	MOL000422	kaempferol	AKT1
Gancao	MOL000422	kaempferol	BCL2
Gancao	MOL000422	kaempferol	BAX
Gancao	MOL000422	kaempferol	TNFAIP6
Gancao	MOL000422	kaempferol	JUN
Gancao	MOL000422	kaempferol	AHSA1
Gancao	MOL000422	kaempferol	CASP3
Gancao	MOL000422	kaempferol	MAPK8
Gancao	MOL000422	kaempferol	MMP1

Gancao	MOL000422	kaempferol	STAT1
Gancao	MOL000422	kaempferol	CDK1
Gancao	MOL000422	kaempferol	PPARG
Gancao	MOL000422	kaempferol	HMOX1
Gancao	MOL000422	kaempferol	CYP3A4
Gancao	MOL000422	kaempferol	CYP1A2
Gancao	MOL000422	kaempferol	CYP1A1
Gancao	MOL000422	kaempferol	ICAM1
Gancao	MOL000422	kaempferol	SELE
Gancao	MOL000422	kaempferol	VCAM1
Gancao	MOL000422	kaempferol	NR1I2
Gancao	MOL000422	kaempferol	CYP1B1
Gancao	MOL000422	kaempferol	ALOX5
Gancao	MOL000422	kaempferol	HAS2
Gancao	MOL000422	kaempferol	GSTP1
Gancao	MOL000422	kaempferol	AHR
Gancao	MOL000422	kaempferol	PSMD3
Gancao	MOL000422	kaempferol	SLC2A4
Gancao	MOL000422	kaempferol	NR1I3
Gancao	MOL000422	kaempferol	INSRR
Gancao	MOL000422	kaempferol	DIO1
Gancao	MOL000422	kaempferol	PPP3CA

Gancao	MOL000422	kaempferol	GSTM1
Gancao	MOL000422	kaempferol	GSTM2
Gancao	MOL000422	kaempferol	AKR1C3
Gancao	MOL000422	kaempferol	SLPI
Gancao	MOL004328	naringenin	PTGS1
Gancao	MOL004328	naringenin	ESR1
Gancao	MOL004328	naringenin	PTGS2
Gancao	MOL004328	naringenin	HSP90AB1
Gancao	MOL004328	naringenin	DPEP1
Gancao	MOL004328	naringenin	RELA
Gancao	MOL004328	naringenin	AKT1
Gancao	MOL004328	naringenin	BCL2
Gancao	MOL004328	naringenin	MAPK3
Gancao	MOL004328	naringenin	MAPK1
Gancao	MOL004328	naringenin	CASP3
Gancao	MOL004328	naringenin	FASN
Gancao	MOL004328	naringenin	LDLR
Gancao	MOL004328	naringenin	BAD
Gancao	MOL004328	naringenin	SOD1
Gancao	MOL004328	naringenin	CAT
Gancao	MOL004328	naringenin	PPARG
Gancao	MOL004328	naringenin	MTTP

Gancao	MOL004328	naringenin	APOB
Gancao	MOL004328	naringenin	PLB1
Gancao	MOL004328	naringenin	HMGCR
Gancao	MOL004328	naringenin	CYP19A1
Gancao	MOL004328	naringenin	GSTP1
Gancao	MOL004328	naringenin	UGT1A1
Gancao	MOL004328	naringenin	PPARA
Gancao	MOL004328	naringenin	SREBF1
Gancao	MOL004328	naringenin	GSR
Gancao	MOL004328	naringenin	ABCC1
Gancao	MOL004328	naringenin	ADIPOR2
Gancao	MOL004328	naringenin	SOAT2
Gancao	MOL004328	naringenin	AKR1C1
Gancao	MOL004328	naringenin	GOT1
Gancao	MOL004328	naringenin	ABAT
Gancao	MOL004328	naringenin	CES1
Gancao	MOL004328	naringenin	SOAT1
Gancao	MOL004805	(2S)-2-[4-hydroxy-3-(3-methylbut-2-enyl)phenyl]-8,8-dimethyl-2,3-dihydropyrano[2,3-f]chromen-4-one	NOS2
Gancao	MOL004805	(2S)-2-[4-hydroxy-3-(3-methylbut-2-enyl)phenyl]-8,8-dimethyl-2,3-dihydropyrano[2,3-f]chromen-4-one	KCNH2
Gancao	MOL004805	(2S)-2-[4-hydroxy-3-(3-methylbut-2-enyl)phenyl]-8,8-dimethyl-2,3-dihydropyrano[2,3-f]chromen-4-one	ESR1
Gancao	MOL0048	(2S)-2-[4-hydroxy-3-(3-methylbut-2-enyl)pheny	AR

	05	l]-8,8-dimethyl-2,3-dihydropyrano[2,3-f]chromen-4-one	
Gancao	MOL004805	(2S)-2-[4-hydroxy-3-(3-methylbut-2-enyl)phenyl]-8,8-dimethyl-2,3-dihydropyrano[2,3-f]chromen-4-one	PPARG
Gancao	MOL004805	(2S)-2-[4-hydroxy-3-(3-methylbut-2-enyl)phenyl]-8,8-dimethyl-2,3-dihydropyrano[2,3-f]chromen-4-one	PTGS2
Gancao	MOL004805	(2S)-2-[4-hydroxy-3-(3-methylbut-2-enyl)phenyl]-8,8-dimethyl-2,3-dihydropyrano[2,3-f]chromen-4-one	ESR2
Gancao	MOL004805	(2S)-2-[4-hydroxy-3-(3-methylbut-2-enyl)phenyl]-8,8-dimethyl-2,3-dihydropyrano[2,3-f]chromen-4-one	MAPK14
Gancao	MOL004805	(2S)-2-[4-hydroxy-3-(3-methylbut-2-enyl)phenyl]-8,8-dimethyl-2,3-dihydropyrano[2,3-f]chromen-4-one	GSK3B
Gancao	MOL004805	(2S)-2-[4-hydroxy-3-(3-methylbut-2-enyl)phenyl]-8,8-dimethyl-2,3-dihydropyrano[2,3-f]chromen-4-one	CAMKMT
Gancao	MOL004806	euchrenone	NOS2
Gancao	MOL004806	euchrenone	KCNH2
Gancao	MOL004806	euchrenone	ESR1
Gancao	MOL004806	euchrenone	SCN5A
Gancao	MOL004806	euchrenone	PTGS2
Gancao	MOL004806	euchrenone	ESR2
Gancao	MOL004806	euchrenone	BACE2
Gancao	MOL004806	euchrenone	CAMKMT
Gancao	MOL004808	glyasperin B	NOS2
Gancao	MOL004808	glyasperin B	ESR1
Gancao	MOL004808	glyasperin B	AR
Gancao	MOL004808	glyasperin B	PPARG

Gancao	MOL004808	glyasperin B	PTGS2
Gancao	MOL004808	glyasperin B	F7
Gancao	MOL004808	glyasperin B	KDR
Gancao	MOL004808	glyasperin B	ACHE
Gancao	MOL004808	glyasperin B	ESR2
Gancao	MOL004808	glyasperin B	DPP4
Gancao	MOL004808	glyasperin B	GSK3B
Gancao	MOL004808	glyasperin B	HSP90AB1
Gancao	MOL004808	glyasperin B	CDK2
Gancao	MOL004808	glyasperin B	PRSS1
Gancao	MOL004808	glyasperin B	CCNA2
Gancao	MOL004808	glyasperin B	NCOA2
Gancao	MOL004808	glyasperin B	CAMKMT
Gancao	MOL004810	glyasperin F	NOS2
Gancao	MOL004810	glyasperin F	PTGS1
Gancao	MOL004810	glyasperin F	ESR1
Gancao	MOL004810	glyasperin F	AR
Gancao	MOL004810	glyasperin F	SCN5A
Gancao	MOL004810	glyasperin F	PPARG
Gancao	MOL004810	glyasperin F	PTGS2
Gancao	MOL004810	glyasperin F	ESR2
Gancao	MOL004810	glyasperin F	MAPK14

Gancao	MOL004810	glyasperin F	GSK3B
Gancao	MOL004810	glyasperin F	HSP90AB1
Gancao	MOL004810	glyasperin F	CDK2
Gancao	MOL004810	glyasperin F	PRSS1
Gancao	MOL004810	glyasperin F	CCNA2
Gancao	MOL004810	glyasperin F	CAMKMT
Gancao	MOL004811	Glyasperin C	NOS2
Gancao	MOL004811	Glyasperin C	KCNH2
Gancao	MOL004811	Glyasperin C	ESR1
Gancao	MOL004811	Glyasperin C	AR
Gancao	MOL004811	Glyasperin C	SCN5A
Gancao	MOL004811	Glyasperin C	PPARG
Gancao	MOL004811	Glyasperin C	PTGS2
Gancao	MOL004811	Glyasperin C	RXRA
Gancao	MOL004811	Glyasperin C	ACHE
Gancao	MOL004811	Glyasperin C	ESR2
Gancao	MOL004811	Glyasperin C	DPP4
Gancao	MOL004811	Glyasperin C	MAPK14
Gancao	MOL004811	Glyasperin C	GSK3B
Gancao	MOL004811	Glyasperin C	HSP90AB1
Gancao	MOL004811	Glyasperin C	CDK2
Gancao	MOL004811	Glyasperin C	CHEK1

Gancao	MOL004811	Glyasperin C	PRSS1
Gancao	MOL004811	Glyasperin C	CCNA2
Gancao	MOL004811	Glyasperin C	NCOA2
Gancao	MOL004811	Glyasperin C	CAMKMT
Gancao	MOL004814	Isotrifoliol	NOS2
Gancao	MOL004814	Isotrifoliol	ESR1
Gancao	MOL004814	Isotrifoliol	AR
Gancao	MOL004814	Isotrifoliol	PTGS2
Gancao	MOL004814	Isotrifoliol	ESR2
Gancao	MOL004814	Isotrifoliol	MAPK14
Gancao	MOL004814	Isotrifoliol	GSK3B
Gancao	MOL004814	Isotrifoliol	HSP90AB1
Gancao	MOL004814	Isotrifoliol	CDK2
Gancao	MOL004814	Isotrifoliol	CHEK1
Gancao	MOL004814	Isotrifoliol	CCNA2
Gancao	MOL004815	(E)-1-(2,4-dihydroxyphenyl)-3-(2,2-dimethylchromen-6-yl)prop-2-en-1-one	NOS2
Gancao	MOL004815	(E)-1-(2,4-dihydroxyphenyl)-3-(2,2-dimethylchromen-6-yl)prop-2-en-1-one	PTGS1
Gancao	MOL004815	(E)-1-(2,4-dihydroxyphenyl)-3-(2,2-dimethylchromen-6-yl)prop-2-en-1-one	ESR1
Gancao	MOL004815	(E)-1-(2,4-dihydroxyphenyl)-3-(2,2-dimethylchromen-6-yl)prop-2-en-1-one	AR
Gancao	MOL004815	(E)-1-(2,4-dihydroxyphenyl)-3-(2,2-dimethylchromen-6-yl)prop-2-en-1-one	SCN5A
Gancao	MOL004815	(E)-1-(2,4-dihydroxyphenyl)-3-(2,2-dimethylchromen-6-yl)prop-2-en-1-one	PPARG
Gancao	MOL004815	(E)-1-(2,4-dihydroxyphenyl)-3-(2,2-dimethylchromen-6-yl)prop-2-en-1-one	PTGS2

Gancao	MOL004815	(E)-1-(2,4-dihydroxyphenyl)-3-(2,2-dimethylchromen-6-yl)prop-2-en-1-one	CA2
Gancao	MOL004815	(E)-1-(2,4-dihydroxyphenyl)-3-(2,2-dimethylchromen-6-yl)prop-2-en-1-one	RXRA
Gancao	MOL004815	(E)-1-(2,4-dihydroxyphenyl)-3-(2,2-dimethylchromen-6-yl)prop-2-en-1-one	ADRA1B
Gancao	MOL004815	(E)-1-(2,4-dihydroxyphenyl)-3-(2,2-dimethylchromen-6-yl)prop-2-en-1-one	ESR2
Gancao	MOL004815	(E)-1-(2,4-dihydroxyphenyl)-3-(2,2-dimethylchromen-6-yl)prop-2-en-1-one	MAPK14
Gancao	MOL004815	(E)-1-(2,4-dihydroxyphenyl)-3-(2,2-dimethylchromen-6-yl)prop-2-en-1-one	GSK3B
Gancao	MOL004815	(E)-1-(2,4-dihydroxyphenyl)-3-(2,2-dimethylchromen-6-yl)prop-2-en-1-one	CDK2
Gancao	MOL004815	(E)-1-(2,4-dihydroxyphenyl)-3-(2,2-dimethylchromen-6-yl)prop-2-en-1-one	CHEK1
Gancao	MOL004815	(E)-1-(2,4-dihydroxyphenyl)-3-(2,2-dimethylchromen-6-yl)prop-2-en-1-one	CCNA2
Gancao	MOL004815	(E)-1-(2,4-dihydroxyphenyl)-3-(2,2-dimethylchromen-6-yl)prop-2-en-1-one	NCOA2
Gancao	MOL004815	(E)-1-(2,4-dihydroxyphenyl)-3-(2,2-dimethylchromen-6-yl)prop-2-en-1-one	CAMKMT
Gancao	MOL004820	kanzonols W	NOS2
Gancao	MOL004820	kanzonols W	PTGS1
Gancao	MOL004820	kanzonols W	ESR1
Gancao	MOL004820	kanzonols W	AR
Gancao	MOL004820	kanzonols W	SCN5A
Gancao	MOL004820	kanzonols W	PPARG
Gancao	MOL004820	kanzonols W	PTGS2
Gancao	MOL004820	kanzonols W	RXRA
Gancao	MOL004820	kanzonols W	ESR2
Gancao	MOL004820	kanzonols W	MAPK14
Gancao	MOL004820	kanzonols W	GSK3B

Gancao	MOL004820	kanzonols W	CDK2
Gancao	MOL004820	kanzonols W	CHEK1
Gancao	MOL004820	kanzonols W	PRSS1
Gancao	MOL004820	kanzonols W	CCNA2
Gancao	MOL004820	kanzonols W	NCOA2
Gancao	MOL004820	kanzonols W	NCOA1
Gancao	MOL004820	kanzonols W	CAMKMT
Gancao	MOL004824	(2S)-6-(2,4-dihydroxyphenyl)-2-(2-hydroxypropan-2-yl)-4-methoxy-2,3-dihydrofuro[3,2-g]chromen-7-one	NOS2
Gancao	MOL004824	(2S)-6-(2,4-dihydroxyphenyl)-2-(2-hydroxypropan-2-yl)-4-methoxy-2,3-dihydrofuro[3,2-g]chromen-7-one	ESR1
Gancao	MOL004824	(2S)-6-(2,4-dihydroxyphenyl)-2-(2-hydroxypropan-2-yl)-4-methoxy-2,3-dihydrofuro[3,2-g]chromen-7-one	AR
Gancao	MOL004824	(2S)-6-(2,4-dihydroxyphenyl)-2-(2-hydroxypropan-2-yl)-4-methoxy-2,3-dihydrofuro[3,2-g]chromen-7-one	PPARG
Gancao	MOL004824	(2S)-6-(2,4-dihydroxyphenyl)-2-(2-hydroxypropan-2-yl)-4-methoxy-2,3-dihydrofuro[3,2-g]chromen-7-one	PTGS2
Gancao	MOL004824	(2S)-6-(2,4-dihydroxyphenyl)-2-(2-hydroxypropan-2-yl)-4-methoxy-2,3-dihydrofuro[3,2-g]chromen-7-one	F7
Gancao	MOL004824	(2S)-6-(2,4-dihydroxyphenyl)-2-(2-hydroxypropan-2-yl)-4-methoxy-2,3-dihydrofuro[3,2-g]chromen-7-one	KDR
Gancao	MOL004824	(2S)-6-(2,4-dihydroxyphenyl)-2-(2-hydroxypropan-2-yl)-4-methoxy-2,3-dihydrofuro[3,2-g]chromen-7-one	ACHE
Gancao	MOL004824	(2S)-6-(2,4-dihydroxyphenyl)-2-(2-hydroxypropan-2-yl)-4-methoxy-2,3-dihydrofuro[3,2-g]chromen-7-one	ESR2
Gancao	MOL004824	(2S)-6-(2,4-dihydroxyphenyl)-2-(2-hydroxypropan-2-yl)-4-methoxy-2,3-dihydrofuro[3,2-g]chromen-7-one	DPP4

Gancao	MOL004824	(2S)-6-(2,4-dihydroxyphenyl)-2-(2-hydroxypropan-2-yl)-4-methoxy-2,3-dihydrofuro[3,2-g]chromen-7-one	MAPK14
Gancao	MOL004824	(2S)-6-(2,4-dihydroxyphenyl)-2-(2-hydroxypropan-2-yl)-4-methoxy-2,3-dihydrofuro[3,2-g]chromen-7-one	GSK3B
Gancao	MOL004824	(2S)-6-(2,4-dihydroxyphenyl)-2-(2-hydroxypropan-2-yl)-4-methoxy-2,3-dihydrofuro[3,2-g]chromen-7-one	CDK2
Gancao	MOL004824	(2S)-6-(2,4-dihydroxyphenyl)-2-(2-hydroxypropan-2-yl)-4-methoxy-2,3-dihydrofuro[3,2-g]chromen-7-one	CHEK1
Gancao	MOL004824	(2S)-6-(2,4-dihydroxyphenyl)-2-(2-hydroxypropan-2-yl)-4-methoxy-2,3-dihydrofuro[3,2-g]chromen-7-one	PRSS1
Gancao	MOL004824	(2S)-6-(2,4-dihydroxyphenyl)-2-(2-hydroxypropan-2-yl)-4-methoxy-2,3-dihydrofuro[3,2-g]chromen-7-one	CCNA2
Gancao	MOL004824	(2S)-6-(2,4-dihydroxyphenyl)-2-(2-hydroxypropan-2-yl)-4-methoxy-2,3-dihydrofuro[3,2-g]chromen-7-one	CAMKMT
Gancao	MOL004827	Semilicoisoflavone B	NOS2
Gancao	MOL004827	Semilicoisoflavone B	ESR1
Gancao	MOL004827	Semilicoisoflavone B	AR
Gancao	MOL004827	Semilicoisoflavone B	SCN5A
Gancao	MOL004827	Semilicoisoflavone B	PPARG
Gancao	MOL004827	Semilicoisoflavone B	PTGS2
Gancao	MOL004827	Semilicoisoflavone B	F7
Gancao	MOL004827	Semilicoisoflavone B	ACHE
Gancao	MOL004827	Semilicoisoflavone B	GSK3B
Gancao	MOL004827	Semilicoisoflavone B	HSP90AB1
Gancao	MOL004827	Semilicoisoflavone B	CDK2
Gancao	MOL0048	Semilicoisoflavone B	CHEK1

	27		
Gancao	MOL004827	Semilicoisoflavone B	PRSS1
Gancao	MOL004827	Semilicoisoflavone B	CAMKMT
Gancao	MOL004828	Glepidotin A	NOS2
Gancao	MOL004828	Glepidotin A	PTGS1
Gancao	MOL004828	Glepidotin A	ESR1
Gancao	MOL004828	Glepidotin A	AR
Gancao	MOL004828	Glepidotin A	SCN5A
Gancao	MOL004828	Glepidotin A	PPARG
Gancao	MOL004828	Glepidotin A	PTGS2
Gancao	MOL004828	Glepidotin A	F7
Gancao	MOL004828	Glepidotin A	KDR
Gancao	MOL004828	Glepidotin A	RXRA
Gancao	MOL004828	Glepidotin A	DPP4
Gancao	MOL004828	Glepidotin A	MAPK14
Gancao	MOL004828	Glepidotin A	GSK3B
Gancao	MOL004828	Glepidotin A	HSP90AB1
Gancao	MOL004828	Glepidotin A	CDK2
Gancao	MOL004828	Glepidotin A	CHEK1
Gancao	MOL004828	Glepidotin A	IGHG1
Gancao	MOL004828	Glepidotin A	PRSS1
Gancao	MOL004828	Glepidotin A	CCNA2

Gancao	MOL004828	Glepidotin A	CAMKMT
Gancao	MOL004829	Glepidotin B	PTGS1
Gancao	MOL004829	Glepidotin B	ESR1
Gancao	MOL004829	Glepidotin B	SCN5A
Gancao	MOL004829	Glepidotin B	PTGS2
Gancao	MOL004829	Glepidotin B	F7
Gancao	MOL004829	Glepidotin B	RXRA
Gancao	MOL004829	Glepidotin B	ADRA1B
Gancao	MOL004829	Glepidotin B	HSP90AB1
Gancao	MOL004829	Glepidotin B	IGHG1
Gancao	MOL004829	Glepidotin B	NCOA1
Gancao	MOL004829	Glepidotin B	CAMKMT
Gancao	MOL004833	Phaseolinisoflavan	NOS2
Gancao	MOL004833	Phaseolinisoflavan	CHRM1
Gancao	MOL004833	Phaseolinisoflavan	ESR1
Gancao	MOL004833	Phaseolinisoflavan	AR
Gancao	MOL004833	Phaseolinisoflavan	SCN5A
Gancao	MOL004833	Phaseolinisoflavan	PPARG
Gancao	MOL004833	Phaseolinisoflavan	PTGS2
Gancao	MOL004833	Phaseolinisoflavan	RXRA
Gancao	MOL004833	Phaseolinisoflavan	ACHE
Gancao	MOL004833	Phaseolinisoflavan	ADRA1B

Gancao	MOL004833	Phaseolinisoflavan	ADRB2
Gancao	MOL004833	Phaseolinisoflavan	ESR2
Gancao	MOL004833	Phaseolinisoflavan	MAPK14
Gancao	MOL004833	Phaseolinisoflavan	GSK3B
Gancao	MOL004833	Phaseolinisoflavan	CDK2
Gancao	MOL004833	Phaseolinisoflavan	CHEK1
Gancao	MOL004833	Phaseolinisoflavan	PRSS1
Gancao	MOL004833	Phaseolinisoflavan	CCNA2
Gancao	MOL004833	Phaseolinisoflavan	NCOA1
Gancao	MOL004833	Phaseolinisoflavan	CAMKMT
Gancao	MOL004835	Glypallichalcone	NOS2
Gancao	MOL004835	Glypallichalcone	PTGS1
Gancao	MOL004835	Glypallichalcone	CHRM1
Gancao	MOL004835	Glypallichalcone	ESR1
Gancao	MOL004835	Glypallichalcone	AR
Gancao	MOL004835	Glypallichalcone	SCN5A
Gancao	MOL004835	Glypallichalcone	PPARG
Gancao	MOL004835	Glypallichalcone	PTGS2
Gancao	MOL004835	Glypallichalcone	CA2
Gancao	MOL004835	Glypallichalcone	ADRA1B
Gancao	MOL004835	Glypallichalcone	SLC6A3
Gancao	MOL004835	Glypallichalcone	ADRB2

Gancao	MOL004835	Glypallichalcone	SLC6A4
Gancao	MOL004835	Glypallichalcone	ESR2
Gancao	MOL004835	Glypallichalcone	MAPK14
Gancao	MOL004835	Glypallichalcone	GSK3B
Gancao	MOL004835	Glypallichalcone	HSP90AB1
Gancao	MOL004835	Glypallichalcone	CDK2
Gancao	MOL004835	Glypallichalcone	LTA4H
Gancao	MOL004835	Glypallichalcone	MAOB
Gancao	MOL004835	Glypallichalcone	CHEK1
Gancao	MOL004835	Glypallichalcone	CCNA2
Gancao	MOL004835	Glypallichalcone	NCOA1
Gancao	MOL004835	Glypallichalcone	PKIA
Gancao	MOL004835	Glypallichalcone	CAMKMT
Gancao	MOL004838	8-(6-hydroxy-2-benzofuranyl)-2,2-dimethyl-5-chromenol	NOS2
Gancao	MOL004838	8-(6-hydroxy-2-benzofuranyl)-2,2-dimethyl-5-chromenol	ESR1
Gancao	MOL004838	8-(6-hydroxy-2-benzofuranyl)-2,2-dimethyl-5-chromenol	PTGS2
Gancao	MOL004838	8-(6-hydroxy-2-benzofuranyl)-2,2-dimethyl-5-chromenol	RXRA
Gancao	MOL004838	8-(6-hydroxy-2-benzofuranyl)-2,2-dimethyl-5-chromenol	HSP90AB1
Gancao	MOL004841	Licochalcone B	NOS2
Gancao	MOL004841	Licochalcone B	PTGS1
Gancao	MOL004841	Licochalcone B	ESR1
Gancao	MOL004841	Licochalcone B	AR

Gancao	MOL004841	Licochalcone B	PPARG
Gancao	MOL004841	Licochalcone B	PTGS2
Gancao	MOL004841	Licochalcone B	CA2
Gancao	MOL004841	Licochalcone B	ADRB2
Gancao	MOL004841	Licochalcone B	ESR2
Gancao	MOL004841	Licochalcone B	MAPK14
Gancao	MOL004841	Licochalcone B	GSK3B
Gancao	MOL004841	Licochalcone B	HSP90AB1
Gancao	MOL004841	Licochalcone B	CDK2
Gancao	MOL004841	Licochalcone B	CHEK1
Gancao	MOL004841	Licochalcone B	CCNA2
Gancao	MOL004841	Licochalcone B	CAMKMT
Gancao	MOL004848	licochalcone G	NOS2
Gancao	MOL004848	licochalcone G	ESR1
Gancao	MOL004848	licochalcone G	AR
Gancao	MOL004848	licochalcone G	PPARG
Gancao	MOL004848	licochalcone G	PTGS2
Gancao	MOL004848	licochalcone G	KDR
Gancao	MOL004848	licochalcone G	ESR2
Gancao	MOL004848	licochalcone G	MAPK14
Gancao	MOL004848	licochalcone G	GSK3B
Gancao	MOL004848	licochalcone G	HSP90AB1

Gancao	MOL0048 48	licochalcone G	CDK2
Gancao	MOL0048 48	licochalcone G	IGHG1
Gancao	MOL0048 48	licochalcone G	CCNA2
Gancao	MOL0048 48	licochalcone G	NCOA2
Gancao	MOL0048 48	licochalcone G	CAMKM T
Gancao	MOL0048 49	3-(2,4-dihydroxyphenyl)-8-(1,1-dimethylprop-2-enyl)-7-hydroxy-5-methoxy-coumarin	NOS2
Gancao	MOL0048 49	3-(2,4-dihydroxyphenyl)-8-(1,1-dimethylprop-2-enyl)-7-hydroxy-5-methoxy-coumarin	KCNH2
Gancao	MOL0048 49	3-(2,4-dihydroxyphenyl)-8-(1,1-dimethylprop-2-enyl)-7-hydroxy-5-methoxy-coumarin	ESR1
Gancao	MOL0048 49	3-(2,4-dihydroxyphenyl)-8-(1,1-dimethylprop-2-enyl)-7-hydroxy-5-methoxy-coumarin	AR
Gancao	MOL0048 49	3-(2,4-dihydroxyphenyl)-8-(1,1-dimethylprop-2-enyl)-7-hydroxy-5-methoxy-coumarin	PPARG
Gancao	MOL0048 49	3-(2,4-dihydroxyphenyl)-8-(1,1-dimethylprop-2-enyl)-7-hydroxy-5-methoxy-coumarin	PTGS2
Gancao	MOL0048 49	3-(2,4-dihydroxyphenyl)-8-(1,1-dimethylprop-2-enyl)-7-hydroxy-5-methoxy-coumarin	F7
Gancao	MOL0048 49	3-(2,4-dihydroxyphenyl)-8-(1,1-dimethylprop-2-enyl)-7-hydroxy-5-methoxy-coumarin	KDR
Gancao	MOL0048 49	3-(2,4-dihydroxyphenyl)-8-(1,1-dimethylprop-2-enyl)-7-hydroxy-5-methoxy-coumarin	ESR2
Gancao	MOL0048 49	3-(2,4-dihydroxyphenyl)-8-(1,1-dimethylprop-2-enyl)-7-hydroxy-5-methoxy-coumarin	DPP4
Gancao	MOL0048 49	3-(2,4-dihydroxyphenyl)-8-(1,1-dimethylprop-2-enyl)-7-hydroxy-5-methoxy-coumarin	MAPK14
Gancao	MOL0048 49	3-(2,4-dihydroxyphenyl)-8-(1,1-dimethylprop-2-enyl)-7-hydroxy-5-methoxy-coumarin	GSK3B
Gancao	MOL0048 49	3-(2,4-dihydroxyphenyl)-8-(1,1-dimethylprop-2-enyl)-7-hydroxy-5-methoxy-coumarin	HSP90AB 1
Gancao	MOL0048 49	3-(2,4-dihydroxyphenyl)-8-(1,1-dimethylprop-2-enyl)-7-hydroxy-5-methoxy-coumarin	CDK2
Gancao	MOL0048 49	3-(2,4-dihydroxyphenyl)-8-(1,1-dimethylprop-2-enyl)-7-hydroxy-5-methoxy-coumarin	CHEK1
Gancao	MOL0048 49	3-(2,4-dihydroxyphenyl)-8-(1,1-dimethylprop-2-enyl)-7-hydroxy-5-methoxy-coumarin	PRSS1
Gancao	MOL0048 49	3-(2,4-dihydroxyphenyl)-8-(1,1-dimethylprop-2-enyl)-7-hydroxy-5-methoxy-coumarin	NCOA2

Gancao	MOL004849	3-(2,4-dihydroxyphenyl)-8-(1,1-dimethylprop-2-enyl)-7-hydroxy-5-methoxy-coumarin	NCOA1
Gancao	MOL004849	3-(2,4-dihydroxyphenyl)-8-(1,1-dimethylprop-2-enyl)-7-hydroxy-5-methoxy-coumarin	CAMKMT
Gancao	MOL004855	Licoricone	NOS2
Gancao	MOL004855	Licoricone	KCNH2
Gancao	MOL004855	Licoricone	ESR1
Gancao	MOL004855	Licoricone	AR
Gancao	MOL004855	Licoricone	PPARG
Gancao	MOL004855	Licoricone	PTGS2
Gancao	MOL004855	Licoricone	KDR
Gancao	MOL004855	Licoricone	CHEK1
Gancao	MOL004855	Licoricone	PRSS1
Gancao	MOL004855	Licoricone	NCOA2
Gancao	MOL004855	Licoricone	CAMKMT
Gancao	MOL004856	Gancaonin A	NOS2
Gancao	MOL004856	Gancaonin A	ESR1
Gancao	MOL004856	Gancaonin A	AR
Gancao	MOL004856	Gancaonin A	SCN5A
Gancao	MOL004856	Gancaonin A	PPARG
Gancao	MOL004856	Gancaonin A	PTGS2
Gancao	MOL004856	Gancaonin A	ACHE
Gancao	MOL004856	Gancaonin A	ESR2
Gancao	MOL004856	Gancaonin A	DPP4

Gancao	MOL004856	Gancaonin A	GSK3B
Gancao	MOL004856	Gancaonin A	HSP90AB1
Gancao	MOL004856	Gancaonin A	CHEK1
Gancao	MOL004856	Gancaonin A	PRSS1
Gancao	MOL004856	Gancaonin A	CCNA2
Gancao	MOL004856	Gancaonin A	NCOA2
Gancao	MOL004856	Gancaonin A	CAMKMT
Gancao	MOL004857	Gancaonin B	NOS2
Gancao	MOL004857	Gancaonin B	ESR1
Gancao	MOL004857	Gancaonin B	AR
Gancao	MOL004857	Gancaonin B	PPARG
Gancao	MOL004857	Gancaonin B	PTGS2
Gancao	MOL004857	Gancaonin B	F7
Gancao	MOL004857	Gancaonin B	KDR
Gancao	MOL004857	Gancaonin B	ADRA1B
Gancao	MOL004857	Gancaonin B	ADRB2
Gancao	MOL004857	Gancaonin B	ESR2
Gancao	MOL004857	Gancaonin B	DPP4
Gancao	MOL004857	Gancaonin B	GSK3B
Gancao	MOL004857	Gancaonin B	HSP90AB1
Gancao	MOL004857	Gancaonin B	CHEK1
Gancao	MOL004857	Gancaonin B	PRSS1

Gancao	MOL004857	Gancaonin B	CCNA2
Gancao	MOL004857	Gancaonin B	NCOA2
Gancao	MOL004857	Gancaonin B	CAMKMT
Gancao	MOL004863	3-(3,4-dihydroxyphenyl)-5,7-dihydroxy-8-(3-methylbut-2-enyl)chromone	NOS2
Gancao	MOL004863	3-(3,4-dihydroxyphenyl)-5,7-dihydroxy-8-(3-methylbut-2-enyl)chromone	ESR1
Gancao	MOL004863	3-(3,4-dihydroxyphenyl)-5,7-dihydroxy-8-(3-methylbut-2-enyl)chromone	AR
Gancao	MOL004863	3-(3,4-dihydroxyphenyl)-5,7-dihydroxy-8-(3-methylbut-2-enyl)chromone	PPARG
Gancao	MOL004863	3-(3,4-dihydroxyphenyl)-5,7-dihydroxy-8-(3-methylbut-2-enyl)chromone	PTGS2
Gancao	MOL004863	3-(3,4-dihydroxyphenyl)-5,7-dihydroxy-8-(3-methylbut-2-enyl)chromone	MAPK14
Gancao	MOL004863	3-(3,4-dihydroxyphenyl)-5,7-dihydroxy-8-(3-methylbut-2-enyl)chromone	GSK3B
Gancao	MOL004863	3-(3,4-dihydroxyphenyl)-5,7-dihydroxy-8-(3-methylbut-2-enyl)chromone	HSP90AB1
Gancao	MOL004863	3-(3,4-dihydroxyphenyl)-5,7-dihydroxy-8-(3-methylbut-2-enyl)chromone	CDK2
Gancao	MOL004863	3-(3,4-dihydroxyphenyl)-5,7-dihydroxy-8-(3-methylbut-2-enyl)chromone	CHEK1
Gancao	MOL004863	3-(3,4-dihydroxyphenyl)-5,7-dihydroxy-8-(3-methylbut-2-enyl)chromone	PRSS1
Gancao	MOL004863	3-(3,4-dihydroxyphenyl)-5,7-dihydroxy-8-(3-methylbut-2-enyl)chromone	CCNA2
Gancao	MOL004863	3-(3,4-dihydroxyphenyl)-5,7-dihydroxy-8-(3-methylbut-2-enyl)chromone	NCOA2
Gancao	MOL004863	3-(3,4-dihydroxyphenyl)-5,7-dihydroxy-8-(3-methylbut-2-enyl)chromone	CAMKMT
Gancao	MOL004864	5,7-dihydroxy-3-(4-methoxyphenyl)-8-(3-methylbut-2-enyl)chromone	NOS2
Gancao	MOL004864	5,7-dihydroxy-3-(4-methoxyphenyl)-8-(3-methylbut-2-enyl)chromone	KCNH2
Gancao	MOL004864	5,7-dihydroxy-3-(4-methoxyphenyl)-8-(3-methylbut-2-enyl)chromone	ESR1
Gancao	MOL004864	5,7-dihydroxy-3-(4-methoxyphenyl)-8-(3-methylbut-2-enyl)chromone	AR
Gancao	MOL004864	5,7-dihydroxy-3-(4-methoxyphenyl)-8-(3-methylbut-2-enyl)chromone	PPARG

Gancao	MOL004864	5,7-dihydroxy-3-(4-methoxyphenyl)-8-(3-methylbut-2-enyl)chromone	PTGS2
Gancao	MOL004864	5,7-dihydroxy-3-(4-methoxyphenyl)-8-(3-methylbut-2-enyl)chromone	ESR2
Gancao	MOL004864	5,7-dihydroxy-3-(4-methoxyphenyl)-8-(3-methylbut-2-enyl)chromone	DPP4
Gancao	MOL004864	5,7-dihydroxy-3-(4-methoxyphenyl)-8-(3-methylbut-2-enyl)chromone	MAPK14
Gancao	MOL004864	5,7-dihydroxy-3-(4-methoxyphenyl)-8-(3-methylbut-2-enyl)chromone	GSK3B
Gancao	MOL004864	5,7-dihydroxy-3-(4-methoxyphenyl)-8-(3-methylbut-2-enyl)chromone	HSP90AB1
Gancao	MOL004864	5,7-dihydroxy-3-(4-methoxyphenyl)-8-(3-methylbut-2-enyl)chromone	CDK2
Gancao	MOL004864	5,7-dihydroxy-3-(4-methoxyphenyl)-8-(3-methylbut-2-enyl)chromone	CHEK1
Gancao	MOL004864	5,7-dihydroxy-3-(4-methoxyphenyl)-8-(3-methylbut-2-enyl)chromone	PRSS1
Gancao	MOL004864	5,7-dihydroxy-3-(4-methoxyphenyl)-8-(3-methylbut-2-enyl)chromone	CCNA2
Gancao	MOL004864	5,7-dihydroxy-3-(4-methoxyphenyl)-8-(3-methylbut-2-enyl)chromone	NCOA2
Gancao	MOL004864	5,7-dihydroxy-3-(4-methoxyphenyl)-8-(3-methylbut-2-enyl)chromone	CAMKMT
Gancao	MOL004866	2-(3,4-dihydroxyphenyl)-5,7-dihydroxy-6-(3-methylbut-2-enyl)chromone	AR
Gancao	MOL004866	2-(3,4-dihydroxyphenyl)-5,7-dihydroxy-6-(3-methylbut-2-enyl)chromone	SCN5A
Gancao	MOL004866	2-(3,4-dihydroxyphenyl)-5,7-dihydroxy-6-(3-methylbut-2-enyl)chromone	PPARG
Gancao	MOL004866	2-(3,4-dihydroxyphenyl)-5,7-dihydroxy-6-(3-methylbut-2-enyl)chromone	PTGS2
Gancao	MOL004866	2-(3,4-dihydroxyphenyl)-5,7-dihydroxy-6-(3-methylbut-2-enyl)chromone	F7
Gancao	MOL004866	2-(3,4-dihydroxyphenyl)-5,7-dihydroxy-6-(3-methylbut-2-enyl)chromone	ADRB2
Gancao	MOL004866	2-(3,4-dihydroxyphenyl)-5,7-dihydroxy-6-(3-methylbut-2-enyl)chromone	DPP4
Gancao	MOL004866	2-(3,4-dihydroxyphenyl)-5,7-dihydroxy-6-(3-methylbut-2-enyl)chromone	HSP90AB1
Gancao	MOL004866	2-(3,4-dihydroxyphenyl)-5,7-dihydroxy-6-(3-methylbut-2-enyl)chromone	CDK2
Gancao	MOL004866	2-(3,4-dihydroxyphenyl)-5,7-dihydroxy-6-(3-methylbut-2-enyl)chromone	CHEK1

Gancao	MOL004866	2-(3,4-dihydroxyphenyl)-5,7-dihydroxy-6-(3-methylbut-2-enyl)chromone	PRSS1
Gancao	MOL004866	2-(3,4-dihydroxyphenyl)-5,7-dihydroxy-6-(3-methylbut-2-enyl)chromone	CCNA2
Gancao	MOL004866	2-(3,4-dihydroxyphenyl)-5,7-dihydroxy-6-(3-methylbut-2-enyl)chromone	CAMKMT
Gancao	MOL004879	Glycyrin	NOS2
Gancao	MOL004879	Glycyrin	KCNH2
Gancao	MOL004879	Glycyrin	ESR1
Gancao	MOL004879	Glycyrin	AR
Gancao	MOL004879	Glycyrin	PPARG
Gancao	MOL004879	Glycyrin	PTGS2
Gancao	MOL004879	Glycyrin	KDR
Gancao	MOL004879	Glycyrin	ESR2
Gancao	MOL004879	Glycyrin	DPP4
Gancao	MOL004879	Glycyrin	CHEK1
Gancao	MOL004879	Glycyrin	PRSS1
Gancao	MOL004879	Glycyrin	NCOA2
Gancao	MOL004879	Glycyrin	CAMKMT
Gancao	MOL004882	Licocoumarone	ESR1
Gancao	MOL004882	Licocoumarone	AR
Gancao	MOL004882	Licocoumarone	ESR2
Gancao	MOL004882	Licocoumarone	GSK3B
Gancao	MOL004882	Licocoumarone	HSP90AB1
Gancao	MOL004882	Licocoumarone	CDK2

Gancao	MOL004882	Licocoumarone	CCNA2
Gancao	MOL004883	Licoisoflavone	NOS2
Gancao	MOL004883	Licoisoflavone	ESR1
Gancao	MOL004883	Licoisoflavone	AR
Gancao	MOL004883	Licoisoflavone	PPARG
Gancao	MOL004883	Licoisoflavone	PTGS2
Gancao	MOL004883	Licoisoflavone	KDR
Gancao	MOL004883	Licoisoflavone	DPP4
Gancao	MOL004883	Licoisoflavone	MAPK14
Gancao	MOL004883	Licoisoflavone	HSP90AB1
Gancao	MOL004883	Licoisoflavone	CDK2
Gancao	MOL004883	Licoisoflavone	CHEK1
Gancao	MOL004883	Licoisoflavone	PRSS1
Gancao	MOL004883	Licoisoflavone	CCNA2
Gancao	MOL004883	Licoisoflavone	NCOA2
Gancao	MOL004883	Licoisoflavone	CAMKMT
Gancao	MOL004884	Licoisoflavone B	NOS2
Gancao	MOL004884	Licoisoflavone B	ESR1
Gancao	MOL004884	Licoisoflavone B	AR
Gancao	MOL004884	Licoisoflavone B	PPARG
Gancao	MOL004884	Licoisoflavone B	PTGS2
Gancao	MOL004884	Licoisoflavone B	ACHE

Gancao	MOL004884	Licoisoflavone B	ESR2
Gancao	MOL004884	Licoisoflavone B	GSK3B
Gancao	MOL004884	Licoisoflavone B	CDK2
Gancao	MOL004884	Licoisoflavone B	CHEK1
Gancao	MOL004884	Licoisoflavone B	PRSS1
Gancao	MOL004884	Licoisoflavone B	CCNA2
Gancao	MOL004884	Licoisoflavone B	CAMKMT
Gancao	MOL004885	licoisoflavanone	NOS2
Gancao	MOL004885	licoisoflavanone	PTGS1
Gancao	MOL004885	licoisoflavanone	ESR1
Gancao	MOL004885	licoisoflavanone	AR
Gancao	MOL004885	licoisoflavanone	SCN5A
Gancao	MOL004885	licoisoflavanone	PPARG
Gancao	MOL004885	licoisoflavanone	PTGS2
Gancao	MOL004885	licoisoflavanone	F7
Gancao	MOL004885	licoisoflavanone	ACHE
Gancao	MOL004885	licoisoflavanone	ESR2
Gancao	MOL004885	licoisoflavanone	GSK3B
Gancao	MOL004885	licoisoflavanone	HSP90AB1
Gancao	MOL004885	licoisoflavanone	CDK2
Gancao	MOL004885	licoisoflavanone	PRSS1
Gancao	MOL004885	licoisoflavanone	CCNA2

Gancao	MOL004885	licoisoflavanone	NCOA1
Gancao	MOL004885	licoisoflavanone	CAMKMT
Gancao	MOL004891	shinpterocarpin	NOS2
Gancao	MOL004891	shinpterocarpin	PTGS1
Gancao	MOL004891	shinpterocarpin	CHRM3
Gancao	MOL004891	shinpterocarpin	KCNH2
Gancao	MOL004891	shinpterocarpin	CHRM1
Gancao	MOL004891	shinpterocarpin	ESR1
Gancao	MOL004891	shinpterocarpin	AR
Gancao	MOL004891	shinpterocarpin	SCN5A
Gancao	MOL004891	shinpterocarpin	PPARG
Gancao	MOL004891	shinpterocarpin	PTGS2
Gancao	MOL004891	shinpterocarpin	HTR3A
Gancao	MOL004891	shinpterocarpin	RXRA
Gancao	MOL004891	shinpterocarpin	OPRD1
Gancao	MOL004891	shinpterocarpin	ADRA1B
Gancao	MOL004891	shinpterocarpin	ADRB2
Gancao	MOL004891	shinpterocarpin	ADRA1D
Gancao	MOL004891	shinpterocarpin	OPRM1
Gancao	MOL004891	shinpterocarpin	ESR2
Gancao	MOL004891	shinpterocarpin	MAPK14
Gancao	MOL004891	shinpterocarpin	GSK3B

Gancao	MOL004891	shinpterocarpin	CDK2
Gancao	MOL004891	shinpterocarpin	RXRB
Gancao	MOL004891	shinpterocarpin	PRSS1
Gancao	MOL004891	shinpterocarpin	CCNA2
Gancao	MOL004891	shinpterocarpin	NCOA1
Gancao	MOL004891	shinpterocarpin	CAMKMT
Gancao	MOL004898	(E)-3-[3,4-dihydroxy-5-(3-methylbut-2-enyl)phenyl]-1-(2,4-dihydroxyphenyl)prop-2-en-1-one	ESR1
Gancao	MOL004898	(E)-3-[3,4-dihydroxy-5-(3-methylbut-2-enyl)phenyl]-1-(2,4-dihydroxyphenyl)prop-2-en-1-one	AR
Gancao	MOL004898	(E)-3-[3,4-dihydroxy-5-(3-methylbut-2-enyl)phenyl]-1-(2,4-dihydroxyphenyl)prop-2-en-1-one	PPARG
Gancao	MOL004898	(E)-3-[3,4-dihydroxy-5-(3-methylbut-2-enyl)phenyl]-1-(2,4-dihydroxyphenyl)prop-2-en-1-one	PTGS2
Gancao	MOL004898	(E)-3-[3,4-dihydroxy-5-(3-methylbut-2-enyl)phenyl]-1-(2,4-dihydroxyphenyl)prop-2-en-1-one	MAPK14
Gancao	MOL004898	(E)-3-[3,4-dihydroxy-5-(3-methylbut-2-enyl)phenyl]-1-(2,4-dihydroxyphenyl)prop-2-en-1-one	GSK3B
Gancao	MOL004898	(E)-3-[3,4-dihydroxy-5-(3-methylbut-2-enyl)phenyl]-1-(2,4-dihydroxyphenyl)prop-2-en-1-one	HSP90AB1
Gancao	MOL004898	(E)-3-[3,4-dihydroxy-5-(3-methylbut-2-enyl)phenyl]-1-(2,4-dihydroxyphenyl)prop-2-en-1-one	CDK2
Gancao	MOL004898	(E)-3-[3,4-dihydroxy-5-(3-methylbut-2-enyl)phenyl]-1-(2,4-dihydroxyphenyl)prop-2-en-1-one	CCNA2
Gancao	MOL004898	(E)-3-[3,4-dihydroxy-5-(3-methylbut-2-enyl)phenyl]-1-(2,4-dihydroxyphenyl)prop-2-en-1-one	NCOA2
Gancao	MOL004898	(E)-3-[3,4-dihydroxy-5-(3-methylbut-2-enyl)phenyl]-1-(2,4-dihydroxyphenyl)prop-2-en-1-one	CAMKMT
Gancao	MOL004903	liquiritin	F7
Gancao	MOL004903	liquiritin	CAMKMT
Gancao	MOL004903	liquiritin	PTGS2
Gancao	MOL004903	liquiritin	KDR
Gancao	MOL004903	liquiritin	SOD1

Gancao	MOL004904	licopyranocoumarin	NOS2
Gancao	MOL004904	licopyranocoumarin	ESR1
Gancao	MOL004904	licopyranocoumarin	AR
Gancao	MOL004904	licopyranocoumarin	PPARG
Gancao	MOL004904	licopyranocoumarin	PTGS2
Gancao	MOL004904	licopyranocoumarin	F7
Gancao	MOL004904	licopyranocoumarin	KDR
Gancao	MOL004904	licopyranocoumarin	ACHE
Gancao	MOL004904	licopyranocoumarin	CDK2
Gancao	MOL004904	licopyranocoumarin	PRSS1
Gancao	MOL004904	licopyranocoumarin	CCNA2
Gancao	MOL004904	licopyranocoumarin	CAMKMT
Gancao	MOL004907	Glyzaglabrin	NOS2
Gancao	MOL004907	Glyzaglabrin	PTGS1
Gancao	MOL004907	Glyzaglabrin	ESR1
Gancao	MOL004907	Glyzaglabrin	AR
Gancao	MOL004907	Glyzaglabrin	PPARG
Gancao	MOL004907	Glyzaglabrin	PTGS2
Gancao	MOL004907	Glyzaglabrin	ESR2
Gancao	MOL004907	Glyzaglabrin	DPP4
Gancao	MOL004907	Glyzaglabrin	MAPK14
Gancao	MOL004907	Glyzaglabrin	GSK3B

Gancao	MOL004907	Glyzaglabrin	HSP90AB1
Gancao	MOL004907	Glyzaglabrin	CDK2
Gancao	MOL004907	Glyzaglabrin	CHEK1
Gancao	MOL004907	Glyzaglabrin	PRSS1
Gancao	MOL004907	Glyzaglabrin	CCNA2
Gancao	MOL004908	Glabridin	NOS2
Gancao	MOL004908	Glabridin	CHRM1
Gancao	MOL004908	Glabridin	ESR1
Gancao	MOL004908	Glabridin	AR
Gancao	MOL004908	Glabridin	SCN5A
Gancao	MOL004908	Glabridin	PPARG
Gancao	MOL004908	Glabridin	PTGS2
Gancao	MOL004908	Glabridin	RXRA
Gancao	MOL004908	Glabridin	ACHE
Gancao	MOL004908	Glabridin	ADRA1B
Gancao	MOL004908	Glabridin	ADRB2
Gancao	MOL004908	Glabridin	ESR2
Gancao	MOL004908	Glabridin	MAPK14
Gancao	MOL004908	Glabridin	GSK3B
Gancao	MOL004908	Glabridin	CDK2
Gancao	MOL004908	Glabridin	CHEK1
Gancao	MOL004908	Glabridin	RXRB

Gancao	MOL004908	Glabridin	IGHG1
Gancao	MOL004908	Glabridin	PRSS1
Gancao	MOL004908	Glabridin	CCNA2
Gancao	MOL004908	Glabridin	NCOA2
Gancao	MOL004908	Glabridin	NCOA1
Gancao	MOL004908	Glabridin	CAMKMT
Gancao	MOL004910	Glabranin	NOS2
Gancao	MOL004910	Glabranin	PTGS1
Gancao	MOL004910	Glabranin	ESR1
Gancao	MOL004910	Glabranin	SCN5A
Gancao	MOL004910	Glabranin	PTGS2
Gancao	MOL004910	Glabranin	HSP90AB1
Gancao	MOL004910	Glabranin	CAMKMT
Gancao	MOL004911	Glabrene	NOS2
Gancao	MOL004911	Glabrene	PTGS1
Gancao	MOL004911	Glabrene	ESR1
Gancao	MOL004911	Glabrene	AR
Gancao	MOL004911	Glabrene	SCN5A
Gancao	MOL004911	Glabrene	PPARG
Gancao	MOL004911	Glabrene	PTGS2
Gancao	MOL004911	Glabrene	RXRA
Gancao	MOL004911	Glabrene	ADRB2

Gancao	MOL004911	Glabrene	ESR2
Gancao	MOL004911	Glabrene	MAPK14
Gancao	MOL004911	Glabrene	GSK3B
Gancao	MOL004911	Glabrene	HSP90AB1
Gancao	MOL004911	Glabrene	CDK2
Gancao	MOL004911	Glabrene	PRSS1
Gancao	MOL004911	Glabrene	NCOA2
Gancao	MOL004911	Glabrene	CAMKMT
Gancao	MOL004912	Glabrone	NOS2
Gancao	MOL004912	Glabrone	PTGS1
Gancao	MOL004912	Glabrone	ESR1
Gancao	MOL004912	Glabrone	AR
Gancao	MOL004912	Glabrone	SCN5A
Gancao	MOL004912	Glabrone	PPARG
Gancao	MOL004912	Glabrone	PTGS2
Gancao	MOL004912	Glabrone	RXRA
Gancao	MOL004912	Glabrone	ACHE
Gancao	MOL004912	Glabrone	ESR2
Gancao	MOL004912	Glabrone	DPP4
Gancao	MOL004912	Glabrone	MAPK14
Gancao	MOL004912	Glabrone	GSK3B
Gancao	MOL004912	Glabrone	CDK2

Gancao	MOL004912	Glabrone	CHEK1
Gancao	MOL004912	Glabrone	PRSS1
Gancao	MOL004912	Glabrone	CCNA2
Gancao	MOL004912	Glabrone	CAMKMT
Gancao	MOL004913	1,3-dihydroxy-9-methoxy-6-benzofurano[3,2-c]chromenone	ESR1
Gancao	MOL004913	1,3-dihydroxy-9-methoxy-6-benzofurano[3,2-c]chromenone	PPARG
Gancao	MOL004913	1,3-dihydroxy-9-methoxy-6-benzofurano[3,2-c]chromenone	ESR2
Gancao	MOL004913	1,3-dihydroxy-9-methoxy-6-benzofurano[3,2-c]chromenone	MAPK14
Gancao	MOL004913	1,3-dihydroxy-9-methoxy-6-benzofurano[3,2-c]chromenone	GSK3B
Gancao	MOL004913	1,3-dihydroxy-9-methoxy-6-benzofurano[3,2-c]chromenone	HSP90AB1
Gancao	MOL004913	1,3-dihydroxy-9-methoxy-6-benzofurano[3,2-c]chromenone	CDK2
Gancao	MOL004913	1,3-dihydroxy-9-methoxy-6-benzofurano[3,2-c]chromenone	CHEK1
Gancao	MOL004913	1,3-dihydroxy-9-methoxy-6-benzofurano[3,2-c]chromenone	CCNA2
Gancao	MOL004914	1,3-dihydroxy-8,9-dimethoxy-6-benzofurano[3,2-c]chromenone	ESR1
Gancao	MOL004914	1,3-dihydroxy-8,9-dimethoxy-6-benzofurano[3,2-c]chromenone	AR
Gancao	MOL004914	1,3-dihydroxy-8,9-dimethoxy-6-benzofurano[3,2-c]chromenone	PPARG
Gancao	MOL004914	1,3-dihydroxy-8,9-dimethoxy-6-benzofurano[3,2-c]chromenone	MAPK14
Gancao	MOL004914	1,3-dihydroxy-8,9-dimethoxy-6-benzofurano[3,2-c]chromenone	GSK3B
Gancao	MOL004914	1,3-dihydroxy-8,9-dimethoxy-6-benzofurano[3,2-c]chromenone	HSP90AB1
Gancao	MOL004914	1,3-dihydroxy-8,9-dimethoxy-6-benzofurano[3,2-c]chromenone	CDK2
Gancao	MOL004914	1,3-dihydroxy-8,9-dimethoxy-6-benzofurano[3,2-c]chromenone	CHEK1
Gancao	MOL004915	Eurycarpin A	NOS2

Gancao	MOL004915	Eurycarpin A	ESR1
Gancao	MOL004915	Eurycarpin A	AR
Gancao	MOL004915	Eurycarpin A	SCN5A
Gancao	MOL004915	Eurycarpin A	PPARG
Gancao	MOL004915	Eurycarpin A	PTGS2
Gancao	MOL004915	Eurycarpin A	ESR2
Gancao	MOL004915	Eurycarpin A	DPP4
Gancao	MOL004915	Eurycarpin A	MAPK14
Gancao	MOL004915	Eurycarpin A	GSK3B
Gancao	MOL004915	Eurycarpin A	HSP90AB1
Gancao	MOL004915	Eurycarpin A	CDK2
Gancao	MOL004915	Eurycarpin A	CHEK1
Gancao	MOL004915	Eurycarpin A	PRSS1
Gancao	MOL004915	Eurycarpin A	CCNA2
Gancao	MOL004915	Eurycarpin A	CAMKMT
Gancao	MOL004924	(-)-Medicocarpin	PTGS2
Gancao	MOL004924	(-)-Medicocarpin	ACHE
Gancao	MOL004935	Sigmoidin-B	ESR1
Gancao	MOL004935	Sigmoidin-B	PTGS2
Gancao	MOL004935	Sigmoidin-B	KDR
Gancao	MOL004935	Sigmoidin-B	HSP90AB1
Gancao	MOL004935	Sigmoidin-B	CAMKMT

Gancao	MOL004941	(2R)-7-hydroxy-2-(4-hydroxyphenyl)chroman-4-one	PTGS1
Gancao	MOL004941	(2R)-7-hydroxy-2-(4-hydroxyphenyl)chroman-4-one	ESR1
Gancao	MOL004941	(2R)-7-hydroxy-2-(4-hydroxyphenyl)chroman-4-one	PTGS2
Gancao	MOL004941	(2R)-7-hydroxy-2-(4-hydroxyphenyl)chroman-4-one	RXRA
Gancao	MOL004941	(2R)-7-hydroxy-2-(4-hydroxyphenyl)chroman-4-one	ADRB2
Gancao	MOL004941	(2R)-7-hydroxy-2-(4-hydroxyphenyl)chroman-4-one	HSP90AB1
Gancao	MOL004941	(2R)-7-hydroxy-2-(4-hydroxyphenyl)chroman-4-one	DPEP1
Gancao	MOL004941	(2R)-7-hydroxy-2-(4-hydroxyphenyl)chroman-4-one	MAOB
Gancao	MOL004941	(2R)-7-hydroxy-2-(4-hydroxyphenyl)chroman-4-one	PKIA
Gancao	MOL004941	(2R)-7-hydroxy-2-(4-hydroxyphenyl)chroman-4-one	CAMKMT
Gancao	MOL004941	(2R)-7-hydroxy-2-(4-hydroxyphenyl)chroman-4-one	GABRA1
Gancao	MOL004941	(2R)-7-hydroxy-2-(4-hydroxyphenyl)chroman-4-one	SLC6A4
Gancao	MOL004945	(2S)-7-hydroxy-2-(4-hydroxyphenyl)-8-(3-methylbut-2-enyl)chroman-4-one	NOS2
Gancao	MOL004945	(2S)-7-hydroxy-2-(4-hydroxyphenyl)-8-(3-methylbut-2-enyl)chroman-4-one	PTGS1
Gancao	MOL004945	(2S)-7-hydroxy-2-(4-hydroxyphenyl)-8-(3-methylbut-2-enyl)chroman-4-one	ESR1
Gancao	MOL004945	(2S)-7-hydroxy-2-(4-hydroxyphenyl)-8-(3-methylbut-2-enyl)chroman-4-one	SCN5A
Gancao	MOL004945	(2S)-7-hydroxy-2-(4-hydroxyphenyl)-8-(3-methylbut-2-enyl)chroman-4-one	PTGS2
Gancao	MOL004945	(2S)-7-hydroxy-2-(4-hydroxyphenyl)-8-(3-methylbut-2-enyl)chroman-4-one	ADRA1B
Gancao	MOL004945	(2S)-7-hydroxy-2-(4-hydroxyphenyl)-8-(3-methylbut-2-enyl)chroman-4-one	ADRB2
Gancao	MOL004945	(2S)-7-hydroxy-2-(4-hydroxyphenyl)-8-(3-methylbut-2-enyl)chroman-4-one	ESR2
Gancao	MOL004945	(2S)-7-hydroxy-2-(4-hydroxyphenyl)-8-(3-methylbut-2-enyl)chroman-4-one	HSP90AB1
Gancao	MOL004945	(2S)-7-hydroxy-2-(4-hydroxyphenyl)-8-(3-methylbut-2-enyl)chroman-4-one	CAMKMT

Gancao	MOL004948	Isoglycyrol	NOS2
Gancao	MOL004948	Isoglycyrol	ESR1
Gancao	MOL004948	Isoglycyrol	AR
Gancao	MOL004948	Isoglycyrol	PTGS2
Gancao	MOL004948	Isoglycyrol	DPP4
Gancao	MOL004948	Isoglycyrol	GSK3B
Gancao	MOL004949	Isolicoflavonol	NOS2
Gancao	MOL004949	Isolicoflavonol	ESR1
Gancao	MOL004949	Isolicoflavonol	AR
Gancao	MOL004949	Isolicoflavonol	PPARG
Gancao	MOL004949	Isolicoflavonol	PTGS2
Gancao	MOL004949	Isolicoflavonol	GSK3B
Gancao	MOL004949	Isolicoflavonol	HSP90AB1
Gancao	MOL004949	Isolicoflavonol	CDK2
Gancao	MOL004949	Isolicoflavonol	PRSS1
Gancao	MOL004949	Isolicoflavonol	CCNA2
Gancao	MOL004949	Isolicoflavonol	NCOA2
Gancao	MOL004949	Isolicoflavonol	CAMKMT
Gancao	MOL004957	HMO	NOS2
Gancao	MOL004957	HMO	PTGS1
Gancao	MOL004957	HMO	CHRM1
Gancao	MOL004957	HMO	ESR1

Gancao	MOL004957	HMO	AR
Gancao	MOL004957	HMO	SCN5A
Gancao	MOL004957	HMO	PPARG
Gancao	MOL004957	HMO	PTGS2
Gancao	MOL004957	HMO	RXRA
Gancao	MOL004957	HMO	SLC6A3
Gancao	MOL004957	HMO	ADRB2
Gancao	MOL004957	HMO	SLC6A4
Gancao	MOL004957	HMO	ESR2
Gancao	MOL004957	HMO	DPP4
Gancao	MOL004957	HMO	MAPK14
Gancao	MOL004957	HMO	GSK3B
Gancao	MOL004957	HMO	CDK2
Gancao	MOL004957	HMO	MAOB
Gancao	MOL004957	HMO	CHEK1
Gancao	MOL004957	HMO	IGHG1
Gancao	MOL004957	HMO	PRSS1
Gancao	MOL004957	HMO	CCNA2
Gancao	MOL004957	HMO	PKIA
Gancao	MOL004957	HMO	CAMKMT
Gancao	MOL004959	1-Methoxyphaseollidin	NOS2
Gancao	MOL004959	1-Methoxyphaseollidin	PTGS1

Gancao	MOL0049<br>59	1-Methoxyphaseollidin	KCNH2
Gancao	MOL0049<br>59	1-Methoxyphaseollidin	ESR1
Gancao	MOL0049<br>59	1-Methoxyphaseollidin	AR
Gancao	MOL0049<br>59	1-Methoxyphaseollidin	SCN5A
Gancao	MOL0049<br>59	1-Methoxyphaseollidin	PPARG
Gancao	MOL0049<br>59	1-Methoxyphaseollidin	PTGS2
Gancao	MOL0049<br>59	1-Methoxyphaseollidin	KDR
Gancao	MOL0049<br>59	1-Methoxyphaseollidin	RXRA
Gancao	MOL0049<br>59	1-Methoxyphaseollidin	ADRA1B
Gancao	MOL0049<br>59	1-Methoxyphaseollidin	ADRB2
Gancao	MOL0049<br>59	1-Methoxyphaseollidin	ADRA1D
Gancao	MOL0049<br>59	1-Methoxyphaseollidin	ESR2
Gancao	MOL0049<br>59	1-Methoxyphaseollidin	MAPK14
Gancao	MOL0049<br>59	1-Methoxyphaseollidin	GSK3B
Gancao	MOL0049<br>59	1-Methoxyphaseollidin	HSP90AB<br>1
Gancao	MOL0049<br>59	1-Methoxyphaseollidin	CDK2
Gancao	MOL0049<br>59	1-Methoxyphaseollidin	PRSS1
Gancao	MOL0049<br>59	1-Methoxyphaseollidin	CCNA2
Gancao	MOL0049<br>59	1-Methoxyphaseollidin	NCOA2
Gancao	MOL0049<br>59	1-Methoxyphaseollidin	NCOA1
Gancao	MOL0049<br>59	1-Methoxyphaseollidin	CAMKM<br>T
Gancao	MOL0049<br>61	Quercetin der.	NOS2

Gancao	MOL004961	Quercetin der.	PTGS1
Gancao	MOL004961	Quercetin der.	ESR1
Gancao	MOL004961	Quercetin der.	AR
Gancao	MOL004961	Quercetin der.	SCN5A
Gancao	MOL004961	Quercetin der.	PPARG
Gancao	MOL004961	Quercetin der.	PTGS2
Gancao	MOL004961	Quercetin der.	ESR2
Gancao	MOL004961	Quercetin der.	DPP4
Gancao	MOL004961	Quercetin der.	MAPK14
Gancao	MOL004961	Quercetin der.	GSK3B
Gancao	MOL004961	Quercetin der.	HSP90AB1
Gancao	MOL004961	Quercetin der.	CDK2
Gancao	MOL004961	Quercetin der.	PRSS1
Gancao	MOL004961	Quercetin der.	NCOA2
Gancao	MOL004961	Quercetin der.	CAMKMT
Gancao	MOL004966	3'-Hydroxy-4'-O-Methylglabridin	NOS2
Gancao	MOL004966	3'-Hydroxy-4'-O-Methylglabridin	PTGS1
Gancao	MOL004966	3'-Hydroxy-4'-O-Methylglabridin	KCNH2
Gancao	MOL004966	3'-Hydroxy-4'-O-Methylglabridin	ESR1
Gancao	MOL004966	3'-Hydroxy-4'-O-Methylglabridin	AR
Gancao	MOL004966	3'-Hydroxy-4'-O-Methylglabridin	SCN5A
Gancao	MOL004966	3'-Hydroxy-4'-O-Methylglabridin	PPARG

Gancao	MOL004966	3'-Hydroxy-4'-O-Methylglabridin	PTGS2
Gancao	MOL004966	3'-Hydroxy-4'-O-Methylglabridin	F7
Gancao	MOL004966	3'-Hydroxy-4'-O-Methylglabridin	KDR
Gancao	MOL004966	3'-Hydroxy-4'-O-Methylglabridin	ADRA1B
Gancao	MOL004966	3'-Hydroxy-4'-O-Methylglabridin	ADRB2
Gancao	MOL004966	3'-Hydroxy-4'-O-Methylglabridin	ESR2
Gancao	MOL004966	3'-Hydroxy-4'-O-Methylglabridin	MAPK14
Gancao	MOL004966	3'-Hydroxy-4'-O-Methylglabridin	GSK3B
Gancao	MOL004966	3'-Hydroxy-4'-O-Methylglabridin	HSP90AB1
Gancao	MOL004966	3'-Hydroxy-4'-O-Methylglabridin	CDK2
Gancao	MOL004966	3'-Hydroxy-4'-O-Methylglabridin	CHEK1
Gancao	MOL004966	3'-Hydroxy-4'-O-Methylglabridin	PRSS1
Gancao	MOL004966	3'-Hydroxy-4'-O-Methylglabridin	CCNA2
Gancao	MOL004966	3'-Hydroxy-4'-O-Methylglabridin	NCOA2
Gancao	MOL004966	3'-Hydroxy-4'-O-Methylglabridin	NCOA1
Gancao	MOL004966	3'-Hydroxy-4'-O-Methylglabridin	CAMKMT
Gancao	MOL000497	licochalcone a	NOS2
Gancao	MOL000497	licochalcone a	PTGS1
Gancao	MOL000497	licochalcone a	CHRM1
Gancao	MOL000497	licochalcone a	ESR1
Gancao	MOL000497	licochalcone a	AR
Gancao	MOL000497	licochalcone a	SCN5A

Gancao	MOL000497	licochalcone a	PPARG
Gancao	MOL000497	licochalcone a	PTGS2
Gancao	MOL000497	licochalcone a	CA2
Gancao	MOL000497	licochalcone a	ADRA1B
Gancao	MOL000497	licochalcone a	SLC6A3
Gancao	MOL000497	licochalcone a	ESR2
Gancao	MOL000497	licochalcone a	MAPK14
Gancao	MOL000497	licochalcone a	GSK3B
Gancao	MOL000497	licochalcone a	HSP90AB1
Gancao	MOL000497	licochalcone a	CDK2
Gancao	MOL000497	licochalcone a	CHEK1
Gancao	MOL000497	licochalcone a	CCNA2
Gancao	MOL000497	licochalcone a	CAMKMT
Gancao	MOL000497	licochalcone a	ADRB2
Gancao	MOL000497	licochalcone a	NCOA2
Gancao	MOL000497	licochalcone a	RELA
Gancao	MOL000497	licochalcone a	STAT3
Gancao	MOL000497	licochalcone a	CCND1
Gancao	MOL000497	licochalcone a	BCL2
Gancao	MOL000497	licochalcone a	EIF6
Gancao	MOL000497	licochalcone a	MAPK1
Gancao	MOL000497	licochalcone a	RB1

Gancao	MOL000497	licochalcone a	CDK4
Gancao	MOL000497	licochalcone a	FOSL2
Gancao	MOL004974	3'-Methoxyglabridin	NOS2
Gancao	MOL004974	3'-Methoxyglabridin	PTGS1
Gancao	MOL004974	3'-Methoxyglabridin	KCNH2
Gancao	MOL004974	3'-Methoxyglabridin	ESR1
Gancao	MOL004974	3'-Methoxyglabridin	AR
Gancao	MOL004974	3'-Methoxyglabridin	SCN5A
Gancao	MOL004974	3'-Methoxyglabridin	PPARG
Gancao	MOL004974	3'-Methoxyglabridin	PTGS2
Gancao	MOL004974	3'-Methoxyglabridin	F7
Gancao	MOL004974	3'-Methoxyglabridin	RXRA
Gancao	MOL004974	3'-Methoxyglabridin	ACHE
Gancao	MOL004974	3'-Methoxyglabridin	ADRA1B
Gancao	MOL004974	3'-Methoxyglabridin	ADRB2
Gancao	MOL004974	3'-Methoxyglabridin	ESR2
Gancao	MOL004974	3'-Methoxyglabridin	MAPK14
Gancao	MOL004974	3'-Methoxyglabridin	GSK3B
Gancao	MOL004974	3'-Methoxyglabridin	HSP90AB1
Gancao	MOL004974	3'-Methoxyglabridin	CDK2
Gancao	MOL004974	3'-Methoxyglabridin	CHEK1
Gancao	MOL004974	3'-Methoxyglabridin	PRSS1

Gancao	MOL004974	3'-Methoxyglabridin	CCNA2
Gancao	MOL004974	3'-Methoxyglabridin	NCOA2
Gancao	MOL004974	3'-Methoxyglabridin	NCOA1
Gancao	MOL004974	3'-Methoxyglabridin	CAMKMT
Gancao	MOL004978	2-[(3R)-8,8-dimethyl-3,4-dihydro-2H-pyrano[6,5-f]chromen-3-yl]-5-methoxyphenol	NOS2
Gancao	MOL004978	2-[(3R)-8,8-dimethyl-3,4-dihydro-2H-pyrano[6,5-f]chromen-3-yl]-5-methoxyphenol	PTGS1
Gancao	MOL004978	2-[(3R)-8,8-dimethyl-3,4-dihydro-2H-pyrano[6,5-f]chromen-3-yl]-5-methoxyphenol	CHRM3
Gancao	MOL004978	2-[(3R)-8,8-dimethyl-3,4-dihydro-2H-pyrano[6,5-f]chromen-3-yl]-5-methoxyphenol	KCNH2
Gancao	MOL004978	2-[(3R)-8,8-dimethyl-3,4-dihydro-2H-pyrano[6,5-f]chromen-3-yl]-5-methoxyphenol	CHRM1
Gancao	MOL004978	2-[(3R)-8,8-dimethyl-3,4-dihydro-2H-pyrano[6,5-f]chromen-3-yl]-5-methoxyphenol	ESR1
Gancao	MOL004978	2-[(3R)-8,8-dimethyl-3,4-dihydro-2H-pyrano[6,5-f]chromen-3-yl]-5-methoxyphenol	AR
Gancao	MOL004978	2-[(3R)-8,8-dimethyl-3,4-dihydro-2H-pyrano[6,5-f]chromen-3-yl]-5-methoxyphenol	SCN5A
Gancao	MOL004978	2-[(3R)-8,8-dimethyl-3,4-dihydro-2H-pyrano[6,5-f]chromen-3-yl]-5-methoxyphenol	PPARG
Gancao	MOL004978	2-[(3R)-8,8-dimethyl-3,4-dihydro-2H-pyrano[6,5-f]chromen-3-yl]-5-methoxyphenol	PTGS2
Gancao	MOL004978	2-[(3R)-8,8-dimethyl-3,4-dihydro-2H-pyrano[6,5-f]chromen-3-yl]-5-methoxyphenol	RXRA
Gancao	MOL004978	2-[(3R)-8,8-dimethyl-3,4-dihydro-2H-pyrano[6,5-f]chromen-3-yl]-5-methoxyphenol	ACHE
Gancao	MOL004978	2-[(3R)-8,8-dimethyl-3,4-dihydro-2H-pyrano[6,5-f]chromen-3-yl]-5-methoxyphenol	ADRA1B
Gancao	MOL004978	2-[(3R)-8,8-dimethyl-3,4-dihydro-2H-pyrano[6,5-f]chromen-3-yl]-5-methoxyphenol	SLC6A3
Gancao	MOL004978	2-[(3R)-8,8-dimethyl-3,4-dihydro-2H-pyrano[6,5-f]chromen-3-yl]-5-methoxyphenol	ADRB2
Gancao	MOL004978	2-[(3R)-8,8-dimethyl-3,4-dihydro-2H-pyrano[6,5-f]chromen-3-yl]-5-methoxyphenol	ESR2
Gancao	MOL004978	2-[(3R)-8,8-dimethyl-3,4-dihydro-2H-pyrano[6,5-f]chromen-3-yl]-5-methoxyphenol	MAPK14
Gancao	MOL004978	2-[(3R)-8,8-dimethyl-3,4-dihydro-2H-pyrano[6,5-f]chromen-3-yl]-5-methoxyphenol	GSK3B

Gancao	MOL004978	2-[(3R)-8,8-dimethyl-3,4-dihydro-2H-pyrano[6,5-f]chromen-3-yl]-5-methoxyphenol	CDK2
Gancao	MOL004978	2-[(3R)-8,8-dimethyl-3,4-dihydro-2H-pyrano[6,5-f]chromen-3-yl]-5-methoxyphenol	CHEK1
Gancao	MOL004978	2-[(3R)-8,8-dimethyl-3,4-dihydro-2H-pyrano[6,5-f]chromen-3-yl]-5-methoxyphenol	RXRB
Gancao	MOL004978	2-[(3R)-8,8-dimethyl-3,4-dihydro-2H-pyrano[6,5-f]chromen-3-yl]-5-methoxyphenol	PRSS1
Gancao	MOL004978	2-[(3R)-8,8-dimethyl-3,4-dihydro-2H-pyrano[6,5-f]chromen-3-yl]-5-methoxyphenol	CCNA2
Gancao	MOL004978	2-[(3R)-8,8-dimethyl-3,4-dihydro-2H-pyrano[6,5-f]chromen-3-yl]-5-methoxyphenol	NCOA2
Gancao	MOL004978	2-[(3R)-8,8-dimethyl-3,4-dihydro-2H-pyrano[6,5-f]chromen-3-yl]-5-methoxyphenol	NCOA1
Gancao	MOL004978	2-[(3R)-8,8-dimethyl-3,4-dihydro-2H-pyrano[6,5-f]chromen-3-yl]-5-methoxyphenol	CAMKMT
Gancao	MOL004980	Inflacoumarin A	ESR1
Gancao	MOL004980	Inflacoumarin A	AR
Gancao	MOL004980	Inflacoumarin A	PPARG
Gancao	MOL004980	Inflacoumarin A	PTGS2
Gancao	MOL004980	Inflacoumarin A	ADRB2
Gancao	MOL004980	Inflacoumarin A	DPP4
Gancao	MOL004980	Inflacoumarin A	HSP90AB1
Gancao	MOL004980	Inflacoumarin A	PRSS1
Gancao	MOL004980	Inflacoumarin A	NCOA2
Gancao	MOL004980	Inflacoumarin A	CAMKMT
Gancao	MOL004980	Inflacoumarin A	PTGS1
Gancao	MOL004980	Inflacoumarin A	SCN5A
Gancao	MOL004985	icos-5-enoic acid	NCOA2
Gancao	MOL004988	Kanzonol F	ESR1

Gancao	MOL004988	Kanzonol F	AR
Gancao	MOL004988	Kanzonol F	PTGS2
Gancao	MOL004988	Kanzonol F	ESR2
Gancao	MOL004988	Kanzonol F	NCOA2
Gancao	MOL004988	Kanzonol F	CAMKMT
Gancao	MOL004989	6-prenylated eriodictyol	NOS2
Gancao	MOL004989	6-prenylated eriodictyol	ESR1
Gancao	MOL004989	6-prenylated eriodictyol	SCN5A
Gancao	MOL004989	6-prenylated eriodictyol	PTGS2
Gancao	MOL004989	6-prenylated eriodictyol	F7
Gancao	MOL004989	6-prenylated eriodictyol	HSP90AB1
Gancao	MOL004989	6-prenylated eriodictyol	CAMKMT
Gancao	MOL004990	7,2',4'-trihydroxy－5-methoxy-3－arylcoumarin	NOS2
Gancao	MOL004990	7,2',4'-trihydroxy－5-methoxy-3－arylcoumarin	PTGS1
Gancao	MOL004990	7,2',4'-trihydroxy－5-methoxy-3－arylcoumarin	ESR1
Gancao	MOL004990	7,2',4'-trihydroxy－5-methoxy-3－arylcoumarin	AR
Gancao	MOL004990	7,2',4'-trihydroxy－5-methoxy-3－arylcoumarin	PPARG
Gancao	MOL004990	7,2',4'-trihydroxy－5-methoxy-3－arylcoumarin	PTGS2
Gancao	MOL004990	7,2',4'-trihydroxy－5-methoxy-3－arylcoumarin	ESR2
Gancao	MOL004990	7,2',4'-trihydroxy－5-methoxy-3－arylcoumarin	DPP4
Gancao	MOL004990	7,2',4'-trihydroxy－5-methoxy-3－arylcoumarin	MAPK14
Gancao	MOL004990	7,2',4'-trihydroxy－5-methoxy-3－arylcoumarin	GSK3B

Gancao	MOL004990	7,2',4'-trihydroxy－5-methoxy-3－arylcoumarin	HSP90AB1
Gancao	MOL004990	7,2',4'-trihydroxy－5-methoxy-3－arylcoumarin	CDK2
Gancao	MOL004990	7,2',4'-trihydroxy－5-methoxy-3－arylcoumarin	CHEK1
Gancao	MOL004991	7-Acetoxy-2-methylisoflavone	NOS2
Gancao	MOL004991	7-Acetoxy-2-methylisoflavone	PTGS1
Gancao	MOL004991	7-Acetoxy-2-methylisoflavone	ESR1
Gancao	MOL004991	7-Acetoxy-2-methylisoflavone	AR
Gancao	MOL004991	7-Acetoxy-2-methylisoflavone	SCN5A
Gancao	MOL004991	7-Acetoxy-2-methylisoflavone	PPARG
Gancao	MOL004991	7-Acetoxy-2-methylisoflavone	PTGS2
Gancao	MOL004991	7-Acetoxy-2-methylisoflavone	RXRA
Gancao	MOL004991	7-Acetoxy-2-methylisoflavone	ACHE
Gancao	MOL004991	7-Acetoxy-2-methylisoflavone	ADRA1B
Gancao	MOL004991	7-Acetoxy-2-methylisoflavone	ADRB2
Gancao	MOL004991	7-Acetoxy-2-methylisoflavone	ADRA1D
Gancao	MOL004991	7-Acetoxy-2-methylisoflavone	GABRA1
Gancao	MOL004991	7-Acetoxy-2-methylisoflavone	DPP4
Gancao	MOL004991	7-Acetoxy-2-methylisoflavone	MAPK14
Gancao	MOL004991	7-Acetoxy-2-methylisoflavone	GSK3B
Gancao	MOL004991	7-Acetoxy-2-methylisoflavone	HSP90AB1
Gancao	MOL004991	7-Acetoxy-2-methylisoflavone	CDK2
Gancao	MOL004991	7-Acetoxy-2-methylisoflavone	CHEK1

Gancao	MOL004991	7-Acetoxy-2-methylisoflavone	PRSS1
Gancao	MOL004991	7-Acetoxy-2-methylisoflavone	NCOA2
Gancao	MOL004991	7-Acetoxy-2-methylisoflavone	CAMKMT
Gancao	MOL004993	8-prenylated eriodictyol	ESR1
Gancao	MOL004993	8-prenylated eriodictyol	SCN5A
Gancao	MOL004993	8-prenylated eriodictyol	PTGS2
Gancao	MOL004993	8-prenylated eriodictyol	F7
Gancao	MOL004993	8-prenylated eriodictyol	HSP90AB1
Gancao	MOL004993	8-prenylated eriodictyol	NCOA1
Gancao	MOL004993	8-prenylated eriodictyol	CAMKMT
Gancao	MOL004996	gadelaidic acid	NCOA2
Gancao	MOL000500	Vestitol	NOS2
Gancao	MOL000500	Vestitol	PTGS1
Gancao	MOL000500	Vestitol	CHRM1
Gancao	MOL000500	Vestitol	ESR1
Gancao	MOL000500	Vestitol	AR
Gancao	MOL000500	Vestitol	SCN5A
Gancao	MOL000500	Vestitol	PPARG
Gancao	MOL000500	Vestitol	PTGS2
Gancao	MOL000500	Vestitol	CHRM4
Gancao	MOL000500	Vestitol	RXRA
Gancao	MOL000500	Vestitol	ADRA1A

Gancao	MOL000500	Vestitol	ADRA1B
Gancao	MOL000500	Vestitol	SLC6A3
Gancao	MOL000500	Vestitol	ADRB2
Gancao	MOL000500	Vestitol	SLC6A4
Gancao	MOL000500	Vestitol	ESR2
Gancao	MOL000500	Vestitol	DPP4
Gancao	MOL000500	Vestitol	MAPK14
Gancao	MOL000500	Vestitol	GSK3B
Gancao	MOL000500	Vestitol	HSP90AB1
Gancao	MOL000500	Vestitol	CDK2
Gancao	MOL000500	Vestitol	CHEK1
Gancao	MOL000500	Vestitol	PRSS1
Gancao	MOL000500	Vestitol	CCNA2
Gancao	MOL000500	Vestitol	PKIA
Gancao	MOL000500	Vestitol	CAMKMT
Gancao	MOL005000	Gancaonin G	NOS2
Gancao	MOL005000	Gancaonin G	ESR1
Gancao	MOL005000	Gancaonin G	AR
Gancao	MOL005000	Gancaonin G	PPARG
Gancao	MOL005000	Gancaonin G	PTGS2
Gancao	MOL005000	Gancaonin G	ESR2
Gancao	MOL005000	Gancaonin G	DPP4

Gancao	MOL005000	Gancaonin G	MAPK14
Gancao	MOL005000	Gancaonin G	GSK3B
Gancao	MOL005000	Gancaonin G	HSP90AB1
Gancao	MOL005000	Gancaonin G	CHEK1
Gancao	MOL005000	Gancaonin G	PRSS1
Gancao	MOL005000	Gancaonin G	CCNA2
Gancao	MOL005000	Gancaonin G	NCOA2
Gancao	MOL005000	Gancaonin G	CAMKMT
Gancao	MOL005001	Gancaonin H	ESR1
Gancao	MOL005001	Gancaonin H	AR
Gancao	MOL005001	Gancaonin H	PTGS2
Gancao	MOL005001	Gancaonin H	KDR
Gancao	MOL005001	Gancaonin H	HSP90AB1
Gancao	MOL005001	Gancaonin H	PRSS1
Gancao	MOL005001	Gancaonin H	CCNA2
Gancao	MOL005001	Gancaonin H	NCOA2
Gancao	MOL005001	Gancaonin H	CAMKMT
Gancao	MOL005003	Licoagrocarpin	NOS2
Gancao	MOL005003	Licoagrocarpin	PTGS1
Gancao	MOL005003	Licoagrocarpin	CHRM3
Gancao	MOL005003	Licoagrocarpin	KCNH2
Gancao	MOL005003	Licoagrocarpin	CHRM1

Gancao	MOL005003	Licoagrocarpin	ESR1
Gancao	MOL005003	Licoagrocarpin	AR
Gancao	MOL005003	Licoagrocarpin	SCN5A
Gancao	MOL005003	Licoagrocarpin	PPARG
Gancao	MOL005003	Licoagrocarpin	CHRM5
Gancao	MOL005003	Licoagrocarpin	PTGS2
Gancao	MOL005003	Licoagrocarpin	RXRA
Gancao	MOL005003	Licoagrocarpin	ACHE
Gancao	MOL005003	Licoagrocarpin	ADRA1B
Gancao	MOL005003	Licoagrocarpin	ADRB2
Gancao	MOL005003	Licoagrocarpin	ESR2
Gancao	MOL005003	Licoagrocarpin	MAPK14
Gancao	MOL005003	Licoagrocarpin	GSK3B
Gancao	MOL005003	Licoagrocarpin	HSP90AB1
Gancao	MOL005003	Licoagrocarpin	CDK2
Gancao	MOL005003	Licoagrocarpin	RXRB
Gancao	MOL005003	Licoagrocarpin	PRSS1
Gancao	MOL005003	Licoagrocarpin	CCNA2
Gancao	MOL005003	Licoagrocarpin	NCOA2
Gancao	MOL005003	Licoagrocarpin	CAMKMT
Gancao	MOL005007	Glyasperins M	NOS2
Gancao	MOL005007	Glyasperins M	PTGS1

Gancao	MOL005007	Glyasperins M	KCNH2
Gancao	MOL005007	Glyasperins M	ESR1
Gancao	MOL005007	Glyasperins M	AR
Gancao	MOL005007	Glyasperins M	SCN5A
Gancao	MOL005007	Glyasperins M	PPARG
Gancao	MOL005007	Glyasperins M	PTGS2
Gancao	MOL005007	Glyasperins M	F7
Gancao	MOL005007	Glyasperins M	KDR
Gancao	MOL005007	Glyasperins M	ACHE
Gancao	MOL005007	Glyasperins M	ESR2
Gancao	MOL005007	Glyasperins M	PPARD
Gancao	MOL005007	Glyasperins M	GSK3B
Gancao	MOL005007	Glyasperins M	HSP90AB1
Gancao	MOL005007	Glyasperins M	CDK2
Gancao	MOL005007	Glyasperins M	PRSS1
Gancao	MOL005007	Glyasperins M	CCNA2
Gancao	MOL005007	Glyasperins M	NCOA2
Gancao	MOL005007	Glyasperins M	NCOA1
Gancao	MOL005007	Glyasperins M	CAMKMT
Gancao	MOL005008	Glycyrrhiza flavonol A	NOS2
Gancao	MOL005008	Glycyrrhiza flavonol A	ESR1
Gancao	MOL005008	Glycyrrhiza flavonol A	AR

Gancao	MOL005008	Glycyrrhiza flavonol A	PTGS2
Gancao	MOL005008	Glycyrrhiza flavonol A	F7
Gancao	MOL005008	Glycyrrhiza flavonol A	ACHE
Gancao	MOL005008	Glycyrrhiza flavonol A	ESR2
Gancao	MOL005008	Glycyrrhiza flavonol A	DPP4
Gancao	MOL005008	Glycyrrhiza flavonol A	GSK3B
Gancao	MOL005008	Glycyrrhiza flavonol A	HSP90AB1
Gancao	MOL005008	Glycyrrhiza flavonol A	CDK2
Gancao	MOL005008	Glycyrrhiza flavonol A	PRSS1
Gancao	MOL005008	Glycyrrhiza flavonol A	CCNA2
Gancao	MOL005008	Glycyrrhiza flavonol A	CAMKMT
Gancao	MOL005012	Licoagroisoflavone	NOS2
Gancao	MOL005012	Licoagroisoflavone	ESR1
Gancao	MOL005012	Licoagroisoflavone	AR
Gancao	MOL005012	Licoagroisoflavone	SCN5A
Gancao	MOL005012	Licoagroisoflavone	PPARG
Gancao	MOL005012	Licoagroisoflavone	PTGS2
Gancao	MOL005012	Licoagroisoflavone	ESR2
Gancao	MOL005012	Licoagroisoflavone	DPP4
Gancao	MOL005012	Licoagroisoflavone	MAPK14
Gancao	MOL005012	Licoagroisoflavone	GSK3B
Gancao	MOL005012	Licoagroisoflavone	CDK2

Gancao	MOL005012	Licoagroisoflavone	CHEK1
Gancao	MOL005012	Licoagroisoflavone	PRSS1
Gancao	MOL005012	Licoagroisoflavone	CCNA2
Gancao	MOL005012	Licoagroisoflavone	CAMKMT
Gancao	MOL005016	Odoratin	NOS2
Gancao	MOL005016	Odoratin	PTGS1
Gancao	MOL005016	Odoratin	ESR1
Gancao	MOL005016	Odoratin	AR
Gancao	MOL005016	Odoratin	SCN5A
Gancao	MOL005016	Odoratin	PPARG
Gancao	MOL005016	Odoratin	PTGS2
Gancao	MOL005016	Odoratin	RXRA
Gancao	MOL005016	Odoratin	ESR2
Gancao	MOL005016	Odoratin	DPP4
Gancao	MOL005016	Odoratin	MAPK14
Gancao	MOL005016	Odoratin	GSK3B
Gancao	MOL005016	Odoratin	HSP90AB1
Gancao	MOL005016	Odoratin	CDK2
Gancao	MOL005016	Odoratin	CHEK1
Gancao	MOL005016	Odoratin	PRSS1
Gancao	MOL005016	Odoratin	CCNA2
Gancao	MOL005016	Odoratin	NCOA2

Gancao	MOL005016	Odoratin	CAMKMT
Gancao	MOL005017	Phaseol	ESR1
Gancao	MOL005017	Phaseol	AR
Gancao	MOL005017	Phaseol	PPARG
Gancao	MOL005017	Phaseol	PTGS2
Gancao	MOL005017	Phaseol	KDR
Gancao	MOL005017	Phaseol	MAPK14
Gancao	MOL005017	Phaseol	GSK3B
Gancao	MOL005017	Phaseol	HSP90AB1
Gancao	MOL005017	Phaseol	CDK2
Gancao	MOL005017	Phaseol	CHEK1
Gancao	MOL005017	Phaseol	CCNA2
Gancao	MOL005018	Xambioona	NOS2
Gancao	MOL005018	Xambioona	ESR1
Gancao	MOL005018	Xambioona	PTGS2
Gancao	MOL005018	Xambioona	ESR2
Gancao	MOL005018	Xambioona	NCOA2
Gancao	MOL005018	Xambioona	CAMKMT
Gancao	MOL005020	dehydroglyasperins C	NOS2
Gancao	MOL005020	dehydroglyasperins C	ESR1
Gancao	MOL005020	dehydroglyasperins C	AR
Gancao	MOL005020	dehydroglyasperins C	SCN5A

Gancao	MOL005020	dehydroglyasperins C	PPARG
Gancao	MOL005020	dehydroglyasperins C	PTGS2
Gancao	MOL005020	dehydroglyasperins C	ADRB2
Gancao	MOL005020	dehydroglyasperins C	ESR2
Gancao	MOL005020	dehydroglyasperins C	MAPK14
Gancao	MOL005020	dehydroglyasperins C	HSP90AB1
Gancao	MOL005020	dehydroglyasperins C	CDK2
Gancao	MOL005020	dehydroglyasperins C	CHEK1
Gancao	MOL005020	dehydroglyasperins C	PRSS1
Gancao	MOL005020	dehydroglyasperins C	CCNA2
Gancao	MOL005020	dehydroglyasperins C	NCOA2
Gancao	MOL005020	dehydroglyasperins C	CAMKMT
Gancao	MOL000098	quercetin	PTGS1
Gancao	MOL000098	quercetin	AR
Gancao	MOL000098	quercetin	PPARG
Gancao	MOL000098	quercetin	PTGS2
Gancao	MOL000098	quercetin	HSP90AB1
Gancao	MOL000098	quercetin	NCOA2
Gancao	MOL000098	quercetin	DPP4
Gancao	MOL000098	quercetin	AKR1B1
Gancao	MOL000098	quercetin	PRSS1
Gancao	MOL000098	quercetin	KCNH2

Gancao	MOL000098	quercetin	SCN5A
Gancao	MOL000098	quercetin	ADRB2
Gancao	MOL000098	quercetin	MMP3
Gancao	MOL000098	quercetin	F7
Gancao	MOL000098	quercetin	RXRA
Gancao	MOL000098	quercetin	ACHE
Gancao	MOL000098	quercetin	GABRA1
Gancao	MOL000098	quercetin	MAOB
Gancao	MOL000098	quercetin	RELA
Gancao	MOL000098	quercetin	EGFR
Gancao	MOL000098	quercetin	AKT1
Gancao	MOL000098	quercetin	VEGFA
Gancao	MOL000098	quercetin	CCND1
Gancao	MOL000098	quercetin	BCL2
Gancao	MOL000098	quercetin	BCL2L1
Gancao	MOL000098	quercetin	FOS
Gancao	MOL000098	quercetin	CDKN1A
Gancao	MOL000098	quercetin	EIF6
Gancao	MOL000098	quercetin	BAX
Gancao	MOL000098	quercetin	CASP9
Gancao	MOL000098	quercetin	PLAU
Gancao	MOL000098	quercetin	MMP2

Gancao	MOL000098	quercetin	MMP9
Gancao	MOL000098	quercetin	MAPK1
Gancao	MOL000098	quercetin	IL10RA
Gancao	MOL000098	quercetin	EGF
Gancao	MOL000098	quercetin	RB1
Gancao	MOL000098	quercetin	TNFAIP6
Gancao	MOL000098	quercetin	JUN
Gancao	MOL000098	quercetin	IL6R
Gancao	MOL000098	quercetin	AHSA1
Gancao	MOL000098	quercetin	CASP3
Gancao	MOL000098	quercetin	TP53
Gancao	MOL000098	quercetin	ELK1
Gancao	MOL000098	quercetin	NFKBIA
Gancao	MOL000098	quercetin	POR
Gancao	MOL000098	quercetin	ODC1
Gancao	MOL000098	quercetin	CASP8
Gancao	MOL000098	quercetin	TOP1
Gancao	MOL000098	quercetin	RAF1
Gancao	MOL000098	quercetin	SOD1
Gancao	MOL000098	quercetin	PRKCA
Gancao	MOL000098	quercetin	MMP1
Gancao	MOL000098	quercetin	HIF1A

Gancao	MOL000098	quercetin	STAT1
Gancao	MOL000098	quercetin	RUNX1T1
Gancao	MOL000098	quercetin	CDK1
Gancao	MOL000098	quercetin	HSPA5
Gancao	MOL000098	quercetin	ERBB2
Gancao	MOL000098	quercetin	PPARG
Gancao	MOL000098	quercetin	ACACA
Gancao	MOL000098	quercetin	HMOX1
Gancao	MOL000098	quercetin	CYP3A4
Gancao	MOL000098	quercetin	CYP1A2
Gancao	MOL000098	quercetin	CAV1
Gancao	MOL000098	quercetin	MYC
Gancao	MOL000098	quercetin	F3
Gancao	MOL000098	quercetin	GJA1
Gancao	MOL000098	quercetin	CYP1A1
Gancao	MOL000098	quercetin	ICAM1
Gancao	MOL000098	quercetin	IL1B
Gancao	MOL000098	quercetin	CCL2
Gancao	MOL000098	quercetin	SELE
Gancao	MOL000098	quercetin	VCAM1
Gancao	MOL000098	quercetin	PTGER3
Gancao	MOL000098	quercetin	CXCL8

Gancao	MOL000098	quercetin	PRKCB
Gancao	MOL000098	quercetin	BIRC5
Gancao	MOL000098	quercetin	DUOX2
Gancao	MOL000098	quercetin	NOS3
Gancao	MOL000098	quercetin	HSPB1
Gancao	MOL000098	quercetin	SULT1E1
Gancao	MOL000098	quercetin	IL2RA
Gancao	MOL000098	quercetin	NR1I2
Gancao	MOL000098	quercetin	CYP1B1
Gancao	MOL000098	quercetin	CCNB1
Gancao	MOL000098	quercetin	PLAT
Gancao	MOL000098	quercetin	THBD
Gancao	MOL000098	quercetin	SERPINE1
Gancao	MOL000098	quercetin	COL1A1
Gancao	MOL000098	quercetin	IFNG
Gancao	MOL000098	quercetin	ALOX5
Gancao	MOL000098	quercetin	IL1A
Gancao	MOL000098	quercetin	MPO
Gancao	MOL000098	quercetin	TOP2A
Gancao	MOL000098	quercetin	NCF1
Gancao	MOL000098	quercetin	ABCG2
Gancao	MOL000098	quercetin	HAS2

Gancao	MOL000098	quercetin	GSTP1
Gancao	MOL000098	quercetin	NFE2L2
Gancao	MOL000098	quercetin	NQO1
Gancao	MOL000098	quercetin	PARP1
Gancao	MOL000098	quercetin	AHR
Gancao	MOL000098	quercetin	PSMD3
Gancao	MOL000098	quercetin	SLC2A4
Gancao	MOL000098	quercetin	COL3A1
Gancao	MOL000098	quercetin	CXCL11
Gancao	MOL000098	quercetin	CXCL2
Gancao	MOL000098	quercetin	DCAF5
Gancao	MOL000098	quercetin	NR1I3
Gancao	MOL000098	quercetin	CHEK2
Gancao	MOL000098	quercetin	INSRR
Gancao	MOL000098	quercetin	CLDN4
Gancao	MOL000098	quercetin	PPARA
Gancao	MOL000098	quercetin	PPARD
Gancao	MOL000098	quercetin	HSF1
Gancao	MOL000098	quercetin	CXCL10
Gancao	MOL000098	quercetin	CHUK
Gancao	MOL000098	quercetin	SPP1
Gancao	MOL000098	quercetin	RUNX2

Gancao	MOL000098	quercetin	RASSF1
Gancao	MOL000098	quercetin	E2F1
Gancao	MOL000098	quercetin	E2F2
Gancao	MOL000098	quercetin	ACP3
Gancao	MOL000098	quercetin	CTSD
Gancao	MOL000098	quercetin	IGFBP3
Gancao	MOL000098	quercetin	IGF2
Gancao	MOL000098	quercetin	CD40LG
Gancao	MOL000098	quercetin	IRF1
Gancao	MOL000098	quercetin	ERBB3
Gancao	MOL000098	quercetin	PON1
Gancao	MOL000098	quercetin	DIO1
Gancao	MOL000098	quercetin	PCOLCE
Gancao	MOL000098	quercetin	NPEPPS
Gancao	MOL000098	quercetin	HK2
Gancao	MOL000098	quercetin	RASA1
Gancao	MOL000098	quercetin	GSTM1
Gancao	MOL000098	quercetin	GSTM2
Guizhi	MOL001736	(-)-taxifolin	PTGS1
Guizhi	MOL001736	(-)-taxifolin	PTGS2
Guizhi	MOL001736	(-)-taxifolin	HSP90AB1
Guizhi	MOL000358	beta-sitosterol	PGR

Guizhi	MOL000358	beta-sitosterol	NCOA2
Guizhi	MOL000358	beta-sitosterol	PTGS1
Guizhi	MOL000358	beta-sitosterol	PTGS2
Guizhi	MOL000358	beta-sitosterol	HSP90AB1
Guizhi	MOL000358	beta-sitosterol	KCNH2
Guizhi	MOL000358	beta-sitosterol	DRD1
Guizhi	MOL000358	beta-sitosterol	CHRM3
Guizhi	MOL000358	beta-sitosterol	CHRM1
Guizhi	MOL000358	beta-sitosterol	SCN5A
Guizhi	MOL000358	beta-sitosterol	CHRM4
Guizhi	MOL000358	beta-sitosterol	ADRA1A
Guizhi	MOL000358	beta-sitosterol	CHRM2
Guizhi	MOL000358	beta-sitosterol	ADRA1B
Guizhi	MOL000358	beta-sitosterol	ADRB2
Guizhi	MOL000358	beta-sitosterol	CHRNA2
Guizhi	MOL000358	beta-sitosterol	SLC6A4
Guizhi	MOL000358	beta-sitosterol	OPRM1
Guizhi	MOL000358	beta-sitosterol	GABRA1
Guizhi	MOL000358	beta-sitosterol	BCL2
Guizhi	MOL000358	beta-sitosterol	BAX
Guizhi	MOL000358	beta-sitosterol	CASP9
Guizhi	MOL000358	beta-sitosterol	JUN

Guizhi	MOL000358	beta-sitosterol	CASP3
Guizhi	MOL000358	beta-sitosterol	CASP8
Guizhi	MOL000358	beta-sitosterol	PRKCA
Guizhi	MOL000358	beta-sitosterol	PON1
Guizhi	MOL000358	beta-sitosterol	MAP2
Guizhi	MOL000359	sitosterol	PGR
Guizhi	MOL000359	sitosterol	NCOA2
Guizhi	MOL000359	sitosterol	NR3C2
Guizhi	MOL000492	(+)-catechin	PTGS1
Guizhi	MOL000492	(+)-catechin	ESR1
Guizhi	MOL000492	(+)-catechin	PTGS2
Guizhi	MOL000492	(+)-catechin	HSP90AB1
Guizhi	MOL000492	(+)-catechin	DPEP1
Guizhi	MOL000492	(+)-catechin	NCOA2
Guizhi	MOL000492	(+)-catechin	CAMKMT
Guizhi	MOL000492	(+)-catechin	RXRA
Guizhi	MOL000492	(+)-catechin	CAT
Guizhi	MOL000492	(+)-catechin	HAS2
Guizhi	MOL000073	ent-Epicatechin	PTGS1
Guizhi	MOL000073	ent-Epicatechin	ESR1
Guizhi	MOL000073	ent-Epicatechin	PTGS2
Guizhi	MOL000073	ent-Epicatechin	HSP90AB1

Guizhi	MOL000073	ent-Epicatechin	DPEP1
Guizhi	MOL004576	taxifolin	PTGS1
Guizhi	MOL004576	taxifolin	PTGS2
Guizhi	MOL004576	taxifolin	HSP90AB1
Guizhi	MOL004576	taxifolin	RXRA
Guizhi	MOL004576	taxifolin	AKR1B1
Guizhi	MOL004576	taxifolin	RELA
Guizhi	MOL004576	taxifolin	ICAM1
Guizhi	MOL004576	taxifolin	DGAT2
Guizhi	MOL004576	taxifolin	MTTP
Guizhi	MOL004576	taxifolin	APOB
