# Supplementary material for: Exploring the molecular mechanism of Ling-Gui-Zhu-Gan decoction for the treatment of type 2 diabetes mellitus based on network pharmacology and molecular docking: A review
Source: Medicine (Baltimore). 2023 Mar 24;102(12):e33210. doi: 10.1097/MD.0000000000033210 (PMC10036033; doi:10.1097/MD.0000000000033210)
Supplement: Supplementary file 2 [file medi-102-e33210-s002.pdf]

Supplementary file 2-Information on active ingredient-target network construction

Exploring the mechanism of Ling-Gui-Zhu-Gan-decoction for the treatment of type 2 diabetes based on network pharmacology and molecular docking

Feng Long, MS[1],Zhe Zhang, MS[1],ChunXiu Luo, MS[1],Xiao Lei, PHD[1]*，Jinlian Guo, MS[1],Lin An, MS[1]

1.Department of traditional Chinese Medicine, affiliated Hospital of North Sichuan Medical College, Nanchong, Sichuan 637000.

*** Correspondence**: Xiao Lei，2424932797@qq.com

Table S1: Basic information of network construction

ID	Symbol
MOL000022	PTGS2
MOL000033	PGR
MOL000049	CHRM3
MOL000049	CHRM1
MOL000049	AR
MOL000049	SCN5A
MOL000049	PTGS2
MOL000049	RXRA
MOL000049	ACHE
MOL000049	ADRA1A
MOL000049	CHRM2
MOL000049	ADRB2

MOL000049	OPRM1
MOL000049	GABRA1
MOL000049	DPP4
MOL000072	PTGS2
MOL000072	GABRA1
MOL000072	NCOA2
MOL000072	NCOA1
MOL000273	NR3C2
MOL000273	NCOA2
MOL000275	NR3C2
MOL000279	NR3C2
MOL000282	PGR
MOL000283	PGR
MOL000296	PGR
MOL000296	NCOA2
MOL000296	CHRM3
MOL000296	CHRM1
MOL000296	CHRM2
MOL000296	ADRA1B
MOL000296	GABRA1
MOL000296	GRIA2
MOL000296	IGHG1
MOL000296	ADH1B
MOL000296	ADH1C
MOL000296	LYG1
MOL000296	PTGS1
MOL000296	SCN5A
MOL000296	PTGS2
MOL000296	RXRA
MOL000296	SLC6A2
MOL001484	PTGS1
MOL001484	CHRM3
MOL001484	SCN5A
MOL001484	PTGS2
MOL001484	HTR3A
MOL001484	RXRA
MOL001484	ADRA1B
MOL001484	ADRA1D
MOL001484	IGHG1
MOL001484	PRSS1
MOL001484	CAMKMT
MOL001484	CHRM1
MOL001484	ADRB2

MOL001484	OPRM1
MOL001484	HSP90AB1
MOL001792	PTGS1
MOL001792	ESR1
MOL001792	PTGS2
MOL001792	RXRA
MOL001792	ADRB2
MOL001792	HSP90AB1
MOL001792	DPEP1
MOL001792	MAOB
MOL001792	SLC6A4
MOL001792	PKIA
MOL000211	PGR
MOL002311	NOS2
MOL002311	ESR1
MOL002311	PPARG
MOL002311	PTGS2
MOL002311	KDR
MOL002311	MAPK14
MOL002311	GSK3B
MOL002311	CHEK1
MOL002311	CCNA2
MOL000239	NOS2
MOL000239	PTGS1
MOL000239	AR
MOL000239	SCN5A
MOL000239	PTGS2
MOL000239	ESR2
MOL000239	DPP4
MOL000239	HSP90AB1
MOL000239	CDK2
MOL000239	CHEK1
MOL000239	PRSS1
MOL000239	NCOA2
MOL000239	CAMKMT
MOL002565	NOS2
MOL002565	PTGS1
MOL002565	DRD1
MOL002565	CHRM3
MOL002565	CHRM1
MOL002565	ESR1
MOL002565	SCN5A
MOL002565	CHRM5

MOL002565	PTGS2
MOL002565	CHRM4
MOL002565	RXRA
MOL002565	ADRA1A
MOL002565	CHRM2
MOL002565	ADRA1B
MOL002565	SLC6A3
MOL002565	ADRB2
MOL002565	SLC6A4
MOL002565	OPRM1
MOL002565	ESR2
MOL002565	DPP4
MOL002565	MAPK10
MOL002565	HSP90AB1
MOL002565	CDK2
MOL002565	PRSS1
MOL002565	CCNA2
MOL002565	CAMKMT
MOL002565	OPRD1
MOL002565	ADRA1D
MOL000354	NOS2
MOL000354	PTGS1
MOL000354	ESR1
MOL000354	AR
MOL000354	PPARG
MOL000354	PTGS2
MOL000354	ESR2
MOL000354	DPP4
MOL000354	MAPK14
MOL000354	GSK3B
MOL000354	HSP90AB1
MOL000354	CDK2
MOL000354	PRSS1
MOL000354	CCNA2
MOL000354	NCOA2
MOL000354	CAMKMT
MOL000354	PYGM
MOL000354	PPARD
MOL000354	CHEK1
MOL000354	AKR1B1
MOL000354	NCOA1
MOL000354	F7
MOL000354	ACHE

MOL000354	GABRA1
MOL000354	MAOB
MOL000354	GRIA2
MOL000354	RELA
MOL000354	NCF1
MOL000354	OLR1
MOL000359	PGR
MOL000359	NCOA2
MOL000359	NR3C2
MOL003656	NOS2
MOL003656	ESR1
MOL003656	AR
MOL003656	SCN5A
MOL003656	PPARG
MOL003656	PTGS2
MOL003656	ESR2
MOL003656	DPP4
MOL003656	MAPK14
MOL003656	GSK3B
MOL003656	HSP90AB1
MOL003656	CDK2
MOL003656	CHEK1
MOL003656	PRSS1
MOL003656	CCNA2
MOL003656	NCOA2
MOL003656	CAMKMT
MOL003896	NOS2
MOL003896	PTGS1
MOL003896	DRD1
MOL003896	CHRM3
MOL003896	CHRM1
MOL003896	ESR1
MOL003896	AR
MOL003896	ADRB1
MOL003896	SCN5A
MOL003896	PPARG
MOL003896	PTGS2
MOL003896	RXRA
MOL003896	ACHE
MOL003896	ADRA1B
MOL003896	SLC6A3
MOL003896	ADRB2
MOL003896	ADRA1D

MOL003896	SLC6A4
MOL003896	ESR2
MOL003896	GABRA1
MOL003896	DPP4
MOL003896	MAPK14
MOL003896	GSK3B
MOL003896	HSP90AB1
MOL003896	CDK2
MOL003896	LTA4H
MOL003896	MAOB
MOL003896	CHEK1
MOL003896	IGHG1
MOL003896	PRSS1
MOL003896	CCNA2
MOL003896	NCOA1
MOL003896	PKIA
MOL003896	CAMKMT
MOL003896	CHRM5
MOL003896	OPRM1
MOL003896	NCOA2
MOL000392	NOS2
MOL000392	PTGS1
MOL000392	CHRM1
MOL000392	ESR1
MOL000392	AR
MOL000392	PPARG
MOL000392	PTGS2
MOL000392	RXRA
MOL000392	ADRA1A
MOL000392	SLC6A3
MOL000392	ADRB2
MOL000392	SLC6A4
MOL000392	ESR2
MOL000392	DPP4
MOL000392	MAPK14
MOL000392	GSK3B
MOL000392	HSP90AB1
MOL000392	CDK2
MOL000392	MAOB
MOL000392	CHEK1
MOL000392	PRSS1
MOL000392	CCNA2
MOL000392	CAMKMT

MOL000392	PKIA
MOL000392	ACHE
MOL000392	DPEP1
MOL000392	JUN
MOL000392	PPARG
MOL000392	IL4
MOL000392	ATP5F1B
MOL000392	HSD3B2
MOL000392	HSD3B1
MOL000417	NOS2
MOL000417	PTGS1
MOL000417	ESR1
MOL000417	AR
MOL000417	PPARG
MOL000417	PTGS2
MOL000417	RXRA
MOL000417	ESR2
MOL000417	DPP4
MOL000417	MAPK14
MOL000417	GSK3B
MOL000417	HSP90AB1
MOL000417	CDK2
MOL000417	CHEK1
MOL000417	PRSS1
MOL000417	CCNA2
MOL000417	NCOA2
MOL000417	CAMKMT
MOL000417	ADRB2
MOL000422	NOS2
MOL000422	PTGS1
MOL000422	AR
MOL000422	PPARG
MOL000422	PTGS2
MOL000422	HSP90AB1
MOL000422	NCOA2
MOL000422	DPP4
MOL000422	PRSS1
MOL000422	PGR
MOL000422	CHRM1
MOL000422	ACHE
MOL000422	SLC6A2
MOL000422	CHRM2
MOL000422	ADRA1B

MOL000422	GABRA1
MOL000422	F7
MOL000422	CAMKMT
MOL000422	RELA
MOL000422	IKBKB
MOL000422	AKT1
MOL000422	BCL2
MOL000422	BAX
MOL000422	TNFAIP6
MOL000422	JUN
MOL000422	AHSA1
MOL000422	CASP3
MOL000422	MAPK8
MOL000422	MMP1
MOL000422	STAT1
MOL000422	CDK1
MOL000422	PPARG
MOL000422	HMOX1
MOL000422	CYP3A4
MOL000422	CYP1A2
MOL000422	CYP1A1
MOL000422	ICAM1
MOL000422	SELE
MOL000422	VCAM1
MOL000422	NR1I2
MOL000422	CYP1B1
MOL000422	ALOX5
MOL000422	HAS2
MOL000422	GSTP1
MOL000422	AHR
MOL000422	PSMD3
MOL000422	SLC2A4
MOL000422	NR1I3
MOL000422	INSRR
MOL000422	DIO1
MOL000422	PPP3CA
MOL000422	GSTM1
MOL000422	GSTM2
MOL000422	AKR1C3
MOL000422	SLPI
MOL004328	PTGS1
MOL004328	ESR1
MOL004328	PTGS2

MOL004328	HSP90AB1
MOL004328	DPEP1
MOL004328	RELA
MOL004328	AKT1
MOL004328	BCL2
MOL004328	MAPK3
MOL004328	MAPK1
MOL004328	CASP3
MOL004328	FASN
MOL004328	LDLR
MOL004328	BAD
MOL004328	SOD1
MOL004328	CAT
MOL004328	PPARG
MOL004328	MTTP
MOL004328	APOB
MOL004328	PLB1
MOL004328	HMGCR
MOL004328	CYP19A1
MOL004328	GSTP1
MOL004328	UGT1A1
MOL004328	PPARA
MOL004328	SREBF1
MOL004328	GSR
MOL004328	ABCC1
MOL004328	ADIPOR2
MOL004328	SOAT2
MOL004328	AKR1C1
MOL004328	GOT1
MOL004328	ABAT
MOL004328	CES1
MOL004328	SOAT1
MOL004805	NOS2
MOL004805	KCNH2
MOL004805	ESR1
MOL004805	AR
MOL004805	PPARG
MOL004805	PTGS2
MOL004805	ESR2
MOL004805	MAPK14
MOL004805	GSK3B
MOL004805	CAMKMT
MOL004806	NOS2

MOL004806	KCNH2
MOL004806	ESR1
MOL004806	SCN5A
MOL004806	PTGS2
MOL004806	ESR2
MOL004806	BACE2
MOL004806	CAMKMT
MOL004808	NOS2
MOL004808	ESR1
MOL004808	AR
MOL004808	PPARG
MOL004808	PTGS2
MOL004808	F7
MOL004808	KDR
MOL004808	ACHE
MOL004808	ESR2
MOL004808	DPP4
MOL004808	GSK3B
MOL004808	HSP90AB1
MOL004808	CDK2
MOL004808	PRSS1
MOL004808	CCNA2
MOL004808	NCOA2
MOL004808	CAMKMT
MOL004810	NOS2
MOL004810	PTGS1
MOL004810	ESR1
MOL004810	AR
MOL004810	SCN5A
MOL004810	PPARG
MOL004810	PTGS2
MOL004810	ESR2
MOL004810	MAPK14
MOL004810	GSK3B
MOL004810	HSP90AB1
MOL004810	CDK2
MOL004810	PRSS1
MOL004810	CCNA2
MOL004810	CAMKMT
MOL004811	NOS2
MOL004811	KCNH2
MOL004811	ESR1
MOL004811	AR

MOL004811	SCN5A
MOL004811	PPARG
MOL004811	PTGS2
MOL004811	RXRA
MOL004811	ACHE
MOL004811	ESR2
MOL004811	DPP4
MOL004811	MAPK14
MOL004811	GSK3B
MOL004811	HSP90AB1
MOL004811	CDK2
MOL004811	CHEK1
MOL004811	PRSS1
MOL004811	CCNA2
MOL004811	NCOA2
MOL004811	CAMKMT
MOL004814	NOS2
MOL004814	ESR1
MOL004814	AR
MOL004814	PTGS2
MOL004814	ESR2
MOL004814	MAPK14
MOL004814	GSK3B
MOL004814	HSP90AB1
MOL004814	CDK2
MOL004814	CHEK1
MOL004814	CCNA2
MOL004815	NOS2
MOL004815	PTGS1
MOL004815	ESR1
MOL004815	AR
MOL004815	SCN5A
MOL004815	PPARG
MOL004815	PTGS2
MOL004815	CA2
MOL004815	RXRA
MOL004815	ADRA1B
MOL004815	ESR2
MOL004815	MAPK14
MOL004815	GSK3B
MOL004815	CDK2
MOL004815	CHEK1
MOL004815	CCNA2

MOL004815	NCOA2
MOL004815	CAMKMT
MOL004820	NOS2
MOL004820	PTGS1
MOL004820	ESR1
MOL004820	AR
MOL004820	SCN5A
MOL004820	PPARG
MOL004820	PTGS2
MOL004820	RXRA
MOL004820	ESR2
MOL004820	MAPK14
MOL004820	GSK3B
MOL004820	CDK2
MOL004820	CHEK1
MOL004820	PRSS1
MOL004820	CCNA2
MOL004820	NCOA2
MOL004820	NCOA1
MOL004820	CAMKMT
MOL004824	NOS2
MOL004824	ESR1
MOL004824	AR
MOL004824	PPARG
MOL004824	PTGS2
MOL004824	F7
MOL004824	KDR
MOL004824	ACHE
MOL004824	ESR2
MOL004824	DPP4
MOL004824	MAPK14
MOL004824	GSK3B
MOL004824	CDK2
MOL004824	CHEK1
MOL004824	PRSS1
MOL004824	CCNA2
MOL004824	CAMKMT
MOL004827	NOS2
MOL004827	ESR1
MOL004827	AR
MOL004827	SCN5A
MOL004827	PPARG
MOL004827	PTGS2

MOL004827	F7
MOL004827	ACHE
MOL004827	GSK3B
MOL004827	HSP90AB1
MOL004827	CDK2
MOL004827	CHEK1
MOL004827	PRSS1
MOL004827	CAMKMT
MOL004828	NOS2
MOL004828	PTGS1
MOL004828	ESR1
MOL004828	AR
MOL004828	SCN5A
MOL004828	PPARG
MOL004828	PTGS2
MOL004828	F7
MOL004828	KDR
MOL004828	RXRA
MOL004828	DPP4
MOL004828	MAPK14
MOL004828	GSK3B
MOL004828	HSP90AB1
MOL004828	CDK2
MOL004828	CHEK1
MOL004828	IGHG1
MOL004828	PRSS1
MOL004828	CCNA2
MOL004828	CAMKMT
MOL004829	PTGS1
MOL004829	ESR1
MOL004829	SCN5A
MOL004829	PTGS2
MOL004829	F7
MOL004829	RXRA
MOL004829	ADRA1B
MOL004829	HSP90AB1
MOL004829	IGHG1
MOL004829	NCOA1
MOL004829	CAMKMT
MOL004833	NOS2
MOL004833	CHRM1
MOL004833	ESR1
MOL004833	AR

MOL004833	SCN5A
MOL004833	PPARG
MOL004833	PTGS2
MOL004833	RXRA
MOL004833	ACHE
MOL004833	ADRA1B
MOL004833	ADRB2
MOL004833	ESR2
MOL004833	MAPK14
MOL004833	GSK3B
MOL004833	CDK2
MOL004833	CHEK1
MOL004833	PRSS1
MOL004833	CCNA2
MOL004833	NCOA1
MOL004833	CAMKMT
MOL004835	NOS2
MOL004835	PTGS1
MOL004835	CHRM1
MOL004835	ESR1
MOL004835	AR
MOL004835	SCN5A
MOL004835	PPARG
MOL004835	PTGS2
MOL004835	CA2
MOL004835	ADRA1B
MOL004835	SLC6A3
MOL004835	ADRB2
MOL004835	SLC6A4
MOL004835	ESR2
MOL004835	MAPK14
MOL004835	GSK3B
MOL004835	HSP90AB1
MOL004835	CDK2
MOL004835	LTA4H
MOL004835	MAOB
MOL004835	CHEK1
MOL004835	CCNA2
MOL004835	NCOA1
MOL004835	PKIA
MOL004835	CAMKMT
MOL004838	NOS2
MOL004838	ESR1

MOL004838	PTGS2
MOL004838	RXRA
MOL004838	HSP90AB1
MOL004841	NOS2
MOL004841	PTGS1
MOL004841	ESR1
MOL004841	AR
MOL004841	PPARG
MOL004841	PTGS2
MOL004841	CA2
MOL004841	ADRB2
MOL004841	ESR2
MOL004841	MAPK14
MOL004841	GSK3B
MOL004841	HSP90AB1
MOL004841	CDK2
MOL004841	CHEK1
MOL004841	CCNA2
MOL004841	CAMKMT
MOL004848	NOS2
MOL004848	ESR1
MOL004848	AR
MOL004848	PPARG
MOL004848	PTGS2
MOL004848	KDR
MOL004848	ESR2
MOL004848	MAPK14
MOL004848	GSK3B
MOL004848	HSP90AB1
MOL004848	CDK2
MOL004848	IGHG1
MOL004848	CCNA2
MOL004848	NCOA2
MOL004848	CAMKMT
MOL004849	NOS2
MOL004849	KCNH2
MOL004849	ESR1
MOL004849	AR
MOL004849	PPARG
MOL004849	PTGS2
MOL004849	F7
MOL004849	KDR
MOL004849	ESR2

MOL004849	DPP4
MOL004849	MAPK14
MOL004849	GSK3B
MOL004849	HSP90AB1
MOL004849	CDK2
MOL004849	CHEK1
MOL004849	PRSS1
MOL004849	NCOA2
MOL004849	NCOA1
MOL004849	CAMKMT
MOL004855	NOS2
MOL004855	KCNH2
MOL004855	ESR1
MOL004855	AR
MOL004855	PPARG
MOL004855	PTGS2
MOL004855	KDR
MOL004855	CHEK1
MOL004855	PRSS1
MOL004855	NCOA2
MOL004855	CAMKMT
MOL004856	NOS2
MOL004856	ESR1
MOL004856	AR
MOL004856	SCN5A
MOL004856	PPARG
MOL004856	PTGS2
MOL004856	ACHE
MOL004856	ESR2
MOL004856	DPP4
MOL004856	GSK3B
MOL004856	HSP90AB1
MOL004856	CHEK1
MOL004856	PRSS1
MOL004856	CCNA2
MOL004856	NCOA2
MOL004856	CAMKMT
MOL004857	NOS2
MOL004857	ESR1
MOL004857	AR
MOL004857	PPARG
MOL004857	PTGS2
MOL004857	F7

MOL004857	KDR
MOL004857	ADRA1B
MOL004857	ADRB2
MOL004857	ESR2
MOL004857	DPP4
MOL004857	GSK3B
MOL004857	HSP90AB1
MOL004857	CHEK1
MOL004857	PRSS1
MOL004857	CCNA2
MOL004857	NCOA2
MOL004857	CAMKMT
MOL004863	NOS2
MOL004863	ESR1
MOL004863	AR
MOL004863	PPARG
MOL004863	PTGS2
MOL004863	MAPK14
MOL004863	GSK3B
MOL004863	HSP90AB1
MOL004863	CDK2
MOL004863	CHEK1
MOL004863	PRSS1
MOL004863	CCNA2
MOL004863	NCOA2
MOL004863	CAMKMT
MOL004864	NOS2
MOL004864	KCNH2
MOL004864	ESR1
MOL004864	AR
MOL004864	PPARG
MOL004864	PTGS2
MOL004864	ESR2
MOL004864	DPP4
MOL004864	MAPK14
MOL004864	GSK3B
MOL004864	HSP90AB1
MOL004864	CDK2
MOL004864	CHEK1
MOL004864	PRSS1
MOL004864	CCNA2
MOL004864	NCOA2
MOL004864	CAMKMT

MOL004866	AR
MOL004866	SCN5A
MOL004866	PPARG
MOL004866	PTGS2
MOL004866	F7
MOL004866	ADRB2
MOL004866	DPP4
MOL004866	HSP90AB1
MOL004866	CDK2
MOL004866	CHEK1
MOL004866	PRSS1
MOL004866	CCNA2
MOL004866	CAMKMT
MOL004879	NOS2
MOL004879	KCNH2
MOL004879	ESR1
MOL004879	AR
MOL004879	PPARG
MOL004879	PTGS2
MOL004879	KDR
MOL004879	ESR2
MOL004879	DPP4
MOL004879	CHEK1
MOL004879	PRSS1
MOL004879	NCOA2
MOL004879	CAMKMT
MOL004882	ESR1
MOL004882	AR
MOL004882	ESR2
MOL004882	GSK3B
MOL004882	HSP90AB1
MOL004882	CDK2
MOL004882	CCNA2
MOL004883	NOS2
MOL004883	ESR1
MOL004883	AR
MOL004883	PPARG
MOL004883	PTGS2
MOL004883	KDR
MOL004883	DPP4
MOL004883	MAPK14
MOL004883	HSP90AB1
MOL004883	CDK2

MOL004883	CHEK1
MOL004883	PRSS1
MOL004883	CCNA2
MOL004883	NCOA2
MOL004883	CAMKMT
MOL004884	NOS2
MOL004884	ESR1
MOL004884	AR
MOL004884	PPARG
MOL004884	PTGS2
MOL004884	ACHE
MOL004884	ESR2
MOL004884	GSK3B
MOL004884	CDK2
MOL004884	CHEK1
MOL004884	PRSS1
MOL004884	CCNA2
MOL004884	CAMKMT
MOL004885	NOS2
MOL004885	PTGS1
MOL004885	ESR1
MOL004885	AR
MOL004885	SCN5A
MOL004885	PPARG
MOL004885	PTGS2
MOL004885	F7
MOL004885	ACHE
MOL004885	ESR2
MOL004885	GSK3B
MOL004885	HSP90AB1
MOL004885	CDK2
MOL004885	PRSS1
MOL004885	CCNA2
MOL004885	NCOA1
MOL004885	CAMKMT
MOL004891	NOS2
MOL004891	PTGS1
MOL004891	CHRM3
MOL004891	KCNH2
MOL004891	CHRM1
MOL004891	ESR1
MOL004891	AR
MOL004891	SCN5A

MOL004891	PPARG
MOL004891	PTGS2
MOL004891	HTR3A
MOL004891	RXRA
MOL004891	OPRD1
MOL004891	ADRA1B
MOL004891	ADRB2
MOL004891	ADRA1D
MOL004891	OPRM1
MOL004891	ESR2
MOL004891	MAPK14
MOL004891	GSK3B
MOL004891	CDK2
MOL004891	RXRB
MOL004891	PRSS1
MOL004891	CCNA2
MOL004891	NCOA1
MOL004891	CAMKMT
MOL004898	ESR1
MOL004898	AR
MOL004898	PPARG
MOL004898	PTGS2
MOL004898	MAPK14
MOL004898	GSK3B
MOL004898	HSP90AB1
MOL004898	CDK2
MOL004898	CCNA2
MOL004898	NCOA2
MOL004898	CAMKMT
MOL004903	F7
MOL004903	CAMKMT
MOL004903	PTGS2
MOL004903	KDR
MOL004903	SOD1
MOL004904	NOS2
MOL004904	ESR1
MOL004904	AR
MOL004904	PPARG
MOL004904	PTGS2
MOL004904	F7
MOL004904	KDR
MOL004904	ACHE
MOL004904	CDK2

MOL004904	PRSS1
MOL004904	CCNA2
MOL004904	CAMKMT
MOL004907	NOS2
MOL004907	PTGS1
MOL004907	ESR1
MOL004907	AR
MOL004907	PPARG
MOL004907	PTGS2
MOL004907	ESR2
MOL004907	DPP4
MOL004907	MAPK14
MOL004907	GSK3B
MOL004907	HSP90AB1
MOL004907	CDK2
MOL004907	CHEK1
MOL004907	PRSS1
MOL004907	CCNA2
MOL004908	NOS2
MOL004908	CHRM1
MOL004908	ESR1
MOL004908	AR
MOL004908	SCN5A
MOL004908	PPARG
MOL004908	PTGS2
MOL004908	RXRA
MOL004908	ACHE
MOL004908	ADRA1B
MOL004908	ADRB2
MOL004908	ESR2
MOL004908	MAPK14
MOL004908	GSK3B
MOL004908	CDK2
MOL004908	CHEK1
MOL004908	RXRB
MOL004908	IGHG1
MOL004908	PRSS1
MOL004908	CCNA2
MOL004908	NCOA2
MOL004908	NCOA1
MOL004908	CAMKMT
MOL004910	NOS2
MOL004910	PTGS1

MOL004910	ESR1
MOL004910	SCN5A
MOL004910	PTGS2
MOL004910	HSP90AB1
MOL004910	CAMKMT
MOL004911	NOS2
MOL004911	PTGS1
MOL004911	ESR1
MOL004911	AR
MOL004911	SCN5A
MOL004911	PPARG
MOL004911	PTGS2
MOL004911	RXRA
MOL004911	ADRB2
MOL004911	ESR2
MOL004911	MAPK14
MOL004911	GSK3B
MOL004911	HSP90AB1
MOL004911	CDK2
MOL004911	PRSS1
MOL004911	NCOA2
MOL004911	CAMKMT
MOL004912	NOS2
MOL004912	PTGS1
MOL004912	ESR1
MOL004912	AR
MOL004912	SCN5A
MOL004912	PPARG
MOL004912	PTGS2
MOL004912	RXRA
MOL004912	ACHE
MOL004912	ESR2
MOL004912	DPP4
MOL004912	MAPK14
MOL004912	GSK3B
MOL004912	CDK2
MOL004912	CHEK1
MOL004912	PRSS1
MOL004912	CCNA2
MOL004912	CAMKMT
MOL004913	ESR1
MOL004913	PPARG
MOL004913	ESR2

MOL004913	MAPK14
MOL004913	GSK3B
MOL004913	HSP90AB1
MOL004913	CDK2
MOL004913	CHEK1
MOL004913	CCNA2
MOL004914	ESR1
MOL004914	AR
MOL004914	PPARG
MOL004914	MAPK14
MOL004914	GSK3B
MOL004914	HSP90AB1
MOL004914	CDK2
MOL004914	CHEK1
MOL004915	NOS2
MOL004915	ESR1
MOL004915	AR
MOL004915	SCN5A
MOL004915	PPARG
MOL004915	PTGS2
MOL004915	ESR2
MOL004915	DPP4
MOL004915	MAPK14
MOL004915	GSK3B
MOL004915	HSP90AB1
MOL004915	CDK2
MOL004915	CHEK1
MOL004915	PRSS1
MOL004915	CCNA2
MOL004915	CAMKMT
MOL004924	PTGS2
MOL004924	ACHE
MOL004935	ESR1
MOL004935	PTGS2
MOL004935	KDR
MOL004935	HSP90AB1
MOL004935	CAMKMT
MOL004941	PTGS1
MOL004941	ESR1
MOL004941	PTGS2
MOL004941	RXRA
MOL004941	ADRB2
MOL004941	HSP90AB1

MOL004941	DPEP1
MOL004941	MAOB
MOL004941	PKIA
MOL004941	CAMKMT
MOL004941	GABRA1
MOL004941	SLC6A4
MOL004945	NOS2
MOL004945	PTGS1
MOL004945	ESR1
MOL004945	SCN5A
MOL004945	PTGS2
MOL004945	ADRA1B
MOL004945	ADRB2
MOL004945	ESR2
MOL004945	HSP90AB1
MOL004945	CAMKMT
MOL004948	NOS2
MOL004948	ESR1
MOL004948	AR
MOL004948	PTGS2
MOL004948	DPP4
MOL004948	GSK3B
MOL004949	NOS2
MOL004949	ESR1
MOL004949	AR
MOL004949	PPARG
MOL004949	PTGS2
MOL004949	GSK3B
MOL004949	HSP90AB1
MOL004949	CDK2
MOL004949	PRSS1
MOL004949	CCNA2
MOL004949	NCOA2
MOL004949	CAMKMT
MOL004957	NOS2
MOL004957	PTGS1
MOL004957	CHRM1
MOL004957	ESR1
MOL004957	AR
MOL004957	SCN5A
MOL004957	PPARG
MOL004957	PTGS2
MOL004957	RXRA

MOL004957	SLC6A3
MOL004957	ADRB2
MOL004957	SLC6A4
MOL004957	ESR2
MOL004957	DPP4
MOL004957	MAPK14
MOL004957	GSK3B
MOL004957	CDK2
MOL004957	MAOB
MOL004957	CHEK1
MOL004957	IGHG1
MOL004957	PRSS1
MOL004957	CCNA2
MOL004957	PKIA
MOL004957	CAMKMT
MOL004959	NOS2
MOL004959	PTGS1
MOL004959	KCNH2
MOL004959	ESR1
MOL004959	AR
MOL004959	SCN5A
MOL004959	PPARG
MOL004959	PTGS2
MOL004959	KDR
MOL004959	RXRA
MOL004959	ADRA1B
MOL004959	ADRB2
MOL004959	ADRA1D
MOL004959	ESR2
MOL004959	MAPK14
MOL004959	GSK3B
MOL004959	HSP90AB1
MOL004959	CDK2
MOL004959	PRSS1
MOL004959	CCNA2
MOL004959	NCOA2
MOL004959	NCOA1
MOL004959	CAMKMT
MOL004961	NOS2
MOL004961	PTGS1
MOL004961	ESR1
MOL004961	AR
MOL004961	SCN5A

MOL004961	PPARG
MOL004961	PTGS2
MOL004961	ESR2
MOL004961	DPP4
MOL004961	MAPK14
MOL004961	GSK3B
MOL004961	HSP90AB1
MOL004961	CDK2
MOL004961	PRSS1
MOL004961	NCOA2
MOL004961	CAMKMT
MOL004966	NOS2
MOL004966	PTGS1
MOL004966	KCNH2
MOL004966	ESR1
MOL004966	AR
MOL004966	SCN5A
MOL004966	PPARG
MOL004966	PTGS2
MOL004966	F7
MOL004966	KDR
MOL004966	ADRA1B
MOL004966	ADRB2
MOL004966	ESR2
MOL004966	MAPK14
MOL004966	GSK3B
MOL004966	HSP90AB1
MOL004966	CDK2
MOL004966	CHEK1
MOL004966	PRSS1
MOL004966	CCNA2
MOL004966	NCOA2
MOL004966	NCOA1
MOL004966	CAMKMT
MOL000497	NOS2
MOL000497	PTGS1
MOL000497	CHRM1
MOL000497	ESR1
MOL000497	AR
MOL000497	SCN5A
MOL000497	PPARG
MOL000497	PTGS2
MOL000497	CA2

MOL000497	ADRA1B
MOL000497	SLC6A3
MOL000497	ESR2
MOL000497	MAPK14
MOL000497	GSK3B
MOL000497	HSP90AB1
MOL000497	CDK2
MOL000497	CHEK1
MOL000497	CCNA2
MOL000497	CAMKMT
MOL000497	ADRB2
MOL000497	NCOA2
MOL000497	RELA
MOL000497	STAT3
MOL000497	CCND1
MOL000497	BCL2
MOL000497	EIF6
MOL000497	MAPK1
MOL000497	RB1
MOL000497	CDK4
MOL000497	FOSL2
MOL004974	NOS2
MOL004974	PTGS1
MOL004974	KCNH2
MOL004974	ESR1
MOL004974	AR
MOL004974	SCN5A
MOL004974	PPARG
MOL004974	PTGS2
MOL004974	F7
MOL004974	RXRA
MOL004974	ACHE
MOL004974	ADRA1B
MOL004974	ADRB2
MOL004974	ESR2
MOL004974	MAPK14
MOL004974	GSK3B
MOL004974	HSP90AB1
MOL004974	CDK2
MOL004974	CHEK1
MOL004974	PRSS1
MOL004974	CCNA2
MOL004974	NCOA2

MOL004974	NCOA1
MOL004974	CAMKMT
MOL004978	NOS2
MOL004978	PTGS1
MOL004978	CHRM3
MOL004978	KCNH2
MOL004978	CHRM1
MOL004978	ESR1
MOL004978	AR
MOL004978	SCN5A
MOL004978	PPARG
MOL004978	PTGS2
MOL004978	RXRA
MOL004978	ACHE
MOL004978	ADRA1B
MOL004978	SLC6A3
MOL004978	ADRB2
MOL004978	ESR2
MOL004978	MAPK14
MOL004978	GSK3B
MOL004978	CDK2
MOL004978	CHEK1
MOL004978	RXRB
MOL004978	PRSS1
MOL004978	CCNA2
MOL004978	NCOA2
MOL004978	NCOA1
MOL004978	CAMKMT
MOL004980	ESR1
MOL004980	AR
MOL004980	PPARG
MOL004980	PTGS2
MOL004980	ADRB2
MOL004980	DPP4
MOL004980	HSP90AB1
MOL004980	PRSS1
MOL004980	NCOA2
MOL004980	CAMKMT
MOL004980	PTGS1
MOL004980	SCN5A
MOL004985	NCOA2
MOL004988	ESR1
MOL004988	AR

MOL004988	PTGS2
MOL004988	ESR2
MOL004988	NCOA2
MOL004988	CAMKMT
MOL004989	NOS2
MOL004989	ESR1
MOL004989	SCN5A
MOL004989	PTGS2
MOL004989	F7
MOL004989	HSP90AB1
MOL004989	CAMKMT
MOL004990	NOS2
MOL004990	PTGS1
MOL004990	ESR1
MOL004990	AR
MOL004990	PPARG
MOL004990	PTGS2
MOL004990	ESR2
MOL004990	DPP4
MOL004990	MAPK14
MOL004990	GSK3B
MOL004990	HSP90AB1
MOL004990	CDK2
MOL004990	CHEK1
MOL004991	NOS2
MOL004991	PTGS1
MOL004991	ESR1
MOL004991	AR
MOL004991	SCN5A
MOL004991	PPARG
MOL004991	PTGS2
MOL004991	RXRA
MOL004991	ACHE
MOL004991	ADRA1B
MOL004991	ADRB2
MOL004991	ADRA1D
MOL004991	GABRA1
MOL004991	DPP4
MOL004991	MAPK14
MOL004991	GSK3B
MOL004991	HSP90AB1
MOL004991	CDK2
MOL004991	CHEK1

MOL004991	PRSS1
MOL004991	NCOA2
MOL004991	CAMKMT
MOL004993	ESR1
MOL004993	SCN5A
MOL004993	PTGS2
MOL004993	F7
MOL004993	HSP90AB1
MOL004993	NCOA1
MOL004993	CAMKMT
MOL004996	NCOA2
MOL000500	NOS2
MOL000500	PTGS1
MOL000500	CHRM1
MOL000500	ESR1
MOL000500	AR
MOL000500	SCN5A
MOL000500	PPARG
MOL000500	PTGS2
MOL000500	CHRM4
MOL000500	RXRA
MOL000500	ADRA1A
MOL000500	ADRA1B
MOL000500	SLC6A3
MOL000500	ADRB2
MOL000500	SLC6A4
MOL000500	ESR2
MOL000500	DPP4
MOL000500	MAPK14
MOL000500	GSK3B
MOL000500	HSP90AB1
MOL000500	CDK2
MOL000500	CHEK1
MOL000500	PRSS1
MOL000500	CCNA2
MOL000500	PKIA
MOL000500	CAMKMT
MOL005000	NOS2
MOL005000	ESR1
MOL005000	AR
MOL005000	PPARG
MOL005000	PTGS2
MOL005000	ESR2

MOL005000	DPP4
MOL005000	MAPK14
MOL005000	GSK3B
MOL005000	HSP90AB1
MOL005000	CHEK1
MOL005000	PRSS1
MOL005000	CCNA2
MOL005000	NCOA2
MOL005000	CAMKMT
MOL005001	ESR1
MOL005001	AR
MOL005001	PTGS2
MOL005001	KDR
MOL005001	HSP90AB1
MOL005001	PRSS1
MOL005001	CCNA2
MOL005001	NCOA2
MOL005001	CAMKMT
MOL005003	NOS2
MOL005003	PTGS1
MOL005003	CHRM3
MOL005003	KCNH2
MOL005003	CHRM1
MOL005003	ESR1
MOL005003	AR
MOL005003	SCN5A
MOL005003	PPARG
MOL005003	CHRM5
MOL005003	PTGS2
MOL005003	RXRA
MOL005003	ACHE
MOL005003	ADRA1B
MOL005003	ADRB2
MOL005003	ESR2
MOL005003	MAPK14
MOL005003	GSK3B
MOL005003	HSP90AB1
MOL005003	CDK2
MOL005003	RXRB
MOL005003	PRSS1
MOL005003	CCNA2
MOL005003	NCOA2
MOL005003	CAMKMT

MOL005007	NOS2
MOL005007	PTGS1
MOL005007	KCNH2
MOL005007	ESR1
MOL005007	AR
MOL005007	SCN5A
MOL005007	PPARG
MOL005007	PTGS2
MOL005007	F7
MOL005007	KDR
MOL005007	ACHE
MOL005007	ESR2
MOL005007	PPARD
MOL005007	GSK3B
MOL005007	HSP90AB1
MOL005007	CDK2
MOL005007	PRSS1
MOL005007	CCNA2
MOL005007	NCOA2
MOL005007	NCOA1
MOL005007	CAMKMT
MOL005008	NOS2
MOL005008	ESR1
MOL005008	AR
MOL005008	PTGS2
MOL005008	F7
MOL005008	ACHE
MOL005008	ESR2
MOL005008	DPP4
MOL005008	GSK3B
MOL005008	HSP90AB1
MOL005008	CDK2
MOL005008	PRSS1
MOL005008	CCNA2
MOL005008	CAMKMT
MOL005012	NOS2
MOL005012	ESR1
MOL005012	AR
MOL005012	SCN5A
MOL005012	PPARG
MOL005012	PTGS2
MOL005012	ESR2
MOL005012	DPP4

MOL005012	MAPK14
MOL005012	GSK3B
MOL005012	CDK2
MOL005012	CHEK1
MOL005012	PRSS1
MOL005012	CCNA2
MOL005012	CAMKMT
MOL005016	NOS2
MOL005016	PTGS1
MOL005016	ESR1
MOL005016	AR
MOL005016	SCN5A
MOL005016	PPARG
MOL005016	PTGS2
MOL005016	RXRA
MOL005016	ESR2
MOL005016	DPP4
MOL005016	MAPK14
MOL005016	GSK3B
MOL005016	HSP90AB1
MOL005016	CDK2
MOL005016	CHEK1
MOL005016	PRSS1
MOL005016	CCNA2
MOL005016	NCOA2
MOL005016	CAMKMT
MOL005017	ESR1
MOL005017	AR
MOL005017	PPARG
MOL005017	PTGS2
MOL005017	KDR
MOL005017	MAPK14
MOL005017	GSK3B
MOL005017	HSP90AB1
MOL005017	CDK2
MOL005017	CHEK1
MOL005017	CCNA2
MOL005018	NOS2
MOL005018	ESR1
MOL005018	PTGS2
MOL005018	ESR2
MOL005018	NCOA2
MOL005018	CAMKMT

MOL005020	NOS2
MOL005020	ESR1
MOL005020	AR
MOL005020	SCN5A
MOL005020	PPARG
MOL005020	PTGS2
MOL005020	ADRB2
MOL005020	ESR2
MOL005020	MAPK14
MOL005020	HSP90AB1
MOL005020	CDK2
MOL005020	CHEK1
MOL005020	PRSS1
MOL005020	CCNA2
MOL005020	NCOA2
MOL005020	CAMKMT
MOL000098	PTGS1
MOL000098	AR
MOL000098	PPARG
MOL000098	PTGS2
MOL000098	HSP90AB1
MOL000098	NCOA2
MOL000098	DPP4
MOL000098	AKR1B1
MOL000098	PRSS1
MOL000098	KCNH2
MOL000098	SCN5A
MOL000098	ADRB2
MOL000098	MMP3
MOL000098	F7
MOL000098	RXRA
MOL000098	ACHE
MOL000098	GABRA1
MOL000098	MAOB
MOL000098	RELA
MOL000098	EGFR
MOL000098	AKT1
MOL000098	VEGFA
MOL000098	CCND1
MOL000098	BCL2
MOL000098	BCL2L1
MOL000098	FOS
MOL000098	CDKN1A

MOL000098	EIF6
MOL000098	BAX
MOL000098	CASP9
MOL000098	PLAU
MOL000098	MMP2
MOL000098	MMP9
MOL000098	MAPK1
MOL000098	IL10RA
MOL000098	EGF
MOL000098	RB1
MOL000098	TNFAIP6
MOL000098	JUN
MOL000098	IL6R
MOL000098	AHSA1
MOL000098	CASP3
MOL000098	TP53
MOL000098	ELK1
MOL000098	NFKBIA
MOL000098	POR
MOL000098	ODC1
MOL000098	CASP8
MOL000098	TOP1
MOL000098	RAF1
MOL000098	SOD1
MOL000098	PRKCA
MOL000098	MMP1
MOL000098	HIF1A
MOL000098	STAT1
MOL000098	RUNX1T1
MOL000098	CDK1
MOL000098	HSPA5
MOL000098	ERBB2
MOL000098	PPARG
MOL000098	ACACA
MOL000098	HMOX1
MOL000098	CYP3A4
MOL000098	CYP1A2
MOL000098	CAV1
MOL000098	MYC
MOL000098	F3
MOL000098	GJA1
MOL000098	CYP1A1
MOL000098	ICAM1

MOL000098	IL1B
MOL000098	CCL2
MOL000098	SELE
MOL000098	VCAM1
MOL000098	PTGER3
MOL000098	CXCL8
MOL000098	PRKCB
MOL000098	BIRC5
MOL000098	DUOX2
MOL000098	NOS3
MOL000098	HSPB1
MOL000098	SULT1E1
MOL000098	IL2RA
MOL000098	NR1I2
MOL000098	CYP1B1
MOL000098	CCNB1
MOL000098	PLAT
MOL000098	THBD
MOL000098	SERPINE1
MOL000098	COL1A1
MOL000098	IFNG
MOL000098	ALOX5
MOL000098	IL1A
MOL000098	MPO
MOL000098	TOP2A
MOL000098	NCF1
MOL000098	ABCG2
MOL000098	HAS2
MOL000098	GSTP1
MOL000098	NFE2L2
MOL000098	NQO1
MOL000098	PARP1
MOL000098	AHR
MOL000098	PSMD3
MOL000098	SLC2A4
MOL000098	COL3A1
MOL000098	CXCL11
MOL000098	CXCL2
MOL000098	DCAF5
MOL000098	NR1I3
MOL000098	CHEK2
MOL000098	INSRR
MOL000098	CLDN4

MOL000098	PPARA
MOL000098	PPARD
MOL000098	HSF1
MOL000098	CXCL10
MOL000098	CHUK
MOL000098	SPP1
MOL000098	RUNX2
MOL000098	RASSF1
MOL000098	E2F1
MOL000098	E2F2
MOL000098	ACP3
MOL000098	CTSD
MOL000098	IGFBP3
MOL000098	IGF2
MOL000098	CD40LG
MOL000098	IRF1
MOL000098	ERBB3
MOL000098	PON1
MOL000098	DIO1
MOL000098	PCOLCE
MOL000098	NPEPPS
MOL000098	HK2
MOL000098	RASA1
MOL000098	GSTM1
MOL000098	GSTM2
MOL001736	PTGS1
MOL001736	PTGS2
MOL001736	HSP90AB1
MOL000358	PGR
MOL000358	NCOA2
MOL000358	PTGS1
MOL000358	PTGS2
MOL000358	HSP90AB1
MOL000358	KCNH2
MOL000358	DRD1
MOL000358	CHRM3
MOL000358	CHRM1
MOL000358	SCN5A
MOL000358	CHRM4
MOL000358	ADRA1A
MOL000358	CHRM2
MOL000358	ADRA1B
MOL000358	ADRB2

MOL000358	CHRNA2
MOL000358	SLC6A4
MOL000358	OPRM1
MOL000358	GABRA1
MOL000358	BCL2
MOL000358	BAX
MOL000358	CASP9
MOL000358	JUN
MOL000358	CASP3
MOL000358	CASP8
MOL000358	PRKCA
MOL000358	PON1
MOL000358	MAP2
MOL000359	PGR
MOL000359	NCOA2
MOL000359	NR3C2
MOL000492	PTGS1
MOL000492	ESR1
MOL000492	PTGS2
MOL000492	HSP90AB1
MOL000492	DPEP1
MOL000492	NCOA2
MOL000492	CAMKMT
MOL000492	RXRA
MOL000492	CAT
MOL000492	HAS2
MOL000073	PTGS1
MOL000073	ESR1
MOL000073	PTGS2
MOL000073	HSP90AB1
MOL000073	DPEP1
MOL004576	PTGS1
MOL004576	PTGS2
MOL004576	HSP90AB1
MOL004576	RXRA
MOL004576	AKR1B1
MOL004576	RELA
MOL004576	ICAM1
MOL004576	DGAT2
MOL004576	MTTP
MOL004576	APOB

Table S1: Types of nodes	
Term	Type
PTGS2	gene
PGR	gene
CHRM3	gene
CHRM1	gene
AR	gene
SCN5A	gene
RXRA	gene
ACHE	gene
ADRA1A	gene
CHRM2	gene
ADRB2	gene
OPRM1	gene
GABRA1	gene
DPP4	gene
NCOA2	gene
NCOA1	gene
NR3C2	gene
ADRA1B	gene
GRIA2	gene
ADH1B	gene
ADH1C	gene
LYG1	gene
PTGS1	gene
SLC6A2	gene
HTR3A	gene
ADRA1D	gene
PRSS1	gene
HSP90AB1	gene
ESR1	gene
DPEP1	gene
MAOB	gene
SLC6A4	gene
NOS2	gene
PPARG	gene
KDR	gene
MAPK14	gene
GSK3B	gene

CHEK1	gene
CCNA2	gene
ESR2	gene
CDK2	gene
DRD1	gene
CHRM5	gene
SLC6A3	gene
MAPK10	gene
OPRD1	gene
PYGM	gene
PPARD	gene
AKR1B1	gene
F7	gene
RELA	gene
NCF1	gene
OLR1	gene
ADRB1	gene
LTA4H	gene
JUN	gene
IL4	gene
ATP5F1B	gene
HSD3B2	gene
HSD3B1	gene
IKBKB	gene
AKT1	gene
BCL2	gene
BAX	gene
TNFAIP6	gene
AHSA1	gene
CASP3	gene
MAPK8	gene
MMP1	gene
STAT1	gene
CDK1	gene
HMOX1	gene
CYP3A4	gene
CYP1A2	gene
CYP1A1	gene
ICAM1	gene
SELE	gene
VCAM1	gene
NR1I2	gene
CYP1B1	gene

ALOX5	gene
HAS2	gene
GSTP1	gene
AHR	gene
PSMD3	gene
SLC2A4	gene
NR1I3	gene
INSRR	gene
DIO1	gene
PPP3CA	gene
GSTM1	gene
GSTM2	gene
AKR1C3	gene
SLPI	gene
MAPK3	gene
MAPK1	gene
FASN	gene
LDLR	gene
BAD	gene
SOD1	gene
CAT	gene
MTTP	gene
APOB	gene
PLB1	gene
HMGCR	gene
CYP19A1	gene
UGT1A1	gene
PPARA	gene
SREBF1	gene
GSR	gene
ABCC1	gene
ADIPOR2	gene
SOAT2	gene
GOT1	gene
ABAT	gene
CES1	gene
SOAT1	gene
KCNH2	gene
BACE2	gene
CA2	gene
RXRB	gene
STAT3	gene
CCND1	gene

EIF6	gene
RB1	gene
CDK4	gene
FOSL2	gene
MMP3	gene
EGFR	gene
VEGFA	gene
BCL2L1	gene
FOS	gene
CDKN1A	gene
CASP9	gene
PLAU	gene
MMP2	gene
MMP9	gene
IL10RA	gene
EGF	gene
IL6R	gene
TP53	gene
ELK1	gene
NFKBIA	gene
POR	gene
ODC1	gene
CASP8	gene
TOP1	gene
RAF1	gene
PRKCA	gene
HIF1A	gene
RUNX1T1	gene
HSPA5	gene
ERBB2	gene
ACACA	gene
CAV1	gene
MYC	gene
F3	gene
GJA1	gene
IL1B	gene
CCL2	gene
PTGER3	gene
CXCL8	gene
PRKCB	gene
BIRC5	gene
DUOX2	gene
NOS3	gene

HSPB1	gene
SULT1E1	gene
IL2RA	gene
CCNB1	gene
PLAT	gene
THBD	gene
SERPINE1	gene
COL1A1	gene
IFNG	gene
IL1A	gene
MPO	gene
TOP2A	gene
ABCG2	gene
NFE2L2	gene
NQO1	gene
PARP1	gene
COL3A1	gene
CXCL11	gene
CXCL2	gene
DCAF5	gene
CHEK2	gene
CLDN4	gene
HSF1	gene
CXCL10	gene
CHUK	gene
SPP1	gene
RUNX2	gene
RASSF1	gene
E2F1	gene
E2F2	gene
ACP3	gene
CTSD	gene
IGFBP3	gene
IGF2	gene
CD40LG	gene
IRF1	gene
ERBB3	gene
PON1	gene
PCOLCE	gene
NPEPPS	gene
HK2	gene
RASA1	gene
MAP2	gene

DGAT2	gene
MOL004829	Gancao
MOL004980	Gancao
MOL000239	Gancao
MOL005012	Gancao
MOL001736	Guizhi
MOL004824	Gancao
MOL004815	Gancao
MOL004866	Gancao
MOL004941	Gancao
MOL000279	Fuling
MOL004884	Gancao
MOL003896	Gancao
MOL004911	Gancao
MOL005016	Gancao
MOL004891	Gancao
MOL005018	Gancao
MOL000358	Guizhi
MOL003656	Gancao
MOL004957	Gancao
MOL001792	Gancao
MOL004907	Gancao
MOL004935	Gancao
MOL000492	Guizhi
MOL000392	Gancao
MOL001484	Gancao
MOL004988	Gancao
MOL004863	Gancao
MOL004974	Gancao
MOL004898	Gancao
MOL002565	Gancao
MOL000283	Fuling
MOL004806	Gancao
MOL004808	Gancao
MOL000022	Baizhu
MOL004856	Gancao
MOL000417	Gancao
MOL004833	Gancao
MOL004948	Gancao
MOL004912	Gancao
MOL005020	Gancao
MOL004827	Gancao
MOL000211	Gancao

MOL004849	Gancao
MOL000282	Fuling
MOL004904	Gancao
MOL004910	Gancao
MOL004885	Gancao
MOL000275	Fuling
MOL004959	Gancao
MOL005007	Gancao
MOL004838	Gancao
MOL004814	Gancao
MOL004913	Gancao
MOL004864	Gancao
MOL004835	Gancao
MOL004828	Gancao
MOL000033	Baizhu
MOL005000	Gancao
MOL000098	Gancao
MOL000073	Guizhi
MOL004882	Gancao
MOL005003	Gancao
MOL004857	Gancao
MOL004945	Gancao
MOL004991	Gancao
MOL000354	Gancao
MOL005008	Gancao
MOL004811	Gancao
MOL000359	multiDrug
MOL004883	Gancao
MOL000072	Baizhu
MOL000296	Fuling
MOL000273	Fuling
MOL004989	Gancao
MOL004820	Gancao
MOL004966	Gancao
MOL004841	Gancao
MOL004805	Gancao
MOL004924	Gancao
MOL004915	Gancao
MOL004855	Gancao
MOL000049	Baizhu
MOL004914	Gancao
MOL004990	Gancao
MOL000500	Gancao

MOL004993	Gancao
MOL004985	Gancao
MOL002311	Gancao
MOL004978	Gancao
MOL004903	Gancao
MOL004576	Guizhi
MOL005017	Gancao
MOL004810	Gancao
MOL004949	Gancao
MOL004328	Gancao
MOL004848	Gancao
MOL004961	Gancao
MOL005001	Gancao
MOL004879	Gancao
MOL000422	Gancao
MOL004908	Gancao
MOL004996	Gancao
MOL000497	Gancao
