# Supplementary material for: Exploring the molecular mechanism of Ling-Gui-Zhu-Gan decoction for the treatment of type 2 diabetes mellitus based on network pharmacology and molecular docking: A review
Source: Medicine (Baltimore). 2023 Mar 24;102(12):e33210. doi: 10.1097/MD.0000000000033210 (PMC10036033; doi:10.1097/MD.0000000000033210)
Supplement: Supplementary file 3 [file medi-102-e33210-s003.pdf]

Supplementary file 3-Results of GO and KEGG pathway analyses

Exploring the mechanism of Ling-Gui-Zhu-Gan-decoction for the treatment of type 2 diabetes based on network pharmacology and molecular docking

Feng Long, MS[1],Zhe Zhang, MS[1],ChunXiu Luo, MS[1],Xiao Lei, PHD[1]*，Jinlian Guo, MS[1],Lin An, MS[1]

1.Department of traditional Chinese Medicine, affiliated Hospital of North Sichuan Medical College, Nanchong, Sichuan 637000.

*** Correspondence**: Xiao Lei，2424932797@qq.com

S1:GO analysis results

Class	ID	Pathway	GeneRatio	BgRatio	pvalue	p.adjust	qvalue	geneID	Count
BP	GO:0009410	response to xenobiotic stimulus	50/210	411/18800	1.59E-37	6.76E-34	3.16E-34	PTGS2/ADRA1A/SLC6A2/HSP90AB1/DPEP1/MAOB/SLC6A4/NOS2/DRD1/SLC6A3/JUN/BCL2/CASP3/STAT1/CDK1/HMOX1/CYP3A4/CYP1A2/CYP1A1/NR1I2/CYP1B1/GSTP1/AHR/GSTM1/GSTM2/BAD/SOD1/CAT/UGT1A1/SREBF1/ABCC1/ABAT/KCNH2/CCND1	50

								/RB1/CDK4/FOS/MMP2/TP53/POR/TOP1/HSPA5/MYC/IL1B/COL1A1/NFE2L2/NQO1/CHEK2/HSF1/E2F1	
BP	GO:0010038	response to metal ion	41/210	351/18800	5.65E-30	1.20E-26	5.61E-27	PTGS2/SCN5A/DPEP1/MAOB/SLC6A3/NCF1/LTA4H/JUN/AKT1/BCL2/CASP3/MAPK8/CDK1/HMOX1/CYP1A2/CYP1A1/VCAM1/PPP3CA/AKR1C3/MAPK3/MAPK1/BAD/SOD1/CAT/MTTP/ABAT/CCND1/EGFR/FOS/CASP9/MMP9/CASP8/HIF1A/HSPA5/CAV1/IL1A/NFE2L2/NQO1/PARP1/HSF1/CHUK	41
BP	GO:0062197	cellular response to chemical stress	39/210	332/18800	1.31E-28	1.86E-25	8.69E-26	PTGS2/AKR1B1/RELA/NCF1/JUN/AKT1/BCL2/CASP3/MAPK8/CDK1/HMOX1/CYP1B1/ALOX5/SLC2A4/AKR1C3/MAPK3/MAPK1/BAD/SOD1/CAT/GSR/ABCC1/MMP3/EGFR/FOS/MMP2/MMP9/TP53/HIF1A/CAV1/MYC/NOS3/HSPB1/MPO/NFE2L2/NQO1/PARP1/HSF1/CHUK	39
BP	GO:0032496	response to lipopolysaccharide	38/210	333/18800	2.08E-27	2.20E-24	1.03E-24	PTGS2/OPRM1/MAOB/NOS2/MAPK14/PPARD/RELA/AKT1/CASP3/MAPK8/CYP1A1/	38

								SELE/VCAM1/GSTP1/SLPI/MAPK3/MAPK1/APOB/UGT1A1/CDK4/FOS/CASP9/IL10RA/NFKBIA/CASP8/PRKCA/IL1B/CCL2/CXCL8/NOS3/THBD/SERPINE1/IL1A/MPO/CXCL11/CXCL2/HSF1/CXCL10	
BP	GO:1901652	response to peptide	44/210	491/18800	2.78E-27	2.36E-24	1.10E-24	PTGS2/ADRB2/PPARG/MAPK14/GSK3B/CCNA2/F7/RELA/LTA4H/AKT1/STAT1/ICAM1/VCAM1/CYP1B1/SLC2A4/INSRR/CAT/PPARA/SREBF1/ABCC1/GOT1/CA2/STAT3/EIF6/RB1/CDK4/MMP3/MMP2/MMP9/TP53/NFKBIA/POR/CAV1/MYC/GJA1/IL1B/PRKCB/PLAT/COL1A1/NFE2L2/PARP1/COL3A1/HSF1/IGF2	44
BP	GO:0031667	response to nutrient levels	42/210	446/18800	6.76E-27	4.78E-24	2.24E-24	PTGS2/RXRA/ADRB2/OPRM1/NCOA1/SLC6A4/PPARG/MAPK14/PPARD/F7/ADRB1/AKT1/BCL2/MAPK8/STAT1/HMOX1/CYP1A1/VCAM1/CYP1B1/AKR1C3/MAPK3/MAPK1/LDLR/SOD1/CAT/UGT1A1/PPARA/SREBF1/CCND1/CDKN1A/TP53/PO	42

								R/HSPA5/COL1A1/IL1A/MPO/NFE2L2/NQO1/HSF1/CXCL10/SPP1/PON1	
BP	GO:0009991	response to extracellular stimulus	43/210	479/18800	1.09E-26	6.60E-24	3.09E-24	PTGS2/RXRA/ADRB2/OPRM1/NCOA1/SLC6A4/PPARG/MAPK14/PPARD/F7/ADRB1/AKT1/BCL2/MAPK8/STAT1/HMOX1/CYP1A1/VCAM1/CYP1B1/AKR1C3/MAPK3/MAPK1/LDLR/SOD1/CAT/UGT1A1/PPARA/SREBF1/CCND1/FOS/CDKN1A/TP53/POR/HSPA5/COL1A1/IL1A/MPO/NFE2L2/NQO1/HSF1/CXCL10/SPP1/PON1	43
BP	GO:0002237	response to molecule of bacterial origin	38/210	354/18800	2.01E-26	1.01E-23	4.74E-24	PTGS2/OPRM1/MAOB/NOS2/MAPK14/PPARD/RELA/AKT1/CASP3/MAPK8/CYP1A1/SELE/VCAM1/GSTP1/SLPI/MAPK3/MAPK1/APOB/UGT1A1/CDK4/FOS/CASP9/IL10RA/NFKBIA/CASP8/PRKCA/IL1B/CCL2/CXCL8/NOS3/THBD/SERPINE1/IL1A/MPO/CXCL11/CXCL2/HSF1/CXCL10	38
BP	GO:0000302	response to reactive	31/210	203/18800	2.30E-26	1.01E-23	4.74E-24	RELA/NCF1/JUN/AKT1/BCL2/CASP3/MAPK8/STAT	31

		oxygen species						1/CDK1/HMOX1/CYP1B1/GSTP1/AKR1C3/MAPK3/MAPK1/BAD/SOD1/CAT/EGFR/FOS/MMP2/MMP9/HIF1A/NOS3/COL1A1/IL1A/MPO/NFE2L2/NQO1/HSF1/CHUK	
BP	GO:0006979	response to oxidative stress	41/210	433/18800	2.38E-26	1.01E-23	4.74E-24	PTGS2/PTGS1/RELA/NCF1/JUN/AKT1/BCL2/CASP3/MAPK8/STAT1/CDK1/HMOX1/CYP1B1/ALOX5/GSTP1/AKR1C3/MAPK3/MAPK1/BAD/SOD1/CAT/GSR/ABCC1/MMP3/EGFR/FOS/MMP2/MMP9/TP53/HIF1A/DUOX2/NOS3/HSPB1/COL1A1/IL1A/MPO/NFE2L2/NQO1/PARP1/HSF1/CHUK	41
BP	GO:0036293	response to decreased oxygen levels	34/210	299/18800	1.64E-24	6.35E-22	2.97E-22	PTGS2/DPP4/SLC6A4/NOS2/PPARG/CCNA2/OPRD1/PPARD/F7/AKT1/BCL2/CASP3/HMOX1/CYP1A1/VCAM1/SLC2A4/BAD/CAT/PPARA/ABAT/VEGFA/PLAU/MMP2/TP53/HIF1A/CAV1/MYC/PLAT/IL1A/NFE2L2/HSF1/E2F1/NPEPPS/HK2	34
BP	GO:0070482	response to oxygen	35/210	324/18800	1.86E-24	6.57E-22	3.07E-22	PTGS2/DPP4/SLC6A4/NOS2/PPARG/CCNA2/OPRD1	35

		levels						/PPARD/F7/AKT1/BCL2/CASP3/HMOX1/CYP1A1/VCAM1/SLC2A4/BAD/CAT/PPARA/ABAT/VEGFA/PLAU/MMP2/TP53/HIF1A/CAV1/MYC/PLAT/COL1A1/IL1A/NFE2L2/HSF1/E2F1/NPEPPS/HK2	
BP	GO:0001666	response to hypoxia	33/210	286/18800	5.21E-24	1.70E-21	7.96E-22	PTGS2/DPP4/SLC6A4/NOS2/PPARG/CCNA2/OPRD1/PPARD/F7/BCL2/CASP3/HMOX1/CYP1A1/VCAM1/SLC2A4/BAD/CAT/PPARA/ABAT/VEGFA/PLAU/MMP2/TP53/HIF1A/CAV1/MYC/PLAT/IL1A/NFE2L2/HSF1/E2F1/NPEPPS/HK2	33
BP	GO:1901654	response to ketone	28/210	190/18800	1.92E-23	5.84E-21	2.73E-21	AR/NCOA2/MAOB/F7/HSD3B2/HSD3B1/AKT1/CYP1B1/AHR/AKR1C3/BAD/SREBF1/CCND1/CDK4/BCL2L1/FOS/CASP9/ELK1/POR/ACACA/CAV1/MYC/PLAT/NQO1/PARP1/CLDN4/HSF1/SPP1	28
BP	GO:0097305	response to alcohol	30/210	235/18800	3.48E-23	9.86E-21	4.61E-21	OPRM1/MAOB/SLC6A3/F7/HSD3B2/HSD3B1/AKT1/CDK1/VCAM1/CYP1B1/AHR/SLC2A4/AKR1C3/BAD/SOD1/CAT/UGT1	30

								A1/PPARA/SREBF1/ABAT/CES1/CCND1/CDK4/BCL2L1/FOS/CASP8/ACACA/MYC/NQO1/PARP1	
BP	GO:0034599	cellular response to oxidative stress	32/210	284/18800	5.61E-23	1.49E-20	6.97E-21	RELA/NCF1/JUN/AKT1/BCL2/MAPK8/CDK1/HMOX1/CYP1B1/ALOX5/AKR1C3/MAPK3/MAPK1/SOD1/CAT/GSR/ABCC1/MMP3/EGFR/FOS/MMP2/MMP9/TP53/HIF1A/NOS3/HSPB1/MPO/NFE2L2/NQO1/PARP1/HSF1/CHUK	32
BP	GO:0009314	response to radiation	37/210	438/18800	4.57E-22	1.14E-19	5.35E-20	PTGS2/OPRM1/MAPK14/CHEK1/DRD1/MAPK10/RELA/AKT1/BCL2/BAX/CASP3/MAPK8/MMP1/VCAM1/CAT/HMGCR/CCND1/MMP3/EGFR/BCL2L1/FOS/CDKN1A/CASP9/MMP2/MMP9/TP53/ELK1/HIF1A/HSPA5/MYC/THBD/IL1A/PARP1/COL3A1/CHEK2/HSF1/CXCL10	37
BP	GO:0032355	response to estradiol	23/210	123/18800	7.13E-22	1.68E-19	7.87E-20	PTGS2/ESR1/SLC6A4/CCNA2/ESR2/F7/CASP3/CYP1B1/BAD/CAT/APOB/CYP19A1/UGT1A1/STAT3/CCND1/EGFR/CASP9/MMP2/CASP8/MYC/COL1A1/NQO	23

								1/HSF1	
BP	GO:0048545	response to steroid hormone	32/210	330/18800	5.79E-21	1.29E-18	6.05E-19	PTGS2/PGR/AR/RXRA/NCOA2/NR3C2/ESR1/MAOB/ESR2/PPARD/HSD3B2/HSD3B1/BCL2/CASP3/CYP1B1/AKR1C3/BAD/UGT1A1/PPARA/SREBF1/GOT1/RXRB/CCND1/FOS/CASP9/POR/CAV1/PLAT/COL1A1/PARP1/CLDN4/SPP1	32
BP	GO:0071241	cellular response to inorganic substance	27/210	221/18800	1.95E-20	4.15E-18	1.94E-18	PTGS2/SCN5A/DPEP1/CCNA2/CDK2/NCF1/JUN/AKT1/MAPK8/HMOX1/CYP1A2/CYP1A1/AKR1C3/MAPK3/MAPK1/BAD/SOD1/MMP3/EGFR/FOS/MMP9/HSPA5/NFE2L2/NQO1/PARP1/HSF1/CHUK	27
BP	GO:0042060	wound healing	35/210	429/18800	2.10E-20	4.25E-18	1.99E-18	OPRM1/PPARG/KDR/MAPK14/PPARD/F7/CASP3/HMOX1/ALOX5/PPP3CA/PPARA/ADIPOR2/ABAT/VEGFA/CDKN1A/PLAU/RAF1/PRKCA/HIF1A/ERBB2/CAV1/F3/DUOX2/NOS3/HSPB1/PLAT/THBD/SERPINE1/COL1A1/IL1A/NFE2L2/COL3A1/CLDN4/CD40LG/ERBB3	35
BP	GO:0071276	cellular response	15/210	37/18800	2.39E-20	4.62E-18	2.16E-18	NCF1/JUN/AKT1/MAPK8/HMOX1/	15

		to cadmium ion						CYP1A2/AKR1C3/MAPK3/MAPK1/SOD1/EGFR/FOS/MMP9/HSF1/CHUK	
BP	GO:0043434	response to peptide hormone	34/210	404/18800	2.91E-20	5.37E-18	2.51E-18	PTGS2/PPARG/MAPK14/GSK3B/CCNA2/F7/RELA/LTA4H/AKT1/STAT1/CYP1B1/SLC2A4/INSRR/CAT/PPARA/SREBF1/GOT1/CA2/STAT3/EIF6/RB1/CDK4/POR/CAV1/MYC/IL1B/PRKCB/PLAT/COL1A1/NFE2L2/PARP1/COL3A1/HSF1/IGF2	34
BP	GO:0008202	steroid metabolic process	31/210	323/18800	3.38E-20	5.98E-18	2.80E-18	RXRA/ESR1/PPARD/AKR1B1/IL4/HSD3B2/HSD3B1/CYP3A4/CYP1A2/CYP1A1/NR1I2/CYP1B1/AKR1C3/LDLR/SOD1/CAT/APOB/HMGCR/CYP19A1/UGT1A1/SREBF1/SOAT2/CES1/SOAT1/POR/SULT1E1/IFNG/IL1A/SPP1/PON1/DGAT2	31
BP	GO:0046686	response to cadmium ion	17/210	59/18800	6.26E-20	1.06E-17	4.98E-18	NCF1/JUN/AKT1/MAPK8/CDK1/HMOX1/CYP1A2/AKR1C3/MAPK3/MAPK1/SOD1/CAT/EGFR/FOS/MMP9/HSF1/CHUK	17
BP	GO:0071466	cellular response to xenobiot	24/210	168/18800	6.80E-20	1.11E-17	5.20E-18	DPEP1/NOS2/CYP3A4/CYP1A2/CYP1A1/NR1I2/CYP1B1/GSTP1/AHR/G	24

		ic stimulus						STM1/GSTM2/UGT1A1/KCNH2/RB1/TP53/POR/HSPA5/MYC/IL1B/NFE2L2/NQO1/CHEK2/HSF1/E2F1	
BP	GO:0007584	response to nutrient	23/210	150/18800	8.01E-20	1.26E-17	5.90E-18	PTGS2/RXRA/NCOA1/SLC6A4/PPARG/PPARD/F7/STAT1/HMOX1/CYP1A1/VCAM1/CYP1B1/AKR1C3/CAT/UGT1A1/CCND1/POR/COL1A1/IL1A/NQO1/HSF1/CXCL10/SPP1	23
BP	GO:0048608	reproductive structure development	34/210	433/18800	2.58E-19	3.92E-17	1.83E-17	PTGS2/PGR/AR/NCOA1/HSP90AB1/ESR1/PPARG/KDR/MAPK14/PPARD/AKT1/BCL2/BAX/TNFAIP6/CASP3/CYP1B1/AKR1C3/MAPK1/SOD1/CYP19A1/CCND1/EGFR/VEGFA/BCL2L1/MMP2/CASP8/HIF1A/HSPA5/MYC/GJA1/NOS3/IL1A/SPP1/IGF2	34
BP	GO:0061458	reproductive system development	34/210	436/18800	3.21E-19	4.70E-17	2.20E-17	PTGS2/PGR/AR/NCOA1/HSP90AB1/ESR1/PPARG/KDR/MAPK14/PPARD/AKT1/BCL2/BAX/TNFAIP6/CASP3/CYP1B1/AKR1C3/MAPK1/SOD1/CYP19A1/CCND1/EGFR/VEGFA/BCL2L1/MMP2/CASP8/HIF1A/HSPA5/MYC/GJA1/NOS3/IL1A/SPP1/IGF2	34

BP	GO:0071214	cellular response to abiotic stimulus	30/210	323/18800	3.65E-19	5.00E-17	2.34E-17	PTGS2/MAPK14/CHEK1/AKR1B1/BAX/CASP3/MAPK8/MMP1/SLC2A4/INSRR/MAPK3/BAD/MMP3/BCL2L1/CDKN1A/CASP9/MMP2/MMP9/TP53/ELK1/CASP8/HSPA5/MYC/GJA1/IL1B/COL1A1/PARP1/CHEK2/HSF1/IRF1	30
BP	GO:0104004	cellular response to environmental stimulus	30/210	323/18800	3.65E-19	5.00E-17	2.34E-17	PTGS2/MAPK14/CHEK1/AKR1B1/BAX/CASP3/MAPK8/MMP1/SLC2A4/INSRR/MAPK3/BAD/MMP3/BCL2L1/CDKN1A/CASP9/MMP2/MMP9/TP53/ELK1/CASP8/HSPA5/MYC/GJA1/IL1B/COL1A1/PARP1/CHEK2/HSF1/IRF1	30
BP	GO:1901653	cellular response to peptide	31/210	361/18800	8.73E-19	1.16E-16	5.42E-17	ADRB2/PPARG/GSK3B/CCNA2/RELA/AKT1/STAT1/ICAM1/VCAM1/CYP1B1/SLC2A4/INSRR/SREBF1/ABCC1/GOT1/CA2/STAT3/RB1/CDK4/TP53/POR/CAV1/MYC/GJA1/IL1B/PRKCB/PLAT/NFE2L2/PARP1/HSF1/IGF2	31
BP	GO:0034614	cellular response to reactive oxygen	22/210	147/18800	9.04E-19	1.16E-16	5.44E-17	RELA/NCF1/JUN/AKT1/MAPK8/CDK1/CYP1B1/AKR1C3/MAPK3/MAPK1/SOD1/CAT/EG	22

		species						FR/FOS/MMP2/MMP9/NOS3/MPO/NFE2L2/NQO1/HSF1/CHUK	
BP	GO:0010817	regulation of hormone levels	35/210	496/18800	2.21E-18	2.76E-16	1.29E-16	DPP4/ADH1B/ADH1C/ESR1/NOS2/PPARG/PPARD/AKR1B1/HSD3B2/HSD3B1/CYP3A4/CYP1A2/CYP1A1/CYP1B1/ALOX5/DIO1/PPP3CA/AKR1C3/BAD/PLB1/CYP19A1/UGT1A1/SREBF1/ABAT/BACE2/POR/RAF1/HIF1A/GJA1/IL1B/DUOX2/SULT1E1/IFNG/SPP1/DGAT2	35
BP	GO:0048511	rhythmic process	28/210	297/18800	4.11E-18	4.99E-16	2.33E-16	PGR/OPRM1/NCOA2/ESR1/SLC6A4/NOS2/PPARG/GSK3B/MAPK10/F7/ADRB1/TNFAIP6/CASP3/MAPK8/CDK1/CYP1B1/HAS2/AHR/MTTP/PPARA/SREBF1/MMP2/TP53/TOP1/HSPA5/NOS3/TOP2A/CLDN4	28
BP	GO:0009636	response to toxic substance	26/210	247/18800	4.51E-18	5.32E-16	2.49E-16	PTGS2/PTGS1/MAOB/SLC6A4/OPRD1/BCL2/BAX/CDK1/CYP1A1/CYP1B1/GSTP1/AHR/GSTM1/GSTM2/SOD1/CAT/GSR/CES1/FOS/DUOX2/NOS3/MPO/ABCG2/NFE2L2/NQO1/PON1	26
BP	GO:00725	reactive	25/210	231/1	1.05E-	1.21E-15	5.65E-16	MAOB/NOS2/MA	25

	93	oxygen species metabolic process		8800	17			PK14/NCF1/BCL2/CYP1A2/CYP1A1/CYP1B1/ALOX5/GSTP1/AKR1C3/SOD1/CAT/PPARA/EIF6/MMP3/CDKN1A/TP53/HIF1A/DUOX2/NOS3/MPO/NFE2L2/NQO1/HK2	
BP	GO:0062012	regulation of small molecule metabolic process	29/210	342/18800	1.78E-17	1.99E-15	9.33E-16	PTGS2/NCOA2/NOS2/PPARG/GSK3B/PPARD/IL4/AKT1/AKR1C3/LDLR/BAD/SOD1/APOB/PPARA/SREBF1/CES1/STAT3/EIF6/TP53/POR/HIF1A/CAV1/MYC/IL1B/NOS3/IFNG/PARP1/IGF2/DGAT2	29
BP	GO:0071248	cellular response to metal ion	23/210	191/18800	2.08E-17	2.27E-15	1.06E-15	PTGS2/SCN5A/DPEP1/NCF1/JUN/AKT1/MAPK8/HMOX1/CYP1A2/CYP1A1/AKR1C3/MAPK3/MAPK1/SOD1/EGFR/FOS/MMP9/HSPA5/NFE2L2/NQO1/PARP1/HSF1/CHUK	23
BP	GO:0071216	cellular response to biotic stimulus	25/210	256/18800	1.24E-16	1.31E-14	6.15E-15	NOS2/MAPK14/GSK3B/PPARD/RELA/AKT1/MAPK8/GSTP1/MAPK3/MAPK1/CDK4/TP53/NFKBIA/PRKCA/HSPA5/IL1B/CCL2/CXCL8/NOS3/SERPINE1/IL1A/CXCL11/CXCL2/HSF1/CXCL10	25
BP	GO:00487	gland	31/210	431/1	1.37E-	1.42E-14	6.64E-15	PGR/AR/ESR1/SL	31

	32	development		8800	16			C6A3/RELA/JUN/AKT1/BCL2/BAX/HMOX1/CYP1A1/CYP1B1/MAPK3/MAPK1/FASN/SOD1/CYP19A1/UGT1A1/CCND1/EGFR/VEGFA/MMP2/EGF/ELK1/RAF1/HIF1A/CAV1/MYC/GJA1/IGF2/HK2	
BP	GO:0009411	response to UV	20/210	146/18800	2.18E-16	2.20E-14	1.03E-14	PTGS2/CHEK1/RELA/AKT1/BCL2/BAX/CASP3/MAPK8/MMP1/CAT/CCND1/MMP3/EGFR/CDKN1A/CASP9/MMP2/MMP9/TP53/MYC/PARP1	20
BP	GO:0034612	response to tumor necrosis factor	24/210	249/18800	7.04E-16	6.96E-14	3.26E-14	PTGS2/MAPK14/RELA/IKBKB/AKT1/CASP3/STAT1/SELE/VCAM1/CYP1B1/HAS2/GSTP1/SLC2A4/MAPK3/MAPK1/APOB/TP53/NFKBIA/CASP8/CCL2/CXCL8/COL1A1/NFE2L2/CHUK	24
BP	GO:0009416	response to light stimulus	26/210	310/18800	1.21E-15	1.16E-13	5.45E-14	PTGS2/CHEK1/DRD1/MAPK10/RELA/AKT1/BCL2/BAX/CASP3/MAPK8/MMP1/CAT/HMGCR/CCND1/MMP3/EGFR/FOS/CDKN1A/CASP9/MMP2/MMP9/TP53/ELK1/HIF1A/MYC/PARP1	26
BP	GO:2001233	regulation of apoptoti	28/210	370/18800	1.26E-15	1.19E-13	5.56E-14	PTGS2/AR/GSK3B/RELA/IL4/AKT1/BCL2/BAX/HM	28

		c signaling pathway						OX1/ICAM1/GSTP1/BAD/SOD1/RB1/BCL2L1/MMP9/TP53/RAF1/HIF1A/CAV1/MYC/IL1B/NOS3/HSPB1/SERPINE1/IL1A/NFE2L2/PARP1	
BP	GO:0042445	hormone metabolic process	23/210	230/18800	1.31E-15	1.21E-13	5.65E-14	DPP4/ADH1B/ADH1C/ESR1/AKR1B1/HSD3B2/HSD3B1/CYP3A4/CYP1A2/CYP1A1/CYP1B1/DIO1/AKR1C3/PLB1/CYP19A1/UGT1A1/BACE2/POR/HIF1A/DUOX2/SULT1E1/SPP1/DGAT2	23
BP	GO:0050673	epithelial cell proliferation	30/210	443/18800	2.25E-15	2.03E-13	9.52E-14	PGR/AR/SCN5A/ESR1/PPARG/KDR/PPARD/JUN/AKT1/BAX/STAT1/HMOX1/ALOX5/HAS2/MAPK1/BAD/STAT3/CCND1/RB1/EGFR/VEGFA/EGF/PRKCA/HIF1A/ERBB2/CAV1/MYC/F3/CCL2/IGF2	30
BP	GO:0050678	regulation of epithelial cell proliferation	28/210	382/18800	2.84E-15	2.51E-13	1.18E-13	PGR/AR/SCN5A/PPARG/KDR/PPARD/JUN/AKT1/BAX/STAT1/HMOX1/ALOX5/HAS2/BAD/STAT3/CCND1/RB1/EGFR/VEGFA/EGF/PRKCA/HIF1A/ERBB2/CAV1/MYC/F3/CCL2/IGF2	28
BP	GO:0071375	cellular response	25/210	294/18800	3.21E-15	2.76E-13	1.29E-13	PPARG/GSK3B/CCNA2/RELA/AKT	25

		to peptide hormone stimulus						1/STAT1/CYP1B1/SLC2A4/INSRR/SREBF1/GOT1/CA2/STAT3/RB1/CDK4/POR/CAV1/MYC/IL1B/PRKCB/PLAT/NFE2L2/PARP1/HSF1/IGF2	
BP	GO:0070997	neuron death	27/210	353/18800	3.25E-15	2.76E-13	1.29E-13	HSP90AB1/GSK3B/JUN/AKT1/BCL2/BAX/CASP3/HMOX1/BAD/SOD1/PPARA/RB1/BCL2L1/FOS/CASP9/TP53/ELK1/CASP8/HIF1A/HSPA5/CCL2/IFNG/NQO1/PARP1/HSF1/ERBB3/RASA1	27
BP	GO:0019221	cytokine-mediated signaling pathway	31/210	486/18800	3.83E-15	3.19E-13	1.49E-13	HSP90AB1/PPARG/RELA/IKBKB/AKT1/STAT1/GSTP1/MAPK3/BAD/ADIPOR2/STAT3/IL10RA/IL6R/TP53/NFKBIA/CASP8/HIF1A/CAV1/F3/IL1B/CCL2/CXCL8/DUOX2/IL2RA/IFNG/IL1A/CXCL11/CXCL2/CXCL10/CHUK/IRF1	31
BP	GO:0071222	cellular response to lipopolysaccharide	22/210	217/18800	4.20E-15	3.43E-13	1.61E-13	NOS2/MAPK14/PPARD/RELA/AKT1/MAPK8/GSTP1/MAPK3/MAPK1/CDK4/NFKBIA/PRKCA/IL1B/CCL2/CXCL8/NOS3/SERPINE1/IL1A/CXCL11/CXCL2/HSF1/CXCL10	22
BP	GO:0006066	alcohol metaboli	27/210	361/18800	5.64E-15	4.52E-13	2.11E-13	RXRA/ADH1B/ADH1C/PPARD/AK	27

		c process						R1B1/IL4/CYP3A4/CYP1A2/CYP1A1/CYP1B1/AKR1C3/LDLR/SOD1/CAT/APOB/PLB1/HMGCR/SREBF1/SOAT2/GOT1/CES1/SOAT1/POR/SULT1E1/ACP3/PON1/DGAT2	
BP	GO:0097191	extrinsic apoptotic signaling pathway	22/210	221/18800	6.17E-15	4.85E-13	2.27E-13	AR/GSK3B/RELA/IL4/AKT1/BCL2/BAX/HMOX1/ICAM1/GSTP1/BAD/BCL2L1/IL6R/CASP8/RAF1/CAV1/IL1B/NOS3/SERPINE1/IFNG/IL1A/ERBB3	22
BP	GO:0016125	sterol metabolic process	19/210	154/18800	9.07E-15	7.00E-13	3.28E-13	RXRA/PPARD/IL4/CYP3A4/CYP1A2/CYP1B1/LDLR/SOD1/CAT/APOB/HMGCR/CYP19A1/SREBF1/SOAT2/CES1/SOAT1/POR/PON1/DGAT2	19
BP	GO:0071219	cellular response to molecule of bacterial origin	22/210	229/18800	1.30E-14	9.84E-13	4.60E-13	NOS2/MAPK14/PPARD/RELA/AKT1/MAPK8/GSTP1/MAPK3/MAPK1/CDK4/NFKBIA/PRKCA/IL1B/CCL2/CXCL8/NOS3/SERPINE1/IL1A/CXCL11/CXCL2/HSF1/CXCL10	22
BP	GO:1901214	regulation of neuron death	25/210	313/18800	1.37E-14	1.02E-12	4.77E-13	HSP90AB1/GSK3B/JUN/AKT1/BCL2/BAX/CASP3/HMOX1/BAD/SOD1/PPARA/BCL2L1/FOS/CASP9/TP53/ELK1/CASP8/HIF	25

								1A/CCL2/IFNG/NQO1/PARP1/HSF1/ERBB3/RASA1	
BP	GO:2001234	negative regulation of apoptotic signaling pathway	22/210	230/18800	1.42E-14	1.04E-12	4.87E-13	PTGS2/AR/GSK3B/RELA/IL4/AKT1/BCL2/BAX/HMOX1/ICAM1/GSTP1/RB1/BCL2L1/MMP9/RAF1/HIF1A/IL1B/NOS3/HSPB1/SERPINE1/IL1A/NFE2L2	22
BP	GO:0045471	response to ethanol	17/210	119/18800	2.05E-14	1.47E-12	6.89E-13	OPRM1/MAOB/SLC6A3/CDK1/VCAM1/SLC2A4/BAD/SOD1/CAT/UGT1A1/PPARA/SREBF1/ABAT/CCND1/CASP8/MYC/NQO1	17
BP	GO:0010212	response to ionizing radiation	18/210	142/18800	2.92E-14	2.06E-12	9.66E-13	MAPK14/BCL2/BAX/CASP3/VCAM1/CCND1/BCL2L1/CDKN1A/TP53/ELK1/HSPA5/MYC/THBD/IL1A/PARP1/CHEK2/HSF1/CXCL10	18
BP	GO:0010332	response to gamma radiation	13/210	56/18800	3.60E-14	2.50E-12	1.17E-12	BCL2/BAX/BCL2L1/CDKN1A/TP53/ELK1/HSPA5/MYC/IL1A/PARP1/CHEK2/HSF1/CXCL10	13
BP	GO:0006631	fatty acid metabolic process	27/210	395/18800	5.05E-14	3.46E-12	1.62E-12	PTGS2/PTGS1/PPARG/MAPK14/PPARD/AKR1B1/AKT1/CYP3A4/CYP1A2/CYP1A1/CYP1B1/ALOX5/GSTP1/GSTM1/GSTM2/AKR1C3/FASN/PPARA/SREBF1/ADIPOR2/CES1/EIF	27

								6/POR/ACACA/CAV1/IL1B/DGAT2	
BP	GO:0051098	regulation of binding	26/210	366/18800	6.26E-14	4.22E-12	1.98E-12	ADRB2/HSP90AB1/PPARG/GSK3B/JUN/AKT1/BAX/MAPK8/HMOX1/PPP3CA/SLPI/MAPK3/PPARA/RB1/CDKN1A/MMP9/EGF/NFKBIA/CAV1/MYC/IFNG/PARP1/HSF1/E2F1/PON1/MAP2	26
BP	GO:0018209	peptidyl-serine modification	25/210	338/18800	7.91E-14	5.25E-12	2.46E-12	PTGS2/HSP90AB1/MAPK14/GSK3B/CDK2/DRD1/OPRD1/IKBKB/AKT1/BCL2/BAX/MAPK8/CDK1/MAPK1/EGFR/VEGFA/TOP1/RAF1/PRKCA/CAV1/PRKCB/IFNG/PARP1/CHEK2/CHUK	25
BP	GO:0008406	gonad development	21/210	226/18800	1.03E-13	6.76E-12	3.16E-12	PGR/AR/ESR1/KDR/BCL2/BAX/TNFAIP6/CASP3/CYP1B1/AKR1C3/SOD1/CYP19A1/CCND1/VEGFA/BCL2L1/MMP2/HSPA5/MYC/GJA1/NOS3/IL1A	21
BP	GO:0071356	cellular response to tumor necrosis factor	21/210	229/18800	1.34E-13	8.53E-12	3.99E-12	MAPK14/RELA/IKBKB/AKT1/STAT1/VCAM1/CYP1B1/HAS2/GSTP1/SLC2A4/MAPK3/MAPK1/APOB/TP53/NFKBIA/CASP8/CCL2/CXCL8/COL1A1/NFE2L2/CHUK	21
BP	GO:00181	peptidyl	24/210	315/1	1.35E-	8.53E-12	3.99E-12	PTGS2/HSP90AB1	24

	05	-serine phosphorylation		8800	13			/MAPK14/GSK3B/CDK2/DRD1/OPRD1/IKBKB/AKT1/BCL2/BAX/MAPK8/CDK1/MAPK1/EGFR/VEGFA/TOP1/RAF1/PRKCA/CAV1/PRKCB/IFNG/CHEK2/CHUK	
BP	GO:0014074	response to purine-containing compound	17/210	134/18800	1.53E-13	9.56E-12	4.47E-12	PTGS2/SLC6A4/CHEK1/SLC6A3/STAT1/CYP1B1/AHR/GSTM2/SOD1/SREBF1/FOS/HSPA5/IL1B/DUOX2/PLAT/THBD/COL1A1	17
BP	GO:0045137	development of primary sexual characteristics	21/210	231/18800	1.59E-13	9.81E-12	4.59E-12	PGR/AR/ESR1/KDR/BCL2/BAX/TNFAIP6/CASP3/CYP1B1/AKR1C3/SOD1/CYP19A1/CCND1/VEGFA/BCL2L1/MMP2/HSPA5/MYC/GJA1/NOS3/IL1A	21
BP	GO:0050878	regulation of body fluid levels	26/210	382/18800	1.70E-13	1.03E-11	4.80E-12	CHRM3/CHRM1/MAPK14/SLC6A3/AKR1B1/F7/HAS2/ABAT/CCND1/VEGFA/PLAU/PRKCA/HIF1A/CAV1/F3/GJA1/NOS3/HSPB1/PLAT/THBD/SERPINE1/NFE2L2/COL3A1/CLDN4/CD40LG/HK2	26
BP	GO:0031960	response to corticosteroid	18/210	157/18800	1.72E-13	1.03E-11	4.80E-12	PTGS2/MAOB/HSD3B2/HSD3B1/BCL2/CASP3/CYP1B1/AKR1C3/BAD/UGT1A1/GOT1/CC	18

								ND1/FOS/CASP9/POR/PLAT/COL1A1/PARP1	
BP	GO:2001237	negative regulation of extrinsic apoptotic signaling pathway	15/210	97/18800	2.20E-13	1.29E-11	6.04E-12	AR/GSK3B/RELA/IL4/AKT1/BCL2/HMOX1/ICAM1/GSTP1/BCL2L1/RAF1/IL1B/NOS3/SERPINE1/IL1A	15
BP	GO:0034754	cellular hormone metabolic process	17/210	137/18800	2.22E-13	1.29E-11	6.04E-12	ADH1B/ADH1C/ESR1/AKR1B1/HSD3B2/HSD3B1/CYP3A4/CYP1A2/CYP1A1/CYP1B1/AKR1C3/PLB1/CYP19A1/UGT1A1/SULT1E1/SPP1/DGAT2	17
BP	GO:0097193	intrinsic apoptotic signaling pathway	23/210	295/18800	2.76E-13	1.59E-11	7.42E-12	PTGS2/AKT1/BCL2/BAX/CASP3/HMOX1/CYP1B1/BAD/SOD1/BCL2L1/CDKN1A/CASP9/MMP9/TP53/HIF1A/CAV1/MYC/HSPB1/NFE2L2/PARP1/CHEK2/E2F1/E2F2	23
BP	GO:0008203	cholesterol metabolic process	17/210	139/18800	2.83E-13	1.60E-11	7.49E-12	RXRA/PPARD/IL4/CYP3A4/CYP1A2/LDLR/SOD1/CAT/APOB/HMGCR/SREBF1/SOAT2/CES1/SOAT1/POR/PON1/DGAT2	17
BP	GO:0048144	fibroblast proliferation	14/210	81/18800	2.88E-13	1.61E-11	7.52E-12	ESR1/CCNA2/JUN/BAX/GSTP1/CDK4/FOSL2/EGFR/CDKN1A/TP53/MYC/CCNB1/COL3A1/E2F1	14

BP	GO:0050727	regulation of inflammatory response	26/210	394/18800	3.48E-13	1.92E-11	8.97E-12	PTGS2/ESR1/PPARG/MAPK14/PPARD/RELA/NCF1/IL4/TNFAIP6/SELE/ALOX5/GSTP1/LDLR/SOD1/CYP19A1/PPARA/ABCC1/RB1/MMP3/MMP9/NFKBIA/IL1B/PTGER3/IL2RA/SERPINE1/IFNG	26
BP	GO:0071453	cellular response to oxygen levels	18/210	166/18800	4.54E-13	2.47E-11	1.16E-11	PTGS2/PPARG/CCNA2/OPRD1/PPARD/AKT1/BCL2/HMOX1/SLC2A4/BAD/VEGFA/TP53/HIF1A/CAV1/MYC/NFE2L2/E2F1/NPEPPS	18
BP	GO:0062013	positive regulation of small molecule metabolic process	17/210	146/18800	6.40E-13	3.44E-11	1.61E-11	PTGS2/NOS2/PPARG/PPARD/IL4/AKT1/PPARA/SREBF1/CES1/POR/HIF1A/MYC/IL1B/NOS3/IFNG/IGF2/DGAT2	17
BP	GO:0071496	cellular response to external stimulus	23/210	309/18800	7.30E-13	3.83E-11	1.79E-11	PTGS2/RXRA/NCOA1/CHEK1/BCL2/MAPK8/HMOX1/VCAM1/AKR1C3/MAPK3/MAPK1/BAD/PPARA/SREBF1/FOS/CDKN1A/TP53/CASP8/HSPA5/IL1B/COL1A1/NFE2L2/IRF1	23
BP	GO:0008585	female gonad development	15/210	105/18800	7.31E-13	3.83E-11	1.79E-11	PGR/ESR1/KDR/BCL2/BAX/TNFAIP6/CASP3/SOD1/CYP19A1/VEGFA/BCL2L1/MMP2/H	15

								SPA5/MYC/NOS3	
BP	GO:1902652	secondary alcohol metabolic process	17/210	149/18800	8.97E-13	4.65E-11	2.17E-11	RXRA/PPARD/IL4/CYP3A4/CYP1A2/LDLR/SOD1/CAT/APOB/HMGCR/SREBF1/SOAT2/CES1/SOAT1/POR/PON1/DGAT2	17
BP	GO:0036294	cellular response to decreased oxygen levels	17/210	151/18800	1.12E-12	5.72E-11	2.68E-11	PTGS2/PPARG/CCNA2/OPRD1/PPARD/AKT1/BCL2/HMOX1/SLC2A4/BAD/VEGFA/TP53/HIF1A/MYC/NFE2L2/E2F1/NPEPPS	17
BP	GO:0009612	response to mechanical stimulus	19/210	201/18800	1.20E-12	6.05E-11	2.83E-11	PTGS2/MAPK14/CHEK1/RELA/JUN/MAPK8/STAT1/MAPK3/BAD/FOS/MMP2/NFKBIA/CASP8/RAF1/IL1B/COL1A1/MPO/CXCL10/IRF1	19
BP	GO:0046545	development of primary female sexual characteristics	15/210	109/18800	1.28E-12	6.41E-11	3.00E-11	PGR/ESR1/KDR/BCL2/BAX/TNFAIP6/CASP3/SOD1/CYP19A1/VEGFA/BCL2L1/MMP2/HSPA5/MYC/NOS3	15
BP	GO:0001503	ossification	26/210	420/18800	1.50E-12	7.43E-11	3.48E-11	PTGS2/ACHE/ADRB2/PPARG/MAPK14/GSK3B/ATP5F1B/AKT1/BCL2/TNFAIP6/ALOX5/NR1I3/MAPK3/MAPK1/FASN/CAT/EGFR/MMP2/MMP9/IL6R/HIF1A/GJA1/COL1A1/SPP1/RUNX2/IGF2	26
BP	GO:0006091	generation of	28/210	494/18800	1.65E-12	8.04E-11	3.76E-11	ADH1B/ADH1C/MAOB/NOS2/GS	28

		precursor metabolites and energy						K3B/PYGM/PPARD/AKR1B1/NCF1/IL4/ATP5F1B/AKT1/CDK1/CYP1A2/CAT/CYP19A1/PPARA/GSR/STAT3/EIF6/TP53/POR/HIF1A/MYC/CCNB1/IFNG/IGF2/HK2	
BP	GO:0044703	multi-organism reproductive process	19/210	205/18800	1.70E-12	8.22E-11	3.85E-11	PTGS2/PGR/AR/ESR1/SLC6A4/PPARD/AKT1/BCL2/CYP1A1/SOD1/VEGFA/FOS/MMP2/MMP9/GJA1/IL1B/THBD/CLDN4/SP1	19
BP	GO:0030879	mammary gland development	16/210	135/18800	2.37E-12	1.13E-10	5.30E-11	PGR/AR/ESR1/SLC6A3/AKT1/BAX/MAPK1/FASN/CYP19A1/CCND1/VEGFA/EGF/HIF1A/CAV1/GJA1/HK2	16
BP	GO:0050679	positive regulation of epithelial cell proliferation	19/210	211/18800	2.85E-12	1.34E-10	6.29E-11	AR/SCN5A/KDR/JUN/AKT1/HMOX1/HAS2/BAD/STAT3/CCND1/EGFR/VEGFA/EGF/PRKCA/HIF1A/ERBB2/MYC/F3/IGF2	19
BP	GO:0044706	multi-multicellular organism process	19/210	213/18800	3.37E-12	1.57E-10	7.36E-11	PTGS2/PGR/AR/ESR1/SLC6A4/PPARD/AKT1/BCL2/CYP1A1/SOD1/VEGFA/FOS/MMP2/MMP9/GJA1/IL1B/THBD/CLDN4/SP1	19
BP	GO:0071478	cellular response to radiation	18/210	187/18800	3.53E-12	1.63E-10	7.62E-11	PTGS2/MAPK14/CHEK1/BAX/MMP1/MMP3/BCL2L1/CDKN1A/CASP9/MMP2/MMP9/T	18

								P53/ELK1/HSPA5/MYC/PARP1/CHEK2/HSF1	
BP	GO:0048145	regulation of fibroblast proliferation	13/210	79/18800	4.01E-12	1.83E-10	8.57E-11	ESR1/CCNA2/JUN/BAX/GSTP1/CDK4/FOSL2/EGFR/CDKN1A/TP53/MYC/CCNB1/E2F1	13
BP	GO:1904019	epithelial cell apoptotic process	15/210	118/18800	4.19E-12	1.89E-10	8.85E-11	KDR/IL4/HMOX1/ICAM1/AKR1C3/BAD/PPARA/RB1/BCL2L1/CCL2/SERPINE1/NFE2L2/E2F1/E2F2/CD40LG	15
BP	GO:0035094	response to nicotine	11/210	49/18800	5.22E-12	2.33E-10	1.09E-10	SLC6A3/RELA/BCL2/CASP3/HMOX1/VCAM1/MAPK1/BAD/PPARA/ABAT/MMP2	11
BP	GO:0071456	cellular response to hypoxia	16/210	143/18800	5.81E-12	2.57E-10	1.20E-10	PTGS2/PPARG/CCNA2/OPRD1/PPARD/BCL2/HMOX1/SLC2A4/BAD/VEGFA/TP53/HIF1A/MYC/NFE2L2/E2F1/NPEPPS	16
BP	GO:0045765	regulation of angiogenesis	23/210	345/18800	7.11E-12	3.09E-10	1.44E-10	PPARG/KDR/STAT1/HMOX1/CYP1B1/ALOX5/STAT3/VEGFA/PRKCA/HIF1A/ERBB2/F3/IL1B/CXCL8/PRKCB/NOS3/HSPB1/SERPINE1/IL1A/NFE2L2/CXCL10/E2F2/HK2	23
BP	GO:0007548	sex differentiation	21/210	281/18800	7.12E-12	3.09E-10	1.44E-10	PGR/AR/ESR1/KDR/BCL2/BAX/TNFAIP6/CASP3/CYP1B1/AKR1C3/SOD1/CYP19A1/CCND1/VEGFA/BCL	21

								2L1/MMP2/HSPA5/MYC/GJA1/NOS3/IL1A	
BP	GO:0097237	cellular response to toxic substance	15/210	123/18800	7.73E-12	3.31E-10	1.55E-10	PTGS2/PTGS1/OPRD1/GSTP1/GSTM1/GSTM2/SOD1/CAT/GSR/DUOX2/NOS3/MPO/ABCG2/NFE2L2/NQO1	15
BP	GO:0051090	regulation of DNA-binding transcription factor activity	26/210	452/18800	7.90E-12	3.35E-10	1.57E-10	AR/ESR1/PPARG/ESR2/MAPK10/OPRD1/RELA/IKBKB/AKT1/MAPK8/HMOX1/CYP1B1/PPP3CA/MAPK3/CAT/STAT3/RB1/VEGFA/NFKBIA/CAV1/IL1B/PRKCB/HSF1/CHUK/E2F1/CD40LG	26
BP	GO:0046660	female sex differentiation	15/210	124/18800	8.70E-12	3.62E-10	1.70E-10	PGR/ESR1/KDR/BCL2/BAX/TNFAIP6/CASP3/SOD1/CYP19A1/VEGFA/BCL2L1/MMP2/HSPA5/MYC/NOS3	15
BP	GO:0046683	response to organophosphorus	15/210	124/18800	8.70E-12	3.62E-10	1.70E-10	PTGS2/SLC6A4/SLC6A3/STAT1/CYP1B1/AHR/SOD1/SREBF1/FOS/HSPA5/IL1B/DUOX2/PLAT/THBD/COL1A1	15
BP	GO:0050900	leukocyte migration	24/210	384/18800	9.46E-12	3.90E-10	1.82E-10	DPP4/F7/IL4/AKT1/TNFAIP6/HMOX1/ICAM1/SELE/VCAM1/ALOX5/MAPK3/MAPK1/CYP19A1/VEGFA/MMP9/IL6R/IL1B/CCL2/CXCL8/SERPINE1/IL1A/CXCL11/CXCL2/CX	24

								CL10	
BP	GO:1901342	regulation of vasculature development	23/210	351/18800	1.01E-11	4.13E-10	1.93E-10	PPARG/KDR/STAT1/HMOX1/CYP1B1/ALOX5/STAT3/VEGFA/PRKCA/HIF1A/ERBB2/F3/IL1B/CXCL8/PRKCB/NOS3/HSPB1/SERPINE1/IL1A/NFE2L2/CXCL10/E2F2/HK2	23
BP	GO:0042391	regulation of membrane potential	25/210	425/18800	1.27E-11	5.13E-10	2.40E-10	CHRM1/SCN5A/ADRA1A/ADRB2/OPRM1/GABRA1/HTR3A/KDR/GSK3B/DRD1/OPRD1/ADRB1/AKT1/BCL2/BAX/PPP3CA/BAD/SOD1/ABAT/KCNH2/BCL2L1/CAV1/MYC/GJA1/PARP1	25
BP	GO:0042542	response to hydrogen peroxide	15/210	128/18800	1.39E-11	5.56E-10	2.60E-10	RELA/BCL2/CASP3/STAT1/CDK1/HMOX1/CYP1B1/BAD/SOD1/CAT/MMP2/COL1A1/NFE2L2/NQO1/HSF1	15
BP	GO:0001659	temperature homeostasis	17/210	177/18800	1.49E-11	5.91E-10	2.76E-10	PTGS2/ACHE/ADRB2/DRD1/ADRB1/IL4/ADIPOR2/ABAT/STAT3/RB1/VEGFA/CAV1/GJA1/IL1B/PTGER3/IL1A/HSF1	17
BP	GO:2001236	regulation of extrinsic apoptotic signaling pathway	16/210	153/18800	1.65E-11	6.48E-10	3.03E-10	AR/GSK3B/RELA/IL4/AKT1/BCL2/HMOX1/ICAM1/GSTP1/BCL2L1/RAF1/CAV1/IL1B/NOS3/SERPINE1/IL1A	16

BP	GO:0007623	circadian rhythm	18/210	205/18800	1.67E-11	6.50E-10	3.04E-10	NCOA2/SLC6A4/NOS2/PPARG/GSK3B/MAPK10/F7/ADRB1/MAPK8/CDK1/AHR/MTTP/PPARA/SREBF1/TP53/TOP1/TOP2A/CLDN4	18
BP	GO:0030522	intracellular receptor signaling pathway	20/210	264/18800	1.80E-11	6.96E-10	3.26E-10	PGR/AR/RXRA/NCOA1/NR3C2/ESR1/PPARG/ESR2/PPARD/RELA/NR1I2/AHR/NR1I3/AKR1C3/PPARA/SREBF1/RXRB/STAT3/NFKBIA/PARP1	20
BP	GO:0120254	olefinic compound metabolic process	16/210	157/18800	2.45E-11	9.37E-10	4.38E-10	PTGS2/ADH1B/ADH1C/PTGS1/AKR1B1/CYP3A4/CYP1A2/CYP1A1/CYP1B1/ALOX5/GSTP1/GSTM2/AKR1C3/PLB1/CYP19A1/DGAT2	16
BP	GO:0045766	positive regulation of angiogenesis	17/210	183/18800	2.54E-11	9.55E-10	4.47E-10	KDR/HMOX1/CYP1B1/STAT3/VEGFA/PRKCA/HIF1A/F3/IL1B/CXCL8/PRKCB/NOS3/HSPB1/SERPINE1/IL1A/NFE2L2/HK2	17
BP	GO:1904018	positive regulation of vasculature development	17/210	183/18800	2.54E-11	9.55E-10	4.47E-10	KDR/HMOX1/CYP1B1/STAT3/VEGFA/PRKCA/HIF1A/F3/IL1B/CXCL8/PRKCB/NOS3/HSPB1/SERPINE1/IL1A/NFE2L2/HK2	17
BP	GO:0007565	female pregnancy	17/210	185/18800	3.02E-11	1.13E-09	5.27E-10	PTGS2/PGR/AR/ESR1/PPARD/AKT1/BCL2/SOD1/VEGFA/FOS/MMP2/MMP9/GJA1/IL1B	17

								/THBD/CLDN4/SP P1	
BP	GO:19907 48	cellular detoxific ation	14/210	115/1 8800	4.02E- 11	1.49E-09	6.95E-10	PTGS2/PTGS1/GS TP1/GSTM1/GST M2/SOD1/CAT/G SR/DUOX2/NOS3/ MPO/ABCG2/NFE 2L2/NQO1	14
BP	GO:19028 93	regulatio n of miRNA transcrip tion	11/210	59/18 800	4.55E- 11	1.67E-09	7.79E-10	PPARG/PPARD/R ELA/JUN/PPARA/ SREBF1/STAT3/F OS/TP53/HIF1A/ MYC	11
BP	GO:00513 84	response to glucocor ticoid	15/210	139/1 8800	4.60E- 11	1.67E-09	7.81E-10	PTGS2/MAOB/HS D3B2/HSD3B1/BC L2/CASP3/CYP1B 1/BAD/UGT1A1/G OT1/CCND1/FOS/ CASP9/POR/PLA T	15
BP	GO:00026 85	regulatio n of leukocyt e migratio n	18/210	218/1 8800	4.66E- 11	1.68E-09	7.85E-10	DPP4/F7/IL4/AKT 1/TNFAIP6/HMO X1/ICAM1/SELE/ MAPK3/MAPK1/ CYP19A1/VEGFA /IL6R/CCL2/CXC L8/SERPINE1/IL1 A/CXCL10	18
BP	GO:00511 00	negative regulatio n of binding	16/210	165/1 8800	5.22E- 11	1.86E-09	8.71E-10	ADRB2/GSK3B/J UN/AKT1/BAX/M APK8/HMOX1/PP P3CA/SLPI/MAPK 3/PPARA/CDKN1 A/NFKBIA/CAV1/ E2F1/MAP2	16
BP	GO:00071 59	leukocyt e cell-cell adhesion	23/210	381/1 8800	5.29E- 11	1.87E-09	8.75E-10	DPP4/RELA/OLR 1/IL4/AKT1/CASP 3/ICAM1/SELE/V CAM1/ALOX5/H AS2/BAD/PPARA/ ERBB2/CAV1/IL1 B/CCL2/IL2RA/IF NG/IL1A/IGF2/CD 40LG/IRF1	23

BP	GO:0061614	miRNA transcription	11/210	60/18800	5.52E-11	1.94E-09	9.06E-10	PPARG/PPARD/RELA/JUN/PPARA/SREBF1/STAT3/FOS/TP53/HIF1A/MYC	11
BP	GO:0022407	regulation of cell-cell adhesion	25/210	456/18800	5.74E-11	2.00E-09	9.36E-10	DPP4/MAPK14/RELA/IL4/AKT1/CASP3/SELE/VCAM1/ALOX5/HAS2/BAD/PPARA/ABAT/VEGFA/PRKCA/ERBB2/CAV1/IL1B/CCL2/IL2RA/IFNG/IL1A/IGF2/CD40LG/IRF1	25
BP	GO:0060326	cell chemotaxis	21/210	315/18800	6.13E-11	2.12E-09	9.90E-10	DPP4/KDR/F7/IL4/TNFAIP6/VCAM1/ALOX5/MAPK3/MAPK1/CYP19A1/ABCC1/VEGFA/IL6R/IL1B/CCL2/CXCL8/HSPB1/SERPINE1/CXCL11/CXCL2/CXCL10	21
BP	GO:0070141	response to UV-A	7/210	14/18800	6.31E-11	2.16E-09	1.01E-09	AKT1/MMP1/CCND1/MMP3/EGFR/MMP2/MMP9	7
BP	GO:0048660	regulation of smooth muscle cell proliferation	16/210	170/18800	8.19E-11	2.78E-09	1.30E-09	PTGS2/PPARG/PPARD/JUN/AKT1/STAT1/HMOX1/CDKN1A/MMP2/MMP9/IL6R/MYC/GJA1/NOS3/IFNG/IGFBP3	16
BP	GO:0030099	myeloid cell differentiation	23/210	391/18800	8.86E-11	2.99E-09	1.40E-09	PPARG/MAPK14/JUN/IL4/TNFAIP6/CASP3/STAT1/FASN/STAT3/EIF6/RB1/VEGFA/FOS/CASP9/MMP9/NFKBIA/CASP8/PRKCA/HIF1A/MYC/IFNG/PARP1/HSF1	23
BP	GO:00321	negative	24/210	429/1	9.46E-	3.16E-09	1.48E-09	DPP4/PPARG/MA	24

	02	regulation of response to external stimulus		8800	11			PK14/DRD1/SLC6A3/PPARD/IL4/TNFAIP6/ALOX5/GSTP1/LDLR/SOD1/CYP19A1/PPARA/ABAT/RB1/PLAU/CCL2/NOS3/IL2RA/PLAT/THBD/SERPINE1/SPP1	
BP	GO:0070542	response to fatty acid	11/210	63/18800	9.63E-11	3.19E-09	1.49E-09	PTGS2/AKR1C3/LDLR/BAD/CAT/SREBF1/CDK4/PLAT/E2F1/PON1/DGAT2	11
BP	GO:0032868	response to insulin	19/210	259/18800	1.03E-10	3.36E-09	1.57E-09	PPARG/MAPK14/GSK3B/AKT1/STAT1/SLC2A4/INSRR/CAT/PPARA/SREBF1/GOT1/EIF6/RB1/CDK4/MYC/IL1B/PRKCB/PARP1/IGF2	19
BP	GO:2000377	regulation of reactive oxygen species metabolic process	15/210	147/18800	1.03E-10	3.36E-09	1.57E-09	MAPK14/BCL2/CYP1B1/ALOX5/GSTP1/AKR1C3/SOD1/PPARA/EIF6/MMP3/CDKN1A/TP53/HIF1A/NFE2L2/HK2	15
BP	GO:0048659	smooth muscle cell proliferation	16/210	173/18800	1.07E-10	3.46E-09	1.62E-09	PTGS2/PPARG/PPARD/JUN/AKT1/STAT1/HMOX1/CDKN1A/MMP2/MMP9/IL6R/MYC/GJA1/NOS3/IFNG/IGFBP3	16
BP	GO:0048146	positive regulation of fibroblast proliferation	10/210	48/18800	1.10E-10	3.55E-09	1.66E-09	ESR1/CCNA2/JUN/CDK4/FOSL2/EGFR/CDKN1A/MYC/CCNB1/E2F1	10

BP	GO:0010634	positive regulation of epithelial cell migration	16/210	174/18800	1.16E-10	3.71E-09	1.74E-09	PTGS2/KDR/JUN/ATP5F1B/AKT1/HMOX1/HAS2/VEGFA/MMP9/EGF/PRKCA/HIF1A/NOS3/HSPB1/IFNG/NFE2L2	16
BP	GO:0046890	regulation of lipid biosynthetic process	16/210	175/18800	1.27E-10	4.02E-09	1.88E-09	PTGS2/AKT1/AKR1C3/LDLR/SOD1/APOB/PPARA/SREBF1/CES1/EIF6/CDK4/POR/IL1B/IFNG/IL1A/DGAT2	16
BP	GO:0033002	muscle cell proliferation	18/210	233/18800	1.40E-10	4.40E-09	2.06E-09	PTGS2/PPARG/MAPK14/PPARD/JUN/AKT1/STAT1/CDK1/HMOX1/CDKN1A/MMP2/MMP9/IL6R/MYC/GJA1/NOS3/IFNG/IGFBP3	18
BP	GO:0043281	regulation of cysteine-type endopeptidase activity involved in apoptotic process	17/210	204/18800	1.42E-10	4.44E-09	2.08E-09	PTGS2/DPEP1/PPARG/AKT1/BAX/BAD/VEGFA/CASP9/MMP9/POR/CASP8/RAF1/MYC/F3/BIRC5/HSF1/CTSD	17
BP	GO:0001523	retinoid metabolic process	12/210	84/18800	1.54E-10	4.77E-09	2.23E-09	ADH1B/ADH1C/PPARD/AKR1B1/CYP3A4/CYP1A2/CYP1A1/CYP1B1/AKR1C3/PLB1/UGT1A1/DGAT2	12
BP	GO:0001936	regulation of endothelial cell	16/210	178/18800	1.64E-10	5.03E-09	2.36E-09	PPARG/KDR/JUN/AKT1/STAT1/HMOX1/ALOX5/STAT3/VEGFA/EGF/	16

		proliferation						PRKCA/HIF1A/CAV1/F3/CCL2/IGF2	
BP	GO:0009266	response to temperature stimulus	16/210	180/18800	1.93E-10	5.91E-09	2.76E-09	PTGS2/ADRB2/HSP90AB1/GSK3B/ADRB1/AKT1/HMOX1/SOD1/FOS/NFKBIA/CASP8/NOS3/HSPB1/IL1A/HSF1/CXCL10	16
BP	GO:0031649	heat generation	7/210	16/18800	2.06E-10	6.26E-09	2.93E-09	PTGS2/ADRB2/ADRB1/ABAT/IL1B/PTGER3/IL1A	7
BP	GO:0042572	retinol metabolic process	10/210	51/18800	2.09E-10	6.30E-09	2.95E-09	ADH1B/ADH1C/AKR1B1/CYP3A4/CYP1A2/CYP1A1/CYP1B1/AKR1C3/PLB1/DGAT2	10
BP	GO:0016101	diterpenoid metabolic process	12/210	87/18800	2.34E-10	6.96E-09	3.26E-09	ADH1B/ADH1C/PPARD/AKR1B1/CYP3A4/CYP1A2/CYP1A1/CYP1B1/AKR1C3/PLB1/UGT1A1/DGAT2	12
BP	GO:1901216	positive regulation of neuron death	12/210	87/18800	2.34E-10	6.96E-09	3.26E-09	GSK3B/BAX/CASP3/BAD/FOS/CASP9/TP53/ELK1/CASP8/IFNG/NQO1/PARP1	12
BP	GO:0019216	regulation of lipid metabolic process	21/210	339/18800	2.38E-10	7.02E-09	3.29E-09	PTGS2/PPARG/CHRM5/PPARD/AKT1/AKR1C3/LDLR/SOD1/APOB/UGT1A1/PPARA/SREBF1/CES1/EIF6/CDK4/POR/CAV1/IL1B/IFNG/IL1A/DGAT2	21
BP	GO:0051402	neuron apoptotic process	18/210	241/18800	2.43E-10	7.12E-09	3.33E-09	HSP90AB1/JUN/BCL2/BAX/CASP3/HMOX1/SOD1/RB1/BCL2L1/CASP9/TP53/HIF1A/HSPA5/CCL2/NQO1/P	18

								ARP1/ERBB3/RASA1	
BP	GO:0034644	cellular response to UV	12/210	88/18800	2.69E-10	7.82E-09	3.66E-09	PTGS2/CHEK1/BAX/MMP1/MMP3/CDKN1A/CASP9/MMP2/MMP9/TP53/MYC/PARP1	12
BP	GO:1903037	regulation of leukocyte cell-cell adhesion	21/210	344/18800	3.11E-10	8.99E-09	4.20E-09	DPP4/RELA/IL4/AKT1/CASP3/SELE/VCAM1/ALOX5/HAS2/BAD/PPARA/ERBB2/CAV1/IL1B/CCL2/IL2RA/IFNG/IL1A/IGF2/CD40LG/IRF1	21
BP	GO:0043620	regulation of DNA-templated transcription in response to stress	10/210	53/18800	3.13E-10	8.99E-09	4.20E-09	CHEK1/RELA/JUN/HMOX1/VEGFA/TP53/HIF1A/HSPA5/NFE2L2/HSF1	10
BP	GO:0043467	regulation of generation of precursor metabolites and energy	14/210	134/18800	3.21E-10	9.14E-09	4.28E-09	NOS2/GSK3B/IL4/AKT1/CDK1/PPARA/STAT3/EIF6/TP53/HIF1A/MYC/CCNB1/IFNG/IGF2	14
BP	GO:0007568	aging	15/210	163/18800	4.47E-10	1.26E-08	5.92E-09	PTGS2/ADRA1A/SLC6A3/CYP1A1/VCAM1/PPP3CA/CAT/SREBF1/ABAT/FOS/CASP9/MMP2/MPO/NFE2L2/NQO1	15
BP	GO:1904645	response to amyloid-beta	10/210	55/18800	4.61E-10	1.30E-08	6.06E-09	ADRB2/GSK3B/ICAM1/VCAM1/ABCC1/MMP3/MMP2/MMP9/GJA1/PARP1	10

BP	GO:0001935	endothelial cell proliferation	16/210	194/18800	5.88E-10	1.62E-08	7.60E-09	PPARG/KDR/JUN/AKT1/STAT1/HMOX1/ALOX5/STAT3/VEGFA/EGF/PRKCA/HIF1A/CAV1/F3/CCL2/IGF2	16
BP	GO:1901655	cellular response to ketone	12/210	94/18800	5.89E-10	1.62E-08	7.60E-09	AR/AKT1/CYP1B1/AHR/AKR1C3/CDK4/CASP9/ELK1/ACACA/MYC/PLAT/SPP1	12
BP	GO:1904035	regulation of epithelial cell apoptotic process	12/210	94/18800	5.89E-10	1.62E-08	7.60E-09	KDR/IL4/HMOX1/ICAM1/AKR1C3/BAD/PPARA/RB1/CCL2/SERPINE1/NFE2L2/CD40LG	12
BP	GO:0035296	regulation of tube diameter	14/210	141/18800	6.33E-10	1.72E-08	8.07E-09	PTGS2/CHRM3/ADRA1A/ADRB2/ADRA1B/ADRA1D/SLC6A4/DRD1/PPARD/ADRB1/SOD1/MMP2/CAV1/NOS3	14
BP	GO:0097746	blood vessel diameter maintenance	14/210	141/18800	6.33E-10	1.72E-08	8.07E-09	PTGS2/CHRM3/ADRA1A/ADRB2/ADRA1B/ADRA1D/SLC6A4/DRD1/PPARD/ADRB1/SOD1/MMP2/CAV1/NOS3	14
BP	GO:0010631	epithelial cell migration	21/210	358/18800	6.44E-10	1.74E-08	8.15E-09	PTGS2/DPP4/PPARG/KDR/PPARD/JUN/IL4/ATP5F1B/AKT1/HMOX1/CYP1B1/HAS2/VEGFA/MMP9/EGF/PRKCA/HIF1A/NOS3/HSPB1/IFNG/NFE2L2	21
BP	GO:0006720	isoprenoid	13/210	117/18800	6.48E-10	1.74E-08	8.16E-09	ADH1B/ADH1C/PPARD/AKR1B1/C	13

		metabolic process						YP3A4/CYP1A2/CYP1A1/CYP1B1/AKR1C3/PLB1/HMGCR/UGT1A1/DGAT2	
BP	GO:0001541	ovarian follicle development	10/210	57/18800	6.67E-10	1.78E-08	8.34E-09	ESR1/KDR/BCL2/BAX/TNFAIP6/SOD1/VEGFA/BCL2L1/MMP2/MYC	10
BP	GO:0043536	positive regulation of blood vessel endothelial cell migration	11/210	75/18800	6.81E-10	1.81E-08	8.46E-09	PTGS2/KDR/ATP5F1B/AKT1/HMOX1/VEGFA/PRKCA/HIF1A/NOS3/HSPB1/NFE2L2	11
BP	GO:0035150	regulation of tube size	14/210	142/18800	6.96E-10	1.84E-08	8.59E-09	PTGS2/CHRM3/ADRA1A/ADRB2/ADRA1B/ADRA1D/SLC6A4/DRD1/PPARD/ADRB1/SOD1/MMP2/CAV1/NOS3	14
BP	GO:0090132	epithelium migration	21/210	361/18800	7.49E-10	1.96E-08	9.19E-09	PTGS2/DPP4/PPARG/KDR/PPARD/JUN/IL4/ATP5F1B/AKT1/HMOX1/CYP1B1/HAS2/VEGFA/MMP9/EGF/PRKCA/HIF1A/NOS3/HSPB1/IFNG/NFE2L2	21
BP	GO:0043279	response to alkaloid	12/210	97/18800	8.54E-10	2.22E-08	1.04E-08	OPRM1/HSP90AB1/CHEK1/CCNA2/DRD1/SLC6A3/CASP3/GSTM2/ABAT/BCL2L1/HSPA5/NQO1	12
BP	GO:2001116	regulation of cysteine-type	17/210	229/18800	8.58E-10	2.22E-08	1.04E-08	PTGS2/DPEP1/PPARG/AKT1/BAX/BAD/VEGFA/CASP9/MMP9/POR/C	17

		endopeptidase activity						ASP8/RAF1/MYC/F3/BIRC5/HSF1/CTSD	
BP	GO:2001242	regulation of intrinsic apoptotic signaling pathway	15/210	171/18800	8.75E-10	2.25E-08	1.05E-08	PTGS2/AKT1/BCL2/BAX/BAD/SOD1/BCL2L1/MMP9/TP53/HIF1A/CAV1/MYC/HSPB1/NFE2L2/PARP1	15
BP	GO:0048638	regulation of developmental growth	20/210	329/18800	9.06E-10	2.32E-08	1.08E-08	AR/ADRB2/SLC6A4/MAPK14/GSK3B/SLC6A3/PPARD/ADRB1/AKT1/BCL2/CDK1/SOD1/PPARA/STAT3/CDK4/VEGFA/CDKN1A/SPP1/IGF2/MAP2	20
BP	GO:0090130	tissue migration	21/210	366/18800	9.60E-10	2.44E-08	1.14E-08	PTGS2/DPP4/PPARG/KDR/PPARD/JUN/IL4/ATP5F1B/AKT1/HMOX1/CYP1B1/HAS2/VEGFA/MMP9/EGF/PRKCA/HIF1A/NOS3/HSPB1/IFNG/NFE2L2	21
BP	GO:0006721	terpenoid metabolic process	12/210	98/18800	9.64E-10	2.44E-08	1.14E-08	ADH1B/ADH1C/PPARD/AKR1B1/CYP3A4/CYP1A2/CYP1A1/CYP1B1/AKR1C3/PLB1/UGT1A1/DGAT2	12
BP	GO:0032103	positive regulation of response to external stimulus	23/210	442/18800	9.79E-10	2.46E-08	1.15E-08	PTGS2/OPRM1/KDR/F7/IL4/IKBKB/MAPK3/MAPK1/LDLR/ABCC1/VEGFA/IL6R/NFKBIA/PRKCA/F3/IL1B/PTGER3/CXCL8/HSPB1/THBD/SERPINE1/IFNG/C	23

								XCL10	
BP	GO:0006690	icosanoid metabolic process	13/210	121/18800	9.88E-10	2.47E-08	1.16E-08	PTGS2/PTGS1/AKR1B1/LTA4H/CYP1A2/CYP1A1/CYP1B1/ALOX5/GSTP1/GSTM1/AKR1C3/ABCC1/IL1B	13
BP	GO:0003018	vascular process in circulatory system	18/210	263/18800	9.98E-10	2.48E-08	1.16E-08	PTGS2/CHRM3/ADRA1A/ADRB2/ADRA1B/ADRA1D/SLC6A4/DRD1/PPARD/ADRB1/SLC2A4/SOD1/ABCC1/VEGFA/MMP2/CAV1/NOS3/ABCG2	18
BP	GO:1990776	response to angiotensin	8/210	30/18800	1.01E-09	2.48E-08	1.16E-08	PTGS2/RELA/CA2/CAV1/MYC/NFE2L2/COL3A1/HSF1	8
BP	GO:0001890	placenta development	14/210	147/18800	1.10E-09	2.70E-08	1.26E-08	PTGS2/NCOA1/HSP90AB1/PPARG/MAPK14/PPARD/AKT1/MAPK1/SOD1/EGFR/CASP8/HIF1A/SPP1/IGF2	14
BP	GO:0051091	positive regulation of DNA-binding transcription factor activity	18/210	265/18800	1.13E-09	2.75E-08	1.29E-08	AR/ESR1/PPARG/ESR2/OPRD1/RELA/IKBKB/AKT1/PPP3CA/CAT/STAT3/VEGFA/CAV1/IL1B/PRKCB/HSF1/CHUK/CD40LG	18
BP	GO:0002064	epithelial cell development	16/210	203/18800	1.14E-09	2.78E-08	1.30E-08	PGR/AR/ESR1/GSK3B/AKR1B1/IKBKB/AKT1/ICAM1/FASN/BAD/FOSL2/VEGFA/HIF1A/GJA1/IL1B/IL1A	16
BP	GO:0045785	positive regulation of cell	23/210	446/18800	1.16E-09	2.79E-08	1.31E-08	DPP4/KDR/GSK3B/RELA/IL4/AKT1/SELE/VCAM1/A	23

		adhesion						LOX5/HAS2/PPP3CA/BAD/VEGFA/PRKCA/ERBB2/CAV1/IL1B/CCL2/IL2RA/IFNG/IL1A/IGF2/CD40LG	
BP	GO:1903829	positive regulation of protein localization	23/210	446/18800	1.16E-09	2.79E-08	1.31E-08	PTGS2/CHRM1/ACHE/HSP90AB1/PPARG/MAPK14/GSK3B/PPARD/AKT1/TNFAIP6/MAPK8/CDK1/BAD/ABAT/EGFR/EGF/HIF1A/ERBB2/IL1B/IFNG/IL1A/PARP1/NPEPPS	23
BP	GO:0006694	steroid biosynthetic process	15/210	175/18800	1.21E-09	2.88E-08	1.35E-08	AKR1B1/HSD3B2/HSD3B1/CYP3A4/CYP1A1/AKR1C3/SOD1/APOB/HMGCR/CYP19A1/SREBF1/CES1/POR/IFNG/IL1A	15
BP	GO:0098869	cellular oxidant detoxification	12/210	100/18800	1.22E-09	2.90E-08	1.36E-08	PTGS2/PTGS1/GSTP1/GSTM2/SOD1/CAT/GSR/DUOX2/NOS3/MPO/NFE2L2/NQO1	12
BP	GO:0071900	regulation of protein serine/threonine kinase activity	21/210	372/18800	1.29E-09	3.03E-08	1.42E-08	ADRB2/HSP90AB1/PPARG/CCNA2/AKT1/CASP3/GSTP1/HMGCR/CCND1/RB1/EGFR/VEGFA/CDKN1A/EGF/ERBB2/CAV1/IL1B/CCNB1/IFNG/IGF2/CD40LG	21
BP	GO:0003012	muscle system process	23/210	449/18800	1.32E-09	3.11E-08	1.45E-08	PTGS2/CHRM3/SCN5A/ADRA1A/CHRM2/ADRB2/ADRA1B/PPARG/DRD1/HMOX1/PPP3CA/GSTM2/SOD1/PPARA/ABAT/	23

								KCNH2/PRKCA/CAV1/GJA1/IL1B/PTGER3/NOS3/PARP1	
BP	GO:0002688	regulation of leukocyte chemotaxis	13/210	124/18800	1.34E-09	3.13E-08	1.47E-08	DPP4/F7/IL4/TNFAIP6/MAPK3/MAPK1/CYP19A1/VEGFA/IL6R/CCL2/CXCL8/SERPINE1/CXCL10	13
BP	GO:0030595	leukocyte chemotaxis	17/210	236/18800	1.36E-09	3.16E-08	1.48E-08	DPP4/F7/IL4/TNFAIP6/ALOX5/MAPK3/MAPK1/CYP19A1/VEGFA/IL6R/IL1B/CCL2/CXCL8/SERPINE1/CXCL11/CXCL2/CXCL10	17
BP	GO:0016572	histone phosphorylation	9/210	45/18800	1.43E-09	3.28E-08	1.53E-08	CHEK1/CCNA2/CDK2/DRD1/CDK1/MAPK3/PRKCA/IL1B/PRKCB	9
BP	GO:1902895	positive regulation of miRNA transcription	9/210	45/18800	1.43E-09	3.28E-08	1.53E-08	PPARG/RELA/JUN/SREBF1/STAT3/FOS/TP53/HIF1A/MYC	9
BP	GO:0098754	detoxification	14/210	150/18800	1.44E-09	3.28E-08	1.53E-08	PTGS2/PTGS1/GSTP1/GSTM1/GSTM2/SOD1/CAT/GSR/DUOX2/NOS3/MPO/ABCG2/NFE2L2/NQO1	14
BP	GO:0023061	signal release	23/210	451/18800	1.44E-09	3.28E-08	1.53E-08	ADRA1A/OPRM1/MAOB/SLC6A4/NOS2/PPARG/GSK3B/DRD1/PPARD/ALOX5/PPP3CA/BAD/CYP19A1/SREBF1/ABAT/RAF1/HIF1A/GJA1/IL1B/PRKCB/IFNG/IL1A/SPP1	23

BP	GO:0043523	regulation of neuron apoptotic process	16/210	207/18800	1.52E-09	3.44E-08	1.61E-08	HSP90AB1/JUN/BCL2/BAX/CASP3/HMOX1/SOD1/BCL2L1/CASP9/TP53/HIF1A/CCL2/NQO1/PARP1/ERBB3/RASA1	16
BP	GO:0046209	nitric oxide metabolic process	11/210	81/18800	1.59E-09	3.57E-08	1.67E-08	PTGS2/HSP90AB1/NOS2/AKT1/CYP1B1/POR/CAV1/IL1B/NOS3/IFNG/NQO1	11
BP	GO:0051223	regulation of protein transport	24/210	495/18800	1.69E-09	3.78E-08	1.77E-08	PTGS2/CHRM1/ACHE/OPRM1/HSP90AB1/NOS2/PPARG/MAPK14/GSK3B/PPARD/MAPK8/CDK1/ALOX5/PPP3CA/BAD/HMGCR/SREBF1/ABAT/HIF1A/ERBB2/IL1B/IFNG/IL1A/NPEPPS	24
BP	GO:0052547	regulation of peptidase activity	23/210	456/18800	1.78E-09	3.96E-08	1.85E-08	PTGS2/DPEP1/PPARG/AKT1/BAX/SLPI/BAD/STAT3/VEGFA/CASP9/MMP9/POR/CASP8/RAF1/CAV1/MYC/F3/BIRC5/SERPINE1/CLDN4/HSF1/CTSD/PCOLCE	23
BP	GO:2001057	reactive nitrogen species metabolic process	11/210	82/18800	1.82E-09	4.02E-08	1.88E-08	PTGS2/HSP90AB1/NOS2/AKT1/CYP1B1/POR/CAV1/IL1B/NOS3/IFNG/NQO1	11
BP	GO:0002573	myeloid leukocyte differentiation	16/210	210/18800	1.88E-09	4.13E-08	1.93E-08	PPARG/MAPK14/JUN/IL4/TNFAIP6/FASN/RB1/VEGFA/FOS/MMP9/CASP8/PRKCA/MYC	16

								/IFNG/PARP1/HSF1	
BP	GO:0033273	response to vitamin	11/210	83/18800	2.08E-09	4.55E-08	2.13E-08	PTGS2/RXRA/PPARD/F7/CYP1A1/CAT/CCND1/COL1A1/IL1A/CXCL10/SPP1	11
BP	GO:0043618	regulation of transcription from RNA polymerase II promoter in response to stress	9/210	47/18800	2.15E-09	4.69E-08	2.19E-08	CHEK1/JUN/HMOX1/VEGFA/TP53/HIF1A/HSPA5/NFE2L2/HSF1	9
BP	GO:0034308	primary alcohol metabolic process	12/210	105/18800	2.16E-09	4.69E-08	2.19E-08	ADH1B/ADH1C/AKR1B1/CYP3A4/CYP1A2/CYP1A1/CYP1B1/AKR1C3/PLB1/SULT1E1/ACP3/DGAT2	12
BP	GO:0006109	regulation of carbohydrate metabolic process	15/210	183/18800	2.24E-09	4.82E-08	2.25E-08	NCOA2/GSK3B/AKT1/HAS2/BAD/PPARA/STAT3/EIF6/EGF/TP53/HIF1A/MYC/IFNG/IGF2/DGAT2	15
BP	GO:0008217	regulation of blood pressure	15/210	183/18800	2.24E-09	4.82E-08	2.25E-08	PTGS2/AR/ADRA1A/ADRB2/ADRA1B/PTGS1/ADRA1D/NOS2/PPARG/ADRB1/HMOX1/SOD1/PPARA/ABAT/NOS3	15
BP	GO:0031668	cellular response to extracellular	17/210	245/18800	2.41E-09	5.12E-08	2.39E-08	RXRA/NCOA1/BCL2/MAPK8/HMOX1/VCAM1/AKR1C3/MAPK3/MAPK1/PPARA/SRE	17

		stimulus						BF1/FOS/CDKN1A/TP53/HSPA5/COL1A1/NFE2L2	
BP	GO:1903039	positive regulation of leukocyte cell-cell adhesion	17/210	245/18800	2.41E-09	5.12E-08	2.39E-08	DPP4/RELA/IL4/AKT1/SELE/VCAM1/ALOX5/HAS2/BAD/CAV1/IL1B/CCL2/IL2RA/IFNG/IL1A/IGF2/CD40LG	17
BP	GO:0008625	extrinsic apoptotic signaling pathway via death domain receptors	11/210	85/18800	2.69E-09	5.69E-08	2.66E-08	GSK3B/BCL2/BAX/HMOX1/ICAM1/BAD/BCL2L1/CASP8/RAF1/NOS3/SERPINE1	11
BP	GO:0030193	regulation of blood coagulation	10/210	66/18800	2.99E-09	6.22E-08	2.91E-08	F7/ABAT/PLAU/CAV1/F3/NOS3/PLAT/THBD/SERPINE1/NFE2L2	10
BP	GO:0038034	signal transduction in absence of ligand	10/210	66/18800	2.99E-09	6.22E-08	2.91E-08	GSK3B/IL4/AKT1/BCL2/BAX/BAD/BCL2L1/IL1B/IL1A/ERBB3	10
BP	GO:0097192	extrinsic apoptotic signaling pathway in absence of ligand	10/210	66/18800	2.99E-09	6.22E-08	2.91E-08	GSK3B/IL4/AKT1/BCL2/BAX/BAD/BCL2L1/IL1B/IL1A/ERBB3	10
BP	GO:0006805	xenobiotic	12/210	108/18800	3.00E-09	6.22E-08	2.91E-08	CYP3A4/CYP1A2/CYP1A1/NR1I2/C	12

		metabolic process						YP1B1/GSTP1/AHR/GSTM1/GSTM2/UGT1A1/POR/NQO1	
BP	GO:0019915	lipid storage	11/210	86/18800	3.06E-09	6.30E-08	2.95E-08	PPARG/PPARD/APOB/PPARA/SREBF1/CES1/SOAT1/NFKBIA/CAV1/IL1B/DGAT2	11
BP	GO:0061041	regulation of wound healing	13/210	133/18800	3.20E-09	6.56E-08	3.07E-08	F7/ALOX5/ABAT/PLAU/CAV1/F3/DUOX2/NOS3/PLAT/THBD/SERPINE1/NFE2L2/CLDN4	13
BP	GO:0031099	regeneration	15/210	188/18800	3.25E-09	6.65E-08	3.11E-08	CCNA2/PPARD/F7/JUN/BCL2/CDK1/HMOX1/PPP3CA/UGT1A1/CCND1/CDKN1A/MMP2/MYC/GJA1/SPP1	15
BP	GO:0090257	regulation of muscle system process	17/210	250/18800	3.27E-09	6.65E-08	3.11E-08	PTGS2/CHRM3/SCN5A/ADRA1A/CHRM2/ADRB2/ADRA1B/PPARG/PPP3CA/GSTM2/SOD1/PPARA/ABAT/PRKCA/CAV1/NOS3/PARP1	17
BP	GO:1903131	mononuclear cell differentiation	22/210	433/18800	3.60E-09	7.27E-08	3.40E-08	PPARG/JUN/IL4/BCL2/BAX/VCAM1/FASN/BAD/SOD1/STAT3/VEGFA/IL6R/TP53/ERBB2/MYC/IL1B/IL2RA/IFNG/IL1A/RUNX2/CD40LG/IRF1	22
BP	GO:0031281	positive regulation of cyclase activity	9/210	50/18800	3.84E-09	7.74E-08	3.62E-08	ADRB2/NOS2/MAPK14/DRD1/ADRB1/MAPK8/MAPK3/RAF1/NOS3	9
BP	GO:0006633	fatty acid	14/210	162/18800	3.94E-09	7.89E-08	3.69E-08	PTGS2/PTGS1/CYP3A4/CYP1A2/CY	14

		biosynthetic process						P1A1/ALOX5/GSTP1/GSTM1/GSTM2/AKR1C3/FASN/EIF6/ACACA/IL1B	
BP	GO:1900046	regulation of hemostasis	10/210	68/18800	4.04E-09	8.05E-08	3.77E-08	F7/ABAT/PLAU/CAV1/F3/NOS3/PLAT/THBD/SERPINE1/NFE2L2	10
BP	GO:0030728	ovulation	7/210	23/18800	4.14E-09	8.21E-08	3.84E-08	PTGS2/PGR/TNFAIP6/MMP2/MYC/NOS3/PLAT	7
BP	GO:0007346	regulation of mitotic cell cycle	23/210	478/18800	4.37E-09	8.63E-08	4.04E-08	CHEK1/CDK2/AKT1/BCL2/CDK1/CYP1A1/CCND1/RB1/CDK4/EGFR/CDKN1A/EGF/TP53/PRKCA/MYC/IL1B/CCL2/CCNB1/IL1A/CHEK2/HSF1/E2F1/IGF2	23
BP	GO:0051591	response to cAMP	11/210	89/18800	4.43E-09	8.71E-08	4.08E-08	SLC6A3/STAT1/CYP1B1/AHR/SREBF1/FOS/HSPA5/DUOX2/PLAT/THBD/COL1A1	11
BP	GO:0033344	cholesterol efflux	10/210	69/18800	4.68E-09	9.15E-08	4.28E-08	RXRA/PPARG/APOB/SOAT2/CES1/SOAT1/EGF/NFKBIA/CAV1/PON1	10
BP	GO:0010632	regulation of epithelial cell migration	18/210	290/18800	4.71E-09	9.18E-08	4.30E-08	PTGS2/PPARG/KDR/JUN/IL4/ATP5F1B/AKT1/HMOX1/HAS2/VEGFA/MMP9/EGF/PRKCA/HIF1A/NOS3/HSPB1/IFNG/NFE2L2	18
BP	GO:0050920	regulation of chemotaxis	16/210	225/18800	5.09E-09	9.87E-08	4.62E-08	DPP4/KDR/F7/IL4/TNFAIP6/MAPK3/MAPK1/CYP19A1/VEGFA/IL6R/F3/CCL2/CXCL8/HSPB1/SERPINE1/C	16

								XCL10	
BP	GO:0042698	ovulation cycle	10/210	70/18800	5.40E-09	1.04E-07	4.85E-08	PGR/OPRM1/ESR1/TNFAIP6/CASP3/CYP1B1/HAS2/MMP2/HSPA5/NOS3	10
BP	GO:1903034	regulation of response to wounding	14/210	166/18800	5.40E-09	1.04E-07	4.85E-08	F7/ALOX5/ABAT/PLAU/CAV1/F3/DUOX2/NOS3/PLAT/THBD/SERPINE1/NFE2L2/CLDN4/SPP1	14
BP	GO:0009743	response to carbohydrate	16/210	226/18800	5.42E-09	1.04E-07	4.85E-08	PTGS2/PPARD/CASP3/ICAM1/PPP3CA/BAD/CAT/APOB/SREBF1/RAF1/HIF1A/MYC/IL1B/PRKCB/IL1A/NQO1	16
BP	GO:0051222	positive regulation of protein transport	18/210	293/18800	5.54E-09	1.05E-07	4.94E-08	PTGS2/CHRM1/ACHE/HSP90AB1/PPARG/MAPK14/GSK3B/PPARD/MAPK8/CDK1/BAD/ABAT/HIF1A/ERBB2/IL1B/IFNG/IL1A/NPEPPS	18
BP	GO:1901522	positive regulation of transcription from RNA polymerase II promoter involved in cellular response to chemica	7/210	24/18800	5.78E-09	1.10E-07	5.13E-08	RELA/JUN/VEGFA/TP53/HIF1A/NFE2L2/RUNX2	7

		l stimulus							
BP	GO:0070661	leukocyte proliferation	19/210	330/18800	5.94E-09	1.12E-07	5.25E-08	IL4/BCL2/BAX/CASP3/VCAM1/GSTP1/AHR/MAPK3/MAPK1/CDKN1A/TP53/ERBB2/GJA1/IL1B/IL2RA/IL1A/IGF2/CD40LG/IRF1	19
BP	GO:0033559	unsaturated fatty acid metabolic process	12/210	115/18800	6.20E-09	1.15E-07	5.39E-08	PTGS2/PTGS1/AKR1B1/CYP1A2/CYP1A1/CYP1B1/ALOX5/GSTP1/GSTM1/GSTM2/AKR1C3/IL1B	12
BP	GO:0008210	estrogen metabolic process	8/210	37/18800	6.20E-09	1.15E-07	5.39E-08	HSD3B1/CYP3A4/CYP1A2/CYP1A1/CYP1B1/CYP19A1/UGT1A1/SULT1E1	8
BP	GO:0050818	regulation of coagulation	10/210	71/18800	6.23E-09	1.15E-07	5.39E-08	F7/ABAT/PLAU/CAV1/F3/NOS3/PLAT/THBD/SERPINE1/NFE2L2	10
BP	GO:0051881	regulation of mitochondrial membrane potential	10/210	71/18800	6.23E-09	1.15E-07	5.39E-08	KDR/OPRD1/AKT1/BCL2/BAX/BAD/SOD1/BCL2L1/MYC/PARP1	10
BP	GO:0051235	maintenance of location	19/210	331/18800	6.24E-09	1.15E-07	5.39E-08	PPARG/DRD1/PPARD/AKT1/BAX/GSTM2/APOB/PPARA/SREBF1/CES1/SOAT1/NFKBIA/HSPA5/CAV1/IL1B/CXCL11/CXCL10/HK2/DGAT2	19
BP	GO:0097529	myeloid leukocyte	16/210	229/18800	6.55E-09	1.20E-07	5.64E-08	DPP4/IL4/TNFAIP6/MAPK3/MAPK1/CYP19A1/VEGF	16

		migration						A/IL6R/IL1B/CCL2/CXCL8/SERPINE1/IL1A/CXCL11/CXCL2/CXCL10	
BP	GO:1903706	regulation of hemopoiesis	20/210	371/18800	7.11E-09	1.30E-07	6.09E-08	MAPK14/IL4/TNFAIP6/STAT1/BAD/SOD1/STAT3/EIF6/RB1/FOS/NFKBIA/CASP8/PRKCA/HIF1A/ERBB2/MYC/IL2RA/IFNG/HSF1/IRF1	20
BP	GO:0042178	xenobiotic catabolic process	7/210	25/18800	7.96E-09	1.45E-07	6.77E-08	CYP3A4/CYP1A2/CYP1A1/NR1I2/GSTM1/GSTM2/UGT1A1	7
BP	GO:0010506	regulation of autophagy	19/210	336/18800	7.97E-09	1.45E-07	6.77E-08	ADRA1A/ADRB2/KDR/GSK3B/IL4/AKT1/BCL2/CASP3/MAPK8/HMOX1/MAPK3/BAD/SREBF1/STAT3/IL10RA/TP53/HIF1A/HSPB1/IFNG	19
BP	GO:0071383	cellular response to steroid hormone stimulus	15/210	203/18800	9.28E-09	1.68E-07	7.85E-08	PGR/AR/RXRA/NR3C2/ESR1/ESR2/PPARD/CYP1B1/AKR1C3/UGT1A1/PPARA/RXRB/CASP9/PLAT/PARP1	15
BP	GO:0032091	negative regulation of protein binding	11/210	96/18800	1.00E-08	1.80E-07	8.42E-08	ADRB2/GSK3B/AKT1/BAX/MAPK8/SLPI/MAPK3/PPARA/CDKN1A/CAV1/MAP2	11
BP	GO:0032869	cellular response to insulin stimulus	15/210	205/18800	1.06E-08	1.90E-07	8.89E-08	PPARG/GSK3B/AKT1/STAT1/SLC2A4/INSRR/SREBF1/GOT1/RB1/CDK4/MYC/IL1B/PRKCB/PARP1/IGF2	15
BP	GO:00068	nitric	10/210	75/18	1.08E-	1.92E-07	8.98E-08	PTGS2/HSP90AB1	10

	09	oxide biosynthetic process		800	08			/NOS2/AKT1/CYP1B1/CAV1/IL1B/NOS3/IFNG/NQO1	
BP	GO:0051101	regulation of DNA binding	12/210	121/18800	1.11E-08	1.96E-07	9.19E-08	PPARG/JUN/MAPK8/HMOX1/RB1/MMP9/EGF/NFKBIA/MYC/IFNG/PARP1/E2F1	12
BP	GO:0071482	cellular response to light stimulus	12/210	121/18800	1.11E-08	1.96E-07	9.19E-08	PTGS2/CHEK1/BAX/MMP1/MMP3/CDKN1A/CASP9/MMP2/MMP9/TP53/MYC/PARP1	12
BP	GO:0043270	positive regulation of ion transport	17/210	273/18800	1.22E-08	2.16E-07	1.01E-07	CHRM1/SCN5A/ADRB2/SLC6A4/DRD1/AKT1/BAX/GSTM2/ABAT/KCNH2/CAV1/IL1B/CCL2/IFNG/IL1A/CXCL11/CXCL10	17
BP	GO:0051051	negative regulation of transport	22/210	464/18800	1.26E-08	2.22E-07	1.04E-07	PTGS2/OPRM1/MAOB/AKT1/BCL2/HMOX1/PPP3CA/GSTM2/HMGCR/SREBF1/ABAT/KCNH2/MMP9/EGF/CAV1/MYC/GJA1/IL1B/PTGER3/PRKCB/NOS3/ERBB3	22
BP	GO:1904951	positive regulation of establishment of protein localization	18/210	309/18800	1.27E-08	2.22E-07	1.04E-07	PTGS2/CHRM1/ACHE/HSP90AB1/PPARG/MAPK14/GSK3B/PPARD/MAPK8/CDK1/BAD/ABAT/HIF1A/ERBB2/IL1B/IFNG/IL1A/NPEPPS	18
BP	GO:0007566	embryo implantation	9/210	57/18800	1.29E-08	2.24E-07	1.05E-07	PTGS2/PPARD/SOD1/VEGFA/MMP2/MMP9/GJA1/IL1B/SPP1	9
BP	GO:00456	regulatio	15/210	208/1	1.29E-	2.24E-07	1.05E-07	MAPK14/IL4/TNF	15

	37	n of myeloid cell differentiation		8800	08			AIP6/STAT1/STAT3/EIF6/RB1/FOS/NFKBIA/CASP8/PRKCA/HIF1A/MYC/IFNG/HSF1	
BP	GO:0034349	glial cell apoptotic process	6/210	16/18800	1.32E-08	2.28E-07	1.07E-07	CASP3/RB1/CASP9/TP53/PRKCA/CCL2	6
BP	GO:0001649	osteoblast differentiation	16/210	241/18800	1.36E-08	2.33E-07	1.09E-07	ACHE/PPARG/MAPK14/GSK3B/ATP5F1B/AKT1/TNFAIP6/NR1I3/FASN/CAT/IL6R/GJA1/COL1A1/SPP1/RUNX2/IGF2	16
BP	GO:1901215	negative regulation of neuron death	15/210	209/18800	1.38E-08	2.36E-07	1.10E-07	HSP90AB1/GSK3B/JUN/AKT1/BCL2/BAX/HMOX1/SOD1/PPARA/BCL2L1/HIF1A/CCL2/HSF1/ERBB3/RASA1	15
BP	GO:0007188	adenylate cyclase-modulating G protein-coupled receptor signaling pathway	16/210	244/18800	1.61E-08	2.75E-07	1.29E-07	CHRM3/CHRM1/ADRA1A/CHRM2/ADRB2/OPRM1/ADRA1B/ADRA1D/DRD1/CHRM5/OPRD1/ADRB1/PRKCA/PTGER3/CXCL11/CXCL10	16
BP	GO:0045834	positive regulation of lipid metabolic process	13/210	153/18800	1.76E-08	2.99E-07	1.40E-07	PTGS2/PPARG/PPARD/AKT1/LDLR/PPARA/SREBF1/CES1/POR/IL1B/IFNG/IL1A/DGAT2	13
BP	GO:0031669	cellular response to	15/210	213/18800	1.78E-08	3.01E-07	1.41E-07	RXRA/NCOA1/BCL2/MAPK8/HMOX1/AKR1C3/MA	15

		nutrient levels						PK3/MAPK1/PPARA/SREBF1/CDKN1A/TP53/HSPA5/COL1A1/NFE2L2	
BP	GO:0046394	carboxylic acid biosynthetic process	18/210	316/18800	1.79E-08	3.01E-07	1.41E-07	PTGS2/PTGS1/LTA4H/CYP3A4/CYP1A2/CYP1A1/ALOX5/GSTP1/GSTM1/GSTM2/AKR1C3/FASN/GOT1/ABAT/CES1/EIF6/ACACA/IL1B	18
BP	GO:0009615	response to virus	20/210	392/18800	1.79E-08	3.01E-07	1.41E-07	CHRM2/MAPK14/RELA/IL4/IKBKB/BCL2/BAX/STAT1/CYP1A1/APOB/BCL2L1/ODC1/HIF1A/IL1B/DUOX2/HSPB1/IFNG/CXCL10/CHUK/IRF1	20
BP	GO:0001819	positive regulation of cytokine production	22/210	475/18800	1.92E-08	3.21E-07	1.50E-07	PTGS2/NOS2/MAPK14/RELA/IL4/STAT1/HMOX1/CYP1B1/SOD1/STAT3/IL6R/CASP8/HIF1A/F3/IL1B/HSPB1/SERPINE1/IFNG/IL1A/CHUK/CD40LG/IRF1	22
BP	GO:0016053	organic acid biosynthetic process	18/210	318/18800	1.98E-08	3.29E-07	1.54E-07	PTGS2/PTGS1/LTA4H/CYP3A4/CYP1A2/CYP1A1/ALOX5/GSTP1/GSTM1/GSTM2/AKR1C3/FASN/GOT1/ABAT/CES1/EIF6/ACACA/IL1B	18
BP	GO:0033674	positive regulation of kinase activity	22/210	476/18800	2.00E-08	3.31E-07	1.55E-07	ADRA1A/ADRB2/HSP90AB1/KDR/NCF1/IL4/AKT1/INSRR/BAD/CCND1/EGFR/VEGFA/CDKN1A/EGF/IL6R/ERBB2/IL1B/C	22

								CNB1/IFNG/IGF2/CD40LG/ERBB3	
BP	GO:0072330	monocarboxylic acid biosynthetic process	15/210	215/18800	2.01E-08	3.33E-07	1.56E-07	PTGS2/PTGS1/CYP3A4/CYP1A2/CYP1A1/ALOX5/GSTP1/GSTM1/GSTM2/AKR1C3/FASN/CES1/EIF6/ACACA/IL1B	15
BP	GO:0015850	organic hydroxy compound transport	16/210	248/18800	2.03E-08	3.34E-07	1.56E-07	SLC6A2/MAOB/SLC6A4/DRD1/CHRM5/SLC6A3/LDLR/MTTP/APOB/CYP19A1/SOAT2/ABAT/CES1/CAV1/MYC/SPP1	16
BP	GO:0001101	response to acid chemical	12/210	129/18800	2.29E-08	3.76E-07	1.76E-07	F7/CASP3/BAD/EGFR/VEGFA/BCL2L1/MMP2/MYC/COL1A1/NQO1/COL3A1/HSF1	12
BP	GO:0001667	ameboidal-type cell migration	22/210	480/18800	2.32E-08	3.78E-07	1.77E-07	PTGS2/DPP4/PPARG/KDR/PPARD/JUN/IL4/ATP5F1B/AKT1/HMOX1/CYP1B1/HAS2/VEGFA/MMP9/EGF/PRKCA/HIF1A/GJA1/NOS3/HSPB1/IFNG/NFE2L2	22
BP	GO:0019218	regulation of steroid metabolic process	11/210	104/18800	2.34E-08	3.81E-07	1.78E-07	AKR1C3/LDLR/SOD1/APOB/UGT1A1/SREBF1/CES1/POR/IFNG/IL1A/DGAT2	11
BP	GO:1903409	reactive oxygen species biosynthetic process	9/210	61/18800	2.39E-08	3.88E-07	1.81E-07	MAOB/NCF1/CYP1A2/CYP1A1/ALOX5/SOD1/PPARA/DUOX2/MPO	9
BP	GO:0042759	long-chain fatty	7/210	29/18800	2.49E-08	4.02E-07	1.88E-07	CYP3A4/CYP1A2/CYP1A1/ALOX5/	7

		acid biosynthetic process						GSTP1/GSTM1/GSTM2	
BP	GO:0010595	positive regulation of endothelial cell migration	12/210	131/18800	2.73E-08	4.39E-07	2.05E-07	PTGS2/KDR/ATP5F1B/AKT1/HMOX1/VEGFA/EGF/PRKCA/HIF1A/NOS3/HSPB1/NFE2L2	12
BP	GO:0031663	lipopolysaccharide-mediated signaling pathway	9/210	62/18800	2.77E-08	4.44E-07	2.08E-07	MAPK14/AKT1/MAPK3/MAPK1/NFKBIA/PRKCA/IL1B/CCL2/NOS3	9
BP	GO:0070663	regulation of leukocyte proliferation	16/210	254/18800	2.84E-08	4.53E-07	2.12E-07	IL4/BCL2/CASP3/VCAM1/GSTP1/AHR/MAPK3/MAPK1/CDKN1A/ERBB2/IL1B/IL2RA/IL1A/IGF2/CD40LG/IRF1	16
BP	GO:0007596	blood coagulation	15/210	221/18800	2.91E-08	4.63E-07	2.17E-07	MAPK14/F7/ABAT/PLAU/PRKCA/CAV1/F3/NOS3/HSPB1/PLAT/THBD/SERPINE1/NFE2L2/COL3A1/CD40LG	15
BP	GO:0010565	regulation of cellular ketone metabolic process	12/210	132/18800	2.97E-08	4.71E-07	2.20E-07	PTGS2/PPARG/PPARD/AKT1/AKR1C3/PPARA/SREBF1/CES1/EIF6/CAV1/IL1B/DGAT2	12
BP	GO:0044843	cell cycle G1/S phase transitio	16/210	255/18800	3.00E-08	4.74E-07	2.22E-07	CCNA2/CDK2/AKT1/BCL2/CYP1A1/PPP3CA/CCND1/RB1/CDK4/EGFR/CDKN1A/TP53/M	16

		n						YC/CCL2/CHEK2/E2F1	
BP	GO:0010876	lipid localization	21/210	446/18800	3.11E-08	4.89E-07	2.29E-07	NOS2/PPARG/PPARD/AKT1/LDLR/MTTP/APOB/CYP19A1/PPARA/SREBF1/ABCC1/SOAT2/CES1/SOAT1/NFKBIA/CAV1/IL1B/IL1A/ABCG2/SPP1/DGAT2	21
BP	GO:0022409	positive regulation of cell-cell adhesion	17/210	291/18800	3.14E-08	4.91E-07	2.30E-07	DPP4/RELA/IL4/AKT1/SELE/VCAM1/ALOX5/HAS2/BAD/CAV1/IL1B/CCL2/IL2RA/IFNG/IL1A/IGF2/CD40LG	17
BP	GO:0008631	intrinsic apoptotic signaling pathway in response to oxidative stress	8/210	45/18800	3.21E-08	5.00E-07	2.34E-07	AKT1/BCL2/CYP1B1/SOD1/HIF1A/HSPB1/NFE2L2/PARP1	8
BP	GO:0071480	cellular response to gamma radiation	7/210	30/18800	3.21E-08	5.00E-07	2.34E-07	BCL2L1/CDKN1A/TP53/ELK1/HSPA5/CHEK2/HSF1	7
BP	GO:0045787	positive regulation of cell cycle	18/210	329/18800	3.33E-08	5.16E-07	2.41E-07	SLC6A4/CHEK1/AKT1/CDK1/CYP1A1/CCND1/RB1/CDK4/EGFR/EGF/PRKCA/MYC/IL1B/CCNB1/IL1A/CHEK2/HSF1/IGF2	18
BP	GO:0043410	positive regulation of	22/210	491/18800	3.46E-08	5.35E-07	2.50E-07	AR/ADRA1A/ADRB2/OPRM1/ADRA1B/ADRA1D/KD	22

		MAPK cascade						R/NCF1/JUN/ICAM1/MAPK3/SOD1/EGFR/VEGFA/EGF/RAF1/PRKCA/ERBB2/IL1B/CCL2/IL1A/IGF2	
BP	GO:0045862	positive regulation of proteolysis	19/210	369/18800	3.59E-08	5.52E-07	2.58E-07	PPARG/GSK3B/AKT1/BAX/BAD/STAT3/CASP9/EGF/CASP8/CAV1/MYC/F3/IL1B/IFNG/NFE2L2/CLDN4/HSF1/CTSD/PCOLCE	19
BP	GO:0046889	positive regulation of lipid biosynthetic process	10/210	85/18800	3.68E-08	5.64E-07	2.64E-07	PTGS2/AKT1/LDLR/PPARA/SREBF1/POR/IL1B/IFNG/IL1A/DGAT2	10
BP	GO:0000082	G1/S transition of mitotic cell cycle	15/210	225/18800	3.69E-08	5.64E-07	2.64E-07	CDK2/AKT1/BCL2/CYP1A1/PPP3CA/CCND1/RB1/CDK4/EGFR/CDKN1A/TP53/MYC/CCL2/CHEK2/E2F1	15
BP	GO:0009408	response to heat	11/210	109/18800	3.83E-08	5.83E-07	2.73E-07	PTGS2/HSP90AB1/GSK3B/AKT1/HMOX1/SOD1/NOS3/HSPB1/IL1A/HSF1/CXCL10	11
BP	GO:0050817	coagulation	15/210	226/18800	3.92E-08	5.94E-07	2.78E-07	MAPK14/F7/ABAT/PLAU/PRKCA/CAV1/F3/NOS3/HSPB1/PLAT/THBD/SERPINE1/NFE2L2/COL3A1/CD40LG	15
BP	GO:0010039	response to iron ion	7/210	31/18800	4.11E-08	6.19E-07	2.90E-07	SLC6A3/BCL2/HMOX1/CYP1A1/ABAT/CCND1/HIF1A	7
BP	GO:19020	negative	7/210	31/18	4.11E-	6.19E-07	2.90E-07	GSK3B/HMOX1/I	7

	42	regulation of extrinsic apoptotic signaling pathway via death domain receptors		800	08			CAM1/BCL2L1/RAF1/NOS3/SERPINE1	
BP	GO:1900542	regulation of purine nucleotide metabolic process	10/210	86/18800	4.13E-08	6.20E-07	2.90E-07	NOS2/IL4/PPARA/STAT3/EIF6/HIF1A/MYC/NOS3/IFNG/PARP1	10
BP	GO:0007599	hemostasis	15/210	227/18800	4.15E-08	6.21E-07	2.91E-07	MAPK14/F7/ABAT/PLAU/PRKCA/CAV1/F3/NOS3/HSPB1/PLAT/THBD/SERPINE1/NFE2L2/COL3A1/CD40LG	15
BP	GO:0060749	mammary gland alveolus development	6/210	19/18800	4.35E-08	6.45E-07	3.02E-07	AR/ESR1/CCND1/VEGFA/EGF/HIF1A	6
BP	GO:0061377	mammary gland lobule development	6/210	19/18800	4.35E-08	6.45E-07	3.02E-07	AR/ESR1/CCND1/VEGFA/EGF/HIF1A	6
BP	GO:0043393	regulation of protein binding	14/210	196/18800	4.51E-08	6.67E-07	3.12E-07	ADRB2/HSP90AB1/GSK3B/AKT1/BAX/MAPK8/SLPI/MAPK3/PPARA/CDKN1A/MMP9/CAV1/HSF1/MAP2	14

BP	GO:0001676	long-chain fatty acid metabolic process	11/210	111/18800	4.64E-08	6.81E-07	3.19E-07	PTGS2/PTGS1/CYP3A4/CYP1A2/CYP1A1/CYP1B1/ALOX5/GSTP1/GSTM1/GSTM2/AKR1C3	11
BP	GO:0001938	positive regulation of endothelial cell proliferation	11/210	111/18800	4.64E-08	6.81E-07	3.19E-07	KDR/JUN/AKT1/HMOX1/STAT3/VEGFA/EGF/PRKCA/HIF1A/F3/IGF2	11
BP	GO:0006140	regulation of nucleotide metabolic process	10/210	88/18800	5.16E-08	7.56E-07	3.54E-07	NOS2/IL4/PPARA/STAT3/EIF6/HIF1A/MYC/NOS3/IFNG/PARP1	10
BP	GO:0002526	acute inflammatory response	11/210	113/18800	5.59E-08	8.15E-07	3.81E-07	PTGS2/OPRM1/IL4/VCAM1/GSTP1/UGT1A1/IL6R/F3/IL1B/PTGER3/IL1A	11
BP	GO:0050921	positive regulation of chemotaxis	12/210	140/18800	5.74E-08	8.35E-07	3.91E-07	KDR/F7/IL4/MAPK3/MAPK1/VEGFA/IL6R/F3/CXCL8/HSPB1/SERPINE1/CXCL10	12
BP	GO:0070555	response to interleukin-1	12/210	141/18800	6.21E-08	9.00E-07	4.21E-07	RELA/IKBKB/SELE/HAS2/MAPK3/MMP2/PRKCA/HIF1A/MYC/IL1B/CCL2/CXCL8	12
BP	GO:0030225	macrophage differentiation	8/210	49/18800	6.46E-08	9.33E-07	4.36E-07	RB1/VEGFA/MMP9/CASP8/PRKCA/IFNG/PARP1/HSF1	8
BP	GO:0052548	regulation of endopeptidase activity	20/210	426/18800	7.07E-08	1.02E-06	4.76E-07	PTGS2/DPEP1/PPARG/AKT1/BAX/SLPI/BAD/STAT3/VEGFA/CASP9/MMP9/POR/CASP	20

								8/RAF1/MYC/F3/BIRC5/SERPINE1/HSF1/CTSD	
BP	GO:0014909	smooth muscle cell migration	10/210	91/18800	7.14E-08	1.02E-06	4.78E-07	PPARD/BCL2/CYP1B1/HAS2/PLAU/MYC/PLAT/SERPINE1/NFE2L2/IGFBP3	10
BP	GO:0030336	negative regulation of cell migration	18/210	346/18800	7.14E-08	1.02E-06	4.78E-07	DPP4/DPEP1/PPARG/PPARD/IL4/AKT1/BCL2/TNFAIP6/HMOX1/CYP1B1/CYP19A1/STAT3/GJA1/CCL2/SERPINE1/NFE2L2/COL3A1/IGFBP3	18
BP	GO:0019217	regulation of fatty acid metabolic process	10/210	92/18800	7.93E-08	1.13E-06	5.29E-07	PTGS2/PPARG/PPARD/AKT1/PPARA/SREBF1/EIF6/CAV1/IL1B/DGAT2	10
BP	GO:0048771	tissue remodeling	13/210	174/18800	8.15E-08	1.16E-06	5.42E-07	ADRB2/PPARG/BAX/PPP3CA/MMP2/TP53/PRKCA/HIF1A/CAV1/GJA1/NOS3/IL1A/SPP1	13
BP	GO:0071260	cellular response to mechanical stimulus	9/210	70/18800	8.23E-08	1.16E-06	5.45E-07	PTGS2/CHEK1/MAPK8/MAPK3/BAD/CASP8/IL1B/COL1A1/IRF1	9
BP	GO:0010878	cholesterol storage	6/210	21/18800	8.53E-08	1.20E-06	5.63E-07	PPARG/PPARD/APOB/PPARA/CES1/SOAT1	6
BP	GO:0035265	organ growth	13/210	175/18800	8.72E-08	1.23E-06	5.74E-07	AR/ADRA1A/ESR1/SLC6A4/MAPK14/AKT1/BCL2/CDK1/SOD1/CYP19A1/PPARA/POR/IGF2	13
BP	GO:19040	negative	8/210	52/18	1.05E-	1.47E-06	6.87E-07	KDR/IL4/HMOX1/	8

	36	regulation of epithelial cell apoptotic process		800	07			ICAM1/PPARA/RB1/SERPINE1/NFE2L2	
BP	GO:0006801	superoxide metabolic process	9/210	72/18800	1.06E-07	1.48E-06	6.90E-07	NOS2/NCF1/GSTP1/SOD1/DUOX2/NOS3/MPO/NFE2L2/NQO1	9
BP	GO:0010821	regulation of mitochondrion organization	12/210	148/18800	1.06E-07	1.48E-06	6.92E-07	PPARG/KDR/GSK3B/AKT1/BAX/MAPK8/BAD/SREBF1/BCL2L1/MMP9/TP53/HIF1A	12
BP	GO:0048661	positive regulation of smooth muscle cell proliferation	10/210	95/18800	1.08E-07	1.50E-06	7.01E-07	PTGS2/JUN/AKT1/STAT1/HMOX1/MMP2/MMP9/IL6R/MYC/GJA1	10
BP	GO:0045860	positive regulation of protein kinase activity	19/210	396/18800	1.09E-07	1.51E-06	7.05E-07	ADRA1A/ADRB2/HSP90AB1/NCF1/IL4/AKT1/CCND1/EGFR/VEGFA/CDKN1A/EGF/IL6R/ERBB2/IL1B/CCNB1/IFNG/IGF2/CD40LG/ERBB3	19
BP	GO:0042180	cellular ketone metabolic process	14/210	211/18800	1.13E-07	1.56E-06	7.30E-07	PTGS2/PPARG/PPARD/AKR1B1/AKT1/AKR1C3/CYP19A1/PPARA/SREBF1/CES1/EIF6/CAV1/IL1B/DGAT2	14
BP	GO:0060249	anatomical structure homeost	17/210	319/18800	1.19E-07	1.63E-06	7.63E-07	PTGS2/ADRB2/SLC6A2/KDR/BCL2/BAX/SOD1/RB1/VEGFA/PRKCA/H	17

		asis						IF1A/ACACA/GJA1/NOS3/HSPB1/COL3A1/SPP1	
BP	GO:0045936	negative regulation of phosphate metabolic process	20/210	440/18800	1.19E-07	1.64E-06	7.65E-07	PPARG/GSK3B/JUN/IKBKB/AKT1/BAX/CASP3/GSTP1/HMGCR/PPARA/STAT3/RB1/CDKN1A/TP53/CAV1/IL1B/HSPB1/IFNG/PARP1/IGFBP3	20
BP	GO:0043535	regulation of blood vessel endothelial cell migration	12/210	150/18800	1.23E-07	1.68E-06	7.87E-07	PTGS2/PPARG/KDR/ATP5F1B/AKT1/HMOX1/VEGFA/PRKCA/HIF1A/NOS3/HSPB1/NFE2L2	12
BP	GO:0010563	negative regulation of phosphorus metabolic process	20/210	441/18800	1.24E-07	1.69E-06	7.89E-07	PPARG/GSK3B/JUN/IKBKB/AKT1/BAX/CASP3/GSTP1/HMGCR/PPARA/STAT3/RB1/CDKN1A/TP53/CAV1/IL1B/HSPB1/IFNG/PARP1/IGFBP3	20
BP	GO:2000146	negative regulation of cell motility	18/210	361/18800	1.35E-07	1.83E-06	8.57E-07	DPP4/DPEP1/PPARG/PPARD/IL4/AKT1/BCL2/TNFAIP6/HMOX1/CYP1B1/CYP19A1/STAT3/GJA1/CCL2/SERPINE1/NFE2L2/COL3A1/IGFBP3	18
BP	GO:0050728	negative regulation of inflammatory response	13/210	182/18800	1.38E-07	1.87E-06	8.74E-07	PPARG/MAPK14/PPARD/IL4/TNFAIP6/ALOX5/GSTP1/LDLR/SOD1/CYP19A1/PPARA/RB1/IL2RA	13
BP	GO:00901	regulatio	7/210	37/18	1.52E-	2.05E-06	9.60E-07	LDLR/SOD1/APO	7

	81	n of cholesterol metabolic process		800	07			B/SREBF1/CES1/POR/DGAT2	
BP	GO:0042886	amide transport	16/210	287/18800	1.54E-07	2.06E-06	9.66E-07	NOS2/PPARD/ALOX5/PPP3CA/BAD/MTTP/SREBF1/ABCC1/ABAT/CA2/RAF1/HIF1A/GJA1/IL1B/IFNG/ABCG2	16
BP	GO:0045639	positive regulation of myeloid cell differentiation	10/210	99/18800	1.60E-07	2.15E-06	1.00E-06	MAPK14/STAT1/STAT3/RB1/FOS/CASP8/PRKCA/HIF1A/IFNG/HSF1	10
BP	GO:0050708	regulation of protein secretion	15/210	252/18800	1.63E-07	2.18E-06	1.02E-06	ACHE/OPRM1/NOS2/PPARG/PPARD/ALOX5/PPP3CA/BAD/HMGCR/SREBF1/ABAT/HIF1A/IL1B/IFNG/IL1A	15
BP	GO:0097530	granulocyte migration	12/210	154/18800	1.64E-07	2.18E-06	1.02E-06	DPP4/IL4/TNFAIP6/MAPK3/MAPK1/IL1B/CCL2/CXCL8/IL1A/CXCL11/CXCL2/CXCL10	12
BP	GO:0042743	hydrogen peroxide metabolic process	8/210	55/18800	1.65E-07	2.18E-06	1.02E-06	MAOB/CYP1A2/CYP1A1/SOD1/CAT/MMP3/DUOX2/MPO	8
BP	GO:0010742	macrophage derived foam cell different	7/210	38/18800	1.85E-07	2.44E-06	1.14E-06	PPARG/STAT1/APOB/PPARA/SOAT2/SOAT1/NFKBIA	7

		iation							
BP	GO:0098926	postsynaptic signal transduction	7/210	38/18800	1.85E-07	2.44E-06	1.14E-06	CHRM3/CHRM1/ACHE/CHRM2/OPRM1/CHRM5/RELA	7
BP	GO:0042063	gliogenesis	16/210	291/18800	1.85E-07	2.44E-06	1.14E-06	CHRM1/DRD1/AKT1/CDK1/MAPK3/MAPK1/LDLR/SOD1/STAT3/ERBB2/MYC/IL1B/CCL2/IFNG/E2F1/ERBB3	16
BP	GO:0051271	negative regulation of cellular component movement	18/210	369/18800	1.87E-07	2.45E-06	1.15E-06	DPP4/DPEP1/PPARG/PPARD/IL4/AKT1/BCL2/TNFAIP6/HMOX1/CYP1B1/CYP19A1/STAT3/GJA1/CCL2/SERPINE1/NFE2L2/COL3A1/IGFBP3	18
BP	GO:0045913	positive regulation of carbohydrate metabolic process	9/210	77/18800	1.91E-07	2.49E-06	1.17E-06	AKT1/HAS2/PPARA/EGF/HIF1A/MYC/IFNG/IGF2/DGAT2	9
BP	GO:0008630	intrinsic apoptotic signaling pathway in response to DNA damage	10/210	101/18800	1.94E-07	2.52E-06	1.18E-06	BCL2/BAX/HMOX1/BAD/BCL2L1/CDKN1A/CASP9/TP53/CHEK2/E2F1	10
BP	GO:0071621	granulocyte chemotaxis	11/210	128/18800	2.02E-07	2.62E-06	1.23E-06	DPP4/IL4/TNFAIP6/MAPK3/MAPK1/IL1B/CCL2/CXCL8/CXCL11/CXCL2/CXCL10	11

BP	GO:0035994	response to muscle stretch	6/210	24/18800	2.06E-07	2.66E-06	1.24E-06	MAPK14/RELA/JUN/FOS/NFKBIA/RAF1	6
BP	GO:0036003	positive regulation of transcription from RNA polymerase II promoter in response to stress	6/210	24/18800	2.06E-07	2.66E-06	1.24E-06	VEGFA/TP53/HIF1A/HSPA5/NFE2L2/HSF1	6
BP	GO:0031279	regulation of cyclase activity	9/210	78/18800	2.14E-07	2.75E-06	1.29E-06	ADRB2/NOS2/MAPK14/DRD1/ADRB1/MAPK8/MAPK3/RAF1/NOS3	9
BP	GO:0032768	regulation of monooxygenase activity	8/210	57/18800	2.19E-07	2.81E-06	1.32E-06	AKT1/EGFR/POR/HIF1A/CAV1/IL1B/IFNG/IL1A	8
BP	GO:0032570	response to progesterone	7/210	39/18800	2.23E-07	2.84E-06	1.33E-06	NCOA2/CYP1B1/BAD/SREBF1/FOS/CAV1/CLDN4	7
BP	GO:0090077	foam cell differentiation	7/210	39/18800	2.23E-07	2.84E-06	1.33E-06	PPARG/STAT1/APOB/PPARA/SOAT2/SOAT1/NFKBIA	7
BP	GO:0009755	hormone-mediated signaling pathway	13/210	190/18800	2.28E-07	2.90E-06	1.35E-06	PGR/AR/RXRA/NCOA1/NR3C2/ESR1/PPARG/ESR2/PPARD/PPARA/ADIPOR2/RXRB/PARP1	13
BP	GO:0006606	protein import into nucleus	12/210	159/18800	2.33E-07	2.95E-06	1.38E-06	PTGS2/HSP90AB1/MAPK14/DRD1/AKT1/CDK1/PPP3CA/STAT3/CDKN	12

								1A/TP53/NFKBIA/IFNG	
BP	GO:0046651	lymphocyte proliferation	16/210	296/18800	2.33E-07	2.95E-06	1.38E-06	IL4/BCL2/BAX/CASP3/VCAM1/AHR/CDKN1A/TP53/ERBB2/GJA1/IL1B/IL2RA/IL1A/IGF2/CD40LG/IRF1	16
BP	GO:0070371	ERK1 and ERK2 cascade	17/210	335/18800	2.38E-07	3.00E-06	1.40E-06	ADRA1A/OPRM1/KDR/JUN/CDK1/ICAM1/GSTP1/MAPK3/MAPK1/EGFR/EGF/PRKCA/ERBB2/MYC/IL1B/CCL2/IL1A	17
BP	GO:1903522	regulation of blood circulation	15/210	260/18800	2.45E-07	3.08E-06	1.44E-06	PTGS2/CHRM3/SCN5A/ADRA1A/CHRM2/ADRA1B/ADRA1D/ADRB1/GSTM2/SREBF1/KCNH2/MMP2/CAV1/GJA1/NOS3	15
BP	GO:2000045	regulation of G1/S transition of mitotic cell cycle	12/210	160/18800	2.49E-07	3.13E-06	1.46E-06	CDK2/AKT1/BCL2/CYP1A1/CCND1/RB1/EGFR/CDKN1A/TP53/CCL2/CHEK2/E2F1	12
BP	GO:0007200	phospholipase C-activating G protein-coupled receptor signaling pathway	10/210	104/18800	2.56E-07	3.18E-06	1.49E-06	CHRM1/ADRA1A/CHRM2/OPRM1/ADRA1B/ADRA1D/ESR1/DRD1/OPRD1/PTGER3	10
BP	GO:0014812	muscle cell migration	10/210	104/18800	2.56E-07	3.18E-06	1.49E-06	PPARD/BCL2/CYP1B1/HAS2/PLAU/MYC/PLAT/SERPINE1/NFE2L2/IG	10

								FBP3	
BP	GO:0010952	positive regulation of peptidase activity	13/210	192/18800	2.57E-07	3.19E-06	1.49E-06	PPARG/BAX/BAD/STAT3/CASP9/CASP8/CAV1/MYC/F3/CLDN4/HSF1/CTSD/PCOLCE	13
BP	GO:0071392	cellular response to estradiol stimulus	7/210	40/18800	2.68E-07	3.31E-06	1.55E-06	ESR1/CCNA2/ESR2/UGT1A1/EGFR/MMP2/HSF1	7
BP	GO:0060333	interferon-gamma-mediated signaling pathway	6/210	25/18800	2.68E-07	3.31E-06	1.55E-06	HSP90AB1/PPARG/STAT1/TP53/IFNG/IRF1	6
BP	GO:0032943	mononuclear cell proliferation	16/210	300/18800	2.80E-07	3.44E-06	1.61E-06	IL4/BCL2/BAX/CASP3/VCAM1/AHR/CDKN1A/TP53/ERBB2/GJA1/IL1B/IL2RA/IL1A/IGF2/CD40LG/IRF1	16
BP	GO:0019748	secondary metabolic process	8/210	59/18800	2.89E-07	3.53E-06	1.65E-06	AKR1B1/BCL2/CYP3A4/CYP1A2/CYP1A1/CYP1B1/AKR1C3/NFE2L2	8
BP	GO:2000351	regulation of endothelial cell apoptotic process	8/210	59/18800	2.89E-07	3.53E-06	1.65E-06	KDR/IL4/ICAM1/AKR1C3/CCL2/SERPINE1/NFE2L2/CD40LG	8
BP	GO:0051170	import into nucleus	12/210	163/18800	3.05E-07	3.72E-06	1.74E-06	PTGS2/HSP90AB1/MAPK14/DRD1/AKT1/CDK1/PPP3CA/STAT3/CDKN1A/TP53/NFKBIA/IFNG	12
BP	GO:00508	regulatio	17/210	342/1	3.18E-	3.87E-06	1.81E-06	DPP4/IL4/AKT1/C	17

	63	n of T cell activation		8800	07			ASP3/VCAM1/BAD/SOD1/ERBB2/CAV1/IL1B/CCL2/IL2RA/IFNG/IL1A/IGF2/CD40LG/IRF1	
BP	GO:0009299	mRNA transcription	7/210	41/18800	3.20E-07	3.87E-06	1.81E-06	RXRA/NCOA1/PPARG/SREBF1/STAT3/TP53/HSF1	7
BP	GO:0033574	response to testosterone	7/210	41/18800	3.20E-07	3.87E-06	1.81E-06	AR/BAD/ELK1/MYC/NQO1/HSF1/SPP1	7
BP	GO:0008637	apoptotic mitochondrial changes	10/210	107/18800	3.34E-07	4.02E-06	1.88E-06	GSK3B/AKT1/BCL2/BAX/MAPK8/BAD/BCL2L1/MMP9/TP53/HK2	10
BP	GO:0033138	positive regulation of peptidyl-serine phosphorylation	10/210	107/18800	3.34E-07	4.02E-06	1.88E-06	PTGS2/HSP90AB1/OPRD1/AKT1/BCL2/EGFR/VEGFA/RAF1/CAV1/IFNG	10
BP	GO:0046883	regulation of hormone secretion	14/210	231/18800	3.43E-07	4.12E-06	1.93E-06	NOS2/PPARG/PPARD/ALOX5/PPP3CA/BAD/CYP19A1/SREBF1/ABAT/HIF1A/GJA1/IL1B/IFNG/SPP1	14
BP	GO:1904385	cellular response to angiotensin	6/210	26/18800	3.46E-07	4.13E-06	1.93E-06	RELA/CA2/CAV1/MYC/NFE2L2/HSF1	6
BP	GO:0007162	negative regulation of cell adhesion	16/210	305/18800	3.50E-07	4.17E-06	1.95E-06	IL4/ATP5F1B/AKT1/CASP3/CYP1B1/PPARA/ABAT/VEGFA/MMP2/ERBB2/IL2RA/SERPINE1/COL1A1/IRF1/ERBB3/RASA1	16

BP	GO:0014910	regulation of smooth muscle cell migration	9/210	83/18800	3.67E-07	4.36E-06	2.04E-06	PPARD/BCL2/CYP1B1/HAS2/PLAU/MYC/SERPINE1/NFE2L2/IGFBP3	9
BP	GO:0001701	in utero embryonic development	18/210	387/18800	3.77E-07	4.47E-06	2.09E-06	AR/NCOA1/CHEK1/AKT1/MAPK1/APOB/EGFR/VEGFA/BCL2L1/TP53/CASP8/HIF1A/MYC/GJA1/NOS3/CCNB1/COL3A1/IGF2	18
BP	GO:0051048	negative regulation of secretion	12/210	167/18800	3.97E-07	4.69E-06	2.20E-06	OPRM1/MAOB/HMOX1/PPP3CA/HMGCR/SREBF1/ABAT/EGF/GJA1/IL1B/PTGER3/ERBB3	12
BP	GO:0072594	establishment of protein localization to organelle	19/210	431/18800	4.00E-07	4.72E-06	2.21E-06	PTGS2/HSP90AB1/MAPK14/DRD1/AKT1/BAX/MAPK8/CDK1/PPP3CA/SREBF1/STAT3/CDKN1A/IL10RA/TP53/NFKBIA/HSPA5/IFNG/NPEPPS/HK2	19
BP	GO:0097194	execution phase of apoptosis	9/210	84/18800	4.07E-07	4.79E-06	2.24E-06	AKT1/BAX/CASP3/BCL2L1/CASP9/TP53/CASP8/TOP2A/HSF1	9
BP	GO:0046902	regulation of mitochondrial membrane permeability	8/210	62/18800	4.27E-07	5.01E-06	2.35E-06	GSK3B/BCL2/BAX/MAPK8/BAD/BCL2L1/TP53/HK2	8
BP	GO:00018	tissue	15/210	272/1	4.36E-	5.10E-06	2.39E-06	PTGS2/ADRB2/K	15

	94	homeostasis		8800	07			DR/BCL2/BAX/SOD1/RB1/VEGFA/PRKCA/ACACA/GJA1/NOS3/HSPB1/COL3A1/SPP1	
BP	GO:0010875	positive regulation of cholesterol efflux	6/210	27/18800	4.40E-07	5.11E-06	2.39E-06	RXRA/PPARG/CES1/NFKBIA/CAV1/PON1	6
BP	GO:0071880	adenylate cyclase-activating adrenergic receptor signaling pathway	6/210	27/18800	4.40E-07	5.11E-06	2.39E-06	ADRA1A/ADRB2/ADRA1B/ADRA1D/DRD1/ADRB1	6
BP	GO:0009306	protein secretion	17/210	350/18800	4.40E-07	5.11E-06	2.39E-06	ACHE/OPRM1/NOS2/PPARG/PPARD/ALOX5/PPP3CA/BAD/MTTP/HMGCR/SREBF1/ABAT/RAF1/HIF1A/IL1B/IFNG/IL1A	17
BP	GO:0035592	establishment of protein localization to extracellular region	17/210	351/18800	4.58E-07	5.30E-06	2.48E-06	ACHE/OPRM1/NOS2/PPARG/PPARD/ALOX5/PPP3CA/BAD/MTTP/HMGCR/SREBF1/ABAT/RAF1/HIF1A/IL1B/IFNG/IL1A	17
BP	GO:0045444	fat cell differentiation	14/210	237/18800	4.69E-07	5.41E-06	2.53E-06	PTGS2/ADRB2/PPARG/MAPK14/PPARD/ADRB1/AKT1/ALOX5/SLC2A4/SREBF1/CCND1/RUNX1T1/SULT1E1/E2F1	14

BP	GO:0071347	cellular response to interleukin-1	10/210	111/18800	4.72E-07	5.43E-06	2.54E-06	RELA/IKBKB/HAS2/MAPK3/MMP2/HIF1A/MYC/IL1B/CCL2/CXCL8	10
BP	GO:0051099	positive regulation of binding	12/210	171/18800	5.12E-07	5.87E-06	2.75E-06	HSP90AB1/PPARG/GSK3B/RB1/MMP9/EGF/CAV1/MYC/IFNG/PARP1/HSF1/PON1	12
BP	GO:0006939	smooth muscle contraction	10/210	112/18800	5.13E-07	5.87E-06	2.75E-06	PTGS2/CHRM3/ADRA1A/CHRM2/ADRB2/DRD1/SOD1/ABAT/CAV1/PTGER3	10
BP	GO:0042098	T cell proliferation	13/210	204/18800	5.15E-07	5.88E-06	2.75E-06	IL4/BAX/CASP3/VCAM1/TP53/ERBB2/GJA1/IL1B/IL2RA/IL1A/IGF2/CD40LG/IRF1	13
BP	GO:0040013	negative regulation of locomotion	18/210	396/18800	5.27E-07	5.98E-06	2.80E-06	DPP4/DPEP1/PPARG/PPARD/IL4/AKT1/BCL2/TNFAIP6/HMOX1/CYP1B1/CYP19A1/STAT3/GJA1/CCL2/SERPINE1/NFE2L2/COL3A1/IGFBP3	18
BP	GO:0007595	lactation	7/210	44/18800	5.30E-07	5.98E-06	2.80E-06	SLC6A3/CCND1/VEGFA/HIF1A/CAV1/GJA1/HK2	7
BP	GO:0045981	positive regulation of nucleotide metabolic process	7/210	44/18800	5.30E-07	5.98E-06	2.80E-06	NOS2/IL4/PPARA/HIF1A/MYC/NOS3/IFNG	7
BP	GO:1900544	positive regulation of purine nucleoti	7/210	44/18800	5.30E-07	5.98E-06	2.80E-06	NOS2/IL4/PPARA/HIF1A/MYC/NOS3/IFNG	7

		de metabolic process							
BP	GO:0002687	positive regulation of leukocyte migration	11/210	141/18800	5.38E-07	6.06E-06	2.84E-06	F7/IL4/ICAM1/MAPK3/MAPK1/VEGFA/IL6R/CXCL8/SERPINE1/IL1A/CXCL10	11
BP	GO:0044772	mitotic cell cycle phase transition	19/210	440/18800	5.48E-07	6.15E-06	2.88E-06	CHEK1/CCNA2/CDK2/AKT1/BCL2/CDK1/CYP1A1/PPP3CA/CCND1/RB1/CDK4/EGFR/CDKN1A/TP53/MYC/CCL2/CCNB1/CHEK2/E2F1	19
BP	GO:0040014	regulation of multicellular organism growth	8/210	64/18800	5.49E-07	6.15E-06	2.88E-06	ADRB2/SLC6A3/ADRB1/BCL2/SOD1/STAT3/CDK4/IGF2	8
BP	GO:1904659	glucose transmembrane transport	10/210	113/18800	5.57E-07	6.23E-06	2.91E-06	MAPK14/DRD1/PPARD/AKT1/SLC2A4/MYC/IL1B/PRKCB/NFE2L2/HK2	10
BP	GO:0010469	regulation of signaling receptor activity	12/210	173/18800	5.80E-07	6.46E-06	3.02E-06	ADRB2/OPRM1/PPARG/ESR2/NCF1/PPARA/PLAU/EGF/HIF1A/CCL2/SERPINE1/IFNG	12
BP	GO:0090066	regulation of anatomical structure size	20/210	487/18800	5.99E-07	6.66E-06	3.12E-06	PTGS2/CHRM3/ADRA1A/ADRB2/ADRA1B/ADRA1D/HSP90AB1/SLC6A4/GSK3B/DRD1/PPARD/ADRB1/SOD1/VEGFA/MM	20

								P2/CAV1/NOS3/SPP1/RASA1/MAP2	
BP	GO:0043542	endothelial cell migration	15/210	279/18800	6.02E-07	6.68E-06	3.13E-06	PTGS2/DPP4/PPARG/KDR/ATP5F1B/AKT1/HMOX1/CYP1B1/VEGFA/EGF/PRKCA/HIF1A/NOS3/HSPB1/NFE2L2	15
BP	GO:0043200	response to amino acid	10/210	114/18800	6.05E-07	6.69E-06	3.13E-06	F7/CASP3/BAD/EGFR/BCL2L1/MMP2/COL1A1/NQO1/COL3A1/HSF1	10
BP	GO:0035264	multicellular organism growth	11/210	143/18800	6.20E-07	6.79E-06	3.18E-06	AR/ADRB2/SLC6A3/ADRB1/BCL2/SOD1/STAT3/CDK4/TP53/COL3A1/IGF2	11
BP	GO:0006940	regulation of smooth muscle contraction	8/210	65/18800	6.20E-07	6.79E-06	3.18E-06	PTGS2/CHRM3/ADRA1A/CHRM2/ADRB2/SOD1/ABAT/CAV1	8
BP	GO:0042446	hormone biosynthetic process	8/210	65/18800	6.20E-07	6.79E-06	3.18E-06	AKR1B1/HSD3B2/HSD3B1/DIO1/CYP19A1/POR/HIF1A/DUOX2	8
BP	GO:0072577	endothelial cell apoptotic process	8/210	65/18800	6.20E-07	6.79E-06	3.18E-06	KDR/IL4/ICAM1/AKR1C3/CCL2/SERPINE1/NFE2L2/CD40LG	8
BP	GO:1904646	cellular response to amyloid-beta	7/210	45/18800	6.22E-07	6.79E-06	3.18E-06	ADRB2/GSK3B/ICAM1/VCAM1/ABCC1/GJA1/PARP1	7
BP	GO:0071692	protein localization to extracellular	17/210	359/18800	6.27E-07	6.83E-06	3.20E-06	ACHE/OPRM1/NOS2/PPARG/PPARD/ALOX5/PPP3CA/BAD/MTTP/HMGCR/SREBF1/A	17

		region						BAT/RAF1/HIF1A/IL1B/IFNG/IL1A	
BP	GO:0071902	positive regulation of protein serine/threonine kinase activity	13/210	208/18800	6.43E-07	6.98E-06	3.27E-06	ADRB2/HSP90AB1/AKT1/CCND1/EGFR/VEGFA/EGF/ERBB2/IL1B/CCNB1/IFNG/IGF2/CD40LG	13
BP	GO:0043534	blood vessel endothelial cell migration	12/210	175/18800	6.56E-07	7.09E-06	3.32E-06	PTGS2/PPARG/KDR/ATP5F1B/AKT1/HMOX1/VEGFA/PRKCA/HIF1A/NOS3/HSPB1/NFE2L2	12
BP	GO:0018107	peptidyl-threonine phosphorylation	10/210	115/18800	6.56E-07	7.09E-06	3.32E-06	GSK3B/CHEK1/AKT1/BCL2/MAPK8/CDK1/MAPK1/EGF/PRKCA/PRKCB	10
BP	GO:0046879	hormone secretion	15/210	281/18800	6.59E-07	7.11E-06	3.33E-06	NOS2/PPARG/PPARD/ALOX5/PPP3CA/BAD/CYP19A1/SREBF1/ABAT/RAF1/HIF1A/GJA1/IL1B/IFNG/SPP1	15
BP	GO:0033135	regulation of peptidyl-serine phosphorylation	11/210	144/18800	6.64E-07	7.12E-06	3.33E-06	PTGS2/HSP90AB1/OPRD1/AKT1/BCL2/BAX/EGFR/VEGFA/RAF1/CAV1/IFNG	11
BP	GO:0051592	response to calcium ion	11/210	144/18800	6.64E-07	7.12E-06	3.33E-06	SCN5A/DPEP1/JUN/PPP3CA/AKR1C3/BAD/MTTP/CCND1/FOS/HSPA5/CAV1	11
BP	GO:0042593	glucose homeostasis	14/210	244/18800	6.65E-07	7.12E-06	3.33E-06	PPARG/PPARD/AKT1/ICAM1/ALOX5/SLC2A4/PPP3CA/BAD/ADIPOR2/BACE2/STAT3/	14

								RAF1/HIF1A/HK2	
BP	GO:0007589	body fluid secretion	9/210	89/18800	6.69E-07	7.13E-06	3.33E-06	CHRM3/CHRM1/SLC6A3/CCND1/VEGFA/HIF1A/CAV1/GJA1/HK2	9
BP	GO:1903578	regulation of ATP metabolic process	9/210	89/18800	6.69E-07	7.13E-06	3.33E-06	IL4/PPARA/STAT3/EIF6/TP53/HIF1A/MYC/IFNG/PARP1	9
BP	GO:1901990	regulation of mitotic cell cycle phase transition	16/210	321/18800	6.91E-07	7.31E-06	3.42E-06	CHEK1/CDK2/AKT1/BCL2/CDK1/CYP1A1/CCND1/RB1/CDK4/EGFR/CDKN1A/TP53/CCL2/CCNB1/CHEK2/E2F1	16
BP	GO:0008209	androgen metabolic process	6/210	29/18800	6.93E-07	7.31E-06	3.42E-06	ESR1/HSD3B2/HSD3B1/CYP3A4/CYP19A1/SPP1	6
BP	GO:0095500	acetylcholine receptor signaling pathway	6/210	29/18800	6.93E-07	7.31E-06	3.42E-06	CHRM3/CHRM1/ACHE/CHRM2/OPRM1/CHRM5	6
BP	GO:1902175	regulation of oxidative stress-induced intrinsic apoptotic signaling pathway	6/210	29/18800	6.93E-07	7.31E-06	3.42E-06	AKT1/SOD1/HIF1A/HSPB1/NFE2L2/PARP1	6
BP	GO:0033500	carbohydrate	14/210	245/18800	6.99E-07	7.33E-06	3.43E-06	PPARG/PPARD/AKT1/ICAM1/ALO	14

		homeostasis						X5/SLC2A4/PPP3CA/BAD/ADIPOR2/BACE2/STAT3/RAF1/HIF1A/HK2	
BP	GO:0045926	negative regulation of growth	14/210	245/18800	6.99E-07	7.33E-06	3.43E-06	ADRB2/SLC6A4/ESR2/PPARD/ADRB1/BCL2/PPARA/RB1/CDKN1A/TP53/HIF1A/GJA1/SPP1/MAP2	14
BP	GO:0043524	negative regulation of neuron apoptotic process	11/210	145/18800	7.12E-07	7.45E-06	3.48E-06	HSP90AB1/JUN/BCL2/BAX/HMOX1/SOD1/BCL2L1/HIF1A/CCL2/ERBB3/RASA1	11
BP	GO:0046677	response to antibiotic	7/210	46/18800	7.26E-07	7.58E-06	3.55E-06	CASP3/CYP1A1/SOD1/CASP9/TP53/CASP8/HSPA5	7
BP	GO:0031331	positive regulation of cellular catabolic process	19/210	449/18800	7.43E-07	7.73E-06	3.62E-06	ADRB2/KDR/GSK3B/IL4/AKT1/BAX/HMOX1/MAPK3/LDLR/BAD/PPARA/EGF/HIF1A/CAV1/MYC/IL1B/IFNG/NFE2L2/HSF1	19
BP	GO:0008645	hexose transmembrane transport	10/210	117/18800	7.70E-07	8.00E-06	3.74E-06	MAPK14/DRD1/PPARD/AKT1/SLC2A4/MYC/IL1B/PRKCB/NFE2L2/HK2	10
BP	GO:0009895	negative regulation of catabolic process	16/210	324/18800	7.81E-07	8.09E-06	3.79E-06	ADRA1A/HSP90AB1/NOS2/MAPK14/RELA/AKT1/BCL2/HMOX1/HMGCR/PPARA/STAT3/EGFR/IL10RA/TP53/IL1B/E2F1	16
BP	GO:0007187	G protein-coupled	8/210	67/18800	7.86E-07	8.12E-06	3.80E-06	CHRM3/CHRM1/CHRM2/OPRM1/DRD1/CHRM5/OP	8

		receptor signaling pathway, coupled to cyclic nucleotide second messenger						RD1/CCL2	
BP	GO:0097306	cellular response to alcohol	9/210	91/18800	8.10E-07	8.33E-06	3.90E-06	AKT1/CYP1B1/AHR/AKR1C3/UGT1A1/CES1/CDK4/ACACA/MYC	9
BP	GO:1903351	cellular response to dopamine	9/210	91/18800	8.10E-07	8.33E-06	3.90E-06	CHRM3/CHRM1/CHRM2/HTR3A/GSK3B/DRD1/CHRM5/MAPK3/MAPK1	9
BP	GO:0043500	muscle adaptation	10/210	118/18800	8.33E-07	8.55E-06	4.00E-06	SCN5A/ADRA1A/PPARG/HMOX1/PPP3CA/PPARA/PRKCA/IL1B/NOS3/PARP1	10
BP	GO:0034764	positive regulation of transmembrane transport	13/210	213/18800	8.41E-07	8.59E-06	4.02E-06	ADRB2/MAPK14/DRD1/AKT1/BAX/GSTM2/KCNH2/CA2/CCL2/IFNG/NFE2L2/CXCL11/CXCL10	13
BP	GO:0042220	response to cocaine	7/210	47/18800	8.45E-07	8.59E-06	4.02E-06	OPRM1/HSP90AB1/CCNA2/DRD1/SLC6A3/ABAT/HSPA5	7
BP	GO:0045776	negative regulation of blood pressure	7/210	47/18800	8.45E-07	8.59E-06	4.02E-06	ADRA1A/ADRB2/NOS2/ADRB1/PPARA/ABAT/NOS3	7
BP	GO:0070849	response to epiderm	7/210	47/18800	8.45E-07	8.59E-06	4.02E-06	AKT1/MAPK3/MAPK1/EGFR/ERBB2/MYC/COL1A1	7

		al growth factor							
BP	GO:0015833	peptide transport	14/210	249/18800	8.48E-07	8.60E-06	4.02E-06	NOS2/PPARD/ALOX5/PPP3CA/BAD/SREBF1/ABCC1/ABAT/CA2/RAF1/HIF1A/GJA1/IL1B/IFNG	14
BP	GO:0043627	response to estrogen	8/210	68/18800	8.82E-07	8.88E-06	4.16E-06	AR/ESR1/F7/HMOX1/CCND1/MMP2/CAV1/MYC	8
BP	GO:0061180	mammary gland epithelium development	8/210	68/18800	8.82E-07	8.88E-06	4.16E-06	PGR/AR/ESR1/AKT1/BAX/MAPK1/CCND1/HIF1A	8
BP	GO:0016042	lipid catabolic process	16/210	327/18800	8.82E-07	8.88E-06	4.16E-06	PPARD/AKT1/CYP3A4/CYP1A2/CYP1B1/AKR1C3/LDLR/APOB/PLB1/CYP19A1/PPARA/CES1/CDK4/IL1B/SULT1E1/SPP1	16
BP	GO:1903350	response to dopamine	9/210	92/18800	8.89E-07	8.93E-06	4.18E-06	CHRM3/CHRM1/CHRM2/HTR3A/GSK3B/DRD1/CHRM5/MAPK3/MAPK1	9
BP	GO:0007249	I-kappaB kinase/NF-kappaB signaling	15/210	288/18800	8.99E-07	9.00E-06	4.21E-06	ESR1/RELA/IKBKB/AKT1/STAT1/HMOX1/GSTP1/NFKBIA/CASP8/GJA1/IL1B/PRKCB/HSPB1/IL1A/CHUK	15
BP	GO:0015749	monosaccharide transmembrane transport	10/210	119/18800	9.01E-07	9.00E-06	4.21E-06	MAPK14/DRD1/PPARD/AKT1/SLC2A4/MYC/IL1B/PRKCB/NFE2L2/HK2	10
BP	GO:0009404	toxin metaboli	5/210	17/18800	9.20E-07	9.11E-06	4.26E-06	CYP3A4/CYP1A2/CYP1A1/CYP1B1/	5

		c process						NFE2L2	
BP	GO:0051412	response to corticosterone	5/210	17/18800	9.20E-07	9.11E-06	4.26E-06	MAOB/HSD3B2/HSD3B1/CCND1/FOS	5
BP	GO:0090399	replicative senescence	5/210	17/18800	9.20E-07	9.11E-06	4.26E-06	CHEK1/CDKN1A/TP53/SERPINE1/CHEK2	5
BP	GO:0090594	inflammatory response to wounding	5/210	17/18800	9.20E-07	9.11E-06	4.26E-06	PPARG/HMOX1/ALOX5/HIF1A/IL1A	5
BP	GO:0009914	hormone transport	15/210	290/18800	9.81E-07	9.68E-06	4.53E-06	NOS2/PPARG/PPARD/ALOX5/PPP3CA/BAD/CYP19A1/SREBF1/ABAT/RAF1/HIF1A/GJA1/IL1B/IFNG/SP1	15
BP	GO:0030258	lipid modification	13/210	216/18800	9.85E-07	9.71E-06	4.54E-06	MAPK14/CHRM5/PPARD/AKT1/CYP3A4/CYP1A1/ALOX5/PPARA/ADIPOR2/SOAT2/SOAT1/POR/DGAT2	13
BP	GO:1905145	cellular response to acetylcholine	6/210	31/18800	1.05E-06	1.04E-05	4.85E-06	CHRM3/CHRM1/ACHE/CHRM2/OPRM1/CHRM5	6
BP	GO:0018108	peptidyl-tyrosine phosphorylation	17/210	373/18800	1.06E-06	1.04E-05	4.88E-06	ADRA1A/KDR/NCF1/IL4/INSRR/MAPK3/EGFR/VEGFA/EGF/IL6R/TP53/ERBB2/CAV1/IFNG/HSF1/IGF2/ERBB3	17
BP	GO:0022411	cellular component	19/210	460/18800	1.07E-06	1.04E-05	4.88E-06	ADRB2/DPP4/PRSS1/GSK3B/BAX/MMP1/CDK1/SRE	19

		disassembly						BF1/MMP3/MMP2/MMP9/TP53/PRKCA/HIF1A/MYC/PRKCB/TOP2A/HSF1/HK2	
BP	GO:0002690	positive regulation of leukocyte chemotaxis	9/210	94/18800	1.07E-06	1.04E-05	4.88E-06	F7/IL4/MAPK3/MAPK1/VEGFA/IL6R/CXCL8/SERPINE1/CXCL10	9
BP	GO:0072521	purine-containing compound metabolic process	18/210	416/18800	1.07E-06	1.04E-05	4.88E-06	NOS2/IL4/ATP5F1B/BAD/HMGCR/PPARA/STAT3/EIF6/HIF1A/ACACA/MYC/NOS3/SULT1E1/IFNG/PARP1/ACP3/HK2/DGAT2	18
BP	GO:0097696	receptor signaling pathway via STAT	12/210	184/18800	1.12E-06	1.09E-05	5.08E-06	PPARG/IL4/STAT1/CYP1B1/STAT3/IL10RA/EGF/IL6R/CAV1/CCL2/IFNG/HSF1	12
BP	GO:0022602	ovulation cycle process	7/210	49/18800	1.13E-06	1.10E-05	5.14E-06	PGR/ESR1/TNFAIP6/CASP3/MMP2/HSPA5/NOS3	7
BP	GO:0034504	protein localization to nucleus	15/210	294/18800	1.16E-06	1.13E-05	5.27E-06	PTGS2/HSP90AB1/MAPK14/GSK3B/DRD1/AKT1/CDK1/PPP3CA/STAT3/CDKN1A/TP53/NFKBIA/COL1A1/IFNG/PARP1	15
BP	GO:0018212	peptidyl-tyrosine modification	17/210	376/18800	1.19E-06	1.15E-05	5.36E-06	ADRA1A/KDR/NCF1/IL4/INSRR/MAPK3/EGFR/VEGFA/EGF/IL6R/TP53/ERBB2/CAV1/IFNG/HSF1/IGF2/ERBB3	17
BP	GO:20003	positive	8/210	71/18	1.23E-	1.19E-05	5.56E-06	MAPK14/CYP1B1	8

	79	regulation of reactive oxygen species metabolic process		800	06			/GSTP1/AKR1C3/SOD1/CDKN1A/TP53/NFE2L2	
BP	GO:0050665	hydrogen peroxide biosynthetic process	5/210	18/18800	1.26E-06	1.21E-05	5.67E-06	MAOB/CYP1A2/CYP1A1/SOD1/DUOX2	5
BP	GO:0051385	response to mineralocorticoid	6/210	32/18800	1.29E-06	1.23E-05	5.76E-06	MAOB/HSD3B2/HSD3B1/CCND1/FOS/PARP1	6
BP	GO:1905144	response to acetylcholine	6/210	32/18800	1.29E-06	1.23E-05	5.76E-06	CHRM3/CHRM1/ACHE/CHRM2/OPRM1/CHRM5	6
BP	GO:0006692	prostanoid metabolic process	7/210	50/18800	1.30E-06	1.24E-05	5.79E-06	PTGS2/PTGS1/AKR1B1/GSTP1/GSTM1/AKR1C3/IL1B	7
BP	GO:0006693	prostaglandin metabolic process	7/210	50/18800	1.30E-06	1.24E-05	5.79E-06	PTGS2/PTGS1/AKR1B1/GSTP1/GSTM1/AKR1C3/IL1B	7
BP	GO:0030857	negative regulation of epithelial cell differentiation	7/210	50/18800	1.30E-06	1.24E-05	5.79E-06	STAT1/CCND1/VEGFA/MMP9/CAV1/IFNG/IL1A	7
BP	GO:1902041	regulation of extrinsic apoptotic	7/210	50/18800	1.30E-06	1.24E-05	5.79E-06	GSK3B/HMOX1/ICAM1/BCL2L1/RAF1/NOS3/SERPINE1	7

		signaling pathway via death domain receptors							
BP	GO:0018210	peptidyl-threonine modification	10/210	124/18800	1.32E-06	1.25E-05	5.83E-06	GSK3B/CHEK1/AKT1/BCL2/MAPK8/CDK1/MAPK1/EGF/PRKCA/PRKCB	10
BP	GO:0050810	regulation of steroid biosynthetic process	8/210	72/18800	1.38E-06	1.30E-05	6.07E-06	AKR1C3/SOD1/APOB/SREBF1/CES1/POR/IFNG/IL1A	8
BP	GO:0007189	adenylate cyclase-activating G protein-coupled receptor signaling pathway	11/210	155/18800	1.38E-06	1.30E-05	6.07E-06	ADRA1A/ADRB2/OPRM1/ADRA1B/ADRA1D/DRD1/ADRB1/PRKCA/PTGER3/CXCL11/CXCL10	11
BP	GO:1905952	regulation of lipid localization	11/210	155/18800	1.38E-06	1.30E-05	6.07E-06	PPARG/PPARD/AKT1/APOB/CYP19A1/PPARA/CES1/NFKBIA/IL1B/IL1A/SPP1	11
BP	GO:1902806	regulation of cell cycle G1/S phase transition	12/210	188/18800	1.40E-06	1.31E-05	6.15E-06	CDK2/AKT1/BCL2/CYP1A1/CCND1/RB1/EGFR/CDKN1A/TP53/CCL2/CHEK2/E2F1	12
BP	GO:0050870	positive regulatio	13/210	223/18800	1.41E-06	1.32E-05	6.17E-06	DPP4/IL4/AKT1/VCAM1/BAD/CAV	13

		n of T cell activation						1/IL1B/CCL2/IL2RA/IFNG/IL1A/IGF2/CD40LG	
BP	GO:0090316	positive regulation of intracellular protein transport	11/210	156/18800	1.47E-06	1.37E-05	6.42E-06	PTGS2/CHRM1/HSP90AB1/MAPK14/GSK3B/MAPK8/CDK1/ERBB2/IL1B/IFNG/NPEPPS	11
BP	GO:0030098	lymphocyte differentiation	17/210	382/18800	1.47E-06	1.37E-05	6.42E-06	IL4/BCL2/BAX/VCAM1/BAD/SOD1/STAT3/IL6R/TP53/ERBB2/IL1B/IL2RA/IFNG/IL1A/RUNX2/CD40LG/IRF1	17
BP	GO:0036473	cell death in response to oxidative stress	9/210	98/18800	1.52E-06	1.41E-05	6.60E-06	AKT1/BCL2/CYP1B1/SOD1/MMP3/HIF1A/HSPB1/NFE2L2/PARP1	9
BP	GO:0120162	positive regulation of cold-induced thermogenesis	9/210	98/18800	1.52E-06	1.41E-05	6.60E-06	ACHE/ADRB2/ADRB1/IL4/ADIPOR2/VEGFA/CAV1/GJA1/HSF1	9
BP	GO:0150076	neuroinflammatory response	8/210	73/18800	1.53E-06	1.42E-05	6.62E-06	PTGS2/JUN/IL4/LDLR/MMP3/MMP9/IL1B/IFNG	8
BP	GO:0071875	adrenergic receptor signaling pathway	6/210	33/18800	1.56E-06	1.44E-05	6.73E-06	ADRA1A/ADRB2/ADRA1B/ADRA1D/DRD1/ADRB1	6
BP	GO:0030856	regulation of epithelia	11/210	157/18800	1.57E-06	1.44E-05	6.75E-06	IKBKB/STAT1/BAD/CCND1/VEGFA/MMP9/CAV1/IL	11

		l cell differentiation						1B/SERPINE1/IFNG/IL1A	
BP	GO:0030217	T cell differentiation	14/210	263/18800	1.62E-06	1.49E-05	6.97E-06	IL4/BCL2/BAD/SOD1/STAT3/IL6R/TP53/ERBB2/IL1B/IL2RA/IFNG/IL1A/RUNX2/IRF1	14
BP	GO:0051054	positive regulation of DNA metabolic process	14/210	263/18800	1.62E-06	1.49E-05	6.97E-06	HSP90AB1/CDK2/IL4/AKT1/BAX/CDK1/CYP1B1/MAPK3/MAPK1/EGFR/EGF/MYC/PARP1/HSF1	14
BP	GO:0070665	positive regulation of leukocyte proliferation	11/210	158/18800	1.67E-06	1.53E-05	7.14E-06	IL4/BCL2/VCAM1/MAPK3/MAPK1/CDKN1A/IL1B/IL2RA/IL1A/IGF2/CD40LG	11
BP	GO:0007213	G protein-coupled acetylcholine receptor signaling pathway	5/210	19/18800	1.70E-06	1.54E-05	7.22E-06	CHRM3/CHRM1/CHRM2/OPRM1/CHRM5	5
BP	GO:0010885	regulation of cholesterol storage	5/210	19/18800	1.70E-06	1.54E-05	7.22E-06	PPARG/PPARD/APOB/PPARA/CES1	5
BP	GO:0055093	response to hyperoxia	5/210	19/18800	1.70E-06	1.54E-05	7.22E-06	CYP1A1/CAT/MMP2/CAV1/COL1A1	5
BP	GO:0010874	regulation of cholesterol	7/210	52/18800	1.72E-06	1.56E-05	7.28E-06	RXRA/PPARG/CES1/EGF/NFKBIA/CAV1/PON1	7

		efflux							
BP	GO:0045931	positive regulation of mitotic cell cycle	10/210	128/18800	1.76E-06	1.59E-05	7.46E-06	AKT1/CDK1/CYP1A1/CCND1/RB1/CDK4/EGFR/PRKCA/CCNB1/HSF1	10
BP	GO:0034383	low-density lipoprotein particle clearance	6/210	34/18800	1.87E-06	1.69E-05	7.89E-06	HMOX1/LDLR/APOB/SOAT2/SOAT1/DGAT2	6
BP	GO:0034405	response to fluid shear stress	6/210	34/18800	1.87E-06	1.69E-05	7.89E-06	PTGS2/AKT1/HAS2/MMP2/NOS3/NFE2L2	6
BP	GO:0042573	retinoic acid metabolic process	6/210	34/18800	1.87E-06	1.69E-05	7.89E-06	ADH1B/ADH1C/CYP3A4/CYP1A1/AKR1C3/UGT1A1	6
BP	GO:0010822	positive regulation of mitochondrion organization	8/210	75/18800	1.88E-06	1.69E-05	7.89E-06	KDR/GSK3B/BAX/MAPK8/BAD/MMP9/TP53/HIF1A	8
BP	GO:0071479	cellular response to ionizing radiation	8/210	75/18800	1.88E-06	1.69E-05	7.89E-06	MAPK14/BCL2L1/CDKN1A/TP53/ELK1/HSPA5/CHEK2/HSF1	8
BP	GO:0009267	cellular response to starvation	11/210	160/18800	1.89E-06	1.69E-05	7.89E-06	BCL2/MAPK8/AKR1C3/MAPK3/MAPK1/PPARA/SREBF1/CDKN1A/TP53/HSPA5/NFE2L2	11
BP	GO:0010594	regulation of endothel	13/210	230/18800	1.99E-06	1.77E-05	8.29E-06	PTGS2/PPARG/KDR/ATP5F1B/AKT1/HMOX1/VEGF	13

		ial cell migration						A/EGF/PRKCA/HIF1A/NOS3/HSPB1/NFE2L2	
BP	GO:0050670	regulation of lymphocyte proliferation	13/210	230/18800	1.99E-06	1.77E-05	8.29E-06	IL4/BCL2/CASP3/VCAM1/AHR/CDKN1A/ERBB2/IL1B/IL2RA/IL1A/IGF2/CD40LG/IRF1	13
BP	GO:0006869	lipid transport	17/210	391/18800	2.02E-06	1.80E-05	8.40E-06	NOS2/PPARG/PPARD/AKT1/LDLR/MTTP/APOB/CYP19A1/PPARA/ABCC1/SOAT2/CES1/CAV1/IL1B/IL1A/ABCG2/SPP1	17
BP	GO:0006913	nucleocytoplasmic transport	15/210	308/18800	2.07E-06	1.83E-05	8.59E-06	PTGS2/HSP90AB1/MAPK14/GSK3B/DRD1/AKT1/CDK1/PPP3CA/STAT3/EIF6/CDKN1A/TP53/NFKBIA/IL1B/IFNG	15
BP	GO:0051169	nuclear transport	15/210	308/18800	2.07E-06	1.83E-05	8.59E-06	PTGS2/HSP90AB1/MAPK14/GSK3B/DRD1/AKT1/CDK1/PPP3CA/STAT3/EIF6/CDKN1A/TP53/NFKBIA/IL1B/IFNG	15
BP	GO:0042594	response to starvation	12/210	196/18800	2.17E-06	1.92E-05	8.97E-06	BCL2/MAPK8/AKR1C3/MAPK3/MAPK1/UGT1A1/PPARA/SREBF1/CDKN1A/TP53/HSPA5/NFE2L2	12
BP	GO:0034219	carbohydrate transmembrane transport	10/210	131/18800	2.17E-06	1.92E-05	8.97E-06	MAPK14/DRD1/PPARD/AKT1/SLC2A4/MYC/IL1B/PRKCB/NFE2L2/HK2	10
BP	GO:0006163	purine nucleotide	17/210	394/18800	2.24E-06	1.96E-05	9.18E-06	NOS2/IL4/ATP5F1B/BAD/HMGCR/PPARA/STAT3/EIF	17

		metabolic process						6/HIF1A/ACACA/MYC/NOS3/SULT1E1/IFNG/PARP1/HK2/DGAT2	
BP	GO:0071398	cellular response to fatty acid	6/210	35/18800	2.24E-06	1.96E-05	9.18E-06	AKR1C3/LDLR/SREBF1/CDK4/E2F1/DGAT2	6
BP	GO:1904031	positive regulation of cyclin-dependent protein kinase activity	6/210	35/18800	2.24E-06	1.96E-05	9.18E-06	HSP90AB1/AKT1/CCND1/EGFR/CDKN1A/CCNB1	6
BP	GO:0032944	regulation of mononuclear cell proliferation	13/210	233/18800	2.30E-06	2.00E-05	9.37E-06	IL4/BCL2/CASP3/VCAM1/AHR/CDKN1A/ERBB2/IL1B/IL2RA/IL1A/IGF2/CD40LG/IRF1	13
BP	GO:0043154	negative regulation of cysteine-type endopeptidase activity involved in apoptotic process	8/210	77/18800	2.31E-06	2.00E-05	9.37E-06	PTGS2/DPEP1/AKT1/VEGFA/MMP9/POR/RAF1/BIRC5	8
BP	GO:0090559	regulation of membrane permeability	8/210	77/18800	2.31E-06	2.00E-05	9.37E-06	GSK3B/BCL2/BAX/MAPK8/BAD/BCL2L1/TP53/HK2	8
BP	GO:0001655	urogenital	16/210	352/18800	2.31E-06	2.00E-05	9.37E-06	AR/ESR1/AKR1B1/BCL2/BAX/STA	16

		system development						T1/HAS2/CAT/CYP19A1/VEGFA/CASP9/MMP2/MMP9/IL6R/ODC1/MYC	
BP	GO:0042176	regulation of protein catabolic process	17/210	395/18800	2.32E-06	2.01E-05	9.40E-06	HSP90AB1/NOS2/GSK3B/CDK2/RELA/AKT1/PSMD3/LDLR/HMGCR/EGFR/EGF/ODC1/CAV1/IL1B/IFNG/NFE2L2/CHEK2	17
BP	GO:0032388	positive regulation of intracellular transport	12/210	198/18800	2.41E-06	2.09E-05	9.77E-06	PTGS2/CHRM1/HSP90AB1/MAPK14/GSK3B/MAPK8/CDK1/ERBB2/IL1B/IFNG/NPEPPS/MAP2	12
BP	GO:0046034	ATP metabolic process	14/210	273/18800	2.51E-06	2.17E-05	1.02E-05	IL4/ATP5F1B/CDK1/BAD/PPARA/STAT3/EIF6/TP53/HIF1A/MYC/CCNB1/IFNG/PARP1/HK2	14
BP	GO:0010883	regulation of lipid storage	7/210	55/18800	2.53E-06	2.17E-05	1.02E-05	PPARG/PPARD/APOB/PPARA/SREBF1/CES1/NFKBIA	7
BP	GO:0043470	regulation of carbohydrate catabolic process	7/210	55/18800	2.53E-06	2.17E-05	1.02E-05	PPARA/STAT3/EIF6/TP53/HIF1A/MYC/IFNG	7
BP	GO:0071312	cellular response to alkaloid	6/210	36/18800	2.66E-06	2.29E-05	1.07E-05	OPRM1/CHEK1/CCNA2/CASP3/GSTM2/BCL2L1	6
BP	GO:0071868	cellular response to monoamine	9/210	105/18800	2.71E-06	2.32E-05	1.09E-05	CHRM3/CHRM1/CHRM2/HTR3A/GSK3B/DRD1/CHRM5/MAPK3/MAPK1	9

		stimulus							
BP	GO:0071870	cellular response to catechol amine stimulus	9/210	105/18800	2.71E-06	2.32E-05	1.09E-05	CHRM3/CHRM1/CHRM2/HTR3A/GSK3B/DRD1/CHRM5/MAPK3/MAPK1	9
BP	GO:0006970	response to osmotic stress	8/210	79/18800	2.80E-06	2.39E-05	1.12E-05	PTGS2/HSP90AB1/AKR1B1/BAX/CASP3/SLC2A4/BAD/TP53	8
BP	GO:0072332	intrinsic apoptotic signaling pathway by p53 class mediator	8/210	79/18800	2.80E-06	2.39E-05	1.12E-05	BCL2/BAX/CDKN1A/TP53/MYC/CHEK2/E2F1/E2F2	8
BP	GO:0006006	glucose metabolic process	12/210	201/18800	2.82E-06	2.40E-05	1.12E-05	NCOA2/MAPK14/GSK3B/PPARD/AKT1/BAD/PPARA/TP53/MYC/IGF2/HK2/DGAT2	12
BP	GO:0043401	steroid hormone mediated signaling pathway	10/210	135/18800	2.86E-06	2.42E-05	1.13E-05	PGR/AR/RXRA/NR3C2/ESR1/ESR2/PPARD/PPARA/RXRB/PARP1	10
BP	GO:0001836	release of cytochrome c from mitochondria	7/210	56/18800	2.86E-06	2.42E-05	1.13E-05	AKT1/BCL2/BAX/BAD/BCL2L1/MMP9/TP53	7
BP	GO:0038083	peptidyl-tyrosine autophosphorylation	5/210	21/18800	2.92E-06	2.45E-05	1.15E-05	KDR/MAPK3/EGFR/VEGFA/CAV1	5

BP	GO:0051767	nitric-oxide synthase biosynthetic process	5/210	21/18800	2.92E-06	2.45E-05	1.15E-05	KDR/STAT1/GSTP1/CCL2/IFNG	5
BP	GO:0051769	regulation of nitric-oxide synthase biosynthetic process	5/210	21/18800	2.92E-06	2.45E-05	1.15E-05	KDR/STAT1/GSTP1/CCL2/IFNG	5
BP	GO:0034379	very-low-density lipoprotein particle assembly	4/210	10/18800	3.01E-06	2.53E-05	1.18E-05	MTTP/APOB/SOAT2/SOAT1	4
BP	GO:0010959	regulation of metal ion transport	17/210	403/18800	3.04E-06	2.54E-05	1.19E-05	PTGS2/SCN5A/ADRB2/DRD1/OPRD1/AKT1/BCL2/BAX/GSTM2/KCNH2/EGF/CAV1/CCL2/NOS3/IFNG/CXCL11/CXCL10	17
BP	GO:0032094	response to food	6/210	37/18800	3.15E-06	2.63E-05	1.23E-05	OPRM1/AKT1/CYP1A1/PPARA/SREBF1/MPO	6
BP	GO:0043388	positive regulation of DNA binding	7/210	57/18800	3.23E-06	2.70E-05	1.26E-05	PPARG/RB1/MMP9/EGF/MYC/IFNG/PARP1	7
BP	GO:0006937	regulation of muscle contraction	11/210	170/18800	3.41E-06	2.84E-05	1.33E-05	PTGS2/CHRM3/SCN5A/ADRA1A/CHRM2/ADRB2/ADRA1B/GSTM2/SOD1/ABAT/CAV1	11
BP	GO:0071867	response to	9/210	108/18800	3.43E-06	2.85E-05	1.33E-05	CHRM3/CHRM1/CHRM2/HTR3A/G	9

		monoamine						SK3B/DRD1/CHRM5/MAPK3/MAPK1	
BP	GO:0071869	response to catecholamine	9/210	108/18800	3.43E-06	2.85E-05	1.33E-05	CHRM3/CHRM1/CHRM2/HTR3A/GSK3B/DRD1/CHRM5/MAPK3/MAPK1	9
BP	GO:0015980	energy derivation by oxidation of organic compounds	15/210	321/18800	3.44E-06	2.85E-05	1.33E-05	NOS2/GSK3B/PYGM/IL4/ATP5F1B/AKT1/CDK1/CYP1A2/CAT/TP53/HIF1A/MYC/CCNB1/IFNG/IGF2	15
BP	GO:0019318	hexose metabolic process	13/210	242/18800	3.49E-06	2.88E-05	1.35E-05	NCOA2/MAPK14/GSK3B/PPARD/AKR1B1/AKT1/BAD/PPARA/TP53/MYC/IGF2/HK2/DGAT2	13
BP	GO:0060284	regulation of cell development	19/210	500/18800	3.62E-06	2.98E-05	1.39E-05	OPRM1/GSK3B/IKBKB/AKT1/BCL2/HAS2/PPP3CA/LDLR/BAD/VEGFA/TP53/HIF1A/MYC/IL1B/IFNG/IL1A/SPP1/E2F1/MAP2	19
BP	GO:0019369	arachidonic acid metabolic process	7/210	58/18800	3.64E-06	2.98E-05	1.39E-05	PTGS2/PTGS1/CYP1A2/CYP1A1/CYP1B1/ALOX5/AKR1C3	7
BP	GO:0048010	vascular endothelial growth factor receptor signaling pathway	7/210	58/18800	3.64E-06	2.98E-05	1.39E-05	KDR/MAPK14/VEGFA/HIF1A/IL1B/PRKCB/HSPB1	7

BP	GO:0060135	maternal process involved in female pregnancy	7/210	58/18800	3.64E-06	2.98E-05	1.39E-05	PTGS2/PGR/AR/ESR1/PPARD/AKT1/SPP1	7
BP	GO:0090303	positive regulation of wound healing	7/210	58/18800	3.64E-06	2.98E-05	1.39E-05	F7/F3/DUOX2/THBD/SERPINE1/NFE2L2/CLDN4	7
BP	GO:0048872	homeostasis of number of cells	14/210	282/18800	3.67E-06	2.99E-05	1.40E-05	MAPK14/AKT1/BCL2/BAX/CASP3/STAT1/HMOX1/SOD1/STAT3/RB1/VEGFA/HIF1A/NOS3/IL2RA	14
BP	GO:0042310	vasoconstriction	8/210	82/18800	3.72E-06	3.03E-05	1.42E-05	PTGS2/CHRM3/ADRA1A/ADRA1B/ADRA1D/SLC6A4/MMP2/CAV1	8
BP	GO:0010888	negative regulation of lipid storage	5/210	22/18800	3.74E-06	3.04E-05	1.42E-05	PPARG/PPARD/PPARA/CES1/NFKBIA	5
BP	GO:0061042	vascular wound healing	5/210	22/18800	3.74E-06	3.04E-05	1.42E-05	KDR/ALOX5/ADIPOR2/VEGFA/SERPINE1	5
BP	GO:1902105	regulation of leukocyte differentiation	14/210	283/18800	3.82E-06	3.10E-05	1.45E-05	IL4/TNFAIP6/BAD/SOD1/RB1/FOS/CASP8/PRKCA/ERBB2/MYC/IL2RA/IFNG/HSF1/IRF1	14
BP	GO:0008584	male gonad development	10/210	140/18800	3.96E-06	3.21E-05	1.50E-05	AR/ESR1/BCL2/BAX/CYP1B1/AKR1C3/CCND1/BCL2L1/GJA1/IL1A	10
BP	GO:0051403	stress-activated MAPK cascade	13/210	245/18800	3.99E-06	3.22E-05	1.51E-05	MAPK14/MAPK10/NCF1/IKBKB/MAPK8/GSTP1/MAPK3/MAPK1/EGF	13

								R/VEGFA/MYC/IL1B/IL1A	
BP	GO:0007259	receptor signaling pathway via JAK-STAT	11/210	173/18800	4.03E-06	3.24E-05	1.52E-05	IL4/STAT1/CYP1B1/STAT3/IL10RA/EGF/IL6R/CAV1/CCL2/IFNG/HSF1	11
BP	GO:0055088	lipid homeostasis	11/210	173/18800	4.03E-06	3.24E-05	1.52E-05	PPARG/LDLR/MTTP/APOB/SOAT2/GOT1/CES1/SOAT1/ACACA/CAV1/DGAT2	11
BP	GO:0007178	transmembrane receptor protein serine/threonine kinase signaling pathway	16/210	368/18800	4.08E-06	3.27E-05	1.53E-05	HSP90AB1/PPARG/KDR/MAPK14/JUN/TNFAIP6/MAPK3/PPARA/STAT3/FOS/TP53/HSPA5/CAV1/PARP1/COL3A1/RUNX2	16
BP	GO:0046546	development of primary male sexual characteristics	10/210	141/18800	4.23E-06	3.39E-05	1.58E-05	AR/ESR1/BCL2/BAX/CYP1B1/AKR1C3/CCND1/BCL2L1/GJA1/IL1A	10
BP	GO:0060047	heart contraction	13/210	247/18800	4.36E-06	3.48E-05	1.63E-05	SCN5A/ADRA1A/CHRM2/ADRA1B/ADRA1D/ADRB1/GSTM2/SOD1/SREBF1/KCNH2/CAV1/GJA1/NOS3	13
BP	GO:0090068	positive regulation of cell cycle process	13/210	247/18800	4.36E-06	3.48E-05	1.63E-05	AKT1/CDK1/CYP1A1/CCND1/RB1/CDK4/EGFR/EGF/IL1B/CCNB1/IL1A/CHEK2/IGF2	13
BP	GO:0017038	protein import	12/210	210/18800	4.44E-06	3.54E-05	1.65E-05	PTGS2/HSP90AB1/MAPK14/DRD1/	12

								AKT1/CDK1/PPP3CA/STAT3/CDKN1A/TP53/NFKBIA/IFNG	
BP	GO:0097006	regulation of plasma lipoprotein particle levels	8/210	84/18800	4.47E-06	3.55E-05	1.66E-05	HMOX1/LDLR/MTTP/APOB/SOAT2/SOAT1/MPO/DGAT2	8
BP	GO:1901987	regulation of cell cycle phase transition	17/210	415/18800	4.49E-06	3.56E-05	1.67E-05	MAPK14/CHEK1/CDK2/AKT1/BCL2/CDK1/CYP1A1/CCND1/RB1/CDK4/EGFR/CDKN1A/TP53/CCL2/CCNB1/CHEK2/E2F1	17
BP	GO:0046824	positive regulation of nucleocytoplasmic transport	7/210	60/18800	4.59E-06	3.64E-05	1.70E-05	PTGS2/HSP90AB1/MAPK14/GSK3B/CDK1/IL1B/IFNG	7
BP	GO:0008016	regulation of heart contraction	12/210	211/18800	4.66E-06	3.68E-05	1.72E-05	SCN5A/ADRA1A/CHRM2/ADRA1B/ADRA1D/ADRB1/GSTM2/SREBF1/KCNH2/CAV1/GJA1/NOS3	12
BP	GO:0001660	fever generation	4/210	11/18800	4.70E-06	3.69E-05	1.72E-05	PTGS2/IL1B/PTGER3/IL1A	4
BP	GO:0010887	negative regulation of cholesterol storage	4/210	11/18800	4.70E-06	3.69E-05	1.72E-05	PPARG/PPARD/PPARA/CES1	4
BP	GO:0071394	cellular response to testoster	4/210	11/18800	4.70E-06	3.69E-05	1.72E-05	AR/ELK1/MYC/SPP1	4

		one stimulus							
BP	GO:0071492	cellular response to UV-A	4/210	11/18800	4.70E-06	3.69E-05	1.72E-05	MMP1/MMP3/MMP2/MMP9	4
BP	GO:0019430	removal of superoxide radicals	5/210	23/18800	4.74E-06	3.70E-05	1.73E-05	SOD1/NOS3/MPO/NFE2L2/NQO1	5
BP	GO:0030194	positive regulation of blood coagulation	5/210	23/18800	4.74E-06	3.70E-05	1.73E-05	F7/F3/THBD/SERPINE1/NFE2L2	5
BP	GO:1900048	positive regulation of hemostasis	5/210	23/18800	4.74E-06	3.70E-05	1.73E-05	F7/F3/THBD/SERPINE1/NFE2L2	5
BP	GO:0071229	cellular response to acid chemical	8/210	85/18800	4.88E-06	3.80E-05	1.78E-05	EGFR/VEGFA/BCL2L1/MMP2/MYC/COL1A1/COL3A1/HSF1	8
BP	GO:2000117	negative regulation of cysteine-type endopeptidase activity	8/210	85/18800	4.88E-06	3.80E-05	1.78E-05	PTGS2/DPEP1/AKT1/VEGFA/MMP9/POR/RAF1/BIRC5	8
BP	GO:0016032	viral process	17/210	418/18800	4.94E-06	3.83E-05	1.79E-05	HSP90AB1/GSK3B/JUN/BCL2/STAT1/CDK1/ICAM1/SLPI/LDLR/PPARA/EGFR/BCL2L1/TP53/CAV1/CCL2/CXCL8/TOP2A	17
BP	GO:0032386	regulation of intracell	15/210	332/18800	5.18E-06	4.02E-05	1.88E-05	PTGS2/CHRM1/HSP90AB1/MAPK14/GSK3B/MAPK8/	15

		ular transport						CDK1/MAPK3/MAPK1/SREBF1/ERBB2/IL1B/IFNG/NPEPPS/MAP2	
BP	GO:2000134	negative regulation of G1/S transition of mitotic cell cycle	8/210	86/18800	5.33E-06	4.13E-05	1.93E-05	CDK2/BCL2/CCND1/RB1/CDKN1A/TP53/CCL2/CHEK2	8
BP	GO:0031098	stress-activated protein kinase signaling cascade	13/210	252/18800	5.43E-06	4.18E-05	1.96E-05	MAPK14/MAPK10/NCF1/IKBKB/MAPK8/GSTP1/MAPK3/MAPK1/EGFR/VEGFA/MYC/IL1B/IL1A	13
BP	GO:0071560	cellular response to transforming growth factor beta stimulus	13/210	252/18800	5.43E-06	4.18E-05	1.96E-05	HSP90AB1/PPARG/JUN/IL4/PPARA/STAT3/FOS/TP53/HSPA5/CAV1/COL1A1/PARP1/COL3A1	13
BP	GO:0030073	insulin secretion	11/210	179/18800	5.60E-06	4.30E-05	2.01E-05	NOS2/PPARD/ALOX5/PPP3CA/BAD/SREBF1/ABAT/RAF1/HIF1A/IL1B/IFNG	11
BP	GO:0030324	lung development	11/210	179/18800	5.60E-06	4.30E-05	2.01E-05	PGR/KDR/LTA4H/CYP1A2/MAPK3/MAPK1/SREBF1/VEGFA/ELK1/NOS3/COL3A1	11
BP	GO:0000077	DNA damage checkpoint signalin	9/210	115/18800	5.77E-06	4.41E-05	2.06E-05	MAPK14/CHEK1/CDK2/CDK1/CCND1/CDKN1A/TP53/CHEK2/E2F1	9

		g							
BP	GO:0106106	cold-induced thermogenesis	10/210	146/18800	5.77E-06	4.41E-05	2.06E-05	ACHE/ADRB2/ADRB1/IL4/ADIPOR2/RB1/VEGFA/CAV1/GJA1/HSF1	10
BP	GO:0120161	regulation of cold-induced thermogenesis	10/210	146/18800	5.77E-06	4.41E-05	2.06E-05	ACHE/ADRB2/ADRB1/IL4/ADIPOR2/RB1/VEGFA/CAV1/GJA1/HSF1	10
BP	GO:0046688	response to copper ion	6/210	41/18800	5.87E-06	4.47E-05	2.09E-05	CDK1/CYP1A1/SOD1/IL1A/NFE2L2/HSF1	6
BP	GO:0150077	regulation of neuroinflammatory response	6/210	41/18800	5.87E-06	4.47E-05	2.09E-05	PTGS2/IL4/LDLR/MMP3/MMP9/IL1B	6
BP	GO:0043122	regulation of I-kappaB kinase/NF-kappaB signaling	13/210	254/18800	5.91E-06	4.49E-05	2.10E-05	ESR1/RELA/IKBKB/STAT1/HMOX1/GSTP1/CASP8/GJA1/IL1B/PRKCB/HSPB1/IL1A/CHUK	13
BP	GO:0043491	protein kinase B signaling	12/210	216/18800	5.92E-06	4.49E-05	2.10E-05	HSP90AB1/KDR/F7/AKT1/AKR1C3/PPARA/EGFR/EGF/F3/IL1B/CCL2/IGF2	12
BP	GO:0050820	positive regulation of coagulation	5/210	24/18800	5.93E-06	4.49E-05	2.10E-05	F7/F3/THBD/SERPINE1/NFE2L2	5
BP	GO:0050796	regulation of insulin secretio	10/210	147/18800	6.13E-06	4.63E-05	2.17E-05	NOS2/PPARD/ALOX5/PPP3CA/BAD/SREBF1/ABAT/HIF1A/IL1B/IFNG	10

		n							
BP	GO:0002683	negative regulation of immune system process	17/210	425/18800	6.15E-06	4.64E-05	2.17E-05	DPP4/PPARG/MAPK14/IL4/AKT1/TNFAIP6/CASP3/HMOX1/AHR/LDLR/CYP19A1/ERBB2/MYC/CCL2/IL2RA/COL3A1/IRF1	17
BP	GO:1901988	negative regulation of cell cycle phase transition	13/210	255/18800	6.17E-06	4.64E-05	2.17E-05	MAPK14/CHEK1/CDK2/BCL2/CDK1/CCND1/RB1/CDKN1A/TP53/CCL2/CCNB1/CHEK2/E2F1	13
BP	GO:0051052	regulation of DNA metabolic process	18/210	472/18800	6.28E-06	4.72E-05	2.21E-05	HSP90AB1/CHEK1/CCNA2/CDK2/IL4/AKT1/BAX/CDK1/CYP1B1/MAPK3/MAPK1/EGFR/CDKN1A/EGF/TP53/MYC/PARP1/HSF1	18
BP	GO:0010001	glial cell differentiation	12/210	218/18800	6.51E-06	4.88E-05	2.28E-05	DRD1/AKT1/CDK1/MAPK3/MAPK1/LDLR/SOD1/STAT3/ERBB2/IL1B/IFNG/ERBB3	12
BP	GO:0042326	negative regulation of phosphorylation	16/210	382/18800	6.54E-06	4.90E-05	2.29E-05	PPARG/JUN/AKT1/BAX/CASP3/GSTP1/HMGCR/PPARA/STAT3/RB1/CDKN1A/CAV1/IL1B/HSPB1/IFNG/IGFBP3	16
BP	GO:0002761	regulation of myeloid leukocyte differentiation	9/210	117/18800	6.64E-06	4.95E-05	2.32E-05	IL4/TNFAIP6/RB1/FOS/CASP8/PRKCA/MYC/IFNG/HSF1	9
BP	GO:00070	mitocho	9/210	117/1	6.64E-	4.95E-05	2.32E-05	GSK3B/BCL2/BA	9

	06	ndrial membrane organization		8800	06			X/MAPK8/BAD/BCL2L1/TP53/MYC/HK2	
BP	GO:0042752	regulation of circadian rhythm	9/210	117/18800	6.64E-06	4.95E-05	2.32E-05	PPARG/GSK3B/MAPK10/ADRB1/MAPK8/CDK1/PPARA/TP53/TOP2A	9
BP	GO:0003015	heart process	13/210	257/18800	6.71E-06	4.99E-05	2.34E-05	SCN5A/ADRA1A/CHRM2/ADRA1B/ADRA1D/ADRB1/GSTM2/SOD1/SREBF1/KCNH2/CAV1/GJA1/NOS3	13
BP	GO:0050999	regulation of nitric-oxide synthase activity	6/210	42/18800	6.78E-06	5.02E-05	2.35E-05	AKT1/EGFR/HIF1A/CAV1/IL1B/IL1A	6
BP	GO:0060964	regulation of miRNA-mediated gene silencing	6/210	42/18800	6.78E-06	5.02E-05	2.35E-05	ESR1/PPARG/PPP3CA/STAT3/EGFR/TP53	6
BP	GO:1900371	regulation of purine nucleotide biosynthetic process	6/210	42/18800	6.78E-06	5.02E-05	2.35E-05	NOS2/IL4/PPARA/MYC/NOS3/PARP1	6
BP	GO:0030323	respiratory tube development	11/210	183/18800	6.92E-06	5.09E-05	2.38E-05	PGR/KDR/LTA4H/CYP1A2/MAPK3/MAPK1/SREBF1/VEGFA/ELK1/NOS3/COL3A1	11
BP	GO:0090087	regulation of	11/210	183/18800	6.92E-06	5.09E-05	2.38E-05	NOS2/PPARD/ALOX5/PPP3CA/BA	11

		peptide transport						D/SREBF1/ABAT/CA2/HIF1A/IL1B/IFNG	
BP	GO:1901991	negative regulation of mitotic cell cycle phase transition	11/210	183/18800	6.92E-06	5.09E-05	2.38E-05	CHEK1/CDK2/BCL2/CDK1/CCND1/RB1/CDKN1A/TP53/CCL2/CCNB1/CHEK2	11
BP	GO:0031650	regulation of heat generation	4/210	12/18800	6.98E-06	5.13E-05	2.40E-05	PTGS2/ABAT/IL1B/PTGER3	4
BP	GO:0071559	response to transforming growth factor beta	13/210	258/18800	7.00E-06	5.13E-05	2.40E-05	HSP90AB1/PPARG/JUN/IL4/PPARA/STAT3/FOS/TP53/HSPA5/CAV1/COL1A1/PARP1/COL3A1	13
BP	GO:0019229	regulation of vasoconstriction	7/210	64/18800	7.11E-06	5.20E-05	2.43E-05	PTGS2/CHRM3/ADRA1A/ADRA1B/ADRA1D/MMP2/CAV1	7
BP	GO:0030278	regulation of ossification	9/210	118/18800	7.12E-06	5.20E-05	2.43E-05	ADRB2/MAPK14/BCL2/ALOX5/MAPK3/MAPK1/HIF1A/GJA1/RUNX2	9
BP	GO:0071346	cellular response to interferon-gamma	9/210	118/18800	7.12E-06	5.20E-05	2.43E-05	HSP90AB1/NOS2/PPARG/STAT1/TP53/MYC/CCL2/IFNG/IRF1	9
BP	GO:0030810	positive regulation of nucleotide biosynthetic	5/210	25/18800	7.34E-06	5.31E-05	2.49E-05	NOS2/IL4/PPARA/MYC/NOS3	5

		process							
BP	GO:0050927	positive regulation of positive chemotaxis	5/210	25/18800	7.34E-06	5.31E-05	2.49E-05	KDR/F7/VEGFA/F3/CXCL8	5
BP	GO:0071450	cellular response to oxygen radical	5/210	25/18800	7.34E-06	5.31E-05	2.49E-05	SOD1/NOS3/MPO/NFE2L2/NQO1	5
BP	GO:0071451	cellular response to superoxide	5/210	25/18800	7.34E-06	5.31E-05	2.49E-05	SOD1/NOS3/MPO/NFE2L2/NQO1	5
BP	GO:1900373	positive regulation of purine nucleotide biosynthetic process	5/210	25/18800	7.34E-06	5.31E-05	2.49E-05	NOS2/IL4/PPARA/MYC/NOS3	5
BP	GO:0046031	ADP metabolic process	8/210	90/18800	7.50E-06	5.42E-05	2.54E-05	BAD/PPARA/STAT3/EIF6/HIF1A/MYC/IFNG/HK2	8
BP	GO:0030808	regulation of nucleotide biosynthetic process	6/210	43/18800	7.81E-06	5.63E-05	2.64E-05	NOS2/IL4/PPARA/MYC/NOS3/PARP1	6
BP	GO:0006749	glutathione metabolic process	7/210	65/18800	7.89E-06	5.67E-05	2.65E-05	DPEP1/GSTP1/GSTM1/GSTM2/SOD1/GSR/NFE2L2	7
BP	GO:0031100	animal organ	7/210	65/18800	7.89E-06	5.67E-05	2.65E-05	CCNA2/F7/CDK1/HMOX1/UGT1A1/	7

		regeneration						CCND1/MYC	
BP	GO:0005996	monosaccharide metabolic process	13/210	261/18800	7.93E-06	5.68E-05	2.66E-05	NCOA2/MAPK14/GSK3B/PPARD/AKR1B1/AKT1/BAD/PPARA/TP53/MYC/IGF2/HK2/DGAT2	13
BP	GO:0050730	regulation of peptidyl-tyrosine phosphorylation	13/210	261/18800	7.93E-06	5.68E-05	2.66E-05	ADRA1A/NCF1/IL4/EGFR/VEGFA/EGF/IL6R/TP53/CAV1/IFNG/HSF1/IGF2/ERBB3	13
BP	GO:0016052	carbohydrate catabolic process	10/210	152/18800	8.25E-06	5.90E-05	2.76E-05	PYGM/BAD/PPARA/STAT3/EIF6/TP53/HIF1A/MYC/IFNG/HK2	10
BP	GO:0019932	second-messenger-mediated signaling	14/210	303/18800	8.35E-06	5.96E-05	2.79E-05	CHRM3/NOS2/KDR/GSK3B/SELE/VCAM1/AHR/PPP3CA/GSTM2/EGFR/VEGFA/CXCL8/NOS3/ERBB3	14
BP	GO:0033157	regulation of intracellular protein transport	12/210	224/18800	8.57E-06	6.11E-05	2.86E-05	PTGS2/CHRM1/HSP90AB1/MAPK14/GSK3B/MAPK8/CDK1/SREBF1/ERBB2/IL1B/IFNG/NPEPPS	12
BP	GO:0034605	cellular response to heat	7/210	66/18800	8.75E-06	6.22E-05	2.91E-05	PTGS2/HSP90AB1/GSK3B/HMOX1/IL1A/HSF1/CXCL10	7
BP	GO:1900407	regulation of cellular response to oxidative stress	8/210	92/18800	8.84E-06	6.27E-05	2.93E-05	AKT1/ALOX5/SOD1/MMP3/HIF1A/HSPB1/NFE2L2/PARP1	8
BP	GO:1903035	negative regulatio	8/210	92/18800	8.84E-06	6.27E-05	2.93E-05	ALOX5/ABAT/PLAU/NOS3/PLAT/T	8

		n of response to wounding						HBD/SERPINE1/SPP1	
BP	GO:0060147	regulation of post-transcriptional gene silencing	6/210	44/18800	8.96E-06	6.33E-05	2.96E-05	ESR1/PPARG/PPP3CA/STAT3/EGFR/TP53	6
BP	GO:1900368	regulation of post-transcriptional gene silencing by RNA	6/210	44/18800	8.96E-06	6.33E-05	2.96E-05	ESR1/PPARG/PPP3CA/STAT3/EGFR/TP53	6
BP	GO:0036296	response to increased oxygen levels	5/210	26/18800	9.01E-06	6.35E-05	2.97E-05	CYP1A1/CAT/MMP2/CAV1/COL1A1	5
BP	GO:0050926	regulation of positive chemotaxis	5/210	26/18800	9.01E-06	6.35E-05	2.97E-05	KDR/F7/VEGFA/F3/CXCL8	5
BP	GO:0051092	positive regulation of NF-kappaB transcription factor activity	10/210	154/18800	9.26E-06	6.51E-05	3.05E-05	AR/RELA/IKBKB/CAT/STAT3/CAV1/IL1B/PRKCB/CHUK/CD40LG	10
BP	GO:0071901	negative regulation of protein	9/210	122/18800	9.35E-06	6.57E-05	3.07E-05	PPARG/AKT1/CASP3/GSTP1/HMGCR/RB1/CDKN1A/CAV1/IL1B	9

		serine/threonine kinase activity							
BP	GO:0006936	muscle contraction	15/210	349/18800	9.43E-06	6.61E-05	3.09E-05	PTGS2/CHRM3/SCN5A/ADRA1A/CHRM2/ADRB2/ADRA1B/DRD1/GSTM2/SOD1/ABAT/KCNH2/CAV1/GJA1/PTGER3	15
BP	GO:0009117	nucleotide metabolic process	18/210	487/18800	9.62E-06	6.73E-05	3.15E-05	NOS2/IL4/ATP5F1B/BAD/HMGCR/PPARA/STAT3/EIF6/HIF1A/ACACA/MYC/NOS3/SULT1E1/IFNG/PARP1/ACP3/HK2/DGAT2	18
BP	GO:0044839	cell cycle G2/M phase transition	10/210	155/18800	9.80E-06	6.85E-05	3.20E-05	CHEK1/CCNA2/CDK2/CDK1/CCND1/CDK4/CDKN1A/TP53/CCNB1/CHEK2	10
BP	GO:0050731	positive regulation of peptidyl-tyrosine phosphorylation	11/210	190/18800	9.88E-06	6.89E-05	3.22E-05	ADRA1A/NCF1/IL4/VEGFA/EGF/IL6R/TP53/IFNG/HSF1/IGF2/ERBB3	11
BP	GO:0031570	DNA integrity checkpoint signaling	9/210	123/18800	9.99E-06	6.93E-05	3.24E-05	MAPK14/CHEK1/CDK2/CDK1/CCND1/CDKN1A/TP53/CHEK2/E2F1	9
BP	GO:1901989	positive regulation of cell cycle phase transitio	9/210	123/18800	9.99E-06	6.93E-05	3.24E-05	AKT1/CDK1/CYP1A1/CCND1/RB1/CDK4/EGFR/CCNB1/CHEK2	9

		n							
BP	GO:0010623	programmed cell death involved in cell development	4/210	13/18800	1.00E-05	6.93E-05	3.24E-05	BCL2/BAX/IL1B/IL1A	4
BP	GO:0061418	regulation of transcription from RNA polymerase II promoter in response to hypoxia	4/210	13/18800	1.00E-05	6.93E-05	3.24E-05	VEGFA/TP53/HIF1A/NFE2L2	4
BP	GO:0006110	regulation of glycolytic process	6/210	45/18800	1.02E-05	7.07E-05	3.31E-05	PPARA/STAT3/EIF6/HIF1A/MYC/IFNG	6
BP	GO:2000273	positive regulation of signaling receptor activity	6/210	45/18800	1.02E-05	7.07E-05	3.31E-05	ADRB2/NCF1/EGF/HIF1A/CCL2/IFNG	6
BP	GO:0008643	carbohydrate transport	10/210	156/18800	1.04E-05	7.15E-05	3.35E-05	MAPK14/DRD1/PPARD/AKT1/SLC2A4/MYC/IL1B/PRKCB/NFE2L2/HK2	10
BP	GO:0031348	negative regulation of defense response	13/210	268/18800	1.05E-05	7.25E-05	3.39E-05	PPARG/MAPK14/PPARD/IL4/TNFAIP6/ALOX5/GSTP1/LDLR/SOD1/CYP19A1/PPARA/RB1/IL2RA	13

BP	GO:0001963	synaptic transmission, dopaminergic	5/210	27/18800	1.10E-05	7.52E-05	3.52E-05	PTGS2/SLC6A2/SLC6A4/DRD1/SLC6A3	5
BP	GO:0006706	steroid catabolic process	5/210	27/18800	1.10E-05	7.52E-05	3.52E-05	CYP3A4/CYP1A2/CYP19A1/SULT1E1/SPP1	5
BP	GO:0043502	regulation of muscle adaptation	8/210	95/18800	1.12E-05	7.68E-05	3.59E-05	SCN5A/ADRA1A/PPARG/PPP3CA/PPARA/PRKCA/NOS3/PARP1	8
BP	GO:0043280	positive regulation of cysteine-type endopeptidase activity involved in apoptotic process	9/210	125/18800	1.14E-05	7.78E-05	3.64E-05	PPARG/BAX/BAD/CASP9/CASP8/MYC/F3/HSF1/CTSD	9
BP	GO:0051348	negative regulation of transferase activity	13/210	270/18800	1.14E-05	7.78E-05	3.64E-05	PPARG/GSK3B/AKT1/CASP3/GSTP1/HMGCR/RB1/CDKN1A/TP53/CAV1/IL1B/HSPB1/IFNG	13
BP	GO:0016241	regulation of macroautophagy	10/210	158/18800	1.16E-05	7.91E-05	3.70E-05	ADRB2/KDR/IL4/AKT1/CASP3/MAPK8/HMOX1/MAPK3/TP53/HIF1A	10
BP	GO:0060966	regulation of gene silencing by RNA	6/210	46/18800	1.17E-05	7.92E-05	3.71E-05	ESR1/PPARG/PPP3CA/STAT3/EGFR/TP53	6
BP	GO:00901	regulatio	6/210	46/18	1.17E-	7.92E-05	3.71E-05	AKT1/BAX/BAD/	6

	99	n of release of cytochrome c from mitochondria		800	05			BCL2L1/MMP9/TP53	
BP	GO:1901361	organic cyclic compound catabolic process	18/210	494/18800	1.17E-05	7.92E-05	3.71E-05	MAOB/MAPK14/SLC6A3/AKT1/BAX/HMOX1/CYP3A4/CYP1A2/CYP1A1/CYP19A1/UGT1A1/ABCC1/PRKCA/SULT1E1/NFE2L2/HSF1/SPP1/E2F1	18
BP	GO:0043254	regulation of protein-containing complex assembly	16/210	401/18800	1.20E-05	8.11E-05	3.80E-05	ESR1/GSK3B/OPRD1/BAX/MMP1/RB1/MMP3/VEGFA/TP53/RAF1/HSPA5/IFNG/PARP1/HSF1/RASA1/MAP2	16
BP	GO:0006753	nucleoside phosphate metabolic process	18/210	495/18800	1.20E-05	8.11E-05	3.80E-05	NOS2/IL4/ATP5F1B/BAD/HMGCR/PPARA/STAT3/EIF6/HIF1A/ACACA/MYC/NOS3/SULT1E1/IFNG/PARP1/ACP3/HK2/DGAT2	18
BP	GO:1990845	adaptive thermogenesis	10/210	159/18800	1.23E-05	8.28E-05	3.87E-05	ACHE/ADRB2/ADRB1/IL4/ADIPOR2/RB1/VEGFA/CAV1/GJA1/HSF1	10
BP	GO:0002262	myeloid cell homeostasis	10/210	160/18800	1.30E-05	8.73E-05	4.09E-05	MAPK14/BAX/CASP3/STAT1/HMOX1/SOD1/STAT3/RB1/VEGFA/HIF1A	10
BP	GO:0002532	production of	8/210	97/18800	1.31E-05	8.79E-05	4.11E-05	NOS2/MAPK14/NCF1/ALOX5/PPA	8

		molecular mediator involved in inflammatory response						RA/STAT3/HIF1A/SERPINE1	
BP	GO:1902807	negative regulation of cell cycle G1/S phase transition	8/210	97/18800	1.31E-05	8.79E-05	4.11E-05	CDK2/BCL2/CCND1/RB1/CDKN1A/TP53/CCL2/CHEK2	8
BP	GO:0000303	response to superoxide	5/210	28/18800	1.32E-05	8.87E-05	4.15E-05	SOD1/NOS3/MPO/NFE2L2/NQO1	5
BP	GO:1905953	negative regulation of lipid localization	6/210	47/18800	1.33E-05	8.87E-05	4.15E-05	PPARG/PPARD/AKT1/PPARA/CES1/NFKBIA	6
BP	GO:2000378	negative regulation of reactive oxygen species metabolic process	6/210	47/18800	1.33E-05	8.87E-05	4.15E-05	BCL2/PPARA/MMP3/TP53/HIF1A/HK2	6
BP	GO:1903532	positive regulation of secretion by cell	13/210	274/18800	1.33E-05	8.90E-05	4.17E-05	ACHE/SLC6A4/PPARG/PPARD/IL4/BAD/CYP19A1/ABAT/HIF1A/IL1B/IFNG/IL1A/SPP1	13
BP	GO:1990266	neutrophil migration	9/210	128/18800	1.38E-05	9.20E-05	4.30E-05	DPP4/TNFAIP6/IL1B/CCL2/CXCL8/IL1A/CXCL11/CXCL2/CXCL10	9

BP	GO:0042632	cholesterol homeostasis	8/210	98/18800	1.41E-05	9.39E-05	4.40E-05	LDLR/MTTP/APOB/SOAT2/CES1/SOAT1/CAV1/DGAT2	8
BP	GO:0003044	regulation of systemic arterial blood pressure mediated by a chemical signal	6/210	48/18800	1.50E-05	9.93E-05	4.65E-05	ADRA1A/ADRB2/ADRA1B/ADRA1D/ADRB1/NOS3	6
BP	GO:0006953	acute-phase response	6/210	48/18800	1.50E-05	9.93E-05	4.65E-05	PTGS2/UGT1A1/IL6R/IL1B/PTGER3/IL1A	6
BP	GO:0030195	negative regulation of blood coagulation	6/210	48/18800	1.50E-05	9.93E-05	4.65E-05	ABAT/PLAU/NOS3/PLAT/THBD/SERPINE1	6
BP	GO:0042311	vasodilation	6/210	48/18800	1.50E-05	9.93E-05	4.65E-05	ADRB2/DRD1/PPARD/ADRB1/SOD1/NOS3	6
BP	GO:0055092	sterol homeostasis	8/210	99/18800	1.52E-05	0.000100424	4.70E-05	LDLR/MTTP/APOB/SOAT2/CES1/SOAT1/CAV1/DGAT2	8
BP	GO:0071674	mononuclear cell migration	11/210	199/18800	1.53E-05	0.000100668	4.71E-05	IL4/AKT1/ICAM1/ALOX5/MAPK3/MAPK1/IL6R/CCL2/SERPINE1/CXCL11/CXCL10	11
BP	GO:1903036	positive regulation of response to wounding	7/210	72/18800	1.56E-05	0.000102958	4.82E-05	F7/F3/DUOX2/THBD/SERPINE1/NFE2L2/CLDN4	7
BP	GO:0000305	response to	5/210	29/18800	1.58E-05	0.000103738	4.85E-05	SOD1/NOS3/MPO/NFE2L2/NQO1	5

		oxygen radical							
BP	GO:0010575	positive regulation of vascular endothelial growth factor production	5/210	29/18800	1.58E-05	0.000103738	4.85E-05	PTGS2/CYP1B1/HIF1A/IL1B/IL1A	5
BP	GO:0031571	mitotic G1 DNA damage checkpoint signaling	5/210	29/18800	1.58E-05	0.000103738	4.85E-05	CDK2/CCND1/CDKN1A/TP53/CHEK2	5
BP	GO:0007179	transforming growth factor beta receptor signaling pathway	11/210	200/18800	1.60E-05	0.000104525	4.89E-05	HSP90AB1/PPARG/JUN/PPARA/STAT3/FOS/TP53/HSPA5/CAV1/PARP1/COL3A1	11
BP	GO:0034284	response to monosaccharide	11/210	200/18800	1.60E-05	0.000104525	4.89E-05	PTGS2/PPARD/CASP3/ICAM1/PPP3CA/BAD/CAT/SREBF1/RAF1/HIF1A/IL1A	11
BP	GO:0046661	male sex differentiation	10/210	164/18800	1.61E-05	0.000104824	4.91E-05	AR/ESR1/BCL2/BAX/CYP1B1/AKR1C3/CCND1/BCL2L1/GJA1/IL1A	10
BP	GO:1901992	positive regulation of mitotic cell cycle	8/210	100/18800	1.64E-05	0.000106629	4.99E-05	AKT1/CDK1/CYP1A1/CCND1/RB1/CDK4/EGFR/CCNB1	8

		phase transition							
BP	GO:0009150	purine ribonucleotide metabolic process	15/210	366/18800	1.65E-05	0.000107513	5.03E-05	IL4/ATP5F1B/BAD/HMGCR/PPARA/STAT3/EIF6/HIF1A/ACACA/MYC/SULT1E1/IFNG/PARP1/HK2/DGAT2	15
BP	GO:0060078	regulation of postsynaptic membrane potential	9/210	131/18800	1.66E-05	0.000107969	5.05E-05	CHRM1/ADRB2/OPRM1/GABRA1/GSK3B/ADRB1/AKT1/PPP3CA/ABAT	9
BP	GO:1900047	negative regulation of hemostasis	6/210	49/18800	1.69E-05	0.000109874	5.14E-05	ABAT/PLAU/NOS3/PLAT/THBD/SERPINE1	6
BP	GO:0033555	multicellular organismal response to stress	7/210	73/18800	1.71E-05	0.000110748	5.18E-05	DPP4/SLC6A2/DRD1/ADRB1/AKT1/BCL2/PPP3CA	7
BP	GO:0048662	negative regulation of smooth muscle cell proliferation	7/210	73/18800	1.71E-05	0.000110748	5.18E-05	PPARG/PPARD/HMOX1/CDKN1A/NOS3/IFNG/IGFBP3	7
BP	GO:0051146	striated muscle cell differentiation	13/210	281/18800	1.74E-05	0.000112371	5.26E-05	ADRA1A/MAPK14/IL4/AKT1/BCL2/CASP3/CDK1/PPP3CA/PPARA/RB1/VEGFA/CXCL10/IGF2	13
BP	GO:1902882	regulation of	8/210	101/18800	1.76E-05	0.000113313	5.30E-05	AKT1/ALOX5/SOD1/MMP3/HIF1A/	8

		response to oxidative stress						HSPB1/NFE2L2/PARP1	
BP	GO:1904029	regulation of cyclin-dependent protein kinase activity	8/210	101/18800	1.76E-05	0.000113313	5.30E-05	HSP90AB1/CCNA2/AKT1/CASP3/CCND1/EGFR/CDKN1A/CCNB1	8
BP	GO:0035270	endocrine system development	9/210	132/18800	1.77E-05	0.000113547	5.31E-05	GSK3B/SLC6A3/AKT1/CYP1B1/MAPK3/MAPK1/BAD/IL6R/RAF1	9
BP	GO:0060541	respiratory system development	11/210	203/18800	1.84E-05	0.000117921	5.52E-05	PGR/KDR/LTA4H/CYP1A2/MAPK3/MAPK1/SREBF1/VEGFA/ELK1/NOS3/COL3A1	11
BP	GO:0035924	cellular response to vascular endothelial growth factor stimulus	7/210	74/18800	1.87E-05	0.000119937	5.61E-05	KDR/MAPK14/RELA/AKT1/VCAM1/VEGFA/HSPB1	7
BP	GO:0002070	epithelial cell maturation	4/210	15/18800	1.88E-05	0.000119937	5.61E-05	PGR/AKR1B1/HIF1A/GJA1	4
BP	GO:0034698	response to gonadotropin	5/210	30/18800	1.88E-05	0.000120026	5.62E-05	CCNA2/CYP1B1/POR/MYC/PLAT	5
BP	GO:0044819	mitotic G1/S transition checkpoint signalin	5/210	30/18800	1.88E-05	0.000120026	5.62E-05	CDK2/CCND1/CDKN1A/TP53/CHEK2	5

		g							
BP	GO:0062014	negative regulation of small molecule metabolic process	8/210	102/18800	1.89E-05	0.000120332	5.63E-05	AKT1/AKR1C3/SOD1/PPARA/STAT3/TP53/PARP1/DGAT2	8
BP	GO:2001243	negative regulation of intrinsic apoptotic signaling pathway	8/210	102/18800	1.89E-05	0.000120332	5.63E-05	PTGS2/AKT1/BCL2/BCL2L1/MMP9/HIF1A/HSPB1/NFE2L2	8
BP	GO:0051251	positive regulation of lymphocyte activation	15/210	371/18800	1.94E-05	0.000122673	5.74E-05	DPP4/IL4/AKT1/BCL2/VCAM1/BAD/CDKN1A/CAV1/IL1B/CCL2/IL2RA/IFNG/IL1A/IGF2/CD40LG	15
BP	GO:0016485	protein processing	12/210	243/18800	1.94E-05	0.000122673	5.74E-05	DPP4/F7/CASP3/BAD/BACE2/CASP9/PLAU/CASP8/F3/PLAT/SERPINE1/PARP1	12
BP	GO:1901617	organic hydroxy compound biosynthetic process	12/210	243/18800	1.94E-05	0.000122673	5.74E-05	SLC6A3/CYP3A4/AKR1C3/SOD1/APOB/HMGCR/CYP19A1/SREBF1/GOT1/CES1/POR/IFNG	12
BP	GO:0006641	triglyceride metabolic process	8/210	103/18800	2.03E-05	0.000128078	5.99E-05	LDLR/CAT/MTTP/APOB/PLB1/SREBF1/CAV1/DGAT2	8
BP	GO:00091	purine	8/210	103/1	2.03E-	0.000128	5.99E-05	BAD/PPARA/STA	8

	35	nucleoside diphosphate metabolic process		8800	05	078		T3/EIF6/HIF1A/MYC/IFNG/HK2	
BP	GO:0009179	purine ribonucleoside diphosphate metabolic process	8/210	103/18800	2.03E-05	0.000128078	5.99E-05	BAD/PPARA/STAT3/EIF6/HIF1A/MYC/IFNG/HK2	8
BP	GO:0021543	pallium development	10/210	169/18800	2.08E-05	0.000131178	6.14E-05	GSK3B/DRD1/HSD3B2/HSD3B1/BAX/CASP3/BAD/EGFR/HIF1A/COL3A1	10
BP	GO:0009409	response to cold	6/210	51/18800	2.14E-05	0.00013419	6.28E-05	ADRB2/ADRB1/FOS/NFKBIA/CASP8/CXCL10	6
BP	GO:0051349	positive regulation of lyase activity	6/210	51/18800	2.14E-05	0.00013419	6.28E-05	ADRB2/NOS2/DRD1/ADRB1/RAF1/NOS3	6
BP	GO:0071622	regulation of granulocyte chemotaxis	6/210	51/18800	2.14E-05	0.00013419	6.28E-05	DPP4/IL4/TNFAIP6/MAPK3/MAPK1/CXCL8	6
BP	GO:0044262	cellular carbohydrate metabolic process	13/210	287/18800	2.17E-05	0.000135979	6.36E-05	NCOA2/GSK3B/PYGM/AKT1/HAS2/BAD/PPARA/GOT1/STAT3/TP53/IGF2/HK2/DGAT2	13
BP	GO:0000075	cell cycle checkpoint	10/210	170/18800	2.19E-05	0.000136821	6.40E-05	MAPK14/CHEK1/CDK2/CDK1/CCND1/CDKN1A/TP53/CCNB1/CHEK2/	10

		signaling						E2F1	
BP	GO:0072331	signal transduction by p53 class mediator	10/210	170/18800	2.19E-05	0.000136821	6.40E-05	CHEK1/AKT1/BCL2/BAX/CDKN1A/TP53/MYC/CHEK2/E2F1/E2F2	10
BP	GO:0060055	angiogenesis involved in wound healing	5/210	31/18800	2.22E-05	0.000138488	6.48E-05	KDR/ALOX5/ADIPOR2/VEGFA/SERPINE1	5
BP	GO:0061045	negative regulation of wound healing	7/210	76/18800	2.23E-05	0.000138956	6.50E-05	ALOX5/ABAT/PLAU/NOS3/PLAT/THBD/SERPINE1	7
BP	GO:0010508	positive regulation of autophagy	9/210	136/18800	2.24E-05	0.000139305	6.52E-05	ADRB2/KDR/GSK3B/IL4/HMOX1/MAPK3/BAD/HIF1A/IFNG	9
BP	GO:0044282	small molecule catabolic process	15/210	376/18800	2.26E-05	0.000140343	6.57E-05	NOS2/PPARD/AKT1/CYP3A4/CYP1A1/AKR1C3/BAD/PPARA/GOT1/ABAT/TP53/NOS3/SULT1E1/PON1/HK2	15
BP	GO:0002027	regulation of heart rate	8/210	105/18800	2.34E-05	0.000144783	6.78E-05	SCN5A/ADRA1A/ADRA1B/ADRA1D/ADRB1/SREBF1/KCNH2/CAV1	8
BP	GO:0006816	calcium ion transport	16/210	424/18800	2.38E-05	0.000146823	6.87E-05	PTGS2/ADRA1A/OPRM1/DRD1/OPRD1/BCL2/BAX/PPP3CA/GSTM2/EGF/CAV1/CCL2/PRKCB/NOS3/CXCL11/CXCL10	16
BP	GO:0002686	negative regulatio	6/210	52/18800	2.40E-05	0.000147892	6.92E-05	DPP4/AKT1/TNFAIP6/HMOX1/CY	6

		n of leukocyte migration						P19A1/CCL2	
BP	GO:0050819	negative regulation of coagulation	6/210	52/18800	2.40E-05	0.000147892	6.92E-05	ABAT/PLAU/NOS3/PLAT/THBD/SERPINE1	6
BP	GO:1903201	regulation of oxidative stress-induced cell death	7/210	77/18800	2.44E-05	0.000149882	7.01E-05	AKT1/SOD1/MMP3/HIF1A/HSPB1/NFE2L2/PARP1	7
BP	GO:0045651	positive regulation of macrophage differentiation	4/210	16/18800	2.48E-05	0.000152324	7.13E-05	RB1/CASP8/PRKCA/HSF1	4
BP	GO:0030593	neutrophil chemotaxis	8/210	106/18800	2.51E-05	0.000153551	7.19E-05	DPP4/TNFAIP6/IL1B/CCL2/CXCL8/CXCL11/CXCL2/CXCL10	8
BP	GO:0051341	regulation of oxidoreductase activity	8/210	106/18800	2.51E-05	0.000153551	7.19E-05	AKT1/EGFR/POR/HIF1A/CAV1/IL1B/IFNG/IL1A	8
BP	GO:0032770	positive regulation of monooxygenase activity	5/210	32/18800	2.61E-05	0.000159838	7.48E-05	AKT1/POR/HIF1A/IL1B/IFNG	5
BP	GO:0010827	regulation of glucose transme	7/210	78/18800	2.65E-05	0.000161942	7.58E-05	MAPK14/AKT1/MYC/IL1B/PRKCB/NFE2L2/HK2	7

		mbrane transport							
BP	GO:0051924	regulation of calcium ion transport	12/210	251/18800	2.67E-05	0.000162279	7.59E-05	PTGS2/DRD1/OPRD1/BCL2/BAX/GSTM2/EGF/CAV1/CCL2/NOS3/CXCL11/CXCL10	12
BP	GO:0010950	positive regulation of endopeptidase activity	10/210	174/18800	2.68E-05	0.000162279	7.59E-05	PPARG/BAX/BAD/STAT3/CASP9/CASP8/MYC/F3/HSF1/CTSD	10
BP	GO:0042129	regulation of T cell proliferation	10/210	174/18800	2.68E-05	0.000162279	7.59E-05	IL4/CASP3/VCAM1/ERBB2/IL1B/IL2RA/IL1A/IGF2/CD40LG/IRF1	10
BP	GO:0042770	signal transduction in response to DNA damage	10/210	174/18800	2.68E-05	0.000162279	7.59E-05	MAPK14/CHEK1/CDK2/CDK1/CCND1/CDKN1A/CASP9/TP53/CHEK2/E2F1	10
BP	GO:0002931	response to ischemia	6/210	53/18800	2.68E-05	0.000162279	7.59E-05	BCL2/CASP9/TP53/CAV1/NQO1/HK2	6
BP	GO:0034381	plasma lipoprotein particle clearance	6/210	53/18800	2.68E-05	0.000162279	7.59E-05	HMOX1/LDLR/APOB/SOAT2/SOAT1/DGAT2	6
BP	GO:0033209	tumor necrosis factor-mediated signaling pathway	8/210	107/18800	2.68E-05	0.000162279	7.59E-05	RELA/IKBKB/STAT1/GSTP1/TP53/NFKBIA/CASP8/CHUK	8
BP	GO:0000086	G2/M transition of mitotic	9/210	140/18800	2.82E-05	0.000170414	7.97E-05	CHEK1/CCNA2/CDK2/CDK1/CCND1/CDK4/CDKN1A/CCNB1/CHEK2	9

		cell cycle							
BP	GO:0034341	response to interferon-gamma	9/210	140/18800	2.82E-05	0.000170414	7.97E-05	HSP90AB1/NOS2/PPARG/STAT1/TP53/MYC/CCL2/IFNG/IRF1	9
BP	GO:0006469	negative regulation of protein kinase activity	11/210	213/18800	2.87E-05	0.000172617	8.08E-05	PPARG/AKT1/CASP3/GSTP1/HMGCR/RB1/CDKN1A/CAV1/IL1B/HSPB1/IFNG	11
BP	GO:1902749	regulation of cell cycle G2/M phase transition	8/210	108/18800	2.87E-05	0.000172617	8.08E-05	CHEK1/CDK2/CDK1/CCND1/CDK4/CDKN1A/TP53/CCNB1	8
BP	GO:0046323	glucose import	7/210	79/18800	2.88E-05	0.000172789	8.09E-05	MAPK14/DRD1/AKT1/SLC2A4/MYC/NFE2L2/HK2	7
BP	GO:0048708	astrocyte differentiation	7/210	79/18800	2.88E-05	0.000172789	8.09E-05	DRD1/MAPK3/MAPK1/LDLR/STAT3/IL1B/IFNG	7
BP	GO:0009259	ribonucleotide metabolic process	15/210	384/18800	2.89E-05	0.000172975	8.09E-05	IL4/ATP5F1B/BAD/HMGCR/PPARA/STAT3/EIF6/HIF1A/ACACA/MYC/SULT1E1/IFNG/PARP1/HK2/DGAT2	15
BP	GO:0010524	positive regulation of calcium ion transport into cytosol	6/210	54/18800	2.99E-05	0.00017852	8.35E-05	DRD1/BAX/GSTM2/CAV1/CXCL11/CXCL10	6
BP	GO:0050671	positive regulatio	9/210	141/18800	2.99E-05	0.00017852	8.35E-05	IL4/BCL2/VCAM1/CDKN1A/IL1B/IL	9

		n of lymphocyte proliferation						2RA/IL1A/IGF2/CD40LG	
BP	GO:0045907	positive regulation of vasoconstriction	5/210	33/18800	3.05E-05	0.000180943	8.47E-05	PTGS2/ADRA1A/ADRA1B/ADRA1D/CAV1	5
BP	GO:0045940	positive regulation of steroid metabolic process	5/210	33/18800	3.05E-05	0.000180943	8.47E-05	SREBF1/CES1/POR/IFNG/IL1A	5
BP	GO:0046685	response to arsenic-containing substance	5/210	33/18800	3.05E-05	0.000180943	8.47E-05	HMOX1/CYP1A1/CYP1B1/MYC/HSF1	5
BP	GO:1901099	negative regulation of signal transduction in absence of ligand	5/210	33/18800	3.05E-05	0.000180943	8.47E-05	AKT1/BCL2/BCL2L1/IL1B/IL1A	5
BP	GO:2001240	negative regulation of extrinsic apoptotic signaling pathway in absence	5/210	33/18800	3.05E-05	0.000180943	8.47E-05	AKT1/BCL2/BCL2L1/IL1B/IL1A	5

		of ligand							
BP	GO:0032526	response to retinoic acid	8/210	109/18800	3.07E-05	0.000181693	8.50E-05	RXRA/SLC6A4/GSK3B/SREBF1/RXRB/MMP2/MYC/COL1A1	8
BP	GO:0090276	regulation of peptide hormone secretion	10/210	177/18800	3.10E-05	0.000183566	8.59E-05	NOS2/PPARD/ALOX5/PPP3CA/BAD/SREBF1/ABAT/HIF1A/IL1B/IFNG	10
BP	GO:0001933	negative regulation of protein phosphorylation	14/210	341/18800	3.12E-05	0.000184149	8.62E-05	PPARG/JUN/AKT1/BAX/CASP3/GSTP1/HMGCR/RB1/CDKN1A/CAV1/IL1B/HSPB1/IFNG/IGFBP3	14
BP	GO:0045786	negative regulation of cell cycle	15/210	387/18800	3.16E-05	0.000186162	8.71E-05	PTGS2/MAPK14/CHEK1/CDK2/BCL2/CASP3/CDK1/CCND1/RB1/CDKN1A/TP53/CCL2/CCNB1/CHEK2/E2F1	15
BP	GO:0045598	regulation of fat cell differentiation	9/210	142/18800	3.16E-05	0.000186162	8.71E-05	PTGS2/PPARG/MAPK14/PPARD/AKT1/ALOX5/RUNX1T1/SULT1E1/E2F1	9
BP	GO:0051770	positive regulation of nitric-oxide synthase biosynthetic process	4/210	17/18800	3.21E-05	0.000188715	8.83E-05	KDR/STAT1/CCL2/IFNG	4
BP	GO:0071732	cellular response to nitric oxide	4/210	17/18800	3.21E-05	0.000188715	8.83E-05	DPEP1/CCNA2/CDK2/MMP3	4
BP	GO:00160	cell	17/210	484/1	3.27E-	0.000191	8.98E-05	ADRA1A/HSP90A	17

	49	growth		8800	05	923		B1/GSK3B/ESR2/PPARD/AKT1/BCL2/PPARA/RB1/EGFR/VEGFA/CDKN1A/TP53/ERBB2/GJA1/SPP1/MAP2	
BP	GO:0002763	positive regulation of myeloid leukocyte differentiation	6/210	55/18800	3.32E-05	0.000194641	9.11E-05	RB1/FOS/CASP8/PRKCA/IFNG/HSF1	6
BP	GO:0032946	positive regulation of mononuclear cell proliferation	9/210	143/18800	3.34E-05	0.000194876	9.12E-05	IL4/BCL2/VCAM1/CDKN1A/IL1B/IL2RA/IL1A/IGF2/CD40LG	9
BP	GO:1903531	negative regulation of secretion by cell	9/210	143/18800	3.34E-05	0.000194876	9.12E-05	OPRM1/MAOB/HMOX1/PPP3CA/HMGCR/SREBF1/ABAT/GJA1/IL1B	9
BP	GO:2001056	positive regulation of cysteine-type endopeptidase activity	9/210	143/18800	3.34E-05	0.000194876	9.12E-05	PPARG/BAX/BAD/CASP9/CASP8/MYC/F3/HSF1/CTSD	9
BP	GO:0034976	response to endoplasmic reticulum stress	12/210	257/18800	3.36E-05	0.00019595	9.17E-05	GSK3B/JUN/BCL2/BAX/ALOX5/CCND1/BCL2L1/TP53/HSPA5/CAV1/CXCL8/NFE2L2	12
BP	GO:0006096	glycolytic	7/210	81/18800	3.39E-05	0.000197003	9.22E-05	PPARA/STAT3/EIF6/HIF1A/MYC/IF	7

		process						NG/HK2	
BP	GO:0044773	mitotic DNA damage checkpoint signaling	7/210	81/18800	3.39E-05	0.000197003	9.22E-05	CHEK1/CDK2/CDK1/CCND1/CDKN1A/TP53/CHEK2	7
BP	GO:0051047	positive regulation of secretion	13/210	300/18800	3.45E-05	0.000200054	9.36E-05	ACHE/SLC6A4/PPARG/PPARD/IL4/BAD/CYP19A1/ABAT/HIF1A/IL1B/IFNG/IL1A/SPP1	13
BP	GO:0014065	phosphatidylinositol 3-kinase signaling	9/210	144/18800	3.53E-05	0.000204479	9.57E-05	KDR/PPARD/NCF1/AKT1/CAT/EGFR/EGF/ERBB2/ERBB3	9
BP	GO:0010638	positive regulation of organelle organization	17/210	487/18800	3.54E-05	0.000204511	9.57E-05	ADRB2/KDR/GSK3B/CDK2/BAX/MAPK8/MAPK3/MAPK1/BAD/RB1/MMP9/EGF/TP53/HIF1A/IL1B/IL1A/IGF2	17
BP	GO:0043276	anoikis	5/210	34/18800	3.54E-05	0.000204511	9.57E-05	AKT1/BCL2/CAV1/CHEK2/E2F1	5
BP	GO:2000352	negative regulation of endothelial cell apoptotic process	5/210	34/18800	3.54E-05	0.000204511	9.57E-05	KDR/IL4/ICAM1/SERPINE1/NFE2L2	5
BP	GO:0010948	negative regulation of cell cycle process	13/210	301/18800	3.57E-05	0.000205651	9.62E-05	MAPK14/CHEK1/CDK2/BCL2/CDK1/CCND1/RB1/CDKN1A/TP53/CCL2/CCNB1/CHEK2/E2F1	13
BP	GO:0002791	regulation of	10/210	180/18800	3.58E-05	0.000206152	9.65E-05	NOS2/PPARD/ALOX5/PPP3CA/BA	10

		peptide secretion						D/SREBF1/ABAT/HIF1A/IL1B/IFNG	
BP	GO:0006757	ATP generation from ADP	7/210	82/18800	3.67E-05	0.000211061	9.88E-05	PPARA/STAT3/EIF6/HIF1A/MYC/IFNG/HK2	7
BP	GO:0009185	ribonucleoside diphosphate metabolic process	8/210	112/18800	3.73E-05	0.000213846	0.000100069	BAD/PPARA/STAT3/EIF6/HIF1A/MYC/IFNG/HK2	8
BP	GO:0034440	lipid oxidation	8/210	112/18800	3.73E-05	0.000213846	0.000100069	MAPK14/PPARD/AKT1/ALOX5/PPARA/ADIPOR2/POR/DGAT2	8
BP	GO:0061448	connective tissue development	12/210	260/18800	3.77E-05	0.000215579	0.00010088	MAPK14/PPARD/MAPK3/RB1/CDK4/IL6R/POR/HIF1A/COL1A1/COL3A1/RUNX2/DGAT2	12
BP	GO:0030072	peptide hormone secretion	11/210	220/18800	3.86E-05	0.000220534	0.000103198	NOS2/PPARD/ALOX5/PPP3CA/BAD/SREBF1/ABAT/RAF1/HIF1A/IL1B/IFNG	11
BP	GO:0019693	ribose phosphate metabolic process	15/210	394/18800	3.88E-05	0.000221378	0.000103593	IL4/ATP5F1B/BAD/HMGCR/PPARA/STAT3/EIF6/HIF1A/ACACA/MYC/SULT1E1/IFNG/PARP1/HK2/DGAT2	15
BP	GO:0006839	mitochondrial transport	10/210	182/18800	3.94E-05	0.000224353	0.000104985	GSK3B/BCL2/BAX/MAPK8/BAD/SREBF1/BCL2L1/TP53/NPEPPS/HK2	10
BP	GO:0015844	monoamine transport	7/210	83/18800	3.97E-05	0.000225896	0.000105707	SLC6A2/MAOB/SLC6A4/DRD1/CHRM5/SLC6A3/ABAT	7

BP	GO:0048678	response to axon injury	7/210	83/18800	3.97E-05	0.000225896	0.000105707	JUN/BCL2/BAX/CDK1/SOD1/MMP2/SPP1	7
BP	GO:0046456	icosanoid biosynthetic process	6/210	57/18800	4.08E-05	0.000231814	0.000108476	PTGS2/PTGS1/LTA4H/ALOX5/AKR1C3/IL1B	6
BP	GO:0060965	negative regulation of miRNA-mediated gene silencing	4/210	18/18800	4.09E-05	0.000232166	0.000108641	ESR1/PPARG/PPP3CA/TP53	4
BP	GO:0048469	cell maturation	10/210	183/18800	4.12E-05	0.000233476	0.000109254	PGR/PPARG/KDR/AKR1B1/BCL2/RB1/VEGFA/HIF1A/GJA1/RUNX2	10
BP	GO:0051899	membrane depolarization	7/210	84/18800	4.29E-05	0.0002425	0.000113477	SCN5A/KDR/BCL2/KCNH2/CAV1/GJA1/PARP1	7
BP	GO:1900182	positive regulation of protein localization to nucleus	7/210	84/18800	4.29E-05	0.0002425	0.000113477	PTGS2/HSP90AB1/MAPK14/AKT1/CDK1/IFNG/PARP1	7
BP	GO:1904064	positive regulation of cation transmembrane transport	9/210	148/18800	4.38E-05	0.000246855	0.000115515	ADRB2/DRD1/BAX/GSTM2/KCNH2/CCL2/IFNG/CXCL11/CXCL10	9
BP	GO:0006695	cholesterol biosynthetic process	6/210	58/18800	4.51E-05	0.0002538	0.000118765	SOD1/APOB/HMGCR/SREBF1/CES1/POR	6
BP	GO:19026	secondar	6/210	58/18	4.51E-	0.000253	0.0001187	SOD1/APOB/HM	6

	53	y alcohol biosynthetic process		800	05	8	65	GCR/SREBF1/CES1/POR	
BP	GO:0046777	protein autophosphorylation	11/210	224/18800	4.55E-05	0.000255382	0.000119505	KDR/GSK3B/JUN/AKT1/INSRR/MAPK3/EGFR/VEGFA/ERBB2/CAV1/CHEK2	11
BP	GO:0010507	negative regulation of autophagy	7/210	85/18800	4.64E-05	0.000259715	0.000121533	ADRA1A/AKT1/BCL2/HMOX1/STAT3/IL10RA/TP53	7
BP	GO:0044774	mitotic DNA integrity checkpoint signaling	7/210	85/18800	4.64E-05	0.000259715	0.000121533	CHEK1/CDK2/CDK1/CCND1/CDKN1A/TP53/CHEK2	7
BP	GO:0030224	monocyte differentiation	5/210	36/18800	4.72E-05	0.000263506	0.000123307	PPARG/JUN/FASN/VEGFA/MYC	5
BP	GO:0042789	mRNA transcription by RNA polymerase II	5/210	36/18800	4.72E-05	0.000263506	0.000123307	RXRA/NCOA1/PPARG/SREBF1/STAT3	5
BP	GO:0030308	negative regulation of cell growth	10/210	186/18800	4.73E-05	0.000263826	0.000123456	ESR2/PPARD/BCL2/PPARA/RB1/CDKN1A/TP53/GJA1/SPP1/MAP2	10
BP	GO:0002790	peptide secretion	11/210	225/18800	4.73E-05	0.000263826	0.000123456	NOS2/PPARD/ALOX5/PPP3CA/BAD/SREBF1/ABAT/RAF1/HIF1A/IL1B/IFNG	11
BP	GO:1904892	regulation of receptor	8/210	116/18800	4.80E-05	0.000267118	0.000124997	PPARG/IL4/CYP1B1/IL10RA/EGF/IL6R/CAV1/IFNG	8

		signaling pathway via STAT							
BP	GO:0010675	regulation of cellular carbohydrate metabolic process	9/210	150/18800	4.86E-05	0.000270237	0.000126457	NCOA2/GSK3B/AKT1/BAD/PPARA/STAT3/TP53/IGF2/DGAT2	9
BP	GO:0070372	regulation of ERK1 and ERK2 cascade	13/210	311/18800	4.99E-05	0.000277155	0.000129694	ADRA1A/OPRM1/KDR/JUN/ICAM1/GSTP1/MAPK3/EGFR/PRKCA/ERBB2/IL1B/CCL2/IL1A	13
BP	GO:1904705	regulation of vascular associated smooth muscle cell proliferation	7/210	86/18800	5.00E-05	0.000277155	0.000129694	PPARG/JUN/HMOX1/CDKN1A/MMP2/MMP9/GJA1	7
BP	GO:0060149	negative regulation of post-transcriptional gene silencing	4/210	19/18800	5.14E-05	0.000282825	0.000132347	ESR1/PPARG/PPP3CA/TP53	4
BP	GO:0060967	negative regulation of gene silencing by RNA	4/210	19/18800	5.14E-05	0.000282825	0.000132347	ESR1/PPARG/PPP3CA/TP53	4

BP	GO:0097709	connective tissue replacement	4/210	19/18800	5.14E-05	0.000282825	0.000132347	PPARG/PPP3CA/HIF1A/IL1A	4
BP	GO:1900369	negative regulation of post-transcriptional gene silencing by RNA	4/210	19/18800	5.14E-05	0.000282825	0.000132347	ESR1/PPARG/PPP3CA/TP53	4
BP	GO:1902170	cellular response to reactive nitrogen species	4/210	19/18800	5.14E-05	0.000282825	0.000132347	DPEP1/CCNA2/CDK2/MMP3	4
BP	GO:1902894	negative regulation of miRNA transcription	4/210	19/18800	5.14E-05	0.000282825	0.000132347	PPARG/PPARD/RELA/PPARA	4
BP	GO:0006575	cellular modified amino acid metabolic process	10/210	188/18800	5.18E-05	0.000284568	0.000133163	DPEP1/GSTP1/DIO1/GSTM1/GSTM2/SOD1/GSR/POR/DUOX2/NFE2L2	10
BP	GO:0060537	muscle tissue development	15/210	405/18800	5.30E-05	0.000290964	0.000136155	SCN5A/ADRA1A/MAPK14/BCL2/CDK1/PPP3CA/PPARA/RB1/VEGFA/FOS/CAV1/GJA1/COL3A1/IGF2/ERBB3	15
BP	GO:0001892	embryonic placenta development	7/210	87/18800	5.39E-05	0.000294735	0.00013792	NCOA1/AKT1/MAPK1/EGFR/CASP8/HIF1A/IGF2	7

BP	GO:1990874	vascular associated smooth muscle cell proliferation	7/210	87/18800	5.39E-05	0.000294735	0.00013792	PPARG/JUN/HMOX1/CDKN1A/MMP2/MMP9/GJA1	7
BP	GO:0045454	cell redox homeostasis	5/210	37/18800	5.40E-05	0.000294943	0.000138018	NOS2/GSR/NOS3/NFE2L2/NQO1	5
BP	GO:1903580	positive regulation of ATP metabolic process	5/210	37/18800	5.40E-05	0.000294943	0.000138018	IL4/PPARA/HIF1A/MYC/IFNG	5
BP	GO:0008286	insulin receptor signaling pathway	8/210	118/18800	5.42E-05	0.000295582	0.000138316	GSK3B/AKT1/INSRR/SREBF1/CDK4/IL1B/PRKCB/IGF2	8
BP	GO:0090287	regulation of cellular response to growth factor stimulus	13/210	314/18800	5.51E-05	0.000299898	0.000140336	HSP90AB1/PPARG/KDR/IL4/TNFAIP6/PPARA/TP53/HIF1A/HSPA5/CAV1/IL1B/PRKCB/RUNX2	13
BP	GO:2000278	regulation of DNA biosynthetic process	8/210	119/18800	5.76E-05	0.000313081	0.000146505	HSP90AB1/CHEK1/CYP1B1/MAPK3/MAPK1/CDKN1A/TP53/MYC	8
BP	GO:0045428	regulation of nitric oxide biosynthetic	6/210	61/18800	6.02E-05	0.000326515	0.000152791	PTGS2/HSP90AB1/AKT1/CAV1/IL1B/IFNG	6

		process							
BP	GO:2001244	positive regulation of intrinsic apoptotic signaling pathway	6/210	61/18800	6.02E-05	0.000326515	0.000152791	BAX/BAD/SOD1/TP53/CAV1/MYC	6
BP	GO:0043271	negative regulation of ion transport	9/210	155/18800	6.27E-05	0.000339909	0.000159059	PTGS2/MAOB/BCL2/GSTM2/ABAT/KCNH2/MMP9/CAV1/NOS3	9
BP	GO:0042053	regulation of dopamine metabolic process	4/210	20/18800	6.37E-05	0.000342462	0.000160254	MAOB/DRD1/SLC6A3/ABAT	4
BP	GO:0042069	regulation of catecholamine metabolic process	4/210	20/18800	6.37E-05	0.000342462	0.000160254	MAOB/DRD1/SLC6A3/ABAT	4
BP	GO:0042953	lipoprotein transport	4/210	20/18800	6.37E-05	0.000342462	0.000160254	PPARG/MTTP/APOB/PRKCB	4
BP	GO:0071371	cellular response to gonadotropin stimulus	4/210	20/18800	6.37E-05	0.000342462	0.000160254	CCNA2/CYP1B1/POR/PLAT	4
BP	GO:0071379	cellular response to prostaglandin stimulus	4/210	20/18800	6.37E-05	0.000342462	0.000160254	AKT1/AKR1C3/APOB/ACACA	4
BP	GO:19021	negative	4/210	20/18	6.37E-	0.000342	0.0001602	AKT1/HIF1A/HSP	4

	76	regulation of oxidative stress-induced intrinsic apoptotic signaling pathway		800	05	462	54	B1/NFE2L2	
BP	GO:0022617	extracellular matrix disassembly	6/210	62/18800	6.60E-05	0.000354095	0.000165697	DPP4/PRSS1/MMP1/MMP3/MMP2/MMP9	6
BP	GO:0032757	positive regulation of interleukin-8 production	6/210	62/18800	6.60E-05	0.000354095	0.000165697	NOS2/RELA/STAT3/F3/IL1B/SERPINE1	6
BP	GO:0070301	cellular response to hydrogen peroxide	7/210	90/18800	6.69E-05	0.000358511	0.000167764	RELA/CDK1/CYP1B1/CAT/NFE2L2/NQO1/HSF1	7
BP	GO:0045930	negative regulation of mitotic cell cycle	11/210	234/18800	6.74E-05	0.000360715	0.000168795	CHEK1/CDK2/BCL2/CDK1/CCND1/RB1/CDKN1A/TP53/CCL2/CCNB1/CHEK2	11
BP	GO:1902107	positive regulation of leukocyte differentiation	9/210	157/18800	6.93E-05	0.000369731	0.000173014	IL4/BAD/RB1/FOS/CASP8/PRKCA/IL2RA/IFNG/HSF1	9
BP	GO:19037	positive	9/210	157/1	6.93E-	0.000369	0.0001730	IL4/BAD/RB1/FO	9

	08	regulation of hemopoiesis		8800	05	731	14	S/CASP8/PRKCA/IL2RA/IFNG/HSF1	
BP	GO:0001558	regulation of cell growth	15/210	415/18800	6.98E-05	0.00037113	0.000173669	GSK3B/ESR2/PPARD/AKT1/BCL2/PPARA/RB1/EGFR/VEGFA/CDKN1A/TP53/ERBB2/GJA1/SPP1/MAP2	15
BP	GO:0032885	regulation of polysaccharide biosynthetic process	5/210	39/18800	7.01E-05	0.00037113	0.000173669	GSK3B/AKT1/HAS2/EGF/IGF2	5
BP	GO:0042307	positive regulation of protein import into nucleus	5/210	39/18800	7.01E-05	0.00037113	0.000173669	PTGS2/HSP90AB1/MAPK14/CDK1/IFNG	5
BP	GO:0043029	T cell homeostasis	5/210	39/18800	7.01E-05	0.00037113	0.000173669	AKT1/BCL2/BAX/CASP3/IL2RA	5
BP	GO:0045923	positive regulation of fatty acid metabolic process	5/210	39/18800	7.01E-05	0.00037113	0.000173669	PTGS2/PPARG/PPARD/PPARA/IL1B	5
BP	GO:0071470	cellular response to osmotic stress	5/210	39/18800	7.01E-05	0.00037113	0.000173669	PTGS2/AKR1B1/CASP3/SLC2A4/BAD	5
BP	GO:0010573	vascular endothelial growth	6/210	63/18800	7.23E-05	0.000382447	0.000178965	PTGS2/CYP1B1/IL6R/HIF1A/IL1B/IL1A	6

		factor production							
BP	GO:0007292	female gamete generation	9/210	158/18800	7.28E-05	0.000383045	0.000179245	PTGS2/PGR/BCL2/TNFAIP6/MMP2/MYC/NOS3/PLAT/TOP2A	9
BP	GO:0033673	negative regulation of kinase activity	11/210	236/18800	7.28E-05	0.000383045	0.000179245	PPARG/AKT1/CASP3/GSTP1/HMGCR/RB1/CDKN1A/CAV1/IL1B/HSPB1/IFNG	11
BP	GO:0010906	regulation of glucose metabolic process	8/210	123/18800	7.28E-05	0.000383045	0.000179245	NCOA2/GSK3B/AKT1/BAD/PPARA/TP53/IGF2/DGAT2	8
BP	GO:0022612	gland morphogenesis	8/210	123/18800	7.28E-05	0.000383045	0.000179245	PGR/AR/ESR1/BCL2/BAX/EGFR/MMP2/CAV1	8
BP	GO:0044872	lipoprotein localization	4/210	21/18800	7.80E-05	0.000408998	0.000191389	PPARG/MTTP/APOB/PRKCB	4
BP	GO:0071498	cellular response to fluid shear stress	4/210	21/18800	7.80E-05	0.000408998	0.000191389	PTGS2/HAS2/MMP2/NFE2L2	4
BP	GO:0071731	response to nitric oxide	4/210	21/18800	7.80E-05	0.000408998	0.000191389	DPEP1/CCNA2/CDK2/MMP3	4
BP	GO:0031638	zymogen activation	6/210	64/18800	7.91E-05	0.000412977	0.000193251	BAD/CASP9/PLAU/CASP8/PLAT/SERPINE1	6
BP	GO:0050918	positive chemotaxis	6/210	64/18800	7.91E-05	0.000412977	0.000193251	KDR/F7/VEGFA/F3/CXCL8/CXCL10	6
BP	GO:0080164	regulation of nitric oxide	6/210	64/18800	7.91E-05	0.000412977	0.000193251	PTGS2/HSP90AB1/AKT1/CAV1/IL1B/IFNG	6

		metabolic process							
BP	GO:0007210	serotonin receptor signaling pathway	5/210	40/18800	7.94E-05	0.000413618	0.000193551	CHRM3/CHRM1/CHRM2/HTR3A/CHRM5	5
BP	GO:0051281	positive regulation of release of sequestered calcium ion into cytosol	5/210	40/18800	7.94E-05	0.000413618	0.000193551	DRD1/BAX/GSTM2/CXCL11/CXCL10	5
BP	GO:0002696	positive regulation of leukocyte activation	15/210	421/18800	8.19E-05	0.000426302	0.000199487	DPP4/IL4/AKT1/BCL2/VCAM1/BAD/CDKN1A/CAV1/IL1B/CCL2/IL2RA/IFNG/IL1A/IGF2/CD40LG	15
BP	GO:0002260	lymphocyte homeostasis	6/210	65/18800	8.63E-05	0.000446933	0.000209141	AKT1/BCL2/BAX/CASP3/HIF1A/IL2RA	6
BP	GO:0014823	response to activity	6/210	65/18800	8.63E-05	0.000446933	0.000209141	PPARD/CDK1/CAT/MMP2/HIF1A/HSF1	6
BP	GO:0016126	sterol biosynthetic process	6/210	65/18800	8.63E-05	0.000446933	0.000209141	SOD1/APOB/HMGCR/SREBF1/CES1/POR	6
BP	GO:1905330	regulation of morphogenesis of an epithelium	6/210	65/18800	8.63E-05	0.000446933	0.000209141	AR/ESR1/VEGFA/EGF/GJA1/CXCL10	6

BP	GO:0030900	forebrain development	14/210	376/18800	8.92E-05	0.000461566	0.000215988	SCN5A/GSK3B/DRD1/SLC6A3/HSD3B2/HSD3B1/BAX/CASP3/BAD/EGFR/HIF1A/MYC/COL3A1/E2F1	14
BP	GO:0045429	positive regulation of nitric oxide biosynthetic process	5/210	41/18800	8.96E-05	0.000462842	0.000216585	PTGS2/HSP90AB1/AKT1/IL1B/IFNG	5
BP	GO:0034767	positive regulation of ion transmembrane transport	9/210	163/18800	9.25E-05	0.000477338	0.000223368	ADRB2/DRD1/BAX/GSTM2/KCNH2/CCL2/IFNG/CXCL11/CXCL10	9
BP	GO:0003073	regulation of systemic arterial blood pressure	7/210	95/18800	9.45E-05	0.000486518	0.000227665	AR/ADRA1A/ADRB2/ADRA1B/ADRA1D/ADRB1/NOS3	7
BP	GO:1903798	regulation of production of miRNAs involved in gene silencing by miRNA	4/210	22/18800	9.45E-05	0.000486518	0.000227665	ESR1/PPP3CA/EGFR/TP53	4
BP	GO:0032735	positive regulation of interleukin-12 production	5/210	42/18800	0.000100771	0.000517504	0.000242164	MAPK14/RELA/IFNG/CD40LG/IRF1	5
BP	GO:19045	positive	5/210	42/18	0.0001	0.000517	0.0002421	PTGS2/HSP90AB1	5

	91	regulation of protein import		800	00771	504	64	/MAPK14/CDK1/IFNG	
BP	GO:0045600	positive regulation of fat cell differentiation	6/210	67/18800	0.000102346	0.000524956	0.000245651	PTGS2/PPARG/MAPK14/PPARD/AKT1/SULT1E1	6
BP	GO:0071695	anatomical structure maturation	11/210	246/18800	0.000105313	0.000539523	0.000252468	PGR/PPARG/KDR/AKR1B1/BCL2/RB1/VEGFA/MMP2/HIF1A/GJA1/RUNX2	11
BP	GO:0007093	mitotic cell cycle checkpoint signaling	8/210	130/18800	0.000107399	0.000548228	0.000256541	CHEK1/CDK2/CDK1/CCND1/CDKN1A/TP53/CCNB1/CHEK2	8
BP	GO:0009132	nucleoside diphosphate metabolic process	8/210	130/18800	0.000107399	0.000548228	0.000256541	BAD/PPARA/STAT3/EIF6/HIF1A/MYC/IFNG/HK2	8
BP	GO:0030168	platelet activation	8/210	130/18800	0.000107399	0.000548228	0.000256541	MAPK14/ABAT/PRKCA/NOS3/HSPB1/THBD/COL3A1/CD40LG	8
BP	GO:0010389	regulation of G2/M transition of mitotic cell cycle	7/210	97/18800	0.000107768	0.000549449	0.000257113	CHEK1/CDK2/CDK1/CCND1/CDK4/CDKN1A/CCNB1	7
BP	GO:0021537	telencephalon develop	11/210	247/18800	0.00010916	0.000555878	0.000260121	SCN5A/GSK3B/DRD1/HSD3B2/HSD3B1/BAX/CASP	11

		ment						3/BAD/EGFR/HIF1A/COL3A1	
BP	GO:0010611	regulation of cardiac muscle hypertrophy	6/210	68/18800	0.000111216	0.000565615	0.000264678	ADRA1A/PPARG/PPP3CA/PPARA/PRKCA/PARP1	6
BP	GO:0022900	electron transport chain	9/210	167/18800	0.000111338	0.000565615	0.000264678	MAOB/AKR1B1/NCF1/CDK1/CYP1A2/CYP19A1/GSR/POR/CCNB1	9
BP	GO:0014075	response to amine	5/210	43/18800	0.000113003	0.000568646	0.000266096	DRD1/CDK1/PPP3CA/SOD1/NQO1	5
BP	GO:0071364	cellular response to epidermal growth factor stimulus	5/210	43/18800	0.000113003	0.000568646	0.000266096	AKT1/EGFR/ERBB2/MYC/COL1A1	5
BP	GO:0140353	lipid export from cell	5/210	43/18800	0.000113003	0.000568646	0.000266096	NOS2/CYP19A1/IL1B/IL1A/SPP1	5
BP	GO:1904407	positive regulation of nitric oxide metabolic process	5/210	43/18800	0.000113003	0.000568646	0.000266096	PTGS2/HSP90AB1/AKT1/IL1B/IFNG	5
BP	GO:0006639	acylglycerol metabolic process	8/210	131/18800	0.000113303	0.000568646	0.000266096	LDLR/CAT/MTTP/APOB/PLB1/SREBF1/CAV1/DGAT2	8
BP	GO:0002689	negative regulation of leukocyte	4/210	23/18800	0.000113408	0.000568646	0.000266096	DPP4/TNFAIP6/CYP19A1/CCL2	4

		chemotaxis							
BP	GO:0010893	positive regulation of steroid biosynthetic process	4/210	23/18800	0.000113408	0.000568646	0.000266096	SREBF1/POR/IFNG/IL1A	4
BP	GO:0045540	regulation of cholesterol biosynthetic process	4/210	23/18800	0.000113408	0.000568646	0.000266096	SOD1/APOB/SREBF1/POR	4
BP	GO:0070920	regulation of production of small RNA involved in gene silencing by RNA	4/210	23/18800	0.000113408	0.000568646	0.000266096	ESR1/PPP3CA/EGFR/TP53	4
BP	GO:0106118	regulation of sterol biosynthetic process	4/210	23/18800	0.000113408	0.000568646	0.000266096	SOD1/APOB/SREBF1/POR	4
BP	GO:2001169	regulation of ATP biosynthetic process	4/210	23/18800	0.000113408	0.000568646	0.000266096	IL4/PPARA/MYC/PARP1	4
BP	GO:0000079	regulation of cyclin-dependent protein	7/210	98/18800	0.000114973	0.000575812	0.000269449	CCNA2/AKT1/CASP3/CCND1/EGFR/CDKN1A/CCNB1	7

		serine/threonine kinase activity							
BP	GO:0006638	neutral lipid metabolic process	8/210	132/18800	0.000119473	0.000596944	0.000279338	LDLR/CAT/MTTP/APOB/PLB1/SREBF1/CAV1/DGAT2	8
BP	GO:1900180	regulation of protein localization to nucleus	8/210	132/18800	0.000119473	0.000596944	0.000279338	PTGS2/HSP90AB1/MAPK14/GSK3B/AKT1/CDK1/IFNG/PARP1	8
BP	GO:0050867	positive regulation of cell activation	15/210	436/18800	0.000120644	0.000602086	0.000281744	DPP4/IL4/AKT1/BCL2/VCAM1/BAD/CDKN1A/CAV1/IL1B/CCL2/IL2RA/IFNG/IL1A/IGF2/CD40LG	15
BP	GO:0006165	nucleoside diphosphate phosphorylation	7/210	99/18800	0.000122564	0.000610232	0.000285556	PPARA/STAT3/EIF6/HIF1A/MYC/IFNG/HK2	7
BP	GO:0019233	sensory perception of pain	7/210	99/18800	0.000122564	0.000610232	0.000285556	PTGS2/OPRM1/OPRD1/MAPK3/CCL2/IL1A/ACP3	7
BP	GO:0034101	erythrocyte homeostasis	8/210	133/18800	0.000125918	0.000625377	0.000292643	MAPK14/CASP3/STAT1/HMOX1/STAT3/RB1/VEGFA/HIF1A	8
BP	GO:0002066	columnar/cuboidal epithelial cell development	5/210	44/18800	0.000126342	0.000625377	0.000292643	GSK3B/AKT1/FASN/BAD/HIF1A	5
BP	GO:0045840	positive regulatio	5/210	44/18800	0.000126342	0.000625377	0.000292643	RB1/EGF/IL1B/IL1A/IGF2	5

		n of mitotic nuclear division							
BP	GO:0060443	mammary gland morphogenesis	5/210	44/18800	0.000126342	0.000625377	0.000292643	PGR/AR/ESR1/BAX/CAV1	5
BP	GO:2001239	regulation of extrinsic apoptotic signaling pathway in absence of ligand	5/210	44/18800	0.000126342	0.000625377	0.000292643	AKT1/BCL2/BCL2L1/IL1B/IL1A	5
BP	GO:0031214	biomineral tissue development	9/210	170/18800	0.000127489	0.000630321	0.000294956	PTGS2/ADRB2/ALOX5/PPARA/HIF1A/GJA1/NOS3/COL1A1/SPP1	9
BP	GO:0031341	regulation of cell killing	7/210	100/18800	0.000130557	0.000643242	0.000301003	HSP90AB1/NOS2/IL4/MAPK8/ICAM1/BCL2L1/IFNG	7
BP	GO:0042100	B cell proliferation	7/210	100/18800	0.000130557	0.000643242	0.000301003	IL4/BCL2/BAX/CASP3/AHR/CDKN1A/CD40LG	7
BP	GO:0046939	nucleotide phosphorylation	7/210	100/18800	0.000130557	0.000643242	0.000301003	PPARA/STAT3/EIF6/HIF1A/MYC/IFNG/HK2	7
BP	GO:0001889	liver development	8/210	134/18800	0.000132647	0.000652783	0.000305468	RELA/JUN/HMOX1/CYP1A1/UGT1A1/CCND1/ELK1/MYC	8
BP	GO:0042730	fibrinolysis	4/210	24/18800	0.000134904	0.00066159	0.000309588	PLAU/PLAT/THBD/SERPINE1	4
BP	GO:0045649	regulation of macroph	4/210	24/18800	0.000134904	0.00066159	0.000309588	RB1/CASP8/PRKCA/HSF1	4

		age differentiation							
BP	GO:1901863	positive regulation of muscle tissue development	4/210	24/18800	0.000134904	0.00066159	0.000309588	BCL2/GJA1/IGF2/ERBB3	4
BP	GO:0032963	collagen metabolic process	7/210	101/18800	0.000138968	0.00067995	0.0003181 8	PPARD/MMP1/MMP3/MMP2/MMP9/HIF1A/COL1A1	7
BP	GO:0046620	regulation of organ growth	7/210	101/18800	0.000138968	0.00067995	0.0003181 8	SLC6A4/MAPK14/AKT1/CDK1/SOD1/PPARA/IGF2	7
BP	GO:0110148	biomineralization	9/210	172/18800	0.000139309	0.000680836	0.000318595	PTGS2/ADRB2/ALOX5/PPARA/HIF1A/GJA1/NOS3/COL1A1/SPP1	9
BP	GO:0006984	ER-nucleus signaling pathway	5/210	45/18800	0.000140854	0.000685231	0.000320651	GSK3B/SREBF1/TP53/HSPA5/NFE2L2	5
BP	GO:0032881	regulation of polysaccharide metabolic process	5/210	45/18800	0.000140854	0.000685231	0.000320651	GSK3B/AKT1/HAS2/EGF/IGF2	5
BP	GO:0048538	thymus development	5/210	45/18800	0.000140854	0.000685231	0.000320651	BCL2/MAPK3/MAPK1/SOD1/RAF1	5
BP	GO:0071827	plasma lipoprotein particle organization	5/210	45/18800	0.000140854	0.000685231	0.000320651	MTTP/APOB/SOAT2/SOAT1/MPO	5
BP	GO:00147	regulatio	6/210	71/18	0.0001	0.000687	0.0003218	ADRA1A/PPARG/	6

	43	n of muscle hypertrophy		800	41542	792	5	PPP3CA/PPARA/PRKCA/PARP1	
BP	GO:0001505	regulation of neurotransmitter levels	10/210	213/18800	0.000146038	0.000708826	0.000331692	ACHE/ADRA1A/SLC6A2/MAOB/SLC6A4/GSK3B/DRD1/SLC6A3/ABAT/PRKCB	10
BP	GO:0043255	regulation of carbohydrate biosynthetic process	7/210	102/18800	0.000147813	0.000716623	0.000335341	GSK3B/AKT1/HAS2/PPARA/EGF/IGF2/DGAT2	7
BP	GO:0060485	mesenchyme development	12/210	301/18800	0.000151863	0.000735421	0.000344138	GSK3B/BCL2/STAT1/HAS2/MAPK3/MAPK1/HIF1A/MYC/IL1B/NOS3/COL1A1/ERBB3	12
BP	GO:0061008	hepaticobiliary system development	8/210	137/18800	0.000154639	0.000747587	0.000349831	RELA/JUN/HMOX1/CYP1A1/UGT1A1/CCND1/ELK1/MYC	8
BP	GO:0001542	ovulation from ovarian follicle	3/210	10/18800	0.000155608	0.000747587	0.000349831	PGR/MMP2/NOS3	3
BP	GO:0032025	response to cobalt ion	3/210	10/18800	0.000155608	0.000747587	0.000349831	CASP3/CASP9/CASP8	3
BP	GO:0033079	immature T cell proliferation	3/210	10/18800	0.000155608	0.000747587	0.000349831	ERBB2/IL1B/IL1A	3
BP	GO:0033197	response to vitamin E	3/210	10/18800	0.000155608	0.000747587	0.000349831	CAT/CCND1/COL1A1	3
BP	GO:0033483	gas homeostasis	3/210	10/18800	0.000155608	0.000747587	0.000349831	GSTP1/HIF1A/CAV1	3

BP	GO:0097267	omega-hydroxylase P450 pathway	3/210	10/18800	0.000155608	0.000747587	0.000349831	CYP1A2/CYP1A1/CYP1B1	3
BP	GO:0010522	regulation of calcium ion transport into cytosol	7/210	103/18800	0.000157109	0.000753094	0.000352408	DRD1/BCL2/BAX/GSTM2/CAV1/CXCL11/CXCL10	7
BP	GO:0042102	positive regulation of T cell proliferation	7/210	103/18800	0.000157109	0.000753094	0.000352408	IL4/VCAM1/IL1B/IL2RA/IL1A/IGF2/CD40LG	7
BP	GO:0035821	modulation of process of another organism	4/210	25/18800	0.000159201	0.000762262	0.000356698	NOS2/SLPI/BCL2L1/IFNG	4
BP	GO:0001822	kidney development	12/210	303/18800	0.000161502	0.000772409	0.000361446	AKR1B1/BCL2/BAX/STAT1/HAS2/CAT/VEGFA/CASP9/MMP9/IL6R/ODC1/MYC	12
BP	GO:0045861	negative regulation of proteolysis	13/210	350/18800	0.00016325	0.000779892	0.000364948	PTGS2/HSP90AB1/DPEP1/AKT1/SLPI/VEGFA/MMP9/TP53/POR/RAF1/BIRC5/PLAT/SERPINE1	13
BP	GO:0050866	negative regulation of cell activation	10/210	216/18800	0.000163672	0.000781029	0.000365479	IL4/CASP3/HMOX1/LDLR/ABAT/ERBB2/NOS3/IL2RA/THBD/IRF1	10
BP	GO:0016239	positive regulation of macroau	6/210	73/18800	0.000165154	0.000787218	0.000368376	ADRB2/KDR/IL4/HMOX1/MAPK3/HIF1A	6

		tophagy							
BP	GO:0048568	embryonic organ development	15/210	449/18800	0.000166267	0.000791634	0.000370442	NCOA1/KDR/AKT1/MAPK3/MAPK1/SOD1/EGFR/VEGFA/TP53/CASP8/HIF1A/GJA1/CXCL8/RUNX2/IGF2	15
BP	GO:0046822	regulation of nucleocytoplasmic transport	7/210	104/18800	0.000166873	0.000793626	0.000371375	PTGS2/HSP90AB1/MAPK14/GSK3B/CDK1/IL1B/IFNG	7
BP	GO:0002831	regulation of response to biotic stimulus	13/210	351/18800	0.000167887	0.000797555	0.000373213	HSP90AB1/PPARG/NCF1/IL4/IKBKB/STAT1/AHR/MAPK3/CASP8/PRKCA/IL1B/NFE2L2/IRF1	13
BP	GO:0032409	regulation of transporter activity	12/210	305/18800	0.00017166	0.000814568	0.000381174	CHRM3/RXRA/ADRB2/OPRM1/PPARG/BCL2/GSTM2/MMP9/CAV1/CCL2/IFNG/PON1	12
BP	GO:1904062	regulation of cation transmembrane transport	13/210	352/18800	0.000172636	0.00081773	0.000382654	SCN5A/ADRB2/OPRM1/DRD1/BAX/GSTM2/KCNH2/MMP9/CAV1/CCL2/IFNG/CXCL11/CXCL10	13
BP	GO:0048015	phosphatidylinositol-mediated signaling	9/210	177/18800	0.000172925	0.00081773	0.000382654	KDR/PPARD/NCF1/AKT1/CAT/EGFR/EGF/ERBB2/ERBB3	9
BP	GO:0002673	regulation of acute inflammatory response	5/210	47/18800	0.000173674	0.00081773	0.000382654	PTGS2/IL4/GSTP1/IL1B/PTGER3	5
BP	GO:0003254	regulation of	5/210	47/18800	0.000173674	0.00081773	0.000382654	SCN5A/KDR/BCL2/GJA1/PARP1	5

		membrane depolarization							
BP	GO:0031294	lymphocyte costimulation	5/210	47/18800	0.000173674	0.00081773	0.000382654	DPP4/IL4/AKT1/CAV1/CD40LG	5
BP	GO:0046717	acid secretion	5/210	47/18800	0.000173674	0.00081773	0.000382654	CHRM5/ABAT/CES1/MYC/PTGER3	5
BP	GO:0051972	regulation of telomerase activity	5/210	47/18800	0.000173674	0.00081773	0.000382654	HSP90AB1/MAPK3/MAPK1/TP53/MYC	5
BP	GO:0050890	cognition	12/210	306/18800	0.00017694	0.000832119	0.000389387	PTGS2/CHRM1/SLC6A4/DRD1/CASP3/SLC2A4/MAPK1/LDLR/HMGCR/EGFR/FOS/HIF1A	12
BP	GO:0046425	regulation of receptor signaling pathway via JAK-STAT	7/210	105/18800	0.000177122	0.000832119	0.000389387	IL4/CYP1B1/IL10RA/EGF/IL6R/CAV1/IFNG	7
BP	GO:0030198	extracellular matrix organization	12/210	307/18800	0.000182359	0.000855775	0.000400457	DPP4/PRSS1/MMP1/CYP1B1/HAS2/RB1/MMP3/MMP2/MMP9/CAV1/COL1A1/COL3A1	12
BP	GO:0002026	regulation of the force of heart contraction	4/210	26/18800	0.000186509	0.000871399	0.000407768	ADRA1A/ADRB1/CAV1/NOS3	4
BP	GO:0033598	mammary gland	4/210	26/18800	0.000186509	0.000871399	0.000407768	ESR1/BAX/MAPK1/CCND1	4

		epithelial cell proliferation							
BP	GO:0034377	plasma lipoprotein particle assembly	4/210	26/18800	0.000186509	0.000871399	0.000407768	MTTP/APOB/SOAT2/SOAT1	4
BP	GO:0090200	positive regulation of release of cytochrome c from mitochondria	4/210	26/18800	0.000186509	0.000871399	0.000407768	BAX/BAD/MMP9/TP53	4
BP	GO:0006090	pyruvate metabolic process	7/210	106/18800	0.000187874	0.000875098	0.000409499	PPARA/STAT3/EIF6/HIF1A/MYC/IFNG/HK2	7
BP	GO:0019395	fatty acid oxidation	7/210	106/18800	0.000187874	0.000875098	0.000409499	MAPK14/PPARD/AKT1/PPARA/ADIPOR2/POR/DGAT2	7
BP	GO:0043062	extracellular structure organization	12/210	308/18800	0.000187918	0.000875098	0.000409499	DPP4/PRSS1/MMP1/CYP1B1/HAS2/RB1/MMP3/MMP2/MMP9/CAV1/COL1A1/COL3A1	12
BP	GO:0034763	negative regulation of transmembrane transport	8/210	141/18800	0.000188552	0.000877089	0.000410431	AKT1/GSTM2/KCNH2/MMP9/CAV1/MYC/IL1B/PRKCB	8
BP	GO:0070374	positive regulation of ERK1 and	10/210	220/18800	0.000189948	0.000882615	0.000413016	ADRA1A/OPRM1/KDR/JUN/ICAM1/MAPK3/EGFR/PRKCA/CCL2/IL1A	10

		ERK2 cascade							
BP	GO:0002534	cytokine production involved in inflammatory response	6/210	75/18800	0.000191777	0.000886898	0.000415021	NOS2/MAPK14/ALOX5/PPARA/STAT3/HIF1A	6
BP	GO:0071230	cellular response to amino acid stimulus	6/210	75/18800	0.000191777	0.000886898	0.000415021	EGFR/BCL2L1/MMP2/COL1A1/COL3A1/HSF1	6
BP	GO:1900015	regulation of cytokine production involved in inflammatory response	6/210	75/18800	0.000191777	0.000886898	0.000415021	NOS2/MAPK14/ALOX5/PPARA/STAT3/HIF1A	6
BP	GO:0060324	face development	5/210	48/18800	0.000192123	0.000886898	0.000415021	MAPK3/MAPK1/MMP2/RAF1/COL1A1	5
BP	GO:0061028	establishment of endothelial barrier	5/210	48/18800	0.000192123	0.000886898	0.000415021	IKBKB/ICAM1/FASN/VEGFA/IL1B	5
BP	GO:0071825	protein-lipid complex subunit organization	5/210	48/18800	0.000192123	0.000886898	0.000415021	MTTP/APOB/SOAT2/SOAT1/MPO	5
BP	GO:0007611	learning or memory	11/210	264/18800	0.000195439	0.000901226	0.000421726	PTGS2/SLC6A4/DRD1/CASP3/SLC2A4/MAPK1/LDLR/HMGCR/EGFR/FOS/HIF1A	11

BP	GO:0055123	digestive system development	8/210	142/18800	0.000197922	0.000911688	0.000426621	BCL2/CYP1A1/RB1/EGFR/HIF1A/CXCL8/COL3A1/IGF2	8
BP	GO:0045229	external encapsulating structure organization	12/210	310/18800	0.000199473	0.000917837	0.000429499	DPP4/PRSS1/MMP1/CYP1B1/HAS2/RB1/MMP3/MMP2/MMP9/CAV1/COL1A1/COL3A1	12
BP	GO:0010721	negative regulation of cell development	9/210	181/18800	0.000204457	0.000938735	0.000439278	GSK3B/PPP3CA/LDLR/VEGFA/TP53/IL1B/IL1A/SPP1/MAP2	9
BP	GO:0048017	inositol lipid-mediated signaling	9/210	181/18800	0.000204457	0.000938735	0.000439278	KDR/PPARD/NCF1/AKT1/CAT/EGFR/EGF/ERBB2/ERBB3	9
BP	GO:0046165	alcohol biosynthetic process	8/210	143/18800	0.000207672	0.000952464	0.000445702	CYP3A4/SOD1/APOB/HMGCR/SREBF1/GOT1/CES1/POR	8
BP	GO:0021761	limbic system development	7/210	108/18800	0.000210961	0.000963866	0.000451038	GSK3B/DRD1/HSD3B2/HSD3B1/BAX/CASP3/MYC	7
BP	GO:0072001	renal system development	12/210	312/18800	0.00021163	0.000963866	0.000451038	AKR1B1/BCL2/BAX/STAT1/HAS2/CAT/VEGFA/CASP9/MMP9/IL6R/ODC1/MYC	12
BP	GO:0050873	brown fat cell differentiation	5/210	49/18800	0.00021203	0.000963866	0.000451038	PTGS2/ADRB2/MAPK14/ADRB1/SLC2A4	5
BP	GO:0010918	positive regulation of mitochondrial membrane	3/210	11/18800	0.0002122	0.000963866	0.000451038	AKT1/BAD/MYC	3

		potential							
BP	GO:0042135	neurotransmitter catabolic process	3/210	11/18800	0.0002122	0.000963866	0.000451038	ACHE/MAOB/ABAT	3
BP	GO:0042447	hormone catabolic process	3/210	11/18800	0.0002122	0.000963866	0.000451038	CYP19A1/SULT1E1/SPP1	3
BP	GO:0043619	regulation of transcription from RNA polymerase II promoter in response to oxidative stress	3/210	11/18800	0.0002122	0.000963866	0.000451038	HMOX1/HIF1A/NFE2L2	3
BP	GO:0072584	caveolin-mediated endocytosis	3/210	11/18800	0.0002122	0.000963866	0.000451038	MAPK3/MAPK1/CAV1	3
BP	GO:1903799	negative regulation of production of miRNAs involved in gene silencing by miRNA	3/210	11/18800	0.0002122	0.000963866	0.000451038	ESR1/PPP3CA/TP53	3
BP	GO:0007263	nitric oxide mediated signal	4/210	27/18800	0.000217039	0.000981646	0.000459358	NOS2/EGFR/VEGFA/NOS3	4

		transduction							
BP	GO:0009651	response to salt stress	4/210	27/18800	0.000217039	0.000981646	0.000459358	HSP90AB1/AKR1B1/BAX/TP53	4
BP	GO:0010460	positive regulation of heart rate	4/210	27/18800	0.000217039	0.000981646	0.000459358	ADRA1A/ADRA1B/ADRA1D/ADRB1	4
BP	GO:0042537	benzene-containing compound metabolic process	4/210	27/18800	0.000217039	0.000981646	0.000459358	CYP1B1/GSTM1/GSTM2/UGT1A1	4
BP	GO:0006919	activation of cysteine-type endopeptidase activity involved in apoptotic process	6/210	77/18800	0.000221675	0.001000483	0.000468172	PPARG/BAX/BAD/CASP9/CASP8/F3	6
BP	GO:0051339	regulation of lyase activity	6/210	77/18800	0.000221675	0.001000483	0.000468172	ADRB2/NOS2/DRD1/ADRB1/RAF1/NOS3	6
BP	GO:0043405	regulation of MAP kinase activity	9/210	183/18800	0.000221933	0.001000585	0.0004682 2	PPARG/GSTP1/HMGCR/EGFR/VEGFA/EGF/ERBB2/CAV1/IL1B	9
BP	GO:0006766	vitamin metabolic process	7/210	109/18800	0.000223333	0.001004764	0.000470176	PPARD/CYP3A4/CYP1A1/AKR1C3/ABCC1/IFNG/ACP3	7
BP	GO:00718	leukocyt	7/210	109/1	0.0002	0.001004	0.0004701	AKT1/BAX/CASP	7

	87	e apoptotic process		8800	23333	764	76	3/CASP9/TP53/HIF1A/IL2RA	
BP	GO:0051604	protein maturation	12/210	314/18800	0.000224413	0.001008551	0.000471948	DPP4/F7/CASP3/BAD/BACE2/CASP9/PLAU/CASP8/F3/PLAT/SERPINE1/PARP1	12
BP	GO:0050729	positive regulation of inflammatory response	8/210	145/18800	0.000228354	0.001025181	0.00047973	PTGS2/LDLR/ABCC1/NFKBIA/IL1B/PTGER3/SERPINE1/IFNG	8
BP	GO:0043457	regulation of cellular respiration	5/210	50/18800	0.000233469	0.001045929	0.000489439	NOS2/IL4/HIF1A/MYC/IFNG	5
BP	GO:0070231	T cell apoptotic process	5/210	50/18800	0.000233469	0.001045929	0.000489439	AKT1/BAX/TP53/HIF1A/IL2RA	5
BP	GO:0007193	adenylate cyclase-inhibiting G protein-coupled receptor signaling pathway	6/210	78/18800	0.00023794	0.001063715	0.000497762	CHRM3/CHRM1/CHRM2/OPRM1/CHRM5/OPRD1	6
BP	GO:0033077	T cell differentiation in thymus	6/210	78/18800	0.00023794	0.001063715	0.000497762	BCL2/SOD1/TP53/ERBB2/IL1B/IL1A	6
BP	GO:0019058	viral life cycle	12/210	317/18800	0.000244819	0.001093318	0.000511614	HSP90AB1/BCL2/CDK1/ICAM1/SLPI/LDLR/PPARA/EGFR/CAV1/CCL2/CXCL8/TOP2A	12

BP	GO:0007088	regulation of mitotic nuclear division	7/210	111/18800	0.000249834	0.001112207	0.000520453	CHEK1/RB1/EGF/IL1B/CCNB1/IL1A/IGF2	7
BP	GO:0014066	regulation of phosphatidylinositol 3-kinase signaling	7/210	111/18800	0.000249834	0.001112207	0.000520453	KDR/PPARD/NCF1/CAT/EGFR/EGF/ERBB3	7
BP	GO:0048640	negative regulation of developmental growth	7/210	111/18800	0.000249834	0.001112207	0.000520453	ADRB2/SLC6A4/ADRB1/PPARA/CDKN1A/SPP1/MAP2	7
BP	GO:0071322	cellular response to carbohydrate stimulus	8/210	147/18800	0.000250697	0.001112773	0.000520718	PPARD/ICAM1/PPP3CA/BAD/RAF1/HIF1A/MYC/PRKCB	8
BP	GO:0002360	T cell lineage commitment	4/210	28/18800	0.000251009	0.001112773	0.000520718	BCL2/STAT3/IL6R/TP53	4
BP	GO:0010971	positive regulation of G2/M transition of mitotic cell cycle	4/210	28/18800	0.000251009	0.001112773	0.000520718	CDK1/CCND1/CDK4/CCNB1	4
BP	GO:0090312	positive regulation of protein deacetylation	4/210	28/18800	0.000251009	0.001112773	0.000520718	SREBF1/VEGFA/TP53/IFNG	4

BP	GO:0014068	positive regulation of phosphatidylinositol 3-kinase signaling	6/210	79/18800	0.000255129	0.001129856	0.000528712	KDR/PPARD/NCF1/CAT/EGF/ERBB3	6
BP	GO:0015872	dopamine transport	5/210	51/18800	0.000256517	0.001134821	0.000531035	SLC6A2/DRD1/CHRM5/SLC6A3/ABAT	5
BP	GO:0043123	positive regulation of I-kappaB kinase/NF-kappaB signaling	9/210	188/18800	0.000271123	0.001198188	0.000560688	RELA/IKBKB/HMOX1/CASP8/GJA1/IL1B/PRKCB/IL1A/CHUK	9
BP	GO:0018958	phenol-containing compound metabolic process	7/210	113/18800	0.00027881	0.001228893	0.000575056	MAOB/DRD1/SLC6A3/BCL2/DIO1/ABAT/DUOX2	7
BP	GO:0006787	porphyrin-containing compound catabolic process	3/210	12/18800	0.000280606	0.001228893	0.000575056	HMOX1/UGT1A1/ABCC1	3
BP	GO:0006983	ER overload response	3/210	12/18800	0.000280606	0.001228893	0.000575056	GSK3B/TP53/HSPA5	3
BP	GO:0033015	tetrapyrrole cataboli	3/210	12/18800	0.000280606	0.001228893	0.000575056	HMOX1/UGT1A1/ABCC1	3

		c process							
BP	GO:0051583	dopamine uptake involved in synaptic transmission	3/210	12/18800	0.000280606	0.001228893	0.000575056	SLC6A2/DRD1/SLC6A3	3
BP	GO:0051934	catecholamine uptake involved in synaptic transmission	3/210	12/18800	0.000280606	0.001228893	0.000575056	SLC6A2/DRD1/SLC6A3	3
BP	GO:0060736	prostate gland growth	3/210	12/18800	0.000280606	0.001228893	0.000575056	AR/ESR1/CYP19A1	3
BP	GO:0090205	positive regulation of cholesterol metabolic process	3/210	12/18800	0.000280606	0.001228893	0.000575056	SREBF1/CES1/POR	3
BP	GO:0045333	cellular respiration	10/210	231/18800	0.000281135	0.001228893	0.000575056	NOS2/IL4/ATP5F1B/CDK1/CYP1A2/CAT/HIF1A/MYC/CCNB1/IFNG	10
BP	GO:0035196	production of miRNAs involved in gene silencing by miRNA	5/210	52/18800	0.000281254	0.001228893	0.000575056	ESR1/PPP3CA/STAT3/EGFR/TP53	5
BP	GO:0043525	positive regulation of neuron	5/210	52/18800	0.000281254	0.001228893	0.000575056	BAX/CASP3/CASP9/TP53/NQO1	5

		apoptotic process							
BP	GO:0051346	negative regulation of hydrolase activity	13/210	371/18800	0.000287519	0.001254978	0.000587263	PTGS2/DPEP1/GSK3B/IKBKB/AKT1/SLPI/VEGFA/MMP9/POR/RAF1/BIRC5/NOS3/SERPINE1	13
BP	GO:0002068	glandular epithelial cell development	4/210	29/18800	0.000288638	0.00125599	0.000587736	GSK3B/AKT1/FASN/BAD	4
BP	GO:0030212	hyaluronan metabolic process	4/210	29/18800	0.000288638	0.00125599	0.000587736	TNFAIP6/HAS2/EGF/IL1B	4
BP	GO:0065005	protein-lipid complex assembly	4/210	29/18800	0.000288638	0.00125599	0.000587736	MTTP/APOB/SOAT2/SOAT1	4
BP	GO:0010660	regulation of muscle cell apoptotic process	6/210	81/18800	0.000292429	0.001271184	0.000594846	PPARG/HMOX1/TP53/IFNG/NFE2L2/HSF1	6
BP	GO:0003014	renal system process	7/210	114/18800	0.000294278	0.001277912	0.000597995	ADRA1A/AKR1B1/BCL2/HAS2/AKR1C3/ABCG2/CLDN4	7
BP	GO:0007204	positive regulation of cytosolic calcium ion concentr	12/210	325/18800	0.000307118	0.001332311	0.00062345	ADRA1A/ADRA1B/ADRA1D/ESR1/DRD1/BCL2/BAX/GSTM2/CAV1/PTGER3/CXCL11/CXCL10	12

		ation							
BP	GO:0006778	porphyrin-containing compound metabolic process	5/210	53/18800	0.000307759	0.001332366	0.000623476	HMOX1/CYP1A2/CYP1A1/UGT1A1/ABCC1	5
BP	GO:0048260	positive regulation of receptor-mediated endocytosis	5/210	53/18800	0.000307759	0.001332366	0.000623476	IL4/SELE/VEGFA/EGF/SERPINE1	5
BP	GO:1905897	regulation of response to endoplasmic reticulum stress	6/210	82/18800	0.000312616	0.001352017	0.000632672	BAX/ALOX5/BCL2L1/HSPA5/CAV1/NFE2L2	6
BP	GO:0071675	regulation of mononuclear cell migration	7/210	116/18800	0.000327282	0.001414004	0.000661678	IL4/AKT1/MAPK3/MAPK1/CCL2/SERPINE1/CXCL10	7
BP	GO:0035051	cardiocyte differentiation	8/210	153/18800	0.000328698	0.001414874	0.000662085	ADRA1A/CDK1/VCAM1/MAPK3/MAPK1/PPARA/EGFR/VEGFA	8
BP	GO:0048754	branching morphogenesis of an epithelial tube	8/210	153/18800	0.000328698	0.001414874	0.000662085	PGR/AR/ESR1/KDR/BCL2/VEGFA/EGF/MYC	8
BP	GO:00097	response	9/210	193/1	0.0003	0.001414	0.0006620	PTGS2/PPARD/C	9

	46	to hexose		8800	29026	874	85	ASP3/ICAM1/PPP3CA/BAD/SREBF1/RAF1/HIF1A	
BP	GO:0007190	activation of adenylate cyclase activity	4/210	30/18800	0.000330148	0.001414874	0.000662085	ADRB2/DRD1/ADRB1/RAF1	4
BP	GO:0048147	negative regulation of fibroblast proliferation	4/210	30/18800	0.000330148	0.001414874	0.000662085	BAX/GSTP1/TP53/MYC	4
BP	GO:0060251	regulation of glial cell proliferation	4/210	30/18800	0.000330148	0.001414874	0.000662085	CHRM1/MYC/IL1B/E2F1	4
BP	GO:0070498	interleukin-1-mediated signaling pathway	4/210	30/18800	0.000330148	0.001414874	0.000662085	RELA/IKBKB/MAPK3/IL1B	4
BP	GO:1903579	negative regulation of ATP metabolic process	4/210	30/18800	0.000330148	0.001414874	0.000662085	PPARA/STAT3/TP53/PARP1	4
BP	GO:0060562	epithelial tube morphogenesis	12/210	328/18800	0.000333718	0.001428732	0.0006685 7	PGR/AR/ESR1/KDR/BCL2/CASP3/VEGFA/EGF/HIF1A/MYC/GJA1/CXCL10	12
BP	GO:0061900	glial cell activation	5/210	54/18800	0.000336114	0.001437538	0.000672691	JUN/IL4/LDLR/IL1B/IFNG	5
BP	GO:0050804	modulation of	14/210	429/18800	0.000347507	0.00148477	0.000694793	PTGS2/ACHE/ADRA1A/ADRB2/SL	14

		chemical synaptic transmission						C6A4/GSK3B/DRD1/PPP3CA/MAPK1/CA2/IL1B/CCL2/PRKCB/PLAT	
BP	GO:0001654	eye development	13/210	379/18800	0.000352603	0.001505028	0.000704273	ACHE/KDR/SLC6A3/JUN/BCL2/BAX/CYP1A1/CYP1B1/STAT3/EGFR/VEGFA/HIF1A/GJA1	13
BP	GO:0099177	regulation of trans-synaptic signaling	14/210	430/18800	0.000355716	0.001516794	0.000709778	PTGS2/ACHE/ADRA1A/ADRB2/SLC6A4/GSK3B/DRD1/PPP3CA/MAPK1/CA2/IL1B/CCL2/PRKCB/PLAT	14
BP	GO:0071277	cellular response to calcium ion	6/210	84/18800	0.00035626	0.001517589	0.00071015	SCN5A/DPEP1/JUN/AKR1C3/FOS/HSPA5	6
BP	GO:0010745	negative regulation of macrophage derived foam cell differentiation	3/210	13/18800	0.00036179	0.001539599	0.00072045	PPARG/PPARA/NFKBIA	3
BP	GO:0032368	regulation of lipid transport	7/210	118/18800	0.000363191	0.001544015	0.000722516	PPARG/AKT1/CYP19A1/PPARA/IL1B/IL1A/SPP1	7
BP	GO:0001782	B cell homeostasis	4/210	31/18800	0.000375765	0.001587933	0.000743067	BCL2/BAX/CASP3/HIF1A	4
BP	GO:0034694	response to prostaglandin	4/210	31/18800	0.000375765	0.001587933	0.000743067	AKT1/AKR1C3/APOB/ACACA	4
BP	GO:00457	positive	4/210	31/18	0.0003	0.001587	0.0007430	AKT1/CCND1/EG	4

	37	regulation of cyclin-dependent protein serine/threonine kinase activity		800	75765	933	67	FR/CCNB1	
BP	GO:0045987	positive regulation of smooth muscle contraction	4/210	31/18800	0.000375765	0.001587933	0.000743067	PTGS2/CHRM3/ADRA1A/ABAT	4
BP	GO:0048384	retinoic acid receptor signaling pathway	4/210	31/18800	0.000375765	0.001587933	0.000743067	RXRA/PPARG/AKR1C3/RXRB	4
BP	GO:1902751	positive regulation of cell cycle G2/M phase transition	4/210	31/18800	0.000375765	0.001587933	0.000743067	CDK1/CCND1/CDK4/CCNB1	4
BP	GO:0010657	muscle cell apoptotic process	6/210	85/18800	0.000379797	0.001603378	0.000750295	PPARG/HMOX1/TP53/IFNG/NFE2L2/HSF1	6
BP	GO:0051897	positive regulation of protein kinase B signaling	7/210	119/18800	0.000382292	0.001610708	0.000753725	HSP90AB1/F7/AKR1C3/EGFR/EGF/F3/IGF2	7
BP	GO:0051928	positive regulatio	7/210	119/18800	0.000382292	0.001610708	0.000753725	DRD1/BAX/GSTM2/CAV1/CCL2/C	7

		n of calcium ion transport						XCL11/CXCL10	
BP	GO:0150063	visual system development	13/210	383/18800	0.000389598	0.001639865	0.000767369	ACHE/KDR/SLC6A3/JUN/BCL2/BAX/CYP1A1/CYP1B1/STAT3/EGFR/VEGFA/HIF1A/GJA1	13
BP	GO:0032872	regulation of stress-activated MAPK cascade	9/210	198/18800	0.000396793	0.001668495	0.000780766	NCF1/GSTP1/MAPK3/MAPK1/EGFR/VEGFA/MYC/IL1B/IL1A	9
BP	GO:0070918	primary sncRNA processing	5/210	56/18800	0.0003 9871	0.001674896	0.000783761	ESR1/PPP3CA/STAT3/EGFR/TP53	5
BP	GO:0034103	regulation of tissue remodeling	6/210	86/18800	0.000404533	0.001697679	0.000794423	PPARG/PPP3CA/TP53/PRKCA/GJA1/SPP1	6
BP	GO:0022408	negative regulation of cell-cell adhesion	9/210	199/18800	0.000411638	0.001725793	0.000807579	IL4/AKT1/CASP3/PPARA/ABAT/VEGFA/ERBB2/IL2RA/IRF1	9
BP	GO:0042113	B cell activation	12/210	336/18800	0.000414384	0.001735592	0.000812164	IL4/BCL2/BAX/CASP3/VCAM1/AHR/BAD/CDKN1A/TP53/CASP8/PRKCB/CD40LG	12
BP	GO:0009308	amine metabolic process	7/210	121/18800	0.000422905	0.001769534	0.000828047	MAOB/DRD1/SLC6A3/CYP1A1/VCAM1/ABAT/ODC1	7
BP	GO:0001516	prostaglandin biosynthetic process	4/210	32/18800	0.000425714	0.00177082	0.000828649	PTGS2/PTGS1/AKR1C3/IL1B	4

BP	GO:0010165	response to X-ray	4/210	32/18800	0.000425714	0.00177082	0.000828649	CASP3/CCND1/TP53/THBD	4
BP	GO:0010743	regulation of macrophage derived foam cell differentiation	4/210	32/18800	0.000425714	0.00177082	0.000828649	PPARG/APOB/PPARA/NFKBIA	4
BP	GO:0046320	regulation of fatty acid oxidation	4/210	32/18800	0.000425714	0.00177082	0.000828649	PPARD/AKT1/PPARA/DGAT2	4
BP	GO:0046457	prostanoid biosynthetic process	4/210	32/18800	0.000425714	0.00177082	0.000828649	PTGS2/PTGS1/AKR1C3/IL1B	4
BP	GO:0071353	cellular response to interleukin-4	4/210	32/18800	0.000425714	0.00177082	0.000828649	HSP90AB1/FASN/CDK4/HSPA5	4
BP	GO:0042692	muscle cell differentiation	13/210	387/18800	0.000429848	0.001786267	0.000835877	ADRA1A/MAPK14/IL4/AKT1/BCL2/CASP3/CDK1/PPP3CA/PPARA/RB1/VEGFA/CXCL10/IGF2	13
BP	GO:0009791	post-embryonic development	6/210	87/18800	0.000430509	0.001787265	0.000836344	KDR/BCL2/BAX/CYP1A2/APOB/VEGFA	6
BP	GO:0006754	ATP biosynthetic process	5/210	57/18800	0.000433121	0.001794601	0.000839777	IL4/ATP5F1B/PPARA/MYC/PARP1	5
BP	GO:0042306	regulation of protein	5/210	57/18800	0.000433121	0.001794601	0.000839777	PTGS2/HSP90AB1/MAPK14/CDK1/IFNG	5

		import into nucleus							
BP	GO:0070302	regulation of stress-activated protein kinase signaling cascade	9/210	201/18800	0.000442704	0.001832517	0.00085752	NCF1/GSTP1/MAPK3/MAPK1/EGFR/VEGFA/MYC/IL1B/IL1A	9
BP	GO:0046887	positive regulation of hormone secretion	7/210	122/18800	0.000444465	0.001838016	0.000860093	PPARG/PPARD/BAD/CYP19A1/ABAT/HIF1A/SPP1	7
BP	GO:0048880	sensory system development	13/210	389/18800	0.000451264	0.001864318	0.000872401	ACHE/KDR/SLC6A3/JUN/BCL2/BAX/CYP1A1/CYP1B1/STAT3/EGFR/VEGFA/HIF1A/GJA1	13
BP	GO:0010867	positive regulation of triglyceride biosynthetic process	3/210	14/18800	0.000456676	0.001873916	0.000876892	LDLR/SREBF1/DGAT2	3
BP	GO:0030213	hyaluronan biosynthetic process	3/210	14/18800	0.000456676	0.001873916	0.000876892	HAS2/EGF/IL1B	3
BP	GO:0035672	oligopeptide transmembrane transport	3/210	14/18800	0.000456676	0.001873916	0.000876892	ABCC1/CA2/GJA1	3
BP	GO:0044849	estrous cycle	3/210	14/18800	0.000456676	0.001873916	0.000876892	OPRM1/CYP1B1/HAS2	3

BP	GO:1901857	positive regulation of cellular respiration	3/210	14/18800	0.000456676	0.001873916	0.000876892	IL4/MYC/IFNG	3
BP	GO:1902947	regulation of tau-protein kinase activity	3/210	14/18800	0.000456676	0.001873916	0.000876892	HSP90AB1/RB1/IFNG	3
BP	GO:2001171	positive regulation of ATP biosynthetic process	3/210	14/18800	0.000456676	0.001873916	0.000876892	IL4/PPARA/MYC	3
BP	GO:0016570	histone modification	15/210	494/18800	0.000458683	0.001879595	0.00087955	NCOA1/CHEK1/CCNA2/CDK2/DRD1/MAPK8/CDK1/MAPK3/SREBF1/VEGFA/TP53/PRKCA/IL1B/PRKCB/IGF2	15
BP	GO:0019722	calcium-mediated signaling	9/210	202/18800	0.000458945	0.001879595	0.00087955	CHRM3/KDR/GSK3B/SELE/VCAM1/PPP3CA/GSTM2/CXCL8/ERBB3	9
BP	GO:0006898	receptor-mediated endocytosis	10/210	246/18800	0.0004621 9	0.00188924	0.000884063	ACHE/ADRB2/IL4/SELE/LDLR/VEGFA/EGF/CAV1/CXCL8/SERPINE1	10
BP	GO:2001020	regulation of response to DNA damage stimulus	10/210	246/18800	0.0004621 9	0.00188924	0.000884063	CHEK1/BCL2/EGFR/BCL2L1/CASP9/TP53/MYC/PARP1/CHEK2/HSF1	10
BP	GO:0001952	regulation of	7/210	123/18800	0.000466894	0.001906633	0.000892202	KDR/GSK3B/BCL2/VEGFA/PLAU/S	7

		cell-matrix adhesion						ERPINE1/RASA1	
BP	GO:0001893	maternal placenta development	4/210	33/18800	0.000480226	0.001949826	0.000912414	PTGS2/PPARD/AKT1/SPP1	4
BP	GO:0045648	positive regulation of erythrocyte differentiation	4/210	33/18800	0.000480226	0.001949826	0.000912414	MAPK14/STAT1/STAT3/HIF1A	4
BP	GO:0051930	regulation of sensory perception of pain	4/210	33/18800	0.000480226	0.001949826	0.000912414	OPRM1/OPRD1/IL1A/ACP3	4
BP	GO:0051931	regulation of sensory perception	4/210	33/18800	0.000480226	0.001949826	0.000912414	OPRM1/OPRD1/IL1A/ACP3	4
BP	GO:0055094	response to lipoprotein particle	4/210	33/18800	0.000480226	0.001949826	0.000912414	PPARG/AKT1/LDLR/CES1	4
BP	GO:2000191	regulation of fatty acid transport	4/210	33/18800	0.000480226	0.001949826	0.000912414	AKT1/PPARA/IL1B/IL1A	4
BP	GO:0001776	leukocyte homeostasis	6/210	89/18800	0.000486356	0.001970947	0.000922297	AKT1/BCL2/BAX/CASP3/HIF1A/IL2RA	6
BP	GO:0070098	chemokine-mediated signaling	6/210	89/18800	0.000486356	0.001970947	0.000922297	HIF1A/CCL2/CXCL8/CXCL11/CXCL2/CXCL10	6

		pathway							
BP	GO:0030218	erythrocyte differentiation	7/210	124/18800	0.000490217	0.001984701	0.000928734	MAPK14/CASP3/STAT1/STAT3/RB1/VEGFA/HIF1A	7
BP	GO:0050680	negative regulation of epithelial cell proliferation	8/210	163/18800	0.000501962	0.002030316	0.000950079	AR/PPARG/PPARD/STAT1/ALOX5/RB1/CAV1/CCL2	8
BP	GO:0010574	regulation of vascular endothelial growth factor production	5/210	59/18800	0.000508611	0.002049402	0.00095901	PTGS2/CYP1B1/HIF1A/IL1B/IL1A	5
BP	GO:0051353	positive regulation of oxidoreductase activity	5/210	59/18800	0.000508611	0.002049402	0.00095901	AKT1/POR/HIF1A/IL1B/IFNG	5
BP	GO:0051785	positive regulation of nuclear division	5/210	59/18800	0.000508611	0.002049402	0.00095901	RB1/EGF/IL1B/IL1A/IGF2	5
BP	GO:0071384	cellular response to corticosteroid stimulus	5/210	59/18800	0.000508611	0.002049402	0.00095901	CYP1B1/AKR1C3/UGT1A1/CASP9/PLAT	5
BP	GO:0021700	developmental maturation	11/210	296/18800	0.000516803	0.00208044	0.000973535	PGR/PPARG/KDR/AKR1B1/BCL2/RB1/VEGFA/MMP2/HIF1A/GJA1/RUNX2	11
BP	GO:00332	regulatio	4/210	34/18	0.0005	0.002165	0.0010134	MAOB/DRD1/SL	4

	38	n of cellular amine metabolic process		800	3953	77	64	C6A3/ABAT	
BP	GO:0051973	positive regulation of telomerase activity	4/210	34/18800	0.00053953	0.00216577	0.001013464	HSP90AB1/MAPK3/MAPK1/MYC	4
BP	GO:0070670	response to interleukin-4	4/210	34/18800	0.00053953	0.00216577	0.001013464	HSP90AB1/FASN/CDK4/HSPA5	4
BP	GO:0010951	negative regulation of endopeptidase activity	10/210	251/18800	0.000540717	0.002168486	0.001014735	PTGS2/DPEP1/AKT1/SLPI/VEGFA/MMP9/POR/RAF1/BIRC5/SERPINE1	10
BP	GO:0090398	cellular senescence	6/210	91/18800	0.000547688	0.00219437	0.001026848	MAPK14/CDK2/MAPK10/MAPK8/CDKN1A/TP53	6
BP	GO:0043407	negative regulation of MAP kinase activity	5/210	60/18800	0.000549868	0.002196885	0.001028024	PPARG/GSTP1/HMGCR/CAV1/IL1B	5
BP	GO:0046324	regulation of glucose import	5/210	60/18800	0.000549868	0.002196885	0.001028024	MAPK14/AKT1/MYC/NFE2L2/HK2	5
BP	GO:0090311	regulation of protein deacetylation	5/210	60/18800	0.000549868	0.002196885	0.001028024	MAPK8/SREBF1/VEGFA/TP53/IFNG	5
BP	GO:0048308	organelle inheritance	3/210	15/18800	0.000566157	0.0022556	0.0010555	CDK1/MAPK3/MAPK1	3

BP	GO:0048313	Golgi inheritance	3/210	15/18800	0.000566157	0.0022556	0.0010555	CDK1/MAPK3/MAPK1	3
BP	GO:2001028	positive regulation of endothelial cell chemotaxis	3/210	15/18800	0.000566157	0.0022556	0.0010555	KDR/VEGFA/HSPB1	3
BP	GO:0002065	columnar/cuboidal epithelial cell differentiation	6/210	92/18800	0.000580525	0.002310674	0.001081272	GSK3B/AKT1/FASN/BAD/HIF1A/SERPINE1	6
BP	GO:0016051	carbohydrate biosynthetic process	9/210	209/18800	0.000586902	0.002333871	0.001092127	GSK3B/AKR1B1/AKT1/HAS2/PPARA/GOT1/EGF/IGF2/DGAT2	9
BP	GO:0050714	positive regulation of protein secretion	7/210	128/18800	0.000592965	0.002353847	0.001101474	ACHE/PPARG/PPARD/BAD/ABAT/HIF1A/IL1A	7
BP	GO:0050994	regulation of lipid catabolic process	5/210	61/18800	0.000593588	0.002353847	0.001101474	AKT1/LDLR/PPARA/CDK4/IL1B	5
BP	GO:1904589	regulation of protein import	5/210	61/18800	0.000593588	0.002353847	0.001101474	PTGS2/HSP90AB1/MAPK14/CDK1/IFNG	5
BP	GO:0033280	response to vitamin D	4/210	35/18800	0.00060386	0.002385667	0.001116365	PTGS2/RXRA/CXCL10/SPP1	4
BP	GO:0090050	positive regulatio	4/210	35/18800	0.00060386	0.002385667	0.001116365	PTGS2/KDR/HMOX1/VEGFA	4

		n of cell migration involved in sprouting angiogenesis							
BP	GO:0098664	G protein-coupled serotonin receptor signaling pathway	4/210	35/18800	0.00060386	0.002385667	0.001116365	CHRM3/CHRM1/CHRM2/CHRM5	4
BP	GO:0098810	neurotransmitter reuptake	4/210	35/18800	0.00060386	0.002385667	0.001116365	SLC6A2/SLC6A4/DRD1/SLC6A3	4
BP	GO:0048863	stem cell differentiation	9/210	211/18800	0.000628396	0.002480296	0.001160646	ESR1/GSK3B/MAPK3/MAPK1/STAT3/TP53/HIF1A/NFE2L2/RUNX2	9
BP	GO:0045927	positive regulation of growth	10/210	256/18800	0.000629999	0.002484313	0.001162525	MAPK14/SLC6A3/PPARD/AKT1/BCL2/CDK1/EGFR/VEGFA/ERBB2/IGF2	10
BP	GO:0048639	positive regulation of developmental growth	8/210	169/18800	0.000637291	0.002510738	0.001174891	MAPK14/SLC6A3/PPARD/AKT1/BCL2/CDK1/VEGFA/IGF2	8
BP	GO:0048565	digestive tract development	7/210	130/18800	0.000650388	0.002559097	0.00119752	BCL2/CYP1A1/RB1/EGFR/HIF1A/CXCL8/COL3A1	7
BP	GO:0015918	sterol transport	6/210	94/18800	0.000650771	0.002559097	0.00119752	LDLR/MTTP/APOB/SOAT2/CES1/CAV1	6
BP	GO:00106	positive	4/210	36/18	0.0006	0.002638	0.0012346	ADRA1A/PPP3CA	4

	13	regulation of cardiac muscle hypertrophy		800	73448	5	76	/PRKCA/PARP1	
BP	GO:0045777	positive regulation of blood pressure	4/210	36/18800	0.000673448	0.0026385	0.001234676	ADRA1A/ADRA1B/ADRA1D/ADRB1	4
BP	GO:0071402	cellular response to lipoprotein particle stimulus	4/210	36/18800	0.000673448	0.0026385	0.001234676	PPARG/AKT1/LDLR/CES1	4
BP	GO:1905332	positive regulation of morphogenesis of an epithelium	4/210	36/18800	0.000673448	0.0026385	0.001234676	AR/VEGFA/EGF/GJA1	4
BP	GO:0042982	amyloid precursor protein metabolic process	6/210	95/18800	0.000688275	0.002673094	0.001250865	ACHE/RELA/CASP3/SOAT1/BACE2/IFNG	6
BP	GO:0060402	calcium ion transport into cytosol	8/210	171/18800	0.000688439	0.002673094	0.001250865	ADRA1A/DRD1/BCL2/BAX/GSTM2/CAV1/CXCL11/CXCL10	8
BP	GO:0032615	interleukin-12 production	5/210	63/18800	0.000688792	0.002673094	0.001250865	MAPK14/RELA/IFNG/CD40LG/IRF1	5
BP	GO:0032655	regulation of interleuk	5/210	63/18800	0.000688792	0.002673094	0.001250865	MAPK14/RELA/IFNG/CD40LG/IRF1	5

		in-12 production							
BP	GO:0051480	regulation of cytosolic calcium ion concentration	12/210	356/18800	0.000690967	0.002673094	0.001250865	ADRA1A/ADRA1B/ADRA1D/ESR1/DRD1/BCL2/BAX/GSTM2/CAV1/PTGER3/CXCL11/CXCL10	12
BP	GO:0006857	oligopeptide transport	3/210	16/18800	0.00069109	0.002673094	0.001250865	ABCC1/CA2/GJA1	3
BP	GO:0009812	flavonoid metabolic process	3/210	16/18800	0.00069109	0.002673094	0.001250865	CYP1A1/UGT1A1/POR	3
BP	GO:0010224	response to UV-B	3/210	16/18800	0.00069109	0.002673094	0.001250865	RELA/BCL2/CDKN1A	3
BP	GO:0034374	low-density lipoprotein particle remodeling	3/210	16/18800	0.00069109	0.002673094	0.001250865	MTTP/APOB/MPO	3
BP	GO:0042159	lipoprotein catabolic process	3/210	16/18800	0.00069109	0.002673094	0.001250865	LDLR/APOB/CTSD	3
BP	GO:0045838	positive regulation of membrane potential	3/210	16/18800	0.00069109	0.002673094	0.001250865	AKT1/BAD/MYC	3
BP	GO:0045986	negative regulation of smooth muscle	3/210	16/18800	0.00069109	0.002673094	0.001250865	PTGS2/ADRB2/SOD1	3

		contraction							
BP	GO:0070242	thymocyte apoptotic process	3/210	16/18800	0.00069109	0.002673094	0.001250865	BAX/TP53/HIF1A	3
BP	GO:0071391	cellular response to estrogen stimulus	3/210	16/18800	0.00069109	0.002673094	0.001250865	AR/ESR1/MYC	3
BP	GO:1905475	regulation of protein localization to membrane	8/210	172/18800	0.000715225	0.002763933	0.001293372	AR/AKT1/TNFAIP6/MAPK8/EGFR/BCL2L1/ERBB2/IFNG	8
BP	GO:0003300	cardiac muscle hypertrophy	6/210	96/18800	0.000727431	0.002808546	0.001314249	ADRA1A/PPARG/PPP3CA/PPARA/PRKCA/PARP1	6
BP	GO:0001885	endothelial cell development	5/210	64/18800	0.000740465	0.002853678	0.001335369	IKBKB/ICAM1/FASN/VEGFA/IL1B	5
BP	GO:0030888	regulation of B cell proliferation	5/210	64/18800	0.000740465	0.002853678	0.001335369	IL4/BCL2/CASP3/AHR/CDKN1A	5
BP	GO:0003158	endothelium development	7/210	133/18800	0.000744704	0.002864816	0.00134058	KDR/IKBKB/ICAM1/FASN/VEGFA/IL1B/CXCL10	7
BP	GO:1903844	regulation of cellular response to transforming growth	7/210	133/18800	0.000744704	0.002864816	0.00134058	HSP90AB1/PPARG/IL4/PPARA/TP53/HSPA5/CAV1	7

		factor beta stimulus							
BP	GO:0008207	C21-steroid hormone metabolic process	4/210	37/18800	0.000748529	0.002866549	0.001341391	AKR1B1/HSD3B2/HSD3B1/AKR1C3	4
BP	GO:0014037	Schwann cell differentiation	4/210	37/18800	0.000748529	0.002866549	0.001341391	AKT1/CDK1/SOD1/ERBB3	4
BP	GO:0014742	positive regulation of muscle hypertrophy	4/210	37/18800	0.000748529	0.002866549	0.001341391	ADRA1A/PPP3CA/PRKCA/PARP1	4
BP	GO:0042596	fear response	4/210	37/18800	0.000748529	0.002866549	0.001341391	DPP4/DRD1/ADRB1/BCL2	4
BP	GO:0045762	positive regulation of adenylate cyclase activity	4/210	37/18800	0.000748529	0.002866549	0.001341391	ADRB2/DRD1/ADRB1/RAF1	4
BP	GO:0010466	negative regulation of peptidase activity	10/210	262/18800	0.000752891	0.002880654	0.001347992	PTGS2/DPEP1/AKT1/SLPI/VEGFA/MMP9/POR/RAF1/BIRC5/SERPINE1	10
BP	GO:0010810	regulation of cell-substrate adhesion	9/210	217/18800	0.000767508	0.002923763	0.001368164	KDR/GSK3B/BCL2/HAS2/VEGFA/PLAU/SERPINE1/COL1A1/RASA1	9
BP	GO:0032642	regulation of chemokine producti	6/210	97/18800	0.000768288	0.002923763	0.001368164	HMOX1/GSTP1/IL6R/HIF1A/IL1B/IFNG	6

		on							
BP	GO:0032755	positive regulation of interleukin-6 production	6/210	97/18800	0.000768288	0.002923763	0.001368164	NOS2/STAT3/IL6R/IL1B/IFNG/IL1A	6
BP	GO:0045807	positive regulation of endocytosis	6/210	97/18800	0.000768288	0.002923763	0.001368164	IL4/SELE/PPP3CA/VEGFA/EGF/SERPINE1	6
BP	GO:1990868	response to chemokine	6/210	97/18800	0.000768288	0.002923763	0.001368164	HIF1A/CCL2/CXCL8/CXCL11/CXCL2/CXCL10	6
BP	GO:1990869	cellular response to chemokine	6/210	97/18800	0.000768288	0.002923763	0.001368164	HIF1A/CCL2/CXCL8/CXCL11/CXCL2/CXCL10	6
BP	GO:0050767	regulation of neurogenesis	12/210	361/18800	0.000780282	0.002966748	0.001388279	OPRM1/PPP3CA/LDLR/VEGFA/TP53/HIF1A/MYC/IL1B/IFNG/SPP1/E2F1/MAP2	12
BP	GO:0032602	chemokine production	6/210	98/18800	0.000810896	0.003080388	0.001441456	HMOX1/GSTP1/IL6R/HIF1A/IL1B/IFNG	6
BP	GO:0055074	calcium ion homeostasis	14/210	468/18800	0.000818045	0.003104768	0.001452865	ADRA1A/ADRA1B/ADRA1D/ESR1/KDR/DRD1/BCL2/BAX/GSTM2/CAV1/PTGER3/PRKCB/CXCL11/CXCL10	14
BP	GO:0051651	maintenance of location in cell	9/210	219/18800	0.000819101	0.003106	0.001453442	DRD1/AKT1/BAX/GSTM2/HSPA5/CAV1/CXCL11/CXCL10/HK2	9
BP	GO:0031330	negative regulatio	10/210	265/18800	0.000821373	0.003111585	0.001456055	ADRA1A/HSP90AB1/MAPK14/AKT	10

		n of cellular catabolic process						1/BCL2/HMOX1/STAT3/IL10RA/TP53/E2F1	
BP	GO:0048246	macrophage chemotaxis	4/210	38/18800	0.000829338	0.003111585	0.001456055	MAPK3/MAPK1/CYP19A1/CCL2	4
BP	GO:0060416	response to growth hormone	4/210	38/18800	0.000829338	0.003111585	0.001456055	F7/AKT1/STAT3/MYC	4
BP	GO:1904037	positive regulation of epithelial cell apoptotic process	4/210	38/18800	0.000829338	0.003111585	0.001456055	AKR1C3/BAD/CCL2/CD40LG	4
BP	GO:0031056	regulation of histone modification	8/210	176/18800	0.000830899	0.003111585	0.001456055	CHEK1/MAPK8/MAPK3/SREBF1/VEGFA/TP53/IL1B/IGF2	8
BP	GO:0002544	chronic inflammatory response	3/210	17/18800	0.000832296	0.003111585	0.001456055	IL4/VCAM1/CYP19A1	3
BP	GO:0006978	DNA damage response, signal transduction by p53 class mediator resulting in transcription of p21	3/210	17/18800	0.000832296	0.003111585	0.001456055	CDKN1A/TP53/CHEK2	3

		class mediator							
BP	GO:0016264	gap junction assembly	3/210	17/18800	0.000832296	0.003111585	0.001456055	CAV1/GJA1/IL1B	3
BP	GO:0030949	positive regulation of vascular endothelial growth factor receptor signaling pathway	3/210	17/18800	0.000832296	0.003111585	0.001456055	HIF1A/IL1B/PRKCB	3
BP	GO:0032310	prostaglandin secretion	3/210	17/18800	0.000832296	0.003111585	0.001456055	NOS2/IL1B/IL1A	3
BP	GO:0033189	response to vitamin A	3/210	17/18800	0.000832296	0.003111585	0.001456055	PPARD/CYP1A1/CAT	3
BP	GO:0048569	post-embryonic animal organ development	3/210	17/18800	0.000832296	0.003111585	0.001456055	KDR/BAX/VEGFA	3
BP	GO:0060252	positive regulation of glial cell proliferation	3/210	17/18800	0.000832296	0.003111585	0.001456055	MYC/IL1B/E2F1	3
BP	GO:0090494	dopamine uptake	3/210	17/18800	0.000832296	0.003111585	0.001456055	SLC6A2/DRD1/SLC6A3	3
BP	GO:1901550	regulation of endothelial cell	3/210	17/18800	0.000832296	0.003111585	0.001456055	IKBKB/VEGFA/IL1B	3

		development							
BP	GO:1903140	regulation of establishment of endothelial barrier	3/210	17/18800	0.000832296	0.003111585	0.001456055	IKBKB/VEGFA/IL1B	3
BP	GO:0031589	cell-substrate adhesion	12/210	364/18800	0.000838366	0.003131523	0.001465385	KDR/GSK3B/PPARD/BCL2/VCAM1/HAS2/VEGFA/PLAU/SERPINE1/COL1A1/COL3A1/RASA1	12
BP	GO:0006879	cellular iron ion homeostasis	5/210	66/18800	0.000852432	0.003178473	0.001487355	HMOX1/SOD1/HIF1A/MYC/IFNG	5
BP	GO:0033013	tetrapyrrole metabolic process	5/210	66/18800	0.000852432	0.003178473	0.001487355	HMOX1/CYP1A2/CYP1A1/UGT1A1/ABCC1	5
BP	GO:0014897	striated muscle hypertrophy	6/210	99/18800	0.000855304	0.00318359	0.00148975	ADRA1A/PPARG/PPP3CA/PPARA/PRKCA/PARP1	6
BP	GO:0015908	fatty acid transport	6/210	99/18800	0.000855304	0.00318359	0.00148975	PPARG/PPARD/AKT1/PPARA/IL1B/IL1A	6
BP	GO:0007586	digestion	7/210	137/18800	0.000886949	0.003298487	0.001543515	CHRM3/CHRM1/PRSS1/CHRM5/LDLR/SOAT2/PTGER3	7
BP	GO:0014912	negative regulation of smooth muscle cell migration	4/210	39/18800	0.000916109	0.003395041	0.001588697	PPARD/SERPINE1/NFE2L2/IGFBP3	4
BP	GO:00458	positive	4/210	39/18	0.0009	0.003395	0.0015886	ADRA1A/ADRA1	4

	23	regulation of heart contraction		800	16109	041	97	B/ADRA1D/ADRB1	
BP	GO:0060306	regulation of membrane repolarization	4/210	39/18800	0.000916109	0.003395041	0.001588697	SCN5A/KCNH2/CAV1/GJA1	4
BP	GO:0097242	amyloid-beta clearance	4/210	39/18800	0.000916109	0.003395041	0.001588697	IL4/LDLR/HMGCR/IFNG	4
BP	GO:0031644	regulation of nervous system process	7/210	138/18800	0.000925654	0.003424438	0.001602453	ADRB2/OPRM1/OPRD1/ABAT/NOS3/IL1A/ACP3	7
BP	GO:0060048	cardiac muscle contraction	7/210	138/18800	0.000925654	0.003424438	0.001602453	SCN5A/ADRA1A/ADRA1B/GSTM2/KCNH2/CAV1/GJA1	7
BP	GO:0015849	organic acid transport	11/210	318/18800	0.000931859	0.003444392	0.001611791	PPARG/PPARD/AKT1/PPARA/ABCC1/ABAT/MYC/GJA1/IL1B/IL1A/ABCG2	11
BP	GO:0014896	muscle hypertrophy	6/210	101/18800	0.000949729	0.003504342	0.001639844	ADRA1A/PPARG/PPP3CA/PPARA/PRKCA/PARP1	6
BP	GO:0099565	chemical synaptic transmission, postsynaptic	6/210	101/18800	0.000949729	0.003504342	0.001639844	ADRB2/OPRM1/GSK3B/AKT1/PPP3CA/ABAT	6
BP	GO:0034765	regulation of ion transmembrane transport	14/210	476/18800	0.000962598	0.003548744	0.001660622	CHRM3/SCN5A/ADRB2/OPRM1/DRD1/BAX/GSTM2/KCNH2/MMP9/CAV1/CCL2/IFNG/	14

								CXCL11/CXCL10	
BP	GO:0006986	response to unfolded protein	7/210	139/18800	0.000965679	0.003553933	0.00166305	HSP90AB1/BAX/CCND1/HSPA5/HSPB1/NFE2L2/HSF1	7
BP	GO:0051783	regulation of nuclear division	7/210	139/18800	0.000965679	0.003553933	0.00166305	CHEK1/RB1/EGF/IL1B/CCNB1/IL1A/IGF2	7
BP	GO:0009206	purine ribonucleoside triphosphate biosynthetic process	5/210	68/18800	0.000976551	0.003587726	0.001678864	IL4/ATP5F1B/PPARA/MYC/PARP1	5
BP	GO:0050709	negative regulation of protein secretion	5/210	68/18800	0.000976551	0.003587726	0.001678864	OPRM1/PPP3CA/HMGCR/SREBF1/IL1B	5
BP	GO:0006977	DNA damage response, signal transduction by p53 class mediator resulting in cell cycle arrest	3/210	18/18800	0.000990566	0.003616042	0.001692114	CDKN1A/TP53/CHEK2	3
BP	GO:0030540	female genitalia development	3/210	18/18800	0.000990566	0.003616042	0.001692114	ESR1/BAX/CYP19A1	3
BP	GO:0030730	sequestering of triglyceride	3/210	18/18800	0.000990566	0.003616042	0.001692114	PPARG/PPARA/IL1B	3

BP	GO:0032354	response to follicle-stimulating hormone	3/210	18/18800	0.000990566	0.003616042	0.001692114	CYP1B1/POR/PLAT	3
BP	GO:0042772	DNA damage response, signal transduction resulting in transcription	3/210	18/18800	0.000990566	0.003616042	0.001692114	CDKN1A/TP53/CHEK2	3
BP	GO:0090493	catecholamine uptake	3/210	18/18800	0.000990566	0.003616042	0.001692114	SLC6A2/DRD1/SLC6A3	3
BP	GO:0097284	hepatocyte apoptotic process	3/210	18/18800	0.000990566	0.003616042	0.001692114	PPARA/RB1/BCL2L1	3
BP	GO:0060348	bone development	9/210	225/18800	0.00099107	0.003616042	0.001692114	KDR/MAPK14/HAS2/TP53/POR/GJA1/COL1A1/COL3A1/RUNX2	9
BP	GO:0032677	regulation of interleukin-8 production	6/210	102/18800	0.000999848	0.003644941	0.001705637	NOS2/RELA/STAT3/F3/IL1B/SERPINE1	6
BP	GO:0042157	lipoprotein metabolic process	7/210	140/18800	0.001007054	0.003666008	0.001715495	OLR1/LDLR/MTTP/APOB/PPARA/CTSD/DGAT2	7
BP	GO:0010863	positive regulation of phospholipase C	4/210	40/18800	0.00100908	0.003666008	0.001715495	ADRA1A/ESR1/SELE/EGFR	4

		activity							
BP	GO:0045740	positive regulation of DNA replication	4/210	40/18800	0.00100908	0.003666008	0.001715495	CDK2/CDK1/EGFR/EGF	4
BP	GO:1903524	positive regulation of blood circulation	4/210	40/18800	0.00100908	0.003666008	0.001715495	ADRA1A/ADRA1B/ADRA1D/ADRB1	4
BP	GO:0009145	purine nucleoside triphosphate biosynthetic process	5/210	69/18800	0.001043415	0.003787508	0.001772351	IL4/ATP5F1B/PPARA/MYC/PARP1	5
BP	GO:0038061	NIK/NF-kappaB signaling	7/210	141/18800	0.001049812	0.003805574	0.001780805	NR3C2/RELA/AKT1/EGFR/NFKBIA/IL1B/CHUK	7
BP	GO:0032637	interleukin-8 production	6/210	103/18800	0.001051977	0.003805574	0.001780805	NOS2/RELA/STAT3/F3/IL1B/SERPINE1	6
BP	GO:1901796	regulation of signal transduction by p53 class mediator	6/210	103/18800	0.001051977	0.003805574	0.001780805	CHEK1/AKT1/BCL2/TP53/MYC/CHEK2	6
BP	GO:1905477	positive regulation of protein localization to membra	6/210	103/18800	0.001051977	0.003805574	0.001780805	AKT1/TNFAIP6/MAPK8/EGFR/ERBB2/IFNG	6

		ne							
BP	GO:0030183	B cell differentiation	7/210	142/18800	0.001093984	0.00395417	0.00185034	IL4/BCL2/BAX/VCAM1/BAD/TP53/CD40LG	7
BP	GO:0022600	digestive system process	6/210	104/18800	0.001106167	0.003994804	0.001869354	CHRM3/CHRM1/CHRM5/LDLR/SOAT2/PTGER3	6
BP	GO:0071548	response to dexamethasone	4/210	41/18800	0.001108485	0.003996381	0.001870092	CYP1B1/CASP9/POR/PLAT	4
BP	GO:0120178	steroid hormone biosynthetic process	4/210	41/18800	0.001108485	0.003996381	0.001870092	AKR1B1/HSD3B2/HSD3B1/CYP19A1	4
BP	GO:0032722	positive regulation of chemokine production	5/210	70/18800	0.001113618	0.00400808	0.001875566	HMOX1/IL6R/HIF1A/IL1B/IFNG	5
BP	GO:0032890	regulation of organic acid transport	5/210	70/18800	0.001113618	0.00400808	0.001875566	AKT1/PPARA/ABAT/IL1B/IL1A	5
BP	GO:0061138	morphogenesis of a branching epithelium	8/210	185/18800	0.00114645	0.004122755	0.001929228	PGR/AR/ESR1/KDR/BCL2/VEGFA/EGF/MYC	8
BP	GO:0045833	negative regulation of lipid metabolic process	6/210	105/18800	0.001162472	0.004170704	0.001951666	AKT1/AKR1C3/SOD1/UGT1A1/IL1B/DGAT2	6
BP	GO:0010544	negative regulatio	3/210	19/18800	0.001166658	0.004170704	0.001951666	ABAT/NOS3/THBD	3

		n of platelet activation							
BP	GO:0015732	prostaglandin transport	3/210	19/18800	0.001166658	0.004170704	0.001951666	NOS2/IL1B/IL1A	3
BP	GO:0019373	epoxygenase P450 pathway	3/210	19/18800	0.001166658	0.004170704	0.001951666	CYP1A2/CYP1A1/CYP1B1	3
BP	GO:0051546	keratinocyte migration	3/210	19/18800	0.001166658	0.004170704	0.001951666	PPARD/HAS2/MMP9	3
BP	GO:0051900	regulation of mitochondrial depolarization	3/210	19/18800	0.001166658	0.004170704	0.001951666	KDR/BCL2/PARP1	3
BP	GO:0097202	activation of cysteine-type endopeptidase activity	3/210	19/18800	0.001166658	0.004170704	0.001951666	BAD/CASP9/CASP8	3
BP	GO:1903038	negative regulation of leukocyte cell-cell adhesion	7/210	144/18800	0.001186702	0.004233662	0.001981127	IL4/AKT1/CASP3/PPARA/ERBB2/IL2RA/IRF1	7
BP	GO:0050805	negative regulation of synaptic transmission	5/210	71/18800	0.00118726	0.004233662	0.001981127	PTGS2/ACHE/SLC6A4/DRD1/IL1B	5
BP	GO:1904427	positive regulation of	5/210	71/18800	0.00118726	0.004233662	0.001981127	DRD1/BAX/GSTM2/CXCL11/CXCL10	5

		calcium ion transmembrane transport							
BP	GO:0010907	positive regulation of glucose metabolic process	4/210	42/18800	0.00121456	0.004309305	0.002016524	AKT1/PPARA/IGF2/DGAT2	4
BP	GO:0014911	positive regulation of smooth muscle cell migration	4/210	42/18800	0.00121456	0.004309305	0.002016524	BCL2/CYP1B1/HAS2/MYC	4
BP	GO:0030574	collagen catabolic process	4/210	42/18800	0.00121456	0.004309305	0.002016524	MMP1/MMP3/MMP2/MMP9	4
BP	GO:0031670	cellular response to nutrient	4/210	42/18800	0.00121456	0.004309305	0.002016524	RXRA/NCOA1/HMOX1/COL1A1	4
BP	GO:0042417	dopamine metabolic process	4/210	42/18800	0.00121456	0.004309305	0.002016524	MAOB/DRD1/SLC6A3/ABAT	4
BP	GO:1900274	regulation of phospholipase C activity	4/210	42/18800	0.00121456	0.004309305	0.002016524	ADRA1A/ESR1/SELE/EGFR	4
BP	GO:0051896	regulation of protein kinase B signaling	8/210	187/18800	0.001228109	0.004353739	0.002037316	HSP90AB1/F7/AKR1C3/PPARA/EGFR/EGF/F3/IGF2	8

BP	GO:0043010	camera-type eye development	11/210	330/18800	0.001255656	0.004447683	0.002081277	ACHE/KDR/SLC6A3/JUN/BAX/CYP1A1/CYP1B1/EGFR/VEGFA/HIF1A/GJA1	11
BP	GO:0051937	catecholamine transport	5/210	72/18800	0.001264444	0.004475078	0.002094097	SLC6A2/DRD1/CHRM5/SLC6A3/ABAT	5
BP	GO:0030004	cellular monovalent inorganic cation homeostasis	6/210	107/18800	0.001281649	0.004532194	0.002120824	ATP5F1B/BCL2/MAPK3/MAPK1/CA2/IL1A	6
BP	GO:0009749	response to glucose	8/210	189/18800	0.00131434	0.004643928	0.002173109	PPARD/CASP3/ICAM1/PPP3CA/BAD/SREBF1/RAF1/HIF1A	8
BP	GO:0001504	neurotransmitter uptake	4/210	43/18800	0.001327541	0.004663412	0.002182227	SLC6A2/SLC6A4/DRD1/SLC6A3	4
BP	GO:0006775	fat-soluble vitamin metabolic process	4/210	43/18800	0.001327541	0.004663412	0.002182227	PPARD/CYP3A4/CYP1A1/IFNG	4
BP	GO:0014002	astrocyte development	4/210	43/18800	0.001327541	0.004663412	0.002182227	DRD1/LDLR/IL1B/IFNG	4
BP	GO:0031063	regulation of histone deacetylation	4/210	43/18800	0.001327541	0.004663412	0.002182227	MAPK8/SREBF1/VEGFA/TP53	4
BP	GO:0042554	superoxide anion generation	4/210	43/18800	0.001327541	0.004663412	0.002182227	NCF1/GSTP1/SOD1/DUOX2	4
BP	GO:0042771	intrinsic apoptotic	4/210	43/18800	0.001327541	0.004663412	0.002182227	BCL2/CDKN1A/TP53/CHEK2	4

		signaling pathway in response to DNA damage by p53 class mediator							
BP	GO:0071604	transforming growth factor beta production	4/210	43/18800	0.001327541	0.004663412	0.002182227	PTGS2/HSP90AB1/HIF1A/COL3A1	4
BP	GO:0007160	cell-matrix adhesion	9/210	235/18800	0.001341987	0.004710263	0.002204151	KDR/GSK3B/BCL2/VCAM1/VEGFA/PLAU/SERPINE1/COL3A1/RASA1	9
BP	GO:0005976	polysaccharide metabolic process	6/210	108/18800	0.001344631	0.004714009	0.002205904	GSK3B/PYGM/AKT1/HAS2/EGF/IGF2	6
BP	GO:2000573	positive regulation of DNA biosynthetic process	5/210	73/18800	0.001345274	0.004714009	0.002205904	HSP90AB1/CYP1B1/MAPK3/MAPK1/MYC	5
BP	GO:0043433	negative regulation of DNA-binding transcription factor activity	8/210	190/18800	0.001359226	0.00473112	0.002213911	ESR1/HMOX1/CYP1B1/CAT/RB1/NFKBIA/CHUK/E2F1	8
BP	GO:0060401	cytosolic	8/210	190/18800	0.001359226	0.00473112	0.002213911	ADRA1A/DRD1/BCL2/BAX/GSTM2	8

		calcium ion transport						/CAV1/CXCL11/CXCL10	
BP	GO:0006007	glucose catabolic process	3/210	20/18800	0.001361297	0.00473112	0.002213911	BAD/TP53/HK2	3
BP	GO:0032495	response to muramyl dipeptide	3/210	20/18800	0.001361297	0.00473112	0.002213911	MAPK14/RELA/NFKBIA	3
BP	GO:0042359	vitamin D metabolic process	3/210	20/18800	0.001361297	0.00473112	0.002213911	CYP3A4/CYP1A1/IFNG	3
BP	GO:0043651	linoleic acid metabolic process	3/210	20/18800	0.001361297	0.00473112	0.002213911	ALOX5/GSTP1/GSTM2	3
BP	GO:0045821	positive regulation of glycolytic process	3/210	20/18800	0.001361297	0.00473112	0.002213911	HIF1A/MYC/IFNG	3
BP	GO:0070293	renal absorption	3/210	20/18800	0.001361297	0.00473112	0.002213911	HAS2/AKR1C3/CLDN4	3
BP	GO:1900221	regulation of amyloid-beta clearance	3/210	20/18800	0.001361297	0.00473112	0.002213911	IL4/HMGCR/IFNG	3
BP	GO:1902004	positive regulation of amyloid-beta formatio	3/210	20/18800	0.001361297	0.00473112	0.002213911	RELA/CASP3/IFNG	3

		n							
BP	GO:0007517	muscle organ development	11/210	334/18800	0.00138223	0.004799944	0.002246117	MAPK14/BCL2/PPP3CA/RB1/FOS/CAV1/GJA1/COL3A1/CXCL10/IGF2/ERBB3	11
BP	GO:0051960	regulation of nervous system development	13/210	440/18800	0.001396711	0.004846269	0.002267794	OPRM1/AKT1/PPP3CA/LDLR/VEGFA/TP53/HIF1A/MYC/IL1B/IFNG/SPP1/E2F1/MAP2	13
BP	GO:0000271	polysaccharide biosynthetic process	5/210	74/18800	0.001429854	0.004953173	0.002317819	GSK3B/AKT1/HAS2/EGF/IGF2	5
BP	GO:0009201	ribonucleoside triphosphate biosynthetic process	5/210	74/18800	0.001429854	0.004953173	0.002317819	IL4/ATP5F1B/PPARA/MYC/PARP1	5
BP	GO:0014009	glial cell proliferation	4/210	44/18800	0.001447661	0.00501077	0.002344772	CHRM1/MYC/IL1B/E2F1	4
BP	GO:0006164	purine nucleotide biosynthetic process	8/210	192/18800	0.001452655	0.005023964	0.002350946	NOS2/IL4/ATP5F1B/PPARA/ACACA/MYC/NOS3/PARP1	8
BP	GO:0030902	hindbrain development	7/210	150/18800	0.001502253	0.005191269	0.002429236	SCN5A/SLC6A4/BCL2/ABAT/EGF/TP53/HSPA5	7
BP	GO:0070227	lymphocyte apoptotic process	5/210	75/18800	0.001518288	0.005242414	0.002453169	AKT1/BAX/TP53/HIF1A/IL2RA	5
BP	GO:0046632	alpha-beta T cell	6/210	111/18800	0.001547822	0.005340049	0.002498857	IL4/BCL2/STAT3/IL6R/IFNG/IRF1	6

		differentiation							
BP	GO:0031334	positive regulation of protein-containing complex assembly	8/210	194/18800	0.001551115	0.005342731	0.002500112	ESR1/GSK3B/BAX/MMP1/MMP3/VEGFA/TP53/IFNG	8
BP	GO:0071897	DNA biosynthetic process	8/210	194/18800	0.001551115	0.005342731	0.002500112	HSP90AB1/CHEK1/CYP1B1/MAPK3/MAPK1/CDKN1A/TP53/MYC	8
BP	GO:0006790	sulfur compound metabolic process	11/210	339/18800	0.001555007	0.005351793	0.002504352	DPEP1/GSTP1/GSTM1/GSTM2/SOD1/GSR/ACACA/SULT1E1/NFE2L2/ACP3/DGAT2	11
BP	GO:0045580	regulation of T cell differentiation	7/210	151/18800	0.001560659	0.005352847	0.002504846	IL4/BAD/SOD1/ERBB2/IL2RA/IFNG/IRF1	7
BP	GO:0031295	T cell costimulation	4/210	45/18800	0.001575154	0.005352847	0.002504846	DPP4/AKT1/CAV1/CD40LG	4
BP	GO:0045933	positive regulation of muscle contraction	4/210	45/18800	0.001575154	0.005352847	0.002504846	PTGS2/CHRM3/ADRA1A/ABAT	4
BP	GO:0046427	positive regulation of receptor signaling pathway via JAK-ST	4/210	45/18800	0.001575154	0.005352847	0.002504846	IL4/CYP1B1/IL10RA/IL6R	4

		AT							
BP	GO:1901861	regulation of muscle tissue development	4/210	45/18800	0.001575154	0.005352847	0.002504846	BCL2/GJA1/IGF2/ERBB3	4
BP	GO:1902622	regulation of neutrophil migration	4/210	45/18800	0.001575154	0.005352847	0.002504846	DPP4/TNFAIP6/CXCL8/IL1A	4
BP	GO:0003085	negative regulation of systemic arterial blood pressure	3/210	21/18800	0.001575179	0.005352847	0.002504846	ADRA1A/ADRB2/ADRB1	3
BP	GO:0006067	ethanol metabolic process	3/210	21/18800	0.001575179	0.005352847	0.002504846	ADH1B/ADH1C/SULT1E1	3
BP	GO:0007252	I-kappaB phosphorylation	3/210	21/18800	0.001575179	0.005352847	0.002504846	IKBKB/AKT1/CHUK	3
BP	GO:0051882	mitochondrial depolarization	3/210	21/18800	0.001575179	0.005352847	0.002504846	KDR/BCL2/PARP1	3
BP	GO:0071243	cellular response to arsenic-containing substance	3/210	21/18800	0.001575179	0.005352847	0.002504846	HMOX1/MYC/HSF1	3
BP	GO:0071404	cellular response to low-den	3/210	21/18800	0.001575179	0.005352847	0.002504846	PPARG/LDLR/CES1	3

		sity lipoprotein particle stimulus							
BP	GO:0072111	cell proliferation involved in kidney development	3/210	21/18800	0.001575179	0.005352847	0.002504846	STAT1/IL6R/MYC	3
BP	GO:1900543	negative regulation of purine nucleotide metabolic process	3/210	21/18800	0.001575179	0.005352847	0.002504846	PPARA/STAT3/PARP1	3
BP	GO:1901739	regulation of myoblast fusion	3/210	21/18800	0.001575179	0.005352847	0.002504846	MAPK14/IL4/CXCL10	3
BP	GO:0031349	positive regulation of defense response	10/210	289/18800	0.001575479	0.005352847	0.002504846	PTGS2/IKBKB/MAPK3/LDLR/ABCC1/NFKBIA/IL1B/PTGER3/SERPINE1/IFNG	10
BP	GO:0051216	cartilage development	8/210	195/18800	0.001602291	0.005439593	0.002545438	MAPK14/MAPK3/RB1/POR/HIF1A/COL1A1/COL3A1/RUNX2	8
BP	GO:0001937	negative regulation of endothelial cell proliferation	5/210	76/18800	0.001610682	0.00546371	0.002556724	PPARG/STAT1/ALOX5/CAV1/CCL2	5
BP	GO:0021987	cerebral cortex	6/210	112/18800	0.001620491	0.005492599	0.002570242	GSK3B/BAX/BAD/EGFR/HIF1A/CO	6

		development						L3A1	
BP	GO:0031623	receptor internalization	6/210	113/18800	0.001695726	0.00574302	0.002687426	ACHE/SELE/VEGFA/EGF/CAV1/CXCL8	6
BP	GO:0055117	regulation of cardiac muscle contraction	5/210	77/18800	0.00170714	0.005769219	0.002699685	SCN5A/ADRA1A/ADRA1B/GSTM2/CAV1	5
BP	GO:0031018	endocrine pancreas development	4/210	46/18800	0.001710254	0.005769219	0.002699685	GSK3B/AKT1/BAD/IL6R	4
BP	GO:0035794	positive regulation of mitochondrial membrane permeability	4/210	46/18800	0.001710254	0.005769219	0.002699685	GSK3B/BAX/MAPK8/TP53	4
BP	GO:0048806	genitalia development	4/210	46/18800	0.001710254	0.005769219	0.002699685	AR/ESR1/BAX/CYP19A1	4
BP	GO:1904707	positive regulation of vascular associated smooth muscle cell proliferation	4/210	46/18800	0.001710254	0.005769219	0.002699685	JUN/MMP2/MMP9/GJA1	4
BP	GO:0048762	mesenchymal cell differentiation	9/210	244/18800	0.001737696	0.005857137	0.002740826	GSK3B/BCL2/STAT1/HAS2/MAPK3/MAPK1/HIF1A/IL1B/COL1A1	9

BP	GO:0001678	cellular glucose homeostasis	7/210	154/18800	0.001746548	0.005882308	0.002752605	PPARD/ICAM1/PPP3CA/BAD/RAF1/HIF1A/HK2	7
BP	GO:0007422	peripheral nervous system development	5/210	78/18800	0.001807771	0.006044604	0.002828551	AKT1/CDK1/SOD1/ERBB2/ERBB3	5
BP	GO:1901616	organic hydroxy compound catabolic process	5/210	78/18800	0.001807771	0.006044604	0.002828551	MAOB/SLC6A3/CYP3A4/AKR1C3/SULT1E1	5
BP	GO:0002363	alpha-beta T cell lineage commitment	3/210	22/18800	0.001808969	0.006044604	0.002828551	BCL2/STAT3/IL6R	3
BP	GO:0010829	negative regulation of glucose transmembrane transport	3/210	22/18800	0.001808969	0.006044604	0.002828551	MYC/IL1B/PRKCB	3
BP	GO:0010866	regulation of triglyceride biosynthetic process	3/210	22/18800	0.001808969	0.006044604	0.002828551	LDLR/SREBF1/DGAT2	3
BP	GO:0031065	positive regulation of histone deacetylation	3/210	22/18800	0.001808969	0.006044604	0.002828551	SREBF1/VEGFA/TP53	3
BP	GO:0043369	CD4-positive or	3/210	22/18800	0.001808969	0.006044604	0.002828551	BCL2/STAT3/IL6R	3

		CD8-positive, alpha-beta T cell lineage commitment							
BP	GO:0044321	response to leptin	3/210	22/18800	0.001808969	0.006044604	0.002828551	CCNA2/STAT3/CCND1	3
BP	GO:0045980	negative regulation of nucleotide metabolic process	3/210	22/18800	0.001808969	0.006044604	0.002828551	PPARA/STAT3/PARP1	3
BP	GO:2000310	regulation of NMDA receptor activity	3/210	22/18800	0.001808969	0.006044604	0.002828551	OPRM1/CCL2/IFNG	3
BP	GO:0007519	skeletal muscle tissue development	7/210	155/18800	0.001812189	0.006050603	0.002831358	MAPK14/BCL2/PPP3CA/RB1/FOS/CAV1/IGF2	7
BP	GO:0001763	morphogenesis of a branching structure	8/210	199/18800	0.00182053	0.006073676	0.002842155	PGR/AR/ESR1/KDR/BCL2/VEGFA/EGF/MYC	8
BP	GO:0001774	microglial cell activation	4/210	47/18800	0.001853191	0.00614233	0.002874281	JUN/IL4/LDLR/IFNG	4
BP	GO:0034198	cellular response to amino acid starvation	4/210	47/18800	0.001853191	0.00614233	0.002874281	MAPK8/MAPK3/MAPK1/CDKN1A	4
BP	GO:00429	xenobiot	4/210	47/18	0.0018	0.006142	0.0028742	NR1I2/ABCC1/GJ	4

	08	ic transport		800	53191	33	81	A1/ABCG2	
BP	GO:0045646	regulation of erythrocyte differentiation	4/210	47/18800	0.001853191	0.00614233	0.002874281	MAPK14/STAT1/STAT3/HIF1A	4
BP	GO:0060711	labyrinthine layer development	4/210	47/18800	0.001853191	0.00614233	0.002874281	NCOA1/AKT1/MAPK1/CASP8	4
BP	GO:0101023	vascular endothelial cell proliferation	4/210	47/18800	0.001853191	0.00614233	0.002874281	PPARG/STAT3/CCL2/IGF2	4
BP	GO:1905562	regulation of vascular endothelial cell proliferation	4/210	47/18800	0.001853191	0.00614233	0.002874281	PPARG/STAT3/CCL2/IGF2	4
BP	GO:0021782	glial cell development	6/210	115/18800	0.001854125	0.00614233	0.002874281	DRD1/AKT1/LDLR/SOD1/IL1B/IFNG	6
BP	GO:0030518	intracellular steroid hormone receptor signaling pathway	6/210	115/18800	0.001854125	0.00614233	0.002874281	PGR/AR/NR3C2/ESR1/ESR2/PARP1	6
BP	GO:0007265	Ras protein signal transduction	11/210	347/18800	0.001867992	0.006183445	0.002893521	ADRA1A/CCNA2/CDK2/JUN/RB1/CDKN1A/TP53/RAF1/COL3A1/RASSF1/RASA1	11
BP	GO:0043588	skin development	10/210	296/18800	0.001878796	0.006214366	0.00290799	RELA/BCL2/CASP3/AKR1C3/FOSL2/EGFR/COL1A1/I	10

								L1A/COL3A1/CLDN4	
BP	GO:0030301	cholesterol transport	5/210	79/18800	0.001912679	0.0063166	0.00295583	LDLR/APOB/SOAT2/CES1/CAV1	5
BP	GO:0034637	cellular carbohydrate biosynthetic process	5/210	79/18800	0.001912679	0.0063166	0.00295583	GSK3B/AKT1/HAS2/GOT1/IGF2	5
BP	GO:0006874	cellular calcium ion homeostasis	13/210	456/18800	0.001914735	0.006318475	0.002956708	ADRA1A/ADRA1B/ADRA1D/ESR1/DRD1/BCL2/BAX/GSTM2/CAV1/PTGER3/PRKCB/CXCL11/CXCL10	13
BP	GO:0010720	positive regulation of cell development	10/210	297/18800	0.001925739	0.006349856	0.002971392	OPRM1/BCL2/HAS2/BAD/VEGFA/HIF1A/MYC/IL1B/IFNG/E2F1	10
BP	GO:0002286	T cell activation involved in immune response	6/210	116/18800	0.001937407	0.006383373	0.002987076	IL4/ICAM1/STAT3/IL6R/TP53/IFNG	6
BP	GO:0055067	monovalent inorganic cation homeostasis	7/210	157/18800	0.001949207	0.006417273	0.00300294	ATP5F1B/BCL2/MAPK3/MAPK1/KCNH2/CA2/IL1A	7
BP	GO:0072522	purine-containing compound biosynthetic process	8/210	202/18800	0.001999112	0.006576473	0.003077437	NOS2/IL4/ATP5F1B/PPARA/ACACA/MYC/NOS3/PARP1	8
BP	GO:00105	regulatio	4/210	48/18	0.0020	0.006577	0.0030781	ABAT/PRKCA/N	4

	43	n of platelet activation		800	04197	919	13	OS3/THBD	
BP	GO:0033628	regulation of cell adhesion mediated by integrin	4/210	48/18800	0.002004197	0.006577919	0.003078113	DPP4/CYP1B1/PLAU/SERPINE1	4
BP	GO:1902108	regulation of mitochondrial membrane permeability involved in apoptotic process	4/210	48/18800	0.002004197	0.006577919	0.003078113	GSK3B/BAX/MAPK8/TP53	4
BP	GO:0001570	vasculogenesis	5/210	80/18800	0.002021972	0.006610588	0.003093401	KDR/HAS2/VEGFA/CAV1/RASA1	5
BP	GO:0021766	hippocampus development	5/210	80/18800	0.002021972	0.006610588	0.003093401	GSK3B/DRD1/HSD3B2/HSD3B1/CASP3	5
BP	GO:0051279	regulation of release of sequestered calcium ion into cytosol	5/210	80/18800	0.002021972	0.006610588	0.003093401	DRD1/BAX/GSTM2/CXCL11/CXCL10	5
BP	GO:0015718	monocarboxylic acid transport	6/210	117/18800	0.00202349	0.006610588	0.003093401	NOS2/CES1/MYC/IL1B/IL1A/ABCG2	6
BP	GO:0032411	positive regulatio	6/210	117/18800	0.00202349	0.006610588	0.003093401	RXRA/ADRB2/GSTM2/CCL2/IFNG/	6

		n of transporter activity						PON1	
BP	GO:0045446	endothelial cell differentiation	6/210	117/18800	0.00202349	0.006610588	0.003093401	KDR/IKBKB/ICAM1/FASN/VEGFA/IL1B	6
BP	GO:0002053	positive regulation of mesenchymal cell proliferation	3/210	23/18800	0.002063303	0.006704551	0.003137371	KDR/STAT1/MYC	3
BP	GO:0003323	type B pancreatic cell development	3/210	23/18800	0.002063303	0.006704551	0.003137371	GSK3B/AKT1/BAD	3
BP	GO:0046697	decidualization	3/210	23/18800	0.002063303	0.006704551	0.003137371	PTGS2/PPARD/SPP1	3
BP	GO:0060353	regulation of cell adhesion molecule production	3/210	23/18800	0.002063303	0.006704551	0.003137371	CAV1/IL1B/CXCL8	3
BP	GO:0060444	branching involved in mammary gland duct morphogenesis	3/210	23/18800	0.002063303	0.006704551	0.003137371	PGR/AR/ESR1	3
BP	GO:0060571	morphogenesis of an epithelial fold	3/210	23/18800	0.002063303	0.006704551	0.003137371	AR/EGFR/HIF1A	3

BP	GO:0090208	positive regulation of triglyceride metabolic process	3/210	23/18800	0.002063303	0.006704551	0.003137371	LDLR/SREBF1/DGAT2	3
BP	GO:0030512	negative regulation of transforming growth factor beta receptor signaling pathway	5/210	81/18800	0.002135758	0.006924094	0.003240105	PPARG/PPARA/TP53/HSPA5/CAV1	5
BP	GO:0031016	pancreas development	5/210	81/18800	0.002135758	0.006924094	0.003240105	GSK3B/AKT1/BAD/IL6R/IGF2	5
BP	GO:0045445	myoblast differentiation	5/210	81/18800	0.002135758	0.006924094	0.003240105	MAPK14/PPARD/RB1/CXCL10/IGFBP3	5
BP	GO:0030850	prostate gland development	4/210	49/18800	0.002163501	0.006987369	0.003269714	AR/ESR1/CYP19A1/MMP2	4
BP	GO:0043330	response to exogenous dsRNA	4/210	49/18800	0.002163501	0.006987369	0.003269714	MAPK3/MAPK1/NFKBIA/CAV1	4
BP	GO:0097300	programmed necrotic cell death	4/210	49/18800	0.002163501	0.006987369	0.003269714	BAX/TP53/CASP8/CAV1	4
BP	GO:1904894	positive regulation of	4/210	49/18800	0.002163501	0.006987369	0.003269714	IL4/CYP1B1/IL10RA/IL6R	4

		receptor signaling pathway via STAT							
BP	GO:1990928	response to amino acid starvation	4/210	49/18800	0.002163501	0.006987369	0.003269714	MAPK8/MAPK3/MAPK1/CDKN1A	4
BP	GO:0035966	response to topologically incorrect protein	7/210	160/18800	0.002169634	0.007001852	0.003276491	HSP90AB1/BAX/CCND1/HSPA5/HSPB1/NFE2L2/HSF1	7
BP	GO:0071774	response to fibroblast growth factor	6/210	119/18800	0.002204301	0.007108327	0.003326316	ELK1/MYC/CCL2/CXCL8/COL1A1/RUNX2	6
BP	GO:0050806	positive regulation of synaptic transmission	7/210	161/18800	0.002247235	0.007241279	0.00338853	PTGS2/ADRA1A/GSK3B/DRD1/MAPK1/CA2/CCL2	7
BP	GO:0009205	purine ribonucleoside triphosphate metabolic process	5/210	82/18800	0.002254144	0.007258035	0.003396372	IL4/ATP5F1B/PPARA/MYC/PARP1	5
BP	GO:0072655	establishment of protein localization to mitochondrion	6/210	120/18800	0.002299149	0.007397338	0.003461558	AKT1/BAX/MAPK8/SREBF1/NPEPPS/HK2	6
BP	GO:00194	aromatic	13/210	466/1	0.0023	0.007434	0.0034791	MAOB/MAPK14/	13

	39	compound catabolic process		8800	12599	979	72	SLC6A3/AKT1/BAX/HMOX1/CYP1A1/UGT1A1/ABCC1/PRKCA/HSF1/E2F1/PON1	
BP	GO:0002269	leukocyte activation involved in inflammatory response	4/210	50/18800	0.00233133	0.007445904	0.003484284	JUN/IL4/LDLR/IFNG	4
BP	GO:0042149	cellular response to glucose starvation	4/210	50/18800	0.00233133	0.007445904	0.003484284	BCL2/TP53/HSPA5/NFE2L2	4
BP	GO:0035162	embryonic hemopoiesis	3/210	24/18800	0.002338789	0.007445904	0.003484284	KDR/VEGFA/HIF1A	3
BP	GO:0036120	cellular response to platelet-derived growth factor stimulus	3/210	24/18800	0.002338789	0.007445904	0.003484284	CCNA2/HAS2/MYC	3
BP	GO:0038095	Fc-epsilon receptor signaling pathway	3/210	24/18800	0.002338789	0.007445904	0.003484284	MAPK10/IKBKB/MAPK8	3
BP	GO:0048143	astrocyte activation	3/210	24/18800	0.002338789	0.007445904	0.003484284	LDLR/IL1B/IFNG	3
BP	GO:1904996	positive regulatio	3/210	24/18800	0.002338789	0.007445904	0.003484284	RELA/SELE/ALOX5	3

		n of leukocyte adhesion to vascular endothelial cell							
BP	GO:2000209	regulation of anoikis	3/210	24/18800	0.002338789	0.007445904	0.003484284	BCL2/CAV1/CHEK2	3
BP	GO:2000353	positive regulation of endothelial cell apoptotic process	3/210	24/18800	0.002338789	0.007445904	0.003484284	AKR1C3/CCL2/CD40LG	3
BP	GO:2000637	positive regulation of miRNA-mediated gene silencing	3/210	24/18800	0.002338789	0.007445904	0.003484284	STAT3/EGFR/TP53	3
BP	GO:2000679	positive regulation of transcription regulatory region DNA binding	3/210	24/18800	0.002338789	0.007445904	0.003484284	RB1/IFNG/PARP1	3
BP	GO:2000737	negative regulation of stem cell differentiation	3/210	24/18800	0.002338789	0.007445904	0.003484284	GSK3B/STAT3/NFE2L2	3

BP	GO:2001026	regulation of endothelial cell chemotaxis	3/210	24/18800	0.002338789	0.007445904	0.003484284	KDR/VEGFA/HSPB1	3
BP	GO:0030641	regulation of cellular pH	5/210	83/18800	0.002377238	0.00755133	0.003533618	ATP5F1B/BCL2/MAPK3/MAPK1/CA2	5
BP	GO:0032092	positive regulation of protein binding	5/210	83/18800	0.002377238	0.00755133	0.003533618	HSP90AB1/GSK3B/MMP9/CAV1/HSF1	5
BP	GO:0042509	regulation of tyrosine phosphorylation of STAT protein	5/210	83/18800	0.002377238	0.00755133	0.003533618	IL4/IL6R/CAV1/IFNG/HSF1	5
BP	GO:0016236	macroautophagy	10/210	306/18800	0.002392376	0.007593738	0.003553463	ADRB2/KDR/IL4/AKT1/CASP3/MAPK8/HMOX1/MAPK3/TP53/HIF1A	10
BP	GO:0038127	ERBB signaling pathway	6/210	121/18800	0.00239704	0.00760286	0.003557731	NCF1/AKT1/MAPK1/EGFR/MMP9/EGF	6
BP	GO:0035637	multicellular organismal signaling	7/210	164/18800	0.002492946	0.007901152	0.003697316	SCN5A/DRD1/CHRM5/SOD1/KCNH2/CAV1/GJA1	7
BP	GO:0010823	negative regulation of mitochondrion organization	4/210	51/18800	0.00250791	0.007924921	0.003708438	PPARG/AKT1/BCL2L1/TP53	4

BP	GO:0043124	negative regulation of I-kappaB kinase/NF-kappaB signaling	4/210	51/18800	0.00250791	0.007924921	0.003708438	ESR1/STAT1/GSTP1/CASP8	4
BP	GO:0045912	negative regulation of carbohydrate metabolic process	4/210	51/18800	0.00250791	0.007924921	0.003708438	GSK3B/PPARA/STAT3/TP53	4
BP	GO:1905710	positive regulation of membrane permeability	4/210	51/18800	0.00250791	0.007924921	0.003708438	GSK3B/BAX/MAPK8/TP53	4
BP	GO:0031639	plasminogen activation	3/210	25/18800	0.002636005	0.008256086	0.003863406	PLAU/PLAT/SERPINE1	3
BP	GO:0032104	regulation of response to extracellular stimulus	3/210	25/18800	0.002636005	0.008256086	0.003863406	RXRA/OPRM1/PPARA	3
BP	GO:0032107	regulation of response to nutrient levels	3/210	25/18800	0.002636005	0.008256086	0.003863406	RXRA/OPRM1/PPARA	3
BP	GO:0036119	response to	3/210	25/18800	0.002636005	0.008256086	0.003863406	CCNA2/HAS2/MYC	3

		platelet-derived growth factor							
BP	GO:0045932	negative regulation of muscle contraction	3/210	25/18800	0.002636005	0.008256086	0.003863406	PTGS2/ADRB2/SOD1	3
BP	GO:0060148	positive regulation of post-transcriptional gene silencing	3/210	25/18800	0.002636005	0.008256086	0.003863406	STAT3/EGFR/TP53	3
BP	GO:0060307	regulation of ventricular cardiac muscle cell membrane repolarization	3/210	25/18800	0.002636005	0.008256086	0.003863406	SCN5A/KCNH2/GJA1	3
BP	GO:1900370	positive regulation of post-transcriptional gene silencing by RNA	3/210	25/18800	0.002636005	0.008256086	0.003863406	STAT3/EGFR/TP53	3
BP	GO:1902993	positive regulation of amyloid precursor protein	3/210	25/18800	0.002636005	0.008256086	0.003863406	RELA/CASP3/IFNG	3

		catabolic process							
BP	GO:0009142	nucleoside triphosphate biosynthetic process	5/210	85/18800	0.002637982	0.008256086	0.003863406	IL4/ATP5F1B/PPARA/MYC/PARP1	5
BP	GO:0055072	iron ion homeostasis	5/210	85/18800	0.002637982	0.008256086	0.003863406	HMOX1/SOD1/HIF1A/MYC/IFNG	5
BP	GO:0098586	cellular response to virus	5/210	85/18800	0.002637982	0.008256086	0.003863406	MAPK14/BAX/HIF1A/CXCL10/CHUK	5
BP	GO:2000177	regulation of neural precursor cell proliferation	5/210	85/18800	0.002637982	0.008256086	0.003863406	SLC6A4/VEGFA/EGF/TP53/HIF1A	5
BP	GO:0001959	regulation of cytokine-mediated signaling pathway	7/210	166/18800	0.002667903	0.008337441	0.003901476	HSP90AB1/PPARG/IKBKB/GSTP1/CASP8/HIF1A/CAV1	7
BP	GO:2000058	regulation of ubiquitin-dependent protein catabolic process	7/210	166/18800	0.002667903	0.008337441	0.003901476	HSP90AB1/GSK3B/CDK2/AKT1/EGF/CAV1/NFE2L2	7
BP	GO:0006636	unsaturated fatty acid biosynth	4/210	52/18800	0.002693465	0.008380326	0.003921543	PTGS2/PTGS1/AKR1C3/IL1B	4

		etic process							
BP	GO:0031103	axon regeneration	4/210	52/18800	0.002693465	0.008380326	0.003921543	JUN/BCL2/MMP2/SPP1	4
BP	GO:0043392	negative regulation of DNA binding	4/210	52/18800	0.002693465	0.008380326	0.003921543	JUN/HMOX1/NFKBIA/E2F1	4
BP	GO:0046622	positive regulation of organ growth	4/210	52/18800	0.002693465	0.008380326	0.003921543	MAPK14/AKT1/CDK1/IGF2	4
BP	GO:0086009	membrane repolarization	4/210	52/18800	0.002693465	0.008380326	0.003921543	SCN5A/KCNH2/CAV1/GJA1	4
BP	GO:1902930	regulation of alcohol biosynthetic process	4/210	52/18800	0.002693465	0.008380326	0.003921543	SOD1/APOB/SREBF1/POR	4
BP	GO:0033044	regulation of chromosome organization	8/210	212/18800	0.002696064	0.008382273	0.003922454	CDK2/MAPK3/MAPK1/RB1/MYC/CCNB1/TOP2A/PARP1	8
BP	GO:0034250	positive regulation of cellular amide metabolic process	7/210	167/18800	0.002758843	0.008564918	0.004007923	RELA/CASP3/CYP1B1/EIF6/ERBB2/IFNG/NFE2L2	7
BP	GO:0060538	skeletal muscle organ development	7/210	167/18800	0.002758843	0.008564918	0.004007923	MAPK14/BCL2/PPP3CA/RB1/FOS/CAV1/IGF2	7

BP	GO:0006112	energy reserve metabolic process	5/210	86/18800	0.002775849	0.008605132	0.004026741	GSK3B/PYGM/AKT1/MYC/IGF2	5
BP	GO:0033627	cell adhesion mediated by integrin	5/210	86/18800	0.002775849	0.008605132	0.004026741	DPP4/ICAM1/CYP1B1/PLAU/SERPINE1	5
BP	GO:0034329	cell junction assembly	12/210	420/18800	0.002784677	0.008626201	0.0040366	ACHE/GABRA1/KDR/DRD1/IKBKB/BCL2/VEGFA/PRKCA/CAV1/GJA1/IL1B/CLDN4	12
BP	GO:0070585	protein localization to mitochondrion	6/210	125/18800	0.002820264	0.008730074	0.004085207	AKT1/BAX/MAPK8/SREBF1/NPEPPS/HK2	6
BP	GO:0071385	cellular response to glucocorticoid stimulus	4/210	53/18800	0.002888216	0.008927405	0.004177547	CYP1B1/UGT1A1/CASP9/PLAT	4
BP	GO:1900087	positive regulation of G1/S transition of mitotic cell cycle	4/210	53/18800	0.002888216	0.008927405	0.004177547	AKT1/CYP1A1/CCND1/EGFR	4
BP	GO:0007260	tyrosine phosphorylation of STAT protein	5/210	87/18800	0.002918858	0.009002461	0.004212669	IL4/IL6R/CAV1/IFNG/HSF1	5
BP	GO:0009144	purine nucleoside	5/210	87/18800	0.002918858	0.009002461	0.004212669	IL4/ATP5F1B/PPARA/MYC/PARP1	5

		triphosphate metabolic process							
BP	GO:0045069	regulation of viral genome replication	5/210	87/18800	0.002918858	0.009002461	0.004212669	BCL2/SLPI/PPARA/CXCL8/TOP2A	5
BP	GO:0030878	thyroid gland development	3/210	26/18800	0.002955505	0.009095674	0.004256288	MAPK3/MAPK1/RAF1	3
BP	GO:0060352	cell adhesion molecule production	3/210	26/18800	0.002955505	0.009095674	0.004256288	CAV1/IL1B/CXCL8	3
BP	GO:0060740	prostate gland epithelium morphogenesis	3/210	26/18800	0.002955505	0.009095674	0.004256288	AR/ESR1/MMP2	3
BP	GO:0006959	humoral immune response	10/210	317/18800	0.003081797	0.009468895	0.004430935	BCL2/ALOX5/SLPI/IL1B/CCL2/CXCL8/IFNG/CXCL11/CXCL2/CXCL10	10
BP	GO:0002067	glandular epithelial cell differentiation	4/210	54/18800	0.003092384	0.009468895	0.004430935	GSK3B/AKT1/FASN/BAD	4
BP	GO:0030520	intracellular estrogen receptor signaling pathway	4/210	54/18800	0.003092384	0.009468895	0.004430935	AR/ESR1/ESR2/PARP1	4

BP	GO:0045661	regulation of myoblast differentiation	4/210	54/18800	0.003092384	0.009468895	0.004430935	MAPK14/PPARD/CXCL10/IGFBP3	4
BP	GO:0071715	icosanoid transport	4/210	54/18800	0.003092384	0.009468895	0.004430935	NOS2/ABCC1/IL1B/IL1A	4
BP	GO:0086002	cardiac muscle cell action potential involved in contraction	4/210	54/18800	0.003092384	0.009468895	0.004430935	SCN5A/KCNH2/CAV1/GJA1	4
BP	GO:1903202	negative regulation of oxidative stress-induced cell death	4/210	54/18800	0.003092384	0.009468895	0.004430935	AKT1/HIF1A/HSPB1/NFE2L2	4
BP	GO:0045165	cell fate commitment	9/210	266/18800	0.003105016	0.009493882	0.004442628	AR/PPARG/KDR/BCL2/CASP3/STAT3/IL6R/TP53/RUNX2	9
BP	GO:0090596	sensory organ morphogenesis	9/210	266/18800	0.003105016	0.009493882	0.004442628	KDR/BCL2/BAX/MAPK3/MAPK1/SOD1/STAT3/VEGFA/HIF1A	9
BP	GO:0090092	regulation of transmembrane receptor protein serine/threonine kinase	9/210	267/18800	0.003182988	0.009725288	0.004550914	HSP90AB1/PPARG/KDR/TNFAIP6/PPARA/TP53/HSPA5/CAV1/PARP1	9

		signaling pathway							
BP	GO:0009199	ribonucleoside triphosphate metabolic process	5/210	89/18800	0.003220733	0.00981942	0.004594962	IL4/ATP5F1B/PPARA/MYC/PARP1	5
BP	GO:0044070	regulation of anion transport	5/210	89/18800	0.003220733	0.00981942	0.004594962	ABAT/CES1/CA2/IL1B/IL1A	5
BP	GO:0051781	positive regulation of cell division	5/210	89/18800	0.003220733	0.00981942	0.004594962	CAT/VEGFA/IL1B/IL1A/IGF2	5
BP	GO:0044242	cellular lipid catabolic process	8/210	219/18800	0.003287604	0.009993862	0.004676592	PPARD/AKT1/CYP1B1/AKR1C3/LDLR/APOB/PLB1/PPARA	8
BP	GO:0002092	positive regulation of receptor internalization	3/210	27/18800	0.003297813	0.009993862	0.004676592	SELE/VEGFA/EGF	3
BP	GO:0002675	positive regulation of acute inflammatory response	3/210	27/18800	0.003297813	0.009993862	0.004676592	PTGS2/IL1B/PTGER3	3
BP	GO:0009065	glutamine family amino acid catabolic process	3/210	27/18800	0.003297813	0.009993862	0.004676592	NOS2/GOT1/NOS3	3
BP	GO:00107	regulatio	3/210	27/18	0.0032	0.009993	0.0046765	MAPK3/MAPK1/	3

	58	n of macrophage chemotaxis		800	97813	862	92	CYP19A1	
BP	GO:0030262	apoptotic nuclear changes	3/210	27/18800	0.003297813	0.009993862	0.004676592	BAX/TOP2A/HSF1	3
BP	GO:0035774	positive regulation of insulin secretion involved in cellular response to glucose stimulus	3/210	27/18800	0.003297813	0.009993862	0.004676592	PPARD/BAD/HIF1A	3
BP	GO:0071280	cellular response to copper ion	3/210	27/18800	0.003297813	0.009993862	0.004676592	CYP1A1/NFE2L2/HSF1	3
BP	GO:0010518	positive regulation of phospholipase activity	4/210	55/18800	0.003306187	0.009993862	0.004676592	ADRA1A/ESR1/SELE/EGFR	4
BP	GO:0048008	platelet-derived growth factor receptor signaling pathway	4/210	55/18800	0.003306187	0.009993862	0.004676592	F7/VEGFA/F3/PLAT	4
BP	GO:0061098	positive regulation of	4/210	55/18800	0.003306187	0.009993862	0.004676592	ADRA1A/NCF1/EGF/ERBB3	4

		protein tyrosine kinase activity							
BP	GO:1905517	macrophage migration	4/210	55/18800	0.003306187	0.009993862	0.004676592	MAPK3/MAPK1/CYP19A1/CCL2	4
BP	GO:0042475	odontogenesis of dentin-containing tooth	5/210	90/18800	0.003379816	0.010194659	0.004770554	SCN5A/BAX/PPARA/SERPINE1/RUNX2	5
BP	GO:0060191	regulation of lipase activity	5/210	90/18800	0.003379816	0.010194659	0.004770554	ADRA1A/ESR1/SELE/EGFR/POR	5
BP	GO:0061097	regulation of protein tyrosine kinase activity	5/210	90/18800	0.003379816	0.010194659	0.004770554	ADRA1A/NCF1/EGF/CAV1/ERBB3	5
BP	GO:0017015	regulation of transforming growth factor beta receptor signaling pathway	6/210	130/18800	0.003425196	0.010316885	0.004827749	HSP90AB1/PPARG/PPARA/TP53/HSPA5/CAV1	6
BP	GO:0042476	odontogenesis	6/210	130/18800	0.003425196	0.010316885	0.004827749	SCN5A/BAX/PPARA/SERPINE1/COL1A1/RUNX2	6
BP	GO:0010676	positive regulation of cellular carbohydrate metaboli	4/210	56/18800	0.003529839	0.010601999	0.004961167	AKT1/PPARA/IGF2/DGAT2	4

		c process							
BP	GO:0043331	response to dsRNA	4/210	56/18800	0.003529839	0.010601999	0.004961167	MAPK3/MAPK1/NFKBIA/CAV1	4
BP	GO:0061756	leukocyte adhesion to vascular endothelial cell	4/210	56/18800	0.003529839	0.010601999	0.004961167	RELA/SELE/VCAM1/ALOX5	4
BP	GO:2000300	regulation of synaptic vesicle exocytosis	4/210	56/18800	0.003529839	0.010601999	0.004961167	ADRA1A/GSK3B/DRD1/PRKCB	4
BP	GO:0006275	regulation of DNA replication	6/210	131/18800	0.003556931	0.010675822	0.004995713	CCNA2/CDK2/CDK1/EGFR/EGF/TP53	6
BP	GO:0007254	JNK cascade	7/210	175/18800	0.003574768	0.01072178	0.005017219	MAPK10/NCF1/MAPK8/GSTP1/EGFR/IL1B/IL1A	7
BP	GO:0042133	neurotransmitter metabolic process	3/210	28/18800	0.00366343	0.01094904	0.005123564	ACHE/MAOB/ABAT	3
BP	GO:0048873	homeostasis of number of cells within a tissue	3/210	28/18800	0.00366343	0.01094904	0.005123564	BCL2/BAX/NOS3	3
BP	GO:0060512	prostate gland morphogenesis	3/210	28/18800	0.00366343	0.01094904	0.005123564	AR/ESR1/MMP2	3
BP	GO:0072539	T-helper 17 cell different	3/210	28/18800	0.00366343	0.01094904	0.005123564	IL4/STAT3/IL6R	3

		iation							
BP	GO:1903649	regulation of cytoplasmic transport	3/210	28/18800	0.00366343	0.01094904	0.005123564	MAPK3/MAPK1/MAP2	3
BP	GO:0022898	regulation of transmembrane transporter activity	9/210	273/18800	0.003683825	0.011002255	0.005148466	CHRM3/ADRB2/OPRM1/BCL2/GSTM2/MMP9/CAV1/CCL2/IFNG	9
BP	GO:2000241	regulation of reproductive process	7/210	176/18800	0.00368842	0.011008235	0.005151264	AR/ESR1/PLB1/VEGFA/GJA1/PLAT/IL1A	7
BP	GO:0071333	cellular response to glucose stimulus	6/210	132/18800	0.003692398	0.011012369	0.005153199	PPARD/ICAM1/PPP3CA/BAD/RAF1/HIF1A	6
BP	GO:0038066	p38MAPK cascade	4/210	57/18800	0.003763554	0.01120099	0.005241463	MAPK14/NCF1/VEGFA/IL1B	4
BP	GO:0060350	endochondral bone morphogenesis	4/210	57/18800	0.003763554	0.01120099	0.005241463	POR/COL1A1/COL3A1/RUNX2	4
BP	GO:2000142	regulation of DNA-templated transcription, initiation	4/210	57/18800	0.003763554	0.01120099	0.005241463	ESR1/JUN/CDK4/TP53	4
BP	GO:0044089	positive regulation of cellular compon	13/210	494/18800	0.003801353	0.011297654	0.005286697	ESR1/KDR/GSK3B/CDK2/BAX/MMP1/MMP3/VEGFA/TP53/PRKCA/CAV1/IFNG/HSF1	13

		ent biogenesis							
BP	GO:0072503	cellular divalent inorganic cation homeostasis	13/210	494/18800	0.003801353	0.011297654	0.005286697	ADRA1A/ADRA1B/ADRA1D/ESR1/DRD1/BCL2/BAX/GSTM2/CAV1/PTGER3/PRKCB/CXCL11/CXCL10	13
BP	GO:0006885	regulation of pH	5/210	93/18800	0.00389095	0.011547774	0.00540374	ATP5F1B/BCL2/MAPK3/MAPK1/CA2	5
BP	GO:0045185	maintenance of protein location	5/210	93/18800	0.00389095	0.011547774	0.00540374	AKT1/NFKBIA/HSPA5/CAV1/HK2	5
BP	GO:0006941	striated muscle contraction	7/210	178/18800	0.00392395	0.011629461	0.005441964	SCN5A/ADRA1A/ADRA1B/GSTM2/KCNH2/CAV1/GJA1	7
BP	GO:0060759	regulation of response to cytokine stimulus	7/210	178/18800	0.00392395	0.011629461	0.005441964	HSP90AB1/PPARG/IKBKB/GSTP1/CASP8/HIF1A/CAV1	7
BP	GO:0006352	DNA-templated transcription, initiation	6/210	134/18800	0.003974779	0.01174731	0.005497112	ESR1/JUN/CDK4/TP53/MYC/E2F2	6
BP	GO:0019079	viral genome replication	6/210	134/18800	0.003974779	0.01174731	0.005497112	BCL2/SLPI/PPARA/CCL2/CXCL8/TOP2A	6
BP	GO:0071331	cellular response to hexose stimulus	6/210	134/18800	0.003974779	0.01174731	0.005497112	PPARD/ICAM1/PPP3CA/BAD/RAF1/HIF1A	6
BP	GO:2001235	positive regulation of	6/210	134/18800	0.003974779	0.01174731	0.005497112	BAX/BAD/SOD1/TP53/CAV1/MYC	6

		apoptotic signaling pathway							
BP	GO:0006584	catecholamine metabolic process	4/210	58/18800	0.004007541	0.011819463	0.005530875	MAOB/DRD1/SLC6A3/ABAT	4
BP	GO:0009712	catechol-containing compound metabolic process	4/210	58/18800	0.004007541	0.011819463	0.005530875	MAOB/DRD1/SLC6A3/ABAT	4
BP	GO:0045761	regulation of adenylate cyclase activity	4/210	58/18800	0.004007541	0.011819463	0.005530875	ADRB2/DRD1/ADRB1/RAF1	4
BP	GO:0002021	response to dietary excess	3/210	29/18800	0.004052829	0.011878788	0.005558636	ADRB2/MAPK14/ADRB1	3
BP	GO:0003309	type B pancreatic cell differentiation	3/210	29/18800	0.004052829	0.011878788	0.005558636	GSK3B/AKT1/BAD	3
BP	GO:0006921	cellular component disassembly involved in execution phase of apoptosi	3/210	29/18800	0.004052829	0.011878788	0.005558636	BAX/TOP2A/HSF1	3

		s							
BP	GO:0007271	synaptic transmission, cholinergic	3/210	29/18800	0.004052829	0.011878788	0.005558636	CHRM3/ACHE/NQO1	3
BP	GO:0010464	regulation of mesenchymal cell proliferation	3/210	29/18800	0.004052829	0.011878788	0.005558636	KDR/STAT1/MYC	3
BP	GO:0019674	NAD metabolic process	3/210	29/18800	0.004052829	0.011878788	0.005558636	TP53/NQO1/HK2	3
BP	GO:0051968	positive regulation of synaptic transmission, glutamatergic	3/210	29/18800	0.004052829	0.011878788	0.005558636	PTGS2/DRD1/CCL2	3
BP	GO:0097421	liver regeneration	3/210	29/18800	0.004052829	0.011878788	0.005558636	HMOX1/CCND1/MYC	3
BP	GO:0099625	ventricular cardiac muscle cell membrane repolarization	3/210	29/18800	0.004052829	0.011878788	0.005558636	SCN5A/KCNH2/GJA1	3
BP	GO:0002042	cell migration involved in sprouting	5/210	94/18800	0.004072983	0.011913194	0.005574736	PTGS2/KDR/AKT1/HMOX1/VEGFA	5

		angiogenesis							
BP	GO:0035249	synaptic transmission, glutamatergic	5/210	94/18800	0.004072983	0.011913194	0.005574736	PTGS2/GRIA2/DRD1/CCL2/PLAT	5
BP	GO:1905954	positive regulation of lipid localization	5/210	94/18800	0.004072983	0.011913194	0.005574736	APOB/CYP19A1/IL1B/IL1A/SPP1	5
BP	GO:0090101	negative regulation of transmembrane receptor protein serine/threonine kinase signaling pathway	6/210	135/18800	0.004121821	0.012047745	0.005637699	PPARG/TNFAIP6/PPARA/TP53/HSPA5/CAV1	6
BP	GO:2001257	regulation of cation channel activity	7/210	180/18800	0.00417073	0.012182318	0.005700672	ADRB2/OPRM1/GSTM2/MMP9/CAV1/CCL2/IFNG	7
BP	GO:0046942	carboxylic acid transport	9/210	279/18800	0.004244568	0.01238947	0.005797608	NOS2/ABCC1/ABAT/CES1/MYC/GJA1/IL1B/IL1A/ABCG2	9
BP	GO:0060079	excitatory postsynaptic potential	5/210	95/18800	0.004261022	0.012413728	0.00580896	ADRB2/OPRM1/GSK3B/AKT1/PPP3CA	5
BP	GO:0031102	neuron projection regenera	4/210	59/18800	0.004262009	0.012413728	0.00580896	JUN/BCL2/MMP2/SPP1	4

		tion							
BP	GO:0035773	insulin secretion involved in cellular response to glucose stimulus	4/210	59/18800	0.004262009	0.012413728	0.00580896	PPARD/BAD/RAF1/HIF1A	4
BP	GO:0046700	heterocycle catabolic process	12/210	443/18800	0.00426457	0.012413728	0.00580896	DPEP1/MAPK14/AKT1/BAX/HMOX1/CYP1A1/UGT1A1/ABCC1/PRKCA/NFE2L2/HSF1/E2F1	12
BP	GO:0071326	cellular response to monosaccharide stimulus	6/210	136/18800	0.00427285	0.01242931	0.005816251	PPARD/ICAM1/PPP3CA/BAD/RAF1/HIF1A	6
BP	GO:0045619	regulation of lymphocyte differentiation	7/210	181/18800	0.004298443	0.012495201	0.005847084	IL4/BAD/SOD1/ERBB2/IL2RA/IFNG/IRF1	7
BP	GO:0032434	regulation of proteasomal ubiquitin-dependent protein catabolic process	6/210	137/18800	0.004427929	0.012851663	0.00601389	HSP90AB1/GSK3B/CDK2/AKT1/CAV1/NFE2L2	6
BP	GO:0006942	regulation of striated muscle	5/210	96/18800	0.004455175	0.012851663	0.00601389	SCN5A/ADRA1A/ADRA1B/GSTM2/CAV1	5

		contraction							
BP	GO:0030316	osteoclast differentiation	5/210	96/18800	0.004455175	0.012851663	0.00601389	MAPK14/IL4/TNFAIP6/FOS/IFNG	5
BP	GO:0001975	response to amphetamine	3/210	30/18800	0.00446646	0.012851663	0.00601389	DRD1/PPP3CA/SOD1	3
BP	GO:0006734	NADH metabolic process	3/210	30/18800	0.00446646	0.012851663	0.00601389	TP53/NQO1/HK2	3
BP	GO:0007202	activation of phospholipase C activity	3/210	30/18800	0.00446646	0.012851663	0.00601389	ADRA1A/SELE/EGFR	3
BP	GO:0008299	isoprenoid biosynthetic process	3/210	30/18800	0.00446646	0.012851663	0.00601389	CYP1A1/AKR1C3/HMGCR	3
BP	GO:0033198	response to ATP	3/210	30/18800	0.00446646	0.012851663	0.00601389	PTGS2/SOD1/IL1B	3
BP	GO:0034368	protein-lipid complex remodeling	3/210	30/18800	0.00446646	0.012851663	0.00601389	MTTP/APOB/MPO	3
BP	GO:0034369	plasma lipoprotein particle remodeling	3/210	30/18800	0.00446646	0.012851663	0.00601389	MTTP/APOB/MPO	3
BP	GO:0040018	positive regulation of multicellular organism	3/210	30/18800	0.00446646	0.012851663	0.00601389	SLC6A3/BCL2/IGF2	3

		growth							
BP	GO:0042744	hydrogen peroxide catabolic process	3/210	30/18800	0.00446646	0.012851663	0.00601389	CAT/DUOX2/MPO	3
BP	GO:0060142	regulation of syncytium formation by plasma membrane fusion	3/210	30/18800	0.00446646	0.012851663	0.00601389	MAPK14/IL4/CXCL10	3
BP	GO:0060603	mammary gland duct morphogenesis	3/210	30/18800	0.00446646	0.012851663	0.00601389	PGR/AR/ESR1	3
BP	GO:0099623	regulation of cardiac muscle cell membrane repolarization	3/210	30/18800	0.00446646	0.012851663	0.00601389	SCN5A/KCNH2/GJA1	3
BP	GO:0046888	negative regulation of hormone secretion	4/210	60/18800	0.004527161	0.013017505	0.006091495	PPP3CA/SREBF1/GJA1/IL1B	4
BP	GO:0044264	cellular polysaccharide metabolic process	5/210	97/18800	0.00465555	0.013377618	0.006260008	GSK3B/PYGM/AKT1/HAS2/IGF2	5
BP	GO:00442	cellular	12/210	449/1	0.0047	0.013610	0.0063688	DPEP1/MAPK14/	12

	70	nitrogen compound catabolic process		8800	39707	233	6	AKT1/BAX/HMOX1/CYP3A4/UGT1A1/ABCC1/POR/PRKCA/HSF1/E2F1	
BP	GO:0045732	positive regulation of protein catabolic process	8/210	233/18800	0.004769546	0.013686663	0.006404625	GSK3B/AKT1/LDLR/EGF/CAV1/IL1B/IFNG/NFE2L2	8
BP	GO:0099601	regulation of neurotransmitter receptor activity	4/210	61/18800	0.004803202	0.013773935	0.006445463	ADRB2/OPRM1/CCL2/IFNG	4
BP	GO:0001906	cell killing	7/210	185/18800	0.004839106	0.013867533	0.006489262	HSP90AB1/NOS2/IL4/MAPK8/ICAM1/BCL2L1/IFNG	7
BP	GO:0034620	cellular response to unfolded protein	5/210	98/18800	0.004862251	0.013915082	0.006511513	BAX/CCND1/HSPA5/NFE2L2/HSF1	5
BP	GO:0060349	bone morphogenesis	5/210	98/18800	0.004862251	0.013915082	0.006511513	HAS2/POR/COL1A1/COL3A1/RUNX2	5
BP	GO:0048738	cardiac muscle tissue development	8/210	234/18800	0.004892258	0.013980179	0.006541975	SCN5A/ADRA1A/MAPK14/CDK1/PPARA/VEGFA/GJA1/ERBB3	8
BP	GO:0005979	regulation of glycogen biosynthetic process	3/210	31/18800	0.004904749	0.013980179	0.006541975	GSK3B/AKT1/IGF2	3
BP	GO:0010962	regulation of	3/210	31/18800	0.004904749	0.013980179	0.006541975	GSK3B/AKT1/IGF2	3

		glucan biosynthetic process							
BP	GO:0035767	endothelial cell chemotaxis	3/210	31/18800	0.004904749	0.013980179	0.006541975	KDR/VEGFA/HSPB1	3
BP	GO:0043171	peptide catabolic process	3/210	31/18800	0.004904749	0.013980179	0.006541975	DPEP1/LTA4H/NPEPPS	3
BP	GO:0062098	regulation of programmed necrotic cell death	3/210	31/18800	0.004904749	0.013980179	0.006541975	TP53/CASP8/CAV1	3
BP	GO:0050768	negative regulation of neurogenesis	6/210	140/18800	0.004918114	0.013999483	0.006551008	PPP3CA/LDLR/TP53/IL1B/SPP1/MAP2	6
BP	GO:1903305	regulation of regulated secretory pathway	6/210	140/18800	0.004918114	0.013999483	0.006551008	ADRA1A/GSK3B/DRD1/IL4/HMOX1/PRKCB	6
BP	GO:0044403	biological process involved in symbiotic interaction	9/210	286/18800	0.004980774	0.014152699	0.006622705	HSP90AB1/JUN/CDK1/ICAM1/LDLR/EGFR/BCL2L1/CAV1/CXCL8	9
BP	GO:0002040	sprouting angiogenesis	7/210	186/18800	0.004981937	0.014152699	0.006622705	PTGS2/KDR/AKT1/HMOX1/ALOX5/VEGFA/E2F2	7

BP	GO:0051302	regulation of cell division	7/210	186/18800	0.004981937	0.014152699	0.006622705	CAT/VEGFA/BCL2L1/MYC/IL1B/IL1A/IGF2	7
BP	GO:0030199	collagen fibril organization	4/210	62/18800	0.005090328	0.014441299	0.006757754	CYP1B1/RB1/COL1A1/COL3A1	4
BP	GO:0032731	positive regulation of interleukin-1 beta production	4/210	62/18800	0.005090328	0.014441299	0.006757754	STAT3/CASP8/HSPB1/IFNG	4
BP	GO:0002138	retinoic acid biosynthetic process	2/210	10/18800	0.005267954	0.014632439	0.006847197	CYP1A1/AKR1C3	2
BP	GO:0002676	regulation of chronic inflammatory response	2/210	10/18800	0.005267954	0.014632439	0.006847197	IL4/CYP19A1	2
BP	GO:0006702	androgen biosynthetic process	2/210	10/18800	0.005267954	0.014632439	0.006847197	HSD3B2/HSD3B1	2
BP	GO:0010749	regulation of nitric oxide mediated signal transduction	2/210	10/18800	0.005267954	0.014632439	0.006847197	EGFR/VEGFA	2
BP	GO:0019062	virion attachment to host cell	2/210	10/18800	0.005267954	0.014632439	0.006847197	HSP90AB1/ICAM1	2
BP	GO:0019614	catechol-containi	2/210	10/18800	0.005267954	0.014632439	0.006847197	MAOB/SLC6A3	2

		ng compound catabolic process							
BP	GO:0021936	regulation of cerebellar granule cell precursor proliferation	2/210	10/18800	0.005267954	0.014632439	0.006847197	SLC6A4/EGF	2
BP	GO:0030638	polyketide metabolic process	2/210	10/18800	0.005267954	0.014632439	0.006847197	AKR1B1/AKR1C3	2
BP	GO:0030647	aminoglycoside antibiotic metabolic process	2/210	10/18800	0.005267954	0.014632439	0.006847197	AKR1B1/AKR1C3	2
BP	GO:0033327	Leydig cell differentiation	2/210	10/18800	0.005267954	0.014632439	0.006847197	AR/CCND1	2
BP	GO:0038003	G protein-coupled opioid receptor signaling pathway	2/210	10/18800	0.005267954	0.014632439	0.006847197	OPRM1/OPRD1	2
BP	GO:0042368	vitamin D biosynthetic	2/210	10/18800	0.005267954	0.014632439	0.006847197	CYP3A4/IFNG	2

		process							
BP	GO:0042420	dopamine catabolic process	2/210	10/18800	0.005267954	0.014632439	0.006847197	MAOB/SLC6A3	2
BP	GO:0042424	catecholamine catabolic process	2/210	10/18800	0.005267954	0.014632439	0.006847197	MAOB/SLC6A3	2
BP	GO:0044598	doxorubicin metabolic process	2/210	10/18800	0.005267954	0.014632439	0.006847197	AKR1B1/AKR1C3	2
BP	GO:0045348	positive regulation of MHC class II biosynthetic process	2/210	10/18800	0.005267954	0.014632439	0.006847197	IL4/IFNG	2
BP	GO:0045542	positive regulation of cholesterol biosynthetic process	2/210	10/18800	0.005267954	0.014632439	0.006847197	SREBF1/POR	2
BP	GO:0048563	post-embryonic animal organ morphogenesis	2/210	10/18800	0.005267954	0.014632439	0.006847197	KDR/BAX	2
BP	GO:0051918	negative regulation of fibrinolysis	2/210	10/18800	0.005267954	0.014632439	0.006847197	THBD/SERPINE1	2
BP	GO:00600	vagina	2/210	10/18	0.0052	0.014632	0.0068471	ESR1/BAX	2

	68	development		800	67954	439	97		
BP	GO:0060346	bone trabecula formation	2/210	10/18800	0.005267954	0.014632439	0.006847197	MMP2/COL1A1	2
BP	GO:0060371	regulation of atrial cardiac muscle cell membrane depolarization	2/210	10/18800	0.005267954	0.014632439	0.006847197	SCN5A/GJA1	2
BP	GO:0060439	trachea morphogenesis	2/210	10/18800	0.005267954	0.014632439	0.006847197	MAPK3/MAPK1	2
BP	GO:0070391	response to lipoteichoic acid	2/210	10/18800	0.005267954	0.014632439	0.006847197	MAPK14/RELA	2
BP	GO:0071223	cellular response to lipoteichoic acid	2/210	10/18800	0.005267954	0.014632439	0.006847197	MAPK14/RELA	2
BP	GO:0071313	cellular response to caffeine	2/210	10/18800	0.005267954	0.014632439	0.006847197	CHEK1/GSTM2	2
BP	GO:0072203	cell proliferation involved in metanephros development	2/210	10/18800	0.005267954	0.014632439	0.006847197	STAT1/MYC	2
BP	GO:0090037	positive regulatio	2/210	10/18800	0.005267954	0.014632439	0.006847197	ADRA1A/VEGFA	2

		n of protein kinase C signaling							
BP	GO:0106120	positive regulation of sterol biosynthetic process	2/210	10/18800	0.005267954	0.014632439	0.006847197	SREBF1/POR	2
BP	GO:1902510	regulation of apoptotic DNA fragmentation	2/210	10/18800	0.005267954	0.014632439	0.006847197	BAX/HSF1	2
BP	GO:1904181	positive regulation of membrane depolarization	2/210	10/18800	0.005267954	0.014632439	0.006847197	KDR/PARP1	2
BP	GO:1904526	regulation of microtubule binding	2/210	10/18800	0.005267954	0.014632439	0.006847197	HSF1/MAP2	2
BP	GO:0030947	regulation of vascular endothelial growth factor receptor signaling pathway	3/210	32/18800	0.005368097	0.014852318	0.006950089	HIF1A/IL1B/PRKCB	3
BP	GO:0034367	protein-containing	3/210	32/18800	0.005368097	0.014852318	0.006950089	MTTP/APOB/MPO	3

		complex remodeling							
BP	GO:0035883	enteroendocrine cell differentiation	3/210	32/18800	0.005368097	0.014852318	0.006950089	GSK3B/AKT1/BAD	3
BP	GO:0090183	regulation of kidney development	3/210	32/18800	0.005368097	0.014852318	0.006950089	STAT1/VEGFA/MYC	3
BP	GO:1900745	positive regulation of p38MAPK cascade	3/210	32/18800	0.005368097	0.014852318	0.006950089	NCF1/VEGFA/IL1B	3
BP	GO:1902253	regulation of intrinsic apoptotic signaling pathway by p53 class mediator	3/210	32/18800	0.005368097	0.014852318	0.006950089	BCL2/TP53/MYC	3
BP	GO:0006949	syncytium formation	4/210	63/18800	0.005388738	0.014890025	0.006967734	MAPK14/IL4/CYP19A1/CXCL10	4
BP	GO:0070059	intrinsic apoptotic signaling pathway in response to endoplas	4/210	63/18800	0.005388738	0.014890025	0.006967734	BCL2/BAX/BCL2L1/TP53	4

		mic reticulum stress							
BP	GO:0046395	carboxylic acid catabolic process	8/210	238/18800	0.005407488	0.014922418	0.006982892	NOS2/PPARD/AKT1/PPARA/GOT1/ABAT/NOS3/PON1	8
BP	GO:0098657	import into cell	8/210	238/18800	0.005407488	0.014922418	0.006982892	SLC6A2/SLC6A4/DRD1/SLC6A3/AKT1/KCNH2/IL10RA/IFNG	8
BP	GO:0051607	defense response to virus	9/210	290/18800	0.005443908	0.015012132	0.007024873	RELA/IL4/BCL2/STAT1/BCL2L1/IL1B/IFNG/CXCL10/IRF1	9
BP	GO:0001508	action potential	6/210	143/18800	0.005447067	0.015012132	0.007024873	SCN5A/ADRA1A/DRD1/KCNH2/CAV1/GJA1	6
BP	GO:0070167	regulation of biomineral tissue development	5/210	101/18800	0.005521381	0.015207073	0.007116095	ADRB2/ALOX5/HIF1A/GJA1/NOS3	5
BP	GO:0140546	defense response to symbiont	9/210	291/18800	0.005564743	0.015316567	0.007167333	RELA/IL4/BCL2/STAT1/BCL2L1/IL1B/IFNG/CXCL10/IRF1	9
BP	GO:0032507	maintenance of protein location in cell	4/210	64/18800	0.005698622	0.015654624	0.007325525	AKT1/HSPA5/CAV1/HK2	4
BP	GO:0045670	regulation of osteoclast differentiation	4/210	64/18800	0.005698622	0.015654624	0.007325525	IL4/TNFAIP6/FOS/IFNG	4
BP	GO:0098930	axonal transport	4/210	64/18800	0.005698622	0.015654624	0.007325525	SOD1/HIF1A/HSPB1/MAP2	4

BP	GO:0051701	biological process involved in interaction with host	7/210	191/18800	0.00574422	0.015769685	0.007379368	CDK1/ICAM1/LDLR/EGFR/BCL2L1/CAV1/CXCL8	7
BP	GO:1902106	negative regulation of leukocyte differentiation	5/210	102/18800	0.00575445	0.015787564	0.007387734	IL4/TNFAIP6/ERBB2/MYC/IRF1	5
BP	GO:0031333	negative regulation of protein-containing complex assembly	6/210	145/18800	0.005822099	0.01595255	0.007464939	GSK3B/OPRD1/RAF1/HSPA5/HSF1/MAP2	6
BP	GO:0051961	negative regulation of nervous system development	6/210	145/18800	0.005822099	0.01595255	0.007464939	PPP3CA/LDLR/TP53/IL1B/SPP1/MAP2	6
BP	GO:0001662	behavioral fear response	3/210	33/18800	0.005856884	0.016016862	0.007495033	DPP4/DRD1/BCL2	3
BP	GO:0010661	positive regulation of muscle cell apoptotic process	3/210	33/18800	0.005856884	0.016016862	0.007495033	PPARG/TP53/IFNG	3
BP	GO:0090022	regulation of	3/210	33/18800	0.005856884	0.016016862	0.007495033	DPP4/TNFAIP6/CXCL8	3

		neutrophil chemotaxis							
BP	GO:0007626	locomotory behavior	7/210	192/18800	0.005906604	0.016142436	0.007553795	DPP4/NCOA2/DRD1/SLC6A3/OPRD1/SOD1/ABAT	7
BP	GO:0016054	organic acid catabolic process	8/210	242/18800	0.0059632	0.016286631	0.007621271	NOS2/PPARD/AKT1/PPARA/GOT1/ABAT/NOS3/PON1	8
BP	GO:0031058	positive regulation of histone modification	5/210	103/18800	0.005994372	0.016340243	0.007646358	MAPK3/SREBF1/VEGFA/TP53/IL1B	5
BP	GO:0110149	regulation of biomineralization	5/210	103/18800	0.005994372	0.016340243	0.007646358	ADRB2/ALOX5/HIF1A/GJA1/NOS3	5
BP	GO:1903076	regulation of protein localization to plasma membrane	5/210	103/18800	0.005994372	0.016340243	0.007646358	AR/AKT1/EGFR/BCL2L1/IFNG	5
BP	GO:0002695	negative regulation of leukocyte activation	7/210	193/18800	0.006072397	0.016542315	0.007740917	IL4/CASP3/HMOX1/LDLR/ERBB2/IL2RA/IRF1	7
BP	GO:0010517	regulation of phospholipase activity	4/210	66/18800	0.006353575	0.017125872	0.00801399	ADRA1A/ESR1/SELE/EGFR	4
BP	GO:00504	amyloid	4/210	66/18	0.0063	0.017125	0.0080139	RELA/CASP3/BA	4

	35	-beta metabolic process		800	53575	872	9	CE2/IFNG	
BP	GO:0070265	necrotic cell death	4/210	66/18800	0.006353575	0.017125872	0.00801399	BAX/TP53/CASP8/CAV1	4
BP	GO:1902808	positive regulation of cell cycle G1/S phase transition	4/210	66/18800	0.006353575	0.017125872	0.00801399	AKT1/CYP1A1/CCND1/EGFR	4
BP	GO:0002209	behavioral defense response	3/210	34/18800	0.006371467	0.017125872	0.00801399	DPP4/DRD1/BCL2	3
BP	GO:0006691	leukotriene metabolic process	3/210	34/18800	0.006371467	0.017125872	0.00801399	LTA4H/ALOX5/ABCC1	3
BP	GO:0090322	regulation of superoxide metabolic process	3/210	34/18800	0.006371467	0.017125872	0.00801399	GSTP1/SOD1/NFE2L2	3
BP	GO:1902692	regulation of neuroblast proliferation	3/210	34/18800	0.006371467	0.017125872	0.00801399	VEGFA/TP53/HIF1A	3
BP	GO:0006527	arginine catabolic process	2/210	11/18800	0.006391454	0.017125872	0.00801399	NOS2/NOS3	2
BP	GO:0006703	estrogen biosynthetic	2/210	11/18800	0.006391454	0.017125872	0.00801399	HSD3B1/CYP19A1	2

		process							
BP	GO:0016102	diterpenoid biosynthetic process	2/210	11/18800	0.006391454	0.017125872	0.00801399	CYP1A1/AKR1C3	2
BP	GO:0031284	positive regulation of guanylate cyclase activity	2/210	11/18800	0.006391454	0.017125872	0.00801399	NOS2/NOS3	2
BP	GO:0033148	positive regulation of intracellular estrogen receptor signaling pathway	2/210	11/18800	0.006391454	0.017125872	0.00801399	AR/PARP1	2
BP	GO:0034350	regulation of glial cell apoptotic process	2/210	11/18800	0.006391454	0.017125872	0.00801399	PRKCA/CCL2	2
BP	GO:0040015	negative regulation of multicellular organism growth	2/210	11/18800	0.006391454	0.017125872	0.00801399	ADRB2/ADRB1	2
BP	GO:0042167	heme catabolic process	2/210	11/18800	0.006391454	0.017125872	0.00801399	HMOX1/ABCC1	2
BP	GO:0046149	pigment catabolic	2/210	11/18800	0.006391454	0.017125872	0.00801399	HMOX1/ABCC1	2

		process							
BP	GO:0051081	nuclear membrane disassembly	2/210	11/18800	0.006391454	0.017125872	0.00801399	PRKCA/PRKCB	2
BP	GO:0070243	regulation of thymocyte apoptotic process	2/210	11/18800	0.006391454	0.017125872	0.00801399	TP53/HIF1A	2
BP	GO:0097278	complement-dependent cytotoxicity	2/210	11/18800	0.006391454	0.017125872	0.00801399	HSP90AB1/IL4	2
BP	GO:0106049	regulation of cellular response to osmotic stress	2/210	11/18800	0.006391454	0.017125872	0.00801399	PTGS2/BAD	2
BP	GO:1900222	negative regulation of amyloid-beta clearance	2/210	11/18800	0.006391454	0.017125872	0.00801399	HMGCR/IFNG	2
BP	GO:1901030	positive regulation of mitochondrial outer membrane permeabilization involved in	2/210	11/18800	0.006391454	0.017125872	0.00801399	GSK3B/MAPK8	2

		apoptotic signaling pathway							
BP	GO:1905050	positive regulation of metallopeptidase activity	2/210	11/18800	0.006391454	0.017125872	0.00801399	STAT3/CLDN4	2
BP	GO:1990440	positive regulation of transcription from RNA polymerase II promoter in response to endoplasmic reticulum stress	2/210	11/18800	0.006391454	0.017125872	0.00801399	TP53/HSPA5	2
BP	GO:2001223	negative regulation of neuron migration	2/210	11/18800	0.006391454	0.017125872	0.00801399	STAT3/COL3A1	2
BP	GO:0006576	cellular biogenic amine metabolic process	5/210	105/18800	0.00649519	0.017392857	0.008138925	MAOB/DRD1/SLC6A3/ABAT/ODC1	5
BP	GO:0009566	fertilization	7/210	196/18800	0.006590665	0.017637402	0.008253359	AR/BAX/CDK1/APOB/PLB1/BCL2L1/PLAT	7
BP	GO:00070	cell-cell	6/210	149/1	0.0066	0.017715	0.0082899	IKBKB/PRKCA/C	6

	43	junction assembly		8800	28219	575	4	AV1/GJA1/IL1B/CLDN4	
BP	GO:0016525	negative regulation of angiogenesis	6/210	149/18800	0.006628219	0.017715575	0.00828994	PPARG/STAT1/ALOX5/SERPINE1/CXCL10/E2F2	6
BP	GO:0002294	CD4-positive, alpha-beta T cell differentiation involved in immune response	4/210	67/18800	0.006699014	0.017815098	0.008336511	IL4/STAT3/IL6R/IFNG	4
BP	GO:0032024	positive regulation of insulin secretion	4/210	67/18800	0.006699014	0.017815098	0.008336511	PPARD/BAD/ABAT/HIF1A	4
BP	GO:0033692	cellular polysaccharide biosynthetic process	4/210	67/18800	0.006699014	0.017815098	0.008336511	GSK3B/AKT1/HAS2/IGF2	4
BP	GO:0042531	positive regulation of tyrosine phosphorylation of STAT protein	4/210	67/18800	0.006699014	0.017815098	0.008336511	IL4/IL6R/IFNG/HSF1	4
BP	GO:0051926	negative regulation of calcium ion	4/210	67/18800	0.006699014	0.017815098	0.008336511	PTGS2/BCL2/GSTM2/NOS3	4

		transport							
BP	GO:0071300	cellular response to retinoic acid	4/210	67/18800	0.006699014	0.017815098	0.008336511	SLC6A4/GSK3B/MYC/COL1A1	4
BP	GO:0072678	T cell migration	4/210	67/18800	0.006699014	0.017815098	0.008336511	ICAM1/CCL2/CXCL11/CXCL10	4
BP	GO:1901224	positive regulation of NIK/NF-kappaB signaling	4/210	67/18800	0.006699014	0.017815098	0.008336511	NR3C2/RELA/EGFR/IL1B	4
BP	GO:0007631	feeding behavior	5/210	106/18800	0.006756291	0.017937931	0.0083939 9	OPRM1/DRD1/OPRD1/STAT3/FOS	5
BP	GO:1903707	negative regulation of hemopoiesis	5/210	106/18800	0.006756291	0.017937931	0.0083939 9	IL4/TNFAIP6/ERBB2/MYC/IRF1	5
BP	GO:0008544	epidermis development	10/210	355/18800	0.006757874	0.017937931	0.0083939 9	PPARD/RELA/BCL2/CASP3/AKR1C3/SOD1/PPARA/FOSL2/EGFR/IL1A	10
BP	GO:0014706	striated muscle tissue development	8/210	248/18800	0.006876999	0.018242733	0.008536621	SCN5A/ADRA1A/MAPK14/CDK1/PPARA/VEGFA/GJA1/ERBB3	8
BP	GO:0007095	mitotic G2 DNA damage checkpoint signaling	3/210	35/18800	0.0069 1218	0.018256236	0.0085429 4	CHEK1/CDK1/CDKN1A	3
BP	GO:0016242	negative regulation of macroau	3/210	35/18800	0.0069 1218	0.018256236	0.0085429 4	AKT1/HMOX1/TP53	3

		tophagy							
BP	GO:0019320	hexose catabolic process	3/210	35/18800	0.00691218	0.018256236	0.00854294	BAD/TP53/HK2	3
BP	GO:0035633	maintenance of blood-brain barrier	3/210	35/18800	0.00691218	0.018256236	0.00854294	PTGS2/VEGFA/GJA1	3
BP	GO:0086005	ventricular cardiac muscle cell action potential	3/210	35/18800	0.00691218	0.018256236	0.00854294	SCN5A/KCNH2/CAV1	3
BP	GO:0097345	mitochondrial outer membrane permeabilization	3/210	35/18800	0.00691218	0.018256236	0.00854294	GSK3B/BAX/MAPK8	3
BP	GO:1905898	positive regulation of response to endoplasmic reticulum stress	3/210	35/18800	0.00691218	0.018256236	0.00854294	BAX/CAV1/NFE2L2	3
BP	GO:0022037	metencephalon development	5/210	107/18800	0.007024656	0.018541773	0.008676556	SCN5A/BCL2/ABAT/TP53/HSPA5	5
BP	GO:0002287	alpha-beta T cell activation involved in immune	4/210	68/18800	0.007056668	0.018578208	0.00869360 6	IL4/STAT3/IL6R/IFNG	4

		response							
BP	GO:0002293	alpha-beta T cell differentiation involved in immune response	4/210	68/18800	0.007056668	0.018578208	0.008693606	IL4/STAT3/IL6R/IFNG	4
BP	GO:0046626	regulation of insulin receptor signaling pathway	4/210	68/18800	0.007056668	0.018578208	0.008693606	CDK4/IL1B/PRKCB/IGF2	4
BP	GO:0060193	positive regulation of lipase activity	4/210	68/18800	0.007056668	0.018578208	0.008693606	ADRA1A/ESR1/SELE/EGFR	4
BP	GO:2000181	negative regulation of blood vessel morphogenesis	6/210	151/18800	0.007060331	0.018578208	0.008693606	PPARG/STAT1/ALOX5/SERPINE1/CXCL10/E2F2	6
BP	GO:1903828	negative regulation of protein localization	7/210	199/18800	0.007141177	0.018779305	0.008787709	OPRM1/GSK3B/PPP3CA/HMGCR/SREBF1/BCL2L1/IL1B	7
BP	GO:1901343	negative regulation of vasculature development	6/210	152/18800	0.007283873	0.019142703	0.008957759	PPARG/STAT1/ALOX5/SERPINE1/CXCL10/E2F2	6
BP	GO:0007173	epidermal growth	5/210	108/18800	0.007300385	0.019162383	0.008966969	NCF1/AKT1/EGFR/MMP9/EGF	5

		factor receptor signaling pathway							
BP	GO:2000060	positive regulation of ubiquitin-dependent protein catabolic process	5/210	108/18800	0.007300385	0.019162383	0.008966969	GSK3B/AKT1/EGF/CAV1/NFE2L2	5
BP	GO:0032370	positive regulation of lipid transport	4/210	69/18800	0.007426714	0.019457899	0.009105254	CYP19A1/IL1B/IL1A/SPP1	4
BP	GO:0032922	circadian regulation of gene expression	4/210	69/18800	0.007426714	0.019457899	0.009105254	NCOA2/AHR/PPARA/TOP1	4
BP	GO:0050922	negative regulation of chemotaxis	4/210	69/18800	0.007426714	0.019457899	0.009105254	DPP4/TNFAIP6/CYP19A1/CCL2	4
BP	GO:0032350	regulation of hormone metabolic process	3/210	36/18800	0.007479337	0.019547536	0.009147199	AKR1C3/POR/HIF1A	3
BP	GO:0043368	positive T cell selection	3/210	36/18800	0.007479337	0.019547536	0.009147199	BCL2/STAT3/IL6R	3
BP	GO:0045742	positive regulation of	3/210	36/18800	0.007479337	0.019547536	0.009147199	NCF1/MMP9/EGF	3

		epidermal growth factor receptor signaling pathway							
BP	GO:1903146	regulation of autophagy of mitochondrion	3/210	36/18800	0.007479337	0.019547536	0.009147199	SREBF1/TP53/HIF1A	3
BP	GO:0044409	entry into host	6/210	153/18800	0.007512489	0.019597007	0.009170349	CDK1/ICAM1/LDLR/EGFR/CAV1/CXCL8	6
BP	GO:0002285	lymphocyte activation involved in immune response	7/210	201/18800	0.007526674	0.019597007	0.009170349	IL4/ICAM1/STAT3/IL6R/TP53/IFNG/CD40LG	7
BP	GO:0015711	organic anion transport	10/210	361/18800	0.007563568	0.019597007	0.009170349	NOS2/MTTP/ABCC1/ABAT/CES1/MYC/GJA1/IL1B/IL1A/ABCG2	10
BP	GO:0002024	diet induced thermogenesis	2/210	12/18800	0.007613624	0.019597007	0.009170349	ADRB2/ADRB1	2
BP	GO:0002674	negative regulation of acute inflammatory response	2/210	12/18800	0.007613624	0.019597007	0.009170349	IL4/GSTP1	2
BP	GO:0010269	response to selenium ion	2/210	12/18800	0.007613624	0.019597007	0.009170349	MAOB/APOB	2

BP	GO:0014745	negative regulation of muscle adaptation	2/210	12/18800	0.007613624	0.019597007	0.009170349	PPARG/NOS3	2
BP	GO:0014854	response to inactivity	2/210	12/18800	0.007613624	0.019597007	0.009170349	SCN5A/CAT	2
BP	GO:0030397	membrane disassembly	2/210	12/18800	0.007613624	0.019597007	0.009170349	PRKCA/PRKCB	2
BP	GO:0030656	regulation of vitamin metabolic process	2/210	12/18800	0.007613624	0.019597007	0.009170349	AKR1C3/IFNG	2
BP	GO:0031953	negative regulation of protein autophosphorylation	2/210	12/18800	0.007613624	0.019597007	0.009170349	JUN/CAV1	2
BP	GO:0033145	positive regulation of intracellular steroid hormone receptor signaling pathway	2/210	12/18800	0.007613624	0.019597007	0.009170349	AR/PARP1	2
BP	GO:0033629	negative regulation of cell adhesion mediated by	2/210	12/18800	0.007613624	0.019597007	0.009170349	CYP1B1/SERPINE1	2

		integrin							
BP	GO:0035865	cellular response to potassium ion	2/210	12/18800	0.007613624	0.019597007	0.009170349	SOD1/HSF1	2
BP	GO:0044650	adhesion of symbiont to host cell	2/210	12/18800	0.007613624	0.019597007	0.009170349	HSP90AB1/ICAM1	2
BP	GO:0046322	negative regulation of fatty acid oxidation	2/210	12/18800	0.007613624	0.019597007	0.009170349	AKT1/DGAT2	2
BP	GO:0046541	saliva secretion	2/210	12/18800	0.007613624	0.019597007	0.009170349	CHRM3/CHRM1	2
BP	GO:0051549	positive regulation of keratinocyte migration	2/210	12/18800	0.007613624	0.019597007	0.009170349	HAS2/MMP9	2
BP	GO:0060134	prepulse inhibition	2/210	12/18800	0.007613624	0.019597007	0.009170349	DRD1/SLC6A3	2
BP	GO:0061517	macrophage proliferation	2/210	12/18800	0.007613624	0.019597007	0.009170349	MAPK3/MAPK1	2
BP	GO:0071372	cellular response to follicle-stimulating hormone stimulus	2/210	12/18800	0.007613624	0.019597007	0.009170349	POR/PLAT	2
BP	GO:01500	regulatio	2/210	12/18	0.0076	0.019597	0.0091703	MAPK8/VEGFA	2

	65	n of deacetylase activity		800	13624	007	49		
BP	GO:1901503	ether biosynthetic process	2/210	12/18800	0.007613624	0.019597007	0.009170349	ALOX5/FASN	2
BP	GO:1903624	regulation of DNA catabolic process	2/210	12/18800	0.007613624	0.019597007	0.009170349	BAX/HSF1	2
BP	GO:1903800	positive regulation of production of miRNAs involved in gene silencing by miRNA	2/210	12/18800	0.007613624	0.019597007	0.009170349	EGFR/TP53	2
BP	GO:0048592	eye morphogenesis	6/210	154/18800	0.007746244	0.019926285	0.009324434	KDR/BCL2/BAX/STAT3/VEGFA/HIF1A	6
BP	GO:0002548	monocyte chemotaxis	4/210	70/18800	0.007809326	0.020076396	0.009394678	IL6R/CCL2/SERPINE1/CXCL10	4
BP	GO:0002062	chondrocyte differentiation	5/210	110/18800	0.007874337	0.020182445	0.009444303	MAPK14/RB1/POR/COL3A1/RUNX2	5
BP	GO:0032611	interleukin-1 beta production	5/210	110/18800	0.007874337	0.020182445	0.009444303	GSTP1/STAT3/CASP8/HSPB1/IFNG	5
BP	GO:0032651	regulation of interleukin-1 beta	5/210	110/18800	0.007874337	0.020182445	0.009444303	GSTP1/STAT3/CASP8/HSPB1/IFNG	5

		production							
BP	GO:0048259	regulation of receptor-mediated endocytosis	5/210	110/18800	0.007874337	0.020182445	0.009444303	IL4/SELE/VEGFA/EGF/SERPINE1	5
BP	GO:1901222	regulation of NIK/NF-kappaB signaling	5/210	110/18800	0.007874337	0.020182445	0.009444303	NR3C2/RELA/EGFR/NFKBIA/IL1B	5
BP	GO:0009165	nucleotide biosynthetic process	8/210	255/18800	0.008072828	0.020617569	0.009647917	NOS2/IL4/ATP5F1B/PPARA/ACACA/MYC/NOS3/PARP1	8
BP	GO:0046326	positive regulation of glucose import	3/210	37/18800	0.008073232	0.020617569	0.009647917	MAPK14/AKT1/NFE2L2	3
BP	GO:0048009	insulin-like growth factor receptor signaling pathway	3/210	37/18800	0.008073232	0.020617569	0.009647917	AR/AKT1/IGFBP3	3
BP	GO:0070873	regulation of glycogen metabolic process	3/210	37/18800	0.008073232	0.020617569	0.009647917	GSK3B/AKT1/IGF2	3
BP	GO:0071542	dopaminergic neuron different	3/210	37/18800	0.008073232	0.020617569	0.009647917	GSK3B/VEGFA/HIF1A	3

		iation							
BP	GO:2000279	negative regulation of DNA biosynthetic process	3/210	37/18800	0.008073232	0.020617569	0.009647917	CHEK1/CDKN1A/TP53	3
BP	GO:0017157	regulation of exocytosis	7/210	204/18800	0.00813352	0.020759049	0.009714123	ADRA1A/GSK3B/DRD1/IL4/HMOX1/PRKCB/IFNG	7
BP	GO:0002764	immune response-regulating signaling pathway	12/210	482/18800	0.008162972	0.020821707	0.009743443	ESR1/MAPK10/RELA/IKBKB/BCL2/BAX/MAPK8/MAPK1/NFKBIA/CAV1/PRKCB/IRF1	12
BP	GO:0009141	nucleoside triphosphate metabolic process	5/210	111/18800	0.008172758	0.020834155	0.009749268	IL4/ATP5F1B/PPARA/MYC/PARP1	5
BP	GO:0050766	positive regulation of phagocytosis	4/210	71/18800	0.008204673	0.020890435	0.009775604	SOD1/IL1B/CCL2/IFNG	4
BP	GO:2001259	positive regulation of cation channel activity	4/210	71/18800	0.008204673	0.020890435	0.009775604	ADRB2/GSTM2/CCL2/IFNG	4
BP	GO:0045216	cell-cell junction organization	7/210	205/18800	0.008343601	0.021231439	0.009935176	IKBKB/VEGFA/PRKCA/CAV1/GJA1/IL1B/CLDN4	7
BP	GO:1901293	nucleoside phospha	8/210	257/18800	0.008441567	0.021467866	0.010045811	NOS2/IL4/ATP5F1B/PPARA/ACACA/MYC/NOS3/PAR	8

		te biosynthetic process						P1	
BP	GO:1903364	positive regulation of cellular protein catabolic process	6/210	157/18800	0.008478963	0.021550062	0.010084274	GSK3B/AKT1/LDLR/EGF/CAV1/NFE2L2	6
BP	GO:0042246	tissue regeneration	4/210	72/18800	0.008612922	0.021877439	0.010237469	PPARD/PPP3CA/CDKN1A/GJA1	4
BP	GO:1903362	regulation of cellular protein catabolic process	8/210	258/18800	0.008630606	0.021909256	0.010252358	HSP90AB1/GSK3B/CDK2/AKT1/LDLR/EGF/CAV1/NFE2L2	8
BP	GO:0007617	mating behavior	3/210	38/18800	0.008694137	0.022031027	0.01030934	SLC6A4/DRD1/ABAT	3
BP	GO:0043403	skeletal muscle tissue regeneration	3/210	38/18800	0.008694137	0.022031027	0.01030934	PPARD/PPP3CA/GJA1	3
BP	GO:1901186	positive regulation of ERBB signaling pathway	3/210	38/18800	0.008694137	0.022031027	0.01030934	NCF1/MMP9/EGF	3
BP	GO:0046631	alpha-beta T cell activation	6/210	158/18800	0.008733896	0.022118578	0.010350309	IL4/BCL2/STAT3/IL6R/IFNG/IRF1	6
BP	GO:0006836	neurotransmitter transport	7/210	207/18800	0.008775721	0.022211254	0.010393677	ADRA1A/SLC6A2/SLC6A4/GSK3B/DRD1/SLC6A3/PRKCB	7

BP	GO:0044344	cellular response to fibroblast growth factor stimulus	5/210	113/18800	0.008792976	0.022241673	0.010407911	MYC/CCL2/CXCL8/COL1A1/RUNX2	5
BP	GO:0019336	phenol-containing compound catabolic process	2/210	13/18800	0.008932139	0.022433351	0.010497606	MAOB/SLC6A3	2
BP	GO:0031000	response to caffeine	2/210	13/18800	0.008932139	0.022433351	0.010497606	CHEK1/GSTM2	2
BP	GO:0031392	regulation of prostaglandin biosynthetic process	2/210	13/18800	0.008932139	0.022433351	0.010497606	PTGS2/IL1B	2
BP	GO:0042362	fat-soluble vitamin biosynthetic process	2/210	13/18800	0.008932139	0.022433351	0.010497606	CYP3A4/IFNG	2
BP	GO:0045741	positive regulation of epidermal growth factor-activated receptor activity	2/210	13/18800	0.008932139	0.022433351	0.010497606	NCF1/EGF	2
BP	GO:0045820	negative regulation of	2/210	13/18800	0.008932139	0.022433351	0.010497606	PPARA/STAT3	2

		glycolytic process							
BP	GO:0061029	eyelid development in camera-type eye	2/210	13/18800	0.008932139	0.022433351	0.010497606	JUN/EGFR	2
BP	GO:0061430	bone trabecula morphogenesis	2/210	13/18800	0.008932139	0.022433351	0.010497606	MMP2/COL1A1	2
BP	GO:0070486	leukocyte aggregation	2/210	13/18800	0.008932139	0.022433351	0.010497606	HAS2/IL1B	2
BP	GO:0071415	cellular response to purine-containing compound	2/210	13/18800	0.008932139	0.022433351	0.010497606	CHEK1/GSTM2	2
BP	GO:0072540	T-helper 17 cell lineage commitment	2/210	13/18800	0.008932139	0.022433351	0.010497606	STAT3/IL6R	2
BP	GO:0072683	T cell extravasation	2/210	13/18800	0.008932139	0.022433351	0.010497606	ICAM1/CCL2	2
BP	GO:0050792	regulation of viral process	6/210	159/18800	0.008994279	0.022571677	0.010562335	BCL2/STAT1/SLPI/PPARA/CXCL8/TOP2A	6
BP	GO:0030100	regulation of endocytosis	7/210	208/18800	0.008997845	0.022571677	0.010562335	IL4/SELE/PPP3CA/VEGFA/EGF/CAV1/SERPINE1	7
BP	GO:0005977	glycogen metaboli	4/210	73/18800	0.009034235	0.022596228	0.010573824	GSK3B/PYGM/AKT1/IGF2	4

		c process							
BP	GO:0019226	transmission of nerve impulse	4/210	73/18800	0.009034235	0.022596228	0.010573824	SCN5A/DRD1/CHRM5/SOD1	4
BP	GO:0032732	positive regulation of interleukin-1 production	4/210	73/18800	0.009034235	0.022596228	0.010573824	STAT3/CASP8/HSPB1/IFNG	4
BP	GO:0042058	regulation of epidermal growth factor receptor signaling pathway	4/210	73/18800	0.009034235	0.022596228	0.010573824	NCF1/EGFR/MMP9/EGF	4
BP	GO:0045123	cellular extravasation	4/210	73/18800	0.009034235	0.022596228	0.010573824	ICAM1/SELE/VCAM1/CCL2	4
BP	GO:0032941	secretion by tissue	3/210	39/18800	0.009342306	0.023270836	0.010889504	CHRM3/CHRM1/GJA1	3
BP	GO:0042755	eating behavior	3/210	39/18800	0.009342306	0.023270836	0.010889504	OPRM1/OPRD1/STAT3	3
BP	GO:0043267	negative regulation of potassium ion transport	3/210	39/18800	0.009342306	0.023270836	0.010889504	KCNH2/CAV1/NOS3	3
BP	GO:0046365	monosaccharide catabolic process	3/210	39/18800	0.009342306	0.023270836	0.010889504	BAD/TP53/HK2	3
BP	GO:0071634	regulation of	3/210	39/18800	0.009342306	0.023270836	0.010889504	PTGS2/HSP90AB1/HIF1A	3

		transforming growth factor beta production							
BP	GO:0086091	regulation of heart rate by cardiac conduction	3/210	39/18800	0.009342306	0.023270836	0.010889504	SCN5A/KCNH2/CAV1	3
BP	GO:1904994	regulation of leukocyte adhesion to vascular endothelial cell	3/210	39/18800	0.009342306	0.023270836	0.010889504	RELA/SELE/ALOX5	3
BP	GO:0046916	cellular transition metal ion homeostasis	5/210	115/18800	0.009445005	0.023475697	0.010985368	HMOX1/SOD1/HIF1A/MYC/IFNG	5
BP	GO:0006073	cellular glucan metabolic process	4/210	74/18800	0.009468771	0.023475697	0.010985368	GSK3B/PYGM/AKT1/IGF2	4
BP	GO:0006305	DNA alkylation	4/210	74/18800	0.009468771	0.023475697	0.010985368	CYP1A1/FOS/MYC/PARP1	4
BP	GO:0006306	DNA methylation	4/210	74/18800	0.009468771	0.023475697	0.010985368	CYP1A1/FOS/MYC/PARP1	4
BP	GO:0044042	glucan metabolic process	4/210	74/18800	0.009468771	0.023475697	0.010985368	GSK3B/PYGM/AKT1/IGF2	4

BP	GO:0070988	demethylation	4/210	74/18800	0.009468771	0.023475697	0.010985368	CYP3A4/CYP1A2/CYP1A1/POR	4
BP	GO:0072088	nephron epithelium morphogenesis	4/210	74/18800	0.009468771	0.023475697	0.010985368	BCL2/STAT1/VEGFA/MYC	4
BP	GO:0140115	export across plasma membrane	4/210	74/18800	0.009468771	0.023475697	0.010985368	ABCC1/KCNH2/GJA1/ABCG2	4
BP	GO:0032412	regulation of ion transmembrane transporter activity	8/210	263/18800	0.00962376	0.023846038	0.011158668	CHRM3/ADRB2/OPRM1/GSTM2/MMP9/CAV1/CCL2/IFNG	8
BP	GO:0043406	positive regulation of MAP kinase activity	5/210	116/18800	0.009783185	0.024212813	0.011330299	EGFR/VEGFA/EGF/ERBB2/IL1B	5
BP	GO:0044106	cellular amine metabolic process	5/210	116/18800	0.009783185	0.024212813	0.011330299	MAOB/DRD1/SLC6A3/ABAT/ODC1	5
BP	GO:0086003	cardiac muscle cell contraction	4/210	75/18800	0.009916685	0.024528924	0.011478222	SCN5A/KCNH2/CAV1/GJA1	4
BP	GO:0019432	triglyceride biosynthetic process	3/210	40/18800	0.010017973	0.02473624	0.011575235	LDLR/SREBF1/DGAT2	3
BP	GO:0072538	T-helper 17 type immune response	3/210	40/18800	0.010017973	0.02473624	0.011575235	IL4/STAT3/IL6R	3

BP	GO:0099622	cardiac muscle cell membrane repolarization	3/210	40/18800	0.010017973	0.02473624	0.011575235	SCN5A/KCNH2/GJA1	3
BP	GO:0035967	cellular response to topologically incorrect protein	5/210	117/18800	0.0101296	0.024997333	0.011697413	BAX/CCND1/HSPA5/NFE2L2/HSF1	5
BP	GO:0007171	activation of transmembrane receptor protein tyrosine kinase activity	2/210	14/18800	0.010344717	0.025177084	0.011781526	ADRB2/EGF	2
BP	GO:0007512	adult heart development	2/210	14/18800	0.010344717	0.025177084	0.011781526	ADRA1A/GJA1	2
BP	GO:0009886	post-embryonic animal morphogenesis	2/210	14/18800	0.010344717	0.025177084	0.011781526	KDR/BAX	2
BP	GO:0030238	male sex determination	2/210	14/18800	0.010344717	0.025177084	0.011781526	AR/INSRR	2
BP	GO:0031282	regulation of guanylate cyclase activity	2/210	14/18800	0.010344717	0.025177084	0.011781526	NOS2/NOS3	2
BP	GO:0032230	positive regulation of	2/210	14/18800	0.010344717	0.025177084	0.011781526	ADRA1A/CA2	2

		synaptic transmission, GABAergic							
BP	GO:0032306	regulation of prostaglandin secretion	2/210	14/18800	0.010344717	0.025177084	0.011781526	IL1B/IL1A	2
BP	GO:0032308	positive regulation of prostaglandin secretion	2/210	14/18800	0.010344717	0.025177084	0.011781526	IL1B/IL1A	2
BP	GO:0032352	positive regulation of hormone metabolic process	2/210	14/18800	0.010344717	0.025177084	0.011781526	POR/HIF1A	2
BP	GO:0038166	angiotensin-activated signaling pathway	2/210	14/18800	0.010344717	0.025177084	0.011781526	CA2/CAV1	2
BP	GO:0043374	CD8-positive, alpha-beta T cell differentiation	2/210	14/18800	0.010344717	0.025177084	0.011781526	BCL2/IRF1	2
BP	GO:0047484	regulation of response to osmotic stress	2/210	14/18800	0.010344717	0.025177084	0.011781526	PTGS2/BAD	2
BP	GO:00480	regulatio	2/210	14/18	0.0103	0.025177	0.0117815	BCL2/BAX	2

	70	n of developmental pigmentation		800	44717	084	26		
BP	GO:0048148	behavioral response to cocaine	2/210	14/18800	0.010344717	0.025177084	0.011781526	DRD1/ABAT	2
BP	GO:0050930	induction of positive chemotaxis	2/210	14/18800	0.010344717	0.025177084	0.011781526	VEGFA/CXCL8	2
BP	GO:0051547	regulation of keratinocyte migration	2/210	14/18800	0.010344717	0.025177084	0.011781526	HAS2/MMP9	2
BP	GO:0051917	regulation of fibrinolysis	2/210	14/18800	0.010344717	0.025177084	0.011781526	THBD/SERPINE1	2
BP	GO:0061043	regulation of vascular wound healing	2/210	14/18800	0.010344717	0.025177084	0.011781526	ALOX5/SERPINE1	2
BP	GO:0071236	cellular response to antibiotic	2/210	14/18800	0.010344717	0.025177084	0.011781526	TP53/HSPA5	2
BP	GO:0120305	regulation of pigmentation	2/210	14/18800	0.010344717	0.025177084	0.011781526	BCL2/BAX	2
BP	GO:1901722	regulation of cell proliferation involved	2/210	14/18800	0.010344717	0.025177084	0.011781526	IL6R/MYC	2

		in kidney development							
BP	GO:1903894	regulation of IRE1-mediated unfolded protein response	2/210	14/18800	0.010344717	0.025177084	0.011781526	BAX/HSPA5	2
BP	GO:1904294	positive regulation of ERAD pathway	2/210	14/18800	0.010344717	0.025177084	0.011781526	CAV1/NFE2L2	2
BP	GO:1905203	regulation of connective tissue replacement	2/210	14/18800	0.010344717	0.025177084	0.011781526	PPARG/PPP3CA	2
BP	GO:0002292	T cell differentiation involved in immune response	4/210	76/18800	0.01037813	0.02521984	0.01180153 4	IL4/STAT3/IL6R/IFNG	4
BP	GO:0030968	endoplasmic reticulum unfolded protein response	4/210	76/18800	0.01037813	0.02521984	0.011801534	BAX/CCND1/HSPA5/NFE2L2	4
BP	GO:0001764	neuron migration	6/210	164/18800	0.010380099	0.02521984	0.011801534	DRD1/BAX/STAT3/VEGFA/GJA1/COL3A1	6
BP	GO:0006476	protein deacetylation	5/210	118/18800	0.010484342	0.025458547	0.011913236	MAPK8/SREBF1/VEGFA/TP53/IFNG	5
BP	GO:0002251	organ or tissue	3/210	41/18800	0.010721354	0.025930291	0.012133987	NOS2/IL4/IL6R	3

		specific immune response							
BP	GO:0010463	mesenchymal cell proliferation	3/210	41/18800	0.010721354	0.025930291	0.012133987	KDR/STAT1/MYC	3
BP	GO:0032733	positive regulation of interleukin-10 production	3/210	41/18800	0.010721354	0.025930291	0.012133987	IL4/STAT3/CD40LG	3
BP	GO:0072595	maintenance of protein localization in organelle	3/210	41/18800	0.010721354	0.025930291	0.012133987	AKT1/HSPA5/HK2	3
BP	GO:1902110	positive regulation of mitochondrial membrane permeability involved in apoptotic process	3/210	41/18800	0.010721354	0.025930291	0.012133987	GSK3B/BAX/MAPK8	3
BP	GO:1905521	regulation of macrophage migration	3/210	41/18800	0.010721354	0.025930291	0.012133987	MAPK3/MAPK1/CYP19A1	3
BP	GO:2000008	regulation of	3/210	41/18800	0.010721354	0.025930291	0.012133987	HSP90AB1/AKT1/EGF	3

		protein localization to cell surface							
BP	GO:0006304	DNA modification	5/210	119/18800	0.010847501	0.026189638	0.012255347	CYP1A1/CYP1B1/FOS/MYC/PARP1	5
BP	GO:0072676	lymphocyte migration	5/210	119/18800	0.010847501	0.026189638	0.012255347	AKT1/ICAM1/CCL2/CXCL11/CXCL10	5
BP	GO:0072028	nephron morphogenesis	4/210	77/18800	0.010853253	0.026189638	0.012255347	BCL2/STAT1/VEGFA/MYC	4
BP	GO:0086001	cardiac muscle cell action potential	4/210	77/18800	0.010853253	0.026189638	0.012255347	SCN5A/KCNH2/CAV1/GJA1	4
BP	GO:0030203	glycosaminoglycan metabolic process	5/210	120/18800	0.011219169	0.027057246	0.012661341	LYG1/TNFAIP6/HAS2/EGF/IL1B	5
BP	GO:0008088	axo-dendritic transport	4/210	78/18800	0.011342197	0.027307434	0.012778416	SOD1/HIF1A/HSPB1/MAP2	4
BP	GO:0042440	pigment metabolic process	4/210	78/18800	0.011342197	0.027307434	0.012778416	BCL2/HMOX1/UGT1A1/ABCC1	4
BP	GO:0090049	regulation of cell migration involved in sprouting angiogenesis	4/210	78/18800	0.011342197	0.027307434	0.012778416	PTGS2/KDR/HMOX1/VEGFA	4
BP	GO:00085	visual	3/210	42/18	0.0114	0.027448	0.0128445	DRD1/HMGCR/HI	3

	42	learning		800	52646	864	98	F1A	
BP	GO:0030890	positive regulation of B cell proliferation	3/210	42/18800	0.011452646	0.027448864	0.012844598	IL4/BCL2/CDKN1A	3
BP	GO:0031952	regulation of protein autophosphorylation	3/210	42/18800	0.011452646	0.027448864	0.012844598	JUN/VEGFA/CAV1	3
BP	GO:0050691	regulation of defense response to virus by host	3/210	42/18800	0.011452646	0.027448864	0.012844598	IL4/STAT1/IL1B	3
BP	GO:0070266	necroptotic process	3/210	42/18800	0.011452646	0.027448864	0.012844598	TP53/CASP8/CAV1	3
BP	GO:0071354	cellular response to interleukin-6	3/210	42/18800	0.011452646	0.027448864	0.012844598	RELA/STAT3/IL6R	3
BP	GO:0090207	regulation of triglyceride metabolic process	3/210	42/18800	0.011452646	0.027448864	0.012844598	LDLR/SREBF1/DGAT2	3
BP	GO:1904706	negative regulation of vascular associated smooth muscle cell prolifera	3/210	42/18800	0.011452646	0.027448864	0.012844598	PPARG/HMOX1/CDKN1A	3

		tion							
BP	GO:0030282	bone mineralization	5/210	121/18800	0.011599433	0.027753686	0.012987238	PTGS2/ADRB2/ALOX5/HIF1A/GJA1	5
BP	GO:0051209	release of sequestered calcium ion into cytosol	5/210	121/18800	0.011599433	0.027753686	0.012987238	DRD1/BAX/GSTM2/CXCL11/CXCL10	5
BP	GO:1903008	organelle disassembly	5/210	121/18800	0.011599433	0.027753686	0.012987238	CDK1/SREBF1/TP53/HIF1A/HK2	5
BP	GO:0048844	artery morphogenesis	4/210	79/18800	0.011845105	0.027957318	0.013082527	LDLR/APOB/VEGFA/COL3A1	4
BP	GO:0061844	antimicrobial humoral immune response mediated by antimicrobial peptide	4/210	79/18800	0.011845105	0.027957318	0.013082527	CXCL8/CXCL11/CXCL2/CXCL10	4
BP	GO:1901184	regulation of ERBB signaling pathway	4/210	79/18800	0.011845105	0.027957318	0.013082527	NCF1/EGFR/MMP9/EGF	4
BP	GO:0002679	respiratory burst involved in defense response	2/210	15/18800	0.01184911	0.027957318	0.013082527	NCF1/MPO	2
BP	GO:0007567	parturition	2/210	15/18800	0.01184911	0.027957318	0.013082527	CYP1A1/MMP2	2
BP	GO:0010889	regulation of	2/210	15/18800	0.01184911	0.027957318	0.013082527	PPARG/PPARA	2

		sequestering of triglyceride							
BP	GO:0021534	cell proliferation in hindbrain	2/210	15/18800	0.01184911	0.027957318	0.013082527	SLC6A4/EGF	2
BP	GO:0021924	cell proliferation in external granule layer	2/210	15/18800	0.01184911	0.027957318	0.013082527	SLC6A4/EGF	2
BP	GO:0021930	cerebellar granule cell precursor proliferation	2/210	15/18800	0.01184911	0.027957318	0.013082527	SLC6A4/EGF	2
BP	GO:0033127	regulation of histone phosphorylation	2/210	15/18800	0.01184911	0.027957318	0.013082527	MAPK3/IL1B	2
BP	GO:0033599	regulation of mammary gland epithelial cell proliferation	2/210	15/18800	0.01184911	0.027957318	0.013082527	BAX/CCND1	2
BP	GO:0034310	primary alcohol catabolic process	2/210	15/18800	0.01184911	0.027957318	0.013082527	AKR1C3/SULT1E1	2
BP	GO:0042976	activation of Janus	2/210	15/18800	0.01184911	0.027957318	0.013082527	IL4/IL6R	2

		kinase activity							
BP	GO:0043129	surfactant homeostasis	2/210	15/18800	0.01184911	0.027957318	0.013082527	KDR/VEGFA	2
BP	GO:0044406	adhesion of symbiont to host	2/210	15/18800	0.01184911	0.027957318	0.013082527	HSP90AB1/ICAM1	2
BP	GO:0045346	regulation of MHC class II biosynthetic process	2/210	15/18800	0.01184911	0.027957318	0.013082527	IL4/IFNG	2
BP	GO:0045725	positive regulation of glycogen biosynthetic process	2/210	15/18800	0.01184911	0.027957318	0.013082527	AKT1/IGF2	2
BP	GO:0046321	positive regulation of fatty acid oxidation	2/210	15/18800	0.01184911	0.027957318	0.013082527	PPARD/PPARA	2
BP	GO:0061307	cardiac neural crest cell differentiation involved in heart development	2/210	15/18800	0.01184911	0.027957318	0.013082527	MAPK3/MAPK1	2
BP	GO:0061308	cardiac neural	2/210	15/18800	0.01184911	0.027957318	0.013082527	MAPK3/MAPK1	2

		crest cell development involved in heart development							
BP	GO:0071380	cellular response to prostaglandin E stimulus	2/210	15/18800	0.01184911	0.027957318	0.013082527	AKT1/ACACA	2
BP	GO:0072075	metanephric mesenchyme development	2/210	15/18800	0.01184911	0.027957318	0.013082527	STAT1/MYC	2
BP	GO:0106070	regulation of adenylate cyclase-activating G protein-coupled receptor signaling pathway	2/210	15/18800	0.01184911	0.027957318	0.013082527	OPRM1/PRKCA	2
BP	GO:2000402	negative regulation of lymphocyte migration	2/210	15/18800	0.01184911	0.027957318	0.013082527	AKT1/CCL2	2
BP	GO:2001279	regulation of unsaturated fatty	2/210	15/18800	0.01184911	0.027957318	0.013082527	PTGS2/IL1B	2

		acid biosynthetic process							
BP	GO:0007613	memory	5/210	122/18800	0.011988382	0.028223202	0.013206946	PTGS2/SLC6A4/DRD1/SLC2A4/LDLR	5
BP	GO:0014902	myotube differentiation	5/210	122/18800	0.011988382	0.028223202	0.013206946	MAPK14/IL4/BCL2/PPP3CA/CXCL10	5
BP	GO:0019730	antimicrobial humoral response	5/210	122/18800	0.011988382	0.028223202	0.013206946	SLPI/CXCL8/CXCL11/CXCL2/CXCL10	5
BP	GO:0051283	negative regulation of sequestering of calcium ion	5/210	122/18800	0.011988382	0.028223202	0.013206946	DRD1/BAX/GSTM2/CXCL11/CXCL10	5
BP	GO:0048512	circadian behavior	3/210	43/18800	0.012212029	0.028717877	0.013438428	NCOA2/ADRB1/TP53	3
BP	GO:1902686	mitochondrial outer membrane permeabilization involved in programmed cell death	3/210	43/18800	0.012212029	0.028717877	0.013438428	GSK3B/BAX/MAPK8	3
BP	GO:0009152	purine ribonucleotide biosynthetic process	6/210	170/18800	0.012235643	0.028757484	0.013456962	IL4/ATP5F1B/PPARA/ACACA/MYC/PARP1	6
BP	GO:0006367	transcription	4/210	80/18800	0.012362112	0.029006569	0.01357352	ESR1/CDK4/TP53/E2F2	4

		initiation from RNA polymerase II promoter							
BP	GO:0008306	associative learning	4/210	80/18800	0.012362112	0.029006569	0.01357352	DRD1/HMGCR/FOS/HIF1A	4
BP	GO:0032204	regulation of telomere maintenance	4/210	80/18800	0.012362112	0.029006569	0.01357352	MAPK3/MAPK1/MYC/PARP1	4
BP	GO:0002244	hematopoietic progenitor cell differentiation	5/210	123/18800	0.012386101	0.02904681	0.013592351	KDR/BCL2/TP53/TOP2A/NFE2L2	5
BP	GO:0050769	positive regulation of neurogenesis	7/210	222/18800	0.012557476	0.029432452	0.01377281	OPRM1/VEGFA/HIF1A/MYC/IL1B/IFNG/E2F1	7
BP	GO:0042177	negative regulation of protein catabolic process	5/210	124/18800	0.012792678	0.029917677	0.01399987	HSP90AB1/NOS2/RELA/HMGCR/EGFR	5
BP	GO:0051224	negative regulation of protein transport	5/210	124/18800	0.012792678	0.029917677	0.01399987	OPRM1/PPP3CA/HMGCR/SREBF1/IL1B	5
BP	GO:0051282	regulation of sequestering of calcium ion	5/210	124/18800	0.012792678	0.029917677	0.01399987	DRD1/BAX/GSTM2/CXCL11/CXCL10	5

BP	GO:1904375	regulation of protein localization to cell periphery	5/210	124/18800	0.012792678	0.029917677	0.01399987	AR/AKT1/EGFR/BCL2L1/IFNG	5
BP	GO:1903050	regulation of proteolysis involved in cellular protein catabolic process	7/210	223/18800	0.012845554	0.030024803	0.014049999	HSP90AB1/GSK3B/CDK2/AKT1/EGF/CAV1/NFE2L2	7
BP	GO:0010833	telomere maintenance via telomere lengthening	4/210	81/18800	0.012893351	0.030076054	0.014073982	HSP90AB1/MAPK3/MAPK1/PARP1	4
BP	GO:0030500	regulation of bone mineralization	4/210	81/18800	0.012893351	0.030076054	0.014073982	ADRB2/ALOX5/HIF1A/GJA1	4
BP	GO:0055013	cardiac muscle cell development	4/210	81/18800	0.012893351	0.030076054	0.014073982	ADRA1A/CDK1/PPARA/VEGFA	4
BP	GO:0032635	interleukin-6 production	6/210	172/18800	0.012902889	0.030076054	0.014073982	NOS2/STAT3/IL6R/IL1B/IFNG/IL1A	6
BP	GO:0032675	regulation of interleukin-6 producti	6/210	172/18800	0.012902889	0.030076054	0.014073982	NOS2/STAT3/IL6R/IL1B/IFNG/IL1A	6

		on							
BP	GO:0006509	membrane protein ectodomain proteolysis	3/210	44/18800	0.012999665	0.030218708	0.014140736	BACE2/IL1B/IFNG	3
BP	GO:0010828	positive regulation of glucose transmembrane transport	3/210	44/18800	0.012999665	0.030218708	0.014140736	MAPK14/AKT1/NFE2L2	3
BP	GO:0019098	reproductive behavior	3/210	44/18800	0.012999665	0.030218708	0.014140736	SLC6A4/DRD1/ABAT	3
BP	GO:0033173	calcineurin-NFAT signaling cascade	3/210	44/18800	0.012999665	0.030218708	0.014140736	GSK3B/PPP3CA/ERBB3	3
BP	GO:1903573	negative regulation of response to endoplasmic reticulum stress	3/210	44/18800	0.012999665	0.030218708	0.014140736	ALOX5/BCL2L1/HSPA5	3
BP	GO:0050868	negative regulation of T cell activation	5/210	125/18800	0.013208195	0.030686654	0.01435971	IL4/CASP3/ERBB2/IL2RA/IRF1	5
BP	GO:0043367	CD4-positive, alpha-beta T cell different	4/210	82/18800	0.013438952	0.030911155	0.014464764	IL4/STAT3/IL6R/IFNG	4

		iation							
BP	GO:1901983	regulation of protein acetylation	4/210	82/18800	0.013438952	0.030911155	0.014464764	GSK3B/CHEK1/MAPK3/IL1B	4
BP	GO:2001021	negative regulation of response to DNA damage stimulus	4/210	82/18800	0.013438952	0.030911155	0.014464764	BCL2/BCL2L1/CHEK2/HSF1	4
BP	GO:0006089	lactate metabolic process	2/210	16/18800	0.013443113	0.030911155	0.014464764	TP53/HIF1A	2
BP	GO:0032095	regulation of response to food	2/210	16/18800	0.013443113	0.030911155	0.014464764	OPRM1/PPARA	2
BP	GO:0034393	positive regulation of smooth muscle cell apoptotic process	2/210	16/18800	0.013443113	0.030911155	0.014464764	PPARG/IFNG	2
BP	GO:0034638	phosphatidylcholine catabolic process	2/210	16/18800	0.013443113	0.030911155	0.014464764	LDLR/PLB1	2
BP	GO:0035864	response to potassium ion	2/210	16/18800	0.013443113	0.030911155	0.014464764	SOD1/HSF1	2
BP	GO:0045342	MHC class II biosynthetic	2/210	16/18800	0.013443113	0.030911155	0.014464764	IL4/IFNG	2

		process							
BP	GO:0045780	positive regulation of bone resorption	2/210	16/18800	0.013443113	0.030911155	0.014464764	PRKCA/SPP1	2
BP	GO:0051044	positive regulation of membrane protein ectodomain proteolysis	2/210	16/18800	0.013443113	0.030911155	0.014464764	IL1B/IFNG	2
BP	GO:0060330	regulation of response to interferon-gamma	2/210	16/18800	0.013443113	0.030911155	0.014464764	HSP90AB1/PPARG	2
BP	GO:0060334	regulation of interferon-gamma-mediated signaling pathway	2/210	16/18800	0.013443113	0.030911155	0.014464764	HSP90AB1/PPARG	2
BP	GO:0070431	nucleotide-binding oligomerization domain containing 2 signaling pathway	2/210	16/18800	0.013443113	0.030911155	0.014464764	RELA/NFKBIA	2

BP	GO:0070875	positive regulation of glycogen metabolic process	2/210	16/18800	0.013443113	0.030911155	0.014464764	AKT1/IGF2	2
BP	GO:0090336	positive regulation of brown fat cell differentiation	2/210	16/18800	0.013443113	0.030911155	0.014464764	PTGS2/MAPK14	2
BP	GO:1901163	regulation of trophoblast cell migration	2/210	16/18800	0.013443113	0.030911155	0.014464764	VEGFA/GJA1	2
BP	GO:1901741	positive regulation of myoblast fusion	2/210	16/18800	0.013443113	0.030911155	0.014464764	MAPK14/IL4	2
BP	GO:2000696	regulation of epithelial cell differentiation involved in kidney development	2/210	16/18800	0.013443113	0.030911155	0.014464764	STAT1/MMP9	2
BP	GO:0006260	DNA replication	8/210	280/18800	0.013643322	0.031354539	0.014672244	CHEK1/CCNA2/CDK2/CDK1/EGFR/EGF/TP53/TOP1	8
BP	GO:0007520	myoblast fusion	3/210	45/18800	0.013815702	0.031630882	0.014801558	MAPK14/IL4/CXCL10	3
BP	GO:0007622	rhythmic	3/210	45/18800	0.013815702	0.031630882	0.014801558	NCOA2/ADRB1/TP53	3

		behavior							
BP	GO:0010677	negative regulation of cellular carbohydrate metabolic process	3/210	45/18800	0.013815702	0.031630882	0.014801558	GSK3B/PPARA/STAT3	3
BP	GO:0032892	positive regulation of organic acid transport	3/210	45/18800	0.013815702	0.031630882	0.014801558	ABAT/IL1B/IL1A	3
BP	GO:0046006	regulation of activated T cell proliferation	3/210	45/18800	0.013815702	0.031630882	0.014801558	CASP3/IL2RA/IGF2	3
BP	GO:0060612	adipose tissue development	3/210	45/18800	0.013815702	0.031630882	0.014801558	PPARD/CDK4/DGAT2	3
BP	GO:1990090	cellular response to nerve growth factor stimulus	3/210	45/18800	0.013815702	0.031630882	0.014801558	AKT1/HSPA5/E2F1	3
BP	GO:0046486	glycerolipid metabolic process	10/210	396/18800	0.013838331	0.031665619	0.014817813	CHRM5/LDLR/CAT/MTTP/APOB/PLB1/SREBF1/CAV1/PON1/DGAT2	10
BP	GO:0060395	SMAD protein signal transduction	4/210	83/18800	0.013999039	0.03201611	0.014981824	PPARG/JUN/FOS/PARP1	4
BP	GO:0048588	developmental	7/210	227/18800	0.014045573	0.032105246	0.015023535	ADRA1A/HSP90AB1/GSK3B/PPAR	7

		cell growth						A/VEGFA/SPP1/MAP2	
BP	GO:0048705	skeletal system morphogenesis	7/210	228/18800	0.014357707	0.032801066	0.015349141	MAPK14/HAS2/MMP2/POR/COL1A1/COL3A1/RUNX2	7
BP	GO:0051208	sequestering of calcium ion	5/210	128/18800	0.014509218	0.033111579	0.015494445	DRD1/BAX/GSTM2/CXCL11/CXCL10	5
BP	GO:1904950	negative regulation of establishment of protein localization	5/210	128/18800	0.014509218	0.033111579	0.015494445	OPRM1/PPP3CA/HMGCR/SREBF1/IL1B	5
BP	GO:0010921	regulation of phosphatase activity	4/210	84/18800	0.014573733	0.033240948	0.015554982	HSP90AB1/GSK3B/IKBKB/IFNG	4
BP	GO:0005978	glycogen biosynthetic process	3/210	46/18800	0.014660268	0.033277477	0.015572076	GSK3B/AKT1/IGF2	3
BP	GO:0009250	glucan biosynthetic process	3/210	46/18800	0.014660268	0.033277477	0.015572076	GSK3B/AKT1/IGF2	3
BP	GO:0010665	regulation of cardiac muscle cell apoptotic process	3/210	46/18800	0.014660268	0.033277477	0.015572076	TP53/NFE2L2/HSF1	3
BP	GO:0010939	regulation of necrotic cell	3/210	46/18800	0.014660268	0.033277477	0.015572076	TP53/CASP8/CAV1	3

		death							
BP	GO:0031640	killing of cells of another organism	3/210	46/18800	0.014660268	0.033277477	0.015572076	NOS2/BCL2L1/IFNG	3
BP	GO:0032309	icosanoid secretion	3/210	46/18800	0.014660268	0.033277477	0.015572076	NOS2/IL1B/IL1A	3
BP	GO:0045581	negative regulation of T cell differentiation	3/210	46/18800	0.014660268	0.033277477	0.015572076	IL4/ERBB2/IRF1	3
BP	GO:0070741	response to interleukin-6	3/210	46/18800	0.014660268	0.033277477	0.015572076	RELA/STAT3/IL6R	3
BP	GO:1900744	regulation of p38MAPK cascade	3/210	46/18800	0.014660268	0.033277477	0.015572076	NCF1/VEGFA/IL1B	3
BP	GO:0052126	movement in host environment	6/210	177/18800	0.014681955	0.033308901	0.015586781	CDK1/ICAM1/LDLR/EGFR/CAV1/CXCL8	6
BP	GO:0032612	interleukin-1 production	5/210	129/18800	0.014961318	0.033888383	0.015857947	GSTP1/STAT3/CASP8/HSPB1/IFNG	5
BP	GO:0032652	regulation of interleukin-1 production	5/210	129/18800	0.014961318	0.033888383	0.015857947	GSTP1/STAT3/CASP8/HSPB1/IFNG	5
BP	GO:0035601	protein deacylation	5/210	129/18800	0.014961318	0.033888383	0.015857947	MAPK8/SREBF1/VEGFA/TP53/IFNG	5

BP	GO:0002366	leukocyte activation involved in immune response	8/210	285/18800	0.015029982	0.033968265	0.015895328	IL4/HMOX1/ICAM1/STAT3/IL6R/TP53/IFNG/CD40LG	8
BP	GO:0002295	T-helper cell lineage commitment	2/210	17/18800	0.015124556	0.033968265	0.015895328	STAT3/IL6R	2
BP	GO:0016114	terpenoid biosynthetic process	2/210	17/18800	0.015124556	0.033968265	0.015895328	CYP1A1/AKR1C3	2
BP	GO:0016202	regulation of striated muscle tissue development	2/210	17/18800	0.015124556	0.033968265	0.015895328	GJA1/ERBB3	2
BP	GO:0020027	hemoglobin metabolic process	2/210	17/18800	0.015124556	0.033968265	0.015895328	CAT/HIF1A	2
BP	GO:0046827	positive regulation of protein export from nucleus	2/210	17/18800	0.015124556	0.033968265	0.015895328	GSK3B/IL1B	2
BP	GO:0048875	chemical homeostasis within a tissue	2/210	17/18800	0.015124556	0.033968265	0.015895328	KDR/VEGFA	2
BP	GO:00550	fatty	2/210	17/18	0.0151	0.033968	0.0158953	GOT1/DGAT2	2

	89	acid homeostasis		800	24556	265	28		
BP	GO:0061450	trophoblast cell migration	2/210	17/18800	0.015124556	0.033968265	0.015895328	VEGFA/GJA1	2
BP	GO:0070230	positive regulation of lymphocyte apoptotic process	2/210	17/18800	0.015124556	0.033968265	0.015895328	BAX/TP53	2
BP	GO:0090036	regulation of protein kinase C signaling	2/210	17/18800	0.015124556	0.033968265	0.015895328	ADRA1A/VEGFA	2
BP	GO:1900034	regulation of cellular response to heat	2/210	17/18800	0.015124556	0.033968265	0.015895328	GSK3B/HSF1	2
BP	GO:1903358	regulation of Golgi organization	2/210	17/18800	0.015124556	0.033968265	0.015895328	MAPK3/MAPK1	2
BP	GO:1904355	positive regulation of telomere capping	2/210	17/18800	0.015124556	0.033968265	0.015895328	MAPK3/MAPK1	2
BP	GO:2000811	negative regulation of anoikis	2/210	17/18800	0.015124556	0.033968265	0.015895328	BCL2/CAV1	2
BP	GO:2001267	regulation of cysteine-type	2/210	17/18800	0.015124556	0.033968265	0.015895328	BAX/MMP9	2

		endopeptidase activity involved in apoptotic signaling pathway							
BP	GO:0050886	endocrine process	4/210	85/18800	0.015163153	0.03401897	0.015919055	PPARG/GJA1/IL1B/NOS3	4
BP	GO:2000243	positive regulation of reproductive process	4/210	85/18800	0.015163153	0.03401897	0.015919055	AR/PLB1/VEGFA/PLAT	4
BP	GO:0045088	regulation of innate immune response	7/210	231/18800	0.015323861	0.034361372	0.016079281	HSP90AB1/PPARG/NCF1/IKBKB/CASP8/NFE2L2/IRF1	7
BP	GO:0035303	regulation of dephosphorylation	5/210	130/18800	0.015422763	0.034564894	0.016174518	HSP90AB1/GSK3B/CHRM5/IKBKB/IFNG	5
BP	GO:0043409	negative regulation of MAPK cascade	6/210	179/18800	0.01543913	0.034565094	0.016174612	PPARG/GSTP1/HMGCR/CAV1/MYC/IL1B	6
BP	GO:0050777	negative regulation of immune response	6/210	179/18800	0.01543913	0.034565094	0.016174612	PPARG/MAPK14/IL4/HMOX1/AHR/COL3A1	6
BP	GO:0045601	regulation of endothelial cell different	3/210	47/18800	0.015533478	0.034721411	0.0162477 6	IKBKB/VEGFA/IL1B	3

		iation							
BP	GO:1990089	response to nerve growth factor	3/210	47/18800	0.015533478	0.034721411	0.01624776	AKT1/HSPA5/E2F1	3
BP	GO:2000144	positive regulation of DNA-templated transcription, initiation	3/210	47/18800	0.015533478	0.034721411	0.01624776	ESR1/JUN/TP53	3
BP	GO:0046928	regulation of neurotransmitter secretion	4/210	86/18800	0.015767412	0.035225774	0.016483775	ADRA1A/GSK3B/DRD1/PRKCB	4
BP	GO:0006022	aminoglycan metabolic process	5/210	131/18800	0.015893629	0.035489087	0.016606991	LYG1/TNFAIP6/HAS2/EGF/IL1B	5
BP	GO:0002263	cell activation involved in immune response	8/210	289/18800	0.016210972	0.036178664	0.016929676	IL4/HMOX1/ICAM1/STAT3/IL6R/TP53/IFNG/CD40LG	8
BP	GO:0000723	telomere maintenance	5/210	132/18800	0.016373993	0.036468794	0.017065441	HSP90AB1/MAPK3/MAPK1/MYC/PARP1	5
BP	GO:0055006	cardiac cell development	4/210	87/18800	0.016386619	0.036468794	0.017065441	ADRA1A/CDK1/PPARA/VEGFA	4
BP	GO:0055017	cardiac muscle tissue growth	4/210	87/18800	0.016386619	0.036468794	0.017065441	ADRA1A/MAPK14/CDK1/PPARA	4
BP	GO:00076	visual	3/210	48/18	0.0164	0.036468	0.0170654	DRD1/HMGCR/HI	3

	32	behavior		800	3543	794	41	F1A	
BP	GO:0010662	regulation of striated muscle cell apoptotic process	3/210	48/18800	0.01643543	0.036468794	0.017065441	TP53/NFE2L2/HSF1	3
BP	GO:0044818	mitotic G2/M transition checkpoint	3/210	48/18800	0.01643543	0.036468794	0.017065441	CHEK1/CDK1/CDKN1A	3
BP	GO:0046460	neutral lipid biosynthetic process	3/210	48/18800	0.01643543	0.036468794	0.017065441	LDLR/SREBF1/DGAT2	3
BP	GO:0046463	acylglycerol biosynthetic process	3/210	48/18800	0.01643543	0.036468794	0.017065441	LDLR/SREBF1/DGAT2	3
BP	GO:0050798	activated T cell proliferation	3/210	48/18800	0.01643543	0.036468794	0.017065441	CASP3/IL2RA/IGF2	3
BP	GO:0097720	calcineurin-mediated signaling	3/210	48/18800	0.01643543	0.036468794	0.017065441	GSK3B/PPP3CA/ERBB3	3
BP	GO:1902003	regulation of amyloid-beta formation	3/210	48/18800	0.01643543	0.036468794	0.017065441	RELA/CASP3/IFNG	3
BP	GO:0007219	Notch signaling pathway	6/210	182/18800	0.016625048	0.03683181	0.017235313	AKT1/GOT1/STAT3/NFKBIA/NOS3/IL2RA	6

BP	GO:0032640	tumor necrosis factor production	6/210	182/18800	0.016625048	0.03683181	0.017235313	IL4/GSTP1/STAT3/HSPB1/IFNG/IL1A	6
BP	GO:0032680	regulation of tumor necrosis factor production	6/210	182/18800	0.016625048	0.03683181	0.017235313	IL4/GSTP1/STAT3/HSPB1/IFNG/IL1A	6
BP	GO:0098732	macromolecule deacylation	5/210	133/18800	0.016863928	0.036997097	0.017312659	MAPK8/SREBF1/VEGFA/TP53/IFNG	5
BP	GO:0001696	gastric acid secretion	2/210	18/18800	0.016891304	0.036997097	0.017312659	CHRM5/PTGER3	2
BP	GO:0002076	osteoblast development	2/210	18/18800	0.016891304	0.036997097	0.017312659	ACHE/RUNX2	2
BP	GO:0006309	apoptotic DNA fragmentation	2/210	18/18800	0.016891304	0.036997097	0.017312659	BAX/HSF1	2
BP	GO:0010612	regulation of cardiac muscle adaptation	2/210	18/18800	0.016891304	0.036997097	0.017312659	PPARG/PPP3CA	2
BP	GO:0010759	positive regulation of macrophage chemotaxis	2/210	18/18800	0.016891304	0.036997097	0.017312659	MAPK3/MAPK1	2
BP	GO:0030299	intestinal choleste	2/210	18/18800	0.016891304	0.036997097	0.017312659	LDLR/SOAT2	2

		rol absorption							
BP	GO:0034433	steroid esterification	2/210	18/18800	0.016891304	0.036997097	0.017312659	SOAT2/SOAT1	2
BP	GO:0034434	sterol esterification	2/210	18/18800	0.016891304	0.036997097	0.017312659	SOAT2/SOAT1	2
BP	GO:0034435	cholesterol esterification	2/210	18/18800	0.016891304	0.036997097	0.017312659	SOAT2/SOAT1	2
BP	GO:0036499	PERK-mediated unfolded protein response	2/210	18/18800	0.016891304	0.036997097	0.017312659	HSPA5/NFE2L2	2
BP	GO:0043217	myelin maintenance	2/210	18/18800	0.016891304	0.036997097	0.017312659	AKT1/SOD1	2
BP	GO:0044320	cellular response to leptin stimulus	2/210	18/18800	0.016891304	0.036997097	0.017312659	CCNA2/STAT3	2
BP	GO:0045023	G0 to G1 transition	2/210	18/18800	0.016891304	0.036997097	0.017312659	CHEK1/MYC	2
BP	GO:0045722	positive regulation of gluconeogenesis	2/210	18/18800	0.016891304	0.036997097	0.017312659	PPARA/DGAT2	2
BP	GO:0060391	positive regulation of SMAD protein signal transduction	2/210	18/18800	0.016891304	0.036997097	0.017312659	PPARG/PARP1	2
BP	GO:00604	trachea	2/210	18/18	0.0168	0.036997	0.0173126	MAPK3/MAPK1	2

	38	development		800	91304	097	59		
BP	GO:0060644	mammary gland epithelial cell differentiation	2/210	18/18800	0.016891304	0.036997097	0.017312659	AKT1/HIF1A	2
BP	GO:0071318	cellular response to ATP	2/210	18/18800	0.016891304	0.036997097	0.017312659	PTGS2/SOD1	2
BP	GO:0150079	negative regulation of neuroinflammatory response	2/210	18/18800	0.016891304	0.036997097	0.017312659	IL4/LDLR	2
BP	GO:1903242	regulation of cardiac muscle hypertrophy in response to stress	2/210	18/18800	0.016891304	0.036997097	0.017312659	PPARG/PPP3CA	2
BP	GO:2000641	regulation of early endosome to late endosome transport	2/210	18/18800	0.016891304	0.036997097	0.017312659	MAPK3/MAPK1	2
BP	GO:0000422	autophagy of mitochondrion	4/210	88/18800	0.017020881	0.037185022	0.017400597	SREBF1/TP53/HIF1A/HK2	4
BP	GO:0014031	mesenchymal cell development	4/210	88/18800	0.017020881	0.037185022	0.017400597	BCL2/MAPK3/MAPK1/HIF1A	4

BP	GO:0046849	bone remodeling	4/210	88/18800	0.017020881	0.037185022	0.017400597	ADRB2/PRKCA/GJA1/SPP1	4
BP	GO:0061726	mitochondrion disassembly	4/210	88/18800	0.017020881	0.037185022	0.017400597	SREBF1/TP53/HIF1A/HK2	4
BP	GO:1903510	mucopolysaccharide metabolic process	4/210	88/18800	0.017020881	0.037185022	0.017400597	TNFAIP6/HAS2/EGF/IL1B	4
BP	GO:0055001	muscle cell development	6/210	183/18800	0.01703394	0.037194419	0.017404995	ADRA1A/BCL2/CDK1/PPP3CA/PPARA/VEGFA	6
BP	GO:1903052	positive regulation of proteolysis involved in cellular protein catabolic process	5/210	134/18800	0.017363507	0.037687422	0.017635694	GSK3B/AKT1/EGF/CAV1/NFE2L2	5
BP	GO:0002762	negative regulation of myeloid leukocyte differentiation	3/210	49/18800	0.017366208	0.037687422	0.017635694	IL4/TNFAIP6/MYC	3
BP	GO:0008089	anterograde axonal transport	3/210	49/18800	0.017366208	0.037687422	0.017635694	SOD1/HSPB1/MAP2	3
BP	GO:0010656	negative regulation of	3/210	49/18800	0.017366208	0.037687422	0.017635694	HMOX1/NFE2L2/HSF1	3

		muscle cell apoptotic process							
BP	GO:0010659	cardiac muscle cell apoptotic process	3/210	49/18800	0.017366208	0.037687422	0.017635694	TP53/NFE2L2/HSF1	3
BP	GO:0042304	regulation of fatty acid biosynthetic process	3/210	49/18800	0.017366208	0.037687422	0.017635694	PTGS2/EIF6/IL1B	3
BP	GO:0045058	T cell selection	3/210	49/18800	0.017366208	0.037687422	0.017635694	BCL2/STAT3/IL6R	3
BP	GO:0046850	regulation of bone remodeling	3/210	49/18800	0.017366208	0.037687422	0.017635694	PRKCA/GJA1/SPP1	3
BP	GO:0048546	digestive tract morphogenesis	3/210	49/18800	0.017366208	0.037687422	0.017635694	BCL2/EGFR/HIF1A	3
BP	GO:0060260	regulation of transcription initiation from RNA polymerase II promoter	3/210	49/18800	0.017366208	0.037687422	0.017635694	ESR1/CDK4/TP53	3
BP	GO:0060986	endocrine hormone secretio	3/210	49/18800	0.017366208	0.037687422	0.017635694	PPARG/GJA1/IL1B	3

		n							
BP	GO:1903793	positive regulation of anion transport	3/210	49/18800	0.017366208	0.037687422	0.017635694	ABAT/IL1B/IL1A	3
BP	GO:0009260	ribonucleotide biosynthetic process	6/210	184/18800	0.017449712	0.037847339	0.017710526	IL4/ATP5F1B/PPARA/ACACA/MYC/PARP1	6
BP	GO:0140014	mitotic nuclear division	8/210	293/18800	0.0174 5772	0.037847339	0.017710526	CHEK1/RB1/EGF/IL1B/CCNB1/IL1A/CHEK2/IGF2	8
BP	GO:0001656	metanephros development	4/210	89/18800	0.0176703	0.038288655	0.017917039	AKR1B1/BCL2/STAT1/MYC	4
BP	GO:0002697	regulation of immune effector process	9/210	353/18800	0.018036285	0.039061754	0.018278808	NOS2/NCF1/IL4/HMOX1/ICAM1/AHR/IL1B/IFNG/CD40LG	9
BP	GO:0038084	vascular endothelial growth factor signaling pathway	3/210	50/18800	0.018325881	0.039607646	0.018534256	KDR/VEGFA/HSPB1	3
BP	GO:0061178	regulation of insulin secretion involved in cellular response to glucose stimulus	3/210	50/18800	0.018325881	0.039607646	0.018534256	PPARD/BAD/HIF1A	3
BP	GO:19030	regulatio	3/210	50/18	0.0183	0.039607	0.0185342	DPP4/HAS2/RB1	3

	53	n of extracellular matrix organization		800	25881	646	56		
BP	GO:1903747	regulation of establishment of protein localization to mitochondrion	3/210	50/18800	0.018325881	0.039607646	0.018534256	MAPK8/SREBF1/NPEPPS	3
BP	GO:0002028	regulation of sodium ion transport	4/210	90/18800	0.018334973	0.039607646	0.018534256	SCN5A/ADRB2/AKT1/NOS3	4
BP	GO:0030534	adult behavior	5/210	136/18800	0.018391885	0.039710389	0.018582334	OPRM1/DRD1/OPRD1/PPARA/ABAT	5
BP	GO:0009060	aerobic respiration	6/210	187/18800	0.018738809	0.040017166	0.018725889	ATP5F1B/CDK1/CAT/HIF1A/MYC/CCNB1	6
BP	GO:0071706	tumor necrosis factor superfamily cytokine production	6/210	187/18800	0.018738809	0.040017166	0.018725889	IL4/GSTP1/STAT3/HSPB1/IFNG/IL1A	6
BP	GO:1903555	regulation of tumor necrosis factor superfamily cytokine production	6/210	187/18800	0.018738809	0.040017166	0.018725889	IL4/GSTP1/STAT3/HSPB1/IFNG/IL1A	6

BP	GO:0002523	leukocyte migration involved in inflammatory response	2/210	19/18800	0.018741263	0.040017166	0.018725889	SELE/ALOX5	2
BP	GO:0006071	glycerol metabolic process	2/210	19/18800	0.018741263	0.040017166	0.018725889	GOT1/DGAT2	2
BP	GO:0006837	serotonin transport	2/210	19/18800	0.018741263	0.040017166	0.018725889	MAOB/SLC6A4	2
BP	GO:0032332	positive regulation of chondrocyte differentiation	2/210	19/18800	0.018741263	0.040017166	0.018725889	POR/RUNX2	2
BP	GO:0032930	positive regulation of superoxide anion generation	2/210	19/18800	0.018741263	0.040017166	0.018725889	GSTP1/SOD1	2
BP	GO:0035743	CD4-positive, alpha-beta T cell cytokine production	2/210	19/18800	0.018741263	0.040017166	0.018725889	IL4/IL1B	2
BP	GO:0042574	retinal metabolic process	2/210	19/18800	0.018741263	0.040017166	0.018725889	CYP1B1/AKR1C3	2
BP	GO:0043031	negative regulation of	2/210	19/18800	0.018741263	0.040017166	0.018725889	IL4/LDLR	2

		macrophage activation							
BP	GO:0045091	regulation of single stranded viral RNA replication via double stranded DNA intermediate	2/210	19/18800	0.018741263	0.040017166	0.018725889	CXCL8/TOP2A	2
BP	GO:0048245	eosinophil chemotaxis	2/210	19/18800	0.018741263	0.040017166	0.018725889	IL4/CCL2	2
BP	GO:0048643	positive regulation of skeletal muscle tissue development	2/210	19/18800	0.018741263	0.040017166	0.018725889	BCL2/IGF2	2
BP	GO:0060004	reflex	2/210	19/18800	0.018741263	0.040017166	0.018725889	ADRA1A/GJA1	2
BP	GO:0060546	negative regulation of necroptotic process	2/210	19/18800	0.018741263	0.040017166	0.018725889	CASP8/CAV1	2
BP	GO:0061298	retina vasculature development in camera-type eye	2/210	19/18800	0.018741263	0.040017166	0.018725889	CYP1B1/HIF1A	2

BP	GO:0070989	oxidative demethylation	2/210	19/18800	0.018741263	0.040017166	0.018725889	CYP3A4/CYP1A2	2
BP	GO:0072074	kidney mesenchyme development	2/210	19/18800	0.018741263	0.040017166	0.018725889	STAT1/MYC	2
BP	GO:0086014	atrial cardiac muscle cell action potential	2/210	19/18800	0.018741263	0.040017166	0.018725889	SCN5A/GJA1	2
BP	GO:0086026	atrial cardiac muscle cell to AV node cell signaling	2/210	19/18800	0.018741263	0.040017166	0.018725889	SCN5A/GJA1	2
BP	GO:0086066	atrial cardiac muscle cell to AV node cell communication	2/210	19/18800	0.018741263	0.040017166	0.018725889	SCN5A/GJA1	2
BP	GO:0010639	negative regulation of organelle organization	9/210	356/18800	0.018939633	0.040420413	0.018914588	PPARG/CHEK1/AKT1/BCL2L1/TP53/CCNB1/TOP2A/PARP1/MAP2	9
BP	GO:0045638	negative regulation of myeloid	4/210	91/18800	0.019014996	0.040499844	0.018951757	IL4/TNFAIP6/NFKBIA/MYC	4

		cell differentiation							
BP	GO:0070664	negative regulation of leukocyte proliferation	4/210	91/18800	0.019014996	0.040499844	0.018951757	CASP3/GSTP1/ERBB2/IL2RA	4
BP	GO:0072080	nephron tubule development	4/210	91/18800	0.019014996	0.040499844	0.018951757	BCL2/STAT1/VEGFA/MYC	4
BP	GO:0090277	positive regulation of peptide hormone secretion	4/210	91/18800	0.019014996	0.040499844	0.018951757	PPARD/BAD/ABAT/HIF1A	4
BP	GO:0007157	heterophilic cell-cell adhesion via plasma membrane cell adhesion molecules	3/210	51/18800	0.019314504	0.041034867	0.019202119	ICAM1/SELE/VCAM1	3
BP	GO:0010658	striated muscle cell apoptotic process	3/210	51/18800	0.019314504	0.041034867	0.019202119	TP53/NFE2L2/HSF1	3
BP	GO:0051932	synaptic transmission, GABAergic	3/210	51/18800	0.019314504	0.041034867	0.019202119	ADRA1A/GABRA1/CA2	3
BP	GO:00606	regulatio	3/210	51/18	0.0193	0.041034	0.0192021	AR/ESR1/VEGFA	3

	88	n of morphogenesis of a branching structure		800	14504	867	19		
BP	GO:2000179	positive regulation of neural precursor cell proliferation	3/210	51/18800	0.019314504	0.041034867	0.019202119	VEGFA/EGF/HIF1A	3
BP	GO:0008277	regulation of G protein-coupled receptor signaling pathway	5/210	138/18800	0.019459684	0.041322638	0.01933678	ADRB2/OPRM1/PRKCA/CXCL8/ACP3	5
BP	GO:0032436	positive regulation of proteasomal ubiquitin-dependent protein catabolic process	4/210	92/18800	0.019710459	0.041834243	0.019576184	GSK3B/AKT1/CAV1/NFE2L2	4
BP	GO:0055076	transition metal ion homeostasis	5/210	139/18800	0.020008534	0.042424485	0.019852386	HMOX1/SOD1/HIF1A/MYC/IFNG	5
BP	GO:1903900	regulation of viral life cycle	5/210	139/18800	0.020008534	0.042424485	0.019852386	BCL2/SLPI/PPARA/CXCL8/TOP2A	5

BP	GO:0061136	regulation of proteasomal protein catabolic process	6/210	190/18800	0.020091621	0.042579397	0.019924876	HSP90AB1/GSK3B/CDK2/AKT1/CAV1/NFE2L2	6
BP	GO:0038093	Fc receptor signaling pathway	3/210	52/18800	0.020332119	0.043024668	0.020133239	MAPK10/IKBKB/MAPK8	3
BP	GO:0048013	ephrin receptor signaling pathway	3/210	52/18800	0.020332119	0.043024668	0.020133239	MMP2/MMP9/RASA1	3
BP	GO:2000677	regulation of transcription regulatory region DNA binding	3/210	52/18800	0.020332119	0.043024668	0.020133239	RB1/IFNG/PARP1	3
BP	GO:0002793	positive regulation of peptide secretion	4/210	93/18800	0.020421448	0.043170677	0.020201564	PPARD/BAD/ABAT/HIF1A	4
BP	GO:0060993	kidney morphogenesis	4/210	93/18800	0.020421448	0.043170677	0.020201564	BCL2/STAT1/VEGFA/MYC	4
BP	GO:0046390	ribose phosphate biosynthetic process	6/210	191/18800	0.020556942	0.043249043	0.020238234	IL4/ATP5F1B/PPARA/ACACA/MYC/PARP1	6
BP	GO:0072073	kidney epitheliu	5/210	140/18800	0.020567438	0.043249043	0.020238234	BCL2/STAT1/CAT/VEGFA/MYC	5

		m develop ment							
BP	GO:0006525	arginine metabolic process	2/210	20/18800	0.02067237	0.043249043	0.020238234	NOS2/NOS3	2
BP	GO:0009110	vitamin biosynthetic process	2/210	20/18800	0.02067237	0.043249043	0.020238234	CYP3A4/IFNG	2
BP	GO:0010288	response to lead ion	2/210	20/18800	0.02067237	0.043249043	0.020238234	PTGS2/CAT	2
BP	GO:0010523	negative regulation of calcium ion transport into cytosol	2/210	20/18800	0.02067237	0.043249043	0.020238234	BCL2/GSTM2	2
BP	GO:0031998	regulation of fatty acid beta-oxidation	2/210	20/18800	0.02067237	0.043249043	0.020238234	AKT1/PPARA	2
BP	GO:0032793	positive regulation of CREB transcription factor activity	2/210	20/18800	0.02067237	0.043249043	0.020238234	OPRD1/VEGFA	2
BP	GO:0035902	response to immobilization stress	2/210	20/18800	0.02067237	0.043249043	0.020238234	CYP1A1/FOS	2
BP	GO:0036498	IRE1-mediated unfolded	2/210	20/18800	0.02067237	0.043249043	0.020238234	BAX/HSPA5	2

		protein response							
BP	GO:0039692	single stranded viral RNA replication via double stranded DNA intermediate	2/210	20/18800	0.02067237	0.043249043	0.020238234	CXCL8/TOP2A	2
BP	GO:0043373	CD4-positive, alpha-beta T cell lineage commitment	2/210	20/18800	0.02067237	0.043249043	0.020238234	STAT3/IL6R	2
BP	GO:0060716	labyrinthine layer blood vessel development	2/210	20/18800	0.02067237	0.043249043	0.020238234	AKT1/MAPK1	2
BP	GO:0062099	negative regulation of programmed necrotic cell death	2/210	20/18800	0.02067237	0.043249043	0.020238234	CASP8/CAV1	2
BP	GO:0070584	mitochondrion morphogenesis	2/210	20/18800	0.02067237	0.043249043	0.020238234	BAX/BCL2L1	2
BP	GO:0090026	positive regulation of monocyte	2/210	20/18800	0.02067237	0.043249043	0.020238234	SERPINE1/CXCL10	2

		chemotaxis							
BP	GO:0090201	negative regulation of release of cytochrome c from mitochondria	2/210	20/18800	0.0206 7237	0.043249 043	0.0202382 34	AKT1/BCL2L1	2
BP	GO:1902074	response to salt	2/210	20/18800	0.0206 7237	0.043249 043	0.0202382 34	HSPA5/HSF1	2
BP	GO:1903209	positive regulation of oxidative stress-induced cell death	2/210	20/18800	0.0206 7237	0.043249 043	0.0202382 34	SOD1/MMP3	2
BP	GO:1904292	regulation of ERAD pathway	2/210	20/18800	0.0206 7237	0.043249 043	0.0202382 34	CAV1/NFE2L2	2
BP	GO:2000010	positive regulation of protein localization to cell surface	2/210	20/18800	0.0206 7237	0.043249 043	0.0202382 34	HSP90AB1/AKT1	2
BP	GO:0046328	regulation of JNK cascade	5/210	141/18800	0.0211 3646	0.044157 205	0.0206632 06	NCF1/GSTP1/EGFR/IL1B/IL1A	5
BP	GO:0001657	ureteric bud development	4/210	94/18800	0.0211 48047	0.044157 205	0.0206632 06	BCL2/CAT/VEGFA/MYC	4
BP	GO:00455	positive	4/210	94/18	0.0211	0.044157	0.0206632	IL4/BAD/IL2RA/I	4

	82	regulation of T cell differentiation		800	48047	205	06	FNG	
BP	GO:0061326	renal tubule development	4/210	94/18800	0.021148047	0.044157205	0.020663206	BCL2/STAT1/VEGFA/MYC	4
BP	GO:0010043	response to zinc ion	3/210	53/18800	0.021378753	0.044616985	0.020878358	LTA4H/VCAM1/PARP1	3
BP	GO:0032147	activation of protein kinase activity	5/210	142/18800	0.021715662	0.045297847	0.021196965	ADRB2/IL4/VEGFA/EGF/IL6R	5
BP	GO:0032088	negative regulation of NF-kappaB transcription factor activity	4/210	95/18800	0.021890334	0.04559502	0.021336026	CYP1B1/CAT/NFKBIA/CHUK	4
BP	GO:0072163	mesonephric epithelium development	4/210	95/18800	0.021890334	0.04559502	0.021336026	BCL2/CAT/VEGFA/MYC	4
BP	GO:0072164	mesonephric tubule development	4/210	95/18800	0.021890334	0.04559502	0.021336026	BCL2/CAT/VEGFA/MYC	4
BP	GO:0006814	sodium ion transport	7/210	249/18800	0.022111218	0.04603252	0.021540753	SCN5A/ADRB2/SLC6A2/SLC6A4/SLC6A3/AKT1/NOS3	7
BP	GO:0061351	neural precursor cell	5/210	143/18800	0.022305104	0.046413413	0.0217189 9	SLC6A4/VEGFA/EGF/TP53/HIF1A	5

		proliferation							
BP	GO:0045744	negative regulation of G protein-coupled receptor signaling pathway	3/210	54/18800	0.022454422	0.046655543	0.021832294	ADRB2/OPRM1/CXCL8	3
BP	GO:0071320	cellular response to cAMP	3/210	54/18800	0.022454422	0.046655543	0.021832294	CYP1B1/AHR/HSPA5	3
BP	GO:0072348	sulfur compound transport	3/210	54/18800	0.022454422	0.046655543	0.021832294	ABCC1/GJA1/ABCG2	3
BP	GO:0060419	heart growth	4/210	96/18800	0.022648384	0.046672968	0.021840448	ADRA1A/MAPK14/CDK1/PPARA	4
BP	GO:0000083	regulation of transcription involved in G1/S transition of mitotic cell cycle	2/210	21/18800	0.022682601	0.046672968	0.021840448	RB1/E2F1	2
BP	GO:0003091	renal water homeostasis	2/210	21/18800	0.022682601	0.046672968	0.021840448	AKR1B1/HAS2	2
BP	GO:0006590	thyroid hormone generation	2/210	21/18800	0.022682601	0.046672968	0.021840448	DIO1/DUOX2	2
BP	GO:0015669	gas transport	2/210	21/18800	0.022682601	0.046672968	0.021840448	CA2/MYC	2
BP	GO:0019370	leukotriene	2/210	21/18800	0.022682601	0.046672968	0.021840448	LTA4H/ALOX5	2

		biosynthetic process							
BP	GO:0019400	alditol metabolic process	2/210	21/18800	0.022682601	0.046672968	0.021840448	GOT1/DGAT2	2
BP	GO:0030220	platelet formation	2/210	21/18800	0.022682601	0.046672968	0.021840448	CASP3/CASP9	2
BP	GO:0032098	regulation of appetite	2/210	21/18800	0.022682601	0.046672968	0.021840448	OPRM1/PPARA	2
BP	GO:0032305	positive regulation of icosanoid secretion	2/210	21/18800	0.022682601	0.046672968	0.021840448	IL1B/IL1A	2
BP	GO:0032727	positive regulation of interferon-alpha production	2/210	21/18800	0.022682601	0.046672968	0.021840448	STAT1/CHUK	2
BP	GO:0033762	response to glucagon	2/210	21/18800	0.022682601	0.046672968	0.021840448	CCNA2/SREBF1	2
BP	GO:0035809	regulation of urine volume	2/210	21/18800	0.022682601	0.046672968	0.021840448	AKR1B1/HAS2	2
BP	GO:0044241	lipid digestion	2/210	21/18800	0.022682601	0.046672968	0.021840448	LDLR/SOAT2	2
BP	GO:0046716	muscle cell cellular homeostasis	2/210	21/18800	0.022682601	0.046672968	0.021840448	SOD1/HIF1A	2
BP	GO:00600	uterus	2/210	21/18	0.0226	0.046672	0.0218404	ESR1/CYP19A1	2

	65	development		800	82601	968	48		
BP	GO:0071605	monocyte chemotactic protein-1 production	2/210	21/18800	0.022682601	0.046672968	0.021840448	GSTP1/IL1B	2
BP	GO:0071637	regulation of monocyte chemotactic protein-1 production	2/210	21/18800	0.022682601	0.046672968	0.021840448	GSTP1/IL1B	2
BP	GO:0098856	intestinal lipid absorption	2/210	21/18800	0.022682601	0.046672968	0.021840448	LDLR/SOAT2	2
BP	GO:2000178	negative regulation of neural precursor cell proliferation	2/210	21/18800	0.022682601	0.046672968	0.021840448	SLC6A4/TP53	2
BP	GO:0048285	organelle fission	11/210	493/18800	0.023071081	0.047449336	0.022203747	PPARG/KDR/CHEK1/RB1/EGF/IL1B/CCNB1/IL1A/TOP2A/CHEK2/IGF2	11
BP	GO:0008593	regulation of Notch signaling pathway	4/210	97/18800	0.023422269	0.048148295	0.022530822	AKT1/STAT3/NFKBIA/NOS3	4
BP	GO:0007612	learning	5/210	145/18800	0.023514943	0.048292052	0.022598093	PTGS2/DRD1/HMGCR/FOS/HIF1A	5

BP	GO:0072006	nephron development	5/210	145/18800	0.023514943	0.048292052	0.022598093	BCL2/STAT1/VEGFA/IL6R/MYC	5
BP	GO:0001954	positive regulation of cell-matrix adhesion	3/210	55/18800	0.023559128	0.048312706	0.022607758	KDR/GSK3B/VEGFA	3
BP	GO:0045620	negative regulation of lymphocyte differentiation	3/210	55/18800	0.023559128	0.048312706	0.022607758	IL4/ERBB2/IRF1	3
BP	GO:1902991	regulation of amyloid precursor protein catabolic process	3/210	55/18800	0.023559128	0.048312706	0.022607758	RELA/CASP3/IFNG	3
BP	GO:0046718	viral entry into host cell	5/210	146/18800	0.024135454	0.049470692	0.023149633	CDK1/ICAM1/LDLR/EGFR/CAV1	5
BP	GO:0016575	histone deacetylation	4/210	98/18800	0.024212057	0.049508236	0.023167202	MAPK8/SREBF1/VEGFA/TP53	4
BP	GO:0021549	cerebellum development	4/210	98/18800	0.024212057	0.049508236	0.023167202	SCN5A/ABAT/TP53/HSPA5	4
BP	GO:0044728	DNA methylation or demethylation	4/210	98/18800	0.024212057	0.049508236	0.023167202	CYP1A1/FOS/MYC/PARP1	4
BP	GO:0061337	cardiac conduction	4/210	98/18800	0.024212057	0.049508236	0.023167202	SCN5A/KCNH2/CAV1/GJA1	4
BP	GO:19040	negative	4/210	98/18	0.0242	0.049508	0.0231672	GSTM2/KCNH2/	4

	63	regulation of cation transmembrane transport		800	12057	236	02	MMP9/CAV1	
BP	GO:0003007	heart morphogenesis	7/210	254/18800	0.024316128	0.049697111	0.023255585	JUN/HAS2/VEGFA/TP53/HIF1A/GJA1/NOS3	7
BP	GO:0034205	amyloid-beta formation	3/210	56/18800	0.024692861	0.05007046	0.023430292	RELA/CASP3/IFNG	3
BP	GO:0048016	inositol phosphate-mediated signaling	3/210	56/18800	0.024692861	0.05007046	0.023430292	GSK3B/PPP3CA/ERBB3	3
BP	GO:0001919	regulation of receptor recycling	2/210	22/18800	0.024769964	0.05007046	0.023430292	ACHE/LDLR	2
BP	GO:0007530	sex determination	2/210	22/18800	0.024769964	0.05007046	0.023430292	AR/INSRR	2
BP	GO:0007620	copulation	2/210	22/18800	0.024769964	0.05007046	0.023430292	SLC6A4/ABAT	2
BP	GO:0010042	response to manganese ion	2/210	22/18800	0.024769964	0.05007046	0.023430292	PTGS2/HSPA5	2
BP	GO:0010226	response to lithium ion	2/210	22/18800	0.024769964	0.05007046	0.023430292	PTGS2/NFE2L2	2
BP	GO:0015874	norepinephrine transport	2/210	22/18800	0.024769964	0.05007046	0.023430292	SLC6A2/SLC6A3	2
BP	GO:0016137	glycoside metabolic	2/210	22/18800	0.024769964	0.05007046	0.023430292	AKR1B1/AKR1C3	2

		process							
BP	GO:0032303	regulation of icosanoid secretion	2/210	22/18800	0.024769964	0.05007046	0.023430292	IL1B/IL1A	2
BP	GO:0032891	negative regulation of organic acid transport	2/210	22/18800	0.024769964	0.05007046	0.023430292	AKT1/ABAT	2
BP	GO:0032928	regulation of superoxide anion generation	2/210	22/18800	0.024769964	0.05007046	0.023430292	GSTP1/SOD1	2
BP	GO:0035357	peroxisome proliferator activated receptor signaling pathway	2/210	22/18800	0.024769964	0.05007046	0.023430292	RXRA/PPARG	2
BP	GO:0035584	calcium-mediated signaling using intracellular calcium source	2/210	22/18800	0.024769964	0.05007046	0.023430292	KDR/VCAM1	2
BP	GO:0036344	platelet morphogenesis	2/210	22/18800	0.024769964	0.05007046	0.023430292	CASP3/CASP9	2
BP	GO:0044346	fibroblast apoptoti	2/210	22/18800	0.024769964	0.05007046	0.023430292	TP53/MYC	2

		c process							
BP	GO:0046885	regulation of hormone biosynthetic process	2/210	22/18800	0.024769964	0.05007046	0.023430292	POR/HIF1A	2
BP	GO:0048634	regulation of muscle organ development	2/210	22/18800	0.024769964	0.05007046	0.023430292	GJA1/ERBB3	2
BP	GO:0051000	positive regulation of nitric-oxide synthase activity	2/210	22/18800	0.024769964	0.05007046	0.023430292	AKT1/HIF1A	2
BP	GO:0090335	regulation of brown fat cell differentiation	2/210	22/18800	0.024769964	0.05007046	0.023430292	PTGS2/MAPK14	2
BP	GO:0140467	integrated stress response signaling	2/210	22/18800	0.024769964	0.05007046	0.023430292	HSPA5/NFE2L2	2
BP	GO:1902644	tertiary alcohol metabolic process	2/210	22/18800	0.024769964	0.05007046	0.023430292	AKR1B1/AKR1C3	2
BP	GO:1903589	positive regulation of blood vessel endothelial cell	2/210	22/18800	0.024769964	0.05007046	0.023430292	HMOX1/VEGFA	2

		proliferation involved in sprouting angiogenesis							
BP	GO:0001823	mesonephros development	4/210	99/18800	0.025017812	0.050499357	0.023630993	BCL2/CAT/VEGFA/MYC	4
BP	GO:0050764	regulation of phagocytosis	4/210	99/18800	0.025017812	0.050499357	0.023630993	SOD1/IL1B/CCL2/IFNG	4
BP	GO:0051588	regulation of neurotransmitter transport	4/210	99/18800	0.025017812	0.050499357	0.023630993	ADRA1A/GSK3B/DRD1/PRKCB	4
BP	GO:0050864	regulation of B cell activation	6/210	200/18800	0.025077399	0.050595589	0.023676025	IL4/BCL2/CASP3/AHR/BAD/CDKN1A	6
BP	GO:0051053	negative regulation of DNA metabolic process	5/210	148/18800	0.025407934	0.051238127	0.023976698	CHEK1/CDKN1A/TP53/PARP1/HSF1	5
BP	GO:0014013	regulation of gliogenesis	4/210	100/18800	0.025839593	0.051992772	0.024329831	LDLR/MYC/IL1B/E2F1	4
BP	GO:0001658	branching involved in ureteric bud morpho	3/210	57/18800	0.0258556	0.051992772	0.024329831	BCL2/VEGFA/MYC	3

		genesis							
BP	GO:0010803	regulation of tumor necrosis factor-mediated signaling pathway	3/210	57/18800	0.0258556	0.051992772	0.024329831	IKBKB/GSTP1/CASP8	3
BP	GO:0070228	regulation of lymphocyte apoptotic process	3/210	57/18800	0.0258556	0.051992772	0.024329831	BAX/TP53/HIF1A	3
BP	GO:0098900	regulation of action potential	3/210	57/18800	0.0258556	0.051992772	0.024329831	SCN5A/ADRA1A/CAV1	3
BP	GO:2001258	negative regulation of cation channel activity	3/210	57/18800	0.0258556	0.051992772	0.024329831	GSTM2/MMP9/CAV1	3
BP	GO:0010640	regulation of platelet-derived growth factor receptor signaling pathway	2/210	23/18800	0.026932503	0.053903083	0.025223754	F7/F3	2
BP	GO:0014821	phasic smooth muscle contraction	2/210	23/18800	0.026932503	0.053903083	0.025223754	DRD1/PTGER3	2
BP	GO:0021854	hypothalamus	2/210	23/18800	0.026932503	0.053903083	0.025223754	BAX/MYC	2

		development							
BP	GO:0032042	mitochondrial DNA metabolic process	2/210	23/18800	0.026932503	0.053903083	0.025223754	TP53/PARP1	2
BP	GO:0034695	response to prostaglandin E	2/210	23/18800	0.026932503	0.053903083	0.025223754	AKT1/ACACA	2
BP	GO:0045663	positive regulation of myoblast differentiation	2/210	23/18800	0.026932503	0.053903083	0.025223754	MAPK14/IGFBP3	2
BP	GO:0045723	positive regulation of fatty acid biosynthetic process	2/210	23/18800	0.026932503	0.053903083	0.025223754	PTGS2/IL1B	2
BP	GO:0072677	eosinophil migration	2/210	23/18800	0.026932503	0.053903083	0.025223754	IL4/CCL2	2
BP	GO:1990840	response to lectin	2/210	23/18800	0.026932503	0.053903083	0.025223754	IKBKB/MYC	2
BP	GO:1990858	cellular response to lectin	2/210	23/18800	0.026932503	0.053903083	0.025223754	IKBKB/MYC	2
BP	GO:0002090	regulation of receptor internalization	3/210	58/18800	0.027047313	0.054005612	0.025271732	SELE/VEGFA/EGF	3
BP	GO:0010559	regulation of glycopro	3/210	58/18800	0.027047313	0.054005612	0.025271732	BCL2/SOAT1/BACE2	3

		tein biosynthetic process							
BP	GO:0032481	positive regulation of type I interferon production	3/210	58/18800	0.027047313	0.054005612	0.025271732	STAT1/CHUK/IRF1	3
BP	GO:0033619	membrane protein proteolysis	3/210	58/18800	0.027047313	0.054005612	0.025271732	BACE2/IL1B/IFNG	3
BP	GO:0086065	cell communication involved in cardiac conduction	3/210	58/18800	0.027047313	0.054005612	0.025271732	SCN5A/CAV1/GJA1	3
BP	GO:0035710	CD4-positive, alpha-beta T cell activation	4/210	102/18800	0.027531458	0.054946476	0.025712006	IL4/STAT3/IL6R/IFNG	4
BP	GO:0000768	syncytium formation by plasma membrane fusion	3/210	59/18800	0.028267954	0.056284107	0.026337946	MAPK14/IL4/CXCL10	3
BP	GO:0035065	regulation of histone acetylation	3/210	59/18800	0.028267954	0.056284107	0.026337946	CHEK1/MAPK3/IL1B	3

BP	GO:0043666	regulation of phosphoprotein phosphatase activity	3/210	59/18800	0.028267954	0.056284107	0.026337946	HSP90AB1/GSK3B/IKBKB	3
BP	GO:0050891	multicellular organismal water homeostasis	3/210	59/18800	0.028267954	0.056284107	0.026337946	AKR1B1/HAS2/CLDN4	3
BP	GO:0140253	cell-cell fusion	3/210	59/18800	0.028267954	0.056284107	0.026337946	MAPK14/IL4/CXCL10	3
BP	GO:0032760	positive regulation of tumor necrosis factor production	4/210	103/18800	0.028401643	0.05649732	0.026437718	STAT3/HSPB1/IFNG/IL1A	4
BP	GO:0070252	actin-mediated cell contraction	4/210	103/18800	0.028401643	0.05649732	0.026437718	SCN5A/KCNH2/CAV1/GJA1	4
BP	GO:0001964	startle response	2/210	24/18800	0.029168295	0.05764437	0.026974476	DRD1/SLC6A3	2
BP	GO:0002052	positive regulation of neuroblast proliferation	2/210	24/18800	0.029168295	0.05764437	0.026974476	VEGFA/HIF1A	2
BP	GO:0035458	cellular response to interferon-beta	2/210	24/18800	0.029168295	0.05764437	0.026974476	STAT1/IRF1	2
BP	GO:00420	protein	2/210	24/18	0.0291	0.057644	0.0269744	HSPA5/HSPB1	2

	26	refolding		800	68295	37	76		
BP	GO:0043567	regulation of insulin-like growth factor receptor signaling pathway	2/210	24/18800	0.029168295	0.05764437	0.026974476	AR/IGFBP3	2
BP	GO:0045672	positive regulation of osteoclast differentiation	2/210	24/18800	0.029168295	0.05764437	0.026974476	FOS/IFNG	2
BP	GO:0048011	neurotrophin TRK receptor signaling pathway	2/210	24/18800	0.029168295	0.05764437	0.026974476	CASP3/RAF1	2
BP	GO:0051043	regulation of membrane protein ectodomain proteolysis	2/210	24/18800	0.029168295	0.05764437	0.026974476	IL1B/IFNG	2
BP	GO:0051156	glucose 6-phosphate metabolic process	2/210	24/18800	0.029168295	0.05764437	0.026974476	TP53/HK2	2
BP	GO:0060143	positive regulation of	2/210	24/18800	0.029168295	0.05764437	0.026974476	MAPK14/IL4	2

		syncytium formation by plasma membrane fusion							
BP	GO:0060547	negative regulation of necrotic cell death	2/210	24/18800	0.029168295	0.05764437	0.026974476	CASP8/CAV1	2
BP	GO:0071676	negative regulation of mononuclear cell migration	2/210	24/18800	0.029168295	0.05764437	0.026974476	AKT1/CCL2	2
BP	GO:0120255	olefinic compound biosynthetic process	2/210	24/18800	0.029168295	0.05764437	0.026974476	AKR1C3/CYP19A1	2
BP	GO:1901028	regulation of mitochondrial outer membrane permeabilization involved in apoptotic signaling pathway	2/210	24/18800	0.029168295	0.05764437	0.026974476	GSK3B/MAPK8	2

BP	GO:0034766	negative regulation of ion transmembrane transport	4/210	104/18800	0.029288059	0.057854133	0.027072634	GSTM2/KCNH2/MMP9/CAV1	4
BP	GO:0097553	calcium ion transmembrane import into cytosol	5/210	154/18800	0.029480679	0.058199021	0.027234023	DRD1/BAX/GSTM2/CXCL11/CXCL10	5
BP	GO:0051205	protein insertion into membrane	3/210	60/18800	0.029517469	0.058199021	0.027234023	BAX/MAPK8/EGFR	3
BP	GO:1903078	positive regulation of protein localization to plasma membrane	3/210	60/18800	0.029517469	0.058199021	0.027234023	AKT1/EGFR/IFNG	3
BP	GO:1903670	regulation of sprouting angiogenesis	3/210	60/18800	0.029517469	0.058199021	0.027234023	ALOX5/VEGFA/E2F2	3
BP	GO:0007405	neuroblast proliferation	3/210	61/18800	0.030795793	0.060663142	0.0283871	VEGFA/TP53/HIF1A	3
BP	GO:1904356	regulation of telomere maintenance via telomere lengthen	3/210	61/18800	0.030795793	0.060663142	0.0283871	MAPK3/MAPK1/PARP1	3

		ing							
BP	GO:1903169	regulation of calcium ion transmembrane transport	5/210	156/18800	0.030924605	0.06088864	0.028492621	DRD1/BAX/GSTM2/CXCL11/CXCL10	5
BP	GO:0032414	positive regulation of ion transmembrane transporter activity	4/210	106/18800	0.031109745	0.061168096	0.028623391	ADRB2/GSTM2/CCL2/IFNG	4
BP	GO:0042116	macrophage activation	4/210	106/18800	0.031109745	0.061168096	0.028623391	JUN/IL4/LDLR/IFNG	4
BP	GO:0060840	artery development	4/210	106/18800	0.031109745	0.061168096	0.028623391	LDLR/APOB/VEGFA/COL3A1	4
BP	GO:0002768	immune response-regulating cell surface receptor signaling pathway	8/210	328/18800	0.031444621	0.061516908	0.028786616	MAPK10/RELA/IKBKB/BCL2/BAX/MAPK8/MAPK1/PRKCB	8
BP	GO:0032469	endoplasmic reticulum calcium ion homeostasis	2/210	25/18800	0.031475451	0.061516908	0.028786616	BCL2/BAX	2
BP	GO:0033081	regulation of T cell different	2/210	25/18800	0.031475451	0.061516908	0.028786616	SOD1/ERBB2	2

		iation in thymus							
BP	GO:0045662	negative regulation of myoblast differentiation	2/210	25/18800	0.031475451	0.061516908	0.028786616	PPARD/CXCL10	2
BP	GO:0060343	trabecula formation	2/210	25/18800	0.031475451	0.061516908	0.028786616	MMP2/COL1A1	2
BP	GO:0070423	nucleotide-binding oligomerization domain containing signaling pathway	2/210	25/18800	0.031475451	0.061516908	0.028786616	RELA/NFKBIA	2
BP	GO:0071459	protein localization to chromosome, centromeric region	2/210	25/18800	0.031475451	0.061516908	0.028786616	CDK1/RB1	2
BP	GO:0086064	cell communication by electrical coupling involved in cardiac conduction	2/210	25/18800	0.031475451	0.061516908	0.028786616	CAV1/GJA1	2

BP	GO:1905048	regulation of metallopeptidase activity	2/210	25/18800	0.031475451	0.061516908	0.028786616	STAT3/CLDN4	2
BP	GO:1905523	positive regulation of macrophage migration	2/210	25/18800	0.031475451	0.061516908	0.028786616	MAPK3/MAPK1	2
BP	GO:1905563	negative regulation of vascular endothelial cell proliferation	2/210	25/18800	0.031475451	0.061516908	0.028786616	PPARG/CCL2	2
BP	GO:1905564	positive regulation of vascular endothelial cell proliferation	2/210	25/18800	0.031475451	0.061516908	0.028786616	STAT3/IGF2	2
BP	GO:2000193	positive regulation of fatty acid transport	2/210	25/18800	0.031475451	0.061516908	0.028786616	IL1B/IL1A	2
BP	GO:0016079	synaptic vesicle exocytosis	4/210	107/18800	0.032045088	0.062543883	0.029267186	ADRA1A/GSK3B/DRD1/PRKCB	4
BP	GO:0045621	positive regulation of lymphocyte different	4/210	107/18800	0.032045088	0.062543883	0.029267186	IL4/BAD/IL2RA/IFNG	4

		iation							
BP	GO:1903557	positive regulation of tumor necrosis factor superfamily cytokine production	4/210	107/18800	0.032045088	0.062543883	0.029267186	STAT3/HSPB1/IFNG/IL1A	4
BP	GO:0010972	negative regulation of G2/M transition of mitotic cell cycle	3/210	62/18800	0.032102851	0.062599085	0.029293018	CHEK1/CDK1/CDKN1A	3
BP	GO:2000736	regulation of stem cell differentiation	3/210	62/18800	0.032102851	0.062599085	0.029293018	GSK3B/STAT3/NFE2L2	3
BP	GO:0051962	positive regulation of nervous system development	7/210	270/18800	0.032377768	0.063106187	0.029530314	OPRM1/VEGFA/HIF1A/MYC/IL1B/IFNG/E2F1	7
BP	GO:0061387	regulation of extent of cell growth	4/210	108/18800	0.032996807	0.06428323	0.030081107	GSK3B/VEGFA/SPP1/MAP2	4
BP	GO:0032613	interleukin-10 production	3/210	63/18800	0.033438557	0.064965027	0.030400152	IL4/STAT3/CD40LG	3
BP	GO:00326	regulatio	3/210	63/18	0.0334	0.064965	0.0304001	IL4/STAT3/CD40	3

	53	n of interleukin-10 production		800	38557	027	52	LG	
BP	GO:0045453	bone resorption	3/210	63/18800	0.033438557	0.064965027	0.030400152	ADRB2/PRKCA/SPP1	3
BP	GO:0060675	ureteric bud morphogenesis	3/210	63/18800	0.033438557	0.064965027	0.030400152	BCL2/VEGFA/MYC	3
BP	GO:2000242	negative regulation of reproductive process	3/210	63/18800	0.033438557	0.064965027	0.030400152	GJA1/PLAT/IL1A	3
BP	GO:2000401	regulation of lymphocyte migration	3/210	63/18800	0.033438557	0.064965027	0.030400152	AKT1/CCL2/CXCL10	3
BP	GO:0000737	DNA catabolic process, endonucleolytic	2/210	26/18800	0.033852113	0.065290611	0.030552507	BAX/HSF1	2
BP	GO:0002726	positive regulation of T cell cytokine production	2/210	26/18800	0.033852113	0.065290611	0.030552507	IL4/IL1B	2
BP	GO:0032515	negative regulation of phosphoprotein phosphatase	2/210	26/18800	0.033852113	0.065290611	0.030552507	GSK3B/IKBKB	2

		activity							
BP	GO:0035872	nucleotide-binding domain, leucine rich repeat containing receptor signaling pathway	2/210	26/18800	0.033852113	0.065290611	0.030552507	RELA/NFKBIA	2
BP	GO:0045745	positive regulation of G protein-coupled receptor signaling pathway	2/210	26/18800	0.033852113	0.065290611	0.030552507	PRKCA/ACP3	2
BP	GO:0046339	diacylglycerol metabolic process	2/210	26/18800	0.033852113	0.065290611	0.030552507	PLB1/DGAT2	2
BP	GO:0048641	regulation of skeletal muscle tissue development	2/210	26/18800	0.033852113	0.065290611	0.030552507	BCL2/IGF2	2
BP	GO:0050857	positive regulation of antigen receptor-mediated signaling	2/210	26/18800	0.033852113	0.065290611	0.030552507	RELA/PRKCB	2

		pathway							
BP	GO:0050995	negative regulation of lipid catabolic process	2/210	26/18800	0.033852113	0.065290611	0.030552507	AKT1/IL1B	2
BP	GO:0051204	protein insertion into mitochondrial membrane	2/210	26/18800	0.033852113	0.065290611	0.030552507	BAX/MAPK8	2
BP	GO:0051894	positive regulation of focal adhesion assembly	2/210	26/18800	0.033852113	0.065290611	0.030552507	KDR/VEGFA	2
BP	GO:0070102	interleukin-6-mediated signaling pathway	2/210	26/18800	0.033852113	0.065290611	0.030552507	STAT3/IL6R	2
BP	GO:0071378	cellular response to growth hormone stimulus	2/210	26/18800	0.033852113	0.065290611	0.030552507	STAT3/MYC	2
BP	GO:0071549	cellular response to dexamethasone stimulus	2/210	26/18800	0.033852113	0.065290611	0.030552507	CASP9/PLAT	2
BP	GO:1901623	regulation of lymphocyte	2/210	26/18800	0.033852113	0.065290611	0.030552507	CCL2/CXCL10	2

		chemotaxis							
BP	GO:1904353	regulation of telomere capping	2/210	26/18800	0.033852113	0.065290611	0.030552507	MAPK3/MAPK1	2
BP	GO:0090263	positive regulation of canonical Wnt signaling pathway	4/210	109/18800	0.03396493	0.065478465	0.030640413	EGFR/EGF/CAV1/COL1A1	4
BP	GO:0006473	protein acetylation	6/210	216/18800	0.0346627	0.066793325	0.031255697	NCOA1/GSK3B/CHEK1/MAPK3/POR/IL1B	6
BP	GO:0051250	negative regulation of lymphocyte activation	5/210	161/18800	0.034726402	0.066850999	0.031282685	IL4/CASP3/ERBB2/IL2RA/IRF1	5
BP	GO:0014015	positive regulation of gliogenesis	3/210	64/18800	0.034802816	0.066850999	0.031282685	MYC/IL1B/E2F1	3
BP	GO:0048247	lymphocyte chemotaxis	3/210	64/18800	0.034802816	0.066850999	0.031282685	CCL2/CXCL11/CXCL10	3
BP	GO:0048857	neural nucleus development	3/210	64/18800	0.034802816	0.066850999	0.031282685	MAOB/BCL2/HSPA5	3
BP	GO:0072171	mesonephric tubule morphogenesis	3/210	64/18800	0.034802816	0.066850999	0.031282685	BCL2/VEGFA/MYC	3
BP	GO:1902750	negative regulatio	3/210	64/18800	0.034802816	0.066850999	0.031282685	CHEK1/CDK1/CDKN1A	3

		n of cell cycle G2/M phase transition							
BP	GO:1903018	regulation of glycoprotein metabolic process	3/210	64/18800	0.034802816	0.066850999	0.031282685	BCL2/SOAT1/BACE2	3
BP	GO:0043266	regulation of potassium ion transport	4/210	110/18800	0.034949481	0.067102372	0.031400314	DRD1/KCNH2/CAV1/NOS3	4
BP	GO:0001837	epithelial to mesenchymal transition	5/210	162/18800	0.035519958	0.06810531	0.031869635	GSK3B/HAS2/HIF1A/IL1B/COL1A1	5
BP	GO:0030509	BMP signaling pathway	5/210	162/18800	0.035519958	0.06810531	0.031869635	PPARG/KDR/TNFAIP6/MAPK3/RUNX2	5
BP	GO:0032200	telomere organization	5/210	162/18800	0.035519958	0.06810531	0.031869635	HSP90AB1/MAPK3/MAPK1/MYC/PARP1	5
BP	GO:0072009	nephron epithelium development	4/210	111/18800	0.03595048	0.068899679	0.032241358	BCL2/STAT1/VEGFA/MYC	4
BP	GO:0007588	excretion	3/210	65/18800	0.036195524	0.068971389	0.032274914	ADRA1A/HMOX1/ABCG2	3
BP	GO:0031343	positive regulation of cell killing	3/210	65/18800	0.036195524	0.068971389	0.032274914	NOS2/MAPK8/IFNG	3
BP	GO:0042093	T-helper cell	3/210	65/18800	0.036195524	0.068971389	0.032274914	IL4/STAT3/IL6R	3

		differentiation							
BP	GO:0048645	animal organ formation	3/210	65/18800	0.036195524	0.068971389	0.032274914	AR/MAPK3/MAPK1	3
BP	GO:2000272	negative regulation of signaling receptor activity	3/210	65/18800	0.036195524	0.068971389	0.032274914	PPARG/ESR2/PPARA	3
BP	GO:0002227	innate immune response in mucosa	2/210	27/18800	0.036296457	0.068971389	0.032274914	NOS2/IL4	2
BP	GO:0018904	ether metabolic process	2/210	27/18800	0.036296457	0.068971389	0.032274914	ALOX5/FASN	2
BP	GO:0032607	interferon-alpha production	2/210	27/18800	0.036296457	0.068971389	0.032274914	STAT1/CHUK	2
BP	GO:0032647	regulation of interferon-alpha production	2/210	27/18800	0.036296457	0.068971389	0.032274914	STAT1/CHUK	2
BP	GO:0034505	tooth mineralization	2/210	27/18800	0.036296457	0.068971389	0.032274914	PPARA/COL1A1	2
BP	GO:0035640	exploration behavior	2/210	27/18800	0.036296457	0.068971389	0.032274914	DPP4/ABAT	2
BP	GO:0036037	CD8-positive, alpha-beta T cell activation	2/210	27/18800	0.036296457	0.068971389	0.032274914	BCL2/IRF1	2

BP	GO:0042403	thyroid hormone metabolic process	2/210	27/18800	0.036296457	0.068971389	0.032274914	DIO1/DUOX2	2
BP	GO:0060669	embryonic placenta morphogenesis	2/210	27/18800	0.036296457	0.068971389	0.032274914	NCOA1/IGF2	2
BP	GO:0071467	cellular response to pH	2/210	27/18800	0.036296457	0.068971389	0.032274914	INSRR/GJA1	2
BP	GO:0071624	positive regulation of granulocyte chemotaxis	2/210	27/18800	0.036296457	0.068971389	0.032274914	IL4/CXCL8	2
BP	GO:1900017	positive regulation of cytokine production involved in inflammatory response	2/210	27/18800	0.036296457	0.068971389	0.032274914	STAT3/HIF1A	2
BP	GO:1902932	positive regulation of alcohol biosynthetic process	2/210	27/18800	0.036296457	0.068971389	0.032274914	SREBF1/POR	2
BP	GO:2000108	positive regulation of leukocyte apoptoti	2/210	27/18800	0.036296457	0.068971389	0.032274914	BAX/TP53	2

		c process							
BP	GO:0030307	positive regulation of cell growth	5/210	163/18800	0.036324654	0.0689941	0.032285542	AKT1/BCL2/EGFR/VEGFA/ERBB2	5
BP	GO:0002456	T cell mediated immunity	4/210	112/18800	0.036967945	0.0701532	0.032827938	IL4/ICAM1/AHR/IL1B	4
BP	GO:0009063	cellular amino acid catabolic process	4/210	112/18800	0.036967945	0.0701532	0.032827938	NOS2/GOT1/ABAT/NOS3	4
BP	GO:0010812	negative regulation of cell-substrate adhesion	3/210	66/18800	0.037616569	0.071256722	0.033344327	SERPINE1/COL1A1/RASA1	3
BP	GO:0030104	water homeostasis	3/210	66/18800	0.037616569	0.071256722	0.033344327	AKR1B1/HAS2/CLDN4	3
BP	GO:0071677	positive regulation of mononuclear cell migration	3/210	66/18800	0.037616569	0.071256722	0.033344327	IL4/SERPINE1/CXCL10	3
BP	GO:1900076	regulation of cellular response to insulin stimulus	3/210	66/18800	0.037616569	0.071256722	0.033344327	IL1B/PRKCB/IGF2	3
BP	GO:0051656	establishment of organell	9/210	404/18800	0.038196197	0.072322448	0.03384303	IL4/HMOX1/SLC2A4/EIF6/HIF1A/GJA1/CCNB1/IFNG	9

		e localization						/MAP2	
BP	GO:0001958	endochondral ossification	2/210	28/18800	0.03880669	0.072861191	0.034095133	COL1A1/RUNX2	2
BP	GO:0010800	positive regulation of peptidyl-threonine phosphorylation	2/210	28/18800	0.03880669	0.072861191	0.034095133	MAPK1/EGF	2
BP	GO:0010818	T cell chemotaxis	2/210	28/18800	0.03880669	0.072861191	0.034095133	CXCL11/CXCL10	2
BP	GO:0022011	myelination in peripheral nervous system	2/210	28/18800	0.03880669	0.072861191	0.034095133	AKT1/SOD1	2
BP	GO:0030431	sleep	2/210	28/18800	0.03880669	0.072861191	0.034095133	ADRB1/FOS	2
BP	GO:0031342	negative regulation of cell killing	2/210	28/18800	0.03880669	0.072861191	0.034095133	HSP90AB1/IL4	2
BP	GO:0032292	peripheral nervous system axon ensheathment	2/210	28/18800	0.03880669	0.072861191	0.034095133	AKT1/SOD1	2
BP	GO:0036075	replacement ossification	2/210	28/18800	0.03880669	0.072861191	0.034095133	COL1A1/RUNX2	2
BP	GO:0042104	positive regulation of	2/210	28/18800	0.03880669	0.072861191	0.034095133	IL2RA/IGF2	2

		activated T cell proliferation							
BP	GO:0042402	cellular biogenic amine catabolic process	2/210	28/18800	0.03880669	0.072861191	0.034095133	MAOB/SLC6A3	2
BP	GO:0042983	amyloid precursor protein biosynthetic process	2/210	28/18800	0.03880669	0.072861191	0.034095133	SOAT1/BACE2	2
BP	GO:0042984	regulation of amyloid precursor protein biosynthetic process	2/210	28/18800	0.03880669	0.072861191	0.034095133	SOAT1/BACE2	2
BP	GO:0044030	regulation of DNA methylation	2/210	28/18800	0.03880669	0.072861191	0.034095133	MYC/PARP1	2
BP	GO:0050996	positive regulation of lipid catabolic process	2/210	28/18800	0.03880669	0.072861191	0.034095133	PPARA/IL1B	2
BP	GO:0060544	regulation of necroptotic process	2/210	28/18800	0.03880669	0.072861191	0.034095133	CASP8/CAV1	2
BP	GO:0070723	response to choleste	2/210	28/18800	0.03880669	0.072861191	0.034095133	F7/CES1	2

		rol							
BP	GO:0090025	regulation of monocyte chemotaxis	2/210	28/18800	0.03880669	0.072861191	0.034095133	SERPINE1/CXCL10	2
BP	GO:0090330	regulation of platelet aggregation	2/210	28/18800	0.03880669	0.072861191	0.034095133	ABAT/PRKCA	2
BP	GO:1903671	negative regulation of sprouting angiogenesis	2/210	28/18800	0.03880669	0.072861191	0.034095133	ALOX5/E2F2	2
BP	GO:0042303	molting cycle	4/210	114/18800	0.039052322	0.073218261	0.034262223	PTGS2/RELA/BCL2/EGFR	4
BP	GO:0042633	hair cycle	4/210	114/18800	0.039052322	0.073218261	0.034262223	PTGS2/RELA/BCL2/EGFR	4
BP	GO:0055021	regulation of cardiac muscle tissue growth	3/210	67/18800	0.03906583	0.073218261	0.034262223	MAPK14/CDK1/PPARA	3
BP	GO:1904377	positive regulation of protein localization to cell periphery	3/210	67/18800	0.03906583	0.073218261	0.034262223	AKT1/EGFR/IFNG	3
BP	GO:0042130	negative regulation of T cell proliferation	3/210	68/18800	0.040543176	0.075853246	0.035495254	CASP3/ERBB2/IL2RA	3

BP	GO:0042987	amyloid precursor protein catabolic process	3/210	68/18800	0.040543176	0.075853246	0.035495254	RELA/CASP3/IFNG	3
BP	GO:0046503	glycerolipid catabolic process	3/210	68/18800	0.040543176	0.075853246	0.035495254	LDLR/APOB/PLB1	3
BP	GO:2000756	regulation of peptidyl-lysine acetylation	3/210	68/18800	0.040543176	0.075853246	0.035495254	CHEK1/MAPK3/IL1B	3
BP	GO:1901800	positive regulation of proteasomal protein catabolic process	4/210	116/18800	0.041202683	0.077053191	0.036056764	GSK3B/AKT1/CAV1/NFE2L2	4
BP	GO:0003298	physiological muscle hypertrophy	2/210	29/18800	0.04138105	0.077081281	0.036069909	ADRA1A/PPARA	2
BP	GO:0003301	physiological cardiac muscle hypertrophy	2/210	29/18800	0.04138105	0.077081281	0.036069909	ADRA1A/PPARA	2
BP	GO:0010667	negative regulation of cardiac muscle cell apoptoti	2/210	29/18800	0.04138105	0.077081281	0.036069909	NFE2L2/HSF1	2

		c process							
BP	GO:0032367	intracellular cholesterol transport	2/210	29/18800	0.04138105	0.077081281	0.036069909	LDLR/CES1	2
BP	GO:0048265	response to pain	2/210	29/18800	0.04138105	0.077081281	0.036069909	SLC6A2/AKT1	2
BP	GO:0051481	negative regulation of cytosolic calcium ion concentration	2/210	29/18800	0.04138105	0.077081281	0.036069909	BCL2/GSTM2	2
BP	GO:0061049	cell growth involved in cardiac muscle cell development	2/210	29/18800	0.04138105	0.077081281	0.036069909	ADRA1A/PPARA	2
BP	GO:1900117	regulation of execution phase of apoptosis	2/210	29/18800	0.04138105	0.077081281	0.036069909	BCL2L1/TP53	2
BP	GO:1903203	regulation of oxidative stress-induced neuron death	2/210	29/18800	0.04138105	0.077081281	0.036069909	HIF1A/PARP1	2
BP	GO:0006520	cellular amino	7/210	285/18800	0.041405726	0.077093432	0.036075595	DPEP1/NOS2/DIO1/GOT1/ABAT/O	7

		acid metabolic process						DC1/NOS3	
BP	GO:0007004	telomere maintenance via telomerase	3/210	69/18800	0.042048469	0.078187325	0.036587478	HSP90AB1/MAPK3/MAPK1	3
BP	GO:0034394	protein localization to cell surface	3/210	69/18800	0.042048469	0.078187325	0.036587478	HSP90AB1/AKT1/EGF	3
BP	GO:0070527	platelet aggregation	3/210	69/18800	0.042048469	0.078187325	0.036587478	ABAT/PRKCA/HSPB1	3
BP	GO:0061564	axon development	10/210	479/18800	0.043251456	0.080389031	0.037617759	HSP90AB1/GSK3B/JUN/BCL2/CASP3/VEGFA/MMP2/ERBB2/SPP1/MAP2	10
BP	GO:0051966	regulation of synaptic transmission, glutamatergic	3/210	70/18800	0.043581565	0.080967152	0.037888288	PTGS2/DRD1/CCL2	3
BP	GO:0001945	lymph vessel development	2/210	30/18800	0.044017807	0.081244513	0.038018079	KDR/VEGFA	2
BP	GO:0007176	regulation of epidermal growth factor-activated receptor activity	2/210	30/18800	0.044017807	0.081244513	0.038018079	NCF1/EGF	2
BP	GO:0009310	amine catabolic	2/210	30/18800	0.044017807	0.081244513	0.038018079	MAOB/SLC6A3	2

		c process							
BP	GO:0045671	negative regulation of osteoclast differentiation	2/210	30/18800	0.044017807	0.081244513	0.038018079	IL4/TNFAIP6	2
BP	GO:0045939	negative regulation of steroid metabolic process	2/210	30/18800	0.044017807	0.081244513	0.038018079	SOD1/UGT1A1	2
BP	GO:0051123	RNA polymerase II preinitiation complex assembly	2/210	30/18800	0.044017807	0.081244513	0.038018079	ESR1/TP53	2
BP	GO:0060045	positive regulation of cardiac muscle cell proliferation	2/210	30/18800	0.044017807	0.081244513	0.038018079	MAPK14/CDK1	2
BP	GO:0060218	hematopoietic stem cell differentiation	2/210	30/18800	0.044017807	0.081244513	0.038018079	TP53/NFE2L2	2
BP	GO:0060325	face morphogenesis	2/210	30/18800	0.044017807	0.081244513	0.038018079	MMP2/COL1A1	2
BP	GO:0070168	negative regulation of	2/210	30/18800	0.044017807	0.081244513	0.038018079	HIF1A/NOS3	2

		biomineral tissue development							
BP	GO:0086011	membrane repolarization during action potential	2/210	30/18800	0.044017807	0.081244513	0.038018079	KCNH2/CAV1	2
BP	GO:0090140	regulation of mitochondrial fission	2/210	30/18800	0.044017807	0.081244513	0.038018079	PPARG/KDR	2
BP	GO:0090151	establishment of protein localization to mitochondrial membrane	2/210	30/18800	0.044017807	0.081244513	0.038018079	BAX/MAPK8	2
BP	GO:1900101	regulation of endoplasmic reticulum unfolded protein response	2/210	30/18800	0.044017807	0.081244513	0.038018079	BAX/HSPA5	2
BP	GO:1902230	negative regulation of intrinsic apoptotic signaling pathway	2/210	30/18800	0.044017807	0.081244513	0.038018079	BCL2/BCL2L1	2

		in response to DNA damage							
BP	GO:0018393	internal peptidyl-lysine acetylation	5/210	173/18800	0.044990035	0.08293085	0.038807194	NCOA1/CHEK1/MAPK3/POR/IL1B	5
BP	GO:0071772	response to BMP	5/210	173/18800	0.044990035	0.08293085	0.038807194	PPARG/KDR/TNFAIP6/MAPK3/RUNX2	5
BP	GO:0071773	cellular response to BMP stimulus	5/210	173/18800	0.044990035	0.08293085	0.038807194	PPARG/KDR/TNFAIP6/MAPK3/RUNX2	5
BP	GO:0090100	positive regulation of transmembrane receptor protein serine/threonine kinase signaling pathway	4/210	120/18800	0.045701376	0.084205529	0.039403675	HSP90AB1/PPARG/KDR/PARP1	4
BP	GO:0002274	myeloid leukocyte activation	6/210	232/18800	0.046346851	0.085141409	0.039841617	JUN/IL4/HMOX1/LDLR/CXCL8/IFNG	6
BP	GO:0000002	mitochondrial genome maintenance	2/210	31/18800	0.046715259	0.085141409	0.039841617	TP53/PARP1	2
BP	GO:0002230	positive regulation of defense response	2/210	31/18800	0.046715259	0.085141409	0.039841617	IL4/STAT1	2

		to virus by host							
BP	GO:0003299	muscle hypertrophy in response to stress	2/210	31/18800	0.046715259	0.085141409	0.039841617	PPARG/PPP3CA	2
BP	GO:0010664	negative regulation of striated muscle cell apoptotic process	2/210	31/18800	0.046715259	0.085141409	0.039841617	NFE2L2/HSF1	2
BP	GO:0014044	Schwann cell development	2/210	31/18800	0.046715259	0.085141409	0.039841617	AKT1/SOD1	2
BP	GO:0014887	cardiac muscle adaptation	2/210	31/18800	0.046715259	0.085141409	0.039841617	PPARG/PPP3CA	2
BP	GO:0014898	cardiac muscle hypertrophy in response to stress	2/210	31/18800	0.046715259	0.085141409	0.039841617	PPARG/PPP3CA	2
BP	GO:0019433	triglyceride catabolic process	2/210	31/18800	0.046715259	0.085141409	0.039841617	APOB/PLB1	2
BP	GO:0033137	negative regulation of peptidyl-serine phosphorylation	2/210	31/18800	0.046715259	0.085141409	0.039841617	BAX/CAV1	2
BP	GO:0034390	smooth muscle	2/210	31/18800	0.046715259	0.085141409	0.039841617	PPARG/IFNG	2

		cell apoptotic process							
BP	GO:0034391	regulation of smooth muscle cell apoptotic process	2/210	31/18800	0.046715259	0.085141409	0.039841617	PPARG/IFNG	2
BP	GO:0035066	positive regulation of histone acetylation	2/210	31/18800	0.046715259	0.085141409	0.039841617	MAPK3/IL1B	2
BP	GO:0046825	regulation of protein export from nucleus	2/210	31/18800	0.046715259	0.085141409	0.039841617	GSK3B/IL1B	2
BP	GO:0060351	cartilage development involved in endochondral bone morphogenesis	2/210	31/18800	0.046715259	0.085141409	0.039841617	POR/COL1A1	2
BP	GO:0060390	regulation of SMAD protein signal transduction	2/210	31/18800	0.046715259	0.085141409	0.039841617	PPARG/PARP1	2
BP	GO:0061036	positive regulation of	2/210	31/18800	0.046715259	0.085141409	0.039841617	POR/RUNX2	2

		cartilage development							
BP	GO:0070528	protein kinase C signaling	2/210	31/18800	0.046715259	0.085141409	0.039841617	ADRA1A/VEGFA	2
BP	GO:0070633	transepithelial transport	2/210	31/18800	0.046715259	0.085141409	0.039841617	ABCC1/ABCG2	2
BP	GO:0071295	cellular response to vitamin	2/210	31/18800	0.046715259	0.085141409	0.039841617	RXRA/COL1A1	2
BP	GO:0110150	negative regulation of biomineralization	2/210	31/18800	0.046715259	0.085141409	0.039841617	HIF1A/NOS3	2
BP	GO:0150117	positive regulation of cell-substrate junction organization	2/210	31/18800	0.046715259	0.085141409	0.039841617	KDR/VEGFA	2
BP	GO:1900027	regulation of ruffle assembly	2/210	31/18800	0.046715259	0.085141409	0.039841617	ICAM1/CAV1	2
BP	GO:0014855	striated muscle cell proliferation	3/210	72/18800	0.046730545	0.085141409	0.039841617	MAPK14/PPARD/CDK1	3
BP	GO:0030330	DNA damage response, signal transduction by	3/210	72/18800	0.046730545	0.085141409	0.039841617	CDKN1A/TP53/CHEK2	3

		p53 class mediator							
BP	GO:0072078	nephron tubule morphogenesis	3/210	72/18800	0.046730545	0.085141409	0.039841617	BCL2/VEGFA/MYC	3
BP	GO:0006475	internal protein amino acid acetylation	5/210	175/18800	0.046859105	0.085280733	0.039906813	NCOA1/CHEK1/MAPK3/POR/IL1B	5
BP	GO:0048675	axon extension	4/210	121/18800	0.046867255	0.085280733	0.039906813	HSP90AB1/GSK3B/VEGFA/MAP2	4
BP	GO:0055007	cardiac muscle cell differentiation	4/210	121/18800	0.046867255	0.085280733	0.039906813	ADRA1A/CDK1/PPARA/VEGFA	4
BP	GO:0002221	pattern recognition receptor signaling pathway	5/210	176/18800	0.047810738	0.086960259	0.040692741	ESR1/RELA/NFKBIA/CAV1/IRF1	5
BP	GO:0045055	regulated exocytosis	6/210	234/18800	0.047960037	0.087157158	0.04078488	ADRA1A/GSK3B/DRD1/IL4/HMOX1/PRKCB	6
BP	GO:0060560	developmental growth involved in morphogenesis	6/210	234/18800	0.047960037	0.087157158	0.04078488	HSP90AB1/ESR1/GSK3B/VEGFA/SPP1/MAP2	6
BP	GO:0016311	dephosphorylation	9/210	422/18800	0.048005288	0.087202078	0.0408059	HSP90AB1/GSK3B/CHRM5/IKBKB/BCL2/PPP3CA/IFNG/ACP3/PON1	9
BP	GO:00108	positive	4/210	122/1	0.0480	0.087207	0.0408086	KDR/GSK3B/HAS	4

	11	regulation of cell-substrate adhesion		8800	49589	951	48	2/VEGFA	
BP	GO:0048593	camera-type eye morphogenesis	4/210	122/18800	0.048049589	0.087207951	0.040808648	KDR/BAX/VEGFA/HIF1A	4
BP	GO:0002224	toll-like receptor signaling pathway	4/210	123/18800	0.049248362	0.089255083	0.041766596	ESR1/NFKBIA/CAV1/IRF1	4
BP	GO:0003180	aortic valve morphogenesis	2/210	32/18800	0.049471737	0.089255083	0.041766596	RB1/NOS3	2
BP	GO:0006308	DNA catabolic process	2/210	32/18800	0.049471737	0.089255083	0.041766596	BAX/HSF1	2
BP	GO:0010644	cell communication by electrical coupling	2/210	32/18800	0.049471737	0.089255083	0.041766596	CAV1/GJA1	2
BP	GO:0032366	intracellular sterol transport	2/210	32/18800	0.049471737	0.089255083	0.041766596	LDLR/CES1	2
BP	GO:0035456	response to interferon-beta	2/210	32/18800	0.049471737	0.089255083	0.041766596	STAT1/IRF1	2
BP	GO:0043304	regulation of mast cell degranulation	2/210	32/18800	0.049471737	0.089255083	0.041766596	IL4/HMOX1	2
BP	GO:00450	positive	2/210	32/18	0.0494	0.089255	0.0417665	PPARA/TOP2A	2

	70	regulation of viral genome replication		800	71737	083	96		
BP	GO:0060674	placenta blood vessel development	2/210	32/18800	0.049471737	0.089255083	0.041766596	AKT1/MAPK1	2
BP	GO:0071168	protein localization to chromatin	2/210	32/18800	0.049471737	0.089255083	0.041766596	ESR1/RB1	2
BP	GO:0072337	modified amino acid transport	2/210	32/18800	0.049471737	0.089255083	0.041766596	ABCC1/GJA1	2
BP	GO:1901380	negative regulation of potassium ion transmembrane transport	2/210	32/18800	0.049471737	0.089255083	0.041766596	KCNH2/CAV1	2
BP	GO:1901889	negative regulation of cell junction assembly	2/210	32/18800	0.049471737	0.089255083	0.041766596	IKBKB/IL1B	2
BP	GO:1902624	positive regulation of neutrophil migration	2/210	32/18800	0.049471737	0.089255083	0.041766596	CXCL8/IL1A	2
BP	GO:0002703	regulation of leukocyt	6/210	236/18800	0.049607625	0.089462244	0.041863536	NOS2/IL4/HMOX1/ICAM1/AHR/IL1B	6

		e mediated immunity							
BP	GO:0046470	phosphatidylcholine metabolic process	3/210	74/18800	0.049988805	0.089996802	0.042113681	LDLR/PLB1/PON1	3
BP	GO:0050688	regulation of defense response to virus	3/210	74/18800	0.049988805	0.089996802	0.042113681	IL4/STAT1/IL1B	3
BP	GO:0060420	regulation of heart growth	3/210	74/18800	0.049988805	0.089996802	0.042113681	MAPK14/CDK1/PPARA	3
BP	GO:1901568	fatty acid derivative metabolic process	3/210	74/18800	0.049988805	0.089996802	0.042113681	ALOX5/ACACA/DGAT2	3
CC	GO:0045121	membrane raft	28/210	326/19594	1.68E-17	3.10E-15	2.12E-15	PTGS2/SCN5A/ADRA1A/OPRM1/DPP4/ADRA1B/SLC6A2/SLC6A4/KDR/SLC6A3/OPRD1/OLR1/IKBKB/CASP3/HMOX1/ICAM1/SELE/HAS2/SLC2A4/MAPK3/MAPK1/EGFR/CASP8/CAV1/GJA1/NOS3/ABCG2/CTSD	28
CC	GO:0098857	membrane microdomain	28/210	327/19594	1.82E-17	3.10E-15	2.12E-15	PTGS2/SCN5A/ADRA1A/OPRM1/DPP4/ADRA1B/SLC6A2/SLC6A4/KD	28

								R/SLC6A3/OPRD1/OLR1/IKBKB/CASP3/HMOX1/ICAM1/SELE/HAS2/SLC2A4/MAPK3/MAPK1/EGFR/CASP8/CAV1/GJA1/NOS3/ABCG2/CTSD	
CC	GO:0005667	transcription regulator complex	26/210	483/19594	1.41E-11	1.59E-09	1.09E-09	RXRA/NCOA2/NCOA1/ESR1/PPARG/CDK2/RELA/JUN/STAT1/NR1I2/AHR/RXRB/STAT3/CCND1/RB1/CDK4/FOSL2/FOS/TP53/HIF1A/MYC/NFE2L2/PARP1/RUNX2/E2F1/E2F2	26
CC	GO:0005901	caveola	11/210	82/19594	1.19E-09	1.01E-07	6.90E-08	PTGS2/SCN5A/ADRA1A/ADRA1B/SLC6A3/HMOX1/SELE/MAPK3/MAPK1/CAV1/NOS3	11
CC	GO:0044853	plasma membrane raft	12/210	113/19594	3.21E-09	2.18E-07	1.49E-07	PTGS2/SCN5A/ADRA1A/ADRA1B/SLC6A3/HMOX1/SELE/HAS2/MAPK3/MAPK1/CAV1/NOS3	12
CC	GO:0090575	RNA polymerase II transcription regulator complex	15/210	230/19594	2.89E-08	1.64E-06	1.12E-06	RXRA/NCOA1/PPARG/JUN/STAT1/RXRB/STAT3/RB1/FOSL2/FOS/HIF1A/MYC/NFE2L2/E2F1/E2F2	15
CC	GO:1904813	ficolin-1-rich granule lumen	11/210	124/19594	9.68E-08	4.70E-06	3.22E-06	HSP90AB1/MAPK14/LTA4H/TNFAIP6/ALOX5/GSTP1/PSMD3/MAPK1/CAT/MMP9/CTSD	11

CC	GO:1902911	protein kinase complex	10/210	115/19594	4.52E-07	1.92E-05	1.31E-05	CCNA2/CDK2/IKBKB/CDK1/INSR/CCND1/CDK4/CDKN1A/CCNB1/CHUK	10
CC	GO:0031983	vesicle lumen	16/210	327/19594	5.15E-07	1.95E-05	1.33E-05	HSP90AB1/MAPK14/ALOX5/GSTP1/PSMD3/SLPI/MAPK1/CAT/APOB/EGFR/VEGFA/EGF/SERPINE1/MPO/CTSD/IGF2	16
CC	GO:0000307	cyclin-dependent protein kinase holoenzyme complex	7/210	49/19594	8.62E-07	2.93E-05	2.00E-05	CCNA2/CDK2/CDK1/CCND1/CDK4/CDKN1A/CCNB1	7
CC	GO:1902554	serine/threonine protein kinase complex	9/210	99/19594	1.18E-06	3.65E-05	2.50E-05	CCNA2/CDK2/IKBKB/CDK1/CCND1/CDK4/CDKN1A/CCNB1/CHUK	9
CC	GO:0045177	apical part of cell	17/210	424/19594	3.47E-06	9.83E-05	6.73E-05	ADRB2/DPP4/HSP90AB1/DPEP1/VCAM1/LDLR/PLB1/ABCC1/CA2/IL6R/ERBB2/GJA1/DUOX2/PLAT/ABCG2/CLDN4/ERBB3	17
CC	GO:1905286	serine-type peptidase complex	4/210	11/19594	3.99E-06	0.000104335	7.14E-05	F7/PLAU/F3/THBD	4
CC	GO:0101002	ficolin-1-rich granule	11/210	185/19594	5.21E-06	0.000126571	8.66E-05	HSP90AB1/MAPK14/LTA4H/TNFAIP6/ALOX5/GSTP1/PSMD3/MAPK1/CAT/MMP9/CTSD	11
CC	GO:0099056	integral compon	7/210	67/19594	7.41E-06	0.000163036	0.000111551	CHRM1/ADRA1A/OPRM1/SLC6A4/	7

		ent of presynaptic membrane						DRD1/SLC6A3/OPRD1	
CC	GO:0045211	postsynaptic membrane	13/210	271/19594	7.67E-06	0.000163036	0.000111551	CHRM3/CHRM1/ADRA1A/CHRM2/OPRM1/GABRA1/GRIA2/HTR3A/SLC6A4/DRD1/CHRM5/SLC6A3/OPRD1	13
CC	GO:0034774	secretory granule lumen	14/210	322/19594	1.05E-05	0.000209669	0.000143457	HSP90AB1/MAPK14/ALOX5/GSTP1/PSMD3/SLPI/MAPK1/CAT/VEGFA/EGF/SERPINE1/MPO/CTSD/IGF2	14
CC	GO:0060205	cytoplasmic vesicle lumen	14/210	325/19594	1.16E-05	0.000219808	0.000150395	HSP90AB1/MAPK14/ALOX5/GSTP1/PSMD3/SLPI/MAPK1/CAT/VEGFA/EGF/SERPINE1/MPO/CTSD/IGF2	14
CC	GO:0098889	intrinsic component of presynaptic membrane	7/210	74/19594	1.44E-05	0.000257634	0.000176276	CHRM1/ADRA1A/OPRM1/SLC6A4/DRD1/SLC6A3/OPRD1	7
CC	GO:0031968	organelle outer membrane	11/210	232/19594	4.32E-05	0.000734614	0.000502631	PTGS2/PGR/MAOB/BCL2/BAX/HMOX1/BAD/BCL2L1/CASP8/RAF1/HK2	11
CC	GO:0019867	outer membrane	11/210	234/19594	4.67E-05	0.000756328	0.000517487	PTGS2/PGR/MAOB/BCL2/BAX/HMOX1/BAD/BCL2L1/CASP8/RAF1/HK2	11
CC	GO:0097060	synaptic membrane	14/210	373/19594	5.30E-05	0.000818705	0.000560167	CHRM3/CHRM1/ADRA1A/CHRM2/OPRM1/GABRA1	14

								/GRIA2/SLC6A2/HTR3A/SLC6A4/DRD1/CHRM5/SLC6A3/OPRD1	
CC	GO:0005741	mitochondrial outer membrane	10/210	205/19594	7.61E-05	0.00112533	0.000769963	PGR/MAOB/BCL2/BAX/HMOX1/BAD/BCL2L1/CASP8/RAF1/HK2	10
CC	GO:0030669	clathrin-coated endocytic vesicle membrane	6/210	72/19594	0.000122363	0.001672033	0.001144023	CHRM2/ADRB2/LDLR/APOB/EGFR/EGF	6
CC	GO:0009897	external side of plasma membrane	15/210	455/19594	0.000122944	0.001672033	0.001144023	GRIA2/KDR/ICAM1/SELE/VCAM1/SLC2A4/LDLR/GSR/IL6R/F3/IL2RA/THBD/ABCG2/CXCL10/CD40LG	15
CC	GO:0016324	apical plasma membrane	13/210	358/19594	0.000136347	0.00177249	0.001212756	ADRB2/DPP4/HSP90AB1/DPEP1/PLB1/ABCC1/IL6R/ERBB2/GJA1/DUOX2/ABCG2/CLDN4/ERBB3	13
CC	GO:0005788	endoplasmic reticulum lumen	12/210	311/19594	0.000140757	0.00177249	0.001212756	PTGS2/F7/MAPK3/MAPK1/MTTP/APOB/CES1/HSPA5/COL1A1/COL3A1/SPP1/IGFBP3	12
CC	GO:0042734	presynaptic membrane	8/210	143/19594	0.00015714	0.001908127	0.00130556	CHRM1/ADRA1A/OPRM1/SLC6A2/SLC6A4/DRD1/SLC6A3/OPRD1	8
CC	GO:1904090	peptidase inhibitor complex	3/210	11/19594	0.000187938	0.002203416	0.001507601	PLAU/PLAT/SERPINE1	3
CC	GO:1905368	peptidase complex	7/210	111/19594	0.000194594	0.002205401	0.001508959	F7/PSMD3/CASP9/PLAU/F3/HSPB1/THBD	7
CC	GO:00056	nuclear	15/210	479/1	0.0002	0.002355	0.0016116	PTGS2/ADRA1A/	15

	35	envelope		9594	14765	483	46	ADRA1B/BCL2/BAX/ALOX5/MAPK3/SREBF1/CCND1/CDK4/EGFR/BCL2L1/PTGER3/SULT1E1/PARP1	
CC	GO:0030666	endocytic vesicle membrane	9/210	194/19594	0.000253083	0.002689003	0.001839844	CHRM2/ADRB2/GRIA2/LDLR/APOB/EGFR/EGF/CAV1/NOS3	9
CC	GO:0005790	smooth endoplasmic reticulum	4/210	30/19594	0.000282395	0.002836329	0.001940646	HSD3B2/HSD3B1/APOB/HSPA5	4
CC	GO:0099055	integral component of postsynaptic membrane	7/210	118/19594	0.000283633	0.002836329	0.001940646	CHRM1/ADRA1A/OPRM1/SLC6A4/DRD1/SLC6A3/OPRD1	7
CC	GO:0030139	endocytic vesicle	12/210	342/19594	0.000336415	0.003268027	0.002236019	CHRM2/ADRB2/DPP4/GRIA2/NCF1/LDLR/APOB/EGFR/EGF/CAV1/NOS3/MPO	12
CC	GO:0098936	intrinsic component of postsynaptic membrane	7/210	123/19594	0.000365305	0.003450102	0.002360596	CHRM1/ADRA1A/OPRM1/SLC6A4/DRD1/SLC6A3/OPRD1	7
CC	GO:0045334	clathrin-coated endocytic vesicle	6/210	91/19594	0.000441148	0.004053793	0.002773648	CHRM2/ADRB2/LDLR/APOB/EGFR/EGF	6
CC	GO:0061695	transferase complex, transferring	10/210	259/19594	0.00050227	0.004493991	0.003074836	CCNA2/CDK2/IKBKB/CDK1/INSRR/CCND1/CDK4/CDKN1A/CCNB1/CHUK	10

		phosphorus-containing groups							
CC	GO:0042383	sarcolemma	7/210	131/19594	0.000534194	0.004657078	0.003186422	SCN5A/ADRA1A/OPRM1/VCAM1/SLC2A4/PPP3CA/CAV1	7
CC	GO:0016328	lateral plasma membrane	5/210	64/19594	0.000615271	0.005110636	0.003496751	SCN5A/ABCC1/GJA1/CLDN4/ERBB3	5
CC	GO:0005925	focal adhesion	13/210	419/19594	0.000616283	0.005110636	0.003496751	OPRM1/DPP4/SLC6A4/ICAM1/MAPK3/MAPK1/CAT/EGFR/PLAU/HSPA5/CAV1/GJA1/HSPB1	13
CC	GO:0030055	cell-substrate junction	13/210	428/19594	0.000750007	0.006058877	0.004145548	OPRM1/DPP4/SLC6A4/ICAM1/MAPK3/MAPK1/CAT/EGFR/PLAU/HSPA5/CAV1/GJA1/HSPB1	13
CC	GO:0062023	collagen-containing extracellular matrix	13/210	429/19594	0.00076627	0.006058877	0.004145548	ACHE/PRSS1/F7/ICAM1/SLPI/MMP2/MMP9/F3/SERPINE1/COL1A1/COL3A1/CTSD/PCOLCE	13
CC	GO:0031143	pseudopodium	3/210	18/19594	0.000879362	0.006795068	0.004649257	MAPK3/MAPK1/RAF1	3
CC	GO:0031253	cell projection membrane	11/210	339/19594	0.001121894	0.008441401	0.005775695	OPRM1/GABRA1/DPP4/HSP90AB1/DPEP1/DRD1/OPRD1/MTTP/PLB1/EGFR/ABCG2	11
CC	GO:0099699	integral component of synaptic membrane	7/210	149/19594	0.001142072	0.008441401	0.005775695	CHRM1/ADRA1A/OPRM1/SLC6A4/DRD1/SLC6A3/OPRD1	7
CC	GO:00306	clathrin-	6/210	111/1	0.0012	0.009085	0.0062163	CHRM2/ADRB2/L	6

	65	coated vesicle membrane		9594	55937	504	97	DLR/APOB/EGFR/EGF	
CC	GO:0031965	nuclear membrane	10/210	300/19594	0.001534126	0.010865706	0.00743443	PTGS2/ADRA1A/ADRA1B/BCL2/ALOX5/CCND1/CDK4/EGFR/BCL2L1/SULT1E1	10
CC	GO:0009925	basal plasma membrane	9/210	251/19594	0.001593796	0.010865706	0.00743443	CHRM3/HSP90AB1/LDLR/MTTP/ABCC1/EGFR/ERBB2/CLDN4/ERBB3	9
CC	GO:1905369	endopeptidase complex	5/210	79/19594	0.001597898	0.010865706	0.00743443	PSMD3/CASP9/PLAU/HSPB1/THBD	5
CC	GO:0099240	intrinsic component of synaptic membrane	7/210	160/19594	0.001721135	0.011474232	0.007850791	CHRM1/ADRA1A/OPRM1/SLC6A4/DRD1/SLC6A3/OPRD1	7
CC	GO:0043025	neuronal cell body	13/210	482/19594	0.002170703	0.014193059	0.00971104	OPRM1/GRIA2/SLC6A2/HSP90AB1/SLC6A3/NCF1/CASP3/SOD1/APOB/ELK1/TOP1/NQO1/MAP2	13
CC	GO:0045178	basal part of cell	9/210	269/19594	0.002547599	0.016343086	0.011182112	CHRM3/HSP90AB1/LDLR/MTTP/ABCC1/EGFR/ERBB2/CLDN4/ERBB3	9
CC	GO:0031045	dense core granule	3/210	26/19594	0.002630665	0.016563449	0.011332886	OPRD1/SOD1/PLAT	3
CC	GO:0120111	neuron projection cytoplasm	5/210	89/19594	0.00270031	0.016692825	0.011421407	OPRM1/SOD1/HIF1A/HSPB1/MAP2	5
CC	GO:0031526	brush border	4/210	55/19594	0.00285276	0.017016465	0.011642844	HSP90AB1/MTTP/PLB1/ABCG2	4

CC		membrane							
CC	GO:1904724	tertiary granule lumen	4/210	55/19594	0.00285276	0.017016465	0.011642844	LTA4H/TNFAIP6/MMP9/CTSD	4
CC	GO:0030662	coated vesicle membrane	7/210	176/19594	0.002943301	0.017253833	0.011805254	CHRM2/ADRB2/LDLR/APOB/SREBF1/EGFR/EGF	7
CC	GO:0016323	basolateral plasma membrane	8/210	226/19594	0.003103649	0.017885432	0.012237401	CHRM3/HSP90AB1/LDLR/MTTP/ABCC1/EGFR/ERBB2/ERBB3	8
CC	GO:0031970	organelle envelope lumen	5/210	93/19594	0.003266877	0.018288165	0.012512955	HSD3B2/HSD3B1/ALOX5/SOD1/CAT	5
CC	GO:0005769	early endosome	11/210	389/19594	0.003281112	0.018288165	0.012512955	ADRB2/KDR/ADRB1/VCAM1/MAPK3/MAPK1/LDLR/APOB/EGFR/ERBB2/CAV1	11
CC	GO:0032839	dendrite cytoplasm	3/210	30/19594	0.003980783	0.021830099	0.014936384	OPRM1/SOD1/MAP2	3
CC	GO:0030136	clathrin-coated vesicle	7/210	192/19594	0.004741131	0.025587057	0.017506934	CHRM2/ADRB2/SLC2A4/LDLR/APOB/EGFR/EGF	7
CC	GO:0005903	brush border	5/210	102/19594	0.004846927	0.0257493	0.017617942	HSP90AB1/MTTP/PLB1/SOAT2/ABCG2	5
CC	GO:0031093	platelet alpha granule lumen	4/210	67/19594	0.005804199	0.029498977	0.020183511	VEGFA/EGF/SERPINE1/IGF2	4
CC	GO:0034663	endoplasmic reticulum chaperone complex	2/210	11/19594	0.005899795	0.029498977	0.020183511	UGT1A1/HSPA5	2
CC	GO:00356	CD40	2/210	11/19	0.0058	0.029498	0.0201835	IKBKB/CHUK	2

	31	receptor complex		594	99795	977	11		
CC	GO:0098691	dopaminergic synapse	2/210	11/19594	0.005899795	0.029498977	0.020183511	ADRA1A/SLC6A3	2
CC	GO:0034358	plasma lipoprotein particle	3/210	36/19594	0.006679132	0.031450148	0.021518523	LDLR/APOB/PON1	3
CC	GO:1990777	lipoprotein particle	3/210	36/19594	0.006679132	0.031450148	0.021518523	LDLR/APOB/PON1	3
CC	GO:0098802	plasma membrane signaling receptor complex	9/210	313/19594	0.006806539	0.031450148	0.021518523	GRIA2/HTR3A/IKBKB/INSRR/IL6R/ERBB2/IL2RA/CHUK/ERBB3	9
CC	GO:0098793	presynapse	12/210	492/19594	0.006987883	0.031450148	0.021518523	CHRM1/ADRA1A/OPRM1/SLC6A2/SLC6A4/DRD1/SLC6A3/OPRD1/SLC2A4/BCL2L1/ELK1/PRKCB	12
CC	GO:0005583	fibrillar collagen trimer	2/210	12/19594	0.007030033	0.031450148	0.021518523	COL1A1/COL3A1	2
CC	GO:0034362	low-density lipoprotein particle	2/210	12/19594	0.007030033	0.031450148	0.021518523	LDLR/APOB	2
CC	GO:0044292	dendrite terminus	2/210	12/19594	0.007030033	0.031450148	0.021518523	HSP90AB1/MAP2	2
CC	GO:0098643	banded collagen fibril	2/210	12/19594	0.007030033	0.031450148	0.021518523	COL1A1/COL3A1	2
CC	GO:0098685	Schaffer collateral - CA1 synapse	4/210	72/19594	0.007475221	0.033007471	0.022584059	CHRM1/ADRB1/PPP3CA/PLAT	4
CC	GO:0098992	neuronal dense	2/210	13/19594	0.008249923	0.035940703	0.024591007	OPRD1/PLAT	2

		core vesicle							
CC	GO:0032994	protein-lipid complex	3/210	39/19594	0.008350928	0.035940703	0.024591007	LDLR/APOB/PON1	3
CC	GO:0070820	tertiary granule	6/210	164/19594	0.008585709	0.036164109	0.024743864	OLR1/LTA4H/TNFAIP6/PLAU/MMP9/CTSD	6
CC	GO:0045171	intercellular bridge	4/210	75/19594	0.008615567	0.036164109	0.024743864	HSD3B2/HSD3B1/GSTM1/GSTM2	4
CC	GO:0017053	transcription repressor complex	4/210	76/19594	0.009019534	0.03739807	0.025588153	JUN/CCND1/TP53/MYC	4
CC	GO:0043020	NADPH oxidase complex	2/210	14/19594	0.009557432	0.03881607	0.026558364	NCF1/DUOX2	2
CC	GO:0032590	dendrite membrane	3/210	41/19594	0.009589853	0.03881607	0.026558364	OPRM1/GABRA1/OPRD1	3
CC	GO:0005819	spindle	10/210	402/19594	0.011708533	0.04683413	0.032044405	MAPK14/AKT1/CDK1/MAPK1/RB1/BIRC5/HSPB1/CCNB1/HSF1/RASSF1	10
CC	GO:0005758	mitochondrial intermembrane space	4/210	83/19594	0.012195327	0.048214082	0.032988582	HSD3B2/HSD3B1/SOD1/CAT	4
CC	GO:0043209	myelin sheath	3/210	45/19594	0.012373614	0.048356652	0.03308613	BCL2/CA2/ERBB2	3
CC	GO:0030135	coated vesicle	8/210	290/19594	0.013201443	0.051005574	0.034898551	CHRM2/ADRB2/SLC2A4/LDLR/APOB/SREBF1/EGFR/EGF	8
CC	GO:0031091	platelet alpha granule	4/210	91/19594	0.016609674	0.063452687	0.043414996	VEGFA/EGF/SERPINE1/IGF2	4
CC	GO:0030315	T-tubule	3/210	51/19594	0.017331816	0.065475749	0.044799197	SCN5A/ADRA1A/SLC2A4	3
CC	GO:00986	chromos	9/210	366/1	0.0175	0.065477	0.0448005	CHEK1/CDK2/CD	9

	87	omal region		9594	24931	765	76	K1/BIRC5/CCNB1/TOP2A/PARP1/CHEK2/HSF1	
CC	GO:0016327	apicolateral plasma membrane	2/210	20/19594	0.019132744	0.070707966	0.048379135	THBD/CLDN4	2
MF	GO:0004879	nuclear receptor activity	15/210	52/18410	1.34E-17	3.85E-15	2.24E-15	PGR/AR/RXRA/NR3C2/ESR1/PPARG/ESR2/PPARD/NR1I2/AHR/NR1I3/PPARA/SREBF1/RXRB/STAT3	15
MF	GO:0098531	ligand-activated transcription factor activity	15/210	52/18410	1.34E-17	3.85E-15	2.24E-15	PGR/AR/RXRA/NR3C2/ESR1/PPARG/ESR2/PPARD/NR1I2/AHR/NR1I3/PPARA/SREBF1/RXRB/STAT3	15
MF	GO:0140297	DNA-binding transcription factor binding	32/210	470/18410	3.68E-16	7.02E-14	4.08E-14	AR/RXRA/NCOA2/NCOA1/ESR1/PPARG/MAPK14/GSK3B/PPARD/RELA/JUN/BCL2/STAT1/NR1I2/AHR/PPARA/STAT3/RB1/FOS/TP53/ELK1/NFKBIA/HIF1A/RUNX1T1/MYC/PRKCB/HSPB1/NFE2L2/PARP1/HSF1/RUNX2/E2F1	32
MF	GO:0061629	RNA polymerase II-specific DNA-binding transcription factor binding	27/210	348/18410	3.77E-15	5.40E-13	3.13E-13	AR/RXRA/NCOA2/NCOA1/ESR1/PPARG/MAPK14/GSK3B/PPARD/RELA/JUN/STAT1/NR1I2/AHR/PPARA/STAT3/RB1/FOS/TP53/ELK1/NFKBIA/HIF1A/PRKCB/HSPB1/NFE2L2/PARP1/HSF1	27

MF	GO:0001221	transcription coregulator binding	15/210	108/18410	1.50E-12	1.72E-10	9.99E-11	PGR/AR/RXRA/ESR1/PPARG/PPARD/RELA/STAT1/AHR/PPARA/SREBF1/FOS/HIF1A/MYC/NFE2L2	15
MF	GO:0003707	nuclear steroid receptor activity	8/210	24/18410	1.58E-10	1.51E-08	8.74E-09	PGR/RXRA/NR3C2/ESR1/ESR2/PPARD/PPARA/RXRB	8
MF	GO:0001223	transcription coactivator binding	9/210	40/18410	5.54E-10	4.54E-08	2.63E-08	PGR/AR/ESR1/PPARD/RELA/STAT1/AHR/PPARA/HIF1A	9
MF	GO:0044389	ubiquitin-like protein ligase binding	19/210	317/18410	4.30E-09	3.08E-07	1.79E-07	SCN5A/HSP90AB1/GSK3B/RELA/JUN/BCL2/STAT1/KCNH2/RB1/EGFR/CDKN1A/TP53/NFKBIA/CASP8/HIF1A/HSPA5/CCNB1/CHEK2/ERBB3	19
MF	GO:0008227	G protein-coupled amine receptor activity	9/210	51/18410	5.53E-09	3.52E-07	2.05E-07	CHRM3/CHRM1/ADRA1A/CHRM2/ADRB2/ADRA1B/ADRA1D/CHRM5/ADRB1	9
MF	GO:0020037	heme binding	13/210	139/18410	7.03E-09	4.03E-07	2.34E-07	PTGS2/PTGS1/NOS2/HMOX1/CYP3A4/CYP1A2/CYP1A1/CYP1B1/CAT/CYP19A1/DUOX2/NOS3/MPO	13
MF	GO:0046906	tetrapyrrole binding	13/210	149/18410	1.63E-08	8.52E-07	4.94E-07	PTGS2/PTGS1/NOS2/HMOX1/CYP3A4/CYP1A2/CYP1A1/CYP1B1/CAT/CYP19A1/DUOX2/NOS3/MPO	13
MF	GO:0005496	steroid binding	11/210	100/18410	1.91E-08	8.70E-07	5.05E-07	PGR/AR/NR3C2/ESR1/ESR2/CYP3A	11

MF								4/UGT1A1/SOAT2/SOAT1/CAV1/SULT1E1	
MF	GO:0001216	DNA-binding transcription activator activity	22/210	466/18410	1.97E-08	8.70E-07	5.05E-07	PGR/AR/ESR1/RELA/JUN/NR1I2/NR1I3/PPARA/SREBF1/RXRB/STAT3/FOSL2/FOS/TP53/ELK1/HIF1A/MYC/NFE2L2/RUNX2/E2F1/E2F2/IRF1	22
MF	GO:0004712	protein serine/threonine/tyrosine kinase activity	21/210	446/18410	4.42E-08	1.60E-06	9.30E-07	KDR/MAPK14/GSK3B/CHEK1/CDK2/MAPK10/IKBKB/AKT1/MAPK8/CDK1/INSRR/MAPK3/MAPK1/CDK4/EGFR/RAF1/PRKCA/ERBB2/PRKCB/CHEK2/ERBB3	21
MF	GO:0099528	G protein-coupled neurotransmitter receptor activity	5/210	10/18410	4.43E-08	1.60E-06	9.30E-07	CHRM3/CHRM1/CHRM2/CHRM5/ADRB1	5
MF	GO:0016209	antioxidant activity	10/210	85/18410	4.47E-08	1.60E-06	9.30E-07	PTGS2/PTGS1/GSTP1/GSTM2/SOD1/CAT/GSR/DUOX2/MPO/NQO1	10
MF	GO:0019902	phosphatase binding	14/210	193/18410	4.79E-08	1.62E-06	9.38E-07	MAPK14/SLC6A3/BCL2/MAPK8/STAT1/MAPK3/MAPK1/BAD/SOD1/PPARA/STAT3/EGFR/TP53/ERBB2	14
MF	GO:0031625	ubiquitin protein ligase binding	17/210	298/18410	5.98E-08	1.90E-06	1.10E-06	SCN5A/HSP90AB1/GSK3B/RELA/JUN/BCL2/KCNH2/RB1/EGFR/CDKN1A/TP53/NFKBI	17

								A/CASP8/HIF1A/HSPA5/CHEK2/ERBB3	
MF	GO:0001228	DNA-binding transcription activator activity, RNA polymerase II-specific	21/210	462/18410	8.03E-08	2.42E-06	1.41E-06	PGR/AR/ESR1/RELA/JUN/NR1I2/NR1I3/PPARA/SREBF1/RXRB/STAT3/FOSL2/FOS/TP53/ELK1/HIF1A/MYC/NFE2L2/RUNX2/E2F2/IRF1	21
MF	GO:0033218	amide binding	19/210	402/18410	1.89E-07	5.42E-06	3.14E-06	RXRA/ACHE/ADRB2/OPRM1/GRIA2/HSP90AB1/PPARG/OPRD1/RELA/PPP3CA/GSTM1/GSTM2/FASN/LDLR/CAT/SOAT2/SOAT1/NFKBIA/NPEPPS	19
MF	GO:0046982	protein heterodimerization activity	17/210	332/18410	2.80E-07	7.64E-06	4.44E-06	ADRA1A/ADRA1B/ADRB1/IKBKB/BCL2/BAX/AHR/MTTP/BCL2L1/TP53/HIF1A/ERBB2/CAV1/TOP2A/HSF1/CHUK/ERBB3	17
MF	GO:0002020	protease binding	11/210	136/18410	4.59E-07	1.20E-05	6.94E-06	DPP4/GSK3B/SLC6A3/BCL2/CASP3/LDLR/TP53/F3/SERPINE1/COL1A1/COL3A1	11
MF	GO:0004707	MAP kinase activity	5/210	15/18410	5.04E-07	1.21E-05	7.04E-06	MAPK14/MAPK10/MAPK8/MAPK3/MAPK1	5
MF	GO:0004674	protein serine/threonine kinase activity	19/210	430/18410	5.28E-07	1.21E-05	7.04E-06	MAPK14/GSK3B/CHEK1/CDK2/MAPK10/IKBKB/AKT1/MAPK8/CDK1/MAPK3/MAPK1/CDK4/EGFR/TOP	19

								1/RAF1/PRKCA/PRKCB/CHEK2/CHUK	
MF	GO:0019207	kinase regulator activity	14/210	235/18410	5.42E-07	1.21E-05	7.04E-06	HSP90AB1/CCNA2/CASP3/GSTP1/CCND1/CDK4/CDKN1A/EGF/HSPB1/CCNB1/CXCL10/IGF2/CD40LG/ERBB3	14
MF	GO:0005126	cytokine receptor binding	15/210	272/18410	5.66E-07	1.21E-05	7.04E-06	IL4/CASP3/STAT1/VEGFA/IL6R/CASP8/IL1B/CCL2/CXCL8/IFNG/IL1A/CXCL11/CXCL2/CXCL10/CD40LG	15
MF	GO:0019838	growth factor binding	11/210	139/18410	5.72E-07	1.21E-05	7.04E-06	SCN5A/KDR/EGFR/IL10RA/IL6R/ERBB2/IL2RA/COL1A1/COL3A1/IGFBP3/ERBB3	11
MF	GO:0035173	histone kinase activity	5/210	16/18410	7.26E-07	1.49E-05	8.63E-06	CHEK1/CDK2/CDK1/PRKCA/PRKCB	5
MF	GO:0019887	protein kinase regulator activity	13/210	207/18410	7.68E-07	1.52E-05	8.81E-06	HSP90AB1/CCNA2/CASP3/CCND1/CDK4/CDKN1A/EGF/HSPB1/CCNB1/CXCL10/IGF2/CD40LG/ERBB3	13
MF	GO:0016705	oxidoreductase activity, acting on paired donors, with incorporation or reduction of molecul	12/210	177/18410	9.20E-07	1.75E-05	1.01E-05	PTGS2/PTGS1/NOS2/HMOX1/CYP3A4/CYP1A2/CYP1A1/CYP1B1/AKR1C3/CYP19A1/POR/NOS3	12

		ar oxygen							
MF	GO:0042277	peptide binding	16/210	322/18410	9.45E-07	1.75E-05	1.01E-05	RXRA/ACHE/ADRB2/OPRM1/GRIA2/HSP90AB1/PPARG/OPRD1/RELA/PPP3CA/GSTM1/GSTM2/LDLR/CAT/NFKBIA/NPEPPS	16
MF	GO:0019903	protein phosphatase binding	11/210	148/18410	1.07E-06	1.91E-05	1.11E-05	MAPK14/SLC6A3/BCL2/MAPK8/STAT1/BAD/SOD1/STAT3/EGFR/TP53/ERBB2	11
MF	GO:0031072	heat shock protein binding	10/210	123/18410	1.47E-06	2.56E-05	1.48E-05	HSP90AB1/KDR/BAX/AHSA1/CDK1/CYP1A1/AHR/HIF1A/HSPA5/HSF1	10
MF	GO:0004601	peroxidase activity	7/210	52/18410	1.97E-06	3.22E-05	1.87E-05	PTGS2/PTGS1/GSTP1/GSTM2/CAT/DUOX2/MPO	7
MF	GO:0140296	general transcription initiation factor binding	7/210	52/18410	1.97E-06	3.22E-05	1.87E-05	AR/ESR1/RELA/JUN/AHR/TP53/HSF1	7
MF	GO:0004497	monooxygenase activity	9/210	103/18410	2.74E-06	4.36E-05	2.53E-05	NOS2/HMOX1/CYP3A4/CYP1A2/CYP1A1/CYP1B1/AKR1C3/CYP19A1/NOS3	9
MF	GO:0016684	oxidoreductase activity, acting on peroxide as acceptor	7/210	56/18410	3.28E-06	5.08E-05	2.95E-05	PTGS2/PTGS1/GSTP1/GSTM2/CAT/DUOX2/MPO	7
MF	GO:0106310	protein serine	16/210	360/18410	4.01E-06	6.04E-05	3.51E-05	MAPK14/GSK3B/CHEK1/CDK2/M	16

		kinase activity						APK10/IKBKB/AKT1/MAPK8/CDK1/MAPK3/MAPK1/CDK4/RAF1/PRKCA/PRKCB/CHEK2	
MF	GO:0016922	nuclear receptor binding	10/210	139/18410	4.46E-06	6.56E-05	3.81E-05	RXRA/NCOA2/NCOA1/ESR1/PPARG/STAT1/NR1I2/HIF1A/PRKCB/PARP1	10
MF	GO:0030594	neurotransmitter receptor activity	9/210	111/18410	5.09E-06	7.30E-05	4.24E-05	CHRM3/CHRM1/CHRM2/GABRA1/GRIA2/HTR3A/DRD1/CHRM5/ADRB1	9
MF	GO:0016712	oxidoreductase activity, acting on paired donors, with incorporation or reduction of molecular oxygen, reduced flavin or flavoprotein as one donor, and incorporation of one atom of oxygen	6/210	40/18410	5.70E-06	7.96E-05	4.62E-05	HMOX1/CYP3A4/CYP1A2/CYP1A1/CYP1B1/CYP19A1	6
MF	GO:00703	aromata	5/210	25/18	8.12E-	0.000108	6.30E-05	CYP3A4/CYP1A2/	5

	30	se activity		410	06	483		CYP1A1/CYP1B1/CYP19A1	
MF	GO:0098960	postsynaptic neurotransmitter receptor activity	7/210	64/18410	8.14E-06	0.000108483	6.30E-05	CHRM3/CHRM1/CHRM2/GABRA1/DRD1/CHRM5/ADRB1	7
MF	GO:0051879	Hsp90 protein binding	6/210	43/18410	8.79E-06	0.000114516	6.65E-05	KDR/AHSA1/CYP1A1/AHR/HIF1A/HSF1	6
MF	GO:0097110	scaffold protein binding	7/210	67/18410	1.11E-05	0.000140983	8.18E-05	SCN5A/IKBKB/KCNH2/CASP8/GJA1/NOS3/CHUK	7
MF	GO:0017171	serine hydrolase activity	11/210	195/18410	1.53E-05	0.000190566	0.000110625	ACHE/DPP4/PRSS1/F7/MMP1/MMP3/PLAU/MMP2/MMP9/F3/PLAT	11
MF	GO:0005125	cytokine activity	12/210	235/18410	1.71E-05	0.000207898	0.000120686	IL4/VEGFA/IL1B/CCL2/CXCL8/IFNG/IL1A/CXCL11/CXCL2/CXCL10/SPP1/CD40LG	12
MF	GO:0016538	cyclin-dependent protein serine/threonine kinase regulator activity	6/210	50/18410	2.14E-05	0.00025604	0.000148634	CCNA2/CASP3/CCND1/CDK4/CDKN1A/CCNB1	6
MF	GO:0050661	NADP binding	6/210	53/18410	3.01E-05	0.000348638	0.000202387	NOS2/CAT/HMGCR/GSR/POR/NOS3	6
MF	GO:0031406	carboxylic acid binding	10/210	173/18410	3.04E-05	0.000348638	0.000202387	RXRA/NOS2/PPARG/PPARD/SELE/GSTP1/GSTM2/AKR1C3/UGT1A1/NOS3	10
MF	GO:0004252	serine-type endopeptidase	10/210	174/18410	3.20E-05	0.000359087	0.000208453	DPP4/PRSS1/F7/MMP1/MMP3/PLAU/MMP2/MMP9/F3/PLAT	10

		activity							
MF	GO:0033293	monocarboxylic acid binding	7/210	81/18410	3.87E-05	0.000426338	0.000247493	RXRA/PPARG/PPARD/GSTP1/GSTM2/AKR1C3/UGT1A1	7
MF	GO:0004708	MAP kinase kinase activity	4/210	18/18410	4.44E-05	0.000471241	0.00027356	MAPK14/MAPK10/MAPK3/MAPK1	4
MF	GO:0045236	CXCR chemokine receptor binding	4/210	18/18410	4.44E-05	0.000471241	0.00027356	CXCL8/CXCL11/CXCL2/CXCL10	4
MF	GO:1901681	sulfur compound binding	12/210	267/18410	5.94E-05	0.000619353	0.00035954	GSTP1/GSTM1/GSTM2/APOB/SOAT2/SOAT1/VEGFA/CXCL8/MPO/CXCL11/CXCL10/PCOLCE	12
MF	GO:0008395	steroid hydroxylase activity	5/210	38/18410	6.80E-05	0.000696137	0.000404114	CYP3A4/CYP1A2/CYP1A1/CYP1B1/CYP19A1	5
MF	GO:0008236	serine-type peptidase activity	10/210	191/18410	7.03E-05	0.000706905	0.000410365	DPP4/PRSS1/F7/MMP1/MMP3/PLAU/MMP2/MMP9/F3/PLAT	10
MF	GO:0005178	integrin binding	9/210	156/18410	7.73E-05	0.000763645	0.000443303	KDR/ICAM1/VCAM1/PRKCA/IL1B/COL3A1/SPP1/IGF2/CD40LG	9
MF	GO:0042826	histone deacetylase binding	8/210	126/18410	9.96E-05	0.000967414	0.000561592	HSP90AB1/RELA/MAPK8/CCND1/TP53/HIF1A/TOP2A/PARP1	8
MF	GO:0015464	acetylcholine receptor activity	4/210	22/18410	0.000102431	0.00097822	0.000567865	CHRM3/CHRM1/CHRM2/CHRM5	4
MF	GO:0001046	core promoter	5/210	42/18410	0.000111113	0.001027853	0.000596678	RELA/STAT1/FOS/TP53/MYC	5

		sequence-specific DNA binding							
MF	GO:0016616	oxidoreductase activity, acting on the CH-OH group of donors, NAD or NADP as acceptor	8/210	128/18410	0.000111216	0.001027853	0.000596678	ADH1B/ADH1C/AKR1B1/HSD3B2/HSD3B1/AKR1C3/FASN/HMGCR	8
MF	GO:0016709	oxidoreductase activity, acting on paired donors, with incorporation or reduction of molecular oxygen, NAD(P)H as one donor, and incorporation of one atom of oxygen	5/210	43/18410	0.000124576	0.001133048	0.000657745	NOS2/CYP3A4/CYP1A1/AKR1C3/NOS3	5
MF	GO:0004175	endopeptidase activity	15/210	432/18410	0.000136883	0.001225527	0.000711429	DPP4/PRSS1/F7/CASP3/MMP1/BACE2/MMP3/CASP9/PLAU/MMP2/MM	15

								P9/CASP8/F3/PLAT/CTSD	
MF	GO:0001091	RNA polymerase II general transcription initiation factor binding	4/210	24/18410	0.000146159	0.001268927	0.000736623	AR/ESR1/AHR/TP53	4
MF	GO:0070412	R-SMAD binding	4/210	24/18410	0.000146159	0.001268927	0.000736623	PPARG/JUN/FOS/PARP1	4
MF	GO:0042379	chemokine receptor binding	6/210	71/18410	0.000158474	0.001355311	0.00078677	STAT1/CCL2/CXCL8/CXCL11/CXCL2/CXCL10	6
MF	GO:0015378	sodium:chloride symporter activity	3/210	10/18410	0.000165505	0.00137441	0.000797857	SLC6A2/SLC6A4/SLC6A3	3
MF	GO:0097199	cysteine-type endopeptidase activity involved in apoptotic signaling pathway	3/210	10/18410	0.000165505	0.00137441	0.000797857	CASP3/CASP9/CASP8	3
MF	GO:0016614	oxidoreductase activity, acting on CH-OH group of donors	8/210	140/18410	0.000206751	0.001692406	0.000982457	ADH1B/ADH1C/AKR1B1/HSD3B2/HSD3B1/AKR1C3/FASN/HMGCR	8
MF	GO:00705	death	3/210	11/18	0.0002	0.001821	0.0010571	BCL2/BAX/BCL2	3

	13	domain binding		410	25657	147	92	L1		
MF	GO:0008009	chemokine activity	5/210	49/18410	0.000233477	0.001858088	0.001078637	CCL2/CXCL8/CXCL11/CXCL2/CXCL10	5
MF	GO:0008353	RNA polymerase II CTD heptapeptide repeat kinase activity	3/210	12/18410	0.000298349	0.002341835	0.001359456	CDK1/MAPK1/CDK4	3
MF	GO:0051117	ATPase binding	6/210	82/18410	0.000349266	0.002704453	0.001569959	PGR/AR/ESR1/PPP3CA/EGFR/CAV1	6
MF	GO:0051721	protein phosphatase 2A binding	4/210	30/18410	0.000357297	0.002729752	0.001584645	SLC6A3/BCL2/STAT1/TP53	4
MF	GO:0015373	anion:sodium symporter activity	3/210	13/18410	0.000384598	0.002789552	0.00161936	SLC6A2/SLC6A4/SLC6A3	3
MF	GO:0043176	amine binding	3/210	13/18410	0.000384598	0.002789552	0.00161936	HTR3A/SLC6A4/SLC6A3	3
MF	GO:0050998	nitric-oxide synthase binding	3/210	13/18410	0.000384598	0.002789552	0.00161936	SCN5A/SLC6A4/CAV1	3
MF	GO:1901338	catecholamine binding	3/210	13/18410	0.000384598	0.002789552	0.00161936	ADRB2/DRD1/SLC6A3	3
MF	GO:0001968	fibronectin binding	4/210	31/18410	0.00040659	0.002876247	0.001669687	TNFAIP6/VEGFA/MMP2/IGFBP3	4
MF	GO:0051059	NF-kappaB binding	4/210	31/18410	0.00040659	0.002876247	0.001669687	GSK3B/PPARD/RELA/NFKBIA	4
MF	GO:0015562	efflux transmembrane	3/210	14/18410	0.000485382	0.003391752	0.001968942	ABCC1/GJA1/ABCG2	3

		transporter activity							
MF	GO:0048018	receptor ligand activity	15/210	489/18410	0.000511997	0.003534629	0.002051884	DPP4/IL4/VEGFA/EGF/IL1B/CCL2/CXCL8/IFNG/IL1A/CXCL11/CXCL2/CXCL10/SPP1/IGF2/CD40LG	15
MF	GO:0009055	electron transfer activity	7/210	125/18410	0.000581939	0.003830438	0.002223603	MAOB/AKR1B1/NCF1/CYP1A2/CYP19A1/GSR/POR	7
MF	GO:0004993	G protein-coupled serotonin receptor activity	4/210	34/18410	0.000583468	0.003830438	0.002223603	CHRM3/CHRM1/CHRM2/CHRM5	4
MF	GO:0030332	cyclin binding	4/210	34/18410	0.000583468	0.003830438	0.002223603	CDK2/CDK1/CDK4/CDKN1A	4
MF	GO:0099589	serotonin receptor activity	4/210	34/18410	0.000583468	0.003830438	0.002223603	CHRM3/CHRM1/CHRM2/CHRM5	4
MF	GO:0030546	signaling receptor activator activity	15/210	496/18410	0.000592769	0.003830438	0.002223603	DPP4/IL4/VEGFA/EGF/IL1B/CCL2/CXCL8/IFNG/IL1A/CXCL11/CXCL2/CXCL10/SPP1/IGF2/CD40LG	15
MF	GO:0010181	FMN binding	3/210	15/18410	0.00060164	0.003830438	0.002223603	NOS2/POR/NOS3	3
MF	GO:0097153	cysteine-type endopeptidase activity involved in apoptotic process	3/210	15/18410	0.00060164	0.003830438	0.002223603	CASP3/CASP9/CASP8	3
MF	GO:00153	cation:c	3/210	16/18	0.0007	0.004623	0.0026839	SLC6A2/SLC6A4/	3

	77	hloride symporter activity		410	34274	504	85	SLC6A3	
MF	GO:0016836	hydro-lyase activity	5/210	64/18410	0.00081305	0.005063888	0.002939632	CYP1A2/CYP1A1/CYP1B1/FASN/CA2	5
MF	GO:0030295	protein kinase activator activity	6/210	104/18410	0.001230618	0.0074726	0.00433791	CDKN1A/EGF/CCNB1/IGF2/CD40LG/ERBB3	6
MF	GO:0004745	NAD-retinol dehydrogenase activity	3/210	19/18410	0.001238913	0.0074726	0.00433791	ADH1B/ADH1C/AKR1C3	3
MF	GO:0005326	neurotransmitter transmembrane transporter activity	3/210	19/18410	0.001238913	0.0074726	0.00433791	SLC6A2/SLC6A4/SLC6A3	3
MF	GO:0051087	chaperone binding	6/210	106/18410	0.001357792	0.008104322	0.004704631	BAX/AHSA1/SOD1/TP53/HSPA5/BIRC5	6
MF	GO:0000979	RNA polymerase II core promoter sequence-specific DNA binding	3/210	20/18410	0.001445356	0.008538029	0.004956402	RELA/STAT1/FOS	3
MF	GO:0019209	kinase activator activity	6/210	110/18410	0.001642018	0.00960078	0.005573338	CDKN1A/EGF/CCNB1/IGF2/CD40LG/ERBB3	6
MF	GO:0015296	anion:cation symporter activity	3/210	21/18410	0.001672155	0.009678231	0.005618299	SLC6A2/SLC6A4/SLC6A3	3

MF	GO:0001618	virus receptor activity	5/210	76/18410	0.001764583	0.010111062	0.005869561	DPP4/CDK1/ICAM1/LDLR/EGFR	5
MF	GO:0140272	exogenous protein binding	5/210	77/18410	0.001869908	0.010608489	0.006158322	DPP4/CDK1/ICAM1/LDLR/EGFR	5
MF	GO:0046332	SMAD binding	5/210	78/18410	0.001979762	0.011121605	0.00645619	PPARG/JUN/FOS/PARP1/COL3A1	5
MF	GO:0016835	carbon-oxygen lyase activity	5/210	79/18410	0.002094259	0.011650587	0.006763269	CYP1A2/CYP1A1/CYP1B1/FASN/CA2	5
MF	GO:0005504	fatty acid binding	4/210	49/18410	0.002333293	0.01261299	0.007321953	PPARG/PPARD/GSTP1/GSTM2	4
MF	GO:0032813	tumor necrosis factor receptor superfamily binding	4/210	49/18410	0.002333293	0.01261299	0.007321953	CASP3/STAT1/CASP8/CD40LG	4
MF	GO:0070888	E-box binding	4/210	49/18410	0.002333293	0.01261299	0.007321953	PPARG/AHR/HIF1A/MYC	4
MF	GO:0016702	oxidoreductase activity, acting on single donors with incorporation of molecular oxygen, incorporation of two atoms of oxygen	3/210	24/18410	0.002481489	0.013288724	0.007714222	PTGS2/PTGS1/ALOX5	3
MF	GO:00167	oxidored	3/210	25/18	0.0027	0.014836	0.0086125	PTGS2/PTGS1/AL	3

	01	uctase activity, acting on single donors with incorporation of molecular oxygen		410	96358	234	65	OX5	
MF	GO:0050660	flavin adenine dinucleotide binding	5/210	85/18410	0.002885183	0.015167063	0.008804615	MAOB/NOS2/GSR/POR/NOS3	5
MF	GO:0004364	glutathione transferase activity	3/210	26/18410	0.003134754	0.016182106	0.009393856	GSTP1/GSTM1/GSTM2	3
MF	GO:0004435	phosphatidylinositol phospholipase C activity	3/210	26/18410	0.003134754	0.016182106	0.009393856	CHRM3/CHRM1/CHRM5	3
MF	GO:0008201	heparin binding	7/210	168/18410	0.003199404	0.01636838	0.00950199	APOB/VEGFA/CXCL8/MPO/CXCL11/CXCL10/PCOLCE	7
MF	GO:0015459	potassium channel regulator activity	4/210	54/18410	0.003332057	0.016704974	0.009697386	ADRB2/AKT1/CAV1/RASA1	4
MF	GO:0016651	oxidoreductase activity, acting on NAD(P)	5/210	88/18410	0.003352656	0.016704974	0.009697386	NCF1/AKR1C3/POR/DUOX2/NQO1	5

		H							
MF	GO:0051219	phosphoprotein binding	5/210	88/18410	0.003352656	0.016704974	0.009697386	MAPK3/MAPK1/RB1/PLAT/RASA1	5
MF	GO:0004190	aspartic-type endopeptidase activity	3/210	27/18410	0.003497221	0.017275064	0.010028328	CASP3/BACE2/CTSD	3
MF	GO:0016298	lipase activity	6/210	130/18410	0.003791702	0.018462459	0.010717621	CHRM3/CHRM1/CHRM5/HMOX1/PLB1/CES1	6
MF	GO:0030374	nuclear receptor coactivator activity	4/210	56/18410	0.003802042	0.018462459	0.010717621	NCOA2/NCOA1/ELK1/PRKCB	4
MF	GO:0004629	phospholipase C activity	3/210	28/18410	0.003884277	0.018547423	0.010766943	CHRM3/CHRM1/CHRM5	3
MF	GO:0070001	aspartic-type peptidase activity	3/210	28/18410	0.003884277	0.018547423	0.010766943	CASP3/BACE2/CTSD	3
MF	GO:0004693	cyclin-dependent protein serine/threonine kinase activity	3/210	29/18410	0.004296413	0.020179055	0.0117141 2	CDK2/CDK1/CDK4	3
MF	GO:0097472	cyclin-dependent protein kinase activity	3/210	29/18410	0.004296413	0.020179055	0.0117141 2	CDK2/CDK1/CDK4	3
MF	GO:0033764	steroid dehydrogenase activity, acting on the CH-OH	3/210	30/18410	0.004734093	0.022053943	0.0128025 1	HSD3B2/HSD3B1/AKR1C3	3

		group of donors, NAD or NADP as acceptor							
MF	GO:0004714	transmembrane receptor protein tyrosine kinase activity	4/210	60/18410	0.0048 7276	0.022516 868	0.0130712 41	KDR/INSRR/EGFR/ERBB2	4
MF	GO:0005164	tumor necrosis factor receptor binding	3/210	31/18410	0.0051 97754	0.023675 089	0.0137435 99	STAT1/CASP8/CD40LG	3
MF	GO:0070851	growth factor receptor binding	6/210	139/18410	0.0052 4987	0.023675 089	0.0137435 99	IL4/VEGFA/EGF/IL6R/IL1B/IL1A	6
MF	GO:0001094	TFIID-class transcription factor complex binding	2/210	10/18410	0.0054 86646	0.023675 089	0.0137435 99	AHR/TP53	2
MF	GO:0004955	prostaglandin receptor activity	2/210	10/18410	0.0054 86646	0.023675 089	0.0137435 99	PPARG/PTGER3	2
MF	GO:0016175	superoxide-generating NAD(P)H oxidase activity	2/210	10/18410	0.0054 86646	0.023675 089	0.0137435 99	NCF1/DUOX2	2
MF	GO:0016653	oxidoreductase activity, acting	2/210	10/18410	0.0054 86646	0.023675 089	0.0137435 99	POR/NQO1	2

		on NAD(P)H, heme protein as acceptor							
MF	GO:0018455	alcohol dehydrogenase [NAD(P)+] activity	2/210	10/18410	0.005486646	0.023675089	0.013743599	ADH1B/ADH1C	2
MF	GO:0043295	glutathione binding	2/210	10/18410	0.005486646	0.023675089	0.013743599	GSTM1/GSTM2	2
MF	GO:0005539	glycosaminoglycan binding	8/210	234/18410	0.005530326	0.023675089	0.013743599	TNFAIP6/APOB/VEGFA/CXCL8/MPO/CXCL11/CXCL10/PCOLCE	8
MF	GO:0015108	chloride transmembrane transporter activity	5/210	99/18410	0.005536583	0.023675089	0.013743599	GABRA1/SLC6A2/SLC6A4/SLC6A3/CLDN4	5
MF	GO:0043177	organic acid binding	6/210	141/18410	0.005622982	0.023866436	0.013854678	RXRA/NOS2/PPARG/PPARD/UGT1A1/NOS3	6
MF	GO:0043621	protein self-association	4/210	63/18410	0.005796983	0.024424055	0.014178381	ACHE/PPARG/TP53/HSF1	4
MF	GO:0016247	channel regulator activity	6/210	143/18410	0.006015127	0.025158158	0.014604534	ADRB2/AKT1/BCL2/CAV1/PRKCB/RASA1	6
MF	GO:0008237	metallopeptidase activity	7/210	189/18410	0.006066077	0.025187406	0.014621512	DPEP1/LTA4H/MMP1/MMP3/MMP2/MMP9/NPEPPS	7
MF	GO:0016248	channel inhibitor activity	3/210	33/18410	0.006204647	0.025394733	0.014741868	BCL2/CAV1/RASA1	3
MF	GO:0030291	protein serine/threonine	3/210	33/18410	0.006204647	0.025394733	0.014741868	CASP3/CDKN1A/HSPB1	3

		kinase inhibitor activity							
MF	GO:0001161	intronic transcription regulatory region sequence-specific DNA binding	2/210	11/18410	0.006655755	0.025768566	0.014958881	NCOA2/HSF1	2
MF	GO:0004954	prostanoid receptor activity	2/210	11/18410	0.006655755	0.025768566	0.014958881	PPARG/PTGER3	2
MF	GO:0035673	oligopeptide transmembrane transporter activity	2/210	11/18410	0.006655755	0.025768566	0.014958881	ABCC1/GJA1	2
MF	GO:0042166	acetylcholine binding	2/210	11/18410	0.006655755	0.025768566	0.014958881	CHRM3/ACHE	2
MF	GO:0048407	platelet-derived growth factor binding	2/210	11/18410	0.006655755	0.025768566	0.014958881	COL1A1/COL3A1	2
MF	GO:0097371	MDM2/MDM4 family protein binding	2/210	11/18410	0.006655755	0.025768566	0.014958881	PPARA/TP53	2
MF	GO:1900750	oligopeptide binding	2/210	11/18410	0.006655755	0.025768566	0.014958881	GSTM1/GSTM2	2
MF	GO:1990459	transferrin receptor binding	2/210	11/18410	0.006655755	0.025768566	0.014958881	IKBKB/CHUK	2

MF	GO:0016229	steroid dehydrogenase activity	3/210	34/18410	0.006748632	0.025952792	0.015065826	HSD3B2/HSD3B1/AKR1C3	3
MF	GO:0047485	protein N-terminus binding	5/210	105/18410	0.007077563	0.027036292	0.015694807	NCOA1/SLC6A3/RELA/TP53/PARP1	5
MF	GO:0016829	lyase activity	7/210	195/18410	0.007158222	0.02716332	0.015768548	CYP1A2/CYP1A1/CYP1B1/FASN/GOT1/CA2/ODC1	7
MF	GO:0052689	carboxylic ester hydrolase activity	6/210	149/18410	0.007311206	0.027210921	0.015796181	ACHE/TNFAIP6/PLB1/CES1/CA2/PON1	6
MF	GO:0042887	amide transmembrane transporter activity	3/210	35/18410	0.007320105	0.027210921	0.015796181	ABCC1/GJA1/ABCG2	3
MF	GO:0097718	disordered domain specific binding	3/210	35/18410	0.007320105	0.027210921	0.015796181	HSP90AB1/RB1/TP53	3
MF	GO:0004620	phospholipase activity	5/210	106/18410	0.00736072	0.027210921	0.015796181	CHRM3/CHRM1/CHRM5/HMOX1/PLB1	5
MF	GO:0005506	iron ion binding	6/210	151/18410	0.007784922	0.028213084	0.016377945	CYP3A4/CYP1A2/CYP1A1/CYP1B1/ALOX5/CYP19A1	6
MF	GO:0015103	inorganic anion transmembrane transporter activity	6/210	151/18410	0.007784922	0.028213084	0.016377945	GABRA1/SLC6A2/SLC6A4/SLC6A3/ABCC1/CLDN4	6
MF	GO:0004032	alditol:NADP+ 1-oxidoreductase	2/210	12/18410	0.007927237	0.028213084	0.016377945	AKR1B1/AKR1C3	2

		activity							
MF	GO:0004861	cyclin-dependent protein serine/threonine kinase inhibitor activity	2/210	12/18410	0.007927237	0.028213084	0.016377945	CASP3/CDKN1A	2
MF	GO:0051378	serotonin binding	2/210	12/18410	0.007927237	0.028213084	0.016377945	HTR3A/SLC6A4	2
MF	GO:0097677	STAT family protein binding	2/210	12/18410	0.007927237	0.028213084	0.016377945	PPARG/HSF1	2
MF	GO:0004177	aminopeptidase activity	3/210	39/18410	0.009886923	0.034543946	0.020053067	DPP4/LTA4H/NPEPPS	3
MF	GO:0019825	oxygen binding	3/210	39/18410	0.009886923	0.034543946	0.020053067	CYP3A4/CYP1A1/CYP19A1	3
MF	GO:0030331	nuclear estrogen receptor binding	3/210	39/18410	0.009886923	0.034543946	0.020053067	NCOA1/ESR1/PARP1	3
MF	GO:0001098	basal transcription machinery binding	4/210	74/18410	0.010166129	0.035091517	0.020370936	AR/ESR1/AHR/TP53	4
MF	GO:0001099	basal RNA polymerase II transcription machinery binding	4/210	74/18410	0.010166129	0.035091517	0.020370936	AR/ESR1/AHR/TP53	4
MF	GO:0043028	cysteine-type endopeptidase	3/210	40/18410	0.010600183	0.036292807	0.021068296	DPEP1/BAD/BIRC5	3

		regulator activity involved in apoptotic process							
MF	GO:0008559	ABC-type xenobiotic transporter activity	2/210	14/18410	0.010767499	0.036292807	0.021068296	ABCC1/ABCG2	2
MF	GO:0016863	intramolecular oxidoreductase activity, transposing C=C bonds	2/210	14/18410	0.010767499	0.036292807	0.021068296	HSD3B2/HSD3B1	2
MF	GO:0052650	NADP-retinol dehydrogenase activity	2/210	14/18410	0.010767499	0.036292807	0.021068296	AKR1B1/AKR1C3	2
MF	GO:0004953	icosanoid receptor activity	2/210	15/18410	0.012331478	0.040376782	0.02343908	PPARG/PTGER3	2
MF	GO:0005041	low-density lipoprotein particle receptor activity	2/210	15/18410	0.012331478	0.040376782	0.02343908	OLR1/LDLR	2
MF	GO:0036041	long-chain fatty acid binding	2/210	15/18410	0.012331478	0.040376782	0.02343908	PPARG/PPARD	2
MF	GO:00506	oxidored	2/210	15/18	0.0123	0.040376	0.0234390	NCF1/DUOX2	2

	64	uctase activity, acting on NAD(P)H, oxygen as acceptor		410	31478	782	8		
MF	GO:1904680	peptide transmembrane transporter activity	2/210	15/18410	0.012331478	0.040376782	0.02343908	ABCC1/GJA1	2
MF	GO:0019199	transmembrane receptor protein kinase activity	4/210	79/18410	0.012706206	0.041343066	0.024000016	KDR/INSRR/EGFR/ERBB2	4
MF	GO:0016504	peptidase activator activity	3/210	43/18410	0.012915198	0.041343066	0.024000016	BAD/CAV1/PCOLCE	3
MF	GO:0044183	protein folding chaperone	3/210	43/18410	0.012915198	0.041343066	0.024000016	HSP90AB1/HSPA5/HSPB1	3
MF	GO:0048156	tau protein binding	3/210	43/18410	0.012915198	0.041343066	0.024000016	HSP90AB1/GSK3B/MAP2	3
MF	GO:0001784	phosphotyrosine residue binding	3/210	44/18410	0.01374587	0.04351593	0.025261383	MAPK3/MAPK1/RASA1	3
MF	GO:0030544	Hsp70 protein binding	3/210	44/18410	0.01374587	0.04351593	0.025261383	BAX/CDK1/CYP1A1	3
MF	GO:0001540	amyloid-beta binding	4/210	81/18410	0.013825768	0.043528379	0.02526861	ACHE/ADRB2/GRIA2/LDLR	4
MF	GO:0004698	calcium-depende	2/210	16/18410	0.013988221	0.043561145	0.025287631	PRKCA/PRKCB	2

		nt protein kinase C activity							
MF	GO:0016641	oxidoreductase activity, acting on the CH-NH2 group of donors, oxygen as acceptor	2/210	16/18410	0.013988221	0.043561145	0.025287631	MAOB/VCAM1	2
MF	GO:0004697	protein kinase C activity	2/210	17/18410	0.015735431	0.048216052	0.027989846	PRKCA/PRKCB	2
MF	GO:0005149	interleukin-1 receptor binding	2/210	17/18410	0.015735431	0.048216052	0.027989846	IL1B/IL1A	2
MF	GO:0042165	neurotransmitter binding	2/210	17/18410	0.015735431	0.048216052	0.027989846	CHRM3/ACHE	2
MF	GO:0061134	peptidase regulator activity	7/210	230/18410	0.016625565	0.050672599	0.029415893	DPEP1/SLPI/BAD/CAV1/BIRC5/SERPINE1/PCOLCE	7
MF	GO:0008013	beta-catenin binding	4/210	86/18410	0.016892785	0.051214633	0.029730549	AR/ESR1/GSK3B/GJA1	4
MF	GO:0030228	lipoprotein particle receptor activity	2/210	18/18410	0.017570847	0.052712542	0.030600098	OLR1/LDLR	2
MF	GO:0047834	D-threo-aldose 1-dehydrogenase activity	2/210	18/18410	0.017570847	0.052712542	0.030600098	AKR1B1/AKR1C3	2

MF	GO:0008081	phosphoric diester hydrolase activity	4/210	88/18410	0.018229314	0.05440311	0.031581488	CHRM3/CHRM1/CHRM5/HMOX1	4
MF	GO:0099529	neurotransmitter receptor activity involved in regulation of postsynaptic membrane potential	3/210	49/18410	0.018347644	0.054472538	0.031621791	CHRM1/GABRA1/ADRB1	3
MF	GO:0015485	cholesterol binding	3/210	50/18410	0.019358312	0.05717687	0.0331916 8	SOAT2/SOAT1/CAV1	3
MF	GO:0099106	ion channel regulator activity	5/210	138/18410	0.021077475	0.06128381	0.035575795	ADRB2/AKT1/CAV1/PRKCB/RASA1	5
MF	GO:1901618	organic hydroxy compound transmembrane transporter activity	3/210	52/18410	0.021470403	0.06128381	0.035575795	SLC6A2/SLC6A4/SLC6A3	3
MF	GO:1901682	sulfur compound transmembrane transporter activity	3/210	52/18410	0.021470403	0.06128381	0.035575795	ABCC1/GJA1/ABCG2	3

MF	GO:0001972	retinoic acid binding	2/210	20/18410	0.021497462	0.06128381	0.035575795	RXRA/UGT1A1	2
MF	GO:0016500	protein-hormone receptor activity	2/210	20/18410	0.021497462	0.06128381	0.035575795	INSRR/ADIPOR2	2
MF	GO:0016638	oxidoreductase activity, acting on the CH-NH2 group of donors	2/210	20/18410	0.021497462	0.06128381	0.035575795	MAOB/VCAM1	2
MF	GO:0030296	protein tyrosine kinase activator activity	2/210	20/18410	0.021497462	0.06128381	0.035575795	EGF/ERBB3	2
MF	GO:0051213	dioxygenase activity	4/210	93/18410	0.021852177	0.061681269	0.035806524	PTGS2/PTGS1/ALOX5/POR	4
MF	GO:0072341	modified amino acid binding	4/210	93/18410	0.021852177	0.061681269	0.035806524	DPEP1/GSTM1/GSTM2/FASN	4
MF	GO:0008374	O-acyltransferase activity	3/210	53/18410	0.02257187	0.063091128	0.036624959	SOAT2/SOAT1/DGAT2	3
MF	GO:0043539	protein serine/threonine kinase activator activity	3/210	53/18410	0.02257187	0.063091128	0.036624959	CCNB1/IGF2/CD40LG	3
MF	GO:0005123	death receptor binding	2/210	21/18410	0.023584336	0.065601091	0.038082015	CASP3/CASP8	2
MF	GO:0005080	protein kinase C binding	3/210	55/18410	0.024865652	0.067375301	0.039111959	PRKCB/HSPB1/TOP2A	3

MF	GO:0030170	pyridoxal phosphate binding	3/210	55/18410	0.024865652	0.067375301	0.039111959	PYGM/GOT1/ABAT	3
MF	GO:0045309	protein phosphorylated amino acid binding	3/210	55/18410	0.024865652	0.067375301	0.039111959	MAPK3/MAPK1/RASA1	3
MF	GO:0050840	extracellular matrix binding	3/210	55/18410	0.024865652	0.067375301	0.039111959	ACHE/VEGFA/SPP1	3
MF	GO:0015267	channel activity	11/210	489/18410	0.025026864	0.067375301	0.039111959	SCN5A/OPRM1/GABRA1/GRIA2/HTR3A/ATP5F1B/BCL2/BAX/KCNH2/GJA1/CLDN4	11
MF	GO:0004896	cytokine receptor activity	4/210	97/18410	0.025045768	0.067375301	0.039111959	IL10RA/IL6R/F3/IL2RA	4
MF	GO:0022803	passive transmembrane transporter activity	11/210	490/18410	0.025354371	0.067375301	0.039111959	SCN5A/OPRM1/GABRA1/GRIA2/HTR3A/ATP5F1B/BCL2/BAX/KCNH2/GJA1/CLDN4	11
MF	GO:0000062	fatty-acyl-CoA binding	2/210	22/18410	0.025750769	0.067375301	0.039111959	SOAT2/SOAT1	2
MF	GO:0004602	glutathione peroxidase activity	2/210	22/18410	0.025750769	0.067375301	0.039111959	GSTP1/GSTM2	2
MF	GO:0009931	calcium-dependent protein serine/threonine kinase	2/210	22/18410	0.025750769	0.067375301	0.039111959	PRKCA/PRKCB	2

		activity							
MF	GO:0043027	cysteine-type endopeptidase inhibitor activity involved in apoptotic process	2/210	22/18410	0.025750769	0.067375301	0.039111959	DPEP1/BIRC5	2
MF	GO:0072349	modified amino acid transmembrane transporter activity	2/210	22/18410	0.025750769	0.067375301	0.039111959	ABCC1/GJA1	2
MF	GO:0120020	cholesterol transfer activity	2/210	22/18410	0.025750769	0.067375301	0.039111959	MTTP/APOB	2
MF	GO:0070279	vitamin B6 binding	3/210	56/18410	0.026057932	0.067869069	0.039398596	PYGM/GOT1/ABAT	3
MF	GO:0005516	calmodulin binding	6/210	200/18410	0.027401669	0.070665018	0.041021669	SCN5A/NOS2/AKT1/PPP3CA/NOS3/MAP2	6
MF	GO:0008106	alcohol dehydrogenase (NADP+) activity	2/210	23/18410	0.027994693	0.070665018	0.041021669	AKR1B1/AKR1C3	2
MF	GO:0008483	transaminase activity	2/210	23/18410	0.027994693	0.070665018	0.041021669	GOT1/ABAT	2
MF	GO:0010857	calcium-dependent protein kinase	2/210	23/18410	0.027994693	0.070665018	0.041021669	PRKCA/PRKCB	2

		activity							
MF	GO:0035035	histone acetyltransferase binding	2/210	23/18410	0.027994693	0.070665018	0.041021669	STAT1/TP53	2
MF	GO:0120015	sterol transfer activity	2/210	23/18410	0.027994693	0.070665018	0.041021669	MTTP/APOB	2
MF	GO:1901567	fatty acid derivative binding	2/210	23/18410	0.027994693	0.070665018	0.041021669	SOAT2/SOAT1	2
MF	GO:0008509	anion transmembrane transporter activity	8/210	315/18410	0.028464569	0.071535955	0.041527256	GABRA1/SLC6A2/SLC6A4/SLC6A3/ABCC1/GJA1/ABCG2/CLDN4	8
MF	GO:0008238	exopeptidase activity	4/210	102/18410	0.029414247	0.073599842	0.042725361	DPP4/DPEP1/LTA4H/NPEPPS	4
MF	GO:0016765	transferase activity, transferring alkyl or aryl (other than methyl) groups	3/210	59/18410	0.029815846	0.073958787	0.042933731	GSTP1/GSTM1/GSTM2	3
MF	GO:0140678	molecular function inhibitor activity	3/210	59/18410	0.029815846	0.073958787	0.042933731	BCL2/CAV1/RASA1	3
MF	GO:0016769	transferase activity, transferring nitrogenous	2/210	24/18410	0.030314075	0.074870539	0.043463011	GOT1/ABAT	2

		groups							
MF	GO:0022824	transmitter-gated ion channel activity	3/210	60/18410	0.03112864	0.075900897	0.044061143	GABRA1/GRIA2/HTR3A	3
MF	GO:0022835	transmitter-gated channel activity	3/210	60/18410	0.03112864	0.075900897	0.044061143	GABRA1/GRIA2/HTR3A	3
MF	GO:0032934	sterol binding	3/210	60/18410	0.03112864	0.075900897	0.044061143	SOAT2/SOAT1/CAV1	3
MF	GO:0001227	DNA-binding transcription repressor activity, RNA polymerase II-specific	8/210	321/18410	0.031330447	0.076069262	0.044158881	PPARG/PPARD/RELA/JUN/PPARA/TP53/MYC/HSF1	8
MF	GO:0005507	copper ion binding	3/210	61/18410	0.032471383	0.078414499	0.045520312	SOD1/TP53/IL1A	3
MF	GO:0017025	TBP-class protein binding	2/210	25/18410	0.03270692	0.078414499	0.045520312	ESR1/AHR	2
MF	GO:0120227	acyl-CoA binding	2/210	25/18410	0.03270692	0.078414499	0.045520312	SOAT2/SOAT1	2
MF	GO:0001217	DNA-binding transcription repressor activity	8/210	325/18410	0.033347492	0.079617136	0.046218453	PPARG/PPARD/RELA/JUN/PPARA/TP53/MYC/HSF1	8
MF	GO:1990841	promoter-specific	3/210	62/18410	0.033843981	0.080467225	0.046711937	STAT1/TP53/HSF1	3

		chromatin binding							
MF	GO:0004709	MAP kinase kinase kinase activity	2/210	26/18410	0.035171267	0.083277422	0.048343282	EGFR/RAF1	2

S2:Kegg pathway analysis results									
Class	ID	Pathway	GeneRatio	BgRatio	pvalue	p.adjust	qvalue	geneID	Count
Kegg Pathway	hsa05417	Lipid and atherosclerosis	47/194	215/8163	4.17E-33	1.15E-30	4.39E-31	RXRA/HSP90AB1/PPARG/MAPK14/GSK3B/MAPK10/RELA/NCF1/OLR1/JUN/IKBKB/AKT1/BCL2/BAX/CASP3/MAPK8/MMP1/CYP1A1/ICAM1/SELE/VCAM1/PPP3CA/MAPK3/MAPK1/LDLR/BAD/APOB/RXRB/STAT3/MMP3/BCL2L1/FOS/CASP9/M	47

								MP9/TP53/NFKBIA/CASP8/PRKCA/HSPA5/IL1B/CCL2/CXCL8/NOS3/NFE2L2/CXCL2/CHUK/CD40LG	
Kegg Pathway	hsa04933	AGE-RAGE signaling pathway in diabetic complications	32/194	100/8163	2.87E-28	3.95E-26	1.51E-26	MAPK14/MAPK10/RELA/JUN/AKT1/BCL2/BAX/CASP3/MAPK8/STAT1/ICAM1/SELE/VCAM1/MAPK3/MAPK1/STAT3/CCND1/CDK4/VEGFA/MMP2/PRKCA/F3/IL1B/CCL2/CXCL8/PRKCB/NOS3/THBD/SERPINE1/COL1A1/IL1A/COL3A1	32
Kegg Pathway	hsa05215	Prostate cancer	30/194	97/8163	4.97E-26	4.56E-24	1.75E-24	AR/HSP90AB1/GSK3B/CDK2/RELA/IKBKB/AKT1/BCL2/GSTP1/INSRR/MAPK3/MAPK1/BAD/CCND1/RB1/MMP3/EGFR/CDKN1A/CASP9/PLAU/MMP9/EGF/TP53/NFKBIA/RAF1/ERBB2/PLAT/CHUK/E2F1/E2F2	30
Kegg Pathway	hsa05418	Fluid shear stress and atherosclerosis	34/194	139/8163	1.18E-25	8.09E-24	3.10E-24	HSP90AB1/KDR/MAPK14/MAPK10/RELA/NCF1/JUN/IKBKB/AKT1/BCL2/MAPK8/HMOX1/ICAM1/SELE/VCAM1/GSTP1/GSTM1/GSTM2/VEGFA/FOS/MMP2/MMP9/TP53/CAV1/IL1B/CCL2/NOS3/PLAT/THBD/IFNG/IL1A/NFE2L2/NQO1/CHUK	34
Kegg Pathway	hsa05207	Chemical carcinogenesis - receptor	39/194	212/8163	1.97E-24	9.18E-23	3.51E-23	PGR/AR/RXRA/ADRB2/HSP90AB1/ESR1/ESR2/RELA/ADRB1/JUN/AKT1/BCL2/CYP3A4/CYP1A2/CYP1A1/CYP1B1/AHR/NR1I3/GSTM1/GSTM2/MAPK3/M	39

		activation						APK1/BAD/UGT1A1/PPARA/RXRB/STAT3/CCND1/RB1/EGFR/VEGFA/FOS/EGF/RAF1/PRKCA/MYC/PRKCB/BIRC5/E2F1	
Kegg Pathway	hsa05161	Hepatitis B	35/194	162/8163	2.00E-24	9.18E-23	3.51E-23	MAPK14/CCNA2/CDK2/MAPK10/RELA/JUN/IKBKB/AKT1/BCL2/BAX/CASP3/MAPK8/STAT1/MAPK3/MAPK1/BAD/STAT3/RB1/FOS/CDKN1A/CASP9/MMP9/TP53/ELK1/NFKBIA/CASP8/RAF1/PRKCA/MYC/CXCL8/PRKCB/BIRC5/CHUK/E2F1/E2F2	35
Kegg Pathway	hsa05212	Pancreatic cancer	26/194	76/8163	6.03E-24	2.37E-22	9.06E-23	MAPK10/RELA/IKBKB/AKT1/BAX/MAPK8/STAT1/MAPK3/MAPK1/BAD/STAT3/CCND1/RB1/CDK4/EGFR/VEGFA/BCL2L1/CDKN1A/CASP9/EGF/TP53/RAF1/ERBB2/CHUK/E2F1/E2F2	26
Kegg Pathway	hsa05160	Hepatitis C	33/194	157/8163	1.21E-22	4.15E-21	1.59E-21	RXRA/GSK3B/CDK2/RELA/IKBKB/AKT1/BAX/CASP3/STAT1/MAPK3/MAPK1/LDLR/BAD/PPARA/STAT3/CCND1/RB1/CDK4/EGFR/CDKN1A/CASP9/EGF/TP53/NFKBIA/CASP8/RAF1/MYC/IFNG/CLDN4/CXCL10/CHUK/E2F1/E2F2	33
Kegg Pathway	hsa05219	Bladder cancer	20/194	41/8163	2.14E-22	6.55E-21	2.51E-21	MMP1/MAPK3/MAPK1/CCND1/RB1/CDK4/EGFR/VEGFA/CDKN1A/MMP2/MMP9/EGF/TP53/RAF1/ERBB2/MYC/CXCL8/RASSF1/E2F1/	20

								E2F2	
Kegg Path way	hsa 051 67	Kaposi sarcoma-associated herpesvirus infection	35/19 4	194/8 163	1.19E-21	3.26E-20	1.25E-20	PTGS2/MAPK14/GSK3B/MAPK10/RELA/JUN/IKBKB/AKT1/BAX/CASP3/MAPK8/STAT1/ICAM1/PPP3CA/MAPK3/MAPK1/STAT3/CCND1/RB1/CDK4/VEGFA/FOS/CDKN1A/CASP9/TP53/NFKBIA/CASP8/RAF1/HIF1A/MYC/CXCL8/CXCL2/CHUK/E2F1/E2F2	35
Kegg Path way	hsa 046 57	IL-17 signaling pathway	26/19 4	94/81 63	2.96E-21	7.41E-20	2.84E-20	PTGS2/HSP90AB1/MAPK14/GSK3B/MAPK10/RELA/JUN/IL4/IKBKB/CASP3/MAPK8/MMP1/MAPK3/MAPK1/MMP3/FOS/MMP9/NFKBIA/CASP8/IL1B/CCL2/CXCL8/IFNG/CXCL2/CXCL10/CHUK	26
Kegg Path way	hsa 052 23	Non-small cell lung cancer	23/19 4	72/81 63	1.69E-20	3.87E-19	1.48E-19	RXRA/AKT1/BAX/MAPK3/MAPK1/BAD/RXRB/STAT3/CCND1/RB1/CDK4/EGFR/CDKN1A/CASP9/EGF/TP53/RAF1/PRKCA/ERBB2/PRKCB/RASSF1/E2F1/E2F2	23
Kegg Path way	hsa 051 63	Human cytomegalovirus infection	36/19 4	225/8 163	2.00E-20	4.24E-19	1.62E-19	PTGS2/MAPK14/GSK3B/RELA/IKBKB/AKT1/BAX/CASP3/PPP3CA/MAPK3/MAPK1/STAT3/CCND1/RB1/CDK4/EGFR/VEGFA/CDKN1A/CASP9/IL10RA/IL6R/TP53/ELK1/NFKBIA/CASP8/RAF1/PRKCA/MYC/IL1B/CCL2/PTGER3/CXCL8/PRKCB/CHUK/E2F1/E2F2	36
Kegg Path	hsa 015	Endocrine	25/19 4	98/81 63	1.60E-19	3.15E-18	1.21E-18	ESR1/MAPK14/ESR2/MAPK10/JUN/AKT1/B	25

way	22	resistance						CL2/BAX/MAPK8/MAPK3/MAPK1/BAD/CCND1/RB1/CDK4/EGFR/FOS/CDKN1A/MMP2/MMP9/TP53/RAF1/ERBB2/E2F1/E2F2	
Kegg Pathway	hsa04668	TNF signaling pathway	25/194	112/8163	5.36E-18	9.82E-17	3.76E-17	PTGS2/MAPK14/MAPK10/RELA/JUN/IKBKB/AKT1/CASP3/MAPK8/ICAM1/SELE/VCAM1/MAPK3/MAPK1/MMP3/FOS/MMP9/NFKBIA/CASP8/IL1B/CCL2/CXCL2/CXCL10/CHUK/IRF1	25
Kegg Pathway	hsa05222	Small cell lung cancer	23/194	92/8163	8.10E-18	1.39E-16	5.33E-17	PTGS2/RXRA/NOS2/CDK2/RELA/IKBKB/AKT1/BCL2/BAX/CASP3/RXRB/CCND1/RB1/CDK4/BCL2L1/CDKN1A/CASP9/TP53/NFKBIA/MYC/CHUK/E2F1/E2F2	23
Kegg Pathway	hsa04659	Th17 cell differentiation	24/194	108/8163	2.89E-17	4.67E-16	1.79E-16	RXRA/HSP90AB1/MAPK14/MAPK10/RELA/JUN/IL4/IKBKB/MAPK8/STAT1/AHR/PPP3CA/MAPK3/MAPK1/RXRB/STAT3/FOS/IL6R/NFKBIA/HIF1A/IL1B/IL2RA/IFNG/CHUK	24
Kegg Pathway	hsa05145	Toxoplasmosis	24/194	112/8163	7.09E-17	1.08E-15	4.15E-16	NOS2/MAPK14/MAPK10/RELA/IKBKB/AKT1/BCL2/CASP3/MAPK8/STAT1/ALOX5/MAPK3/MAPK1/LDLR/BAD/STAT3/BCL2L1/CASP9/IL10RA/NFKBIA/CASP8/IFNG/CHUK/CD40LG	24
Kegg Pathway	hsa05208	Chemical carcinogen	32/194	223/8163	9.07E-17	1.31E-15	5.03E-16	MAPK14/MAPK10/RELA/NCF1/JUN/ATP5F1B/IKBKB/AKT1/MAPK8/HMOX1/CYP1A2/CY	32

		esis-reactive oxygen species						P1A1/CYP1B1/AHR/GSTM1/GSTM2/AKR1C3/MAPK3/MAPK1/BAD/SOD1/CAT/EGFR/VEGFA/FOS/EGF/NFKBIA/RAF1/HIF1A/NFE2L2/NQO1/CHUK	
Kegg Pathway	hsa05162	Measles	26/194	139/8163	1.17E-16	1.61E-15	6.16E-16	GSK3B/CDK2/MAPK10/RELA/JUN/IKBKB/AKT1/BCL2/BAX/CASP3/MAPK8/STAT1/BAD/STAT3/CCND1/CDK4/BCL2L1/FOS/CASP9/TP53/NFKBIA/CASP8/IL1B/IL2RA/IL1A/CHUK	26
Kegg Pathway	hsa05210	Colorectal cancer	21/194	86/8163	3.97E-16	5.19E-15	1.99E-15	GSK3B/MAPK10/JUN/AKT1/BCL2/BAX/CASP3/MAPK8/MAPK3/MAPK1/BAD/CCND1/EGFR/FOS/CDKN1A/CASP9/EGF/TP53/RAF1/MYC/BIRC5	21
Kegg Pathway	hsa05225	Hepatocellular carcinoma	27/194	168/8163	1.59E-15	1.98E-14	7.59E-15	GSK3B/AKT1/BAX/HMOX1/GSTP1/GSTM1/GSTM2/MAPK3/MAPK1/BAD/CCND1/RB1/CDK4/EGFR/BCL2L1/CDKN1A/TP53/ELK1/RAF1/PRKCA/MYC/PRKCB/NFE2L2/NQO1/E2F1/E2F2/IGF2	27
Kegg Pathway	hsa05169	Epstein-Barr virus infection	29/194	202/8163	2.87E-15	3.44E-14	1.32E-14	MAPK14/CCNA2/CDK2/MAPK10/RELA/JUN/IKBKB/AKT1/BCL2/BAX/CASP3/MAPK8/STAT1/ICAM1/PSMD3/STAT3/CCND1/RB1/CDK4/CDKN1A/CASP9/TP53/NFKBIA/CASP8/MYC/CXCL10/CHUK/E2F1/E2F2	29
Kegg Pathway	hsa052	Chronic	19/194	76/8163	6.73E-15	7.71E-14	2.95E-14	RELA/IKBKB/AKT1/BAX/MAPK3/MAPK1/BA	19

way	20	myeloid leukemia						D/CCND1/RB1/CDK4/BCL2L1/CDKN1A/TP53/NFKBIA/RAF1/MYC/CHUK/E2F1/E2F2	
Kegg Pathway	hsa01521	EGFR tyrosine kinase inhibitor resistance	19/194	79/8163	1.46E-14	1.61E-13	6.15E-14	KDR/GSK3B/AKT1/BCL2/BAX/MAPK3/MAPK1/BAD/STAT3/EGFR/VEGFA/BCL2L1/EGF/IL6R/RAF1/PRKCA/ERBB2/PRKCB/ERBB3	19
Kegg Pathway	hsa04218	Cellular senescence	25/194	156/8163	2.06E-14	2.18E-13	8.34E-14	MAPK14/CHEK1/CCNA2/CDK2/RELA/AKT1/CDK1/PPP3CA/MAPK3/MAPK1/CCND1/RB1/CDK4/CDKN1A/TP53/RAF1/MYC/CXCL8/CCNB1/SERPINE1/IL1A/CHEK2/E2F1/E2F2/IGFBP3	25
Kegg Pathway	hsa05166	Human T-cell leukemia virus 1 infection	29/194	222/8163	3.62E-14	3.69E-13	1.41E-13	CHEK1/CCNA2/CDK2/MAPK10/RELA/JUN/IKBKB/AKT1/BAX/MAPK8/ICAM1/PPP3CA/MAPK3/MAPK1/CCND1/RB1/CDK4/BCL2L1/FOS/CDKN1A/TP53/ELK1/NFKBIA/MYC/IL2RA/CHEK2/CHUK/E2F1/E2F2	29
Kegg Pathway	hsa01524	Platinum drug resistance	18/194	73/8163	4.65E-14	4.57E-13	1.75E-13	AKT1/BCL2/BAX/CASP3/GSTP1/GSTM1/GSTM2/MAPK3/MAPK1/BAD/BCL2L1/CDKN1A/CASP9/TP53/CASP8/ERBB2/BIRC5/TOP2A	18
Kegg Pathway	hsa04151	PI3K-Akt signaling pathway	36/194	354/8163	5.94E-14	5.64E-13	2.16E-13	CHRM1/RXRA/CHRM2/HSP90AB1/KDR/GSK3B/CDK2/RELA/IL4/IKBKB/AKT1/BCL2/MAPK3/MAPK1/BAD/CCND1/CDK4/EGFR/VEGF	36

								A/BCL2L1/CDKN1A/CASP9/EGF/IL6R/TP53/RAF1/PRKCA/ERBB2/MYC/NOS3/IL2RA/COL1A1/CHUK/SPP1/IGF2/ERBB3	
Kegg Pathway	hsa04066	HIF-1 signaling pathway	21/194	109/8163	6.54E-14	6.00E-13	2.30E-13	NOS2/RELA/AKT1/BCL2/HMOX1/MAPK3/MAPK1/STAT3/EGFR/VEGFA/CDKN1A/EGF/IL6R/PRKCA/HIF1A/ERBB2/PRKCB/NOS3/SERPINE1/IFNG/HK2	21
Kegg Pathway	hsa04210	Apoptosis	23/194	136/8163	7.42E-14	6.58E-13	2.52E-13	MAPK10/RELA/JUN/IKBKB/AKT1/BCL2/BAX/CASP3/MAPK8/MAPK3/MAPK1/BAD/BCL2L1/FOS/CASP9/TP53/NFKBIA/CASP8/RAF1/BIRC5/PARP1/CHUK/CTSD	23
Kegg Pathway	hsa05213	Endometrial cancer	16/194	58/8163	1.86E-13	1.60E-12	6.11E-13	GSK3B/AKT1/BAX/MAPK3/MAPK1/BAD/CCND1/EGFR/CDKN1A/CASP9/EGF/TP53/ELK1/RAF1/ERBB2/MYC	16
Kegg Pathway	hsa05205	Proteoglycans in cancer	27/194	205/8163	2.45E-13	2.04E-12	7.82E-13	ESR1/KDR/MAPK14/AKT1/CASP3/MAPK3/MAPK1/STAT3/CCND1/EGFR/VEGFA/CDKN1A/PLAU/MMP2/MMP9/TP53/ELK1/RAF1/PRKCA/HIF1A/ERBB2/CAV1/MYC/PRKCB/COL1A1/IGF2/ERBB3	27
Kegg Pathway	hsa05224	Breast cancer	23/194	147/8163	4.14E-13	3.35E-12	1.28E-12	PGR/NCOA1/ESR1/GSK3B/ESR2/JUN/AKT1/BAX/MAPK3/MAPK1/CCND1/RB1/CDK4/EGFR/FOS/CDKN1A/EGF/TP53/RAF1/ERBB2/MYC/E2F1/E2F2	23
Kegg Path	hsa041	p53 signa	17/194	73/8163	6.72E-13	5.28E-12	2.02E-12	CHEK1/CDK2/BCL2/BAX/CASP3/CDK1/CCN	17

way	15	ling pathway						D1/CDK4/BCL2L1/CDKN1A/CASP9/TP53/CASP8/CCNB1/SERPINE1/CHEK2/IGFBP3	
Kegg Pathway	hsa04012	ErbB signaling pathway	18/194	85/8163	7.95E-13	6.08E-12	2.33E-12	GSK3B/MAPK10/JUN/AKT1/MAPK8/MAPK3/MAPK1/BAD/EGFR/CDKN1A/EGF/ELK1/RAF1/PRKCA/ERBB2/MYC/PRKCB/ERBB3	18
Kegg Pathway	hsa05164	Influenza A	24/194	171/8163	1.43E-12	1.06E-11	4.07E-12	PRSS1/RELA/IKBKB/AKT1/BAX/CASP3/STAT1/ICAM1/MAPK3/MAPK1/CDK4/CASP9/NFKBIA/CASP8/RAF1/PRKCA/IL1B/CCL2/CXCL8/PRKCB/IFNG/IL1A/CXCL10/CHUK	24
Kegg Pathway	hsa04936	Alcoholic liver disease	22/194	142/8163	1.70E-12	1.21E-11	4.63E-12	ADH1B/ADH1C/MAPK14/GSK3B/MAPK10/RELA/IKBKB/AKT1/CASP3/MAPK8/FASN/PPARA/SREBF1/ADIPOR2/CCND1/NFKBIA/CASP8/ACACA/IL1B/CXCL8/CXCL2/CHUK	22
Kegg Pathway	hsa05140	Leishmaniasis	17/194	77/8163	1.72E-12	1.21E-11	4.63E-12	PTGS2/NOS2/MAPK14/RELA/NCF1/JUN/IL4/STAT1/MAPK3/MAPK1/FOS/ELK1/NFKBIA/IL1B/PRKCB/IFNG/IL1A	17
Kegg Pathway	hsa05235	PD-L1 expression and PD-1 checkpoint pathway in cancer	18/194	89/8163	1.84E-12	1.26E-11	4.84E-12	MAPK14/RELA/JUN/IKBKB/AKT1/STAT1/PPP3CA/MAPK3/MAPK1/STAT3/EGFR/FOS/EGF/NFKBIA/RAF1/HIF1A/IFNG/CHUK	18
Kegg	hsa	Chaga	19/19	102/8	2.06E-12	1.37E-11	5.24E-12	NOS2/MAPK14/MAPK1	19

Path way	05142	s disease	4	163				0/RELA/JUN/IKBKB/AKT1/MAPK8/MAPK3/MAPK1/FOS/NFKBIA/CASP8/IL1B/CCL2/CXCL8/SERPINE1/IFNG/CHUK	
Kegg Path way	hsa04926	Relaxin signaling pathway	21/194	129/8163	2.09E-12	1.37E-11	5.24E-12	NOS2/MAPK14/MAPK10/RELA/JUN/AKT1/MAPK8/MMP1/MAPK3/MAPK1/EGFR/VEGFA/FOS/MMP2/MMP9/NFKBIA/RAF1/PRKCA/NOS3/COL1A1/COL3A1	21
Kegg Path way	hsa04620	Toll-like receptor signaling pathway	19/194	104/8163	2.96E-12	1.85E-11	7.08E-12	MAPK14/MAPK10/RELA/JUN/IKBKB/AKT1/MAPK8/STAT1/MAPK3/MAPK1/FOS/NFKBIA/CASP8/IL1B/CXCL8/CXCL11/CXCL10/CHUK/SPP1	19
Kegg Path way	hsa04660	T cell receptor signaling pathway	19/194	104/8163	2.96E-12	1.85E-11	7.08E-12	MAPK14/GSK3B/MAPK10/RELA/JUN/IL4/IKBKB/AKT1/MAPK8/PPP3CA/MAPK3/MAPK1/CDK4/FOS/NFKBIA/RAF1/IFNG/CHUK/CD40LG	19
Kegg Path way	hsa05170	Human immunodeficiency virus 1 infection	26/194	212/8163	3.80E-12	2.32E-11	8.89E-12	MAPK14/CHEK1/MAPK10/RELA/JUN/IKBKB/AKT1/BCL2/BAX/CASP3/MAPK8/CDK1/PPP3CA/MAPK3/MAPK1/BAD/BCL2L1/FOS/CASP9/NFKBIA/CASP8/RAF1/PRKCA/PRKCB/CCNB1/CHUK	26
Kegg Path way	hsa04370	VEGF signaling pathway	15/194	59/8163	4.04E-12	2.42E-11	9.25E-12	PTGS2/KDR/MAPK14/AKT1/PPP3CA/MAPK3/MAPK1/BAD/VEGFA/CASP9/RAF1/PRKCA/PRKCB/NOS3/HSPB1	15
Kegg Path	hsa049	Prolactin	16/194	70/8163	4.49E-12	2.63E-11	1.01E-11	ESR1/MAPK14/GSK3B/ESR2/MAPK10/RELA	16

way	17	signaling pathway						/AKT1/MAPK8/STAT1/MAPK3/MAPK1/STAT3/CCND1/FOS/RAF1/IRF1	
Kegg Pathway	hsa04919	Thyroid hormone signaling pathway	20/194	121/8163	5.39E-12	3.09E-11	1.18E-11	RXRA/NCOA2/NCOA1/ESR1/GSK3B/AKT1/STAT1/DIO1/MAPK3/MAPK1/BAD/RXRB/CCND1/CASP9/TP53/RAF1/PRKCA/HIF1A/MYC/PRKCB	20
Kegg Pathway	hsa04010	MAPK signaling pathway	30/194	294/8163	8.86E-12	4.97E-11	1.90E-11	KDR/MAPK14/MAPK10/RELA/JUN/IKBKB/AKT1/CASP3/MAPK8/PPP3CA/MAPK3/MAPK1/EGFR/VEGFA/FOS/EGF/TP53/ELK1/RAF1/PRKCA/ERBB2/MYC/IL1B/PRKCB/HSPB1/IL1A/CHUK/IGF2/ERBB3/RASA1	30
Kegg Pathway	hsa05214	Glioma	16/194	75/8163	1.39E-11	7.67E-11	2.94E-11	AKT1/BAX/MAPK3/MAPK1/CCND1/RB1/CDK4/EGFR/CDKN1A/EGF/TP53/RAF1/PRKCA/PRKCB/E2F1/E2F2	16
Kegg Pathway	hsa04380	Osteoclast differentiation	20/194	128/8163	1.58E-11	8.52E-11	3.26E-11	PPARG/MAPK14/MAPK10/RELA/NCF1/JUN/IKBKB/AKT1/MAPK8/STAT1/PPP3CA/MAPK3/MAPK1/FOSL2/FOS/NFKBIA/IL1B/IFNG/IL1A/CHUK	20
Kegg Pathway	hsa04625	C-type lectin receptor signaling pathway	18/194	104/8163	2.95E-11	1.54E-10	5.89E-11	PTGS2/MAPK14/MAPK10/RELA/JUN/IKBKB/AKT1/MAPK8/STAT1/PPP3CA/MAPK3/MAPK1/NFKBIA/CASP8/RAF1/IL1B/CHUK/IRF1	18

Kegg Pathway	hsa05221	Acute myeloid leukemia	15/194	67/8163	2.97E-11	1.54E-10	5.89E-11	CCNA2/PPARD/RELA/IKBKB/AKT1/MAPK3/MAPK1/BAD/STAT3/CCND1/RAF1/RUNX1T1/MYC/MPO/CHUK	15
Kegg Pathway	hsa04932	Non-alcoholic fatty liver disease	21/194	155/8163	7.81E-11	3.98E-10	1.52E-10	RXRA/PPARG/MAPK14/GSK3B/MAPK10/RELA/JUN/IKBKB/AKT1/BAX/CASP3/MAPK8/PPARA/SREBF1/ADIPOR2/FOS/IL6R/CASP8/IL1B/CXCL8/IL1A	21
Kegg Pathway	hsa05218	Melanoma	15/194	72/8163	8.93E-11	4.47E-10	1.71E-10	AKT1/BAX/MAPK3/MAPK1/BAD/CCND1/RB1/CDK4/EGFR/CDKN1A/EGF/TP53/RAF1/E2F1/E2F2	15
Kegg Pathway	hsa05152	Tuberculosis	22/194	180/8163	2.09E-10	1.03E-09	3.94E-10	NOS2/MAPK14/MAPK10/RELA/AKT1/BCL2/BAX/CASP3/MAPK8/STAT1/PPP3CA/MAPK3/MAPK1/BAD/CASP9/IL10RA/CASP8/RAF1/IL1B/IFNG/IL1A/CTSD	22
Kegg Pathway	hsa05226	Gastric cancer	20/194	149/8163	2.69E-10	1.30E-09	4.97E-10	RXRA/GSK3B/CDK2/AKT1/BCL2/BAX/MAPK3/MAPK1/RXRB/CCND1/RB1/EGFR/CDKN1A/EGF/TP53/RAF1/ERBB2/MYC/E2F1/E2F2	20
Kegg Pathway	hsa04658	Th1 and Th2 cell differentiation	16/194	92/8163	3.61E-10	1.71E-09	6.55E-10	MAPK14/MAPK10/RELA/JUN/IL4/IKBKB/MAPK8/STAT1/PPP3CA/MAPK3/MAPK1/FOS/NFKBIA/IL2RA/IFNG/CHUK	16
Kegg Pathway	hsa04915	Estrogen signaling pathway	19/194	138/8163	4.96E-10	2.31E-09	8.85E-10	PGR/OPRM1/NCOA2/NCOA1/HSP90AB1/ESR1/ESR2/JUN/AKT1/BCL2/MAPK3/MAPK1/EGFR/FOS/MMP2/MMP9/RAF1/NOS3/CTSD	19

Kegg Pathway	hsa04068	FoxO signaling pathway	18/194	131/8163	1.50E-09	6.89E-09	2.64E-09	MAPK14/CDK2/MAPK10/IKBKB/AKT1/MAPK8/SLC2A4/MAPK3/MAPK1/CAT/STAT3/CCND1/EGFR/CDKN1A/EGF/RAF1/CCNB1/CHUK	18
Kegg Pathway	hsa04510	Focal adhesion	22/194	201/8163	1.78E-09	8.01E-09	3.07E-09	KDR/GSK3B/MAPK10/JUN/AKT1/BCL2/MAPK8/MAPK3/MAPK1/BAD/CCND1/EGFR/VEGFA/EGF/ELK1/RAF1/PRKCA/ERBB2/CAV1/PRKCB/COL1A1/SPP1	22
Kegg Pathway	hsa05133	Pertussis	14/194	76/8163	2.17E-09	9.60E-09	3.68E-09	NOS2/MAPK14/MAPK10/RELA/JUN/CASP3/MAPK8/MAPK3/MAPK1/FOS/IL1B/CXCL8/IL1A/IRF1	14
Kegg Pathway	hsa04064	NF-kappa B signaling pathway	16/194	104/8163	2.37E-09	1.02E-08	3.91E-09	PTGS2/RELA/IKBKB/BCL2/ICAM1/VCAM1/BCL2L1/PLAU/NFKBIA/IL1B/CXCL8/PRKCB/PARP1/CXCL2/CHUK/CD40LG	16
Kegg Pathway	hsa04071	Sphingolipid signaling pathway	17/194	119/8163	2.38E-09	1.02E-08	3.91E-09	MAPK14/MAPK10/OPRD1/RELA/AKT1/BCL2/BAX/MAPK8/MAPK3/MAPK1/ABCC1/TP53/RAF1/PRKCA/PRKCB/NOS3/CTSD	17
Kegg Pathway	hsa05165	Human papillomavirus infection	28/194	331/8163	3.66E-09	1.55E-08	5.92E-09	PTGS2/GSK3B/CCNA2/CDK2/RELA/IKBKB/AKT1/BAX/CASP3/STAT1/MAPK3/MAPK1/BAD/CCND1/RB1/CDK4/EGFR/VEGFA/CDKN1A/EGF/TP53/CASP8/RAF1/COL1A1/CHUK/SPP1/E2F1/IRF1	28
Kegg Pathway	hsa05171	Coronavirus disea	23/194	232/8163	5.17E-09	2.16E-08	8.25E-09	MAPK14/MAPK10/RELA/JUN/IKBKB/MAPK8/MMP1/STAT1/MAPK3/MAPK1/STAT3/MMP3	23

		se - COVID -19						/EGFR/FOS/IL6R/NFKBIA/PRKCA/IL1B/CCL2/CXCL8/PRKCB/CXCL10/CHUK	
Kegg Pathway	hsa 04920	Adipocytokine signaling pathway	13/194	69/8163	6.21E-09	2.55E-08	9.76E-09	RXRA/MAPK10/RELA/IKBKB/AKT1/MAPK8/SLC2A4/PPARA/ADIPOR2/RXRB/STAT3/NFKBIA/CHUK	13
Kegg Pathway	hsa 05216	Thyroid cancer	10/194	37/8163	9.09E-09	3.68E-08	1.41E-08	RXRA/PPARG/BAX/MAPK3/MAPK1/RXRB/CCND1/CDKN1A/TP53/MYC	10
Kegg Pathway	hsa 05206	MicroRNAs in cancer	26/194	310/8163	1.68E-08	6.68E-08	2.56E-08	PTGS2/IKBKB/BCL2/CASP3/HMOX1/CYP1B1/MAPK3/MAPK1/ABCC1/STAT3/CCND1/EGFR/VEGFA/CDKN1A/PLAU/MMP9/TP53/RAF1/PRKCA/ERBB2/MYC/PRKCB/RASSF1/E2F1/E2F2/ERBB3	26
Kegg Pathway	hsa 04722	Neurotrophin signaling pathway	16/194	119/8163	1.75E-08	6.89E-08	2.64E-08	MAPK14/GSK3B/MAPK10/RELA/JUN/IKBKB/AKT1/BCL2/BAX/MAPK8/MAPK3/MAPK1/BAD/TP53/NFKBIA/RAF1	16
Kegg Pathway	hsa 04215	Apoptosis - multiple species	9/194	32/8163	3.51E-08	1.36E-07	5.21E-08	MAPK10/BCL2/BAX/CASP3/MAPK8/BCL2L1/CASP9/CASP8/BIRC5	9
Kegg Pathway	hsa 04020	Calcium signaling pathway	22/194	240/8163	4.79E-08	1.83E-07	7.00E-08	CHRM3/CHRM1/ADRA1A/CHRM2/ADRB2/ADRA1B/ADRA1D/NOS2/KDR/DRD1/CHRM5/ADRB1/PPP3CA/EGFR/VEGFA/EGF/PRKCA/ERBB2/PTGER3/PRKCB/N	22

								OS3/ERBB3	
Kegg Pathway	hsa04662	B cell receptor signaling pathway	13/194	82/8163	5.47E-08	2.06E-07	7.89E-08	GSK3B/RELA/JUN/IKBKB/AKT1/PPP3CA/MAPK3/MAPK1/FOS/NFKBIA/RAF1/PRKCB/CHUK	13
Kegg Pathway	hsa05415	Diabetic cardiomyopathy	20/194	203/8163	6.15E-08	2.29E-07	8.75E-08	MAPK14/GSK3B/MAPK10/RELA/NCF1/ATP5F1B/AKT1/MAPK8/SLC2A4/PPARA/GSR/MMP2/MMP9/PRKCA/PRKCB/NOS3/COL1A1/PARP1/COL3A1/CTSD	20
Kegg Pathway	hsa04621	NOD-like receptor signaling pathway	19/194	184/8163	6.31E-08	2.31E-07	8.86E-08	HSP90AB1/MAPK14/MAPK10/RELA/JUN/IKBKB/BCL2/MAPK8/STAT1/MAPK3/MAPK1/BCL2L1/NFKBIA/CASP8/IL1B/CCL2/CXCL8/CXCL2/CHUK	19
Kegg Pathway	hsa05022	Pathways of neurodegeneration - multiple diseases	32/194	476/8163	6.85E-08	2.48E-07	9.49E-08	PTGS2/CHRM3/CHRM1/GRIA2/NOS2/MAPK14/GSK3B/CHRM5/SLC6A3/MAPK10/RELA/ATP5F1B/BCL2/BAX/CASP3/MAPK8/PSMD3/PPP3CA/MAPK3/MAPK1/BAD/SOD1/CAT/BCL2L1/CASP9/CASP8/RAF1/PRKCA/HSPA5/IL1B/PRKCB/IL1A	32
Kegg Pathway	hsa05120	Epithelial cell signaling in Helicobacter pylor	12/194	70/8163	7.36E-08	2.63E-07	1.01E-07	MAPK14/MAPK10/RELA/JUN/IKBKB/CASP3/MAPK8/EGFR/NFKBIA/CXCL8/CXCL2/CHUK	12

		i infection							
Kegg Pathway	hsa04062	Chemokine signaling pathway	19/194	192/8163	1.25E-07	4.41E-07	1.69E-07	GSK3B/RELA/NCF1/IKBKB/AKT1/STAT1/MAPK3/MAPK1/BAD/STAT3/NFKBIA/RAF1/CCL2/CXCL8/PRKCB/CXCL11/CXCL2/CXCL10/CHUK	19
Kegg Pathway	hsa04910	Insulin signaling pathway	16/194	137/8163	1.33E-07	4.56E-07	1.75E-07	GSK3B/MAPK10/PYGM/IKBKB/AKT1/MAPK8/SLC2A4/MAPK3/MAPK1/FASN/BAD/SREBF1/ELK1/RAF1/ACACA/HK2	16
Kegg Pathway	hsa05135	Yersinia infection	16/194	137/8163	1.33E-07	4.56E-07	1.75E-07	MAPK14/GSK3B/MAPK10/RELA/JUN/IKBKB/AKT1/MAPK8/MAPK3/MAPK1/FOS/NFKBIA/IL1B/CCL2/CXCL8/CHUK	16
Kegg Pathway	hsa05202	Transcriptional misregulation in cancer	19/194	193/8163	1.36E-07	4.61E-07	1.76E-07	RXRA/PPARG/CCNA2/RELA/BAX/RXRB/MMP3/BCL2L1/CDKN1A/PLAU/MMP9/TP53/RUNX1T1/MYC/CXCL8/PLAT/MPO/RUNX2/IGFBP3	19
Kegg Pathway	hsa04014	Ras signaling pathway	21/194	235/8163	1.53E-07	5.13E-07	1.96E-07	KDR/MAPK10/RELA/IKBKB/AKT1/MAPK8/MAPK3/MAPK1/BAD/EGFR/VEGFA/BCL2L1/EGF/ELK1/RAF1/PRKCA/PRKCB/CHUK/RASSF1/IGF2/RASA1	21
Kegg Pathway	hsa04931	Insulin resistance	14/194	108/8163	2.26E-07	7.49E-07	2.87E-07	GSK3B/MAPK10/PYGM/RELA/IKBKB/AKT1/MAPK8/SLC2A4/PPARA/SREBF1/STAT3/NFKBIA/PRKCB/NOS3	14
Kegg Path	hsa041	Cell cycle	15/194	126/8163	2.58E-07	8.45E-07	3.23E-07	GSK3B/CHEK1/CCNA2/CDK2/CDK1/CCND1/	15

way	10							RB1/CDK4/CDKN1A/TP53/MYC/CCNB1/CHEK2/E2F1/E2F2	
Kegg Pathway	hsa05203	Viral carcinogenesis	19/194	204/8163	3.26E-07	1.05E-06	4.03E-07	CHEK1/CCNA2/CDK2/RELA/JUN/BAX/CASP3/CDK1/MAPK3/MAPK1/BAD/STAT3/CCND1/RB1/CDK4/CDKN1A/TP53/NFKBIA/CASP8	19
Kegg Pathway	hsa05132	Salmonella infection	21/194	249/8163	4.06E-07	1.30E-06	4.97E-07	HSP90AB1/MAPK14/MAPK10/RELA/JUN/IKBKB/AKT1/BCL2/BAX/CASP3/MAPK8/MAPK3/MAPK1/FOS/NFKBIA/CASP8/RAF1/MYC/IL1B/CXCL8/CHUK	21
Kegg Pathway	hsa05231	Choline metabolism in cancer	13/194	98/8163	4.72E-07	1.49E-06	5.71E-07	MAPK10/JUN/AKT1/MAPK8/MAPK3/MAPK1/EGFR/FOS/EGF/RAF1/PRKCA/HIF1A/PRKCB	13
Kegg Pathway	hsa04914	Progesterone-mediated oocyte maturation	13/194	102/8163	7.56E-07	2.36E-06	9.05E-07	PGR/HSP90AB1/MAPK14/CCNA2/CDK2/MAPK10/AKT1/MAPK8/CDK1/MAPK3/MAPK1/RAF1/CCNB1	13
Kegg Pathway	hsa04935	Growth hormone synthesis, secretion and action	14/194	120/8163	8.43E-07	2.60E-06	9.97E-07	MAPK14/GSK3B/MAPK10/AKT1/MAPK8/STAT1/MAPK3/MAPK1/STAT3/FOS/RAF1/PRKCA/PRKCB/IGFBP3	14
Kegg	hsa	Patho	18/19	197/8	8.98E-07	2.74E-06	1.05E-06	MAPK14/MAPK10/REL	18

Path way	05130	genic Escherichia coli infection	4	163				A/JUN/IKBKB/BAX/CASP3/MAPK8/MAPK3/MAPK1/FOS/CASP9/NFKBIA/CASP8/IL1B/CXCL8/CLDN4/CHUK	
Kegg Path way	hsa04024	cAMP signaling pathway	19/194	221/8163	1.12E-06	3.39E-06	1.30E-06	CHRM1/CHRM2/ADRB2/GRIA2/DRD1/MAPK10/RELA/ADRB1/JUN/AKT1/MAPK8/MAPK3/MAPK1/BAD/PPARA/FOS/NFKBIA/RAF1/PTGER3	19
Kegg Path way	hsa04630	JAK-STAT signaling pathway	16/194	162/8163	1.33E-06	3.98E-06	1.52E-06	IL4/AKT1/BCL2/STAT1/STAT3/CCND1/EGFR/BCL2L1/CDKN1A/IL10RA/EGF/IL6R/RAF1/MYC/IL2RA/IFNG	16
Kegg Path way	hsa00140	Steroid hormone biosynthesis	10/194	61/8163	1.43E-06	4.24E-06	1.62E-06	HSD3B2/HSD3B1/CYP3A4/CYP1A2/CYP1A1/CYP1B1/AKR1C3/CYP19A1/UGT1A1/SULT1E1	10
Kegg Path way	hsa05131	Shigellosis	20/194	247/8163	1.49E-06	4.35E-06	1.66E-06	MAPK14/GSK3B/MAPK10/RELA/JUN/IKBKB/AKT1/BCL2/BAX/MAPK8/MAPK3/MAPK1/EGFR/BCL2L1/TP53/NFKBIA/IL1B/CXCL8/CHUK/HK2	20
Kegg Path way	hsa04912	GnRH signaling pathway	12/194	93/8163	1.79E-06	5.12E-06	1.96E-06	MAPK14/MAPK10/JUN/MAPK8/MAPK3/MAPK1/EGFR/MMP2/ELK1/RAF1/PRKCA/PRKCB	12
Kegg Path way	hsa05323	Rheumatoid arthritis	12/194	93/8163	1.79E-06	5.12E-06	1.96E-06	JUN/MMP1/ICAM1/MMP3/VEGFA/FOS/IL1B/CCL2/CXCL8/IFNG/IL1A/CXCL2	12
Kegg Path	hsa049	Ovarian	9/194	51/8163	2.58E-06	7.31E-06	2.80E-06	PTGS2/HSD3B2/HSD3B1/CYP1A1/CYP1B1/	9

way	13	steroidogenesis						ALOX5/AKR1C3/LDLR/CYP19A1	
Kegg Pathway	hsa04934	Cushing syndrome	15/194	155/8163	3.72E-06	1.05E-05	4.00E-06	GSK3B/CDK2/HSD3B2/HSD3B1/AHR/MAPK3/MAPK1/LDLR/CCND1/RB1/CDK4/EGFR/CDKN1A/E2F1/E2F2	15
Kegg Pathway	hsa05010	Alzheimer disease	25/194	384/8163	3.91E-06	1.09E-05	4.16E-06	PTGS2/CHRM3/CHRM1/NOS2/GSK3B/CHRM5/MAPK10/RELA/ATP5F1B/IKBKB/AKT1/CASP3/MAPK8/PSMD3/PPP3CA/MAPK3/MAPK1/BAD/BACE2/CASP9/CASP8/RAF1/IL1B/IL1A/CHUK	25
Kegg Pathway	hsa04664	Fc epsilon RI signaling pathway	10/194	68/8163	4.00E-06	1.10E-05	4.21E-06	MAPK14/MAPK10/IL4/AKT1/MAPK8/ALOX5/MAPK3/MAPK1/RAF1/PRKCA	10
Kegg Pathway	hsa05146	Amoebiasis	12/194	102/8163	4.80E-06	1.31E-05	5.01E-06	NOS2/RELA/CASP3/PRKCA/IL1B/CXCL8/PRKCB/HSPB1/COL1A1/IFNG/COL3A1/CXCL2	12
Kegg Pathway	hsa04622	RIG-I-like receptor signaling pathway	10/194	70/8163	5.24E-06	1.40E-05	5.35E-06	MAPK14/MAPK10/RELA/IKBKB/MAPK8/NFKBIA/CASP8/CXCL8/CXCL10/CHUK	10
Kegg Pathway	hsa05230	Central carbon metabolism in	10/194	70/8163	5.24E-06	1.40E-05	5.35E-06	AKT1/MAPK3/MAPK1/EGFR/TP53/RAF1/HIF1A/ERBB2/MYC/HK2	10

		cancer							
Kegg Pathway	hsa04540	Gap junction	11/194	88/8163	6.67E-06	1.76E-05	6.75E-06	DRD1/ADRB1/CDK1/MAPK3/MAPK1/EGFR/EGF/RAF1/PRKCA/GJA1/PRKCB	11
Kegg Pathway	hsa05134	Legionellosis	9/194	57/8163	6.74E-06	1.77E-05	6.76E-06	RELA/CASP3/CASP9/NFKBIA/CASP8/IL1B/CXCL8/CXCL2/HSF1	9
Kegg Pathway	hsa04928	Parathyroid hormone synthesis, secretion and action	12/194	106/8163	7.20E-06	1.87E-05	7.15E-06	RXRA/BCL2/MAPK3/MAPK1/RXRB/EGFR/FOS/CDKN1A/RAF1/PRKCA/PRKCB/RUNX2	12
Kegg Pathway	hsa04728	Dopaminergic synapse	13/194	132/8163	1.40E-05	3.55E-05	1.36E-05	GRIA2/MAOB/MAPK14/GSK3B/DRD1/SLC6A3/MAPK10/AKT1/MAPK8/PPP3CA/FOS/PRKCA/PRKCB	13
Kegg Pathway	hsa04725	Cholinergic synapse	12/194	113/8163	1.40E-05	3.55E-05	1.36E-05	CHRM3/CHRM1/ACHE/CHRM2/CHRM5/AKT1/BCL2/MAPK3/MAPK1/FOS/PRKCA/PRKCB	12
Kegg Pathway	hsa00980	Metabolism of xenobiotics by cytochrome P450	10/194	78/8163	1.41E-05	3.55E-05	1.36E-05	ADH1B/ADH1C/CYP3A4/CYP1A2/CYP1A1/CYP1B1/GSTP1/GSTM1/GSTM2/UGT1A1	10
Kegg Pathway	hsa04921	Oxytocin signaling	14/194	154/8163	1.63E-05	4.08E-05	1.56E-05	PTGS2/JUN/PPP3CA/MAPK3/MAPK1/CCND1/EGFR/FOS/CDKN1A/ELK1/RAF1/PRKCA/P	14

		pathway						RKCB/NOS3	
Kegg Pathway	hsa04726	Serotonergic synapse	12/194	115/8163	1.67E-05	4.15E-05	1.59E-05	PTGS2/PTGS1/HTR3A/MAOB/SLC6A4/CASP3/ALOX5/MAPK3/MAPK1/RAF1/PRKCA/PRKCB	12
Kegg Pathway	hsa05144	Malaria	8/194	50/8163	2.01E-05	4.93E-05	1.89E-05	ICAM1/SELE/VCAM1/IL1B/CCL2/CXCL8/IFNG/CD40LG	8
Kegg Pathway	hsa05143	African trypanosomiasis	7/194	37/8163	2.16E-05	5.26E-05	2.01E-05	ICAM1/SELE/VCAM1/PRKCA/IL1B/PRKCB/IFNG	7
Kegg Pathway	hsa05020	Prion disease	19/194	273/8163	2.45E-05	5.91E-05	2.26E-05	MAPK14/GSK3B/MAPK10/NCF1/ATP5F1B/BAX/CASP3/MAPK8/PSMD3/PPP3CA/MAPK3/MAPK1/BAD/SOD1/CASP9/HSPA5/CAV1/IL1B/IL1A	19
Kegg Pathway	hsa05031	Amphetamine addiction	9/194	69/8163	3.32E-05	7.81E-05	2.99E-05	GRIA2/MAOB/DRD1/SLC6A3/JUN/PPP3CA/FOS/PRKCA/PRKCB	9
Kegg Pathway	hsa05204	Chemical carcinogenesis - DNA adducts	9/194	69/8163	3.32E-05	7.81E-05	2.99E-05	PTGS2/CYP3A4/CYP1A2/CYP1A1/CYP1B1/GSTP1/GSTM1/GSTM2/UGT1A1	9
Kegg Pathway	hsa05211	Renal cell carcinoma	9/194	69/8163	3.32E-05	7.81E-05	2.99E-05	JUN/AKT1/MAPK3/MAPK1/BAD/VEGFA/CDKN1A/RAF1/HIF1A	9
Kegg Pathway	hsa00982	Drug metabolism - cytoc	9/194	72/8163	4.69E-05	0.000109323	4.18E-05	ADH1B/ADH1C/MAOB/CYP3A4/CYP1A2/GSTP1/GSTM1/GSTM2/UGT1A1	9

		hrome P450							
Kegg Pathway	hsa01523	Antifolate resistance	6/194	30/8163	6.15E-05	0.000142178	5.44E-05	RELA/IKBKB/ABCC1/IL1B/ABCG2/CHUK	6
Kegg Pathway	hsa04930	Type II diabetes mellitus	7/194	46/8163	9.38E-05	0.000214893	8.23E-05	MAPK10/IKBKB/MAPK8/SLC2A4/MAPK3/MAPK1/HK2	7
Kegg Pathway	hsa04217	Necroptosis	13/194	159/8163	9.95E-05	0.000226131	8.66E-05	HSP90AB1/MAPK10/PYGM/BCL2/BAX/MAPK8/STAT1/STAT3/CASP8/IL1B/IFNG/IL1A/PARP1	13
Kegg Pathway	hsa04929	GnRH secretion	8/194	64/8163	0.000124683	0.000281047	0.000107578	ESR2/AKT1/MAPK3/MAPK1/RAF1/PRKCA/PRKCB/SPP1	8
Kegg Pathway	hsa04152	AMPK signaling pathway	11/194	121/8163	0.000134485	0.000300678	0.000115092	ADRA1A/PPARG/CCNA2/AKT1/SLC2A4/FASN/HMGCR/SREBF1/ADIPOR2/CCND1/ACACA	11
Kegg Pathway	hsa05321	Inflammatory bowel disease	8/194	65/8163	0.000139328	0.000308994	0.000118275	RELA/JUN/IL4/STAT1/STAT3/IL1B/IFNG/IL1A	8
Kegg Pathway	hsa04022	cGMP-PKG signaling pathway	13/194	167/8163	0.000163516	0.000359735	0.000137698	ADRA1A/ADRB2/ADRA1B/ADRA1D/OPRD1/ADRB1/AKT1/PPP3CA/MAPK3/MAPK1/BAD/RAF1/NOS3	13
Kegg Pathway	hsa04261	Adrenergic signaling in cardiomyoc	12/194	150/8163	0.000226608	0.000494582	0.000189314	SCN5A/ADRA1A/ADRB2/ADRA1B/ADRA1D/MAPK14/ADRB1/AKT1/BCL2/MAPK3/MAPK1/PRKCA	12

		ytes							
Kegg Pathway	hsa04137	Mitophagy - animal	8/194	72/8163	0.00028671	0.000620829	0.000237638	MAPK10/RELA/JUN/MAPK8/BCL2L1/TP53/HIF1A/E2F1	8
Kegg Pathway	hsa05416	Viral myocarditis	7/194	60/8163	0.000511117	0.001093058	0.000418395	CASP3/ICAM1/CCND1/CASP9/CASP8/CAV1/CD40LG	7
Kegg Pathway	hsa04140	Autophagy - animal	11/194	141/8163	0.000512744	0.001093058	0.000418395	MAPK10/AKT1/BCL2/MAPK8/MAPK3/MAPK1/BAD/BCL2L1/RAF1/HIF1A/CTSD	11
Kegg Pathway	hsa05030	Cocaine addiction	6/194	49/8163	0.000996079	0.002100798	0.000804133	GRIA2/MAOB/DRD1/SLC6A3/RELA/JUN	6
Kegg Pathway	hsa04720	Long-term potentiation	7/194	67/8163	0.001000744	0.002100798	0.000804133	GRIA2/PPP3CA/MAPK3/MAPK1/RAF1/PRKCA/PRKCB	7
Kegg Pathway	hsa04970	Salivary secretion	8/194	92/8163	0.001488765	0.003101594	0.001187213	CHRM3/ADRA1A/ADRB2/ADRA1B/ADRA1D/ADRB1/PRKCA/PRKCB	8
Kegg Pathway	hsa04670	Leukocyte transendothelial migration	9/194	114/8163	0.001516823	0.003136288	0.001200493	MAPK14/NCF1/ICAM1/VCAM1/MMP2/MMP9/PRKCA/PRKCB/CLDN4	9
Kegg Pathway	hsa05012	Parkinson disease	15/194	266/8163	0.00163748	0.0033605	0.001286316	MAOB/DRD1/SLC6A3/MAPK10/ATP5F1B/BAX/CASP3/MAPK8/PSMD3/SOD1/BCL2L1/CASP9/TP53/HSPA5/NFE2L2	15
Kegg Pathway	hsa04960	Aldosterone-regulate	5/194	37/8163	0.001691351	0.003445025	0.00131867	NR3C2/MAPK3/MAPK1/PRKCA/PRKCB	5

		d sodium reabsorption							
Kegg Pathway	hsa04371	Apelin signaling pathway	10/194	139/8163	0.001703721	0.003445025	0.00131867	NOS2/AKT1/MAPK3/MAPK1/CCND1/RAF1/NOS3/PLAT/SERPINE1/SPP1	10
Kegg Pathway	hsa03320	PPAR signaling pathway	7/194	75/8163	0.001947465	0.003909145	0.001496324	RXRA/PPARG/PPARD/OLR1/MMP1/PPARA/RXRB	7
Kegg Pathway	hsa04923	Regulation of lipolysis in adipocytes	6/194	57/8163	0.002209377	0.004402743	0.001685261	PTGS2/ADRB2/PTGS1/ADRB1/AKT1/PTGER3	6
Kegg Pathway	hsa04310	Wnt signaling pathway	11/194	170/8163	0.002369682	0.004688219	0.001794534	GSK3B/MAPK10/PPARD/JUN/MAPK8/PPP3CA/CCND1/TP53/PRKCA/MYC/PRKCB	11
Kegg Pathway	hsa04061	Viral protein interaction with cytokine and cytokine receptor	8/194	100/8163	0.002533715	0.00497694	0.001905049	IL10RA/IL6R/CCL2/CXCL8/IL2RA/CXCL11/CXCL2/CXCL10	8
Kegg	hsa	Retro	10/19	148/8	0.002710	0.005285	0.002023	PTGS2/GABRA1/GRIA	10

Path way	047 23	grade endoc annab inoid signa ling	4	163	046	552	178	2/MAPK14/MAPK10/MAPK8/MAPK3/MAPK1/PRKCA/PRKCB	
Kegg Path way	hsa 009 83	Drug metab olism - other enzym es	7/194	80/81 63	0.002821 924	0.005464 993	0.002091 863	CYP3A4/GSTP1/GSTM1/GSTM2/UGT1A1/CES1/MPO	7
Kegg Path way	hsa 047 30	Long-term depre ssion	6/194	60/81 63	0.002874 255	0.005527 413	0.002115 756	GRIA2/MAPK3/MAPK1/RAF1/PRKCA/PRKCB	6
Kegg Path way	hsa 003 80	Trypt ophan metab olism	5/194	42/81 63	0.002999 195	0.005727 63	0.002192 394	MAOB/CYP1A2/CYP1A1/CYP1B1/CAT	5
Kegg Path way	hsa 005 90	Arach idoni c acid metab olism	6/194	61/81 63	0.003126 07	0.005928 754	0.002269 38	PTGS2/PTGS1/LTA4H/ALOX5/AKR1C3/PLB1	6
Kegg Path way	hsa 046 23	Cytos olic DNA-s ensin g pathw ay	6/194	63/81 63	0.003678 679	0.006929 018	0.002652 256	RELA/IKBKB/NFKBIA/IL1B/CXCL10/CHUK	6
Kegg Path way	hsa 041 14	Oocyt e meios is	9/194	131/8 163	0.003918 993	0.007281 913	0.002787 335	PGR/AR/MAPK14/CDK2/CDK1/PPP3CA/MAPK3/MAPK1/CCNB1	9
Kegg Path way	hsa 046 50	Natur al kille r cell media ted cytot	9/194	131/8 163	0.003918 993	0.007281 913	0.002787 335	CASP3/ICAM1/PPP3CA/MAPK3/MAPK1/RAF1/PRKCA/PRKCB/IFNG	9

		oxicity							
Kegg Pathway	hsa04211	Longevity regulating pathway	7/194	89/8163	0.00511992	0.00938652	0.003592926	PPARG/RELA/AKT1/BAX/CAT/ADIPOR2/TP53	7
Kegg Pathway	hsa04976	Bile secretion	7/194	89/8163	0.00511992	0.00938652	0.003592926	RXRA/CYP3A4/LDLR/HMGCR/UGT1A1/CA2/ABCG2	7
Kegg Pathway	hsa00830	Retinol metabolism	6/194	68/8163	0.005375077	0.009789048	0.003747004	ADH1B/ADH1C/CYP3A4/CYP1A2/CYP1A1/UGT1A1	6
Kegg Pathway	hsa00330	Arginine and proline metabolism	5/194	50/8163	0.006412173	0.011600971	0.004440563	MAOB/NOS2/GOT1/ODC1/NOS3	5
Kegg Pathway	hsa04666	Fc gamma R-mediated phagocytosis	7/194	97/8163	0.008148631	0.014646232	0.005606213	NCF1/AKT1/MAPK3/MAPK1/RAF1/PRKCA/PRKCB	7
Kegg Pathway	hsa04611	Platelet activation	8/194	124/8163	0.009266612	0.016547521	0.006333979	PTGS1/MAPK14/AKT1/MAPK3/MAPK1/NOS3/COL1A1/COL3A1	8
Kegg Pathway	hsa00350	Tyrosine metabolism	4/194	36/8163	0.010055109	0.01783971	0.006828597	ADH1B/ADH1C/MAOB/GOT1	4
Kegg Pathway	hsa00480	Glutathione metabolism	5/194	57/8163	0.011093397	0.019555667	0.007485423	GSTP1/GSTM1/GSTM2/GSR/ODC1	5
Kegg Path	hsa040	Rap1 signa	11/194	210/8163	0.011388293	0.019947647	0.007635463	KDR/MAPK14/AKT1/MAPK3/MAPK1/EGFR/V	11

way	15	ling pathway						EGFA/EGF/RAF1/PRKCA/PRKCB	
Kegg Pathway	hsa04150	mTOR signaling pathway	9/194	156/8163	0.011929829	0.020763944	0.007947921	GSK3B/IKBKB/AKT1/MAPK3/MAPK1/RAF1/PRKCA/PRKCB/CHUK	9
Kegg Pathway	hsa04080	Neuroactive ligand-receptor interaction	16/194	362/8163	0.012241309	0.021172075	0.008104144	CHRM3/CHRM1/ADRA1A/CHRM2/ADRB2/OPRM1/GABRA1/ADRA1B/GRIA2/ADRA1D/PRSS1/DRD1/CHRM5/OPRD1/ADRB1/PTGER3	16
Kegg Pathway	hsa05014	Amyotrophic lateral sclerosis	16/194	364/8163	0.01285014	0.022086178	0.008454039	GRIA2/NOS2/MAPK14/ATP5F1B/BCL2/BAX/CASP3/PSMD3/PPP3CA/BAD/SOD1/CAT/BCL2L1/CASP9/TP53/HSPA5	16
Kegg Pathway	hsa04270	Vascular smooth muscle contraction	8/194	134/8163	0.01436842	0.024542333	0.009394194	ADRA1A/ADRA1B/ADRA1D/MAPK3/MAPK1/RAF1/PRKCA/PRKCB	8
Kegg Pathway	hsa00220	Arginine biosynthesis	3/194	22/8163	0.014592764	0.024771668	0.009481978	NOS2/GOT1/NOS3	3
Kegg Pathway	hsa04613	Neutrophil extracellular trap	10/194	190/8163	0.015046417	0.025385059	0.009716769	MAPK14/RELA/NCF1/AKT1/MAPK3/MAPK1/RAF1/PRKCA/PRKCB/MPO	10

		formation							
Kegg Pathway	hsa04610	Complement and coagulation cascades	6/194	85/8163	0.015439708	0.025889755	0.009909954	F7/PLAU/F3/PLAT/THBD/SERPINE1	6
Kegg Pathway	hsa04977	Vitamin digestion and absorption	3/194	24/8163	0.018526333	0.030877221	0.011819032	APOB/PLB1/ABCC1	3
Kegg Pathway	hsa04550	Signaling pathways regulating pluripotency of stem cells	8/194	143/8163	0.020487472	0.03394009	0.012991422	MAPK14/GSK3B/AKT1/MAPK3/MAPK1/STAT3/RAF1/MYC	8
Kegg Pathway	hsa04060	Cytokine-cytokine receptor interaction	13/194	295/8163	0.023410556	0.038550317	0.014756102	IL4/IL10RA/IL6R/IL1B/CCL2/CXCL8/IL2RA/IFNG/IL1A/CXCL11/CXCL2/CXCL10/CD40LG	13
Kegg Pathway	hsa04072	Phospholipase D signaling pathway	8/194	148/8163	0.024592628	0.040255791	0.015408915	AKT1/MAPK3/MAPK1/EGFR/EGF/RAF1/PRKCA/CXCL8	8

Kegg Pathway	hsa04713	Circadian entrainment	6/194	97/8163	0.027680104	0.04504159	0.0172408	GRIA2/MAPK3/MAPK1/FOS/PRKCA/PRKCB	6
Kegg Pathway	hsa04750	Inflammatory mediator regulation of TRP channels	6/194	98/8163	0.028924207	0.046789158	0.017909726	MAPK14/MAPK10/MAPK8/PRKCA/IL1B/PRKCB	6
Kegg Pathway	hsa00591	Linoleic acid metabolism	3/194	29/8163	0.030684304	0.049346104	0.018888461	CYP3A4/CYP1A2/PLB1	3
Kegg Pathway	hsa04979	Cholesterol metabolism	4/194	51/8163	0.032446128	0.051769306	0.019816002	LDLR/APOB/SOAT2/SOAT1	4
Kegg Pathway	hsa04918	Thyroid hormone synthesis	5/194	75/8163	0.0325676	0.051769306	0.019816002	GSR/PRKCA/HSPA5/PRKCB/DUOX2	5
Kegg Pathway	hsa04916	Melanogenesis	6/194	101/8163	0.032875829	0.051958925	0.019888584	GSK3B/MAPK3/MAPK1/RAF1/PRKCA/PRKCB	6
Kegg Pathway	hsa04810	Regulation of actin cytoskeleton	10/194	218/8163	0.034895375	0.054835589	0.020989699	CHRM3/CHRM1/CHRM2/CHRM5/INSRR/MAPK3/MAPK1/EGFR/EGF/RAF1	10
